# Uncovering natural allelic and structural variants of *OsCENH3* gene by targeted resequencing and in silico mining in genus *Oryza*

**DOI:** 10.1038/s41598-023-28053-w

**Published:** 2023-01-16

**Authors:** Karminderbir Kaur, Kumari Neelam, Jasdeep Singh, Palvi Malik, Kuldeep Singh

**Affiliations:** 1 School of Agricultural Biotechnology, Punjab Agricultural University, Ludhiana, Punjab India; 2 International Crops Research Institute for the Semi-Arid Tropics, Patancheru, Telangana India

**Keywords:** Biotechnology, Genetics, Plant sciences

## Abstract

Plant breeding efforts to boost rice productivity have focused on developing a haploid development pipeline. *CENH3* gene has emerged as a leading player that can be manipulated to engineer haploid induction system. Currently, allele mining for the *OsCENH3* gene was done by PCR-based resequencing of 33 wild species accessions of genus *Oryza* and in silico mining of alleles from pre-existing data. We have identified and characterized *CENH3* variants in genus *Oryza*. Our results indicated that the majority *CENH3* alleles present in the *Oryza* gene pool carry synonymous substitutions. A few non-synonymous substitutions occur in the N-terminal Tail domain (NTT). SNP A/G at position 69 was found in accessions of AA genome and non-AA genome species. Phylogenetic analysis revealed that non-synonymous substitutions carrying alleles follow pre-determined evolutionary patterns. *O. longistaminata* accessions carry SNPs in four codons along with indels in introns 3 and 6. Fifteen haplotypes were mined from our panel; representative mutant alleles exhibited structural variations upon modeling. Structural analysis indicated that more than one structural variant may be exhibited by different accessions of single species (*Oryza barthii*). NTT allelic mutants, though not directly implicated in HI, may show variable interactions. HI and interactive behavior could be ascertained in future investigations.

## Introduction

To overcome limitations in rice productivity, plant breeding efforts have focused on developing a haploid development pipeline. In rice, haploids have been produced primarily through anther culture^1^. Several rice varieties have also been developed and released, particularly in Korea and China. These varieties are of japonica type, which are responsive to anther culture. Indica varieties show recalcitrance upon anther culture; often producing a high frequency of albino plants that eventually die^1,2^. Thus, anther culture is not a genotype-independent method of haploid induction. To be successful, any method of haploid induction should lead to a high frequency of haploid induction while being genotype independent. Wheat × Maize system for obtaining wheat haploids is a perfect example of this^3^. Rice wide crosses with either maize or pearl millet fail to recapitulate this behavior^4^. Chromosome manipulation and elimination of all alien chromosomes in wide hybrids is a pre-requisite to induce a crop haploid. Work on intraspecific and interspecific hybrids has evidenced the experimental links between the loss of CENH3 (Centromere specific Histone 3 variant) and the occurrence of uniparental chromosome elimination^5,6^.

Centromeres control genome inheritance; helping mediate precise movement of chromosomes during cell division by attaching to spindle microtubules via the kinetochore protein complex. The centromere is epigenetically specified by CENH3^7,8^. It replaces canonical H3 in most of the centromere-specific nucleosomes and recruits various kinetochore proteins^9^. In contrast to highly conserved canonical histones, CENH3 is rapidly evolving; the tail domain evolves so rapidly that its sequence can barely be aligned between closely related species^10^. The coming together of genomes loaded with centromeres carrying different CENH3 proteins impedes chromosome segregation during zygotic mitosis. The ‘defective’ CENH3 are selectively removed from centromeres during reproduction, leading to failure of kinetochore assembly onto chromosomes derived from the variant parent^11^. These chromosomes are subsequently lost. Thus, the modified *CENH3* gene acts as a haploid inducer gene^12,13^. It has been reported that progressive natural divergence in CENH3 can cause genome elimination in *Arabidopsis thaliana*^14^. These findings imply that *Arabidopsis* plants having the *cenh3-1* null mutant (embryo lethal) can be complemented with CENH3 from another species or genera to obtain a haploid inducer. Similarly, it has been found that a single point mutation in native CENH3 is sufficient to generate a haploid inducer line^15^.

The *CENH3* gene has been functionally characterized in many agronomically important plant species, including rice^16^. In rice, this gene is located on chromosome 5 (http://rice.plantbiology.msu.edu/, Locus ID: LOC_Os05g41080). The natural variation for this has not been exploited in the *Oryza* species. Evolutionary and expression patterns of CENH3 in a few diploid and allotetraploid species have been evaluated earlier^17^. Protein and nucleotide sequences for CENH3 from various *Oryza* species are available (http://www.uniprot.org/uniprot/; http://www.ebi.ac.uk/ena). Preliminary BLAST analysis of the protein revealed that some of these are identical among species (e.g., *O. latifolia* protein ID E2GIB7_9ORYZ and *O. alta* protein ID D0EKP9_9ORYZ) while others show variation at amino acid level. It is not yet known if this variation may contribute to haploid induction. Uncovering this knowledge can provide leads towards specific structure of *OsCENH3* and how it can be tinkered to obtain CENH3-based HI lines. In the current study, a detailed analysis of sequence variation at the *OsCENH3* locus (LOC_Os05g41080) was performed in silico and in a panel of wild *Oryza* species accessions to identify the evolutionary patterns of *CENH3* alleles. We have identified and characterized *CENH3* variants in genus *Oryza* along with phylogenetic relationships among various *Oryza* species. Our results indicate that the majority *CENH3* alleles present in the *Oryza* gene pool carry synonymous substitutions. A few non-synonymous substitutions where present effect the protein conformation to a certain extent.

## Results

### In silico analysis elucidates that the* OsCENH3* gene has no other paralogs and is under purifying selection within genus oryza

The tBLASTn analysis revealed significant hits on Chromosome 5 in the region 24070193 – 24067694 which coincided with the *OsCENH3* coordinates (Supplementary Fig. S1, Supplementary Table S1)^18^. Similar results were seen upon carrying out Nucleotide BLAST (Supplementary Table S2)^18^. The lack of significant hits on any other part of the genome assembly established that the rice genome, in all likelihood, does not have any paralogs for the *CENH3* gene.

**Figure 1:**
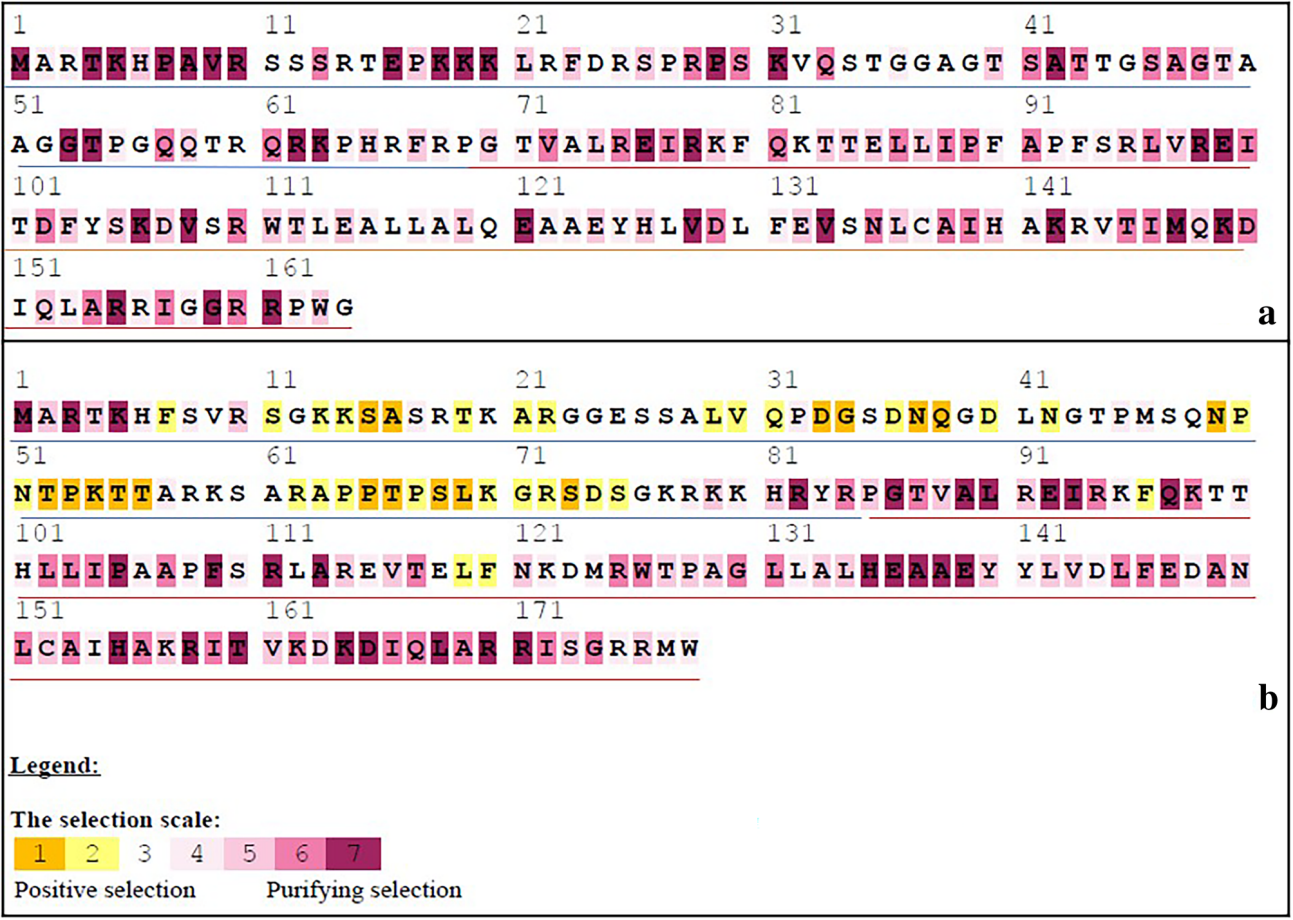
(**a**) Selecton analysis of *Oryza* genus CENH3: The Selecton analysis carried out through selecton server (http://selecton.tau.ac.il/) revealed rice CENH3 protein to be under purifying selection (please refer to the selection scale at the bottom of the figure). (**b**) Selecton analysis of CENH3 from various species: The various residues on comparison with a larger query set show that positive selection is operating at various residues of CENH3 protein(s) in various species. Notably this positive selection acts mainly in the region of N-terminal tail domain. The NTT residues are underlined in blue, starting at residue 1 and HFD starts at the motif PGTVAL; underlined in red.

Selecton analysis of *Oryza* genus CENH3 CDS sequences revealed the protein to be under purifying selection (Fig. 1a)^19^. Varying degree of purifying selection was observed among 164 residues of rice CENH3 protein. Contrastingly, the analysis based on a query set of 98 CDS sequences, many residues of NTT showed positive selection (Fig. 1b). This implied that the CENH3 protein sequences are highly conserved within the *Oryza* genus, while exhibiting some variation in the NTT region among different genera. This could be explained by the fact that different species and genera have a propensity to incorporate certain residues at higher rates than others. NTT is known to be more gapped/unalignable while HFD is highly conserved^15^. This is also in conference with results obtained earlier by other workers^17^.

### Allelic variation in the wild species accessions for the gene *OsCENH3*

Amplification of the germplasm accessions with overlapping primer pairs (Supplementary Table S3, Supplementary Fig. S2) yielded single sharp bands of expected amplicon size (Supplementary Fig. S3, Table 1). The complete allelic sequence of *OsCENH3*, comprised of seven exons (Supplementary Fig. S4), could be sequenced in 25 out of 33 wild *Oryza* accessions and were found to be ~ 2166 bp long. In total, *CENH3* alleles from eight *O. rufipogon* accessions, five *O. nivara* accessions, four accessions of *O. glaberrima*, three of *O barthii*, two each of *O. meridionalis* and *O. longistaminata* and one accession of *O. glumaepatula* could be fully sequenced. For the eight accessions where the complete gene sequence could not be obtained, the sequence region pertaining to HFD was analyzed. SNPs observed in exonic regions and major indels found in introns are listed in Table 2. No indels were found in exons.

**Table 1:** List of wild species accessions selected for allele mining of *OsCENH3* locus.

Field ID	Species (Accession No.)	Genome	Country of origin
W1	*O. glaberrima (IR100983)*	AA	Senegal
W2	*O. glaberrima (IR101800)*	AA	Nigeria
W3	*O. glaberrima (IR102196)*	AA	Liberia
W27	*O. glaberrima (IR102925)*	AA	Burkina Faso
W39	*O. glaberrima (IR103990)*	AA	Tanzania
W44	*O. barthii (IR89146)*	AA	Zambia
W45	*O. barthii (IR100223)*	AA	Guinea
W46	*O. barthii (IR100934)*	AA	Mali
W53	*O. barthii (IR104102)*	AA	Chad
W58	*O. barthii (IR104136)*	AA	Cameroon
W79	*O. nivara (IR80547)*	AA	India
W82	*O. nivara (IR80722)*	AA	Mynamar
W101	*O. nivara (IR104650A)*	AA	Thailand
W103	*O. nivara (IR104688)*	AA	Sri Lanka
W277	*O. nivara (CR100373)*	AA	India
W509	*O. rufipogon (IR80433)*	AA	India
W517	*O. rufipogon (IR80762)*	AA	Mynamar
W522	*O. rufipogon (IR103404)*	AA	Philippines
W525	*O. rufipogon (IR104389)*	AA	Bangladesh
W526	*O. rufipogon (IR104404)*	AA	Thailand
W634	*O. rufipogon (CR100055)*	AA	India
W726	*O. rufipogon (IR93076)*	AA	Cambodia
W776	*O. rufipogon (IR101411)*	AA	Australia
W924	*O. longistaminata (IR81965)*	AA	Zambia
W926	*O. longistaminata (IR101210)*	AA	Ivory Coast
W927	*O. longistaminata (IR104301)*	AA	Gambia
W944	*O. longistaminata (IR86485)*	AA	Botswana
W950	*O. longistaminata (IR92607)*	AA	Mali
W1014	*O. longistaminata (IR105198)*	AA	Ethiopia
W1031	*O. meridionalis (IR101146)*	AA	Australia
W1037	*O. meridionalis (IR93266)*	AA	Indonesia
W1056	*O. glumaepatula (IR100184)*	AA	Cuba
W1120	*O. australiensis (IR105270)*	EE	Australia

**Table 2:** Variations observed in alleles of *CENH3* in wild species of *Oryza*.

Variation	Codon/position (ATG)	Effect	Species
GAA > GAG	23/69	Syn	*O. glaberrima, O. barthii, O. nivara, O. rufipogon, O. meridionalis,*
GGC > GGG or GGT	36/233	Syn	*O. glaberrima, O. barthii, O. meridionalis, O. glumaepatula*
ACG > GCG	38/236	A > T	*O barthii*
GGT > GGG	39/241	Syn	*O. glaberrima, O. barthii, O. longistaminata*
AAG > GAG	61/964	K > E	*O. rufipogon*
AGG > ACG	63/971	R > T	*O. longistaminata, O. meridionalis,*
GCA > GCG	74/1005	Syn	*O. nivara, O. australiensis, O. rufipogon*
ACC > ACT	85/ 1038	Syn	*O. glaberrima, O. barthii, O. nivara,*
TCT > TCC	95/1068	Syn	*O. longistaminata*
TGC > TGT	138/1728	Syn	*O. longistaminata*
GCA > GCT	139/1731	Syn	*O. meridionalis, O. glumaepatula*
GCC > GCT	155/ 2136	Syn	*O. longistaminata*
ATC > ATT	158/2145	Syn	*O. longistaminata*
Indel in intron 3	(39 bases)	–	*O. longistaminata*
Indel in intron 6	(38 bases)	–	*O. longistaminata*

**Figure 2:**
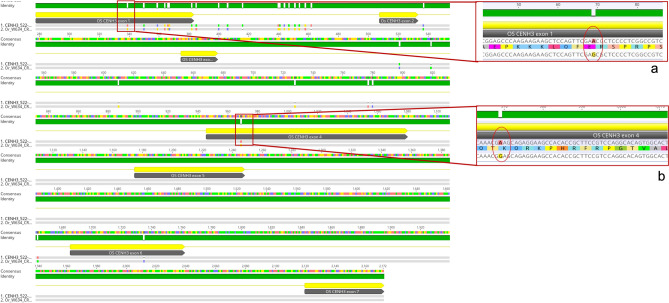
Alignment window of *O. rufipogon* accession CR100055 showing (**a**) a SNP at position 69/codon 23 in exon 1 of the *CENH3* gene when compared to *OsCENH3* reference. The SNP is highlighted in red circle. This variation is found in many other AA genome species accessions as well. (**b**) Non-synonymous substitution found in *O. rufipogon* accession CR100055 when aligned to reference at position 964/codon 61. Please also refer to Table 2. (The alignment was carried out using Geneious Prime version 2021.1.1; images were generated from Geneious Prime version 2021.1.1itself).

**Figure 3:**
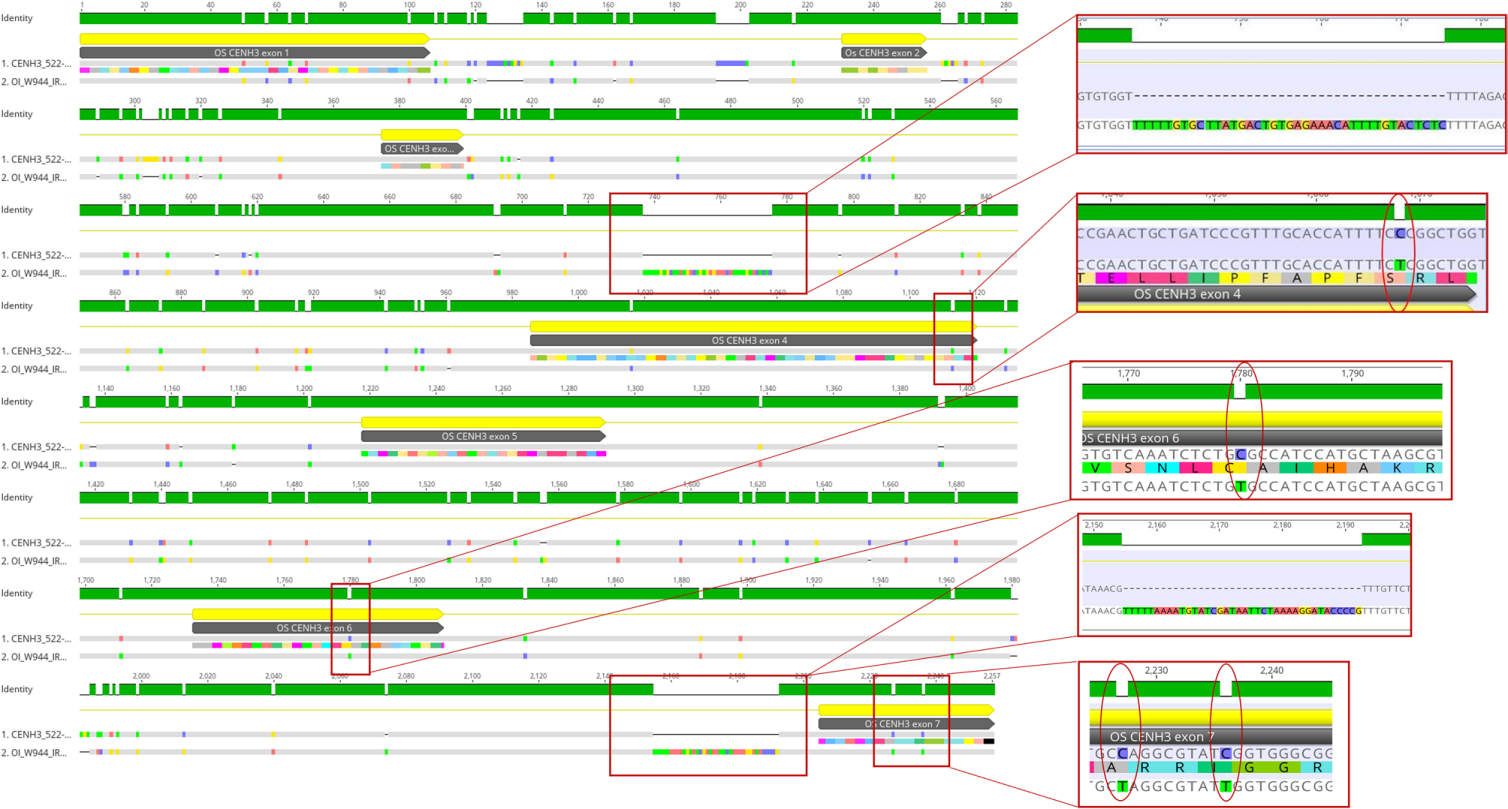
Variations seen solely in *O. longistaminata* accessions when compared to reference (**a**) Indel found in Intron 3, (**b**) SNP at position168/codon 95, (**c**) SNP at position 1728/codon 138, (**d**) Indel in intron 6 and (**e**) SNPs at codons 155 and 158. Please also refer to Table 2. (The alignment was carried out using Geneious Prime version 2021.1.1; images were generated from Geneious Prime version 2021.1.1 itself).

Please note that SNP positions are noted in reference coordinates and may be seen differently in Fig. 2 and 3 due to presence of indels between exons.

Notably, various SNPs were found across genus *Oryza* irrespective of species e.g., SNP A/G at position 69 was found in accessions of AA genome species (*O. glaberrima, O barthii, O. nivara, O rufipogon, O. longistaminata, O. glumaepatula* and *O. meridionalis*) as well as non-AA genome species (*O. australiensis)* (Fig. 2a). Some accessions of the progenitor species exhibited no SNP at this position compared to *OsCENH3* which indicated that the actual progenitor of the present-day cultivated rice might have carried this version of the gene. It is interesting to note that synonymous SNPs seen in *O. glaberrima* are also present *O. barthii* (Table 2). Non-synonymous SNPs were found to occur in NTT region only (Fig. 2b). Based on sequence data available, *O. nivara*, *O.meridionalis*, *O. glumaepatula* and *O. australiensis* (Incomplete sequence in *O. australiensis*) do not carry any non-synonymous SNPs (Table 2). Therefore, different species exhibit different alleles at nucleotide level but amino acid changes are few and limited to NTT.

### Species specific variation in *OsCENH3* alleles in genus* Oryza*

A few of the SNPs or Indels were observed to be species specific (Table 2). *O. longistaminata* accessions carried SNPs not seen in other *Oryza* species accessions under study. This included SNPs in codons 95, 138, 155 and 158 as well as indels in introns 3 and 6 (Fig. 3). In a similar trend, SNP at position 1731 (Codon 139) was observed in only *O. meridionalis* and *O. glumaepatula* accessions (Table 2). At position 233, *O. glaberrima* accessions carried base G in place of C in the reference while *O. nivara* as well *O. meridionalis* accessions had a T nucleotide. Therefore, the differences among various species, arose from major indels in introns (seen in *O. longistaminata* consistently), or different SNPs at the same location.

### Haplotype detection

Sixteen haplotypes (Table 3, Supplementary Table S4) were detected upon manual curation of SNP data (complete genic sequences or sequences for which sequence data for all exons and relevant introns was available were used for this). All the 15 haplotypes were comparable to one another at one position or the other. Thus, a single SNP may be part of several different haplotypes based on what other SNPs it co-exists with, in different accessions. The accessions belonging to same species tend to have a similar haplotype structure.

**Table 3:** Haplotypes based on SNPs from complete *CENH3* genic data: the table reports SNP in the affected codons (columns) for sixteen haplotypes (rows).

SNP/ Haplotype	Codon 23	Codon 36	Codon 38	Codon 39	Codon 61	Codon 63	Codon 74	Codon 85	Codon 95	Codon 138	Codon 139	Codon 155	Codon 158	Indel intron 3	Indel intron 6
*OsCENH3*	GAA	GGC	ACG	GGT	AAG	AGG	GCA	ACC	TCT	TGC	GCC	GCC	ATC	–	–
H1	GAG	GGG	ACG	GGT	AAG	AGG	GCA	ACT	TCT	TGC	GCC	GCC	ATC	–	–
H2	GAG	GGG	ACG	GGG	AAG	AGG	GCA	ACC	TCT	TGC	GCC	GCC	ATC	–	–
H3	GAG	GGC	ACG	GGT	AAG	AGG	GCA	ACT	TCT	TGC	GCC	GCC	ATC	–	–
H4	GAG	GGG	ACG	GGG	AAG	AGG	GCA	ACT	TCT	TGC	GCC	GCC	ATC	–	–
H5	GAG	GGG	GCG	GGT	AAG	AGG	GCA	ACT	TCT	TGC	GCC	GCC	ATC	–	–
H6	GAG	GGT	ACG	GGT	AAG	AGG	GCA	ACT	TCT	TGC	GCC	GCC	ATC	–	–
H7	GAG	GGT	ACG	GGT	AAG	AGG	GCG	ACC	TCT	TGC	GCC	GCC	ATC	–	–
H8	GAG	GGC	ACG	GGT	GAG	AGG	GCA	ACT	TCT	TGC	GCC	GCC	ATC	–	–
H9	GAG	GGC	ACG	GGT	AAG	AGG	GCG	ACT	TCT	TGC	GCC	GCC	ATC	–	–
H10	GAA	GGC	ACG	GGT	GAG	AGG	GCA	ACT	TCT	TGC	GCC	GCC	ATC	–	–
H11	GAA	GGC	ACG	GGT	AAG	AGG	GCG	ACT	TCT	TGC	GCC	GCC	ATC	–	–
H12	GAG	GGC	ACG	GGT	AAG	ACG	GCA	ACT	TCC	TGT	GCC	GCT	ATT	39	38
H13	GAG	GGC	ACG	GGT	AAG	ACG	GCA	ACT	TCC	TGT	GCC	GCC	ATC	39	38
H14	GAG	GGT	ACG	GGT	AAG	AGG	GCA	ACT	TCT	TGC	GCA	GCC	ATC	–	–
H15	GAA	GGC	ACG	GGT	AAG	AGG	GCA	ACT	TCT	TGC	GCC	GCC	ATC	–	–
H16	GAG	GGC	ACG	GGT	AAG	AGG	GCA	ACC	TCT	TGC	GCC	GCC	ATC		

Haplotype structure varied mostly amongst species but sometimes also within the species. Within the species, these differences were attributable to at most one or two SNPs, that in most cases did not lead to any amino acid changes. Thus, different nucleotide haplotypes could be reduced to a single protein sequence. For instance, haplotypes H8 and H10 translate to identical protein sequence. Similar is the case with H12, H13 and H14.

### Phylogenetic relationships of *OsCENH3* gene among diverse wild species germplasm of rice

**Figure 4:**
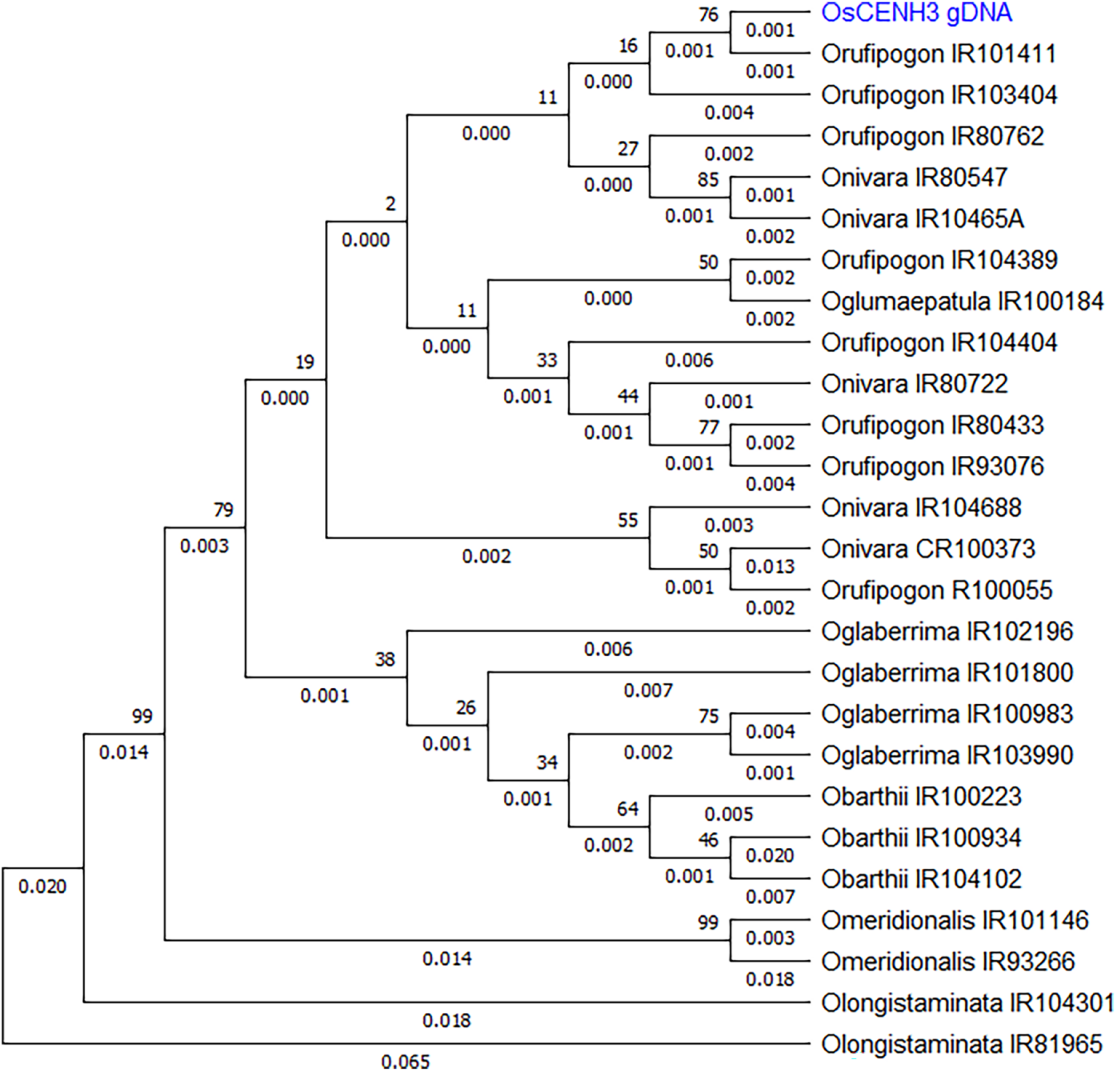
Phylogenetic analysis of sampled *CENH3* alleles: Dendrogram generated using complete *CENH3* sequences of 25 Oryza accessions in MEGA version X at bootstrap 1000 using neighbor-joining method. Accessions as listed in Table 1. Reference clustered closest to *O. rufipogon* and *O. nivara* accessions.

**Figure 5:**
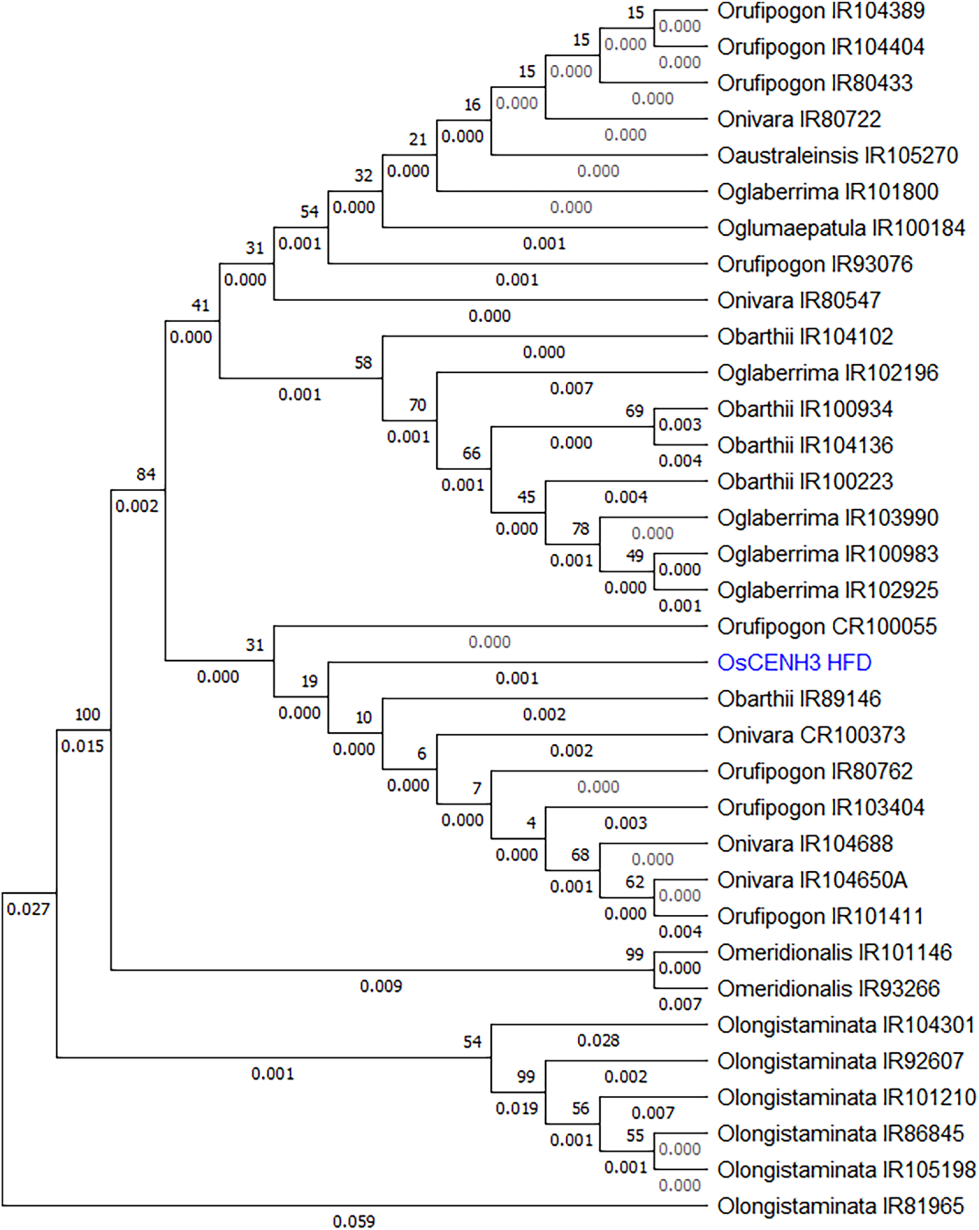
Phylogenetic analysis limited to HFD domain of sampled *CENH3* alleles: Dendrogram generated using only *CENH3* HFD region sequences of 33 accessions in MEGA version X at bootstrap 1000 using the neighbour-joining method. Accessions as listed in Table 1.

**Figure 6:**
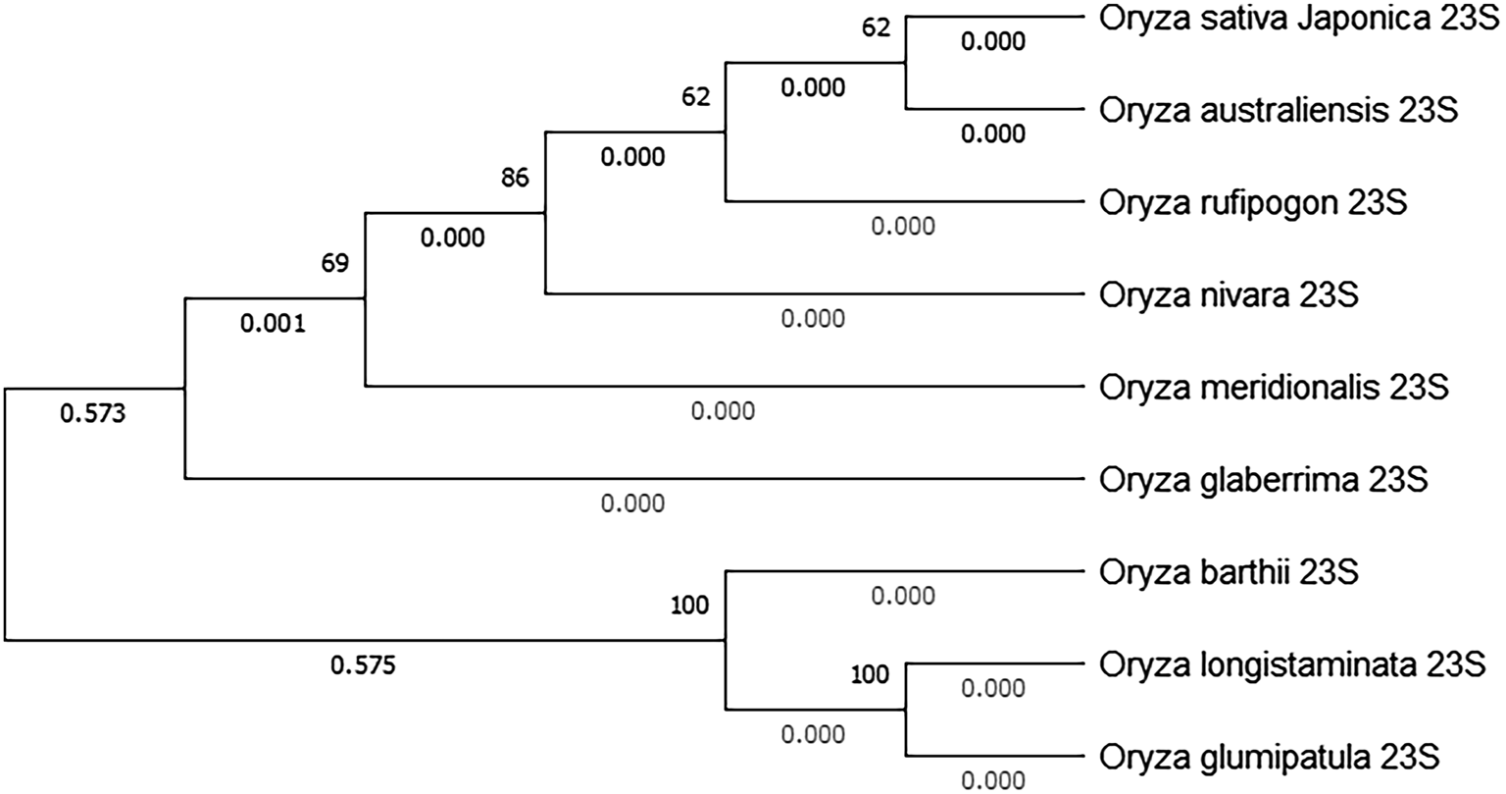
Dendrogram constructed from 23S rRNA sequences of various *Oryza* species: *O. rufipogon* and *O. nivara* cluster close to cultivated species along with *O. australiensis*. *O. longistaminta* and *O. glumaepatula* are the most divergent.

Upon phylogenetic analysis of complete *CENH3* sequences, the accessions clustered into four major clades (Fig. 4). Upon analysis limited to just the Histone Fold Domain (HFD), two major clades were formed (Fig. 5) and the African species *O. barthii* and *O. glaberrima* formed a distinct subclade. *CENH3* from *O. rufipogon* accessions cluster closer to *OsCENH3* reference when analyzed with complete gene sequences as well as with only HFD (Figs. 4 and 5). The other species were distant depending upon their genome group. *O. meridionalis* and *O. longistaminata* are the most distant among the species under study in both cases. These differences though were not reflected in protein sequences as no major amino acid substitutions were seen, except for in NTT region of *O. rufipogon* and *O. longistaminata*. The *O. rufipogon* accessions (CR100373, IR80762, IR103404 and CR100055) that exhibited amino acid changes (though these were not expected to lead to functional changes in CENH3 behavior) still cluster close to *OsCENH3*. For comparison of evolutionary patterns exhibited by *CENH3* sequences to those found at species level, a dendrogram of 23S ribosomal RNA from different *Oryza* species was also constructed (Fig. 6). In addition to *O rufipogon*, *O. australiensis* 23S rRNA clustered closest to *O sativa subspecies japonica*. *O. longistaminata* and *O. glumaepatula* were the most divergent*.* It is to be noted that *O. glumaepatula CENH3* allele in contrast clusters close to cultivated species reference as well as *O. rufipogon.*

### In silico HFD mining of protein sequences from *Oryza* genus

The protein length varies from 146 to 170 residues in different *Oryza* species (Table 4); the insertions and deletions leading to length variation being present in the NTT region. For almost all AA species (except *O. barthii*), CENH3 protein is composed of 164 residues. Ten haplotypes were seen in *Oryza* genus upon aligning and analyzing entire protein length (Table 4). In these ten haplotypes, the major differences arise from changes in NTT, while HFD in many variants is identical. Upon limiting the analysis to just the HFD, five variant types were observed (Table 4). It is noteworthy that *O. meyeriana* carries the CENH3 variant (A0A6G1EGA2) with most changes (ten) in the HFD region when compared to the reference protein Q6T367 (*Oryza sativa ssp japonica*), which can be attributed to the fact that it belongs to the tertiary gene pool. Interestingly, eight out of these ten SNPs were not observed in any other species. The most common changes (P70S and L117I) appear to always co-exist and were observed only in the CC genome. Another set of amino acid substitutions (I131L and M152I) also co-exist and were seen to occur in BB, DD as well EE genome. AA genome species do not exhibit any changes in the HFD region, indicating that the CENH3 has remained functionally conserved in the primary gene-pool of the genus *Oryza*. Curiously, the *O. barthii* CENH3 sequence on alignment with other AA-genome CENH3 sequences was revealed to be missing exon 2 completely. Resequencing of *O. barthii CENH3* alleles from our panel revealed one accession to carry a SNP in beginning of exon 2 which we inferred to lead to an amino acid substitution. This anomaly is not seen in any other species including *O glaberrima* which is closely related to *O. barthii*.

**Table 4:** Haplotype characterization of Oryza genus CENH3 protein sequences based off their alignment with rice CENH3: Haplotypes were mined on basis of variations observed in protein sequences when compared to reference.

Species	Accession number	Genome	Protein length (amino acid residues)	e-value	%identity	SNPs observed in HFD	Haplotype	Haplotype on basis of HFD
*Oryza alta*	D0EKP8	CCDD	164	1.90E−100	90.9	I131L, M152I	Hα	H-I
*Oryza alta*	D0EKP9	CCDD	166	1.00E−104	93.4	P70S, L117I	Hβ	H-II
*Oryza australiensis*	D0EKQ2	EE	164	4.40E−91	88.5	I131L, M152I	Hγ	H-I
*Oryza barthii*	A0A0D3G978	AA	146	3.40E−97	89	none	Hδ	H-III
*Oryza glumipatula*	A0A0E0A0Z6	AA	164	1.80E−114	100	none	Hε	H-III
*Oryza latifolia*	E2GIB7	CCDD	166	1.10E−104	93.4	P70S, L117I	Hβ	H-II
*Oryza latifolia*	E2GIB8	CCDD	164	1.90E−100	90.9	I131L, M152I	Hα	H-I
*Oryza meridonalis*	A0A0E0DTC0	AA	164	1.80E−114	100	none	Hε	H-III
*Oryza meyeriana vra. Granulata*	A0A6G1EGA2	GG	146	1.90E−91	82	K80R, T84S, D108E, S110T, L114Y, L118V, I131L, M152I, R162H, P163S	Hζ	H-IV
*Oryza minuta*	DOEKQ0	BBCC	166	3.84E−103	92.2	K83R	Hη	H-V
*Oryza minuta*	D0EKQ1	BBCC	166	9.24E−104	92.8	P70S, L117I	Hθ	H-II
*Oryza nivara*	A0A0E0HG58	AA	164	1.80E−114	100	none	Hε	H-III
*Oryza officianalis*	E2GIB6	CC	166	9.24E−104	92.8	P70S, L117I	Hθ	H-II
*Oryza punctata*	D0EKQ3	BBCC	166	2.20E−102	96.6	K83R	Hκ	H-V
*Oryza punctata*	A0A0E0L436	BBCC	170	6.40E−87	91.3	K83R	Hλ	H-V
*Oryza rufipogon*	A0A0E0PP98	AA	164	1.80E−114	100	none	Hε	H-III
*Oryza rhizomatis*	D0EKQ4	CC	166	9.24E−104	92.8	P70S, L117I	Hθ	H-II
*Oryza sativa ssp indica*	B8AZH6	AA	164	1.80E−114	100	none	Hε	H-III

We found synonymous as well non-synonymous SNPs upon resequencing *CENH3* gene in primary gene pool (AA-genome species) of genus *Oryza*. Additionally, sequencing of the single *O. australienesis* (EE) accession did not yield any SNPs. In contrast, the *in-silico* mining of the protein sequences belonging to species from secondary and tertiary gene pools exhibited several amino acid changes specific to genome groups. Most allotetraploid species belonging to the *O. officianalis* complex (secondary gene pool) exhibit at most two changes in HFD. It could also be inferred that various allotetraploid species carry the same CENH3 complement E.g., In *O. alta* (CCDD) *and O. latifolia* (CCDD), both the CENH3 copies are identical (D0EKP8 = E2GIB8, D0EKP9 = E2GIB7). Similarly, *O. rhizomatis* (CC) and *O. officianalis* (CC) CENH3s showed 100% identity.

### Mining allelic variants in HDRA and SNPSeek panels

Our analysis of HDRA and SNPseek panels returned a total of nine *OsCENH3* variants (Supplementary Table S5)^20–22^. The HDRA variant allele (rs# 5655822) was found to have a synonymous effect. Of the eight variants mined from SNPseek, only one (rs#175193154) occuring in exon 6, was found to have non synonymous effect (Cysteine to Serine). Of the remaining seven, six were intronic in nature while one was placed in 3′ UTR. rs#175193154 was employed for homology modelling and alignment.

### Homology modelling and structural alignment

Variant secondary structures exhibit differences from the structure of the reference OsCENH3 (Supplementary Fig. S5a–g). The OsCENH3 secondary structure presents six helices with one helix present in NTT. The haplotype variants H5 and H8 also have six helices but also carry a two-residue strand not seen in the reference. H12 variant has six helices in the regions comparable to the reference but these helices differ in length, a similar case is presented by rs#175193154 (Supplementary Fig. S5h). *O. barthii* variant Hδ and *O. officinalis* variant Hθ carry only four helices, all of them comparable to corresponding regions in OsCENH3 HFD and amongst themselves. *O. meyeriana* CENH3 protein (Hζ) shows five helices.

**Figure 7:**
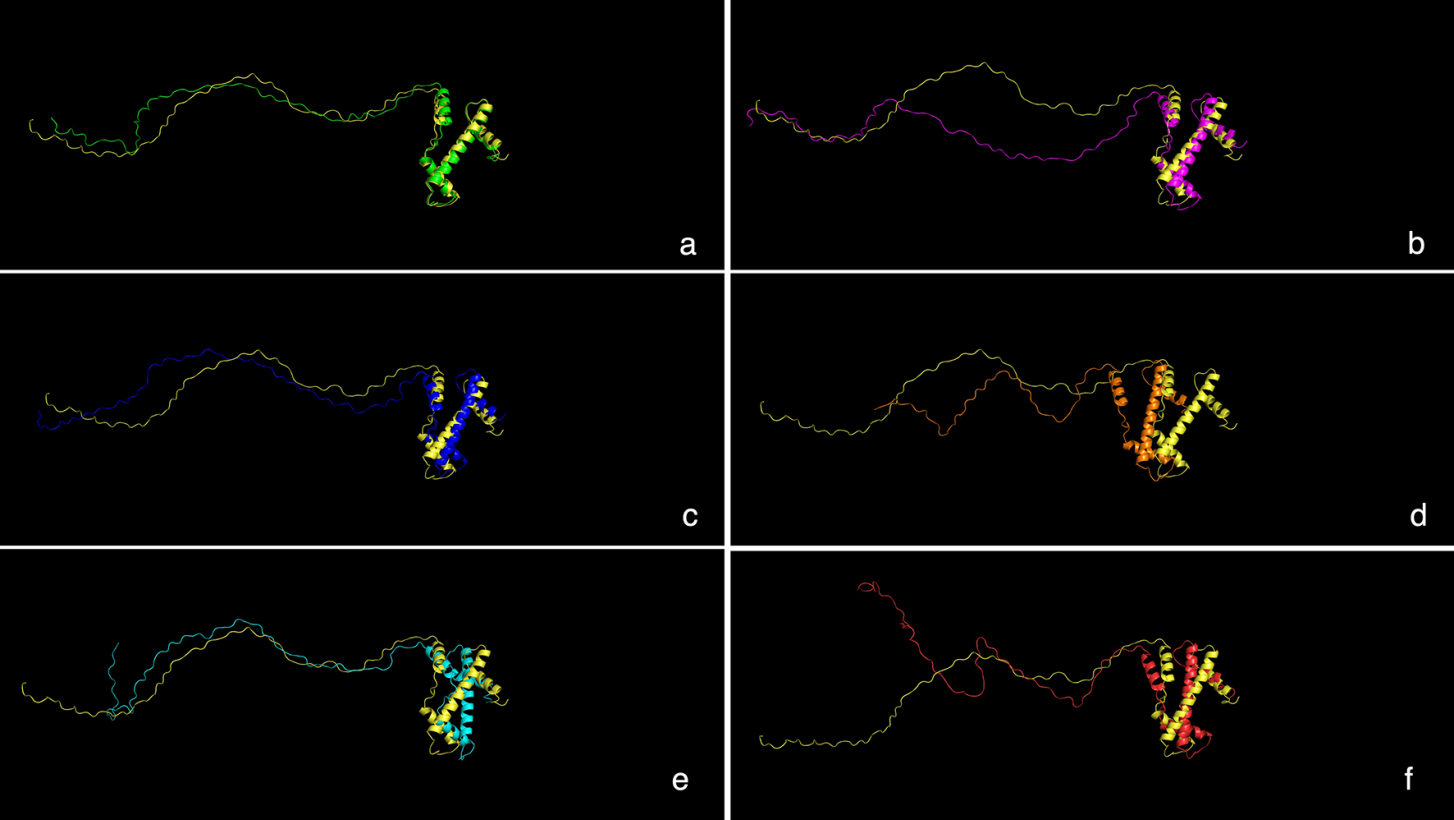
Comparison of 3D models of *Oryza* genus CENH3s: Superimposition of reference OsCENH3 (yellow) with (**a**) H5, (**b**) H10, (**c**) H12, (**d**) Hδ, (**e**) Hθ, and (**f**) Hζ. The models were generated using Modeller 10.1 and superimposed using PyMOL version 2.5.0.

To better understand structural differences, 3D modelling was carried out. The human nucleosome structure containing the histone variant H3.2 - 3AV1_A exhibited highest percent identity and was employed as template for homology modelling of the reference (Supplementary Fig. S6). Five models were built for each sequence respectively and the model with minimum DOPE score (Discrete Optimized Protein Energy) was selected (Supplementary Table S6). Protein structures for OsCENH3 and of variant representatives H5, H10, H12 as well as Hδ, Hζ and Hθ (Fig. 7) as well as rs#175193154 were superimposed (Supplementary Fig. S7) and analyzed for structural differences. All models showed > 90% residues in most favored region (Supplementary Figs. S8, S9 and S10). The protein structure of OsCENH3 displayed 4 helices (αN Helix, α1 Helix, α2 Helix and α3 Helix) and two loops (loop1 and loop2), loop1and α2 Helix define CATD (Supplementary Fig. S11), characteristic of CENH3 structure as studied by various workers in different species^15,23–27^.

**Figure 8:**
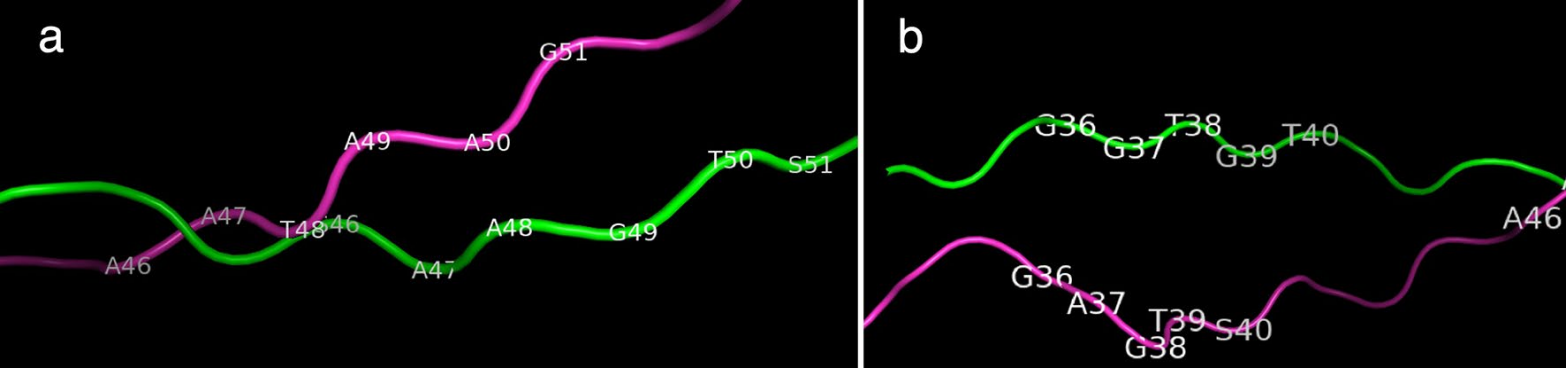
Variations in OsCENH3 (green) and Hθ (pink): (**a**) an extra triplet of TAA amino acids occurs from position 48–50 in Hθ, (**b**) Thr 38 is absent. Despite these facts, the helical structure is unaltered. The models were generated using Modeller 10.1 and superimposed using PyMOL version 2.5.0.

The comparison of 3D structures showed that H5 and H10 are very similar to OsCENH3 with RMSD (Root mean square deviation) of 2.704 and 8.330 respectively (Supplementary Table S7). No major changes in the overall architecture of proteins were observed. The position 71 in H5 (red) was part of helix while it is not in OsCENH3 (green) (Supplementary Fig. S12). All the helix and loops in H12 and H10 were comparable to OsCENH3. Gly 37 to Gly 54 was deleted (disordered region) in Hδ *(O. barthii*) but still all helices and loops are conserved. In Hθ (*O. officinalis*), Thr 38 was absent (purple) and an extra triplet of TAA amino acids is found at position 48–50, still the loops and helices were conserved (Fig. 8). In *O. meyeriana* (Hζ), the helix regions were identical but some variation existed in the loops, e.g., Gly 27 to Pro 31 region does not occur as loop in OsCENH3. Like Hθ, Thr38 was missing in Hζ. An extra loop spanning tripeptide Trp 52 to Ala 54 was also seen in Hζ. Although, the wild relatives exhibit natural variants of *OsCENH3* but the mutations (SNPs or Deletions) fail to disrupt the major structural components. Additionally, the rs#175193154 exhibited complete superimposition with reference OsCENH3 (DOPE score: − 10105.18555).

## Discussion

CENH3 mutations have been profiled extensively in *Arabidopsis thaliana*^15,28,29^. A corresponding resource does not exist for rice CENH3 mutants. Most of the allelic variation at any given locus is expected to occur in the wild germplasm and not the crop itself, due to the unavoidable loss of variation during domestication^30–32^. In the current study, we have scanned the *Oryza* germplasm accessions for *CENH3* variants with simultaneous in silico analysis. This study underlines that the *OsCENH3* gene is a highly conserved, single copy gene. Contrastingly, barley, wheat and rye have two functional *CENH3* genes: α*CENH3* and β*CENH3*^6,33,34^. Recent reports in maize suggest that it is easier to manipulate haploid induction in genomes carrying single copy of *CENH3*^26^.

### Wild germplasm presents synonymous as well as non-synonymous variations

To maximize chances of identifying variant alleles, the allele mining subset was selected using “P” procedure^35^; while also considering the original geographical distribution of the accessions. Despite having distinctive morphology, life-cycle duration and pollination system, the amino acid sequences of CENH3 in the *Oryza* accessions are highly similar. Synonymous changes outnumber non-synonymous variations at the level of the DNA. The absence of non-synonymous changes across HFD in *CENH3*s of genus *Oryza* is explained by earlier findings wherein multiple sequence alignment has shown that HFD amino acid sequence is similar even among species belonging to different genera^15^. The *in-silico* analyses of the *OsCENH3* gene carried out as part of the present study also indicated in the same direction, in coherence with results obtained in rye^34^.

On aligning *Arabidopsis thaliana* and rice CENH3 protein sequences, various motifs were seen to be conserved between these two species (Supplementary Fig. S13) which was in coherence with the present investigation as well as previous findings^15^. This could be attributed to the fact that CENH3 like other histones, has certain specific motifs that are indispensable to its function. The MARTKH and PGTVAL motifs, that mark the beginning of NTT and HFD respectively in CENH3 protein and thus define its identity, are seen invariably in all species’ sequences used for alignment by Kuppu et al.^15^. While mining alleles for *OsCENH3* in wild species germplasm of *Oryza* genus, not all accessions could be amplified uniformly. Similar problems were faced by other workers while mining alleles for specific genes from *Oryza* germplasm^36,37^. Transferability of the primer pairs between species belonging to the same genus, is not always 100% as observed in *Passiflora* and *Allium*^38,39^.

L117I was one of the most common single-point amino acid change observed on in silico analysis of *Oryza* genus CENH3 protein sequences. Its corresponding mutant in *A. thaliana*, L130F/ L130I, has been reported to successfully complement *cenh3* null mutant with haploid inducing abilities (HI, 4.8%)^29^. Arabidopsis CENH3 mutants P82S (corresponding to P70S) and A127V (corresponding to L114Y) also exhibit HI capabilities^15^. Notably, Arabidopsis CENH3 variant with A127T is non-complementing^15,28^.

A total of 157 single-point amino acid substitutions (arising from nucleotide transitions) are possible in *OsCENH3*-HFD (unpublished data), but only a handful of these were seen to exist naturally in *Oryza* relatives and none in closely related- AA genome species. The AA genome species might still be carrying SNPs that are not reflected upon translation as seen on resequencing a subset of accessions for *OsCENH3*. Similar studies in banana (*Musa* spp.) and carrot (*Daucus* spp.) also noted highly similar *CENH3s* between different species within the genus^40,41^. Our structural analyses point out that these natural variants do not exhibit major variations in conformation.

Various studies have etched out specific roles played by certain important residues found in NTT region of CENH3 in different species^42–44^. Arginine residues seem to play important role in kinetochore recruitment and subsequent interactions^42,43^. Lysine residue in HFD and Serine residue in NTT have also been implicated in controlling certain functions/behaviours^44,45^. In our analysis, we came across variants in which arginine and lysine residues had been replaced by other amino acids. These variants can be analyzed further for their interactions during kinetochore recruitment. Another allelic variant discovered from SNPseek panel has a serine residue replacing cysteine, and this might also modulate HI behavior of the accessions carrying these.

### Phylogenetic implications of resequencing and in silico analysis

Phylogenetic relationships among different species of genus *Oryza* have been studied based on entire genomes, chloroplast genome and individual genes by various workers previously. Analysis of various nuclear genes established *O. meridionalis* to be most divergent among AA genome species^46,47^. Current study found *CENH3* alleles of *O.longistaminata* to be most divergent from *O. sativa* reference and other species. This could be explained by presence of two long intronic indels in *CENH3* sequences pertaining to *O.longistaminata*. This pattern is also represented to an extent by 23S rRNA of these species (Fig. 6). Interestingly, *O. australiensis* (EE genome) HFD sequence clusters with *O. nivara* and *O. rufipgon*. This is understandable considering that the 23S rRNA originating from *O. australiensis* also exhibited the same pattern, in addition to being closest to cultivated species. This can also be explained by the fact that *Adh1* sequences sourced from various diploid species of genus *Oryza* were found to be identical^47^.

Currently, we sampled eight accessions of *O. rufipogon* and five accessions of *O. nivara* that capture the geographical diversity of the two species. Phylogenetic analyses show that they are scattered in two subclades and overlap with each other as well as *O. sativa* reference, which elucidates their close genetic relationships. On both phylogenetic trees, *O. rufipogon* and *O. nivara* accessions do not cluster distinctly at the species level. This is identical to findings of Zhu and Ge^47^ but in contrast to results obtained by Wambugu et al. ^48^ who observed that even *O. rufipogon* accessions segregate into “Asian” and “Australian” clusters. Currently, the African species *O. barthii* and *O. glaberrima* form a distinct cluster which was also seen upon analysis of chloroplast sequences^48^.

Currently sequenced variant alleles from *Oryza* species germplasm exhibit amino acid changes in region of N-terminal tail domain. Histone fold domain does not exhibit any functional SNPs and thus any variants that might be useful towards haploid induction pipeline were not present in our allele mining panel. The single copy *OsCENH3* gene could be easily manipulated for creating mutants that can then be tested for HI trait. The variants found in wild species germplasm along with other OsCENH3 mutants sourced from our mutant garden are under analysis in our lab for their HI capabilities.

## Materials and methods

### Preliminary in silico characterization of *OsCENH3* gene

Nucleotide and protein sequences of *CENH3* were retrieved from NCBI (https://www.ncbi.nlm.nih.gov/) and UniProt (https://www.uniprot.org), respectively. Of 894 nucleotide entries found on NCBI, the complete coding sequences and protein sequences were retrieved for 286 entries only. Further, 111 sequences (out of 419 returned upon keyword search) were downloaded from UniProtKB. Redundancy was removed from both nucleotide and protein sequence files using CD-HIT suite with identity cut-off of 0.99, leaving behind 107 nucleotide, 106 CDS derived protein sequences and 89 protein sequences from UniProtKB. The non-redundant nucleotide and protein sequences were subjected to domain analysis using CD search at NCBI server. Any sequences with domains other than pfam00125 (histone-specific domain) were not considered further. The two protein sequence files were combined and redundancy check completed again, returning 144 non-redundant protein sequences. Thus, 144 protein and 107 nucleotide sequences were used for investigating any *OsCENH3* paralogs. The sequences were BLAST searched^18^ (BLAST + version was used;^49^) against the complete rice genome assembly version 7.0^50^, retrieved from the RGAP database (ftp://ftp.plantbiology.msu.edu/pub/data/Eukaryotic_Projects/o_sativa/annotation_dbs/pseudomolecules/version_7.0/all.dir/). Standalone BLASTn and tBLASTn searches were performed using the rice genome assembly as database and the *CENH3* CDS and protein FASTA files as the query sequences. In a separate analysis, effect of amino acid substitution in the CENH3 sequences was analysed using Selecton server (http://selecton.tau.ac.il/)^19^.

### Natural allelic diversity for *CENH3* gene in wild species of rice

#### Plant materials

A set of 33 wild species accessions representing eight diploid wild species of genus *Oryza* viz. *O. rufipogon* (n = 8), *O. nivara* (n = 5), *O. glaberrima* (n = 5), *O. barthii* (n = 5), *O. glumaepatula* (n = 1), *O. longistaminata* (n = 6), *O. meridionalis* (n = 2)*, O. australienesis* (n = 1) was used for the present study. These accessions were initially procured from International Rice Research Institute (IRRI), Philippines and National Rice Research Institute (NRRI), Cuttack, India and are being actively maintained at Punjab Agricultural University, Ludhiana. Standard agronomic practices were followed to raise the crop, as reported previously^51^. Voucher specimens for all accessions used currently have been deposited in the herbarium of National Bureau of Plant Genetic Resources (NBPGR), New Delhi, India. These accessions are part of the global and national germplasm collections maintained by IRRI (identifiable by IRGC accession numbers) and NRRI (identifiable by CR accession numbers) and can be accessed through these institutions as well as NBPGR. Initial identification of these materials was carried out by scientists of IRRI and NRRI undertaking germplasm collection. Experimental research and field studies on these accessions including the collection of plant material, were in accordance with the relevant institutional, national, and international guidelines and legislation. Since the plant material has been maintained by PAU, India, permissions regarding the collection of seed specimens were not required.

#### Primer designing and PCR amplification

Genomic DNA was isolated using previously reported protocol^36,52^. The complete sequence of chromosome 5 was retrieved and aligned with gene sequence obtained from RGAP database (RGAP Locus ID: LOC_Os05g41080, http://rice.plantbiology.msu.edu/index.shtml) using offline BLAST and further trimmed to obtain gene sequence flanked by additional 267 bp before the start codon and 467 bp after stop codon, yielding a sequence of 2900 bases. This sequence was used to design three sets of overlapping primer pairs (Supplementary Table S3). Supplementary Figs. S1, S4 show structure of the gene and Supplementary Fig. S2 shows position of primers along the length of the gene. PCR was performed in a 30 μl reaction mix containing 0.25 μl TaKaRa Ex-Taq DNA polymerase, 1 μl of genomic DNA (100 ng/μl), 3 μl of 10X TaKaRa Ex-Taq buffer, 3 μl of dNTPs (1 mM), 1.5 μl each of forward and reverse primers (5 μM), and 19.75 μl Nuclease Free Water. The thermal cycling conditions were as follows: an initial denaturation at 94 °C for 5 min; 35 cycles of 1 min denaturation at 94 °C followed by 45 s annealing at 55 °C and 1 min extension at 72 °C; and a final 7 min extension at 72 °C. Detailed protocols may be found in^52^.

#### Sequencing of *OsCENH3* gene in selected accessions

5 μl PCR product for each sample was electrophoresed on the ethidium bromide stained 1.0% agarose gel along with 1 kb plus ladder (Thermo Scientific Generuler) to estimate the DNA fragment size. The Wizard® SV PCR Clean-Up System (Promega, USA) as per the manufacturer’s protocol was followed to purify the DNA fragments from remaining 25 μl PCR product. The nucleotide sequence information of the PCR products was generated as described previously^51^.

#### Analysis of the generated nucleotide sequences

For comparative sequence analysis, contigs were assembled from individual reads produced by overlapping primers, using DNA Baser Assembler v5.15.0., to generate the contiguous sequence of *OsCENH3* alleles from selected genotypes. ClustalX 2.0.11 was employed to align contig sequences individually with the reference (RGAP Locus ID: LOC_Os05g41080) and to trim the sequences at both ends to retain only the genic portion (i.e., from ATG to TGA). Sequences were also trimmed to retain just HFD or individual exons.

Pairwise alignments and multiple sequence alignments of the trimmed sequences with annotated version of the reference were carried out using Geneious Prime version 2021.1.1. Based on these alignments, SNPs and haplotypes were predicted. The detected SNPs were then manually curated by analyzing and comparing chromatogram files to the Geneious alignment files and DNA Baser contig files. Effects of detected SNPs in terms of coded amino acids, were also visualized using Geneious Prime version 2021.1.1.

#### Phylogenetic analysis

The MEGA (version X) software was used to generate the phylogenetic trees using multiple sequence alignment file^53^. The evolutionary distances were computed using the Neighbor Joining Method with 1,000 bootstraps using the Kimura2-parameter model.

#### In silico mining of *OsCENH3* alleles

The *OsCENH3* alleles from wild *Oryza* species not represented in the resequencing panel, were mined in silico on UniProt server. Eleven protein sequences (D0EKP8, D0EKP9, D0EKQ2, A0A0D3G978, A0A0EA0Z6, E2GIB7, E2GIB8, A0A0EDTC0, A0A6EGA2, DOEKQ0, DOEKQ1, A0A0E0HG58, E2GIB6, D0EKQ3, A0A0E0L436, A0A0E0PP98, D0EKQ4, B8AZH6 and Q6T367) were retrieved using search “CENH3 AND *Oryza*”. Q6T367 is the CENH3 protein from *O. sativa ssp. japonica* pertaining to gene ID Os05g0489800 which is a RAP-DB (https://rapdb.dna.affrc.go.jp/) equivalent of RGAP Locus ID: LOC_Os05g41080 and was thus used as the reference for pairwise alignment of the other 19 sequences using blastp at UniProt server. The alignments were studied to mine SNPs in HFD.

Additionally, SNP calling was carried out on SNPSeek and HDRA panels in the region limited to *OsCENH3* coordinates. Effects of detected SNPs in terms of coded amino acids, were also visualized using Geneious Prime version 2021.1.1.

#### Structure analysis of variants

Representative variant protein sequences along with OsCENH3 reference were subjected to secondary structure prediction using PsiPred^54,55^. Further, OsCENH3 protein sequence was subjected to blastp search against the PDB database at NCBI (https://blast.ncbi.nlm.nih.gov/Blast.cgi) for identification of a suitable template. Homology modeling of 3D structures for OsCENH3 as well as variant representatives H5, H10, H12 as well as Hδ, Hζ and Hθ, and rs#175193154 was done using Modeller 10.1^56^ Quality of structures was accessed through the SAVES server version 6.0 (https://saves.mbi.ucla.edu/) and Ramachandran plot was made for the selected models to assess the stereo-chemical properties. The structures were aligned with OsCENH3 using PyMOL version 2.5.0. OsCENH3 was also aligned with 3av1.

## Supplementary Information

Supplementary Figures.

Supplementary Tables.

Supplementary Table S1.

Supplementary Table S2.

Supplementary Table S5.

## Data Availability

The sequence data generated as part of this study have been submitted to GenBank, Accession numbers: OK500353-OK500376; OM514977-OM514984 are available to public. Materials can be requested to KN. Link for Data: https://www.ncbi.nlm.nih.gov/nuccore/OK500353.1/. Similarly GenBank accession numbers (OK500353-OK500376; OM514977-OM514984) can be replaced in the above link.
