# Supplementary material for: Uncovering natural allelic and structural variants of OsCENH3 gene by targeted resequencing and in silico mining in genus Oryza
Source: Sci Rep. 2023 Jan 16;13:830. doi: 10.1038/s41598-023-28053-w (PMC9842635; doi:10.1038/s41598-023-28053-w)
Supplement: Supplementary file 1 — Supplementary Figures. [file 41598_2023_28053_MOESM1_ESM.pdf]

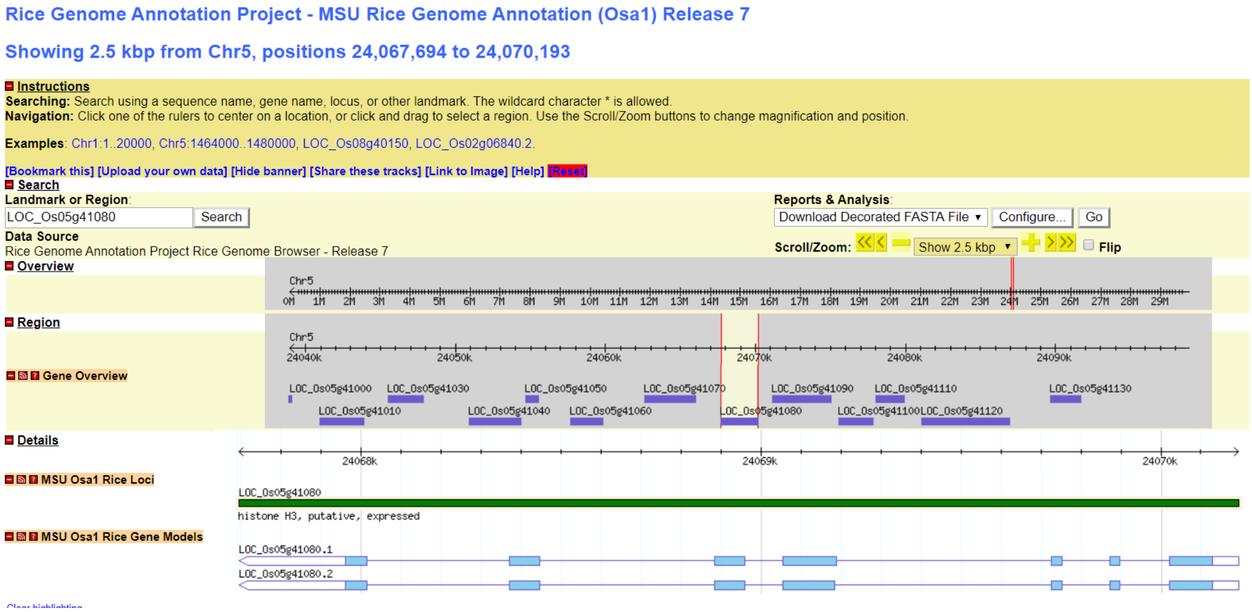

Figure S1: Snapshot of the initial search on MSU-RGAP for *OsCENH3* locus reveals the coordinates of the gene on Chromosome 5.

```
> trimmed-sequence-cenh3+regions

AAAAAAGGAAAAAAAAACAAGAAACATAACTCGATCACTCGACCGTTGGACACATACGCGCGCGTGGCGTTGTG
GCCTTGTGGGATGTGAAAAACGCAACTGACACAGACTGACCCATAAATAACAACAAAACCAAGGGCCTAACCGCA
AAAAGGGATCCCCAACCCAAAACCAAAAGAAACCCTCTCCCGTTTGCCTCCACGCCGCTTCAGTTTGAAAACCCAC
CGCCACGTCGCCGCCGCCGCCGCCGCCGCCGCCGACGCCGAGATGGCTCGCACGAAGCACCCGGCGGTGAGGAA
GTCGAAGGCGGAGCCCAAGAAGAAGCTCCAGTTCGAACGCTCCCCTCGGCCGTCGAAGGCGCAGCGCGCTGGTG
GTGAGCGCGCGCTCTCTCCCCCTCTGCGTTTCTTTTTTTTTTTCCTTTTTCTTTCAATGGCGGTGGATGGTGAAGCTT
ATGCCCCCCCCCCCCCCTTCCCGCCTCTTGCTTGTCCCCTTTGCAGGCGGCACGGGTACCTCGGCGACCACGGTGCG
TGCGGGAGCGGGTCTTTCGTTTGGTGATTTTTTGATTTTGTGGGGGGATATGTTTTTGTTTTGTATCTTGGCTGGAT
GGATGGCTTGCTCACCACCTGTTTGATGGAATGCAGAGGAGCGCGGCTGGAACATCGGCTTCAGGTGCGTTCTCT
TGGGGGGGTTTCTAGGGTTATTCATGGGCTCGTTGGAGCTTTTCCTTTCTGTCTCTTGGATTCCGGGGGACCTGAG
GGGCTCAATGTGTCCCTTTTCTTGCTCTGTTTTACCGTGTGCTGTACTTTCCTCATCGTTGTTTTCTGAATATATTATA
AGAACAGTAGTTGCAGAAAGATCTTCAATTGCTCATCAGTCAAAGCTTTTCTTGTTTTCATTCTGAAATAATAGCAA
ATCCAGTTTGGTCCATGGAGGGGTTATCTGAAACATTATGACCATAAAACATGGTATTAAGCATTGCTAGCCAAGA
AATGTGTGGTTTTTAGACACGATGTTGATAGGTGATTTTTATGCTCATCCATTATTAGTCTTTGCATCGTGGGAACT
GATTTAGTAAACTTTCTTTAGTGTCATGGTTCAAATAGCGTCTGTTCTACCTAGATGATAGGTATCCATATGGAAGT
CTTGGCTTTGGAATTGCTCTCCTTTTGTTCTCCTGTGATTAAATAACTTTAACATGTGTGTGAAGCAGGGACGCCTA
GGCAGCAAACGAAGCAGAGGAAGCCACACCGCTTCCGTCCAGGCACAGTGGCACTGCGGGAGATCAGGAAATTT
CAGAAAACCACCGAACTGCTGATCCCGTTTGCACCATTTTCTCGGCTGGTGGGTACATCCTGAACCTGCCTTCTCTC
TATATCAAATATTTCGTAGTGCAAACTTGTGTGATGGAAGCTTTTTGTGCCGATAAAATTTGCAGGTCAGGGAGAT
CACTGATTTCTATTCAAAGGATGTGTCACGGTGGACCCTTGAAGCTCTCCTTGCATTGCAAGAGGTCAGTGGTCAA
ACCTGTTTATTATAAGTTTACAACTGATGGCTTAGTTAGGGAAGGGTCAGACTGAATTATACTGTTTAAATTCCATT
CTGCTTCAAGACTCAAGTCACGGCTCAAGAGTGTAACTGAAAAATGTACAAATCTTCCATGATCAATAAAATGAAT
ATCTCTGTGTGTTGATTTATGAGTCAGATTGCTAAATTATTATCCTTTTTCAGTAGAACACCTATATACTACAAATAT
GCAACCTCCCTATTTTGTTGTGTCTGTTCAAGATTGCTATCATAGAGTATACCAATTTCAGTTCCTTCTTTCCAGCCA
TGTCTGTTTCTGCATAACCAGGAAAAGGAACAAAGAGCTGACTTAATTCTCACAAAATAAATTATGTTATTTACTTG
CTGTCCTGCAAATTTCCAGTGGTTTTCCCTCTCCTGCAGGCAGCAGAATACCACTTAGTGGACATATTTGAAGTGTC
AAATCTCTGCGCCATCCATGCTAAGCGTGTTACCATCAGTAAGTTGTCATTCTGAATGAACTTTTCTCTTTCTTTTTCT
CCCTTTATATTATTATGCTAAATGGATATCATATATGCCACAGCCTACATGATATCATATACGCATCCACTTCAAAAG
CATTCTATTTTTTTATAGGAATAACATTCTAATTGCAGGATGATTCTTAATACATGTGTTTATATTTAATGTCATATCT
AGTTTTCATACTCTTAAATTTATCATGATTATTGATTAAACATAGGGAGAATTAGTTGGTTTGTGAGTTTTGAGGTG
TGAAATATGCTGCTTGCTATTCCCTGTAAAGCTTATCAGCGTTGTCATTGTGTGGTTTAACAAATAAACGTTTGTTCT
GCAGTGCAAAAGGACATGCAACTTGCCAGGCGTATCGGTGGGCGGAGGCCATGGTGAAAATTTGTTTGCGAGCC
ATGCAGCATGATGGACAAGGAGCAACATGTGTCGTTGATTAACATTTTAGAAAGTAGTGTAGATGTATCTTCACAT
AGGGATCAACTTACCCTTCGTTCCCATTCTAATTCAGTTGATGTTAGTATTTACCTTTTGCTCCATTTGGATTGGTCG
AATTCAGGATTTCATCAAACAGTCGATTGTGAAATGTGAACCAGGAATTGTTGTGTTGATTGCAATAATGGGTTCC
TCTCACCTGCTTCTTCCATCAGATATTTGAACAGCTGTTTTCAAGAACCCTGTGCTCAAGGAACACAGGCAGTTGTT
GAGTGCAGAGGACCTTGAAATATTTCCTCCAACCCCATATATTGCACTCATGATTTGTTATTCAGACAATGTTACAA
AGTTACTGGATCTAGATGGAAACAAGTGGCAAGATGGAAAAAATGGCAGGATCTACACGGAAGCCA
```

Figure S2: Sequence of the gene in which primer binding regions have been highlighted. ATG in green represents start codon and TGA in red represents stop codon. The binding site for primer pair cenh3-p3b is highlighted in pink; for primer pair cenh3-p2a in purple and that of primer paircenh3-p1a in blue.

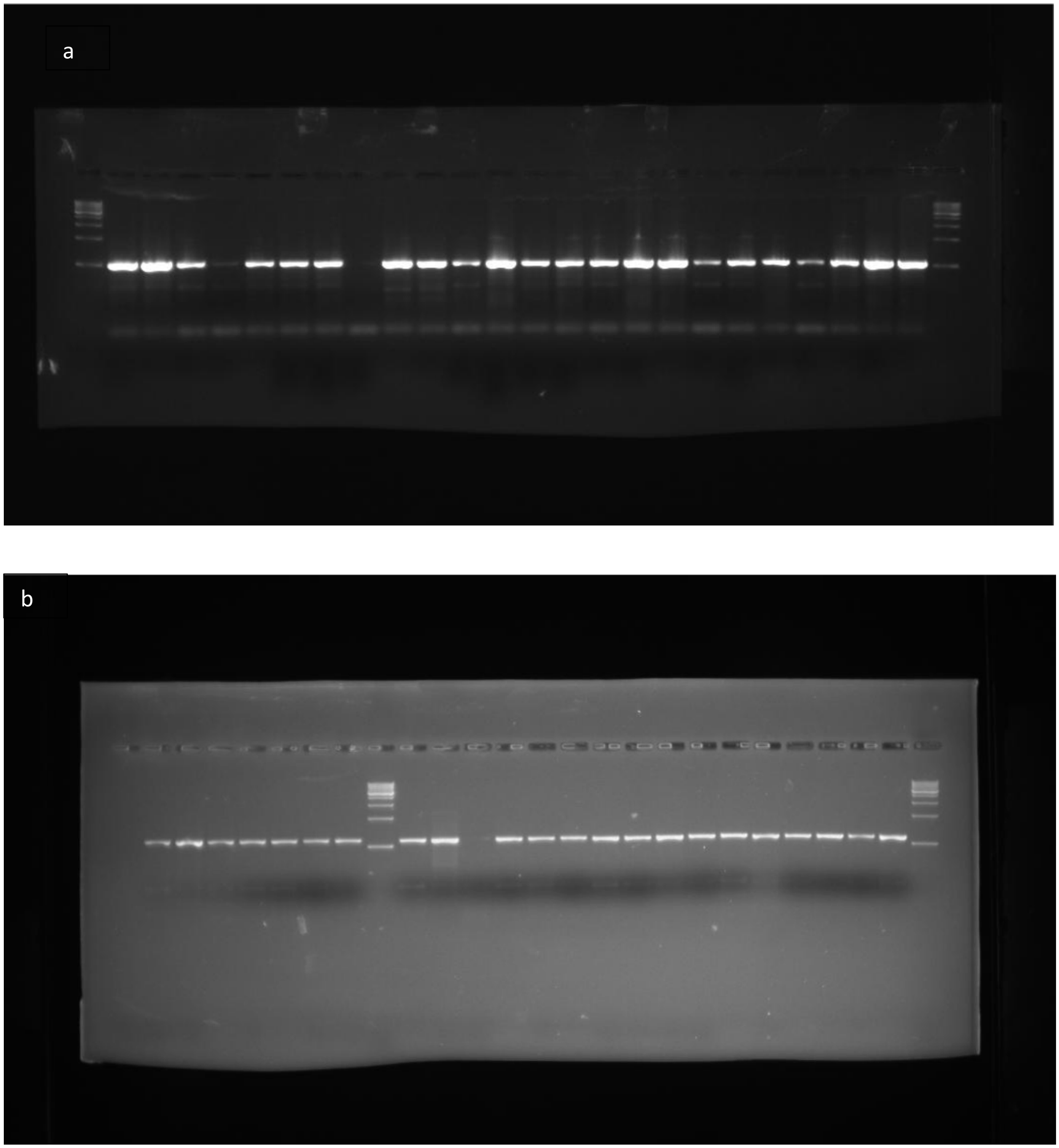
a
b

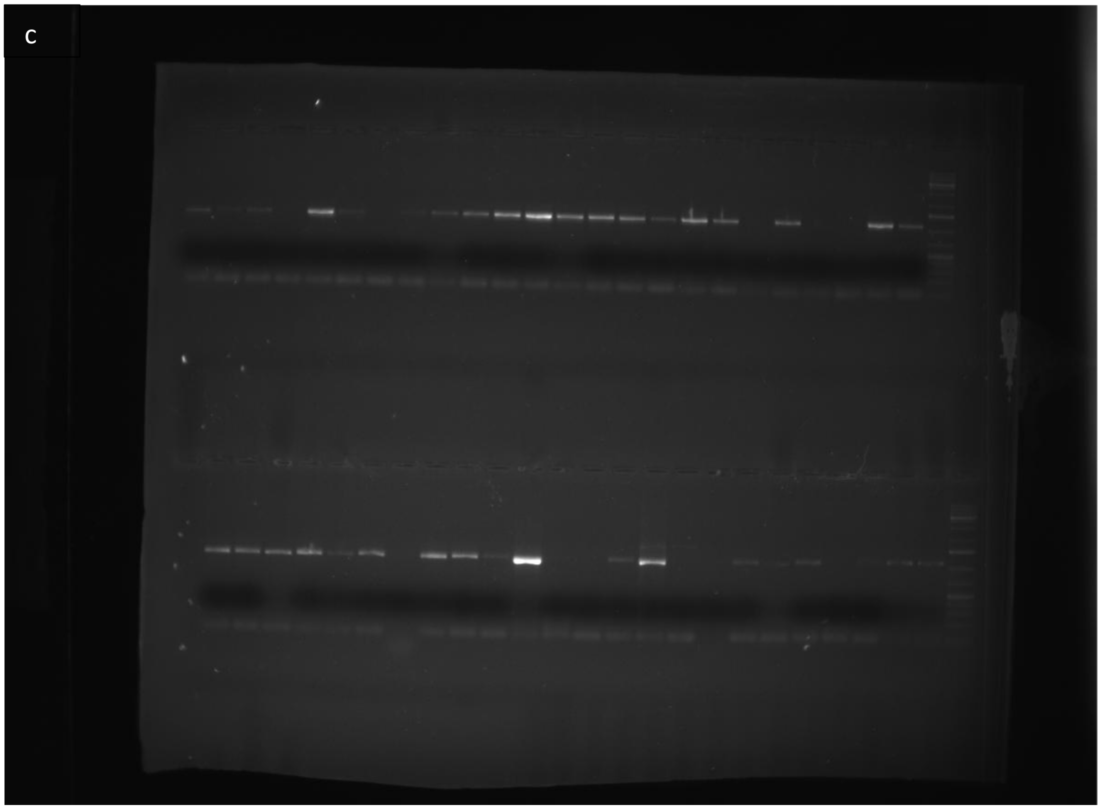

Figure S3: Amplification profile in different wild species using primer pairs (a) cenh3p1a, marker used NEB 1Kb DNA ladder(b) cenh3p2a, marker used NEB 1Kb DNA ladder and (c) cenh3p3b, marker used Thermoscientic Generuler 1kb plus DNA ladder.

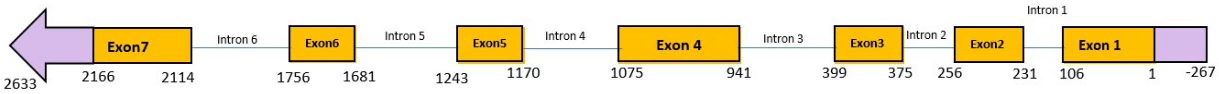

Figure S4: Schematic representation of the gene OsCENH3. It has seven exons and six introns. The numbers ranging from 1 (ATG) to 2166 (TGA) signify the length of the gene and positions of exons on it. Other numbers mark preceding, and trailing regions included for designing overlapping primers.

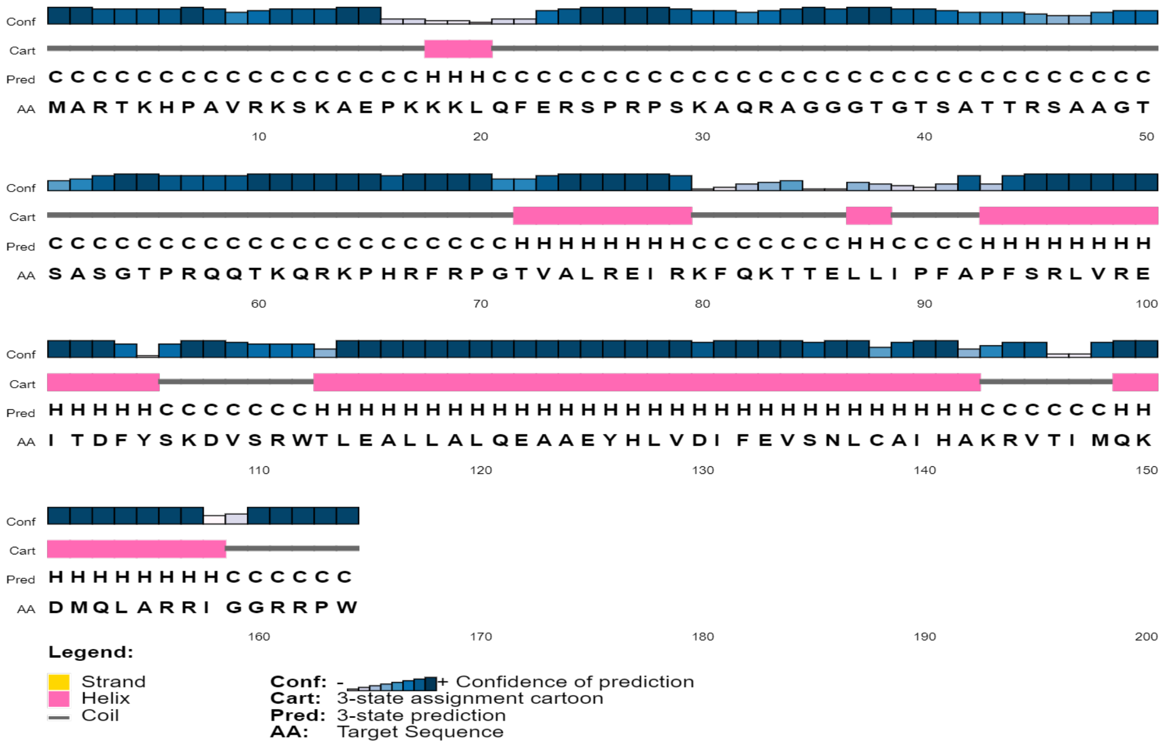

Figure S5A: Secondary structure predicted for OsCENH3 reference

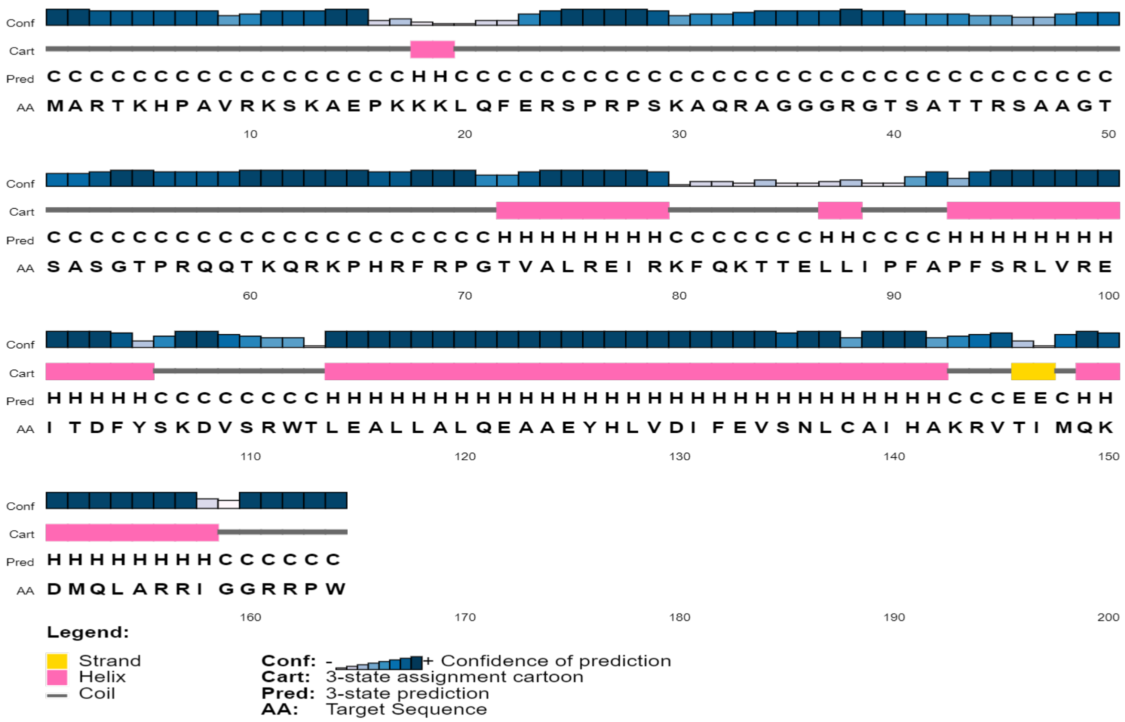

Figure S5B: Secondary structure predicted for H5

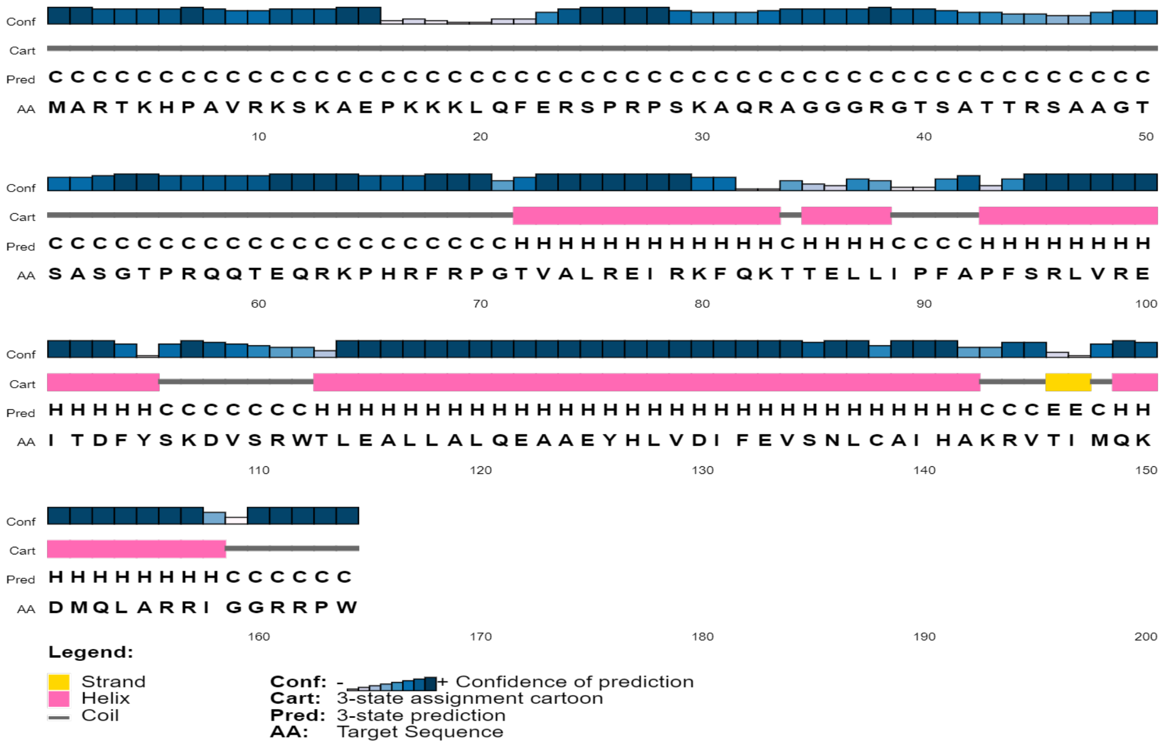

Figure S5C: Secondary structure predicted for H10

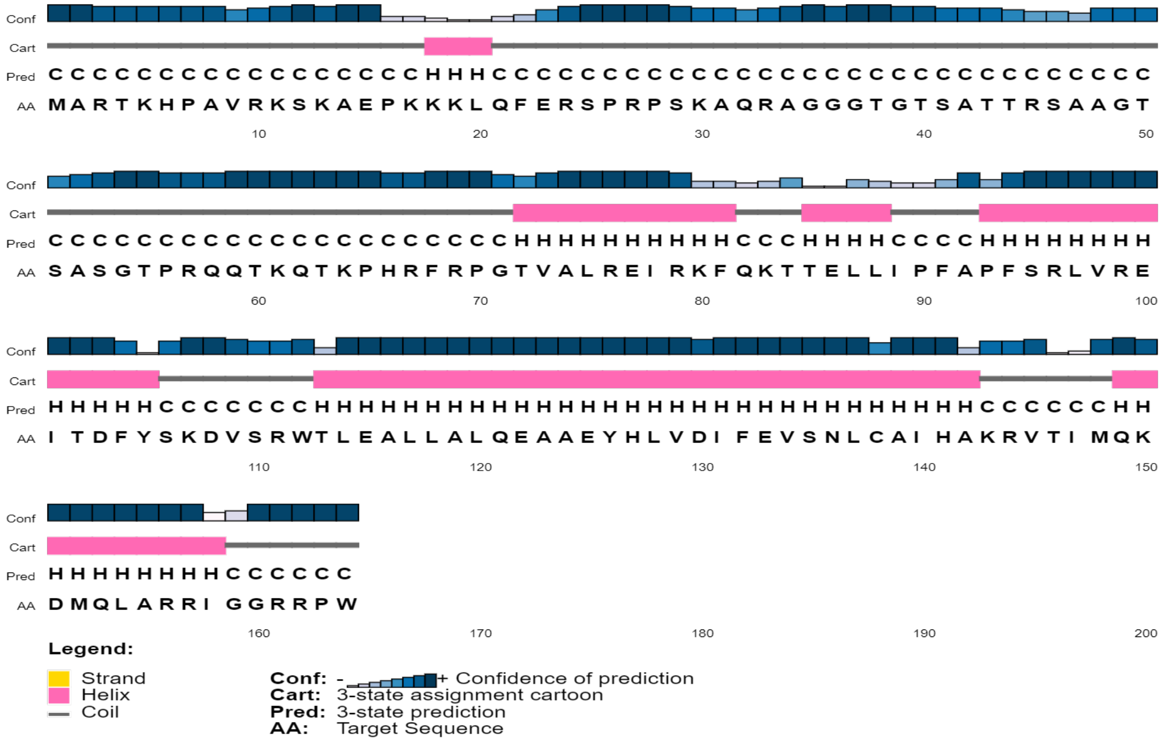

Figure S5D: Secondary structure predicted for H12

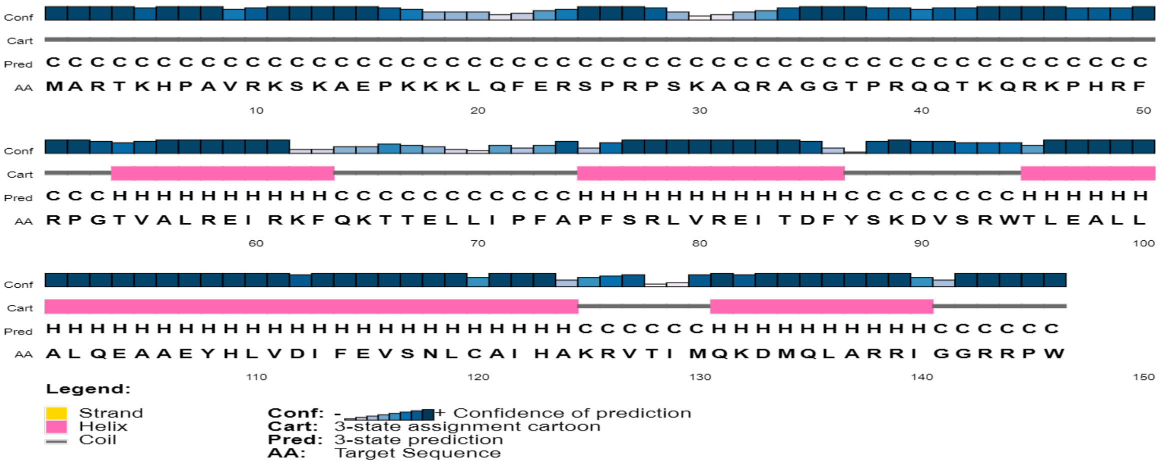

Figure S5E: Secondary structure predicted for Hδ

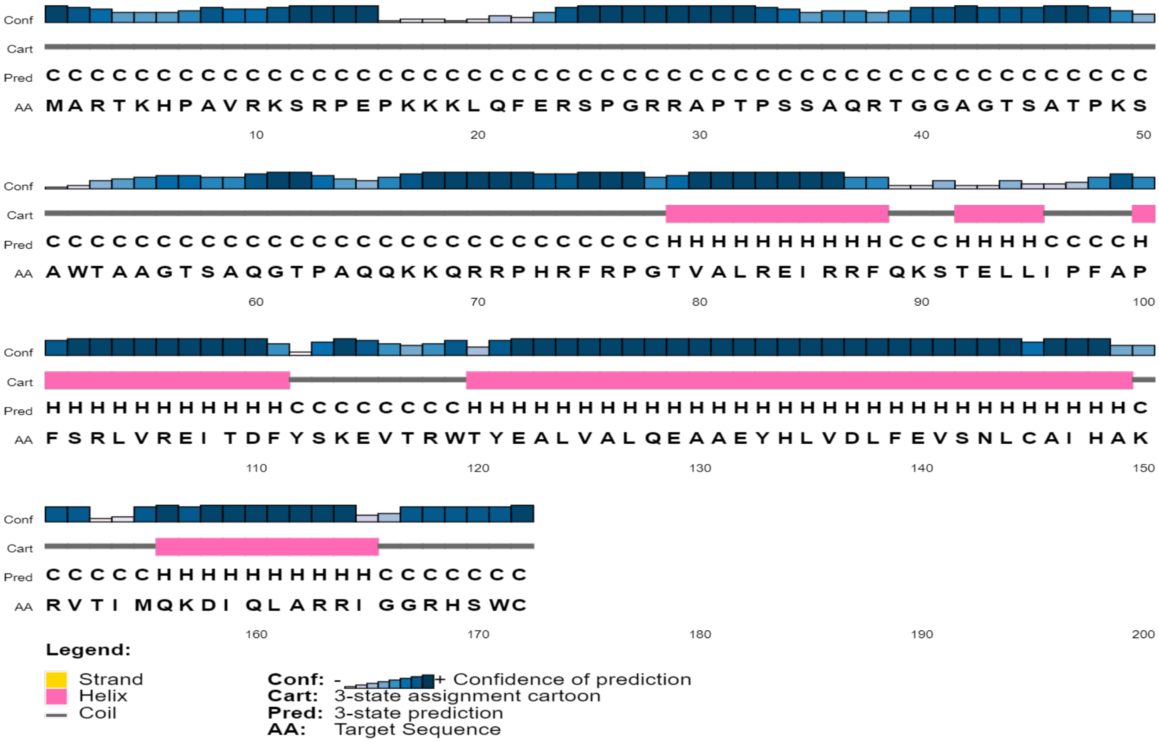

Figure S5F: Secondary structure predicted for Hζ

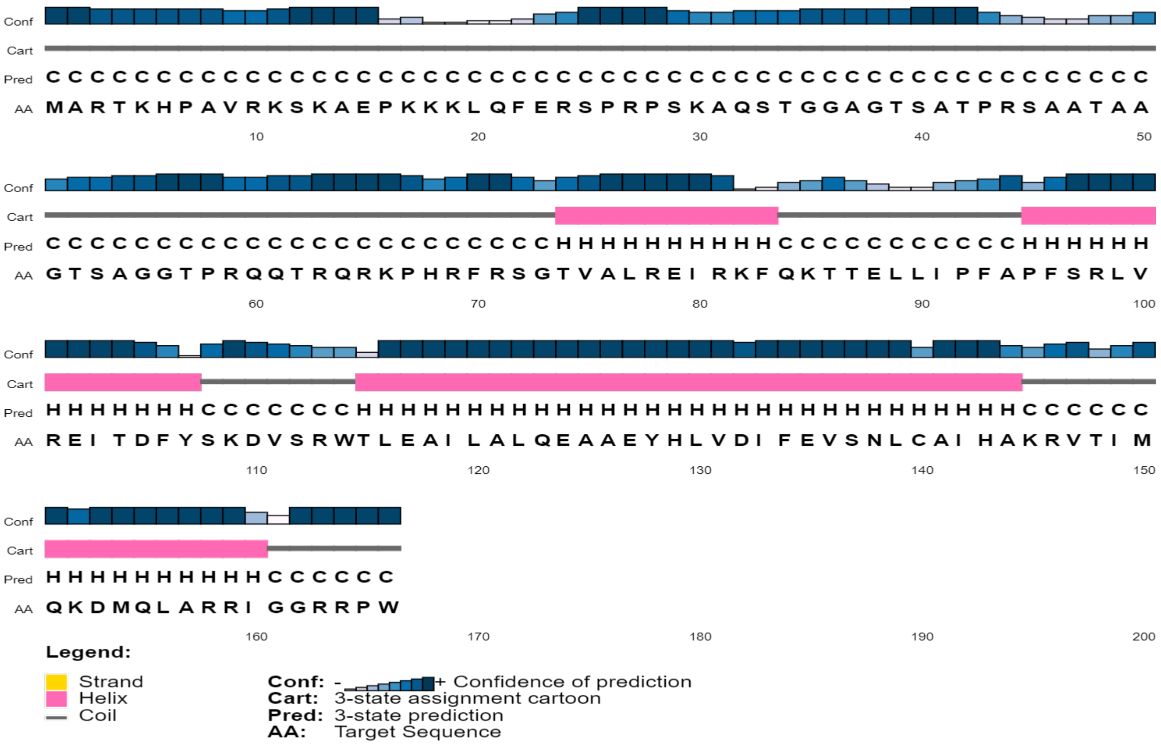

Figure S5G: Secondary structure predicted for Hθ

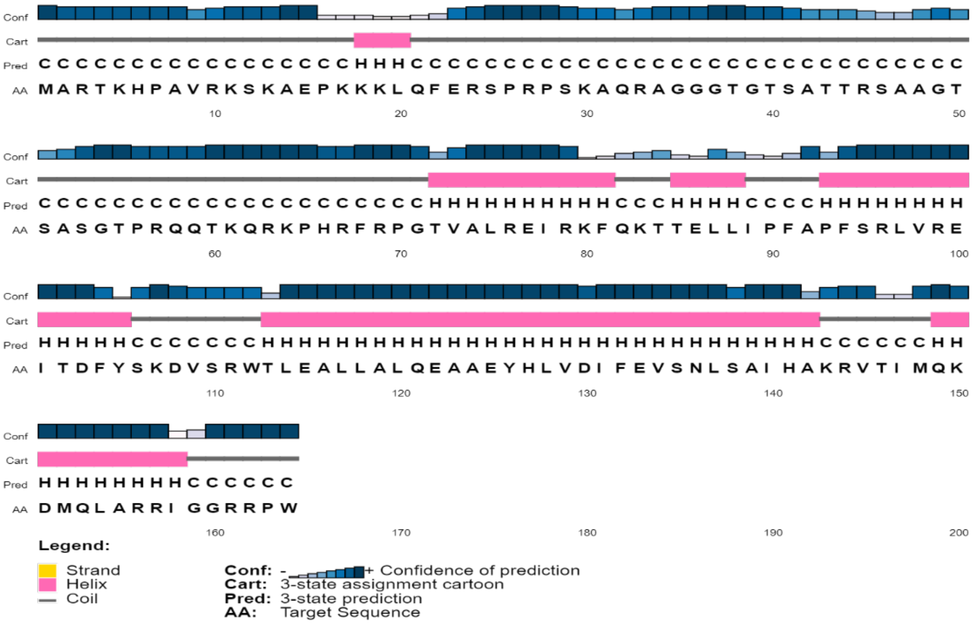

Figure S5H: Secondary structure predicted for rs# 175193154

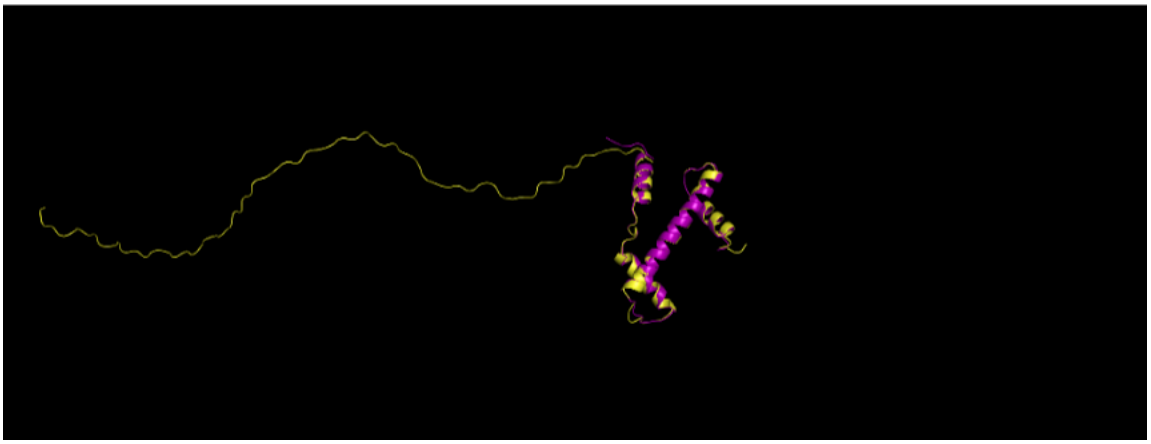

Figure S6: Alignment of 3av1 (Purple) and our reference OsCENH3(yellow). (Superimposed using PyMOL version 2.5.0)

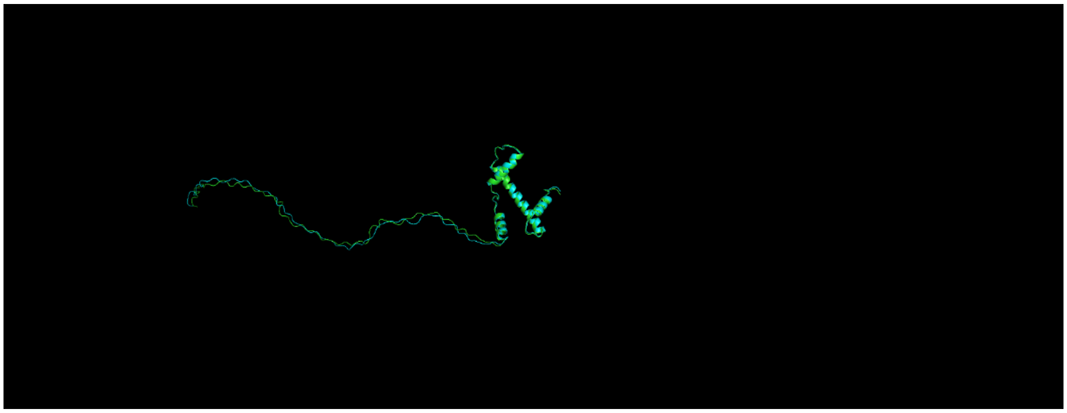

Figure S7: Alignment of rs# 175193154 (Blue) with our reference OsCENH3 (green). (Superimposed using PyMOL version 2.5.0)

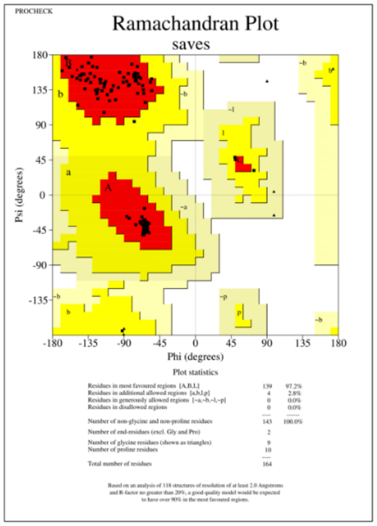

Figure S8: Ramachandran Plot of OsCENH3 (visualized using SAVES server version 6.0)

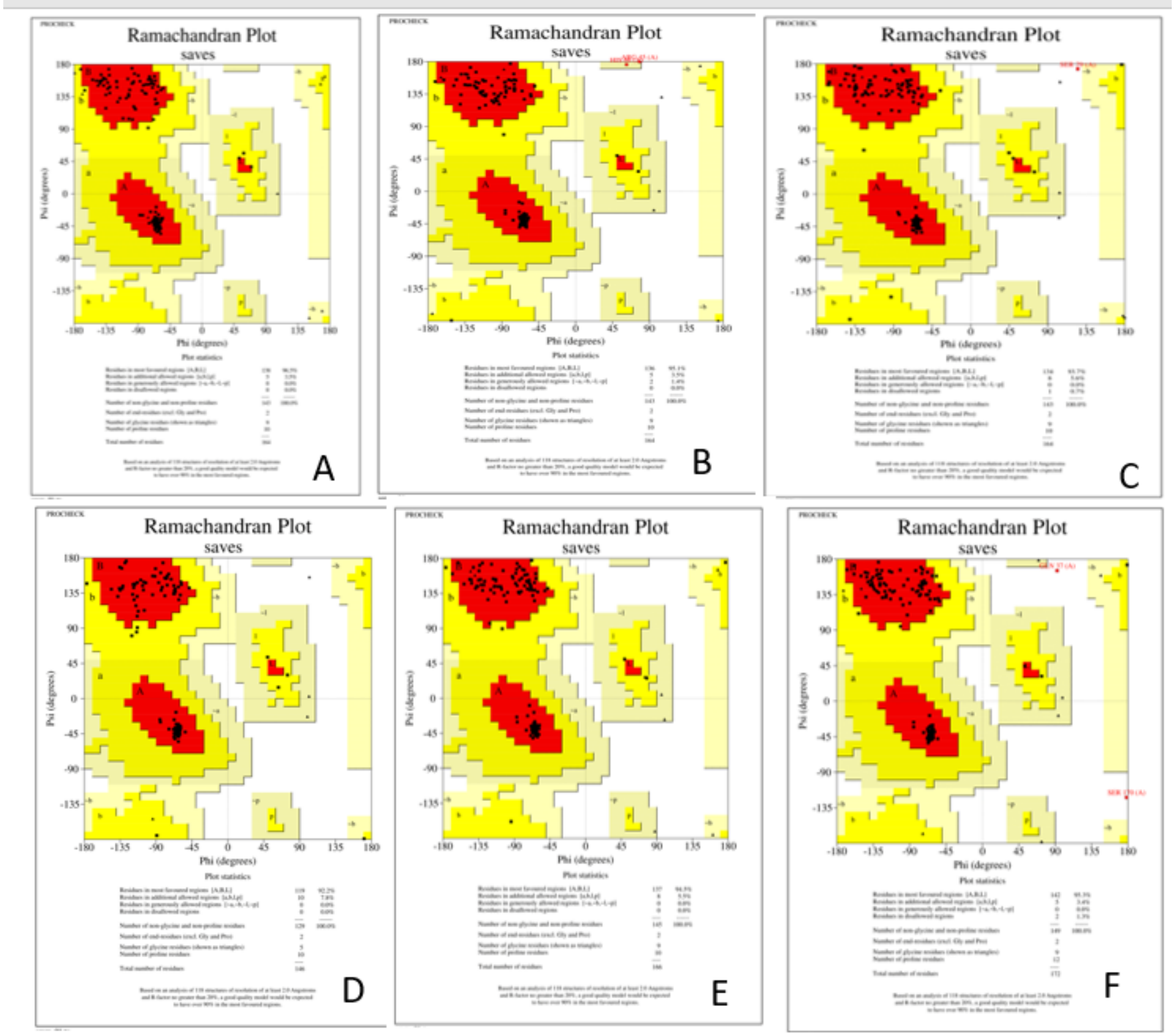

Figure S9A: Ramachandran Plot of H5, B: Ramachandran Plot of H10, C: Ramachandran Plot of H12, D: Ramachandran Plot of Hδ, E; Ramachandran Plot of Hθ, F: Ramachandran Plot of Hζ. (visualized using SAVES server version 6.0)

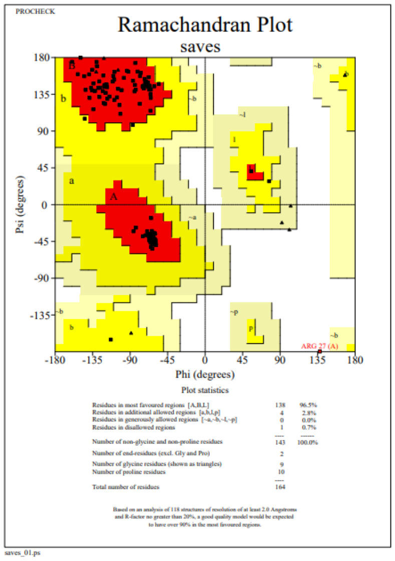

Figure S10: Ramchandran plot for rs# 175193154. (visualized using SAVES server version 6.0)

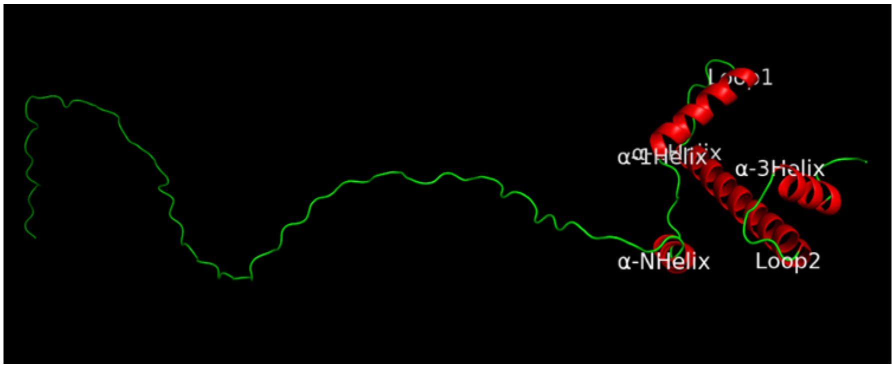

Figure S11: Detailed structure of OsCENH3: OsCENH3 displays 4 helices (αN Helix, α1 Helix, α2 Helix and α3 Helix) and two loops (loop1 and loop2) colored red and green respectively. (Visualised Using PyMOL 2.5.0)

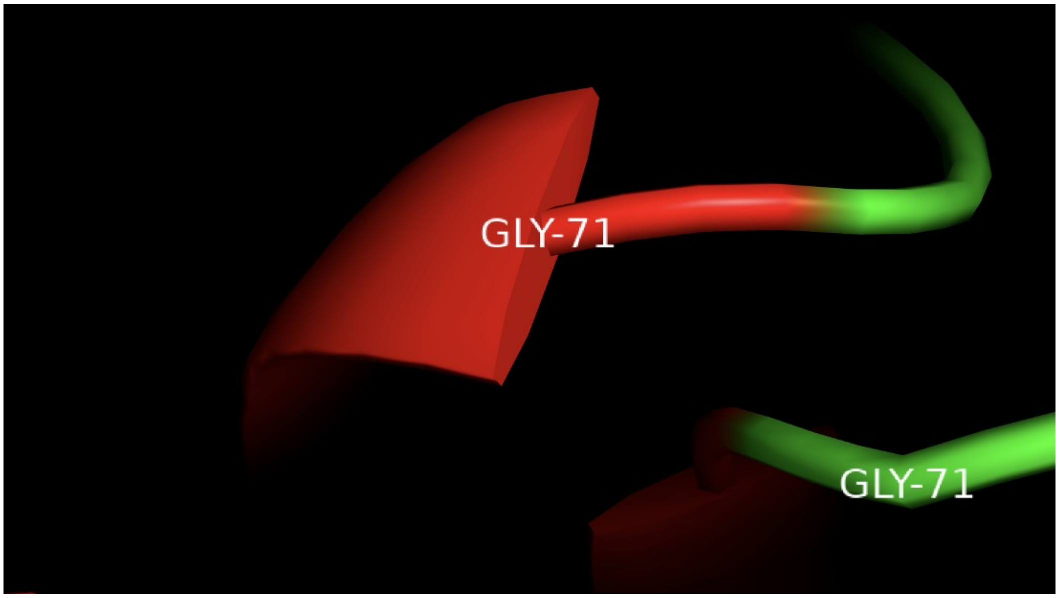

Figure S12: Variations in OsCENH3 and H5: Position 71 in H5 (top) is part of helix while it is not in OsCENH3 (below). Red color represents helical structure. In spite of this, structural change, overall architecture of the reference and H5 do not exhibit major changes as seen in Figure 6

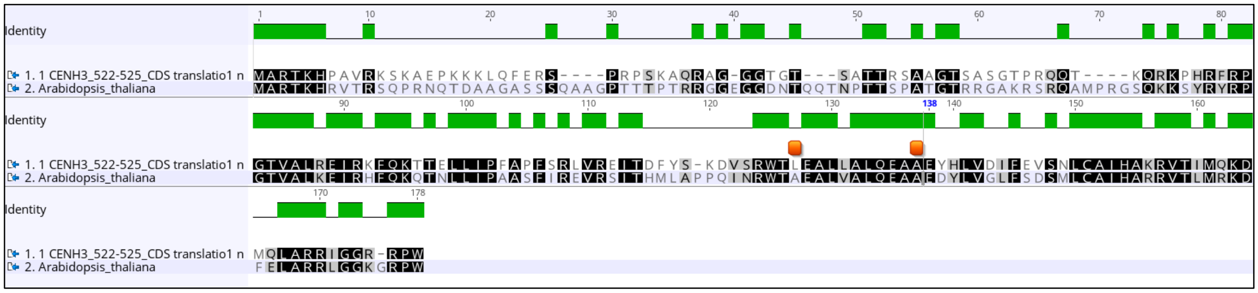

Figure S13: Protein Alignment of OsCENH3 with *Arabidopsis thaliana* CENH3 (Carried out using Geneious Prime version 2021.1.1)
