# Supplementary material for: Uncovering natural allelic and structural variants of OsCENH3 gene by targeted resequencing and in silico mining in genus Oryza
Source: Sci Rep. 2023 Jan 16;13:830. doi: 10.1038/s41598-023-28053-w (PMC9842635; doi:10.1038/s41598-023-28053-w)
Supplement: Supplementary file 2 — Supplementary Tables. [file 41598_2023_28053_MOESM2_ESM.pdf]

Supplementary Table 3: List of primer pairs designed for amplification and sequencing of *OsCENH3*

**Primer_ID**	**Forward Primer (5' – 3')**	**Reverse Primer (5' – 3')**	**Amplicon size* (Range)****
**Cenh3-P1a**	ACTTGTTTCCATCTAGATCCAG	TACTACAAATATGCAACCTCCC	1119 (2594 – 1475)
**Cenh3-P2a**	ACTAAGTGGTATTCTGCTGCCTG	CCGTGTGCTGTACTTTCCT	1176 (1700 – 524)
**Cenh3-P3a**	ACCTATCATCTAGGTAGAACAGAC	AAACCAAGGGCCTAACCG	998 (861 – (-107))
**Cenh3-P3b**	CAGACGCTATTTGAACCATGACAC	AAACAAGAAACATAACTCGATCACT	1094 (842 – (-252))

Supplementary Table S4: List of wild *Oryza* species accessions carrying different haplotypes

Haplotype	Accessions carrying haplotype
H1	Ogl_IR100983, Ob_IR104102
H2	Ogl_IR101800
H3	Ogl_IR102196, On_IR80722, On_IR104688, Or_IR104389, Or_IR104404, Or_IR93076
H4	Ogl_IR103990
H5	Ob_IR100934
H6	On_IR80547
H7	On_IR104650A
H8	On_CR100373, Or_IR80762, Or_IR103404
H9	Or_IR80433
H10	Or_CR100055
H11	Or_IR101411
H12	Ol_IR81965, Ol_IR86485
H13	Ol_IR104301
H14	Om_IR93266,
H15	Oglp_IR100184
H16	Ob_IR89146

Supplementary Table S6: The minimum DOPE scores out of the 5 models built for OsCENH3, -H5, H12, H18 Oryza barthii, Oryza officinalis and Oryza meyeriana var. granulata respectively

**S. No**	**Structure**	**DOPE Score**	**Residues in Most Favored Region**
1	OsCENH3	-10153.37207	97.20%
2	H5	-10164.88867	96.50%
3	H12	-10171.8584	93.70%
4	H8	-10218.48145	95.10%
5	*Oryza barthii*	-9926.43945	92.10%
6	*Oryza officinalis*	-10259.7832	94.50%
7	*Oryza meyeriana var. granulata*	-10560.49219	95.30%

Supplementary Table S7: The alignment of structures with OsCENH3

**S. No**	**Structure**	**Color**	**Match Align Score**	**RMSD (5 Cycles)**
1	H5	Green	832	2.704 (129 to 129 atoms)
2	H12	Blue	832	8.330 (160 to 160 atoms)
3	H8	Magenta	828	3.942 (125 to 125 atoms)
4	*Oryza barthii*	Orange	733.5	27.790 (146 to 146 atoms)
5	*Oryza officinalis*	Cyan	773	9.251 (151 to 151 atoms)
6	Oryza meyeriana var. granulata	Red	702	10.682 (138 to 138 atoms)
