# Supplementary material for: Uncovering natural allelic and structural variants of OsCENH3 gene by targeted resequencing and in silico mining in genus Oryza
Source: Sci Rep. 2023 Jan 16;13:830. doi: 10.1038/s41598-023-28053-w (PMC9842635; doi:10.1038/s41598-023-28053-w)
Supplement: Supplementary file 3 — Supplementary Table S1. [file 41598_2023_28053_MOESM3_ESM.pdf]

Supplementary Table S1: Results of TBLASTn of CENH3 protein query sequences against rice gen

TBLASTN 2.10.1+
Query: lcl|KP202363.1_prot_AJF96734.1_1 [gene=CENH3] [protein=CENH3] [protein_id=AJF967
Database: Nippon
Fields: query acc.ver, subject acc.ver, % identity, alignment length, mismatches, gap opens, q. s
18 hits found

lcl|KP202:	Chr12	52.174	115	51	2	62	176	12808352
lcl|KP202:	Chr12	47.826	115	56	2	62	176	12792796
lcl|KP202:	Chr1	56.566	99	41	1	77	175	37514005
lcl|KP202:	Chr6	56	100	42	1	77	176	3040837
lcl|KP202:	Chr6	56	100	42	1	77	176	3056279
lcl|KP202:	Chr6	56	100	42	1	77	176	3055514
lcl|KP202:	Chr6	57	100	41	1	77	176	1646006
lcl|KP202:	Chr6	56	100	42	1	77	176	3046867
lcl|KP202:	Chr11	56	100	42	1	77	176	2626112
lcl|KP202:	Chr5	56	100	42	1	77	176	21499987
lcl|KP202:	Chr5	60	40	16	0	73	112	24069169
lcl|KP202:	Chr4	56	100	42	1	77	176	20739869
lcl|KP202:	Chr4	35.028	177	49	4	60	176	22463218
lcl|KP202:	Chr2	38.462	117	42	1	90	176	15225499
lcl|KP202:	Chr2	60	35	14	0	142	176	15204221
lcl|KP202:	Chr3	62.857	35	13	0	142	176	15647702
lcl|KP202:	Chr3	55.556	27	12	0	91	117	15647458
lcl|KP202:	Chr3	68.75	16	5	0	77	92	15647325

TBLASTN 2.10.1+
Query: lcl|KP177475.1_prot_AJD07128.1_1 [gene=CenH3] [protein=CenH3] [protein_id=AJD071:
Database: Nippon
Fields: query acc.ver, subject acc.ver, % identity, alignment length, mismatches, gap opens, q. s
22 hits found

lcl|KP1774	Chr1	60	95	37	1	54	148	37514014
lcl|KP1774	Chr1	32.53	83	48	2	75	149	25931765
lcl|KP1774	Chr6	60	95	37	1	54	148	3056288
lcl|KP1774	Chr6	60	95	37	1	54	148	3040828
lcl|KP1774	Chr6	60	95	37	1	54	148	3055505
lcl|KP1774	Chr6	60	95	37	1	54	148	3046858
lcl|KP1774	Chr6	58.947	95	38	1	54	148	1645997
lcl|KP1774	Chr5	60	95	37	1	54	148	21499978
lcl|KP1774	Chr5	66.667	33	11	0	54	86	24069148
lcl|KP1774	Chr5	46.429	28	15	0	85	112	24068961
lcl|KP1774	Chr5	57.143	28	12	0	110	137	24068449
lcl|KP1774	Chr11	60	95	37	1	54	148	2626103
lcl|KP1774	Chr4	60	95	37	1	54	148	20739878
lcl|KP1774	Chr4	41.739	115	36	1	65	148	22463383
lcl|KP1774	Chr12	55.102	98	37	2	54	148	12808304
lcl|KP1774	Chr12	50.526	95	46	1	54	148	12792748
lcl|KP1774	Chr3	66	50	17	0	99	148	15647654
lcl|KP1774	Chr3	48.571	35	18	0	65	99	15647458
lcl|KP1774	Chr2	39.655	116	39	1	64	148	15225499
lcl|KP1774	Chr2	62	50	19	0	99	148	15204269

lcl|KP1774	Chr2	51.724	29	14	0	65	93	15204465
lcl|KP1774	Chr2	30.263	76	50	2	66	141	2140530

TBLASTN 2.10.1+
Query: lcl|KP177470.1_prot_AJD07123.1_1 [gene=CenH3] [protein=CenH3] [protein_id=AJD0712
Database: Nippon
Fields: query acc.ver, subject acc.ver, % identity, alignment length, mismatches, gap opens, q. s
25 hits found

lcl|KP1774	Chr11	56.637	113	48	1	36	148	2626157
lcl|KP1774	Chr6	56.637	113	48	1	36	148	3040882
lcl|KP1774	Chr6	56.637	113	48	1	36	148	3055559
lcl|KP1774	Chr6	56.637	113	48	1	36	148	3046912
lcl|KP1774	Chr6	56.637	113	48	1	36	148	3056234
lcl|KP1774	Chr6	58.491	106	43	1	43	148	1646030
lcl|KP1774	Chr5	56.637	113	48	1	36	148	21500032
lcl|KP1774	Chr5	63.158	38	14	0	49	86	24069163
lcl|KP1774	Chr5	42.857	28	16	0	85	112	24068961
lcl|KP1774	Chr5	57.143	28	12	0	110	137	24068449
lcl|KP1774	Chr1	57.522	113	44	2	36	148	37513969
lcl|KP1774	Chr4	56.637	113	48	1	36	148	20739824
lcl|KP1774	Chr4	36.527	167	45	2	43	148	22463227
lcl|KP1774	Chr12	52.632	114	47	2	38	148	12808352
lcl|KP1774	Chr12	47.748	111	57	1	38	148	12792796
lcl|KP1774	Chr3	68	50	16	0	99	148	15647654
lcl|KP1774	Chr3	45.714	35	19	0	65	99	15647458
lcl|KP1774	Chr3	50	24	12	0	43	66	15647301
lcl|KP1774	Chr3	46.667	30	16	0	74	103	35714101
lcl|KP1774	Chr2	64	50	18	0	99	148	15204269
lcl|KP1774	Chr2	51.724	29	14	0	65	93	15204465
lcl|KP1774	Chr2	40.517	116	38	2	64	148	15225499
lcl|KP1774	Chr2	63.158	19	7	0	50	68	20134897
lcl|KP1774	Chr2	41.304	46	17	2	105	148	20135034
lcl|KP1774	Chr7	45.833	24	13	0	109	132	26026461

TBLASTN 2.10.1+
Query: lcl|KP177464.1_prot_AJD07117.1_1 [gene=CenH3] [protein=CenH3] [protein_id=AJD0711
Database: Nippon
Fields: query acc.ver, subject acc.ver, % identity, alignment length, mismatches, gap opens, q. s
24 hits found

lcl|KP1774	Chr1	60.606	99	38	1	50	148	37514002
lcl|KP1774	Chr6	60	100	39	1	49	148	3040843
lcl|KP1774	Chr6	60	100	39	1	49	148	3056273
lcl|KP1774	Chr6	60	100	39	1	49	148	3055520
lcl|KP1774	Chr6	60	100	39	1	49	148	3046873
lcl|KP1774	Chr6	59	100	40	1	49	148	1646012
lcl|KP1774	Chr11	60	100	39	1	49	148	2626118
lcl|KP1774	Chr5	60	100	39	1	49	148	21499993
lcl|KP1774	Chr5	63.889	36	13	0	51	86	24069157
lcl|KP1774	Chr5	46.429	28	15	0	85	112	24068961
lcl|KP1774	Chr5	57.143	28	12	0	110	137	24068449

lcl|KP1774	Chr4	60	100	39	1	49	148	20739863
lcl|KP1774	Chr4	36.25	160	41	2	50	148	22463248
lcl|KP1774	Chr12	56.311	103	38	2	49	148	12808319
lcl|KP1774	Chr12	52	100	47	1	49	148	12792763
lcl|KP1774	Chr3	68	50	16	0	99	148	15647654
lcl|KP1774	Chr3	48.571	35	18	0	65	99	15647458
lcl|KP1774	Chr3	50	18	9	0	49	66	15647319
lcl|KP1774	Chr2	40.87	115	37	1	65	148	15225496
lcl|KP1774	Chr2	64	50	18	0	99	148	15204269
lcl|KP1774	Chr2	51.724	29	14	0	65	93	15204465
lcl|KP1774	Chr2	66.667	18	6	0	51	68	20134900
lcl|KP1774	Chr2	41.304	46	17	2	105	148	20135034
lcl|KP1774	Chr7	45.833	24	13	0	109	132	26026461

TBLASTN 2.10.1+
Query: lcl|KP177463.1_prot_AJD07116.1_1 [gene=CenH3] [protein=CenH3] [protein_id=AJD0711
Database: Nippon
Fields: query acc.ver, subject acc.ver, % identity, alignment length, mismatches, gap opens, q. s
26 hits found

lcl|KP1774	Chr11	58.407	113	46	1	36	148	2626157
lcl|KP1774	Chr6	58.407	113	46	1	36	148	3040882
lcl|KP1774	Chr6	58.407	113	46	1	36	148	3055559
lcl|KP1774	Chr6	58.407	113	46	1	36	148	3046912
lcl|KP1774	Chr6	58.407	113	46	1	36	148	3056234
lcl|KP1774	Chr6	59.434	106	42	1	43	148	1646030
lcl|KP1774	Chr5	58.407	113	46	1	36	148	21500032
lcl|KP1774	Chr5	68.421	38	12	0	49	86	24069163
lcl|KP1774	Chr5	42.857	28	16	0	85	112	24068961
lcl|KP1774	Chr5	57.143	28	12	0	110	137	24068449
lcl|KP1774	Chr4	58.407	113	46	1	36	148	20739824
lcl|KP1774	Chr4	37.126	167	44	2	43	148	22463227
lcl|KP1774	Chr1	58.407	113	46	1	36	148	37513960
lcl|KP1774	Chr12	53.509	114	46	2	38	148	12808352
lcl|KP1774	Chr12	49.55	111	55	1	38	148	12792796
lcl|KP1774	Chr3	68	50	16	0	99	148	15647654
lcl|KP1774	Chr3	48.571	35	18	0	65	99	15647458
lcl|KP1774	Chr3	50	24	12	0	43	66	15647301
lcl|KP1774	Chr2	41.379	116	37	1	64	148	15225499
lcl|KP1774	Chr2	64	50	18	0	99	148	15204269
lcl|KP1774	Chr2	55.172	29	13	0	65	93	15204465
lcl|KP1774	Chr2	68.421	19	6	0	50	68	20134897
lcl|KP1774	Chr2	41.304	46	17	2	105	148	20135034
lcl|KP1774	Chr2	27.632	76	52	2	66	141	2140530
lcl|KP1774	Chr2	50	24	12	0	43	66	15225793
lcl|KP1774	Chr7	45.833	24	13	0	109	132	26026461

TBLASTN 2.10.1+
Query: lcl|KP177462.1_prot_AJD07115.1_1 [gene=CenH3] [protein=CenH3] [protein_id=AJD0711
Database: Nippon
Fields: query acc.ver, subject acc.ver, % identity, alignment length, mismatches, gap opens, q. s

26 hits found

lcl|KP1774	Chr11	54.867	113	50	1	36	148	2626157
lcl|KP1774	Chr6	54.867	113	50	1	36	148	3055559
lcl|KP1774	Chr6	54.867	113	50	1	36	148	3040882
lcl|KP1774	Chr6	54.867	113	50	1	36	148	3046912
lcl|KP1774	Chr6	54.867	113	50	1	36	148	3056234
lcl|KP1774	Chr6	56.604	106	45	1	43	148	1646030
lcl|KP1774	Chr5	54.867	113	50	1	36	148	21500032
lcl|KP1774	Chr5	63.158	38	14	0	49	86	24069163
lcl|KP1774	Chr5	42.857	28	16	0	85	112	24068961
lcl|KP1774	Chr5	53.571	28	13	0	110	137	24068449
lcl|KP1774	Chr1	55.752	113	46	2	36	148	37513969
lcl|KP1774	Chr4	54.867	113	50	1	36	148	20739824
lcl|KP1774	Chr4	35.329	167	47	2	43	148	22463227
lcl|KP1774	Chr12	51.351	111	53	1	38	148	12808352
lcl|KP1774	Chr12	46.847	111	58	1	38	148	12792796
lcl|KP1774	Chr3	66	50	17	0	99	148	15647654
lcl|KP1774	Chr3	50	24	12	0	43	66	15647301
lcl|KP1774	Chr3	53.846	26	12	0	65	90	15647458
lcl|KP1774	Chr3	50	30	15	0	74	103	35714101
lcl|KP1774	Chr3	43.333	30	17	0	74	103	35730434
lcl|KP1774	Chr2	39.655	116	39	2	64	148	15225499
lcl|KP1774	Chr2	62	50	19	0	99	148	15204269
lcl|KP1774	Chr2	51.852	27	13	0	64	90	15204468
lcl|KP1774	Chr2	63.158	19	7	0	50	68	20134897
lcl|KP1774	Chr2	41.304	46	17	2	105	148	20135034
lcl|KP1774	Chr10	32.692	52	35	0	54	105	9042169

TBLASTN 2.10.1+
Query: lcl|KP177459.1_prot_AJD07112.1_1 [gene=CenH3] [protein=CenH3] [protein_id=AJD0711
Database: Nippon
Fields: query acc.ver, subject acc.ver, % identity, alignment length, mismatches, gap opens, q. s
28 hits found

lcl|KP1774	Chr11	55.752	113	49	1	36	148	2626157
lcl|KP1774	Chr6	55.752	113	49	1	36	148	3040882
lcl|KP1774	Chr6	55.752	113	49	1	36	148	3056234
lcl|KP1774	Chr6	55.752	113	49	1	36	148	3055559
lcl|KP1774	Chr6	55.752	113	49	1	36	148	3046912
lcl|KP1774	Chr6	57.547	106	44	1	43	148	1646030
lcl|KP1774	Chr5	55.752	113	49	1	36	148	21500032
lcl|KP1774	Chr5	60.526	38	15	0	49	86	24069163
lcl|KP1774	Chr5	42.857	28	16	0	85	112	24068961
lcl|KP1774	Chr5	57.143	28	12	0	110	137	24068449
lcl|KP1774	Chr5	37.288	59	34	2	34	92	27687688
lcl|KP1774	Chr1	55.752	113	49	1	36	148	37513960
lcl|KP1774	Chr4	55.752	113	49	1	36	148	20739824
lcl|KP1774	Chr4	35.928	167	46	2	43	148	22463227
lcl|KP1774	Chr12	53.211	109	44	2	43	148	12808337
lcl|KP1774	Chr12	50	106	52	1	43	148	12792781

lcl|KP1774	Chr3	68	50	16	0	99	148	15647654
lcl|KP1774	Chr3	45.714	35	19	0	65	99	15647458
lcl|KP1774	Chr3	45.833	24	13	0	43	66	15647301
lcl|KP1774	Chr3	46.667	30	16	0	74	103	35714101
lcl|KP1774	Chr2	40.517	116	38	2	64	148	15225499
lcl|KP1774	Chr2	64	50	18	0	99	148	15204269
lcl|KP1774	Chr2	51.724	29	14	0	65	93	15204465
lcl|KP1774	Chr2	57.895	19	8	0	50	68	20134897
lcl|KP1774	Chr2	41.304	46	17	2	105	148	20135034
lcl|KP1774	Chr2	33.898	59	38	1	55	112	7812463
lcl|KP1774	Chr8	42.424	33	19	0	34	66	23759027
lcl|KP1774	Chr7	45.833	24	13	0	109	132	26026461

TBLASTN 2.10.1+
Query: lcl|KP177458.1_prot_AJD07111.1_1 [gene=CenH3] [protein=CenH3] [protein_id=AJD071
Database: Nippon
Fields: query acc.ver, subject acc.ver, % identity, alignment length, mismatches, gap opens, q. s
25 hits found

lcl|KP1774	Chr11	54.867	113	50	1	36	148	2626157
lcl|KP1774	Chr6	54.867	113	50	1	36	148	3040882
lcl|KP1774	Chr6	54.867	113	50	1	36	148	3055559
lcl|KP1774	Chr6	54.867	113	50	1	36	148	3046912
lcl|KP1774	Chr6	54.867	113	50	1	36	148	3056234
lcl|KP1774	Chr6	56.604	106	45	1	43	148	1646030
lcl|KP1774	Chr5	54.867	113	50	1	36	148	21500032
lcl|KP1774	Chr5	63.158	38	14	0	49	86	24069163
lcl|KP1774	Chr5	46.429	28	15	0	85	112	24068961
lcl|KP1774	Chr5	50	28	14	0	110	137	24068449
lcl|KP1774	Chr1	55.752	113	46	2	36	148	37513969
lcl|KP1774	Chr4	54.867	113	50	1	36	148	20739824
lcl|KP1774	Chr4	35.329	167	47	2	43	148	22463227
lcl|KP1774	Chr12	50.877	114	49	2	38	148	12808352
lcl|KP1774	Chr12	45.946	111	59	1	38	148	12792796
lcl|KP1774	Chr3	64	50	18	0	99	148	15647654
lcl|KP1774	Chr3	48.571	35	18	0	65	99	15647458
lcl|KP1774	Chr3	50	24	12	0	43	66	15647301
lcl|KP1774	Chr2	38.793	116	40	1	64	148	15225499
lcl|KP1774	Chr2	60	50	20	0	99	148	15204269
lcl|KP1774	Chr2	51.724	29	14	0	65	93	15204465
lcl|KP1774	Chr2	63.158	19	7	0	50	68	20134897
lcl|KP1774	Chr2	39.13	46	18	2	105	148	20135034
lcl|KP1774	Chr2	30.137	73	49	2	55	125	7812463
lcl|KP1774	Chr2	27.632	76	52	2	66	141	2140530

TBLASTN 2.10.1+
Query: lcl|GQ849334.1_prot_ACX30895.1_1 [gene=CenH3] [protein=CENH3] [protein_id=ACX30
Database: Nippon
Fields: query acc.ver, subject acc.ver, % identity, alignment length, mismatches, gap opens, q. s
25 hits found

lcl|GQ849	Chr6	68	100	31	1	65	164	3040837

lcl|GQ849	Chr6	68	100	31	1	65	164	3055514
lcl|GQ849	Chr6	68	100	31	1	65	164	3056279
lcl|GQ849	Chr6	68	100	31	1	65	164	3046867
lcl|GQ849	Chr6	68	100	31	1	65	164	1646006
lcl|GQ849	Chr11	68	100	31	1	65	164	2626112
lcl|GQ849	Chr5	68	100	31	1	65	164	21499987
lcl|GQ849	Chr5	95.122	41	2	0	60	100	24069172
lcl|GQ849	Chr5	100	16	0	0	100	115	24068958
lcl|GQ849	Chr5	100	32	0	0	1	32	24070126
lcl|GQ849	Chr5	100	22	0	0	128	149	24068437
lcl|GQ849	Chr5	62.069	29	4	1	138	166	24068029
lcl|GQ849	Chr4	68	100	31	1	65	164	20739869
lcl|GQ849	Chr4	40.994	161	34	2	65	164	22463251
lcl|GQ849	Chr1	68.367	98	30	1	65	162	37514005
lcl|GQ849	Chr12	61.165	103	33	2	65	164	12808313
lcl|GQ849	Chr12	61	100	38	1	65	164	12792757
lcl|GQ849	Chr2	47.458	118	31	1	78	164	15225499
lcl|GQ849	Chr2	72.222	36	10	0	129	164	15204221
lcl|GQ849	Chr2	66.667	30	10	0	78	107	15204468
lcl|GQ849	Chr2	77.778	18	4	0	65	82	20134900
lcl|GQ849	Chr3	72.973	37	10	0	129	165	15647702
lcl|GQ849	Chr3	57.143	35	15	0	79	113	15647458
lcl|GQ849	Chr3	68.75	16	5	0	65	80	15647325
lcl|GQ849	Chr7	60	20	8	0	94	113	11774928

TBLASTN 2.10.1+
Query: lcl|GQ849333.1_prot_ACX30894.1_1 [gene=CenH3] [protein=CENH3] [protein_id=ACX30
Database: Nippon
Fields: query acc.ver, subject acc.ver, % identity, alignment length, mismatches, gap opens, q. s
26 hits found

lcl|GQ849	Chr6	68	100	31	1	65	164	3040837
lcl|GQ849	Chr6	68	100	31	1	65	164	3055514
lcl|GQ849	Chr6	68	100	31	1	65	164	3056279
lcl|GQ849	Chr6	68	100	31	1	65	164	3046867
lcl|GQ849	Chr6	68	100	31	1	65	164	1646006
lcl|GQ849	Chr11	68	100	31	1	65	164	2626112
lcl|GQ849	Chr5	68	100	31	1	65	164	21499987
lcl|GQ849	Chr5	93.023	43	3	0	58	100	24069178
lcl|GQ849	Chr5	100	16	0	0	100	115	24068958
lcl|GQ849	Chr5	96.875	32	1	0	1	32	24070126
lcl|GQ849	Chr5	100	22	0	0	128	149	24068437
lcl|GQ849	Chr5	62.069	29	4	1	138	166	24068029
lcl|GQ849	Chr4	68	100	31	1	65	164	20739869
lcl|GQ849	Chr4	40.994	161	34	2	65	164	22463251
lcl|GQ849	Chr1	68.367	98	30	1	65	162	37514005
lcl|GQ849	Chr12	61.165	103	33	2	65	164	12808313
lcl|GQ849	Chr12	61	100	38	1	65	164	12792757
lcl|GQ849	Chr2	46.61	118	32	1	78	164	15225499
lcl|GQ849	Chr2	72.222	36	10	0	129	164	15204221

lcl|GQ849	Chr2	63.333	30	11	0	78	107	15204468
lcl|GQ849	Chr2	83.333	18	3	0	65	82	20134900
lcl|GQ849	Chr2	75	16	4	0	65	80	15225769
lcl|GQ849	Chr3	72.973	37	10	0	129	165	15647702
lcl|GQ849	Chr3	54.286	35	16	0	79	113	15647458
lcl|GQ849	Chr3	66.667	18	6	0	65	82	15647325
lcl|GQ849	Chr7	60	20	8	0	94	113	11774928

TBLASTN 2.10.1+
Query: lcl|GQ849332.1_prot_ACX30893.1_1 [gene=CenH3] [protein=CENH3] [protein_id=ACX30
Database: Nippon
Fields: query acc.ver, subject acc.ver, % identity, alignment length, mismatches, gap opens, q. s
26 hits found

lcl|GQ849	Chr6	71	100	28	1	62	161	3040837
lcl|GQ849	Chr6	71	100	28	1	62	161	3055514
lcl|GQ849	Chr6	71	100	28	1	62	161	3056279
lcl|GQ849	Chr6	71	100	28	1	62	161	3046867
lcl|GQ849	Chr6	71	100	28	1	62	161	1646006
lcl|GQ849	Chr11	71	100	28	1	62	161	2626112
lcl|GQ849	Chr5	71	100	28	1	62	161	21499987
lcl|GQ849	Chr5	95.349	43	2	0	55	97	24069178
lcl|GQ849	Chr5	100	16	0	0	97	112	24068958
lcl|GQ849	Chr5	78.788	33	6	1	1	33	24070126
lcl|GQ849	Chr5	95.455	22	1	0	125	146	24068437
lcl|GQ849	Chr5	58.621	29	5	1	135	163	24068029
lcl|GQ849	Chr4	71	100	28	1	62	161	20739869
lcl|GQ849	Chr4	42.857	161	31	2	62	161	22463251
lcl|GQ849	Chr1	71.429	98	27	1	62	159	37514005
lcl|GQ849	Chr12	62.963	108	31	3	62	164	12808313
lcl|GQ849	Chr12	62.857	105	36	2	62	164	12792757
lcl|GQ849	Chr2	48.305	118	30	1	75	161	15225499
lcl|GQ849	Chr2	54.43	79	19	1	100	161	15204350
lcl|GQ849	Chr2	66.667	30	10	0	75	104	15204468
lcl|GQ849	Chr2	83.333	18	3	0	62	79	20134900
lcl|GQ849	Chr2	75	16	4	0	62	77	15225769
lcl|GQ849	Chr3	73.81	42	8	1	126	164	15647702
lcl|GQ849	Chr3	57.143	35	15	0	76	110	15647458
lcl|GQ849	Chr3	75	16	4	0	62	77	15647325
lcl|GQ849	Chr7	60	20	8	0	91	110	11774928

TBLASTN 2.10.1+
Query: lcl|GQ849328.1_prot_ACX30889.1_1 [gene=CenH3] [protein=CENH3] [protein_id=ACX30
Database: Nippon
Fields: query acc.ver, subject acc.ver, % identity, alignment length, mismatches, gap opens, q. s
26 hits found

lcl|GQ849	Chr6	71	100	28	1	62	161	3040837
lcl|GQ849	Chr6	71	100	28	1	62	161	3055514
lcl|GQ849	Chr6	71	100	28	1	62	161	3056279
lcl|GQ849	Chr6	71	100	28	1	62	161	3046867
lcl|GQ849	Chr6	71	100	28	1	62	161	1646006

lcl|GQ849	Chr11	71	100	28	1	62	161	2626112
lcl|GQ849	Chr5	71	100	28	1	62	161	21499987
lcl|GQ849	Chr5	95.349	43	2	0	55	97	24069178
lcl|GQ849	Chr5	100	16	0	0	97	112	24068958
lcl|GQ849	Chr5	87.879	33	3	1	1	33	24070126
lcl|GQ849	Chr5	95.455	22	1	0	125	146	24068437
lcl|GQ849	Chr5	58.621	29	5	1	135	163	24068029
lcl|GQ849	Chr4	71	100	28	1	62	161	20739869
lcl|GQ849	Chr4	42.857	161	31	2	62	161	22463251
lcl|GQ849	Chr1	71.429	98	27	1	62	159	37514005
lcl|GQ849	Chr12	62.963	108	31	3	62	164	12808313
lcl|GQ849	Chr12	62.857	105	36	2	62	164	12792757
lcl|GQ849	Chr2	48.305	118	30	1	75	161	15225499
lcl|GQ849	Chr2	54.43	79	19	1	100	161	15204350
lcl|GQ849	Chr2	66.667	30	10	0	75	104	15204468
lcl|GQ849	Chr2	83.333	18	3	0	62	79	20134900
lcl|GQ849	Chr2	75	16	4	0	62	77	15225769
lcl|GQ849	Chr3	73.81	42	8	1	126	164	15647702
lcl|GQ849	Chr3	57.143	35	15	0	76	110	15647458
lcl|GQ849	Chr3	75	16	4	0	62	77	15647325
lcl|GQ849	Chr7	60	20	8	0	91	110	11774928

TBLASTN 2.10.1+
Query: lcl|KJ651124.1_prot_AJO61187.1_1 [protein=CenH3] [protein_id=AJO61187.1] [location=
Database: Nippon
Fields: query acc.ver, subject acc.ver, % identity, alignment length, mismatches, gap opens, q. s
20 hits found

lcl|KJ6511	Chr6	49.533	107	53	1	12	118	1646033
lcl|KJ6511	Chr6	49.533	107	53	1	12	118	3040864
lcl|KJ6511	Chr6	49.533	107	53	1	12	118	3055541
lcl|KJ6511	Chr6	49.533	107	53	1	12	118	3056252
lcl|KJ6511	Chr6	49.533	107	53	1	12	118	3046894
lcl|KJ6511	Chr5	49.533	107	53	1	12	118	21500014
lcl|KJ6511	Chr5	56.098	41	18	0	16	56	24069172
lcl|KJ6511	Chr5	59.259	27	11	0	56	82	24068958
lcl|KJ6511	Chr5	50	30	15	0	78	107	24068455
lcl|KJ6511	Chr11	49.533	107	53	1	12	118	2626139
lcl|KJ6511	Chr4	49.533	107	53	1	12	118	20739842
lcl|KJ6511	Chr4	32.143	168	53	3	12	118	22463224
lcl|KJ6511	Chr1	49.533	107	53	1	12	118	37513978
lcl|KJ6511	Chr12	47.664	107	55	1	12	118	12808340
lcl|KJ6511	Chr12	47.959	98	50	1	21	118	12792757
lcl|KJ6511	Chr2	36.207	116	43	1	34	118	15225499
lcl|KJ6511	Chr2	40.299	67	35	1	52	118	15204305
lcl|KJ6511	Chr2	60	30	12	0	34	63	15204468
lcl|KJ6511	Chr3	52	50	24	0	69	118	15647654
lcl|KJ6511	Chr3	58.621	29	12	0	35	63	15647458

TBLASTN 2.10.1+
Query: lcl|KJ651120.1_prot_AJO61183.1_1 [protein=CenH3] [protein_id=AJO61183.1] [location=

Database: Nippon
Fields: query acc.ver, subject acc.ver, % identity, alignment length, mismatches, gap opens, q. s
22 hits found

lcl|KJ6511	Chr5	50	100	48	2	12	110	21500014
lcl|KJ6511	Chr5	58.537	41	16	1	16	55	24069172
lcl|KJ6511	Chr5	59.259	27	11	0	55	81	24068958
lcl|KJ6511	Chr5	50	30	15	0	77	106	24068455
lcl|KJ6511	Chr6	50	100	48	2	12	110	3040864
lcl|KJ6511	Chr6	50	100	48	2	12	110	3056252
lcl|KJ6511	Chr6	50	100	48	2	12	110	3055541
lcl|KJ6511	Chr6	49	100	49	2	12	110	1646033
lcl|KJ6511	Chr6	50	100	48	2	12	110	3046894
lcl|KJ6511	Chr1	50	100	48	2	12	110	37513978
lcl|KJ6511	Chr11	50	100	48	2	12	110	2626139
lcl|KJ6511	Chr4	50	100	48	2	12	110	20739842
lcl|KJ6511	Chr4	37.037	108	37	2	34	110	22463383
lcl|KJ6511	Chr4	24.419	86	53	2	16	89	358622
lcl|KJ6511	Chr12	48	100	50	2	12	110	12808340
lcl|KJ6511	Chr12	47	100	51	2	12	110	12792784
lcl|KJ6511	Chr3	51.163	43	21	0	68	110	15647654
lcl|KJ6511	Chr3	48.571	35	18	0	34	68	15647458
lcl|KJ6511	Chr2	33.945	109	41	1	33	110	15225499
lcl|KJ6511	Chr2	38.333	60	32	1	51	110	15204305
lcl|KJ6511	Chr2	58.621	29	12	0	34	62	15204465
lcl|KJ6511	Chr8	41.935	31	18	0	54	84	23860296

TBLASTN 2.10.1+
Query: lcl|KJ651113.1_prot_AJO61176.1_1 [protein=CenH3] [protein_id=AJO61176.1] [location=
Database: Nippon
Fields: query acc.ver, subject acc.ver, % identity, alignment length, mismatches, gap opens, q. s
23 hits found

lcl|KJ6511	Chr6	53.846	91	41	1	20	110	3056279
lcl|KJ6511	Chr6	51	100	48	1	11	110	3040864
lcl|KJ6511	Chr6	53.846	91	41	1	20	110	3055514
lcl|KJ6511	Chr6	53.846	91	41	1	20	110	1646006
lcl|KJ6511	Chr6	53.846	91	41	1	20	110	3046867
lcl|KJ6511	Chr5	51	100	48	1	11	110	21500014
lcl|KJ6511	Chr5	67.568	37	12	0	19	55	24069160
lcl|KJ6511	Chr5	59.259	27	11	0	55	81	24068958
lcl|KJ6511	Chr5	53.333	30	14	0	77	106	24068455
lcl|KJ6511	Chr11	53.846	91	41	1	20	110	2626112
lcl|KJ6511	Chr1	53.846	91	41	1	20	110	37514005
lcl|KJ6511	Chr4	53.846	91	41	1	20	110	20739869
lcl|KJ6511	Chr4	32.895	152	41	3	20	110	22463251
lcl|KJ6511	Chr12	51.648	91	43	1	20	110	12808313
lcl|KJ6511	Chr12	50.549	91	44	1	20	110	12792757
lcl|KJ6511	Chr3	51.163	43	21	0	68	110	15647654
lcl|KJ6511	Chr3	58.621	29	12	0	34	62	15647458
lcl|KJ6511	Chr3	71.429	14	4	0	20	33	15647325

lcl|KJ6511	Chr3	40	35	21	0	2	36	12758493
lcl|KJ6511	Chr2	34.862	109	40	1	33	110	15225499
lcl|KJ6511	Chr2	38.333	60	32	1	51	110	15204305
lcl|KJ6511	Chr2	60	30	12	0	33	62	15204468
lcl|KJ6511	Chr2	30.986	71	35	1	20	76	20134900

TBLASTN 2.10.1+
Query: lcl|KJ651193.1_prot_AJO61254.1_1 [protein=CenH3] [protein_id=AJO61254.1] [location=
Database: Nippon
Fields: query acc.ver, subject acc.ver, % identity, alignment length, mismatches, gap opens, q. s
25 hits found

lcl|KJ6511	Chr11	57.143	98	41	1	17	114	2626112
lcl|KJ6511	Chr11	38.235	34	21	0	58	91	27980107
lcl|KJ6511	Chr6	57.143	98	41	1	17	114	3056279
lcl|KJ6511	Chr6	57.143	98	41	1	17	114	3040837
lcl|KJ6511	Chr6	57.143	98	41	1	17	114	1646006
lcl|KJ6511	Chr6	57.143	98	41	1	17	114	3055514
lcl|KJ6511	Chr6	57.143	98	41	1	17	114	3046867
lcl|KJ6511	Chr12	53.211	109	50	1	17	125	12808313
lcl|KJ6511	Chr12	51.376	109	52	1	17	125	12792757
lcl|KJ6511	Chr12	22.785	79	51	2	49	127	4809489
lcl|KJ6511	Chr5	57.143	98	41	1	17	114	21499987
lcl|KJ6511	Chr5	63.158	38	14	0	15	52	24069163
lcl|KJ6511	Chr5	54.545	33	14	1	52	83	24068958
lcl|KJ6511	Chr5	60	30	12	0	74	103	24068455
lcl|KJ6511	Chr1	57.143	98	41	1	17	114	37514005
lcl|KJ6511	Chr4	57.143	98	41	1	17	114	20739869
lcl|KJ6511	Chr4	35.849	159	41	3	17	114	22463251
lcl|KJ6511	Chr2	38.793	116	40	2	30	114	15225499
lcl|KJ6511	Chr2	54	50	23	0	65	114	15204269
lcl|KJ6511	Chr2	56.667	30	13	0	30	59	15204468
lcl|KJ6511	Chr2	33.333	42	28	0	59	100	10943085
lcl|KJ6511	Chr3	58	50	21	0	65	114	15647654
lcl|KJ6511	Chr3	40.323	62	34	2	31	92	15647458
lcl|KJ6511	Chr3	71.429	14	4	0	17	30	15647325
lcl|KJ6511	Chr3	33.333	42	28	0	59	100	9963056

TBLASTN 2.10.1+
Query: lcl|KJ651190.1_prot_AJO61251.1_1 [protein=CenH3] [protein_id=AJO61251.1] [location=
Database: Nippon
Fields: query acc.ver, subject acc.ver, % identity, alignment length, mismatches, gap opens, q. s
23 hits found

lcl|KJ6511	Chr6	54.082	98	44	1	17	114	3056279
lcl|KJ6511	Chr6	54.082	98	44	1	17	114	3040837
lcl|KJ6511	Chr6	54.082	98	44	1	17	114	3055514
lcl|KJ6511	Chr6	54.082	98	44	1	17	114	1646006
lcl|KJ6511	Chr6	54.082	98	44	1	17	114	3046867
lcl|KJ6511	Chr11	54.082	98	44	1	17	114	2626112
lcl|KJ6511	Chr12	54.082	98	44	1	17	114	12808313
lcl|KJ6511	Chr12	51.02	98	47	1	17	114	12792757

lcl|KJ6511	Chr5	54.082	98	44	1	17	114	21499987
lcl|KJ6511	Chr5	58.696	46	17	1	7	52	24069181
lcl|KJ6511	Chr5	51.515	33	15	1	52	83	24068958
lcl|KJ6511	Chr5	60	30	12	0	74	103	24068455
lcl|KJ6511	Chr1	54.082	98	44	1	17	114	37514005
lcl|KJ6511	Chr4	54.082	98	44	1	17	114	20739869
lcl|KJ6511	Chr4	33.962	159	44	3	17	114	22463251
lcl|KJ6511	Chr2	38.793	116	40	2	30	114	15225499
lcl|KJ6511	Chr2	54	50	23	0	65	114	15204269
lcl|KJ6511	Chr2	59.259	27	11	0	30	56	15204468
lcl|KJ6511	Chr2	26.23	61	45	0	40	100	10943142
lcl|KJ6511	Chr3	58	50	21	0	65	114	15647654
lcl|KJ6511	Chr3	37.097	62	36	2	31	92	15647458
lcl|KJ6511	Chr3	64.286	14	5	0	17	30	15647325
lcl|KJ6511	Chr3	33.333	39	26	0	62	100	9963065

TBLASTN 2.10.1+
Query: lcl|KJ651189.1_prot_AJO61250.1_1 [protein=CenH3] [protein_id=AJO61250.1] [location=
Database: Nippon
Fields: query acc.ver, subject acc.ver, % identity, alignment length, mismatches, gap opens, q. s
23 hits found

lcl|KJ6511	Chr6	55.102	98	43	1	17	114	3056279
lcl|KJ6511	Chr6	55.102	98	43	1	17	114	3040837
lcl|KJ6511	Chr6	55.102	98	43	1	17	114	3055514
lcl|KJ6511	Chr6	55.102	98	43	1	17	114	1646006
lcl|KJ6511	Chr6	55.102	98	43	1	17	114	3046867
lcl|KJ6511	Chr11	55.102	98	43	1	17	114	2626112
lcl|KJ6511	Chr12	55.102	98	43	1	17	114	12808313
lcl|KJ6511	Chr12	52.041	98	46	1	17	114	12792757
lcl|KJ6511	Chr5	55.102	98	43	1	17	114	21499987
lcl|KJ6511	Chr5	60.87	46	16	1	7	52	24069181
lcl|KJ6511	Chr5	51.515	33	15	1	52	83	24068958
lcl|KJ6511	Chr5	60	30	12	0	74	103	24068455
lcl|KJ6511	Chr4	55.102	98	43	1	17	114	20739869
lcl|KJ6511	Chr4	34.591	159	43	3	17	114	22463251
lcl|KJ6511	Chr1	55.102	98	43	1	17	114	37514005
lcl|KJ6511	Chr2	38.793	116	40	2	30	114	15225499
lcl|KJ6511	Chr2	54	50	23	0	65	114	15204269
lcl|KJ6511	Chr2	55.172	29	13	0	30	58	15204468
lcl|KJ6511	Chr2	33.333	39	26	0	62	100	10943076
lcl|KJ6511	Chr3	58	50	21	0	65	114	15647654
lcl|KJ6511	Chr3	35.938	64	38	2	31	94	15647458
lcl|KJ6511	Chr3	71.429	14	4	0	17	30	15647325
lcl|KJ6511	Chr3	33.333	39	26	0	62	100	9963065

TBLASTN 2.10.1+
Query: lcl|KJ651187.1_prot_AJO61248.1_1 [protein=CenH3] [protein_id=AJO61248.1] [location=
Database: Nippon
Fields: query acc.ver, subject acc.ver, % identity, alignment length, mismatches, gap opens, q. s
24 hits found

lcl|KJ6511	Chr6	55.102	98	43	1	17	114	3056279
lcl|KJ6511	Chr6	55.102	98	43	1	17	114	3040837
lcl|KJ6511	Chr6	55.102	98	43	1	17	114	1646006
lcl|KJ6511	Chr6	55.102	98	43	1	17	114	3055514
lcl|KJ6511	Chr6	55.102	98	43	1	17	114	3046867
lcl|KJ6511	Chr11	55.102	98	43	1	17	114	2626112
lcl|KJ6511	Chr12	55.102	98	43	1	17	114	12808313
lcl|KJ6511	Chr12	52.041	98	46	1	17	114	12792757
lcl|KJ6511	Chr5	55.102	98	43	1	17	114	21499987
lcl|KJ6511	Chr5	60.976	41	16	0	12	52	24069172
lcl|KJ6511	Chr5	51.515	33	15	1	52	83	24068958
lcl|KJ6511	Chr5	60	30	12	0	74	103	24068455
lcl|KJ6511	Chr1	55.102	98	43	1	17	114	37514005
lcl|KJ6511	Chr4	55.102	98	43	1	17	114	20739869
lcl|KJ6511	Chr4	34.591	159	43	3	17	114	22463251
lcl|KJ6511	Chr2	37.931	116	41	2	30	114	15225499
lcl|KJ6511	Chr2	54	50	23	0	65	114	15204269
lcl|KJ6511	Chr2	57.143	28	12	0	30	57	15204468
lcl|KJ6511	Chr2	41.935	31	18	0	4	34	11947317
lcl|KJ6511	Chr2	33.333	39	26	0	62	100	10943076
lcl|KJ6511	Chr3	58	50	21	0	65	114	15647654
lcl|KJ6511	Chr3	37.097	62	36	2	31	92	15647458
lcl|KJ6511	Chr3	71.429	14	4	0	17	30	15647325
lcl|KJ6511	Chr3	33.333	39	26	0	62	100	9963065

TBLASTN 2.10.1+
Query: lcl|KJ651185.1_prot_AJO61246.1_1 [protein=CenH3] [protein_id=AJO61246.1] [location=
Database: Nippon
Fields: query acc.ver, subject acc.ver, % identity, alignment length, mismatches, gap opens, q. s
23 hits found

lcl|KJ6511	Chr6	55.102	98	43	1	17	114	3040837
lcl|KJ6511	Chr6	55.102	98	43	1	17	114	3056279
lcl|KJ6511	Chr6	55.102	98	43	1	17	114	1646006
lcl|KJ6511	Chr6	55.102	98	43	1	17	114	3055514
lcl|KJ6511	Chr6	55.102	98	43	1	17	114	3046867
lcl|KJ6511	Chr11	55.102	98	43	1	17	114	2626112
lcl|KJ6511	Chr5	55.102	98	43	1	17	114	21499987
lcl|KJ6511	Chr5	58.696	46	17	1	7	52	24069181
lcl|KJ6511	Chr5	51.515	33	15	1	52	83	24068958
lcl|KJ6511	Chr5	60	30	12	0	74	103	24068455
lcl|KJ6511	Chr12	55.102	98	43	1	17	114	12808313
lcl|KJ6511	Chr12	52.041	98	46	1	17	114	12792757
lcl|KJ6511	Chr1	55.102	98	43	1	17	114	37514005
lcl|KJ6511	Chr4	55.102	98	43	1	17	114	20739869
lcl|KJ6511	Chr4	34.591	159	43	3	17	114	22463251
lcl|KJ6511	Chr2	37.931	116	41	2	30	114	15225499
lcl|KJ6511	Chr2	54	50	23	0	65	114	15204269
lcl|KJ6511	Chr2	55.172	29	13	0	30	58	15204468
lcl|KJ6511	Chr2	30.435	69	37	2	43	100	10943166

lcl|KJ6511	Chr3	58	50	21	0	65	114	15647654
lcl|KJ6511	Chr3	35.938	64	38	2	31	94	15647458
lcl|KJ6511	Chr3	71.429	14	4	0	17	30	15647325
lcl|KJ6511	Chr3	33.333	39	26	0	62	100	9963065

TBLASTN 2.10.1+
Query: lcl|KJ651184.1_prot_AJO61245.1_1 [protein=CenH3] [protein_id=AJO61245.1] [location=
Database: Nippon
Fields: query acc.ver, subject acc.ver, % identity, alignment length, mismatches, gap opens, q. s
20 hits found

lcl|KJ6511	Chr11	53.846	91	41	1	24	114	2626091
lcl|KJ6511	Chr6	53.846	91	41	1	24	114	3040816
lcl|KJ6511	Chr6	53.846	91	41	1	24	114	3056300
lcl|KJ6511	Chr6	53.846	91	41	1	24	114	3055493
lcl|KJ6511	Chr6	53.846	91	41	1	24	114	3046846
lcl|KJ6511	Chr6	53.846	91	41	1	24	114	1645985
lcl|KJ6511	Chr5	53.846	91	41	1	24	114	21499966
lcl|KJ6511	Chr5	68.966	29	9	0	24	52	24069136
lcl|KJ6511	Chr5	48.485	33	16	1	52	83	24068958
lcl|KJ6511	Chr5	60	30	12	0	74	103	24068455
lcl|KJ6511	Chr1	53.846	91	41	1	24	114	37514026
lcl|KJ6511	Chr4	53.846	91	41	1	24	114	20739890
lcl|KJ6511	Chr4	40	115	38	2	31	114	22463383
lcl|KJ6511	Chr12	52.747	91	42	1	24	114	12808292
lcl|KJ6511	Chr12	50.549	91	44	1	24	114	12792736
lcl|KJ6511	Chr2	38.261	115	40	2	31	114	15225496
lcl|KJ6511	Chr2	54	50	23	0	65	114	15204269
lcl|KJ6511	Chr2	53.571	28	13	0	31	58	15204465
lcl|KJ6511	Chr3	58	50	21	0	65	114	15647654
lcl|KJ6511	Chr3	35.938	64	38	2	31	94	15647458

TBLASTN 2.10.1+
Query: lcl|KJ651182.1_prot_AJO61244.1_1 [protein=CenH3] [protein_id=AJO61244.1] [location=
Database: Nippon
Fields: query acc.ver, subject acc.ver, % identity, alignment length, mismatches, gap opens, q. s
20 hits found

lcl|KJ6511	Chr11	53.846	91	41	1	24	114	2626091
lcl|KJ6511	Chr6	53.846	91	41	1	24	114	3040816
lcl|KJ6511	Chr6	53.846	91	41	1	24	114	3056300
lcl|KJ6511	Chr6	53.846	91	41	1	24	114	3055493
lcl|KJ6511	Chr6	53.846	91	41	1	24	114	3046846
lcl|KJ6511	Chr6	53.846	91	41	1	24	114	1645985
lcl|KJ6511	Chr5	53.846	91	41	1	24	114	21499966
lcl|KJ6511	Chr5	65.517	29	10	0	24	52	24069136
lcl|KJ6511	Chr5	51.515	33	15	1	52	83	24068958
lcl|KJ6511	Chr5	60	30	12	0	74	103	24068455
lcl|KJ6511	Chr1	53.846	91	41	1	24	114	37514026
lcl|KJ6511	Chr4	53.846	91	41	1	24	114	20739890
lcl|KJ6511	Chr4	39.13	115	39	2	31	114	22463383
lcl|KJ6511	Chr12	52.747	91	42	1	24	114	12808292

lcl|KJ6511 Chr12	50.549	91	44	1	24	114	12792736
lcl|KJ6511 Chr2	38.261	115	40	2	31	114	15225496
lcl|KJ6511 Chr2	54	50	23	0	65	114	15204269
lcl|KJ6511 Chr2	53.571	28	13	0	31	58	15204465
lcl|KJ6511 Chr3	58	50	21	0	65	114	15647654
lcl|KJ6511 Chr3	35.938	64	38	2	31	94	15647458

TBLASTN 2.10.1+
Query: lcl|KJ651181.1_prot_AJO61243.1_1 [protein=CenH3] [protein_id=AJO61243.1] [location=
Database: Nippon
Fields: query acc.ver, subject acc.ver, % identity, alignment length, mismatches, gap opens, q. s
25 hits found

lcl|KJ6511 Chr11	47.761	134	49	2	1	114	2626220
lcl|KJ6511 Chr11	41.176	34	20	0	58	91	27980107
lcl|KJ6511 Chr6	47.761	134	49	2	1	114	3040945
lcl|KJ6511 Chr6	47.761	134	49	2	1	114	3055622
lcl|KJ6511 Chr6	47.761	134	49	2	1	114	3056171
lcl|KJ6511 Chr6	57.143	98	41	1	17	114	1646006
lcl|KJ6511 Chr6	57.143	98	41	1	17	114	3046867
lcl|KJ6511 Chr5	47.761	134	49	2	1	114	21500095
lcl|KJ6511 Chr5	60.87	46	16	1	7	52	24069181
lcl|KJ6511 Chr5	54.545	33	14	1	52	83	24068958
lcl|KJ6511 Chr5	60	30	12	0	74	103	24068455
lcl|KJ6511 Chr12	58.163	98	40	1	17	114	12808313
lcl|KJ6511 Chr12	54.082	98	44	1	17	114	12792757
lcl|KJ6511 Chr4	47.761	134	49	2	1	114	20739761
lcl|KJ6511 Chr4	35.849	159	41	3	17	114	22463251
lcl|KJ6511 Chr1	57.143	98	41	1	17	114	37514005
lcl|KJ6511 Chr2	40.517	116	38	2	30	114	15225499
lcl|KJ6511 Chr2	54	50	23	0	65	114	15204269
lcl|KJ6511 Chr2	62.069	29	11	0	30	58	15204468
lcl|KJ6511 Chr2	33.333	39	26	0	62	100	10943076
lcl|KJ6511 Chr3	58	50	21	0	65	114	15647654
lcl|KJ6511 Chr3	37.5	64	37	2	31	94	15647458
lcl|KJ6511 Chr3	71.429	14	4	0	17	30	15647325
lcl|KJ6511 Chr3	33.333	39	26	0	62	100	9963065
lcl|KJ6511 Chr8	38.636	44	26	1	11	54	23882895

TBLASTN 2.10.1+
Query: lcl|KJ651179.1_prot_AJO61241.1_1 [protein=CenH3] [protein_id=AJO61241.1] [location=
Database: Nippon
Fields: query acc.ver, subject acc.ver, % identity, alignment length, mismatches, gap opens, q. s
25 hits found

lcl|KJ6511 Chr11	47.015	134	50	2	1	114	2626220
lcl|KJ6511 Chr11	40	35	21	0	57	91	27980110
lcl|KJ6511 Chr6	47.015	134	50	2	1	114	3040945
lcl|KJ6511 Chr6	47.015	134	50	2	1	114	3055622
lcl|KJ6511 Chr6	47.015	134	50	2	1	114	3056171
lcl|KJ6511 Chr6	56.122	98	42	1	17	114	3046867
lcl|KJ6511 Chr6	56.122	98	42	1	17	114	1646006

lcl|KJ6511	Chr5	47.015	134	50	2	1	114	21500095
lcl|KJ6511	Chr5	60.87	46	16	1	7	52	24069181
lcl|KJ6511	Chr5	54.545	33	14	1	52	83	24068958
lcl|KJ6511	Chr5	60	30	12	0	74	103	24068455
lcl|KJ6511	Chr12	57.143	98	41	1	17	114	12808313
lcl|KJ6511	Chr12	53.061	98	45	1	17	114	12792757
lcl|KJ6511	Chr4	47.015	134	50	2	1	114	20739761
lcl|KJ6511	Chr4	35.22	159	42	3	17	114	22463251
lcl|KJ6511	Chr1	56.122	98	42	1	17	114	37514005
lcl|KJ6511	Chr3	56	50	22	0	65	114	15647654
lcl|KJ6511	Chr3	68	25	8	0	31	55	15647458
lcl|KJ6511	Chr3	71.429	14	4	0	17	30	15647325
lcl|KJ6511	Chr3	31.818	44	30	0	57	100	9963050
lcl|KJ6511	Chr2	39.655	116	39	2	30	114	15225499
lcl|KJ6511	Chr2	52	50	24	0	65	114	15204269
lcl|KJ6511	Chr2	69.231	26	8	0	30	55	15204468
lcl|KJ6511	Chr2	31.818	44	30	0	57	100	10943091
lcl|KJ6511	Chr8	38.776	49	29	1	11	59	23882895

TBLASTN 2.10.1+
Query: lcl|KJ651178.1_prot_AJO61240.1_1 [protein=CenH3] [protein_id=AJO61240.1] [location=
Database: Nippon
Fields: query acc.ver, subject acc.ver, % identity, alignment length, mismatches, gap opens, q. s
20 hits found

lcl|KJ6511	Chr6	53.061	98	45	1	17	114	3040837
lcl|KJ6511	Chr6	53.061	98	45	1	17	114	3056279
lcl|KJ6511	Chr6	53.061	98	45	1	17	114	3055514
lcl|KJ6511	Chr6	53.061	98	45	1	17	114	3046867
lcl|KJ6511	Chr6	53.061	98	45	1	17	114	1646006
lcl|KJ6511	Chr5	53.061	98	45	1	17	114	21499987
lcl|KJ6511	Chr5	58.14	43	18	0	10	52	24069178
lcl|KJ6511	Chr5	51.515	33	15	1	52	83	24068958
lcl|KJ6511	Chr5	60	30	12	0	74	103	24068455
lcl|KJ6511	Chr11	53.061	98	45	1	17	114	2626112
lcl|KJ6511	Chr11	38.71	31	19	0	29	59	18697002
lcl|KJ6511	Chr4	53.061	98	45	1	17	114	20739869
lcl|KJ6511	Chr4	32.075	159	47	2	17	114	22463251
lcl|KJ6511	Chr1	53.061	98	45	1	17	114	37514005
lcl|KJ6511	Chr12	53.061	98	45	1	17	114	12808313
lcl|KJ6511	Chr12	50	98	48	1	17	114	12792757
lcl|KJ6511	Chr2	37.931	116	41	2	30	114	15225499
lcl|KJ6511	Chr2	54	50	23	0	65	114	15204269
lcl|KJ6511	Chr2	45.455	33	18	0	30	62	15204468
lcl|KJ6511	Chr3	58	50	21	0	65	114	15647654

TBLASTN 2.10.1+
Query: lcl|KJ651174.1_prot_AJO61236.1_1 [protein=CenH3] [protein_id=AJO61236.1] [location=
Database: Nippon
Fields: query acc.ver, subject acc.ver, % identity, alignment length, mismatches, gap opens, q. s
21 hits found

lcl|KJ6511 Chr6	54.082	98	44	1	17	114	3056279
lcl|KJ6511 Chr6	54.082	98	44	1	17	114	1646006
lcl|KJ6511 Chr6	54.082	98	44	1	17	114	3040837
lcl|KJ6511 Chr6	54.082	98	44	1	17	114	3055514
lcl|KJ6511 Chr6	54.082	98	44	1	17	114	3046867
lcl|KJ6511 Chr11	54.082	98	44	1	17	114	2626112
lcl|KJ6511 Chr12	54.082	98	44	1	17	114	12808313
lcl|KJ6511 Chr12	51.02	98	47	1	17	114	12792757
lcl|KJ6511 Chr5	54.082	98	44	1	17	114	21499987
lcl|KJ6511 Chr5	60.87	46	16	1	7	52	24069181
lcl|KJ6511 Chr5	53.125	32	14	1	52	82	24068958
lcl|KJ6511 Chr5	56.667	30	13	0	74	103	24068455
lcl|KJ6511 Chr4	54.082	98	44	1	17	114	20739869
lcl|KJ6511 Chr4	33.962	159	44	3	17	114	22463251
lcl|KJ6511 Chr1	54.082	98	44	1	17	114	37514005
lcl|KJ6511 Chr2	37.931	116	41	2	30	114	15225499
lcl|KJ6511 Chr2	52	50	24	0	65	114	15204269
lcl|KJ6511 Chr2	55.172	29	13	0	30	58	15204468
lcl|KJ6511 Chr3	56	50	22	0	65	114	15647654
lcl|KJ6511 Chr3	37.097	62	36	2	31	92	15647458
lcl|KJ6511 Chr3	71.429	14	4	0	17	30	15647325

TBLASTN 2.10.1+
Query: lcl|KJ651169.1_prot_AJO61231.1_1 [protein=CenH3] [protein_id=AJO61231.1] [location=
Database: Nippon
Fields: query acc.ver, subject acc.ver, % identity, alignment length, mismatches, gap opens, q. s
20 hits found

lcl|KJ6511 Chr11	50	134	52	3	1	120	2626220
lcl|KJ6511 Chr6	50	134	52	3	1	120	3040945
lcl|KJ6511 Chr6	50	134	52	3	1	120	3055622
lcl|KJ6511 Chr6	50	134	52	3	1	120	3056171
lcl|KJ6511 Chr6	59.574	94	37	1	27	120	3046855
lcl|KJ6511 Chr6	58.511	94	38	1	27	120	1645994
lcl|KJ6511 Chr5	50	134	52	3	1	120	21500095
lcl|KJ6511 Chr5	81.25	32	6	0	27	58	24069145
lcl|KJ6511 Chr5	56.522	23	10	0	87	109	24068434
lcl|KJ6511 Chr4	50	134	52	3	1	120	20739761
lcl|KJ6511 Chr4	33.974	156	42	2	26	120	22463260
lcl|KJ6511 Chr1	59.574	94	37	1	27	120	37514017
lcl|KJ6511 Chr12	45.522	134	58	3	1	120	12808421
lcl|KJ6511 Chr12	43.284	134	61	3	1	120	12792865
lcl|KJ6511 Chr2	38.793	116	40	1	36	120	15225499
lcl|KJ6511 Chr2	55.882	34	15	0	87	120	15204221
lcl|KJ6511 Chr2	53.125	32	15	0	36	67	15204468
lcl|KJ6511 Chr3	61.765	34	13	0	87	120	15647702
lcl|KJ6511 Chr3	45.714	35	19	0	37	71	15647458
lcl|KJ6511 Chr3	80	10	2	0	27	36	15647337

TBLASTN 2.10.1+
Query: lcl|KJ651166.1_prot_AJO61228.1_1 [protein=CenH3] [protein_id=AJO61228.1] [location=

Database: Nippon
Fields: query acc.ver, subject acc.ver, % identity, alignment length, mismatches, gap opens, q. s
21 hits found

lcl|KJ6511	Chr6	49.153	118	58	2	8	124	3040897
lcl|KJ6511	Chr6	49.153	118	58	2	8	124	3055574
lcl|KJ6511	Chr6	49.153	118	58	2	8	124	3056219
lcl|KJ6511	Chr6	50	112	54	2	14	124	3046909
lcl|KJ6511	Chr6	52.83	106	48	2	20	124	1646030
lcl|KJ6511	Chr6	50	28	14	0	9	36	1833569
lcl|KJ6511	Chr11	49.153	118	58	2	8	124	2626172
lcl|KJ6511	Chr5	49.153	118	58	2	8	124	21500047
lcl|KJ6511	Chr5	60.976	41	16	0	22	62	24069172
lcl|KJ6511	Chr5	56.522	23	10	0	91	113	24068434
lcl|KJ6511	Chr5	46.429	28	15	0	90	117	10354962
lcl|KJ6511	Chr4	49.153	118	58	2	8	124	20739809
lcl|KJ6511	Chr4	39.13	115	39	2	41	124	22463383
lcl|KJ6511	Chr1	50	112	54	2	14	124	37513963
lcl|KJ6511	Chr12	51.546	97	46	1	28	124	12808310
lcl|KJ6511	Chr12	44.167	120	65	2	6	124	12792823
lcl|KJ6511	Chr2	37.069	116	42	1	40	124	15225499
lcl|KJ6511	Chr2	46.269	67	31	1	58	124	15204305
lcl|KJ6511	Chr2	53.571	28	13	0	40	67	15204468
lcl|KJ6511	Chr3	61.765	34	13	0	91	124	15647702
lcl|KJ6511	Chr3	42.857	35	20	0	41	75	15647458

TBLASTN 2.10.1+
Query: lcl|KJ651163.1_prot_AJO61225.1_1 [protein=CenH3] [protein_id=AJO61225.1] [location=
Database: Nippon
Fields: query acc.ver, subject acc.ver, % identity, alignment length, mismatches, gap opens, q. s
20 hits found

lcl|KJ6511	Chr6	53.061	98	45	1	27	124	3040837
lcl|KJ6511	Chr6	53.061	98	45	1	27	124	3056279
lcl|KJ6511	Chr6	53.061	98	45	1	27	124	3055514
lcl|KJ6511	Chr6	53.061	98	45	1	27	124	3046867
lcl|KJ6511	Chr6	54.082	98	44	1	27	124	1646006
lcl|KJ6511	Chr5	53.061	98	45	1	27	124	21499987
lcl|KJ6511	Chr5	53.191	47	22	0	16	62	24069190
lcl|KJ6511	Chr5	56.522	23	10	0	91	113	24068434
lcl|KJ6511	Chr5	46.429	28	15	0	90	117	10354962
lcl|KJ6511	Chr11	53.061	98	45	1	27	124	2626112
lcl|KJ6511	Chr1	53.061	98	45	1	27	124	37514005
lcl|KJ6511	Chr4	53.061	98	45	1	27	124	20739869
lcl|KJ6511	Chr4	40	115	38	2	41	124	22463383
lcl|KJ6511	Chr12	52.041	98	46	1	27	124	12808313
lcl|KJ6511	Chr12	48.98	98	49	1	27	124	12792757
lcl|KJ6511	Chr2	37.931	116	41	1	40	124	15225499
lcl|KJ6511	Chr2	47.761	67	30	1	58	124	15204305
lcl|KJ6511	Chr2	46.875	32	17	0	40	71	15204468
lcl|KJ6511	Chr3	61.765	34	13	0	91	124	15647702

lcl|KJ6511	Chr3	42.857	35	20	0	41	75	15647458

TBLASTN 2.10.1+
Query: lcl|KJ651160.1_prot_AJO61222.1_1 [protein=CenH3] [protein_id=AJO61222.1] [location=
Database: Nippon
Fields: query acc.ver, subject acc.ver, % identity, alignment length, mismatches, gap opens, q. s
24 hits found

lcl|KJ6511	Chr6	58.242	91	37	1	24	114	1645985
lcl|KJ6511	Chr6	57.143	91	38	1	24	114	3040816
lcl|KJ6511	Chr6	57.143	91	38	1	24	114	3055493
lcl|KJ6511	Chr6	57.143	91	38	1	24	114	3056300
lcl|KJ6511	Chr6	57.143	91	38	1	24	114	3046846
lcl|KJ6511	Chr11	57.143	91	38	1	24	114	2626091
lcl|KJ6511	Chr11	38.235	34	21	0	58	91	27980107
lcl|KJ6511	Chr5	57.143	91	38	1	24	114	21499966
lcl|KJ6511	Chr5	79.31	29	6	0	24	52	24069136
lcl|KJ6511	Chr5	51.351	37	17	1	52	87	24068958
lcl|KJ6511	Chr5	54.545	33	15	0	71	103	24068464
lcl|KJ6511	Chr4	57.143	91	38	1	24	114	20739890
lcl|KJ6511	Chr4	40.87	115	37	2	31	114	22463383
lcl|KJ6511	Chr1	57.143	91	38	1	24	114	37514026
lcl|KJ6511	Chr1	26.923	78	48	2	42	114	30558714
lcl|KJ6511	Chr12	56.044	91	39	1	24	114	12808292
lcl|KJ6511	Chr12	53.846	91	41	1	24	114	12792736
lcl|KJ6511	Chr2	40.517	116	38	2	30	114	15225499
lcl|KJ6511	Chr2	46.269	67	31	1	48	114	15204305
lcl|KJ6511	Chr2	65.385	26	9	0	30	55	15204468
lcl|KJ6511	Chr2	35.897	39	25	0	62	100	10943076
lcl|KJ6511	Chr3	60	50	20	0	65	114	15647654
lcl|KJ6511	Chr3	35.938	64	38	2	31	94	15647458
lcl|KJ6511	Chr3	35.897	39	25	0	62	100	9963065

TBLASTN 2.10.1+
Query: lcl|KJ651157.1_prot_AJO61219.1_1 [protein=CenH3] [protein_id=AJO61219.1] [location=
Database: Nippon
Fields: query acc.ver, subject acc.ver, % identity, alignment length, mismatches, gap opens, q. s
21 hits found

lcl|KJ6511	Chr12	52.577	97	45	1	22	118	12808310
lcl|KJ6511	Chr12	48.454	97	49	1	22	118	12792754
lcl|KJ6511	Chr6	51.546	97	46	1	22	118	1646003
lcl|KJ6511	Chr6	51.546	97	46	1	22	118	3040834
lcl|KJ6511	Chr6	52.128	94	44	1	25	118	3055502
lcl|KJ6511	Chr6	51.546	97	46	1	22	118	3056282
lcl|KJ6511	Chr6	51.546	97	46	1	22	118	3046864
lcl|KJ6511	Chr11	52.128	94	44	1	25	118	2626100
lcl|KJ6511	Chr5	51.546	97	46	1	22	118	21499984
lcl|KJ6511	Chr5	57.5	40	17	0	17	56	24069169
lcl|KJ6511	Chr5	51.852	27	13	0	56	82	24068958
lcl|KJ6511	Chr5	51.515	33	16	0	75	107	24068464
lcl|KJ6511	Chr4	51.546	97	46	1	22	118	20739872

lcl|KJ6511	Chr4	31.361	169	55	3	11	118	22463221
lcl|KJ6511	Chr1	51.546	97	46	1	22	118	37514008
lcl|KJ6511	Chr2	35.345	116	44	1	34	118	15225499
lcl|KJ6511	Chr2	47.761	67	30	1	52	118	15204305
lcl|KJ6511	Chr2	32.609	46	29	1	28	71	3450588
lcl|KJ6511	Chr3	60	50	20	0	69	118	15647654
lcl|KJ6511	Chr3	30.882	68	45	1	35	100	26238454
lcl|KJ6511	Chr10	32.075	53	28	1	34	86	19897764

TBLASTN 2.10.1+
Query: lcl|KJ651154.1_prot_AJO61216.1_1 [protein=CenH3] [protein_id=AJO61216.1] [location=
Database: Nippon
Fields: query acc.ver, subject acc.ver, % identity, alignment length, mismatches, gap opens, q. s
19 hits found

lcl|KJ6511	Chr11	57.143	91	38	1	24	114	2626091
lcl|KJ6511	Chr6	57.143	91	38	1	24	114	3040816
lcl|KJ6511	Chr6	57.143	91	38	1	24	114	3055493
lcl|KJ6511	Chr6	57.143	91	38	1	24	114	3046846
lcl|KJ6511	Chr6	57.143	91	38	1	24	114	3056300
lcl|KJ6511	Chr6	58.242	91	37	1	24	114	1645985
lcl|KJ6511	Chr5	57.143	91	38	1	24	114	21499966
lcl|KJ6511	Chr5	82.759	29	5	0	24	52	24069136
lcl|KJ6511	Chr5	59.091	22	9	0	82	103	24068431
lcl|KJ6511	Chr4	57.143	91	38	1	24	114	20739890
lcl|KJ6511	Chr4	40.87	115	37	2	31	114	22463383
lcl|KJ6511	Chr12	57.143	91	38	1	24	114	12808292
lcl|KJ6511	Chr12	53.846	91	41	1	24	114	12792736
lcl|KJ6511	Chr1	57.143	91	38	1	24	114	37514026
lcl|KJ6511	Chr2	40.517	116	38	2	30	114	15225499
lcl|KJ6511	Chr2	54.545	33	15	0	82	114	15204218
lcl|KJ6511	Chr2	58.621	29	12	0	30	58	15204468
lcl|KJ6511	Chr3	60.606	33	13	0	82	114	15647705
lcl|KJ6511	Chr3	35.484	62	37	1	31	92	15647458

TBLASTN 2.10.1+
Query: lcl|KJ651153.1_prot_AJO61215.1_1 [protein=CenH3] [protein_id=AJO61215.1] [location=
Database: Nippon
Fields: query acc.ver, subject acc.ver, % identity, alignment length, mismatches, gap opens, q. s
22 hits found

lcl|KJ6511	Chr6	56.044	91	39	1	24	114	1645985
lcl|KJ6511	Chr6	54.945	91	40	1	24	114	3040816
lcl|KJ6511	Chr6	54.945	91	40	1	24	114	3055493
lcl|KJ6511	Chr6	54.945	91	40	1	24	114	3056300
lcl|KJ6511	Chr6	54.945	91	40	1	24	114	3046846
lcl|KJ6511	Chr11	54.945	91	40	1	24	114	2626091
lcl|KJ6511	Chr12	54.945	91	40	1	24	114	12808292
lcl|KJ6511	Chr12	51.648	91	43	1	24	114	12792736
lcl|KJ6511	Chr5	54.945	91	40	1	24	114	21499966
lcl|KJ6511	Chr5	82.759	29	5	0	24	52	24069136
lcl|KJ6511	Chr5	58.333	36	14	1	52	86	24068958

lcl|KJ6511	Chr5	48.485	33	17	0	71	103	24068464
lcl|KJ6511	Chr4	54.945	91	40	1	24	114	20739890
lcl|KJ6511	Chr4	39.13	115	39	2	31	114	22463383
lcl|KJ6511	Chr4	41.463	41	23	1	39	79	34485572
lcl|KJ6511	Chr1	54.945	91	40	1	24	114	37514026
lcl|KJ6511	Chr1	31.034	58	31	2	42	94	30558714
lcl|KJ6511	Chr2	39.655	116	39	2	30	114	15225499
lcl|KJ6511	Chr2	44.776	67	32	1	48	114	15204305
lcl|KJ6511	Chr2	58.621	29	12	0	30	58	15204468
lcl|KJ6511	Chr3	56	50	22	0	65	114	15647654
lcl|KJ6511	Chr3	37.097	62	36	2	31	92	15647458

TBLASTN 2.10.1+
Query: lcl|KJ651146.1_prot_AJO61208.1_1 [protein=CenH3] [protein_id=AJO61208.1] [location=
Database: Nippon
Fields: query acc.ver, subject acc.ver, % identity, alignment length, mismatches, gap opens, q. s
23 hits found

lcl|KJ6511	Chr6	58.242	91	37	1	24	114	1645985
lcl|KJ6511	Chr6	57.143	91	38	1	24	114	3040816
lcl|KJ6511	Chr6	57.143	91	38	1	24	114	3055493
lcl|KJ6511	Chr6	57.143	91	38	1	24	114	3056300
lcl|KJ6511	Chr6	57.143	91	38	1	24	114	3046846
lcl|KJ6511	Chr11	57.143	91	38	1	24	114	2626091
lcl|KJ6511	Chr11	38.235	34	21	0	58	91	27980107
lcl|KJ6511	Chr5	57.143	91	38	1	24	114	21499966
lcl|KJ6511	Chr5	82.759	29	5	0	24	52	24069136
lcl|KJ6511	Chr5	51.351	37	17	1	52	87	24068958
lcl|KJ6511	Chr5	54.545	33	15	0	71	103	24068464
lcl|KJ6511	Chr4	57.143	91	38	1	24	114	20739890
lcl|KJ6511	Chr4	40.87	115	37	2	31	114	22463383
lcl|KJ6511	Chr1	57.143	91	38	1	24	114	37514026
lcl|KJ6511	Chr12	56.044	91	39	1	24	114	12808292
lcl|KJ6511	Chr12	53.846	91	41	1	24	114	12792736
lcl|KJ6511	Chr2	39.655	116	39	2	30	114	15225499
lcl|KJ6511	Chr2	46.269	67	31	1	48	114	15204305
lcl|KJ6511	Chr2	58.621	29	12	0	30	58	15204468
lcl|KJ6511	Chr2	35.897	39	25	0	62	100	10943076
lcl|KJ6511	Chr3	60	50	20	0	65	114	15647654
lcl|KJ6511	Chr3	35.938	64	38	2	31	94	15647458
lcl|KJ6511	Chr3	35.897	39	25	0	62	100	9963065

TBLASTN 2.10.1+
Query: lcl|KJ651142.1_prot_AJO61205.1_1 [protein=CenH3] [protein_id=AJO61205.1] [location=
Database: Nippon
Fields: query acc.ver, subject acc.ver, % identity, alignment length, mismatches, gap opens, q. s
23 hits found

lcl|KJ6511	Chr6	57.143	91	38	1	24	114	1645985
lcl|KJ6511	Chr6	56.044	91	39	1	24	114	3040816
lcl|KJ6511	Chr6	56.044	91	39	1	24	114	3055493
lcl|KJ6511	Chr6	56.044	91	39	1	24	114	3056300

lcl|KJ6511 Chr6	56.044	91	39	1	24	114	3046846
lcl|KJ6511 Chr11	56.044	91	39	1	24	114	2626091
lcl|KJ6511 Chr5	56.044	91	39	1	24	114	21499966
lcl|KJ6511 Chr5	79.31	29	6	0	24	52	24069136
lcl|KJ6511 Chr5	51.351	37	17	1	52	87	24068958
lcl|KJ6511 Chr5	55.882	34	15	0	70	103	24068467
lcl|KJ6511 Chr4	56.044	91	39	1	24	114	20739890
lcl|KJ6511 Chr4	40	115	38	2	31	114	22463383
lcl|KJ6511 Chr4	41.026	39	22	1	39	77	34485572
lcl|KJ6511 Chr1	56.044	91	39	1	24	114	37514026
lcl|KJ6511 Chr12	54.945	91	40	1	24	114	12808292
lcl|KJ6511 Chr12	51.648	91	43	1	24	114	12792736
lcl|KJ6511 Chr2	38.793	116	40	2	30	114	15225499
lcl|KJ6511 Chr2	44.776	67	32	1	48	114	15204305
lcl|KJ6511 Chr2	65.385	26	9	0	30	55	15204468
lcl|KJ6511 Chr2	34.091	44	29	0	57	100	10943091
lcl|KJ6511 Chr3	58	50	21	0	65	114	15647654
lcl|KJ6511 Chr3	35.938	64	38	1	31	94	15647458
lcl|KJ6511 Chr3	34.091	44	29	0	57	100	9963050

TBLASTN 2.10.1+
Query: lcl|KJ651141.1_prot_AJO61204.1_1 [protein=CenH3] [protein_id=AJO61204.1] [location=
Database: Nippon
Fields: query acc.ver, subject acc.ver, % identity, alignment length, mismatches, gap opens, q. s
19 hits found

lcl|KJ6511 Chr6	57.143	91	38	1	24	114	3040816
lcl|KJ6511 Chr6	57.143	91	38	1	24	114	3055493
lcl|KJ6511 Chr6	57.143	91	38	1	24	114	3046846
lcl|KJ6511 Chr6	57.143	91	38	1	24	114	3056300
lcl|KJ6511 Chr6	58.242	91	37	1	24	114	1645985
lcl|KJ6511 Chr11	57.143	91	38	1	24	114	2626091
lcl|KJ6511 Chr5	57.143	91	38	1	24	114	21499966
lcl|KJ6511 Chr5	79.31	29	6	0	24	52	24069136
lcl|KJ6511 Chr5	63.636	22	8	0	82	103	24068431
lcl|KJ6511 Chr4	57.143	91	38	1	24	114	20739890
lcl|KJ6511 Chr4	40.87	115	37	2	31	114	22463383
lcl|KJ6511 Chr1	57.143	91	38	1	24	114	37514026
lcl|KJ6511 Chr12	56.044	91	39	1	24	114	12808292
lcl|KJ6511 Chr12	52.747	91	42	1	24	114	12792736
lcl|KJ6511 Chr2	40.517	116	38	2	30	114	15225499
lcl|KJ6511 Chr2	54.545	33	15	0	82	114	15204218
lcl|KJ6511 Chr2	55.882	34	13	1	30	61	15204468
lcl|KJ6511 Chr3	60.606	33	13	0	82	114	15647705
lcl|KJ6511 Chr3	64	25	9	0	31	55	15647458

TBLASTN 2.10.1+
Query: lcl|KJ651140.1_prot_AJO61203.1_1 [protein=CenH3] [protein_id=AJO61203.1] [location=
Database: Nippon
Fields: query acc.ver, subject acc.ver, % identity, alignment length, mismatches, gap opens, q. s
23 hits found

lcl|KJ6511	Chr6	56.044	91	39	1	24	114	1645985
lcl|KJ6511	Chr6	54.945	91	40	1	24	114	3040816
lcl|KJ6511	Chr6	54.945	91	40	1	24	114	3055493
lcl|KJ6511	Chr6	54.945	91	40	1	24	114	3056300
lcl|KJ6511	Chr6	54.945	91	40	1	24	114	3046846
lcl|KJ6511	Chr6	52.632	19	9	0	32	50	16886026
lcl|KJ6511	Chr11	54.945	91	40	1	24	114	2626091
lcl|KJ6511	Chr5	54.945	91	40	1	24	114	21499966
lcl|KJ6511	Chr5	82.759	29	5	0	24	52	24069136
lcl|KJ6511	Chr5	56.667	30	13	0	52	81	24068958
lcl|KJ6511	Chr5	48.485	33	17	0	71	103	24068464
lcl|KJ6511	Chr4	54.945	91	40	1	24	114	20739890
lcl|KJ6511	Chr4	37.391	115	41	1	31	114	22463383
lcl|KJ6511	Chr1	57.143	91	38	1	24	114	37514026
lcl|KJ6511	Chr1	38.462	39	19	1	42	75	30558714
lcl|KJ6511	Chr12	54.945	91	40	1	24	114	12808292
lcl|KJ6511	Chr12	52.747	91	42	1	24	114	12792736
lcl|KJ6511	Chr2	37.931	116	41	1	30	114	15225499
lcl|KJ6511	Chr2	43.284	67	33	1	48	114	15204305
lcl|KJ6511	Chr2	54.545	33	15	0	30	62	15204468
lcl|KJ6511	Chr3	50.82	61	30	0	54	114	15647621
lcl|KJ6511	Chr3	45.714	35	19	0	31	65	15647458
lcl|KJ6511	Chr10	37.778	45	27	1	32	75	1887345

TBLASTN 2.10.1+
Query: lcl|KJ651136.1_prot_AJO61199.1_1 [protein=CenH3] [protein_id=AJO61199.1] [location=
Database: Nippon
Fields: query acc.ver, subject acc.ver, % identity, alignment length, mismatches, gap opens, q. s
22 hits found

lcl|KJ6511	Chr6	57.143	91	38	1	24	114	1645985
lcl|KJ6511	Chr6	56.044	91	39	1	24	114	3040816
lcl|KJ6511	Chr6	56.044	91	39	1	24	114	3055493
lcl|KJ6511	Chr6	56.044	91	39	1	24	114	3056300
lcl|KJ6511	Chr6	56.044	91	39	1	24	114	3046846
lcl|KJ6511	Chr11	56.044	91	39	1	24	114	2626091
lcl|KJ6511	Chr11	38.235	34	21	0	58	91	27980107
lcl|KJ6511	Chr5	56.044	91	39	1	24	114	21499966
lcl|KJ6511	Chr5	79.31	29	6	0	24	52	24069136
lcl|KJ6511	Chr5	52.778	36	16	1	52	86	24068958
lcl|KJ6511	Chr5	54.545	33	15	0	71	103	24068464
lcl|KJ6511	Chr4	56.044	91	39	1	24	114	20739890
lcl|KJ6511	Chr4	40	115	38	2	31	114	22463383
lcl|KJ6511	Chr1	56.044	91	39	1	24	114	37514026
lcl|KJ6511	Chr1	31.034	58	31	2	42	94	30558714
lcl|KJ6511	Chr12	54.945	91	40	1	24	114	12808292
lcl|KJ6511	Chr12	52.747	91	42	1	24	114	12792736
lcl|KJ6511	Chr2	39.655	116	39	2	30	114	15225499
lcl|KJ6511	Chr2	44.776	67	32	1	48	114	15204305
lcl|KJ6511	Chr2	54.839	31	14	0	30	60	15204468

lcl|KJ6511	Chr3	58	50	21	0	65	114	15647654
lcl|KJ6511	Chr3	35.938	64	38	2	31	94	15647458

TBLASTN 2.10.1+
Query: lcl|KJ651133.1_prot_AJO61196.1_1 [protein=CenH3] [protein_id=AJO61196.1] [location=
Database: Nippon
Fields: query acc.ver, subject acc.ver, % identity, alignment length, mismatches, gap opens, q. s
22 hits found

lcl|KJ6511	Chr6	56.044	91	39	1	24	114	1645985
lcl|KJ6511	Chr6	54.945	91	40	1	24	114	3040816
lcl|KJ6511	Chr6	54.945	91	40	1	24	114	3055493
lcl|KJ6511	Chr6	54.945	91	40	1	24	114	3056300
lcl|KJ6511	Chr6	54.945	91	40	1	24	114	3046846
lcl|KJ6511	Chr11	54.945	91	40	1	24	114	2626091
lcl|KJ6511	Chr5	54.945	91	40	1	24	114	21499966
lcl|KJ6511	Chr5	79.31	29	6	0	24	52	24069136
lcl|KJ6511	Chr5	56.667	30	13	0	52	81	24068958
lcl|KJ6511	Chr5	51.515	33	16	0	71	103	24068464
lcl|KJ6511	Chr4	54.945	91	40	1	24	114	20739890
lcl|KJ6511	Chr4	37.391	115	41	1	31	114	22463383
lcl|KJ6511	Chr1	57.143	91	38	1	24	114	37514026
lcl|KJ6511	Chr1	38.462	39	19	1	42	75	30558714
lcl|KJ6511	Chr12	54.945	91	40	1	24	114	12808292
lcl|KJ6511	Chr12	52.747	91	42	1	24	114	12792736
lcl|KJ6511	Chr2	37.931	116	41	1	30	114	15225499
lcl|KJ6511	Chr2	43.284	67	33	1	48	114	15204305
lcl|KJ6511	Chr2	54.545	33	15	0	30	62	15204468
lcl|KJ6511	Chr3	50.82	61	30	0	54	114	15647621
lcl|KJ6511	Chr3	45.714	35	19	0	31	65	15647458
lcl|KJ6511	Chr10	40	45	26	1	32	75	1887345

TBLASTN 2.10.1+
Query: lcl|KJ651128.1_prot_AJO61191.1_1 [protein=CenH3] [protein_id=AJO61191.1] [location=
Database: Nippon
Fields: query acc.ver, subject acc.ver, % identity, alignment length, mismatches, gap opens, q. s
23 hits found

lcl|KJ6511	Chr6	47.664	107	55	1	12	118	1646033
lcl|KJ6511	Chr6	51.087	92	44	1	27	118	3040819
lcl|KJ6511	Chr6	47.664	107	55	1	12	118	3056252
lcl|KJ6511	Chr6	47.664	107	55	1	12	118	3055541
lcl|KJ6511	Chr6	47.664	107	55	1	12	118	3046894
lcl|KJ6511	Chr5	47.664	107	55	1	12	118	21500014
lcl|KJ6511	Chr5	70	30	9	0	27	56	24069139
lcl|KJ6511	Chr5	55.556	27	12	0	56	82	24068958
lcl|KJ6511	Chr5	50	30	15	0	78	107	24068455
lcl|KJ6511	Chr11	51.087	92	44	1	27	118	2626094
lcl|KJ6511	Chr4	47.664	107	55	1	12	118	20739842
lcl|KJ6511	Chr4	36.522	115	42	2	35	118	22463383
lcl|KJ6511	Chr1	50	98	48	1	21	118	37514005
lcl|KJ6511	Chr12	50	92	45	1	27	118	12808295

lcl|KJ6511	Chr12	48.352	91	46	1	28	118	12792736
lcl|KJ6511	Chr2	34.483	116	45	1	34	118	15225499
lcl|KJ6511	Chr2	40.299	67	35	1	52	118	15204305
lcl|KJ6511	Chr2	53.333	30	14	0	34	63	15204468
lcl|KJ6511	Chr3	52	50	24	0	69	118	15647654
lcl|KJ6511	Chr3	51.724	29	14	0	35	63	15647458
lcl|KJ6511	Chr3	29.31	58	34	1	16	66	21409383
lcl|KJ6511	Chr3	30.769	117	52	6	1	91	19946460
lcl|KJ6511	Chr9	46.154	39	19	1	37	75	14583509

TBLASTN 2.10.1+
Query: lcl|KJ651123.1_prot_AJO61186.1_1 [protein=CenH3] [protein_id=AJO61186.1] [location=
Database: Nippon
Fields: query acc.ver, subject acc.ver, % identity, alignment length, mismatches, gap opens, q. s
20 hits found

lcl|KJ6511	Chr6	47.664	107	55	1	12	118	1646033
lcl|KJ6511	Chr6	47.664	107	55	1	12	118	3040864
lcl|KJ6511	Chr6	47.664	107	55	1	12	118	3056252
lcl|KJ6511	Chr6	47.664	107	55	1	12	118	3055541
lcl|KJ6511	Chr6	47.664	107	55	1	12	118	3046894
lcl|KJ6511	Chr5	47.664	107	55	1	12	118	21500014
lcl|KJ6511	Chr5	34.066	91	48	3	14	94	24069187
lcl|KJ6511	Chr5	55.556	27	12	0	56	82	24068958
lcl|KJ6511	Chr5	50	30	15	0	78	107	24068455
lcl|KJ6511	Chr11	47.664	107	55	1	12	118	2626139
lcl|KJ6511	Chr4	47.664	107	55	1	12	118	20739842
lcl|KJ6511	Chr4	35.652	115	43	1	35	118	22463383
lcl|KJ6511	Chr1	50	98	48	1	21	118	37514005
lcl|KJ6511	Chr12	48.98	98	49	1	21	118	12808313
lcl|KJ6511	Chr12	46.939	98	51	1	21	118	12792757
lcl|KJ6511	Chr2	36.207	116	43	2	34	118	15225499
lcl|KJ6511	Chr2	40.299	67	35	1	52	118	15204305
lcl|KJ6511	Chr2	56.667	30	13	0	34	63	15204468
lcl|KJ6511	Chr3	52	50	24	0	69	118	15647654
lcl|KJ6511	Chr3	45.714	35	19	0	35	69	15647458

TBLASTN 2.10.1+
Query: lcl|KJ651119.1_prot_AJO61182.1_1 [protein=CenH3] [protein_id=AJO61182.1] [location=
Database: Nippon
Fields: query acc.ver, subject acc.ver, % identity, alignment length, mismatches, gap opens, q. s
22 hits found

lcl|KJ6511	Chr6	50.467	107	52	1	12	118	1646033
lcl|KJ6511	Chr6	50.467	107	52	1	12	118	3056252
lcl|KJ6511	Chr6	50.467	107	52	1	12	118	3040864
lcl|KJ6511	Chr6	50.467	107	52	1	12	118	3055541
lcl|KJ6511	Chr6	50.467	107	52	1	12	118	3046894
lcl|KJ6511	Chr5	50.467	107	52	1	12	118	21500014
lcl|KJ6511	Chr5	66.667	36	12	0	21	56	24069157
lcl|KJ6511	Chr5	51.852	27	13	0	56	82	24068958
lcl|KJ6511	Chr5	50	30	15	0	78	107	24068455

lcl|KJ6511	Chr11	50.467	107	52	1	12	118	2626139
lcl|KJ6511	Chr4	50.467	107	52	1	12	118	20739842
lcl|KJ6511	Chr4	33.333	168	51	3	12	118	22463224
lcl|KJ6511	Chr1	50.467	107	52	1	12	118	37513978
lcl|KJ6511	Chr12	48.598	107	54	1	12	118	12808340
lcl|KJ6511	Chr12	46.729	107	56	1	12	118	12792784
lcl|KJ6511	Chr3	52	50	24	0	69	118	15647654
lcl|KJ6511	Chr3	48.571	35	18	0	35	69	15647458
lcl|KJ6511	Chr3	52.174	23	11	0	12	34	15647298
lcl|KJ6511	Chr3	29.31	58	34	1	16	66	21409383
lcl|KJ6511	Chr2	37.069	116	42	2	34	118	15225499
lcl|KJ6511	Chr2	40.299	67	35	1	52	118	15204305
lcl|KJ6511	Chr2	56.667	30	13	0	34	63	15204468

TBLASTN 2.10.1+
Query: lcl|KJ651117.1_prot_AJO61180.1_1 [protein=CenH3] [protein_id=AJO61180.1] [location=
Database: Nippon
Fields: query acc.ver, subject acc.ver, % identity, alignment length, mismatches, gap opens, q. s
22 hits found

lcl|KJ6511	Chr6	49.533	107	53	1	12	118	1646033
lcl|KJ6511	Chr6	49.533	107	53	1	12	118	3056252
lcl|KJ6511	Chr6	49.533	107	53	1	12	118	3040864
lcl|KJ6511	Chr6	49.533	107	53	1	12	118	3055541
lcl|KJ6511	Chr6	49.074	108	54	1	11	118	3046897
lcl|KJ6511	Chr5	49.533	107	53	1	12	118	21500014
lcl|KJ6511	Chr5	54.167	48	15	1	21	61	24069157
lcl|KJ6511	Chr5	55.556	27	12	0	56	82	24068958
lcl|KJ6511	Chr5	46.667	30	16	0	78	107	24068455
lcl|KJ6511	Chr4	49.533	107	53	1	12	118	20739842
lcl|KJ6511	Chr4	32.143	168	53	3	12	118	22463224
lcl|KJ6511	Chr11	49.533	107	53	1	12	118	2626139
lcl|KJ6511	Chr1	49.533	107	53	1	12	118	37513978
lcl|KJ6511	Chr12	48.598	107	54	1	12	118	12808340
lcl|KJ6511	Chr12	46.729	107	56	1	12	118	12792784
lcl|KJ6511	Chr3	50	50	25	0	69	118	15647654
lcl|KJ6511	Chr3	55.172	29	13	0	35	63	15647458
lcl|KJ6511	Chr3	52.174	23	11	0	12	34	15647298
lcl|KJ6511	Chr3	29.31	58	34	1	16	66	21409383
lcl|KJ6511	Chr2	34.483	116	45	1	34	118	15225499
lcl|KJ6511	Chr2	38.806	67	36	1	52	118	15204305
lcl|KJ6511	Chr2	56.667	30	13	0	34	63	15204468

TBLASTN 2.10.1+
Query: lcl|KJ651116.1_prot_AJO61179.1_1 [protein=CenH3] [protein_id=AJO61179.1] [location=
Database: Nippon
Fields: query acc.ver, subject acc.ver, % identity, alignment length, mismatches, gap opens, q. s
22 hits found

lcl|KJ6511	Chr6	49.533	107	53	1	12	118	3040864
lcl|KJ6511	Chr6	49.533	107	53	1	12	118	3056252
lcl|KJ6511	Chr6	49.533	107	53	1	12	118	3055541

lcl|KJ6511 Chr6	49.533	107	53	1	12	118	1646033
lcl|KJ6511 Chr6	49.533	107	53	1	12	118	3046894
lcl|KJ6511 Chr5	49.533	107	53	1	12	118	21500014
lcl|KJ6511 Chr5	36.047	86	46	2	16	94	24069172
lcl|KJ6511 Chr5	59.259	27	11	0	56	82	24068958
lcl|KJ6511 Chr5	50	30	15	0	78	107	24068455
lcl|KJ6511 Chr11	49.533	107	53	1	12	118	2626139
lcl|KJ6511 Chr4	49.533	107	53	1	12	118	20739842
lcl|KJ6511 Chr4	32.143	168	53	3	12	118	22463224
lcl|KJ6511 Chr1	49.533	107	53	1	12	118	37513978
lcl|KJ6511 Chr12	48.598	107	54	1	12	118	12808340
lcl|KJ6511 Chr12	47.959	98	50	1	21	118	12792757
lcl|KJ6511 Chr2	36.207	116	43	1	34	118	15225499
lcl|KJ6511 Chr2	40.299	67	35	1	52	118	15204305
lcl|KJ6511 Chr2	60	30	12	0	34	63	15204468
lcl|KJ6511 Chr3	52	50	24	0	69	118	15647654
lcl|KJ6511 Chr3	58.621	29	12	0	35	63	15647458
lcl|KJ6511 Chr3	27.869	61	41	2	18	77	9249068
lcl|KJ6511 Chr3	29.06	117	54	5	1	91	19946460

TBLASTN 2.10.1+
Query: lcl|KJ651112.1_prot_AJO61175.1_1 [protein=CenH3] [protein_id=AJO61175.1] [location=
Database: Nippon
Fields: query acc.ver, subject acc.ver, % identity, alignment length, mismatches, gap opens, q. s
23 hits found

lcl|KJ6511 Chr6	55.102	98	43	1	17	114	1646006
lcl|KJ6511 Chr6	55.102	98	43	1	17	114	3056279
lcl|KJ6511 Chr6	55.102	98	43	1	17	114	3040837
lcl|KJ6511 Chr6	55.102	98	43	1	17	114	3055514
lcl|KJ6511 Chr6	55.102	98	43	1	17	114	3046867
lcl|KJ6511 Chr11	55.102	98	43	1	17	114	2626112
lcl|KJ6511 Chr5	55.102	98	43	1	17	114	21499987
lcl|KJ6511 Chr5	65.116	43	15	0	10	52	24069178
lcl|KJ6511 Chr5	50	36	17	1	52	86	24068958
lcl|KJ6511 Chr5	60	30	12	0	74	103	24068455
lcl|KJ6511 Chr4	55.102	98	43	1	17	114	20739869
lcl|KJ6511 Chr4	33.333	159	45	2	17	114	22463251
lcl|KJ6511 Chr1	55.102	98	43	1	17	114	37514005
lcl|KJ6511 Chr1	26.923	78	48	2	42	114	30558714
lcl|KJ6511 Chr12	55.102	98	43	1	17	114	12808313
lcl|KJ6511 Chr12	52.041	98	46	1	17	114	12792757
lcl|KJ6511 Chr12	23.81	105	76	3	16	118	9050848
lcl|KJ6511 Chr3	58	50	21	0	65	114	15647654
lcl|KJ6511 Chr3	45.714	35	19	0	31	65	15647458
lcl|KJ6511 Chr3	64.286	14	5	0	17	30	15647325
lcl|KJ6511 Chr2	39.655	116	39	2	30	114	15225499
lcl|KJ6511 Chr2	44.776	67	32	1	48	114	15204305
lcl|KJ6511 Chr2	51.515	33	16	0	30	62	15204468

TBLASTN 2.10.1+

Query: lcl|KJ651108.1_prot_AJO61171.1_1 [protein=CenH3] [protein_id=AJO61171.1] [location=
Database: Nippon
Fields: query acc.ver, subject acc.ver, % identity, alignment length, mismatches, gap opens, q. s
22 hits found

lcl|KJ6511	Chr6	56.044	91	39	1	24	114	1645985
lcl|KJ6511	Chr6	54.945	91	40	1	24	114	3040816
lcl|KJ6511	Chr6	54.945	91	40	1	24	114	3055493
lcl|KJ6511	Chr6	54.945	91	40	1	24	114	3056300
lcl|KJ6511	Chr6	54.945	91	40	1	24	114	3046846
lcl|KJ6511	Chr11	54.945	91	40	1	24	114	2626091
lcl|KJ6511	Chr11	38.235	34	21	0	58	91	27980107
lcl|KJ6511	Chr5	54.945	91	40	1	24	114	21499966
lcl|KJ6511	Chr5	75.862	29	7	0	24	52	24069136
lcl|KJ6511	Chr5	51.351	37	17	1	52	87	24068958
lcl|KJ6511	Chr5	51.515	33	16	0	71	103	24068464
lcl|KJ6511	Chr12	54.945	91	40	1	24	114	12808292
lcl|KJ6511	Chr12	52.747	91	42	1	24	114	12792736
lcl|KJ6511	Chr4	54.945	91	40	1	24	114	20739890
lcl|KJ6511	Chr4	39.13	115	39	2	31	114	22463383
lcl|KJ6511	Chr1	54.945	91	40	1	24	114	37514026
lcl|KJ6511	Chr1	26.923	78	48	2	42	114	30558714
lcl|KJ6511	Chr2	38.793	116	40	2	30	114	15225499
lcl|KJ6511	Chr2	44.776	67	32	1	48	114	15204305
lcl|KJ6511	Chr2	55.172	29	13	0	30	58	15204468
lcl|KJ6511	Chr3	58	50	21	0	65	114	15647654
lcl|KJ6511	Chr3	35.484	62	37	2	31	92	15647458

TBLASTN 2.10.1+
Query: lcl|KJ651107.1_prot_AJO61170.1_1 [protein=CenH3] [protein_id=AJO61170.1] [location=
Database: Nippon
Fields: query acc.ver, subject acc.ver, % identity, alignment length, mismatches, gap opens, q. s
21 hits found

lcl|KJ6511	Chr12	52.041	98	46	1	21	118	12808313
lcl|KJ6511	Chr12	50	98	48	1	21	118	12792757
lcl|KJ6511	Chr5	52.041	98	46	1	21	118	21499987
lcl|KJ6511	Chr5	66.667	36	12	0	21	56	24069157
lcl|KJ6511	Chr5	51.852	27	13	0	56	82	24068958
lcl|KJ6511	Chr5	53.333	30	14	0	78	107	24068455
lcl|KJ6511	Chr6	52.041	98	46	1	21	118	3056279
lcl|KJ6511	Chr6	52.041	98	46	1	21	118	3040837
lcl|KJ6511	Chr6	49.533	107	53	1	12	118	1646033
lcl|KJ6511	Chr6	52.041	98	46	1	21	118	3055514
lcl|KJ6511	Chr6	52.041	98	46	1	21	118	3046867
lcl|KJ6511	Chr11	52.041	98	46	1	21	118	2626112
lcl|KJ6511	Chr4	52.041	98	46	1	21	118	20739869
lcl|KJ6511	Chr4	32.143	168	53	3	12	118	22463224
lcl|KJ6511	Chr1	52.041	98	46	1	21	118	37514005
lcl|KJ6511	Chr2	37.069	116	42	2	34	118	15225499
lcl|KJ6511	Chr2	43.284	67	33	1	52	118	15204305

<table>
<tr><td>lcl|KJ6511</td><td>Chr2</td><td>53.333</td><td>30</td><td>14</td><td>0</td><td>34</td><td>63</td><td>15204468</td></tr>
<tr><td>lcl|KJ6511</td><td>Chr3</td><td>54</td><td>50</td><td>23</td><td>0</td><td>69</td><td>118</td><td>15647654</td></tr>
<tr><td>lcl|KJ6511</td><td>Chr3</td><td>42.857</td><td>35</td><td>20</td><td>0</td><td>35</td><td>69</td><td>15647458</td></tr>
<tr><td>lcl|KJ6511</td><td>Chr3</td><td>64.286</td><td>14</td><td>5</td><td>0</td><td>21</td><td>34</td><td>15647325</td></tr>
</table>

TBLASTN 2.10.1+
Query: lcl|KJ507244.1_prot_AHW98238.1_1 [gene=CenH3] [protein=centromeric histone 3] [pro
Database: Nippon
Fields: query acc.ver, subject acc.ver, % identity, alignment length, mismatches, gap opens, q. s
24 hits found

<table>
<tr><td>lcl|KJ5072</td><td>Chr12</td><td>53.608</td><td>97</td><td>44</td><td>1</td><td>22</td><td>118</td><td>12808310</td></tr>
<tr><td>lcl|KJ5072</td><td>Chr12</td><td>49.485</td><td>97</td><td>48</td><td>1</td><td>22</td><td>118</td><td>12792754</td></tr>
<tr><td>lcl|KJ5072</td><td>Chr11</td><td>53.191</td><td>94</td><td>43</td><td>1</td><td>25</td><td>118</td><td>2626100</td></tr>
<tr><td>lcl|KJ5072</td><td>Chr6</td><td>52.577</td><td>97</td><td>45</td><td>1</td><td>22</td><td>118</td><td>3040834</td></tr>
<tr><td>lcl|KJ5072</td><td>Chr6</td><td>52.577</td><td>97</td><td>45</td><td>1</td><td>22</td><td>118</td><td>1646003</td></tr>
<tr><td>lcl|KJ5072</td><td>Chr6</td><td>53.191</td><td>94</td><td>43</td><td>1</td><td>25</td><td>118</td><td>3055502</td></tr>
<tr><td>lcl|KJ5072</td><td>Chr6</td><td>49.074</td><td>108</td><td>54</td><td>1</td><td>11</td><td>118</td><td>3056249</td></tr>
<tr><td>lcl|KJ5072</td><td>Chr6</td><td>52.577</td><td>97</td><td>45</td><td>1</td><td>22</td><td>118</td><td>3046864</td></tr>
<tr><td>lcl|KJ5072</td><td>Chr5</td><td>49.074</td><td>108</td><td>54</td><td>1</td><td>11</td><td>118</td><td>21500017</td></tr>
<tr><td>lcl|KJ5072</td><td>Chr5</td><td>60</td><td>40</td><td>16</td><td>0</td><td>17</td><td>56</td><td>24069169</td></tr>
<tr><td>lcl|KJ5072</td><td>Chr5</td><td>51.852</td><td>27</td><td>13</td><td>0</td><td>56</td><td>82</td><td>24068958</td></tr>
<tr><td>lcl|KJ5072</td><td>Chr5</td><td>51.515</td><td>33</td><td>16</td><td>0</td><td>75</td><td>107</td><td>24068464</td></tr>
<tr><td>lcl|KJ5072</td><td>Chr4</td><td>52.577</td><td>97</td><td>45</td><td>1</td><td>22</td><td>118</td><td>20739872</td></tr>
<tr><td>lcl|KJ5072</td><td>Chr4</td><td>32.911</td><td>158</td><td>45</td><td>3</td><td>22</td><td>118</td><td>22463254</td></tr>
<tr><td>lcl|KJ5072</td><td>Chr4</td><td>24.793</td><td>121</td><td>66</td><td>5</td><td>20</td><td>119</td><td>1121700</td></tr>
<tr><td>lcl|KJ5072</td><td>Chr1</td><td>52.577</td><td>97</td><td>45</td><td>1</td><td>22</td><td>118</td><td>37514008</td></tr>
<tr><td>lcl|KJ5072</td><td>Chr2</td><td>36.207</td><td>116</td><td>43</td><td>1</td><td>34</td><td>118</td><td>15225499</td></tr>
<tr><td>lcl|KJ5072</td><td>Chr2</td><td>47.761</td><td>67</td><td>30</td><td>1</td><td>52</td><td>118</td><td>15204305</td></tr>
<tr><td>lcl|KJ5072</td><td>Chr2</td><td>50</td><td>26</td><td>13</td><td>0</td><td>34</td><td>59</td><td>15204468</td></tr>
<tr><td>lcl|KJ5072</td><td>Chr2</td><td>30</td><td>60</td><td>42</td><td>0</td><td>12</td><td>71</td><td>28327638</td></tr>
<tr><td>lcl|KJ5072</td><td>Chr3</td><td>60</td><td>50</td><td>20</td><td>0</td><td>69</td><td>118</td><td>15647654</td></tr>
<tr><td>lcl|KJ5072</td><td>Chr3</td><td>37.143</td><td>35</td><td>22</td><td>0</td><td>35</td><td>69</td><td>15647458</td></tr>
<tr><td>lcl|KJ5072</td><td>Chr3</td><td>45.833</td><td>24</td><td>13</td><td>0</td><td>11</td><td>34</td><td>15647295</td></tr>
<tr><td>lcl|KJ5072</td><td>Chr8</td><td>50</td><td>28</td><td>14</td><td>0</td><td>20</td><td>47</td><td>25284192</td></tr>
</table>

TBLASTN 2.10.1+
Query: lcl|KJ507242.1_prot_AHW98236.1_1 [gene=CenH3] [protein=centromeric histone 3] [pro
Database: Nippon
Fields: query acc.ver, subject acc.ver, % identity, alignment length, mismatches, gap opens, q. s
24 hits found

<table>
<tr><td>lcl|KJ5072</td><td>Chr12</td><td>54.639</td><td>97</td><td>43</td><td>1</td><td>22</td><td>118</td><td>12808310</td></tr>
<tr><td>lcl|KJ5072</td><td>Chr12</td><td>50.515</td><td>97</td><td>47</td><td>1</td><td>22</td><td>118</td><td>12792754</td></tr>
<tr><td>lcl|KJ5072</td><td>Chr11</td><td>54.255</td><td>94</td><td>42</td><td>1</td><td>25</td><td>118</td><td>2626100</td></tr>
<tr><td>lcl|KJ5072</td><td>Chr11</td><td>42.857</td><td>42</td><td>23</td><td>1</td><td>8</td><td>48</td><td>4447208</td></tr>
<tr><td>lcl|KJ5072</td><td>Chr6</td><td>53.608</td><td>97</td><td>44</td><td>1</td><td>22</td><td>118</td><td>1646003</td></tr>
<tr><td>lcl|KJ5072</td><td>Chr6</td><td>53.608</td><td>97</td><td>44</td><td>1</td><td>22</td><td>118</td><td>3040834</td></tr>
<tr><td>lcl|KJ5072</td><td>Chr6</td><td>54.255</td><td>94</td><td>42</td><td>1</td><td>25</td><td>118</td><td>3055502</td></tr>
<tr><td>lcl|KJ5072</td><td>Chr6</td><td>53.608</td><td>97</td><td>44</td><td>1</td><td>22</td><td>118</td><td>3056282</td></tr>
<tr><td>lcl|KJ5072</td><td>Chr6</td><td>53.608</td><td>97</td><td>44</td><td>1</td><td>22</td><td>118</td><td>3046864</td></tr>
<tr><td>lcl|KJ5072</td><td>Chr6</td><td>52</td><td>25</td><td>12</td><td>0</td><td>5</td><td>29</td><td>6305245</td></tr>
</table>

lcl|KJ5072	Chr5	53.608	97	44	1	22	118	21499984
lcl|KJ5072	Chr5	58.537	41	17	0	16	56	24069172
lcl|KJ5072	Chr5	51.852	27	13	0	56	82	24068958
lcl|KJ5072	Chr5	54.545	33	15	0	75	107	24068464
lcl|KJ5072	Chr4	53.608	97	44	1	22	118	20739872
lcl|KJ5072	Chr4	33.544	158	44	3	22	118	22463254
lcl|KJ5072	Chr4	24.793	121	66	5	20	119	1121700
lcl|KJ5072	Chr1	53.608	97	44	1	22	118	37514008
lcl|KJ5072	Chr2	37.069	116	42	1	34	118	15225499
lcl|KJ5072	Chr2	49.254	67	29	1	52	118	15204305
lcl|KJ5072	Chr2	50	26	13	0	34	59	15204468
lcl|KJ5072	Chr3	62	50	19	0	69	118	15647654
lcl|KJ5072	Chr3	37.143	35	22	0	35	69	15647458
lcl|KJ5072	Chr3	69.231	13	4	0	22	34	15647328

TBLASTN 2.10.1+
Query: lcl|KJ507235.1_prot_AHW98229.1_1 [gene=CenH3] [protein=centromeric histone 3] [pro
Database: Nippon
Fields: query acc.ver, subject acc.ver, % identity, alignment length, mismatches, gap opens, q. s
21 hits found

lcl|KJ5072	Chr6	60.748	107	40	2	47	152	3040864
lcl|KJ5072	Chr6	60.748	107	40	2	47	152	3056252
lcl|KJ5072	Chr6	60.748	107	40	2	47	152	3055541
lcl|KJ5072	Chr6	60.748	107	40	2	47	152	3046894
lcl|KJ5072	Chr6	61.682	107	39	2	47	152	1646033
lcl|KJ5072	Chr5	60.748	107	40	2	47	152	21500014
lcl|KJ5072	Chr5	60	40	16	0	51	90	24069169
lcl|KJ5072	Chr5	68.75	16	5	0	90	105	24068958
lcl|KJ5072	Chr5	66.667	21	7	0	121	141	24068428
lcl|KJ5072	Chr11	60.748	107	40	2	47	152	2626139
lcl|KJ5072	Chr1	60.748	107	40	2	47	152	37513978
lcl|KJ5072	Chr4	62.245	98	36	1	55	152	20739869
lcl|KJ5072	Chr4	38.69	168	41	3	47	152	22463224
lcl|KJ5072	Chr12	61.224	98	37	1	55	152	12808313
lcl|KJ5072	Chr12	58.163	98	40	1	55	152	12792757
lcl|KJ5072	Chr2	43.966	116	34	1	68	152	15225499
lcl|KJ5072	Chr2	58.209	67	23	1	86	152	15204305
lcl|KJ5072	Chr2	50	32	16	0	68	99	15204468
lcl|KJ5072	Chr3	75	32	8	0	121	152	15647708
lcl|KJ5072	Chr3	48.571	35	18	0	69	103	15647458
lcl|KJ5072	Chr3	44.444	27	14	1	47	72	15647298

TBLASTN 2.10.1+
Query: lcl|KJ507234.1_prot_AHW98228.1_1 [gene=CenH3] [protein=centromeric histone 3] [pro
Database: Nippon
Fields: query acc.ver, subject acc.ver, % identity, alignment length, mismatches, gap opens, q. s
20 hits found

lcl|KJ5072	Chr6	59.813	107	41	2	47	152	3040864
lcl|KJ5072	Chr6	59.813	107	41	2	47	152	3056252
lcl|KJ5072	Chr6	59.813	107	41	2	47	152	3055541

lcl|KJ5072	Chr6	59.813	107	41	2	47	152	3046894
lcl|KJ5072	Chr6	60.748	107	40	2	47	152	1646033
lcl|KJ5072	Chr5	59.813	107	41	2	47	152	21500014
lcl|KJ5072	Chr5	64.103	39	14	0	52	90	24069166
lcl|KJ5072	Chr5	56.25	16	7	0	90	105	24068958
lcl|KJ5072	Chr5	72.222	18	5	0	124	141	24068419
lcl|KJ5072	Chr11	59.813	107	41	2	47	152	2626139
lcl|KJ5072	Chr1	59.813	107	41	2	47	152	37513978
lcl|KJ5072	Chr4	59.813	107	41	2	47	152	20739842
lcl|KJ5072	Chr4	38.69	168	41	3	47	152	22463224
lcl|KJ5072	Chr12	59.184	98	39	1	55	152	12808313
lcl|KJ5072	Chr12	56.122	98	42	1	55	152	12792757
lcl|KJ5072	Chr2	43.103	116	35	1	68	152	15225499
lcl|KJ5072	Chr2	56.716	67	24	1	86	152	15204305
lcl|KJ5072	Chr2	50	32	16	0	68	99	15204468
lcl|KJ5072	Chr3	75.862	29	7	0	124	152	15647717
lcl|KJ5072	Chr3	48.571	35	18	0	69	103	15647458

TBLASTN 2.10.1+
Query: lcl|HM582916.1_prot_ADN92693.1_1 [gene=CENH3] [protein=centromere-specific H3 va
Database: Nippon
Fields: query acc.ver, subject acc.ver, % identity, alignment length, mismatches, gap opens, q. s
22 hits found

lcl|HM582	Chr6	53.211	109	49	1	71	179	1646033
lcl|HM582	Chr6	53.211	109	49	2	71	179	3040864
lcl|HM582	Chr6	52.294	109	50	1	71	179	3056252
lcl|HM582	Chr6	53.211	109	49	2	71	179	3055541
lcl|HM582	Chr6	52.294	109	50	1	71	179	3046894
lcl|HM582	Chr1	52.778	108	49	1	71	178	37513978
lcl|HM582	Chr5	53.211	109	49	2	71	179	21500014
lcl|HM582	Chr5	60.976	41	16	0	75	115	24069172
lcl|HM582	Chr5	51.724	29	13	1	114	142	24068961
lcl|HM582	Chr5	51.613	31	15	0	140	170	24068449
lcl|HM582	Chr11	53.211	109	49	2	71	179	2626139
lcl|HM582	Chr4	52.294	109	50	1	71	179	20739842
lcl|HM582	Chr4	35.329	167	46	3	74	179	22463233
lcl|HM582	Chr12	50.893	112	47	2	71	179	12808340
lcl|HM582	Chr12	48.624	109	48	2	74	179	12792775
lcl|HM582	Chr3	60.784	51	20	0	129	179	15647654
lcl|HM582	Chr3	51.724	29	14	0	94	122	15647458
lcl|HM582	Chr3	44.444	27	15	0	71	97	15647298
lcl|HM582	Chr2	39.831	118	39	2	93	179	15225499
lcl|HM582	Chr2	58.824	51	21	0	129	179	15204269
lcl|HM582	Chr2	53.333	30	14	0	93	122	15204468
lcl|HM582	Chr2	36.842	38	24	0	125	162	24733423

TBLASTN 2.10.1+
Query: lcl|MN625524.1_prot_QGY64363.1_1 [gene=CENH3] [protein=histone H3-like centromei
Database: Nippon
Fields: query acc.ver, subject acc.ver, % identity, alignment length, mismatches, gap opens, q. s

22 hits found

lcl|MN625	Chr11	60.909	110	40	2	45	154	2626139
lcl|MN625	Chr6	60.909	110	40	2	45	154	3040864
lcl|MN625	Chr6	60.909	110	40	2	45	154	3055541
lcl|MN625	Chr6	59.821	112	42	2	43	154	3046900
lcl|MN625	Chr6	60.909	110	40	2	45	154	3056252
lcl|MN625	Chr6	60	110	41	2	45	154	1646033
lcl|MN625	Chr5	61.468	109	39	2	46	154	21500011
lcl|MN625	Chr5	74.419	43	11	0	49	91	24069178
lcl|MN625	Chr5	57.692	26	11	0	120	145	24068434
lcl|MN625	Chr1	62.037	108	38	2	46	153	37513981
lcl|MN625	Chr4	60.909	110	40	2	45	154	20739842
lcl|MN625	Chr4	38.824	170	41	3	46	154	22463227
lcl|MN625	Chr12	61.818	110	39	2	45	154	12808340
lcl|MN625	Chr12	58.716	109	42	2	46	154	12792781
lcl|MN625	Chr2	43.697	119	32	2	69	154	15225499
lcl|MN625	Chr2	65.714	35	12	0	120	154	15204221
lcl|MN625	Chr2	56.667	30	13	0	69	98	15204468
lcl|MN625	Chr2	76.471	17	4	0	56	72	20134900
lcl|MN625	Chr2	50	32	14	1	40	71	15225811
lcl|MN625	Chr3	68.571	35	11	0	120	154	15647702
lcl|MN625	Chr3	48.571	35	18	0	70	104	15647458
lcl|MN625	Chr3	53.571	28	11	1	46	73	15647301

TBLASTN 2.10.1+
Query: lcl|MN625523.1_prot_QGY64362.1_1 [gene=CENH3] [protein=histone H3-like centrome
Database: Nippon
Fields: query acc.ver, subject acc.ver, % identity, alignment length, mismatches, gap opens, q. s
22 hits found

lcl|MN625	Chr11	61.818	110	39	2	45	154	2626139
lcl|MN625	Chr6	61.818	110	39	2	45	154	3040864
lcl|MN625	Chr6	61.818	110	39	2	45	154	3055541
lcl|MN625	Chr6	60.714	112	41	2	43	154	3046900
lcl|MN625	Chr6	61.818	110	39	2	45	154	3056252
lcl|MN625	Chr6	60.909	110	40	2	45	154	1646033
lcl|MN625	Chr5	61.818	110	39	2	45	154	21500014
lcl|MN625	Chr5	76.744	43	10	0	49	91	24069178
lcl|MN625	Chr5	57.692	26	11	0	120	145	24068434
lcl|MN625	Chr4	61.818	110	39	2	45	154	20739842
lcl|MN625	Chr4	39.412	170	40	3	46	154	22463227
lcl|MN625	Chr1	62.963	108	37	2	46	153	37513981
lcl|MN625	Chr12	62.727	110	38	2	45	154	12808340
lcl|MN625	Chr12	58.182	110	43	2	45	154	12792784
lcl|MN625	Chr2	43.697	119	32	2	69	154	15225499
lcl|MN625	Chr2	65.714	35	12	0	120	154	15204221
lcl|MN625	Chr2	56.667	30	13	0	69	98	15204468
lcl|MN625	Chr2	77.778	18	4	0	56	73	20134900
lcl|MN625	Chr2	53.125	32	13	1	40	71	15225811
lcl|MN625	Chr3	68.571	35	11	0	120	154	15647702

lcl|MN625	Chr3	48.571	35	18	0	70	104	15647458
lcl|MN625	Chr3	61.538	26	8	1	46	71	15647301

TBLASTN 2.10.1+
Query: lcl|MN625521.1_prot_QGY64360.1_1 [gene=CENH3] [protein=histone H3-like centrome
Database: Nippon
Fields: query acc.ver, subject acc.ver, % identity, alignment length, mismatches, gap opens, q. s
20 hits found

lcl|MN625	Chr12	57.547	106	44	1	40	145	12808334
lcl|MN625	Chr12	53.774	106	48	1	40	145	12792778
lcl|MN625	Chr11	48.276	145	64	3	1	145	2626220
lcl|MN625	Chr6	48.276	145	64	3	1	145	3055622
lcl|MN625	Chr6	48.276	145	64	3	1	145	3040945
lcl|MN625	Chr6	56.604	106	45	1	40	145	3046888
lcl|MN625	Chr6	56.604	106	45	1	40	145	1646027
lcl|MN625	Chr6	48.276	145	64	3	1	145	3056171
lcl|MN625	Chr5	48.276	145	64	3	1	145	21500095
lcl|MN625	Chr5	74.286	35	9	0	48	82	24069154
lcl|MN625	Chr4	48.276	145	64	3	1	145	20739761
lcl|MN625	Chr4	37.126	167	44	3	40	145	22463230
lcl|MN625	Chr1	48.611	144	63	3	1	144	37513897
lcl|MN625	Chr3	57.692	26	11	0	61	86	15647458
lcl|MN625	Chr3	52	25	12	0	40	64	15647304
lcl|MN625	Chr3	66.667	21	7	0	125	145	15647744
lcl|MN625	Chr2	40.171	117	39	1	60	145	15225499
lcl|MN625	Chr2	55.556	27	12	0	60	86	15204468
lcl|MN625	Chr2	45.946	37	20	0	45	81	20134894
lcl|MN625	Chr2	61.905	21	8	0	125	145	15204179

TBLASTN 2.10.1+
Query: lcl|MN625520.1_prot_QGY64359.1_1 [gene=CENH3] [protein=histone H3-like centrome
Database: Nippon
Fields: query acc.ver, subject acc.ver, % identity, alignment length, mismatches, gap opens, q. s
24 hits found

lcl|MN625	Chr11	51.389	144	60	3	1	144	2626220
lcl|MN625	Chr6	51.389	144	60	3	1	144	3055622
lcl|MN625	Chr6	51.389	144	60	3	1	144	3040945
lcl|MN625	Chr6	51.389	144	60	3	1	144	3056171
lcl|MN625	Chr6	57.944	107	44	1	38	144	3046891
lcl|MN625	Chr6	57.009	107	45	1	38	144	1646030
lcl|MN625	Chr5	51.389	144	60	3	1	144	21500095
lcl|MN625	Chr5	60.87	46	18	0	35	80	24069190
lcl|MN625	Chr5	44.444	27	15	0	81	107	24068958
lcl|MN625	Chr5	54.545	33	15	0	103	135	24068455
lcl|MN625	Chr4	51.389	144	60	3	1	144	20739761
lcl|MN625	Chr4	37.278	169	43	4	38	144	22463227
lcl|MN625	Chr4	25.472	106	73	2	24	124	22951643
lcl|MN625	Chr1	51.748	143	59	3	1	143	37513897
lcl|MN625	Chr12	48.98	147	59	4	1	144	12808421
lcl|MN625	Chr12	46.259	147	63	5	1	144	12792865

lcl|MN62!	Chr3	60.784	51	20	0	94	144	15647654
lcl|MN62!	Chr3	43.333	30	17	0	38	67	15647301
lcl|MN62!	Chr3	57.692	26	11	0	60	85	15647458
lcl|MN62!	Chr2	39.316	117	40	1	59	144	15225499
lcl|MN62!	Chr2	58.824	51	21	0	94	144	15204269
lcl|MN62!	Chr2	45	40	22	0	59	98	15204468
lcl|MN62!	Chr2	40.678	59	33	1	46	102	20134900
lcl|MN62!	Chr2	45.161	31	17	0	31	61	15225814

TBLASTN 2.10.1+
Query: lcl|MN625519.1_prot_QGY64358.1_1 [gene=CENH3] [protein=histone H3-like centromei
Database: Nippon
Fields: query acc.ver, subject acc.ver, % identity, alignment length, mismatches, gap opens, q. s
24 hits found

lcl|MN62!	Chr11	52.482	141	57	3	1	141	2626220
lcl|MN62!	Chr6	52.482	141	57	3	1	141	3055622
lcl|MN62!	Chr6	52.482	141	57	3	1	141	3040945
lcl|MN62!	Chr6	52.482	141	57	3	1	141	3056171
lcl|MN62!	Chr6	59.615	104	41	1	38	141	3046891
lcl|MN62!	Chr6	58.654	104	42	1	38	141	1646030
lcl|MN62!	Chr5	52.482	141	57	3	1	141	21500095
lcl|MN62!	Chr5	60.87	46	18	0	35	80	24069190
lcl|MN62!	Chr5	44.444	27	15	0	81	107	24068958
lcl|MN62!	Chr5	51.515	33	16	0	103	135	24068455
lcl|MN62!	Chr4	52.482	141	57	3	1	141	20739761
lcl|MN62!	Chr4	37.952	166	40	4	38	141	22463227
lcl|MN62!	Chr4	25.439	114	79	2	16	124	22951667
lcl|MN62!	Chr1	52.482	141	57	3	1	141	37513897
lcl|MN62!	Chr12	49.306	144	57	4	1	141	12808421
lcl|MN62!	Chr12	48.592	142	57	5	1	139	12792865
lcl|MN62!	Chr3	64.583	48	17	0	94	141	15647654
lcl|MN62!	Chr3	48.148	27	14	0	38	64	15647301
lcl|MN62!	Chr3	57.692	26	11	0	60	85	15647458
lcl|MN62!	Chr2	42.105	114	35	1	59	141	15225499
lcl|MN62!	Chr2	66.667	48	16	0	94	141	15204269
lcl|MN62!	Chr2	45	40	22	0	59	98	15204468
lcl|MN62!	Chr2	40.678	59	33	1	46	102	20134900
lcl|MN62!	Chr2	45.161	31	17	0	31	61	15225814

TBLASTN 2.10.1+
Query: lcl|MG384774.1_prot_AUN88460.1_1 [gene=CENH3] [protein=alpha centromeric histon
Database: Nippon
Fields: query acc.ver, subject acc.ver, % identity, alignment length, mismatches, gap opens, q. s
53 hits found

lcl|MG38	Chr11	64	100	35	1	65	164	2626112
lcl|MG38	Chr11	40.909	44	25	1	86	129	18024174
lcl|MG38	Chr11	43.182	44	24	1	86	129	24024670
lcl|MG38	Chr6	64	100	35	1	65	164	3040837
lcl|MG38	Chr6	64	100	35	1	65	164	3055514
lcl|MG38	Chr6	64	100	35	1	65	164	3056279

lcl|MG384	Chr6	64	100	35	1	65	164	3046867
lcl|MG384	Chr6	63	100	36	1	65	164	1646006
lcl|MG384	Chr6	38.636	44	26	1	86	129	27987410
lcl|MG384	Chr6	38.636	44	26	1	86	129	16492799
lcl|MG384	Chr6	38.636	44	26	1	86	129	14859603
lcl|MG384	Chr6	38.636	44	26	1	86	129	23790658
lcl|MG384	Chr5	64	100	35	1	65	164	21499987
lcl|MG384	Chr5	84.211	38	6	0	63	100	24069163
lcl|MG384	Chr5	48.148	27	14	0	100	126	24068958
lcl|MG384	Chr5	76.923	26	6	0	124	149	24068449
lcl|MG384	Chr5	85	20	3	0	1	20	24070126
lcl|MG384	Chr5	55.172	29	12	1	139	166	24068050
lcl|MG384	Chr5	38.636	44	26	1	86	129	11985329
lcl|MG384	Chr5	38.636	44	26	1	86	129	7739575
lcl|MG384	Chr5	36.364	44	27	1	86	129	8333986
lcl|MG384	Chr5	38.636	44	26	1	86	129	11691808
lcl|MG384	Chr5	38.636	44	26	1	86	129	7092135
lcl|MG384	Chr4	64	100	35	1	65	164	20739869
lcl|MG384	Chr4	39.752	161	36	3	65	164	22463251
lcl|MG384	Chr4	38.636	44	26	1	86	129	6034854
lcl|MG384	Chr4	38.636	44	26	1	86	129	6358900
lcl|MG384	Chr1	64.286	98	34	1	65	162	37514005
lcl|MG384	Chr1	40.909	44	25	1	86	129	10092849
lcl|MG384	Chr12	59	100	40	1	65	164	12808313
lcl|MG384	Chr12	57	100	42	1	65	164	12792757
lcl|MG384	Chr12	38.636	44	26	1	86	129	26725609
lcl|MG384	Chr2	42.857	119	37	2	78	165	15225499
lcl|MG384	Chr2	62.264	53	20	0	113	165	15204269
lcl|MG384	Chr2	56.667	30	13	0	78	107	15204468
lcl|MG384	Chr2	51.429	35	17	0	65	99	20134900
lcl|MG384	Chr2	39.583	48	19	2	119	164	20135034
lcl|MG384	Chr2	75	16	4	0	65	80	15225769
lcl|MG384	Chr2	38.636	44	26	1	86	129	15122807
lcl|MG384	Chr3	65.385	52	18	0	113	164	15647654
lcl|MG384	Chr3	54.286	35	16	0	79	113	15647458
lcl|MG384	Chr3	44.444	45	17	2	65	102	15647325
lcl|MG384	Chr3	38.636	44	26	1	86	129	19623246
lcl|MG384	Chr3	38.636	44	26	1	86	129	19302632
lcl|MG384	Chr3	38.636	44	26	1	86	129	19499802
lcl|MG384	Chr10	38.636	44	26	1	86	129	6630385
lcl|MG384	Chr10	38.636	44	26	1	86	129	9325176
lcl|MG384	Chr10	38.636	44	26	1	86	129	11365557
lcl|MG384	Chr10	38.636	44	26	1	86	129	13418214
lcl|MG384	Chr10	38.636	44	26	1	86	129	7380895
lcl|MG384	Chr9	38.636	44	26	1	86	129	7580887
lcl|MG384	Chr7	52.174	23	11	0	107	129	4633593
lcl|MG384	Chr8	38.636	44	26	1	86	129	9874145
# TBLASTN 2.10.1+								

Query: lcl|KT600804.1_prot_AMH40810.1_1 [gene=CENH3] [protein=centromere-specific histor
Database: Nippon
Fields: query acc.ver, subject acc.ver, % identity, alignment length, mismatches, gap opens, q. s
22 hits found

lcl|KT6008	Chr6	57.609	92	38	1	74	165	3040813
lcl|KT6008	Chr6	57.609	92	38	1	74	165	3056303
lcl|KT6008	Chr6	57.609	92	38	1	74	165	3055490
lcl|KT6008	Chr6	57.609	92	38	1	74	165	3046843
lcl|KT6008	Chr6	57.609	92	38	1	74	165	1645982
lcl|KT6008	Chr11	57.609	92	38	1	74	165	2626088
lcl|KT6008	Chr5	57.609	92	38	1	74	165	21499963
lcl|KT6008	Chr5	78.571	28	6	0	74	101	24069133
lcl|KT6008	Chr5	70.37	27	8	0	101	127	24068958
lcl|KT6008	Chr5	61.538	26	10	0	125	150	24068449
lcl|KT6008	Chr5	65	20	7	0	148	167	24068023
lcl|KT6008	Chr5	42.105	38	21	1	3	39	5980000
lcl|KT6008	Chr1	57.778	90	37	1	74	163	37514029
lcl|KT6008	Chr4	57.609	92	38	1	74	165	20739893
lcl|KT6008	Chr4	42.735	117	36	2	80	165	22463383
lcl|KT6008	Chr12	55.435	92	40	1	74	165	12808289
lcl|KT6008	Chr12	54.945	91	40	1	75	165	12792730
lcl|KT6008	Chr2	41.525	118	38	2	79	165	15225499
lcl|KT6008	Chr2	46.835	79	25	2	104	165	15204350
lcl|KT6008	Chr2	63.333	30	10	1	79	107	15204468
lcl|KT6008	Chr3	61.538	52	20	0	114	165	15647654
lcl|KT6008	Chr3	59.259	27	11	0	80	106	15647458

TBLASTN 2.10.1+
Query: lcl|KT600803.1_prot_AMH40809.1_1 [gene=CENH3] [protein=centromere-specific histor
Database: Nippon
Fields: query acc.ver, subject acc.ver, % identity, alignment length, mismatches, gap opens, q. s
33 hits found

lcl|KT6008	Chr6	59.375	96	38	1	70	165	3040825
lcl|KT6008	Chr6	42.424	165	65	4	1	165	3055622
lcl|KT6008	Chr6	59.375	96	38	1	70	165	3056291
lcl|KT6008	Chr6	59.375	96	38	1	70	165	3046855
lcl|KT6008	Chr6	58.333	96	39	1	70	165	1645994
lcl|KT6008	Chr6	33.333	60	38	1	54	113	11621270
lcl|KT6008	Chr11	42.424	165	65	4	1	165	2626220
lcl|KT6008	Chr5	59.375	96	38	1	70	165	21499975
lcl|KT6008	Chr5	68.889	45	14	0	57	101	24069184
lcl|KT6008	Chr5	70.37	27	8	0	101	127	24068958
lcl|KT6008	Chr5	61.538	26	10	0	125	150	24068449
lcl|KT6008	Chr5	76.471	17	4	0	151	167	24068014
lcl|KT6008	Chr5	42.105	38	21	1	3	39	5980000
lcl|KT6008	Chr1	59.574	94	37	1	70	163	37514017
lcl|KT6008	Chr1	31.707	41	26	1	95	135	10032185
lcl|KT6008	Chr1	36.842	38	22	1	25	62	10032051
lcl|KT6008	Chr4	59.375	96	38	1	70	165	20739881

lcl|KT6008	Chr4	36.943	157	38	3	70	165	22463263
lcl|KT6008	Chr4	34.426	61	38	1	54	114	11135552
lcl|KT6008	Chr4	35	60	37	1	54	113	9964857
lcl|KT6008	Chr12	57.292	96	40	1	70	165	12808301
lcl|KT6008	Chr12	48.062	129	62	4	38	165	12792835
lcl|KT6008	Chr12	33.333	60	38	1	54	113	11492926
lcl|KT6008	Chr2	41.525	118	38	2	79	165	15225499
lcl|KT6008	Chr2	46.835	79	25	2	104	165	15204350
lcl|KT6008	Chr2	63.333	30	10	1	79	107	15204468
lcl|KT6008	Chr2	48.485	33	15	1	50	82	20134858
lcl|KT6008	Chr2	36	50	22	2	120	167	20135034
lcl|KT6008	Chr3	61.538	52	20	0	114	165	15647654
lcl|KT6008	Chr3	59.259	27	11	0	80	106	15647458
lcl|KT6008	Chr8	38.333	60	35	1	54	113	22270002
lcl|KT6008	Chr8	33.333	60	38	1	54	113	22986630
lcl|KT6008	Chr8	23.622	127	91	2	40	162	27133858

TBLASTN 2.10.1+
Query: lcl|KP878239.1_prot_AKI32619.1_1 [gene=CENH3] [protein=centromere specific histone
Database: Nippon
Fields: query acc.ver, subject acc.ver, % identity, alignment length, mismatches, gap opens, q. s
31 hits found

lcl|KP8782	Chr6	52.542	118	52	3	38	154	3046915
lcl|KP8782	Chr6	58.333	96	39	1	59	154	3040825
lcl|KP8782	Chr6	58.333	96	39	1	59	154	3055502
lcl|KP8782	Chr6	58.333	96	39	1	59	154	3056291
lcl|KP8782	Chr6	57.292	96	40	1	59	154	1645994
lcl|KP8782	Chr6	42.5	40	11	1	38	65	7680155
lcl|KP8782	Chr11	58.333	96	39	1	59	154	2626100
lcl|KP8782	Chr5	58.333	96	39	1	59	154	21499975
lcl|KP8782	Chr5	65.306	49	16	1	43	90	24069196
lcl|KP8782	Chr5	66.667	27	9	0	90	116	24068958
lcl|KP8782	Chr5	61.538	26	10	0	114	139	24068449
lcl|KP8782	Chr5	68.421	19	6	0	138	156	24068020
lcl|KP8782	Chr4	58.333	96	39	1	59	154	20739881
lcl|KP8782	Chr4	32.804	189	59	4	34	154	22463167
lcl|KP8782	Chr4	33.871	62	35	2	46	103	11135555
lcl|KP8782	Chr4	32.203	59	38	1	46	102	21299723
lcl|KP8782	Chr1	58.511	94	38	1	59	152	37514017
lcl|KP8782	Chr12	43.949	157	63	5	1	154	12808421
lcl|KP8782	Chr12	55.208	96	42	1	59	154	12792745
lcl|KP8782	Chr12	32.787	61	35	2	46	102	11492929
lcl|KP8782	Chr12	32.292	96	59	3	8	103	2433708
lcl|KP8782	Chr2	40.678	118	39	2	68	154	15225499
lcl|KP8782	Chr2	45.57	79	26	2	93	154	15204350
lcl|KP8782	Chr2	63.333	30	10	1	68	96	15204468
lcl|KP8782	Chr2	37.255	51	22	2	108	156	20135031
lcl|KP8782	Chr2	84.615	13	2	0	59	71	20134912
lcl|KP8782	Chr3	59.615	52	21	0	103	154	15647654

lcl|KP8782	Chr3	55.172	29	13	0	69	97	15647458
lcl|KP8782	Chr8	35.593	59	36	1	46	102	22270005
lcl|KP8782	Chr10	28.125	96	58	2	31	120	8621404
lcl|KP8782	Chr7	28.169	71	47	2	88	156	28183548

TBLASTN 2.10.1+
Query: lcl|KP878237.1_prot_AKI32617.1_1 [gene=CENH3] [protein=centromere specific histone
Database: Nippon
Fields: query acc.ver, subject acc.ver, % identity, alignment length, mismatches, gap opens, q. s
13 hits found

lcl|KP8782	Chr5	54.762	42	18	1	43	83	24069196
lcl|KP8782	Chr5	75	20	5	0	59	78	21499975
lcl|KP8782	Chr12	33.333	99	39	5	1	93	12808421
lcl|KP8782	Chr12	47.368	38	17	1	59	93	12792745
lcl|KP8782	Chr6	50	42	18	2	38	78	3046915
lcl|KP8782	Chr6	47.368	38	17	1	59	93	3056291
lcl|KP8782	Chr6	75	20	5	0	59	78	3040825
lcl|KP8782	Chr6	44.737	38	18	1	59	93	1645994
lcl|KP8782	Chr6	75	20	5	0	59	78	3055502
lcl|KP8782	Chr6	42.5	40	11	1	38	65	7680155
lcl|KP8782	Chr4	47.368	38	17	1	59	93	20739881
lcl|KP8782	Chr11	75	20	5	0	59	78	2626100
lcl|KP8782	Chr1	75	20	5	0	59	78	37514017

TBLASTN 2.10.1+
Query: lcl|KP878236.1_prot_AKI32616.1_1 [gene=CENH3] [protein=centromere specific histone
Database: Nippon
Fields: query acc.ver, subject acc.ver, % identity, alignment length, mismatches, gap opens, q. s
23 hits found

lcl|KP8782	Chr6	50	118	55	3	38	154	3046915
lcl|KP8782	Chr6	55.208	96	42	1	59	154	3040825
lcl|KP8782	Chr6	55.208	96	42	1	59	154	3056291
lcl|KP8782	Chr6	55.208	96	42	1	59	154	3055502
lcl|KP8782	Chr6	54.167	96	43	1	59	154	1645994
lcl|KP8782	Chr6	42.5	40	11	1	38	65	7680155
lcl|KP8782	Chr11	55.208	96	42	1	59	154	2626100
lcl|KP8782	Chr5	55.208	96	42	1	59	154	21499975
lcl|KP8782	Chr5	63.265	49	17	1	43	90	24069196
lcl|KP8782	Chr5	62.963	27	10	0	90	116	24068958
lcl|KP8782	Chr5	61.538	26	10	0	114	139	24068449
lcl|KP8782	Chr1	56.383	94	40	1	59	152	37514017
lcl|KP8782	Chr4	55.208	96	42	1	59	154	20739881
lcl|KP8782	Chr4	31.217	189	62	4	34	154	22463167
lcl|KP8782	Chr12	43.312	157	64	5	1	154	12808421
lcl|KP8782	Chr12	54.167	96	43	1	59	154	12792745
lcl|KP8782	Chr2	38.136	118	42	2	68	154	15225499
lcl|KP8782	Chr2	44.304	79	27	2	93	154	15204350
lcl|KP8782	Chr2	56.667	30	12	1	68	96	15204468
lcl|KP8782	Chr2	38.776	49	20	2	108	154	20135031
lcl|KP8782	Chr2	76.923	13	3	0	59	71	20134912

lcl|KP8782	Chr3	57.692	52	22	0	103	154	15647654
lcl|KP8782	Chr3	50	28	14	0	70	97	15647461

TBLASTN 2.10.1+
Query: lcl|KP878232.1_prot_AKI32612.1_1 [gene=CENH3] [protein=centromere specific histone
Database: Nippon
Fields: query acc.ver, subject acc.ver, % identity, alignment length, mismatches, gap opens, q. s
21 hits found

lcl|KP8782	Chr6	52.055	73	34	1	74	146	3040813
lcl|KP8782	Chr6	52.055	73	34	1	74	146	3055490
lcl|KP8782	Chr6	52.055	73	34	1	74	146	3056303
lcl|KP8782	Chr6	52.055	73	34	1	74	146	3046843
lcl|KP8782	Chr6	52.055	73	34	1	74	146	1645982
lcl|KP8782	Chr11	52.055	73	34	1	74	146	2626088
lcl|KP8782	Chr5	52.055	73	34	1	74	146	21499963
lcl|KP8782	Chr5	78.571	28	6	0	74	101	24069133
lcl|KP8782	Chr5	70.37	27	8	0	101	127	24068958
lcl|KP8782	Chr5	46.429	28	15	0	125	152	24068449
lcl|KP8782	Chr5	42.105	38	21	1	3	39	5980000
lcl|KP8782	Chr1	52.778	72	33	1	74	145	37514029
lcl|KP8782	Chr4	48.75	80	40	1	74	153	20739893
lcl|KP8782	Chr4	34.286	105	38	2	80	153	22463383
lcl|KP8782	Chr12	52.055	73	34	1	74	146	12808289
lcl|KP8782	Chr12	51.389	72	34	1	75	146	12792730
lcl|KP8782	Chr2	33.962	106	39	2	79	153	15225499
lcl|KP8782	Chr2	42.5	40	23	0	114	153	15204269
lcl|KP8782	Chr2	63.333	30	10	1	79	107	15204468
lcl|KP8782	Chr3	53.125	32	15	0	114	145	15647654
lcl|KP8782	Chr3	59.259	27	11	0	80	106	15647458

TBLASTN 2.10.1+
Query: lcl|KP878228.1_prot_AKI32608.1_1 [gene=CENH3] [protein=centromere specific histone
Database: Nippon
Fields: query acc.ver, subject acc.ver, % identity, alignment length, mismatches, gap opens, q. s
8 hits found

lcl|KP8782	Chr11	52	25	12	0	47	71	5306225
lcl|KP8782	Chr5	42.105	38	21	1	3	39	5980000
lcl|KP8782	Chr5	42.857	42	20	1	27	68	11875930
lcl|KP8782	Chr12	40.476	42	25	0	28	69	2141632
lcl|KP8782	Chr2	30.137	73	46	2	4	71	11990306
lcl|KP8782	Chr6	42.424	33	19	0	39	71	25463760
lcl|KP8782	Chr3	40.541	37	21	1	3	38	35302276
lcl|KP8782	Chr10	35.714	28	18	0	42	69	17187504

TBLASTN 2.10.1+
Query: lcl|KP878222.1_prot_AKI32602.1_1 [gene=CENH3] [protein=centromere specific histone
Database: Nippon
Fields: query acc.ver, subject acc.ver, % identity, alignment length, mismatches, gap opens, q. s
20 hits found

lcl|KP8782	Chr6	57.609	92	38	1	65	156	3040813
lcl|KP8782	Chr6	57.609	92	38	1	65	156	3056303

lcl|KP878	Chr6	57.609	92	38	1	65	156	3055490
lcl|KP878	Chr6	57.609	92	38	1	65	156	3046843
lcl|KP878	Chr6	57.609	92	38	1	65	156	1645982
lcl|KP878	Chr11	57.609	92	38	1	65	156	2626088
lcl|KP878	Chr5	57.609	92	38	1	65	156	21499963
lcl|KP878	Chr5	78.571	28	6	0	65	92	24069133
lcl|KP878	Chr5	70.37	27	8	0	92	118	24068958
lcl|KP878	Chr5	61.538	26	10	0	116	141	24068449
lcl|KP878	Chr1	57.778	90	37	1	65	154	37514029
lcl|KP878	Chr4	57.609	92	38	1	65	156	20739893
lcl|KP878	Chr4	42.735	117	36	2	71	156	22463383
lcl|KP878	Chr12	56.522	92	39	1	65	156	12808289
lcl|KP878	Chr12	56.044	91	39	1	66	156	12792730
lcl|KP878	Chr2	41.525	118	38	2	70	156	15225499
lcl|KP878	Chr2	46.835	79	25	2	95	156	15204350
lcl|KP878	Chr2	63.333	30	10	1	70	98	15204468
lcl|KP878	Chr3	61.538	52	20	0	105	156	15647654
lcl|KP878	Chr3	59.259	27	11	0	71	97	15647458

TBLASTN 2.10.1+
Query: lcl|KP878221.1_prot_AKI32601.1_1 [gene=CENH3] [protein=centromere specific histone
Database: Nippon
Fields: query acc.ver, subject acc.ver, % identity, alignment length, mismatches, gap opens, q. s
23 hits found

lcl|KP878	Chr6	59.375	96	38	1	59	154	3040825
lcl|KP878	Chr6	45.455	154	65	4	1	154	3055622
lcl|KP878	Chr6	59.375	96	38	1	59	154	3056291
lcl|KP878	Chr6	59.375	96	38	1	59	154	3046855
lcl|KP878	Chr6	58.333	96	39	1	59	154	1645994
lcl|KP878	Chr11	45.455	154	65	4	1	154	2626220
lcl|KP878	Chr5	59.375	96	38	1	59	154	21499975
lcl|KP878	Chr5	70.732	41	12	0	50	90	24069172
lcl|KP878	Chr5	70.37	27	8	0	90	116	24068958
lcl|KP878	Chr5	61.538	26	10	0	114	139	24068449
lcl|KP878	Chr5	65	20	7	0	137	156	24068023
lcl|KP878	Chr1	59.574	94	37	1	59	152	37514017
lcl|KP878	Chr4	59.375	96	38	1	59	154	20739881
lcl|KP878	Chr4	36.943	157	38	3	59	154	22463263
lcl|KP878	Chr12	57.292	96	40	1	59	154	12808301
lcl|KP878	Chr12	56.25	96	41	1	59	154	12792745
lcl|KP878	Chr2	41.525	118	38	2	68	154	15225499
lcl|KP878	Chr2	46.835	79	25	2	93	154	15204350
lcl|KP878	Chr2	63.333	30	10	1	68	96	15204468
lcl|KP878	Chr2	84.615	13	2	0	59	71	20134912
lcl|KP878	Chr2	36	50	22	2	109	156	20135034
lcl|KP878	Chr3	61.538	52	20	0	103	154	15647654
lcl|KP878	Chr3	45.714	35	19	0	69	103	15647458

TBLASTN 2.10.1+
Query: lcl|KP878220.1_prot_AKI32600.1_1 [gene=CENH3] [protein=centromere specific histone

Database: Nippon
Fields: query acc.ver, subject acc.ver, % identity, alignment length, mismatches, gap opens, q. s
30 hits found

lcl|KP8782	Chr6	47.436	156	74	6	11	165	3040987
lcl|KP8782	Chr6	59.375	96	38	1	70	165	3056291
lcl|KP8782	Chr6	59.375	96	38	1	70	165	3055502
lcl|KP8782	Chr6	59.375	96	38	1	70	165	3046855
lcl|KP8782	Chr6	58.333	96	39	1	70	165	1645994
lcl|KP8782	Chr6	33.333	60	38	1	54	113	11621270
lcl|KP8782	Chr11	59.375	96	38	1	70	165	2626100
lcl|KP8782	Chr5	59.375	96	38	1	70	165	21499975
lcl|KP8782	Chr5	68.889	45	14	0	57	101	24069184
lcl|KP8782	Chr5	58.333	36	14	1	101	135	24068958
lcl|KP8782	Chr5	61.538	26	10	0	125	150	24068449
lcl|KP8782	Chr5	65	20	7	0	148	167	24068023
lcl|KP8782	Chr1	59.574	94	37	1	70	163	37514017
lcl|KP8782	Chr4	59.375	96	38	1	70	165	20739881
lcl|KP8782	Chr4	36.943	157	38	3	70	165	22463263
lcl|KP8782	Chr4	34.426	61	38	1	54	114	11135552
lcl|KP8782	Chr4	35	60	37	1	54	113	9964857
lcl|KP8782	Chr12	57.292	96	40	1	70	165	12808301
lcl|KP8782	Chr12	48.062	129	62	4	38	165	12792835
lcl|KP8782	Chr12	33.333	60	38	1	54	113	11492926
lcl|KP8782	Chr2	41.525	118	38	2	79	165	15225499
lcl|KP8782	Chr2	46.835	79	25	2	104	165	15204350
lcl|KP8782	Chr2	63.333	30	10	1	79	107	15204468
lcl|KP8782	Chr2	48.485	33	15	1	50	82	20134858
lcl|KP8782	Chr2	36	50	22	2	120	167	20135034
lcl|KP8782	Chr3	61.538	52	20	0	114	165	15647654
lcl|KP8782	Chr3	59.259	27	11	0	80	106	15647458
lcl|KP8782	Chr8	38.333	60	35	1	54	113	22270002
lcl|KP8782	Chr8	33.333	60	38	1	54	113	22986630
lcl|KP8782	Chr8	23.256	129	93	2	38	162	27133864

TBLASTN 2.10.1+
Query: lcl|AB770164.1_prot_BAP26971.1_1 [gene=CENH3] [protein=centromeric histone H3 iso
Database: Nippon
Fields: query acc.ver, subject acc.ver, % identity, alignment length, mismatches, gap opens, q. s
23 hits found

lcl|AB770	Chr11	64.356	101	35	1	68	168	2626115
lcl|AB770	Chr6	64.356	101	35	1	68	168	3040840
lcl|AB770	Chr6	64.356	101	35	1	68	168	3055517
lcl|AB770	Chr6	64.356	101	35	1	68	168	3046870
lcl|AB770	Chr6	64.356	101	35	1	68	168	3056276
lcl|AB770	Chr6	65.347	101	34	1	68	168	1646009
lcl|AB770	Chr5	64.356	101	35	1	68	168	21499990
lcl|AB770	Chr5	80.556	36	7	0	69	104	24069157
lcl|AB770	Chr5	75	16	4	0	104	119	24068958
lcl|AB770	Chr5	77.778	27	6	0	1	27	24070126

lcl|AB770	Chr5	68.182	22	7	0	132	153	24068437
lcl|AB770	Chr4	64.356	101	35	1	68	168	20739866
lcl|AB770	Chr4	40.123	162	36	2	68	168	22463248
lcl|AB770	Chr1	64.646	99	34	1	68	166	37514002
lcl|AB770	Chr12	59.406	101	40	1	68	168	12808316
lcl|AB770	Chr12	58.416	101	41	1	68	168	12792760
lcl|AB770	Chr2	44.068	118	35	1	82	168	15225499
lcl|AB770	Chr2	64.865	37	13	0	133	169	15204221
lcl|AB770	Chr2	58.065	31	13	0	81	111	15204471
lcl|AB770	Chr2	73.684	19	5	0	68	86	20134897
lcl|AB770	Chr3	69.444	36	11	0	133	168	15647702
lcl|AB770	Chr3	54.286	35	16	0	83	117	15647458
lcl|AB770	Chr3	70.588	17	5	0	68	84	15647322

TBLASTN 2.10.1+
Query: lcl|AB770163.1_prot_BAP26970.1_1 [gene=CENH3] [protein=centromeric histone H3 iso
Database: Nippon
Fields: query acc.ver, subject acc.ver, % identity, alignment length, mismatches, gap opens, q. s
23 hits found

lcl|AB770	Chr11	64.356	101	35	1	61	161	2626115
lcl|AB770	Chr6	64.356	101	35	1	61	161	3040840
lcl|AB770	Chr6	64.356	101	35	1	61	161	3055517
lcl|AB770	Chr6	64.356	101	35	1	61	161	3046870
lcl|AB770	Chr6	64.356	101	35	1	61	161	3056276
lcl|AB770	Chr6	65.347	101	34	1	61	161	1646009
lcl|AB770	Chr5	64.356	101	35	1	61	161	21499990
lcl|AB770	Chr5	80.556	36	7	0	62	97	24069157
lcl|AB770	Chr5	75	16	4	0	97	112	24068958
lcl|AB770	Chr5	77.778	27	6	0	1	27	24070126
lcl|AB770	Chr5	68.182	22	7	0	125	146	24068437
lcl|AB770	Chr4	64.356	101	35	1	61	161	20739866
lcl|AB770	Chr4	40.123	162	36	2	61	161	22463248
lcl|AB770	Chr1	64.646	99	34	1	61	159	37514002
lcl|AB770	Chr12	59.406	101	40	1	61	161	12808316
lcl|AB770	Chr12	58.416	101	41	1	61	161	12792760
lcl|AB770	Chr2	44.068	118	35	1	75	161	15225499
lcl|AB770	Chr2	64.865	37	13	0	126	162	15204221
lcl|AB770	Chr2	58.065	31	13	0	74	104	15204471
lcl|AB770	Chr2	73.684	19	5	0	61	79	20134897
lcl|AB770	Chr3	69.444	36	11	0	126	161	15647702
lcl|AB770	Chr3	54.286	35	16	0	76	110	15647458
lcl|AB770	Chr3	70.588	17	5	0	61	77	15647322

TBLASTN 2.10.1+
Query: lcl|JF969287.1_prot_AEH95352.1_1 [gene=CENH3] [protein=centromeric histone 3] [pro
Database: Nippon
Fields: query acc.ver, subject acc.ver, % identity, alignment length, mismatches, gap opens, q. s
56 hits found

lcl|JF9692	Chr6	62	100	37	1	62	161	3040837
lcl|JF9692	Chr6	62	100	37	1	62	161	3055514

lcl|JF9692 Chr6	62	100	37	1	62	161	3056279
lcl|JF9692 Chr6	62	100	37	1	62	161	3046867
lcl|JF9692 Chr6	61	100	38	1	62	161	1646006
lcl|JF9692 Chr6	38.636	44	26	1	83	126	27987410
lcl|JF9692 Chr6	38.636	44	26	1	83	126	16492799
lcl|JF9692 Chr6	38.636	44	26	1	83	126	14859603
lcl|JF9692 Chr6	38.636	44	26	1	83	126	23790658
lcl|JF9692 Chr11	62	100	37	1	62	161	2626112
lcl|JF9692 Chr11	40.909	44	25	1	83	126	18024174
lcl|JF9692 Chr11	43.182	44	24	1	83	126	24024670
lcl|JF9692 Chr5	62	100	37	1	62	161	21499987
lcl|JF9692 Chr5	78.947	38	8	0	60	97	24069163
lcl|JF9692 Chr5	48.148	27	14	0	97	123	24068958
lcl|JF9692 Chr5	76.923	26	6	0	121	146	24068449
lcl|JF9692 Chr5	66.667	30	6	1	1	26	24070126
lcl|JF9692 Chr5	55.172	29	12	1	136	163	24068050
lcl|JF9692 Chr5	38.636	44	26	1	83	126	11985329
lcl|JF9692 Chr5	38.636	44	26	1	83	126	7739575
lcl|JF9692 Chr5	36.364	44	27	1	83	126	8333986
lcl|JF9692 Chr5	38.636	44	26	1	83	126	11691808
lcl|JF9692 Chr5	38.636	44	26	1	83	126	7092135
lcl|JF9692 Chr4	62	100	37	1	62	161	20739869
lcl|JF9692 Chr4	38.509	161	38	3	62	161	22463251
lcl|JF9692 Chr4	38.636	44	26	1	83	126	6034854
lcl|JF9692 Chr4	38.636	44	26	1	83	126	6358900
lcl|JF9692 Chr4	38.636	44	26	1	83	126	16909571
lcl|JF9692 Chr1	62.245	98	36	1	62	159	37514005
lcl|JF9692 Chr1	40.909	44	25	1	83	126	10092849
lcl|JF9692 Chr12	58	100	41	1	62	161	12808313
lcl|JF9692 Chr12	56	100	43	1	62	161	12792757
lcl|JF9692 Chr12	38.636	44	26	1	83	126	26725609
lcl|JF9692 Chr2	41.176	119	39	2	75	162	15225499
lcl|JF9692 Chr2	62.264	53	20	0	110	162	15204269
lcl|JF9692 Chr2	50	30	15	0	75	104	15204468
lcl|JF9692 Chr2	54.286	35	16	0	62	96	20134900
lcl|JF9692 Chr2	39.583	48	19	2	116	161	20135034
lcl|JF9692 Chr2	75	16	4	0	62	77	15225769
lcl|JF9692 Chr2	38.636	44	26	1	83	126	15122807
lcl|JF9692 Chr3	65.385	52	18	0	110	161	15647654
lcl|JF9692 Chr3	48.571	35	18	0	76	110	15647458
lcl|JF9692 Chr3	38.636	44	26	1	83	126	19623246
lcl|JF9692 Chr3	38.636	44	26	1	83	126	19302632
lcl|JF9692 Chr3	44.444	45	17	2	62	99	15647325
lcl|JF9692 Chr3	38.636	44	26	1	83	126	19499802
lcl|JF9692 Chr10	38.636	44	26	1	83	126	6630385
lcl|JF9692 Chr10	38.636	44	26	1	83	126	9325176
lcl|JF9692 Chr10	38.636	44	26	1	83	126	11365557
lcl|JF9692 Chr10	38.636	44	26	1	83	126	13418214

lcl|JF9692 Chr10	38.636	44	26	1	83	126	7380895
lcl|JF9692 Chr9	38.636	44	26	1	83	126	7580887
lcl|JF9692 Chr9	36.364	44	27	1	83	126	2880881
lcl|JF9692 Chr7	38.636	44	26	1	83	126	4633533
lcl|JF9692 Chr8	38.636	44	26	1	83	126	9874145
lcl|JF9692 Chr8	47.826	23	12	0	104	126	13449432

TBLASTN 2.10.1+
Query: lcl|JF969285.1_prot_AEH95350.1_1 [gene=CENH3] [protein=centromeric histone 3] [pro
Database: Nippon
Fields: query acc.ver, subject acc.ver, % identity, alignment length, mismatches, gap opens, q. s
53 hits found

lcl|JF9692 Chr6	63	100	36	1	65	164	3040837
lcl|JF9692 Chr6	63	100	36	1	65	164	3055514
lcl|JF9692 Chr6	63	100	36	1	65	164	3056279
lcl|JF9692 Chr6	63	100	36	1	65	164	3046867
lcl|JF9692 Chr6	62	100	37	1	65	164	1646006
lcl|JF9692 Chr6	38.636	44	26	1	86	129	27987410
lcl|JF9692 Chr6	38.636	44	26	1	86	129	16492799
lcl|JF9692 Chr6	38.636	44	26	1	86	129	14859603
lcl|JF9692 Chr6	38.636	44	26	1	86	129	23790658
lcl|JF9692 Chr11	63	100	36	1	65	164	2626112
lcl|JF9692 Chr11	40.909	44	25	1	86	129	18024174
lcl|JF9692 Chr11	44.186	43	23	1	86	128	24024670
lcl|JF9692 Chr5	63	100	36	1	65	164	21499987
lcl|JF9692 Chr5	81.579	38	7	0	63	100	24069163
lcl|JF9692 Chr5	48.148	27	14	0	100	126	24068958
lcl|JF9692 Chr5	76.923	26	6	0	124	149	24068449
lcl|JF9692 Chr5	85	20	3	0	1	20	24070126
lcl|JF9692 Chr5	55.172	29	12	1	139	166	24068050
lcl|JF9692 Chr5	38.636	44	26	1	86	129	11985329
lcl|JF9692 Chr5	38.636	44	26	1	86	129	7739575
lcl|JF9692 Chr5	36.364	44	27	1	86	129	8333986
lcl|JF9692 Chr5	38.636	44	26	1	86	129	11691808
lcl|JF9692 Chr5	38.636	44	26	1	86	129	7092135
lcl|JF9692 Chr4	63	100	36	1	65	164	20739869
lcl|JF9692 Chr4	39.13	161	37	3	65	164	22463251
lcl|JF9692 Chr4	38.636	44	26	1	86	129	6034854
lcl|JF9692 Chr4	38.636	44	26	1	86	129	6358900
lcl|JF9692 Chr1	63.265	98	35	1	65	162	37514005
lcl|JF9692 Chr1	40.909	44	25	1	86	129	10092849
lcl|JF9692 Chr12	58	100	41	1	65	164	12808313
lcl|JF9692 Chr12	56	100	43	1	65	164	12792757
lcl|JF9692 Chr12	38.636	44	26	1	86	129	26725609
lcl|JF9692 Chr2	42.017	119	38	2	78	165	15225499
lcl|JF9692 Chr2	62.264	53	20	0	113	165	15204269
lcl|JF9692 Chr2	53.333	30	14	0	78	107	15204468
lcl|JF9692 Chr2	51.429	35	17	0	65	99	20134900
lcl|JF9692 Chr2	39.583	48	19	2	119	164	20135034

lcl|JF9692	Chr2	75	16	4	0	65	80	15225769
lcl|JF9692	Chr2	38.636	44	26	1	86	129	15122807
lcl|JF9692	Chr3	65.385	52	18	0	113	164	15647654
lcl|JF9692	Chr3	51.429	35	17	0	79	113	15647458
lcl|JF9692	Chr3	38.636	44	26	1	86	129	19623246
lcl|JF9692	Chr3	44.444	45	17	2	65	102	15647325
lcl|JF9692	Chr3	38.636	44	26	1	86	129	19302632
lcl|JF9692	Chr3	38.636	44	26	1	86	129	19499802
lcl|JF9692	Chr10	38.636	44	26	1	86	129	6630385
lcl|JF9692	Chr10	38.636	44	26	1	86	129	9325176
lcl|JF9692	Chr10	38.636	44	26	1	86	129	11365557
lcl|JF9692	Chr10	38.636	44	26	1	86	129	13418214
lcl|JF9692	Chr10	38.636	44	26	1	86	129	7380895
lcl|JF9692	Chr9	38.636	44	26	1	86	129	7580887
lcl|JF9692	Chr7	38.636	44	26	1	86	129	4633533
lcl|JF9692	Chr8	38.636	44	26	1	86	129	9874145

TBLASTN 2.10.1+
Query: lcl|JF419330.1_prot_AEK21394.1_1 [gene=CENH3] [protein=beta centromeric histone H3
Database: Nippon
Fields: query acc.ver, subject acc.ver, % identity, alignment length, mismatches, gap opens, q. s
26 hits found

lcl|JF4193	Chr11	51.825	137	53	3	1	130	2626220
lcl|JF4193	Chr6	51.825	137	53	3	1	130	3055622
lcl|JF4193	Chr6	51.825	137	53	3	1	130	3040945
lcl|JF4193	Chr6	61.386	101	33	1	30	130	1646006
lcl|JF4193	Chr6	51.825	137	53	3	1	130	3056171
lcl|JF4193	Chr6	60.396	101	34	1	30	130	3046867
lcl|JF4193	Chr5	53.285	137	51	4	1	130	21500095
lcl|JF4193	Chr5	75.61	41	10	0	25	65	24069172
lcl|JF4193	Chr5	67.742	31	10	0	89	119	24068464
lcl|JF4193	Chr1	53.285	137	51	4	1	130	37513897
lcl|JF4193	Chr4	51.825	137	53	3	1	130	20739761
lcl|JF4193	Chr4	38.217	157	41	2	30	130	22463251
lcl|JF4193	Chr4	45.946	37	20	0	16	52	31958781
lcl|JF4193	Chr12	48.905	137	57	4	1	130	12808421
lcl|JF4193	Chr12	46.377	138	61	4	1	131	12792865
lcl|JF4193	Chr12	28.788	66	47	0	12	77	3562813
lcl|JF4193	Chr12	41.176	34	20	0	102	135	3718543
lcl|JF4193	Chr3	68.182	44	14	0	87	130	15647666
lcl|JF4193	Chr3	72.414	29	8	0	44	72	15647458
lcl|JF4193	Chr3	68.75	16	5	0	30	45	15647325
lcl|JF4193	Chr2	44.737	114	37	1	43	130	15225499
lcl|JF4193	Chr2	65.909	44	15	0	87	130	15204257
lcl|JF4193	Chr2	70	30	9	0	43	72	15204468
lcl|JF4193	Chr2	75	20	5	0	30	49	20134900
lcl|JF4193	Chr2	42.857	28	16	0	8	35	27393711
lcl|JF4193	Chr8	38.095	42	24	1	21	62	4507089

TBLASTN 2.10.1+

Query: lcl|JF419329.1_prot_AEK21393.1_1 [gene=CENH3] [protein=beta centromeric histone H3
Database: Nippon
Fields: query acc.ver, subject acc.ver, % identity, alignment length, mismatches, gap opens, q. s
23 hits found

lcl|JF4193	Chr11	57.547	106	34	3	34	137	2626103
lcl|JF4193	Chr6	57.547	106	34	3	34	137	3040828
lcl|JF4193	Chr6	57.547	106	34	3	34	137	3055505
lcl|JF4193	Chr6	57.547	106	34	3	34	137	3046858
lcl|JF4193	Chr6	57.143	105	36	2	34	137	3056288
lcl|JF4193	Chr6	57	100	35	1	34	133	1645997
lcl|JF4193	Chr5	57.143	105	36	2	34	137	21499978
lcl|JF4193	Chr5	55.357	56	25	0	32	87	24069154
lcl|JF4193	Chr5	61.29	31	12	0	92	122	24068464
lcl|JF4193	Chr4	57.143	105	36	2	34	137	20739878
lcl|JF4193	Chr4	37.179	156	44	3	32	133	22463254
lcl|JF4193	Chr1	56.863	102	36	2	32	133	37514008
lcl|JF4193	Chr1	30.337	89	51	4	39	117	17014352
lcl|JF4193	Chr12	54	100	38	1	34	133	12808304
lcl|JF4193	Chr12	50.495	101	42	1	34	134	12792748
lcl|JF4193	Chr2	44.737	114	39	2	44	133	15225499
lcl|JF4193	Chr2	72.093	43	12	0	91	133	15204254
lcl|JF4193	Chr2	60	30	12	0	44	73	15204468
lcl|JF4193	Chr2	76.471	17	4	0	34	50	20134909
lcl|JF4193	Chr3	69.767	43	13	0	91	133	15647669
lcl|JF4193	Chr3	62.069	29	11	0	45	73	15647458
lcl|JF4193	Chr3	60	15	6	0	32	46	15647328
lcl|JF4193	Chr7	43.75	32	14	1	111	138	16364996

TBLASTN 2.10.1+
Query: lcl|GU166744.1_prot_ACZ04984.1_1 [gene=CENH3] [protein=centromere-specific H3 va
Database: Nippon
Fields: query acc.ver, subject acc.ver, % identity, alignment length, mismatches, gap opens, q. s
23 hits found

lcl|GU166	Chr6	53.774	106	47	1	72	177	1646024
lcl|GU166	Chr6	53.774	106	47	2	72	177	3040855
lcl|GU166	Chr6	52.83	106	48	1	72	177	3056261
lcl|GU166	Chr6	53.774	106	47	2	72	177	3055532
lcl|GU166	Chr6	52.83	106	48	1	72	177	3046885
lcl|GU166	Chr1	53.333	105	47	1	72	176	37513987
lcl|GU166	Chr5	53.774	106	47	2	72	177	21500005
lcl|GU166	Chr5	60.976	41	16	0	73	113	24069172
lcl|GU166	Chr5	51.724	29	13	1	112	140	24068961
lcl|GU166	Chr5	48.387	31	16	0	138	168	24068449
lcl|GU166	Chr11	53.774	106	47	2	72	177	2626130
lcl|GU166	Chr11	32.308	65	39	2	104	164	1069460
lcl|GU166	Chr4	52.83	106	48	1	72	177	20739851
lcl|GU166	Chr4	34.731	167	47	3	72	177	22463233
lcl|GU166	Chr12	51.376	109	45	2	72	177	12808331
lcl|GU166	Chr12	48.113	106	53	1	72	177	12792775

lcl|GU166	Chr3	58.824	51	21	0	127	177	15647654
lcl|GU166	Chr3	51.724	29	14	0	92	120	15647458
lcl|GU166	Chr3	50	24	12	0	72	95	15647307
lcl|GU166	Chr2	38.983	118	40	2	91	177	15225499
lcl|GU166	Chr2	56.863	51	22	0	127	177	15204269
lcl|GU166	Chr2	53.333	30	14	0	91	120	15204468
lcl|GU166	Chr2	29.688	64	42	1	97	160	24733354

TBLASTN 2.10.1+
Query: lcl|GU166742.1_prot_ACZ04982.1_1 [gene=CENH3] [protein=centromere-specific H3 va
Database: Nippon
Fields: query acc.ver, subject acc.ver, % identity, alignment length, mismatches, gap opens, q. s
22 hits found

lcl|GU166	Chr6	52.294	109	50	1	72	180	1646033
lcl|GU166	Chr6	49.242	132	65	2	49	180	3055610
lcl|GU166	Chr6	49.242	132	65	2	49	180	3040933
lcl|GU166	Chr6	51.376	109	51	1	72	180	3056252
lcl|GU166	Chr6	51.376	109	51	1	72	180	3046894
lcl|GU166	Chr1	51.852	108	50	1	72	179	37513978
lcl|GU166	Chr11	49.242	132	65	2	49	180	2626208
lcl|GU166	Chr5	52.294	109	50	2	72	180	21500014
lcl|GU166	Chr5	60.976	41	16	0	76	116	24069172
lcl|GU166	Chr5	55.172	29	12	1	115	143	24068961
lcl|GU166	Chr5	48.387	31	16	0	141	171	24068449
lcl|GU166	Chr4	51.376	109	51	1	72	180	20739842
lcl|GU166	Chr4	34.118	170	50	3	72	180	22463224
lcl|GU166	Chr12	45	140	69	2	44	180	12808424
lcl|GU166	Chr12	42.336	137	77	1	44	180	12792868
lcl|GU166	Chr3	58.824	51	21	0	130	180	15647654
lcl|GU166	Chr3	51.724	29	14	0	95	123	15647458
lcl|GU166	Chr3	48	25	13	0	72	96	15647298
lcl|GU166	Chr2	38.983	118	40	2	94	180	15225499
lcl|GU166	Chr2	56.863	51	22	0	130	180	15204269
lcl|GU166	Chr2	53.333	30	14	0	94	123	15204468
lcl|GU166	Chr2	29.688	64	42	1	100	163	24733354

TBLASTN 2.10.1+
Query: lcl|GU166740.1_prot_ACZ04980.1_1 [gene=CENH3] [protein=centromere-specific H3 va
Database: Nippon
Fields: query acc.ver, subject acc.ver, % identity, alignment length, mismatches, gap opens, q. s
19 hits found

lcl|GU166	Chr6	53.211	109	49	1	70	178	1646033
lcl|GU166	Chr6	52.294	109	50	1	70	178	3040864
lcl|GU166	Chr6	52.294	109	50	1	70	178	3055541
lcl|GU166	Chr6	52.294	109	50	1	70	178	3046894
lcl|GU166	Chr6	52.294	109	50	1	70	178	3056252
lcl|GU166	Chr11	52.294	109	50	1	70	178	2626139
lcl|GU166	Chr1	52.778	108	49	1	70	177	37513978
lcl|GU166	Chr5	52.294	109	50	1	70	178	21500014
lcl|GU166	Chr5	60.976	41	16	0	74	114	24069172

lcl|GU166	Chr5	52	25	12	0	145	169	24068431
lcl|GU166	Chr4	52.294	109	50	1	70	178	20739842
lcl|GU166	Chr4	34.911	169	50	3	70	178	22463224
lcl|GU166	Chr12	53.774	106	47	1	73	178	12808331
lcl|GU166	Chr12	50	106	51	1	73	178	12792775
lcl|GU166	Chr2	39.316	117	41	1	92	178	15225499
lcl|GU166	Chr2	62.857	35	13	0	144	178	15204221
lcl|GU166	Chr3	65.714	35	12	0	144	178	15647702
lcl|GU166	Chr3	44.444	27	15	0	70	96	15647298
lcl|GU166	Chr3	51.852	27	13	0	93	119	15647458

TBLASTN 2.10.1+
Query: lcl|GU166738.1_prot_ACZ04978.1_1 [gene=CENH3] [protein=centromere-specific H3 var
Database: Nippon
Fields: query acc.ver, subject acc.ver, % identity, alignment length, mismatches, gap opens, q. s
19 hits found

lcl|GU166	Chr6	58.333	96	38	1	63	158	1645994
lcl|GU166	Chr6	57.292	96	39	1	63	158	3056291
lcl|GU166	Chr6	57.292	96	39	1	63	158	3046855
lcl|GU166	Chr6	57.292	96	39	1	63	158	3040825
lcl|GU166	Chr6	57.292	96	39	1	63	158	3055502
lcl|GU166	Chr5	57.292	96	39	1	63	158	21499975
lcl|GU166	Chr5	75	32	8	0	63	94	24069145
lcl|GU166	Chr5	50	26	13	0	124	149	24068434
lcl|GU166	Chr11	57.292	96	39	1	63	158	2626100
lcl|GU166	Chr1	57.895	95	38	1	63	157	37514017
lcl|GU166	Chr4	57.292	96	39	1	63	158	20739881
lcl|GU166	Chr4	36.538	156	39	3	63	158	22463263
lcl|GU166	Chr12	57.292	96	39	1	63	158	12808301
lcl|GU166	Chr12	53.125	96	43	1	63	158	12792745
lcl|GU166	Chr2	40.171	117	40	1	72	158	15225499
lcl|GU166	Chr2	65.714	35	12	0	124	158	15204221
lcl|GU166	Chr3	68.571	35	11	0	124	158	15647702
lcl|GU166	Chr3	51.852	27	13	0	73	99	15647458
lcl|GU166	Chr3	64.286	14	5	0	63	76	15647337

TBLASTN 2.10.1+
Query: lcl|MH329772.1_prot_QBB85873.1_1 [gene=CENH3] [protein=centromere specific histo
Database: Nippon
Fields: query acc.ver, subject acc.ver, % identity, alignment length, mismatches, gap opens, q. s
23 hits found

lcl|MH32S	Chr6	53.774	106	47	1	75	180	1646024
lcl|MH32S	Chr6	53.774	106	47	2	75	180	3040855
lcl|MH32S	Chr6	53.774	106	47	2	75	180	3055532
lcl|MH32S	Chr6	52.83	106	48	1	75	180	3046885
lcl|MH32S	Chr6	52.83	106	48	1	75	180	3056261
lcl|MH32S	Chr1	53.333	105	47	1	75	179	37513987
lcl|MH32S	Chr11	53.774	106	47	2	75	180	2626130
lcl|MH32S	Chr11	32.308	65	39	2	107	167	1069460
lcl|MH32S	Chr5	53.774	106	47	2	75	180	21500005

lcl|MH329	Chr5	60.976	41	16	0	76	116	24069172
lcl|MH329	Chr5	55.172	29	12	1	115	143	24068961
lcl|MH329	Chr5	48.387	31	16	0	141	171	24068449
lcl|MH329	Chr4	52.83	106	48	1	75	180	20739851
lcl|MH329	Chr4	34.731	167	47	3	75	180	22463233
lcl|MH329	Chr12	51.376	109	45	2	75	180	12808331
lcl|MH329	Chr12	48.113	106	53	1	75	180	12792775
lcl|MH329	Chr3	58.824	51	21	0	130	180	15647654
lcl|MH329	Chr3	51.724	29	14	0	95	123	15647458
lcl|MH329	Chr3	50	24	12	0	75	98	15647307
lcl|MH329	Chr2	38.983	118	40	2	94	180	15225499
lcl|MH329	Chr2	56.863	51	22	0	130	180	15204269
lcl|MH329	Chr2	53.333	30	14	0	94	123	15204468
lcl|MH329	Chr2	29.688	64	42	1	100	163	24733354

TBLASTN 2.10.1+
Query: lcl|MH329770.1_prot_QBB85871.1_1 [gene=CENH3] [protein=centromere specific histo
Database: Nippon
Fields: query acc.ver, subject acc.ver, % identity, alignment length, mismatches, gap opens, q. s
23 hits found

lcl|MH329	Chr6	51.818	110	51	1	65	174	1646036
lcl|MH329	Chr6	51.818	110	51	2	65	174	3040867
lcl|MH329	Chr6	50.909	110	52	1	65	174	3056249
lcl|MH329	Chr6	51.818	110	51	2	65	174	3055544
lcl|MH329	Chr6	51.376	109	51	1	66	174	3046894
lcl|MH329	Chr1	51.376	109	51	1	65	173	37513975
lcl|MH329	Chr11	51.818	110	51	2	65	174	2626142
lcl|MH329	Chr11	32.308	65	39	2	101	161	1069460
lcl|MH329	Chr5	51.818	110	51	2	65	174	21500017
lcl|MH329	Chr5	60.976	41	16	0	70	110	24069172
lcl|MH329	Chr5	55.172	29	12	1	109	137	24068961
lcl|MH329	Chr5	48.387	31	16	0	135	165	24068449
lcl|MH329	Chr4	50.909	110	52	1	65	174	20739839
lcl|MH329	Chr4	33.918	171	51	3	65	174	22463221
lcl|MH329	Chr12	49.558	113	49	2	65	174	12808343
lcl|MH329	Chr12	46.364	110	57	1	65	174	12792787
lcl|MH329	Chr3	58.824	51	21	0	124	174	15647654
lcl|MH329	Chr3	51.724	29	14	0	89	117	15647458
lcl|MH329	Chr3	46.154	26	14	0	65	90	15647295
lcl|MH329	Chr2	38.983	118	40	2	88	174	15225499
lcl|MH329	Chr2	56.863	51	22	0	124	174	15204269
lcl|MH329	Chr2	53.333	30	14	0	88	117	15204468
lcl|MH329	Chr2	29.688	64	42	1	94	157	24733354

TBLASTN 2.10.1+
Query: lcl|KT932954.1_prot_AOR06535.1_1 [gene=CENH3] [protein=centromere histone H3 var
Database: Nippon
Fields: query acc.ver, subject acc.ver, % identity, alignment length, mismatches, gap opens, q. s
23 hits found

lcl|KT932	Chr6	67	100	32	1	73	172	3040837

lcl|KT9329	Chr6	67	100	32	1	73	172	3055514
lcl|KT9329	Chr6	67	100	32	1	73	172	3056279
lcl|KT9329	Chr6	67	100	32	1	73	172	3046867
lcl|KT9329	Chr6	68	100	31	1	73	172	1646006
lcl|KT9329	Chr11	67	100	32	1	73	172	2626112
lcl|KT9329	Chr5	67	100	32	1	73	172	21499987
lcl|KT9329	Chr5	70.732	41	12	0	68	108	24069172
lcl|KT9329	Chr5	73.333	15	4	0	108	122	24068958
lcl|KT9329	Chr5	61.905	21	8	0	137	157	24068434
lcl|KT9329	Chr1	68.367	98	30	1	73	170	37514005
lcl|KT9329	Chr4	67	100	32	1	73	172	20739869
lcl|KT9329	Chr4	40.994	161	34	2	73	172	22463251
lcl|KT9329	Chr12	63	100	36	1	73	172	12808313
lcl|KT9329	Chr12	60	100	39	1	73	172	12792757
lcl|KT9329	Chr2	46.218	119	33	2	86	173	15225499
lcl|KT9329	Chr2	67.568	37	12	0	137	173	15204221
lcl|KT9329	Chr2	56.667	30	13	0	86	115	15204468
lcl|KT9329	Chr2	40.476	42	25	0	66	107	20134879
lcl|KT9329	Chr2	41.667	36	13	1	137	172	20135070
lcl|KT9329	Chr3	75	36	9	0	137	172	15647702
lcl|KT9329	Chr3	51.429	35	17	0	87	121	15647458
lcl|KT9329	Chr3	68.75	16	5	0	73	88	15647325

TBLASTN 2.10.1+
Query: lcl|KT932953.1_prot_AOR06534.1_1 [gene=CENH3] [protein=centromere histone H3 var
Database: Nippon
Fields: query acc.ver, subject acc.ver, % identity, alignment length, mismatches, gap opens, q. s
23 hits found

lcl|KT9329	Chr6	67	100	32	1	81	180	3040837
lcl|KT9329	Chr6	67	100	32	1	81	180	3055514
lcl|KT9329	Chr6	67	100	32	1	81	180	3056279
lcl|KT9329	Chr6	67	100	32	1	81	180	3046867
lcl|KT9329	Chr6	68	100	31	1	81	180	1646006
lcl|KT9329	Chr11	67	100	32	1	81	180	2626112
lcl|KT9329	Chr5	67	100	32	1	81	180	21499987
lcl|KT9329	Chr5	70.732	41	12	0	76	116	24069172
lcl|KT9329	Chr5	73.333	15	4	0	116	130	24068958
lcl|KT9329	Chr5	61.905	21	8	0	145	165	24068434
lcl|KT9329	Chr1	68.367	98	30	1	81	178	37514005
lcl|KT9329	Chr4	67	100	32	1	81	180	20739869
lcl|KT9329	Chr4	40.994	161	34	2	81	180	22463251
lcl|KT9329	Chr12	63	100	36	1	81	180	12808313
lcl|KT9329	Chr12	60	100	39	1	81	180	12792757
lcl|KT9329	Chr2	46.218	119	33	2	94	181	15225499
lcl|KT9329	Chr2	67.568	37	12	0	145	181	15204221
lcl|KT9329	Chr2	56.667	30	13	0	94	123	15204468
lcl|KT9329	Chr2	40.476	42	25	0	74	115	20134879
lcl|KT9329	Chr2	41.667	36	13	1	145	180	20135070
lcl|KT9329	Chr3	75	36	9	0	145	180	15647702

lcl|KT932	Chr3	51.429	35	17	0	95	129	15647458
lcl|KT932	Chr3	68.75	16	5	0	81	96	15647325

TBLASTN 2.10.1+
Query: lcl|KU837266.1_prot_AOH73624.1_1 [gene=CENH3] [protein=centromere-specific histor
Database: Nippon
Fields: query acc.ver, subject acc.ver, % identity, alignment length, mismatches, gap opens, q. s
22 hits found

lcl|KU837	Chr6	53.211	109	49	1	81	189	1646033
lcl|KU837	Chr6	53.211	109	49	2	81	189	3040864
lcl|KU837	Chr6	52.294	109	50	1	81	189	3056252
lcl|KU837	Chr6	53.211	109	49	2	81	189	3055541
lcl|KU837	Chr6	52.294	109	50	1	81	189	3046894
lcl|KU837	Chr1	52.778	108	49	1	81	188	37513978
lcl|KU837	Chr5	53.211	109	49	2	81	189	21500014
lcl|KU837	Chr5	60.976	41	16	0	85	125	24069172
lcl|KU837	Chr5	51.724	29	13	1	124	152	24068961
lcl|KU837	Chr5	51.613	31	15	0	150	180	24068449
lcl|KU837	Chr11	53.211	109	49	2	81	189	2626139
lcl|KU837	Chr4	52.294	109	50	1	81	189	20739842
lcl|KU837	Chr4	35.329	167	46	3	84	189	22463233
lcl|KU837	Chr12	50.893	112	47	2	81	189	12808340
lcl|KU837	Chr12	49.057	106	52	1	84	189	12792775
lcl|KU837	Chr3	60.784	51	20	0	139	189	15647654
lcl|KU837	Chr3	51.724	29	14	0	104	132	15647458
lcl|KU837	Chr3	48	25	13	0	81	105	15647298
lcl|KU837	Chr2	39.831	118	39	2	103	189	15225499
lcl|KU837	Chr2	58.824	51	21	0	139	189	15204269
lcl|KU837	Chr2	53.333	30	14	0	103	132	15204468
lcl|KU837	Chr2	29.688	64	42	1	109	172	24733354

TBLASTN 2.10.1+
Query: lcl|KU837264.1_prot_AOH73622.1_1 [gene=CENH3] [protein=centromere-specific histor
Database: Nippon
Fields: query acc.ver, subject acc.ver, % identity, alignment length, mismatches, gap opens, q. s
21 hits found

lcl|KU837	Chr6	53.211	109	49	1	67	175	1646033
lcl|KU837	Chr6	53.211	109	49	2	67	175	3040864
lcl|KU837	Chr6	52.294	109	50	1	67	175	3056252
lcl|KU837	Chr6	53.211	109	49	2	67	175	3055541
lcl|KU837	Chr6	52.294	109	50	1	67	175	3046894
lcl|KU837	Chr1	52.778	108	49	1	67	174	37513978
lcl|KU837	Chr11	53.211	109	49	2	67	175	2626139
lcl|KU837	Chr5	53.211	109	49	2	67	175	21500014
lcl|KU837	Chr5	60.976	41	16	0	71	111	24069172
lcl|KU837	Chr5	51.724	29	13	1	110	138	24068961
lcl|KU837	Chr5	51.613	31	15	0	136	166	24068449
lcl|KU837	Chr4	52.294	109	50	1	67	175	20739842
lcl|KU837	Chr4	34.706	170	49	3	67	175	22463224
lcl|KU837	Chr12	52.294	109	44	2	70	175	12808331

lcl|KU837	Chr12	48.624	109	48	2	70	175	12792775
lcl|KU837	Chr3	60.784	51	20	0	125	175	15647654
lcl|KU837	Chr3	51.724	29	14	0	90	118	15647458
lcl|KU837	Chr3	44.444	27	15	0	67	93	15647298
lcl|KU837	Chr2	39.831	118	39	2	89	175	15225499
lcl|KU837	Chr2	58.824	51	21	0	125	175	15204269
lcl|KU837	Chr2	53.333	30	14	0	89	118	15204468

TBLASTN 2.10.1+
Query: lcl|KU837263.1_prot_AOH73621.1_1 [gene=CENH3] [protein=centromere-specific histor
Database: Nippon
Fields: query acc.ver, subject acc.ver, % identity, alignment length, mismatches, gap opens, q. s
19 hits found

lcl|KU837	Chr6	51.786	112	52	1	67	178	1646042
lcl|KU837	Chr6	52.101	119	52	2	63	178	3040894
lcl|KU837	Chr6	51.786	112	52	1	67	178	3046903
lcl|KU837	Chr6	52.101	119	52	2	63	178	3055571
lcl|KU837	Chr6	52.101	119	52	2	63	178	3056222
lcl|KU837	Chr5	52.101	119	52	2	63	178	21500044
lcl|KU837	Chr5	60.976	41	16	0	74	114	24069172
lcl|KU837	Chr5	56	25	11	0	145	169	24068431
lcl|KU837	Chr11	52.101	119	52	2	63	178	2626169
lcl|KU837	Chr1	52.542	118	51	2	63	177	37513948
lcl|KU837	Chr4	52.101	119	52	2	63	178	20739812
lcl|KU837	Chr4	34.884	172	52	3	67	178	22463215
lcl|KU837	Chr12	51.818	110	51	1	69	178	12808343
lcl|KU837	Chr12	48.182	110	55	1	69	178	12792787
lcl|KU837	Chr2	39.316	117	41	1	92	178	15225499
lcl|KU837	Chr2	62.857	35	13	0	144	178	15204221
lcl|KU837	Chr3	65.714	35	12	0	144	178	15647702
lcl|KU837	Chr3	42.857	28	16	0	69	96	15647295
lcl|KU837	Chr3	51.852	27	13	0	93	119	15647458

TBLASTN 2.10.1+
Query: lcl|KU837262.1_prot_AOH73620.1_1 [gene=CENH3] [protein=centromere-specific histor
Database: Nippon
Fields: query acc.ver, subject acc.ver, % identity, alignment length, mismatches, gap opens, q. s
19 hits found

lcl|KU837	Chr11	56.25	96	40	1	62	157	2626100
lcl|KU837	Chr6	57.292	96	39	1	62	157	1645994
lcl|KU837	Chr6	56.25	96	40	1	62	157	3056291
lcl|KU837	Chr6	56.25	96	40	1	62	157	3040825
lcl|KU837	Chr6	56.25	96	40	1	62	157	3055502
lcl|KU837	Chr6	56.25	96	40	1	62	157	3046855
lcl|KU837	Chr1	56.842	95	39	1	62	156	37514017
lcl|KU837	Chr5	56.25	96	40	1	62	157	21499975
lcl|KU837	Chr5	75	32	8	0	62	93	24069145
lcl|KU837	Chr5	46.154	26	14	0	123	148	24068434
lcl|KU837	Chr4	56.25	96	40	1	62	157	20739881
lcl|KU837	Chr4	35.897	156	40	3	62	157	22463263

lcl|KU837	Chr12	57.292	96	39	1	62	157	12808301
lcl|KU837	Chr12	53.125	96	43	1	62	157	12792745
lcl|KU837	Chr2	39.316	117	41	1	71	157	15225499
lcl|KU837	Chr2	62.857	35	13	0	123	157	15204221
lcl|KU837	Chr3	65.714	35	12	0	123	157	15647702
lcl|KU837	Chr3	51.852	27	13	0	72	98	15647458
lcl|KU837	Chr3	64.286	14	5	0	62	75	15647337

TBLASTN 2.10.1+
Query: lcl|KU674827.1_prot_AOH73617.1_1 [gene=CENH3] [protein=centromere-specific histor
Database: Nippon
Fields: query acc.ver, subject acc.ver, % identity, alignment length, mismatches, gap opens, q. s
20 hits found

lcl|KU674	Chr6	57	100	41	1	81	180	3040837
lcl|KU674	Chr6	58	100	40	1	81	180	1646006
lcl|KU674	Chr6	57	100	41	1	81	180	3056279
lcl|KU674	Chr6	57	100	41	1	81	180	3055514
lcl|KU674	Chr6	57	100	41	1	81	180	3046867
lcl|KU674	Chr5	57	100	41	1	81	180	21499987
lcl|KU674	Chr5	62.5	40	15	0	77	116	24069169
lcl|KU674	Chr5	52	25	12	0	147	171	24068431
lcl|KU674	Chr11	57	100	41	1	81	180	2626112
lcl|KU674	Chr4	57	100	41	1	81	180	20739869
lcl|KU674	Chr4	36.646	161	40	4	81	180	22463251
lcl|KU674	Chr1	57.576	99	40	1	81	179	37514005
lcl|KU674	Chr12	57	100	41	1	81	180	12808313
lcl|KU674	Chr12	53	100	45	1	81	180	12792757
lcl|KU674	Chr2	40.833	120	35	2	94	180	15225499
lcl|KU674	Chr2	60	35	14	0	146	180	15204221
lcl|KU674	Chr2	60	30	12	0	94	123	15204468
lcl|KU674	Chr3	62.857	35	13	0	146	180	15647702
lcl|KU674	Chr3	55.172	29	13	0	95	123	15647458
lcl|KU674	Chr3	61.111	18	7	0	81	98	15647325

TBLASTN 2.10.1+
Query: lcl|KR029619.1_prot_ALF04640.1_1 [gene=CENH3] [protein=centromeric histone H3 vari
Database: Nippon
Fields: query acc.ver, subject acc.ver, % identity, alignment length, mismatches, gap opens, q. s
25 hits found

lcl|KR029	Chr11	59.2	125	41	2	54	176	2626166
lcl|KR029	Chr6	59.2	125	41	2	54	176	3040891
lcl|KR029	Chr6	59.2	125	41	2	54	176	3055568
lcl|KR029	Chr6	65.347	101	35	0	76	176	3056273
lcl|KR029	Chr6	65.347	101	35	0	76	176	3046873
lcl|KR029	Chr6	65.385	104	36	0	73	176	1646021
lcl|KR029	Chr5	59.2	125	41	2	54	176	21500041
lcl|KR029	Chr5	75.61	41	10	0	73	113	24069172
lcl|KR029	Chr5	78.947	19	4	0	143	161	24068428
lcl|KR029	Chr4	65.347	101	35	0	76	176	20739863
lcl|KR029	Chr4	40.659	182	36	4	57	176	22463218

lcl|KR029(	Chr1	65.657	99	34	0	76	174	37513999
lcl|KR029(	Chr1	41.935	31	17	1	149	178	19908803
lcl|KR029(	Chr12	48.921	139	54	4	47	178	12808391
lcl|KR029(	Chr12	54.902	102	46	0	75	176	12792766
lcl|KR029(	Chr3	65.517	58	20	0	119	176	15647636
lcl|KR029(	Chr3	58.621	29	12	0	92	120	15647458
lcl|KR029(	Chr3	61.905	21	8	0	73	93	15647310
lcl|KR029(	Chr2	47.059	119	31	1	91	177	15225499
lcl|KR029(	Chr2	67.273	55	18	0	123	177	15204275
lcl|KR029(	Chr2	60	30	12	0	91	120	15204468
lcl|KR029(	Chr2	44.186	43	24	0	70	112	20134876
lcl|KR029(	Chr2	50	34	9	1	143	176	20135076
lcl|KR029(	Chr2	35.556	45	27	1	63	105	28996632
lcl|KR029(	Chr2	47.5	40	11	2	54	93	15225811

TBLASTN 2.10.1+
Query: lcl|KR029618.1_prot_ALF04639.1_1 [gene=CENH3] [protein=centromeric histone H3 vari
Database: Nippon
Fields: query acc.ver, subject acc.ver, % identity, alignment length, mismatches, gap opens, q. s
25 hits found

lcl|KR029(	Chr6	64.356	101	36	0	76	176	3040843
lcl|KR029(	Chr6	62.5	104	39	0	73	176	3055529
lcl|KR029(	Chr6	64.356	101	36	0	76	176	3056273
lcl|KR029(	Chr6	64.356	101	36	0	76	176	3046873
lcl|KR029(	Chr6	66.337	101	34	0	76	176	1646012
lcl|KR029(	Chr11	64.356	101	36	0	76	176	2626118
lcl|KR029(	Chr5	64.356	101	36	0	76	176	21499993
lcl|KR029(	Chr5	76.19	42	10	0	72	113	24069175
lcl|KR029(	Chr5	73.684	19	5	0	143	161	24068428
lcl|KR029(	Chr4	64.356	101	36	0	76	176	20739863
lcl|KR029(	Chr4	40.659	182	36	4	57	176	22463218
lcl|KR029(	Chr1	64.646	99	35	0	76	174	37513999
lcl|KR029(	Chr1	41.935	31	17	1	149	178	19908803
lcl|KR029(	Chr12	51.2	125	49	3	56	178	12808349
lcl|KR029(	Chr12	53.922	102	47	0	75	176	12792766
lcl|KR029(	Chr3	63.793	58	21	0	119	176	15647636
lcl|KR029(	Chr3	58.621	29	12	0	92	120	15647458
lcl|KR029(	Chr3	61.905	21	8	0	73	93	15647310
lcl|KR029(	Chr2	46.218	119	32	1	91	177	15225499
lcl|KR029(	Chr2	65.455	55	19	0	123	177	15204275
lcl|KR029(	Chr2	60	30	12	0	91	120	15204468
lcl|KR029(	Chr2	44.186	43	24	0	70	112	20134876
lcl|KR029(	Chr2	47.059	34	10	1	143	176	20135076
lcl|KR029(	Chr2	35.556	45	27	1	63	105	28996632
lcl|KR029(	Chr2	68.421	19	6	0	75	93	15225778

TBLASTN 2.10.1+
Query: lcl|KF214777.1_prot_AHH01567.1_1 [gene=CenH3] [protein=centromere-specific histone
Database: Nippon
Fields: query acc.ver, subject acc.ver, % identity, alignment length, mismatches, gap opens, q. s

20 hits found

lcl|KF2147	Chr6	65.306	98	33	1	57	154	3040837
lcl|KF2147	Chr6	65.306	98	33	1	57	154	3056279
lcl|KF2147	Chr6	65.306	98	33	1	57	154	3055514
lcl|KF2147	Chr6	65.306	98	33	1	57	154	3046867
lcl|KF2147	Chr6	65.306	98	33	1	57	154	1646006
lcl|KF2147	Chr11	65.306	98	33	1	57	154	2626112
lcl|KF2147	Chr5	65.306	98	33	1	57	154	21499987
lcl|KF2147	Chr5	66.667	39	13	0	54	92	24069166
lcl|KF2147	Chr5	65.217	23	8	0	121	143	24068434
lcl|KF2147	Chr4	65.306	98	33	1	57	154	20739869
lcl|KF2147	Chr4	40.881	159	33	2	57	154	22463251
lcl|KF2147	Chr1	65.306	98	33	1	57	154	37514005
lcl|KF2147	Chr12	63.265	98	35	1	57	154	12808313
lcl|KF2147	Chr12	58.163	98	40	1	57	154	12792757
lcl|KF2147	Chr2	45.69	116	32	2	70	154	15225499
lcl|KF2147	Chr2	70.588	34	10	0	121	154	15204221
lcl|KF2147	Chr2	52.778	36	15	1	70	105	15204468
lcl|KF2147	Chr3	76.471	34	8	0	121	154	15647702
lcl|KF2147	Chr3	51.429	35	17	0	71	105	15647458
lcl|KF2147	Chr3	68.75	16	5	0	57	72	15647325

TBLASTN 2.10.1+
Query: lcl|KC491791.1_prot_AGQ21573.1_1 [gene=CENH3] [protein=centromere specific histon
Database: Nippon
Fields: query acc.ver, subject acc.ver, % identity, alignment length, mismatches, gap opens, q. s
20 hits found

lcl|KC4917	Chr6	60.204	98	38	1	61	158	3040837
lcl|KC4917	Chr6	60.204	98	38	1	61	158	3056279
lcl|KC4917	Chr6	60.204	98	38	1	61	158	3055514
lcl|KC4917	Chr6	60.204	98	38	1	61	158	3046867
lcl|KC4917	Chr6	60.204	98	38	1	61	158	1646006
lcl|KC4917	Chr11	60.204	98	38	1	61	158	2626112
lcl|KC4917	Chr5	60.204	98	38	1	61	158	21499987
lcl|KC4917	Chr5	61.538	39	15	0	58	96	24069166
lcl|KC4917	Chr5	62.5	16	6	0	96	111	24068958
lcl|KC4917	Chr1	60.204	98	38	1	61	158	37514005
lcl|KC4917	Chr4	60.204	98	38	1	61	158	20739869
lcl|KC4917	Chr4	44.348	115	33	1	75	158	22463383
lcl|KC4917	Chr12	59.184	98	39	1	61	158	12808313
lcl|KC4917	Chr12	55.102	98	43	1	61	158	12792757
lcl|KC4917	Chr2	42.241	116	36	1	74	158	15225499
lcl|KC4917	Chr2	70	30	9	0	129	158	15204209
lcl|KC4917	Chr2	50	32	16	0	74	105	15204468
lcl|KC4917	Chr3	76.667	30	7	0	129	158	15647714
lcl|KC4917	Chr3	48.571	35	18	0	75	109	15647458
lcl|KC4917	Chr3	52.174	23	11	0	66	88	29457978

TBLASTN 2.10.1+
Query: lcl|AY438639.1_prot_AAR85315.1_1 [gene=CenH3] [protein=centromeric histone 3] [prc

Database: Nippon
Fields: query acc.ver, subject acc.ver, % identity, alignment length, mismatches, gap opens, q. s
26 hits found

lcl|AY438(	Chr6	69	100	30	1	63	162	3040837
lcl|AY438(	Chr6	69	100	30	1	63	162	3055514
lcl|AY438(	Chr6	69	100	30	1	63	162	3056279
lcl|AY438(	Chr6	69	100	30	1	63	162	3046867
lcl|AY438(	Chr6	69	100	30	1	63	162	1646006
lcl|AY438(	Chr11	69	100	30	1	63	162	2626112
lcl|AY438(	Chr5	69	100	30	1	63	162	21499987
lcl|AY438(	Chr5	100	43	0	0	56	98	24069178
lcl|AY438(	Chr5	100	16	0	0	98	113	24068958
lcl|AY438(	Chr5	100	32	0	0	1	32	24070126
lcl|AY438(	Chr5	100	22	0	0	126	147	24068437
lcl|AY438(	Chr5	62.069	29	4	1	136	164	24068029
lcl|AY438(	Chr4	69	100	30	1	63	162	20739869
lcl|AY438(	Chr4	41.615	161	33	2	63	162	22463251
lcl|AY438(	Chr1	69.388	98	29	1	63	160	37514005
lcl|AY438(	Chr12	62.136	103	32	2	63	162	12808313
lcl|AY438(	Chr12	62	100	37	1	63	162	12792757
lcl|AY438(	Chr2	47.458	118	31	1	76	162	15225499
lcl|AY438(	Chr2	72.222	36	10	0	127	162	15204221
lcl|AY438(	Chr2	66.667	30	10	0	76	105	15204468
lcl|AY438(	Chr2	83.333	18	3	0	63	80	20134900
lcl|AY438(	Chr2	75	16	4	0	63	78	15225769
lcl|AY438(	Chr3	72.973	37	10	0	127	163	15647702
lcl|AY438(	Chr3	57.143	35	15	0	77	111	15647458
lcl|AY438(	Chr3	75	16	4	0	63	78	15647325
lcl|AY438(	Chr7	60	20	8	0	92	111	11774928

TBLASTN 2.10.1+
Query: lcl|AF519807.2_prot_AAM74226.1_1 [protein=centromeric histone H3-like protein] [pro
Database: Nippon
Fields: query acc.ver, subject acc.ver, % identity, alignment length, mismatches, gap opens, q. s
22 hits found

lcl|AF5198	Chr6	67.677	99	28	2	58	155	3040828
lcl|AF5198	Chr6	67.677	99	28	2	58	155	3055505
lcl|AF5198	Chr6	67.677	99	28	2	58	155	3046858
lcl|AF5198	Chr6	67.677	99	28	2	58	155	3056288
lcl|AF5198	Chr6	68.687	99	27	2	58	155	1645997
lcl|AF5198	Chr11	67.677	99	28	2	58	155	2626103
lcl|AF5198	Chr5	67.677	99	28	2	58	155	21499978
lcl|AF5198	Chr5	71.429	42	11	1	49	90	24069172
lcl|AF5198	Chr5	72.727	22	6	0	119	140	24068437
lcl|AF5198	Chr5	80	25	5	0	1	25	24070126
lcl|AF5198	Chr4	67.677	99	28	2	58	155	20739878
lcl|AF5198	Chr4	39.752	161	37	3	55	155	22463251
lcl|AF5198	Chr1	68.041	97	27	2	58	153	37514014
lcl|AF5198	Chr12	61.224	98	36	1	58	155	12808304

lcl|AF5198	Chr12	59.184	98	38	1	58	155	12792748
lcl|AF5198	Chr2	46.61	118	33	1	68	155	15225499
lcl|AF5198	Chr2	70.27	37	11	0	120	156	15204221
lcl|AF5198	Chr2	69.231	26	8	0	68	93	15204468
lcl|AF5198	Chr2	65.217	23	8	0	50	72	20134885
lcl|AF5198	Chr3	77.778	36	8	0	120	155	15647702
lcl|AF5198	Chr3	72	25	7	0	69	93	15647458
lcl|AF5198	Chr3	76.923	13	3	0	58	70	15647334

TBLASTN 2.10.1+
Query: lcl|HM988988.1_prot_ADM18965.1_1 [gene=CENH3-B] [protein=centromeric histone H3
Database: Nippon
Fields: query acc.ver, subject acc.ver, % identity, alignment length, mismatches, gap opens, q. s
22 hits found

lcl|HM988	Chr6	63.542	96	34	1	80	175	3040837
lcl|HM988	Chr6	63.542	96	34	1	80	175	3056279
lcl|HM988	Chr6	63.542	96	34	1	80	175	3055514
lcl|HM988	Chr6	51.493	134	61	3	43	175	3046975
lcl|HM988	Chr6	64.583	96	33	1	80	175	1646006
lcl|HM988	Chr1	63.542	96	34	1	80	175	37514005
lcl|HM988	Chr11	60	110	42	2	67	175	2626154
lcl|HM988	Chr5	63.542	96	34	1	80	175	21499987
lcl|HM988	Chr5	70	40	12	0	76	115	24069169
lcl|HM988	Chr5	73.333	15	4	0	115	129	24068958
lcl|HM988	Chr5	59.091	22	9	0	143	164	24068437
lcl|HM988	Chr4	63.542	96	34	1	80	175	20739869
lcl|HM988	Chr4	39.881	168	37	5	71	175	22463221
lcl|HM988	Chr12	61.458	96	36	1	80	175	12808313
lcl|HM988	Chr12	57.143	98	41	1	80	177	12792757
lcl|HM988	Chr2	45.614	114	31	2	93	175	15225499
lcl|HM988	Chr2	71.875	32	9	0	144	175	15204221
lcl|HM988	Chr2	54.054	37	15	1	93	127	15204468
lcl|HM988	Chr2	42.857	35	20	0	80	114	20134900
lcl|HM988	Chr3	78.125	32	7	0	144	175	15647702
lcl|HM988	Chr3	60	25	10	0	94	118	15647458
lcl|HM988	Chr3	41.463	41	18	2	80	115	15647325

TBLASTN 2.10.1+
Query: lcl|MH094618.1_prot_AYA72175.1_1 [protein=centromeric-specific histone H3 variant] |
Database: Nippon
Fields: query acc.ver, subject acc.ver, % identity, alignment length, mismatches, gap opens, q. s
23 hits found

lcl|MH094	Chr11	51.282	156	50	3	1	151	2626220
lcl|MH094	Chr6	51.282	156	50	3	1	151	3040945
lcl|MH094	Chr6	51.282	156	50	3	1	151	3055622
lcl|MH094	Chr6	51.282	156	50	3	1	151	3056171
lcl|MH094	Chr6	64.706	102	35	1	50	151	3046873
lcl|MH094	Chr6	64.706	102	35	1	50	151	1646012
lcl|MH094	Chr5	51.282	156	50	3	1	151	21500095
lcl|MH094	Chr5	77.273	44	10	0	44	87	24069181

lcl|MH094	Chr5	70.37	27	8	0	87	113	24068958
lcl|MH094	Chr5	57.692	26	11	0	111	136	24068449
lcl|MH094	Chr4	51.282	156	50	3	1	151	20739761
lcl|MH094	Chr4	40.491	163	36	2	50	151	22463245
lcl|MH094	Chr1	51.299	154	49	3	1	149	37513897
lcl|MH094	Chr12	48.993	149	60	3	1	149	12808421
lcl|MH094	Chr12	49.007	151	61	4	1	151	12792865
lcl|MH094	Chr2	45.763	118	33	1	65	151	15225499
lcl|MH094	Chr2	61.538	52	20	0	100	151	15204269
lcl|MH094	Chr2	80.769	26	5	0	65	90	15204468
lcl|MH094	Chr2	66.667	27	6	1	46	69	20134873
lcl|MH094	Chr2	38.776	49	20	2	108	154	20135040
lcl|MH094	Chr3	65.385	52	18	0	100	151	15647654
lcl|MH094	Chr3	57.143	35	15	0	66	100	15647458
lcl|MH094	Chr3	66.667	18	6	0	50	67	15647319

TBLASTN 2.10.1+
Query: lcl|MH182682.1_prot_AYA72192.1_1 [protein=centromeric-specific histone H3 variant] |
Database: Nippon
Fields: query acc.ver, subject acc.ver, % identity, alignment length, mismatches, gap opens, q. s
24 hits found

lcl|MH182	Chr6	49.359	156	53	3	1	151	3040945
lcl|MH182	Chr6	49.359	156	53	3	1	151	3056171
lcl|MH182	Chr6	49.359	156	53	3	1	151	3055622
lcl|MH182	Chr6	63.725	102	36	1	50	151	3046873
lcl|MH182	Chr6	59.483	116	40	2	36	151	1646036
lcl|MH182	Chr11	49.359	156	53	3	1	151	2626220
lcl|MH182	Chr5	49.359	156	53	3	1	151	21500095
lcl|MH182	Chr5	68.182	44	14	0	44	87	24069181
lcl|MH182	Chr5	70.37	27	8	0	87	113	24068958
lcl|MH182	Chr5	57.692	26	11	0	111	136	24068449
lcl|MH182	Chr4	49.359	156	53	3	1	151	20739761
lcl|MH182	Chr4	39.548	177	40	3	36	151	22463221
lcl|MH182	Chr1	49.351	154	52	3	1	149	37513897
lcl|MH182	Chr12	49.032	155	55	4	1	151	12808421
lcl|MH182	Chr12	57.843	102	42	1	50	151	12792763
lcl|MH182	Chr2	46.61	118	32	2	65	151	15225499
lcl|MH182	Chr2	61.538	52	20	0	100	151	15204269
lcl|MH182	Chr2	76.923	26	6	0	65	90	15204468
lcl|MH182	Chr2	68.421	19	6	0	51	69	20134897
lcl|MH182	Chr2	36.735	49	21	2	108	154	20135040
lcl|MH182	Chr2	66.667	18	6	0	50	67	15225775
lcl|MH182	Chr3	65.385	52	18	0	100	151	15647654
lcl|MH182	Chr3	76	25	6	0	66	90	15647458
lcl|MH182	Chr3	66.667	18	6	0	50	67	15647319

TBLASTN 2.10.1+
Query: lcl|MH153697.1_prot_AYA72176.1_1 [protein=centromeric-specific histone H3 variant] |
Database: Nippon
Fields: query acc.ver, subject acc.ver, % identity, alignment length, mismatches, gap opens, q. s

25 hits found

lcl|MH153	Chr11	54.305	151	53	4	1	151	2626220
lcl|MH153	Chr6	54.305	151	53	4	1	151	3040945
lcl|MH153	Chr6	54.305	151	53	4	1	151	3055622
lcl|MH153	Chr6	54.305	151	53	4	1	151	3056171
lcl|MH153	Chr6	55.944	143	55	4	12	151	3046984
lcl|MH153	Chr6	63.636	110	35	2	42	151	1646024
lcl|MH153	Chr6	27.5	40	29	0	66	105	3153949
lcl|MH153	Chr5	54.305	151	53	4	1	151	21500095
lcl|MH153	Chr5	71.111	45	13	0	43	87	24069184
lcl|MH153	Chr5	62.963	27	10	0	87	113	24068958
lcl|MH153	Chr5	65.385	26	9	0	111	136	24068449
lcl|MH153	Chr4	54.305	151	53	4	1	151	20739761
lcl|MH153	Chr4	40.341	176	40	3	37	151	22463218
lcl|MH153	Chr1	55.034	149	51	4	1	149	37513897
lcl|MH153	Chr12	48.344	151	62	3	1	151	12808421
lcl|MH153	Chr12	46.154	156	58	3	1	151	12792865
lcl|MH153	Chr2	44.068	118	35	1	65	151	15225499
lcl|MH153	Chr2	61.538	52	20	0	100	151	15204269
lcl|MH153	Chr2	76	25	6	0	65	89	15204468
lcl|MH153	Chr2	33.721	86	47	2	45	128	20134879
lcl|MH153	Chr2	72.222	18	5	0	50	67	15225775
lcl|MH153	Chr2	35.294	51	23	2	106	154	20135034
lcl|MH153	Chr3	65.385	52	18	0	100	151	15647654
lcl|MH153	Chr3	79.167	24	5	0	66	89	15647458
lcl|MH153	Chr3	61.538	26	6	1	42	67	15647307

TBLASTN 2.10.1+
Query: lcl|MG384788.1_prot_AUN88474.1_1 [gene=betaCENH3] [protein=centromeric histone
Database: Nippon
Fields: query acc.ver, subject acc.ver, % identity, alignment length, mismatches, gap opens, q. s
25 hits found

lcl|MG384	Chr11	65.306	98	32	1	52	149	2626103
lcl|MG384	Chr6	65.306	98	32	1	52	149	3040828
lcl|MG384	Chr6	65.306	98	32	1	52	149	3055505
lcl|MG384	Chr6	65.306	98	32	1	52	149	3046858
lcl|MG384	Chr6	64	100	34	1	50	149	3056282
lcl|MG384	Chr6	64.286	98	33	1	52	149	1645997
lcl|MG384	Chr5	64	100	34	1	50	149	21499984
lcl|MG384	Chr5	80	35	7	0	50	84	24069154
lcl|MG384	Chr5	42.857	28	15	1	84	111	24068958
lcl|MG384	Chr5	78.571	28	6	0	107	134	24068455
lcl|MG384	Chr5	55.172	29	6	1	123	151	24068029
lcl|MG384	Chr4	64	100	34	1	50	149	20739872
lcl|MG384	Chr4	40.881	159	32	4	52	149	22463260
lcl|MG384	Chr4	36.364	33	21	0	64	96	19539683
lcl|MG384	Chr1	64.286	98	33	1	50	147	37514008
lcl|MG384	Chr12	60	100	38	1	50	149	12808310
lcl|MG384	Chr12	59	100	39	1	50	149	12792754

lcl|MG384	Chr2	47.059	119	33	4	62	150	15225499
lcl|MG384	Chr2	60	60	21	1	94	150	15204290
lcl|MG384	Chr2	73.077	26	7	0	62	87	15204468
lcl|MG384	Chr2	80	15	3	0	52	66	20134909
lcl|MG384	Chr2	37.5	48	20	2	104	149	20135034
lcl|MG384	Chr3	66.667	51	17	0	99	149	15647657
lcl|MG384	Chr3	65.625	32	11	0	63	94	15647458
lcl|MG384	Chr3	60	15	6	0	50	64	15647328

TBLASTN 2.10.1+
Query: lcl|MG384783.1_prot_AUN88469.1_1 [gene=betaCENH3] [protein=beta centromeric his
Database: Nippon
Fields: query acc.ver, subject acc.ver, % identity, alignment length, mismatches, gap opens, q. s
25 hits found

lcl|MG384	Chr6	66.327	98	31	2	52	149	3040828
lcl|MG384	Chr6	66.327	98	31	2	52	149	3055505
lcl|MG384	Chr6	65	100	33	2	50	149	3056282
lcl|MG384	Chr6	66.327	98	31	2	52	149	3046858
lcl|MG384	Chr6	65.306	98	32	2	52	149	1645997
lcl|MG384	Chr11	66.327	98	31	2	52	149	2626103
lcl|MG384	Chr5	65	100	33	2	50	149	21499984
lcl|MG384	Chr5	80	35	7	0	50	84	24069154
lcl|MG384	Chr5	42.857	28	15	1	84	111	24068958
lcl|MG384	Chr5	78.571	28	6	0	107	134	24068455
lcl|MG384	Chr5	73.684	19	5	0	133	151	24068020
lcl|MG384	Chr4	65	100	33	2	50	149	20739872
lcl|MG384	Chr4	41.509	159	31	4	52	149	22463260
lcl|MG384	Chr4	33.333	33	22	0	64	96	19539683
lcl|MG384	Chr1	65.306	98	32	2	50	147	37514008
lcl|MG384	Chr12	60	100	38	1	50	149	12808310
lcl|MG384	Chr12	59	100	39	1	50	149	12792754
lcl|MG384	Chr2	45.833	120	33	3	62	150	15225499
lcl|MG384	Chr2	58.333	60	22	1	94	150	15204290
lcl|MG384	Chr2	73.077	26	7	0	62	87	15204468
lcl|MG384	Chr2	80	15	3	0	52	66	20134909
lcl|MG384	Chr2	37.5	48	20	2	104	149	20135034
lcl|MG384	Chr3	66.667	51	17	0	99	149	15647657
lcl|MG384	Chr3	65.625	32	11	0	63	94	15647458
lcl|MG384	Chr3	60	15	6	0	50	64	15647328

TBLASTN 2.10.1+
Query: lcl|MG384777.1_prot_AUN88463.1_1 [gene=betaCENH3-2] [protein=beta centromeric h
Database: Nippon
Fields: query acc.ver, subject acc.ver, % identity, alignment length, mismatches, gap opens, q. s
24 hits found

lcl|MG384	Chr11	66.327	98	31	2	54	151	2626103
lcl|MG384	Chr6	66.327	98	31	2	54	151	3040828
lcl|MG384	Chr6	66.327	98	31	2	54	151	3055505
lcl|MG384	Chr6	65	100	33	2	52	151	3056282
lcl|MG384	Chr6	65	100	33	2	52	151	3046864

lcl|MG384	Chr6	65.306	98	32	2	54	151	1645997
lcl|MG384	Chr5	65	100	33	2	52	151	21499984
lcl|MG384	Chr5	74.286	35	9	0	52	86	24069154
lcl|MG384	Chr5	46.429	28	14	1	86	113	24068958
lcl|MG384	Chr5	74.194	31	8	0	106	136	24068464
lcl|MG384	Chr5	55.172	29	6	1	125	153	24068029
lcl|MG384	Chr4	65	100	33	2	52	151	20739872
lcl|MG384	Chr4	40.373	161	34	4	52	151	22463254
lcl|MG384	Chr1	65.306	98	32	2	52	149	37514008
lcl|MG384	Chr12	58	100	40	1	52	151	12808310
lcl|MG384	Chr12	57	100	41	1	52	151	12792754
lcl|MG384	Chr2	45.833	120	33	3	64	152	15225499
lcl|MG384	Chr2	52.113	71	28	1	82	152	15204305
lcl|MG384	Chr2	65.385	26	9	0	64	89	15204468
lcl|MG384	Chr2	80	15	3	0	54	68	20134909
lcl|MG384	Chr2	41.304	46	17	2	108	151	20135040
lcl|MG384	Chr3	68.627	51	16	0	101	151	15647657
lcl|MG384	Chr3	59.375	32	13	0	65	96	15647458
lcl|MG384	Chr3	66.667	15	5	0	52	66	15647328

TBLASTN 2.10.1+
Query: lcl|KR676382.1_prot_ALK04343.1_1 [protein=centromeric histone H3 protein] [protein_i
Database: Nippon
Fields: query acc.ver, subject acc.ver, % identity, alignment length, mismatches, gap opens, q. s
21 hits found

lcl|KR676:	Chr11	48.529	136	49	3	44	172	2626184
lcl|KR676:	Chr6	48.529	136	49	3	44	172	3040909
lcl|KR676:	Chr6	48.529	136	49	3	44	172	3055586
lcl|KR676:	Chr6	49.219	128	63	1	45	172	3046951
lcl|KR676:	Chr6	47.482	139	52	3	41	172	3056198
lcl|KR676:	Chr6	55.963	109	46	1	64	172	1646033
lcl|KR676:	Chr5	48.529	136	49	3	44	172	21500059
lcl|KR676:	Chr5	65	40	14	0	69	108	24069169
lcl|KR676:	Chr5	56	25	11	0	139	163	24068431
lcl|KR676:	Chr1	47.826	138	51	3	41	171	37513924
lcl|KR676:	Chr4	47.482	139	52	3	41	172	20739788
lcl|KR676:	Chr4	37.87	169	45	4	64	172	22463224
lcl|KR676:	Chr12	55.046	109	47	1	64	172	12808340
lcl|KR676:	Chr12	51.376	109	51	1	64	172	12792784
lcl|KR676:	Chr2	42.735	117	37	2	86	172	15225499
lcl|KR676:	Chr2	65.714	35	12	0	138	172	15204221
lcl|KR676:	Chr2	41.667	36	21	0	54	89	20134843
lcl|KR676:	Chr2	55.172	29	13	0	86	114	15204468
lcl|KR676:	Chr2	75	16	4	0	73	88	15225769
lcl|KR676:	Chr3	68.571	35	11	0	138	172	15647702
lcl|KR676:	Chr3	38.776	49	23	1	64	105	15647298

TBLASTN 2.10.1+
Query: lcl|KR676380.1_prot_ALK04341.1_1 [protein=centromeric histone H3 protein] [protein_i
Database: Nippon

Fields: query acc.ver, subject acc.ver, % identity, alignment length, mismatches, gap opens, q. s
21 hits found

lcl|KR676:	Chr1	56.566	99	41	1	71	169	37514005
lcl|KR676:	Chr6	57	100	41	1	71	170	1646006
lcl|KR676:	Chr6	56	100	42	1	71	170	3056279
lcl|KR676:	Chr6	56	100	42	1	71	170	3040837
lcl|KR676:	Chr6	56	100	42	1	71	170	3055514
lcl|KR676:	Chr6	56	100	42	1	71	170	3046867
lcl|KR676:	Chr12	57	100	41	1	71	170	12808313
lcl|KR676:	Chr12	52	100	46	1	71	170	12792757
lcl|KR676:	Chr4	56	100	42	1	71	170	20739869
lcl|KR676:	Chr4	37.5	160	40	3	71	170	22463251
lcl|KR676:	Chr5	56	100	42	1	71	170	21499987
lcl|KR676:	Chr5	67.5	40	13	0	67	106	24069169
lcl|KR676:	Chr5	48.276	29	14	1	105	133	24068961
lcl|KR676:	Chr5	54.839	31	14	0	131	161	24068449
lcl|KR676:	Chr11	56	100	42	1	71	170	2626112
lcl|KR676:	Chr3	62.745	51	19	0	120	170	15647654
lcl|KR676:	Chr3	56	25	11	0	85	109	15647458
lcl|KR676:	Chr3	61.111	18	7	0	71	88	15647325
lcl|KR676:	Chr2	60.784	51	20	0	120	170	15204269
lcl|KR676:	Chr2	57.692	26	11	0	84	109	15204468
lcl|KR676:	Chr2	40.171	117	40	2	84	170	15225499

TBLASTN 2.10.1+
Query: lcl|AB793504.1_prot_BAO51832.1_1 [gene=TbCENH3] [protein=centromere specific hist
Database: Nippon
Fields: query acc.ver, subject acc.ver, % identity, alignment length, mismatches, gap opens, q. s
22 hits found

lcl|AB793	Chr11	54.73	148	53	4	1	148	2626220
lcl|AB793	Chr6	54.73	148	53	4	1	148	3055622
lcl|AB793	Chr6	54.73	148	53	4	1	148	3040945
lcl|AB793	Chr6	54.73	148	53	4	1	148	3056171
lcl|AB793	Chr6	58.929	112	45	1	37	148	3046906
lcl|AB793	Chr6	60.185	108	42	1	41	148	1646033
lcl|AB793	Chr5	54.73	148	53	4	1	148	21500095
lcl|AB793	Chr5	70.27	37	11	0	49	85	24069160
lcl|AB793	Chr5	53.846	26	12	0	114	139	24068434
lcl|AB793	Chr4	54.73	148	53	4	1	148	20739761
lcl|AB793	Chr4	38.462	169	43	2	41	148	22463224
lcl|AB793	Chr1	54.422	147	53	3	1	147	37513897
lcl|AB793	Chr12	56.481	108	46	1	41	148	12808340
lcl|AB793	Chr12	52.778	108	50	1	41	148	12792784
lcl|AB793	Chr2	41.88	117	37	1	63	148	15225499
lcl|AB793	Chr2	62.857	35	13	0	114	148	15204221
lcl|AB793	Chr2	60	30	12	0	63	92	15204468
lcl|AB793	Chr2	51.22	41	19	1	25	65	15204835
lcl|AB793	Chr3	65.714	35	12	0	114	148	15647702
lcl|AB793	Chr3	51.429	35	17	0	64	98	15647458

lcl|AB793	Chr3	37.5	64	28	2	33	87	15647274
lcl|AB793	Chr7	38.462	39	24	0	33	71	4666485

TBLASTN 2.10.1+
Query: lcl|AF465802.1_prot_AAL86777.1_1 [gene=HTR12] [protein=centromeric histone H3 HTF
Database: Nippon
Fields: query acc.ver, subject acc.ver, % identity, alignment length, mismatches, gap opens, q. s
20 hits found

lcl|AF465	Chr11	59	100	39	1	73	172	2626112
lcl|AF465	Chr6	59	100	39	1	73	172	3040837
lcl|AF465	Chr6	59	100	39	1	73	172	3055514
lcl|AF465	Chr6	59	100	39	1	73	172	3046867
lcl|AF465	Chr6	59	100	39	1	73	172	3056279
lcl|AF465	Chr6	58	100	40	1	73	172	1646006
lcl|AF465	Chr1	59.596	99	38	1	73	171	37514005
lcl|AF465	Chr5	59	100	39	1	73	172	21499987
lcl|AF465	Chr5	72.973	37	10	0	72	108	24069160
lcl|AF465	Chr5	53.846	26	12	0	138	163	24068434
lcl|AF465	Chr4	59	100	39	1	73	172	20739869
lcl|AF465	Chr4	36.875	160	41	3	73	172	22463251
lcl|AF465	Chr12	59	100	39	1	73	172	12808313
lcl|AF465	Chr12	55	100	43	1	73	172	12792757
lcl|AF465	Chr2	41.026	117	39	2	86	172	15225499
lcl|AF465	Chr2	65.714	35	12	0	138	172	15204221
lcl|AF465	Chr2	82.353	17	3	0	73	89	20134900
lcl|AF465	Chr3	68.571	35	11	0	138	172	15647702
lcl|AF465	Chr3	68.75	16	5	0	73	88	15647325
lcl|AF465	Chr3	51.852	27	13	0	87	113	15647458

TBLASTN 2.10.1+
Query: lcl|AF465800.1_prot_AAL86775.1_1 [gene=HTR12] [protein=centromeric histone H3 HTF
Database: Nippon
Fields: query acc.ver, subject acc.ver, % identity, alignment length, mismatches, gap opens, q. s
18 hits found

lcl|AF465	Chr6	53.982	113	49	2	63	174	3040876
lcl|AF465	Chr6	53.982	113	49	2	63	174	3055553
lcl|AF465	Chr6	53.043	115	51	2	61	174	3056234
lcl|AF465	Chr6	56.311	103	43	1	72	174	3046876
lcl|AF465	Chr6	57.282	103	42	1	72	174	1646015
lcl|AF465	Chr11	53.982	113	49	2	63	174	2626151
lcl|AF465	Chr1	53.509	114	50	2	61	173	37513960
lcl|AF465	Chr5	53.982	113	49	2	63	174	21500026
lcl|AF465	Chr5	67.568	37	12	0	74	110	24069160
lcl|AF465	Chr5	53.846	26	12	0	140	165	24068434
lcl|AF465	Chr4	53.043	115	51	2	61	174	20739824
lcl|AF465	Chr4	36.257	171	48	4	65	174	22463218
lcl|AF465	Chr12	50.847	118	54	3	63	178	12808352
lcl|AF465	Chr12	48.673	113	55	2	63	174	12792796
lcl|AF465	Chr2	40.171	117	40	2	88	174	15225499
lcl|AF465	Chr2	65.714	35	12	0	140	174	15204221

lcl|AF465{	Chr2	46.154	39	18	1	53	91	20134843
lcl|AF465{	Chr3	68.571	35	11	0	140	174	15647702

TBLASTN 2.10.1+
Query: lcl|AF465801.1_prot_AAL86776.1_1 [gene=HTR12] [protein=centromeric histone H3 HTI
Database: Nippon
Fields: query acc.ver, subject acc.ver, % identity, alignment length, mismatches, gap opens, q. s
20 hits found

lcl|AF465{	Chr11	59	100	39	1	73	172	2626112
lcl|AF465{	Chr6	59	100	39	1	73	172	3040837
lcl|AF465{	Chr6	59	100	39	1	73	172	3055514
lcl|AF465{	Chr6	59	100	39	1	73	172	3046867
lcl|AF465{	Chr6	59	100	39	1	73	172	3056279
lcl|AF465{	Chr6	58	100	40	1	73	172	1646006
lcl|AF465{	Chr1	59.596	99	38	1	73	171	37514005
lcl|AF465{	Chr5	59	100	39	1	73	172	21499987
lcl|AF465{	Chr5	72.973	37	10	0	72	108	24069160
lcl|AF465{	Chr5	53.846	26	12	0	138	163	24068434
lcl|AF465{	Chr4	59	100	39	1	73	172	20739869
lcl|AF465{	Chr4	36.875	160	41	3	73	172	22463251
lcl|AF465{	Chr12	59	100	39	1	73	172	12808313
lcl|AF465{	Chr12	55	100	43	1	73	172	12792757
lcl|AF465{	Chr2	41.026	117	39	2	86	172	15225499
lcl|AF465{	Chr2	65.714	35	12	0	138	172	15204221
lcl|AF465{	Chr2	82.353	17	3	0	73	89	20134900
lcl|AF465{	Chr3	68.571	35	11	0	138	172	15647702
lcl|AF465{	Chr3	68.75	16	5	0	73	88	15647325
lcl|AF465{	Chr3	51.852	27	13	0	87	113	15647458

TBLASTN 2.10.1+
Query: tr|A0A0G3YJC7|A0A0G3YJC7_AEGTA Centromeric histone H3 OS=Aegilops tauschii OX=
Database: Nippon
Fields: query acc.ver, subject acc.ver, % identity, alignment length, mismatches, gap opens, q. s
50 hits found

tr|A0A0G:	Chr11	55.714	140	56	4	1	139	2626220
tr|A0A0G:	Chr11	40.909	44	25	1	61	104	18024174
tr|A0A0G:	Chr11	37.037	27	17	0	88	114	13565372
tr|A0A0G:	Chr11	44.186	43	23	1	61	103	24024670
tr|A0A0G:	Chr6	55.714	140	56	4	1	139	3040945
tr|A0A0G:	Chr6	55.714	140	56	4	1	139	3055622
tr|A0A0G:	Chr6	55.714	140	56	4	1	139	3056171
tr|A0A0G:	Chr6	60.748	107	39	2	33	139	3046882
tr|A0A0G:	Chr6	60	100	39	1	40	139	1646006
tr|A0A0G:	Chr6	52.174	23	11	0	82	104	27987470
tr|A0A0G:	Chr6	52.174	23	11	0	82	104	16492739
tr|A0A0G:	Chr5	55.714	140	56	4	1	139	21500095
tr|A0A0G:	Chr5	75	40	10	0	36	75	24069169
tr|A0A0G:	Chr5	48.148	27	14	0	75	101	24068958
tr|A0A0G:	Chr5	76.923	26	6	0	99	124	24068449
tr|A0A0G:	Chr5	58.333	36	11	1	1	32	24070126

<table>
<tr><td>tr|A0A0G</td><td>Chr5</td><td>55.172</td><td>29</td><td>12</td><td>1</td><td>114</td><td>141</td><td>24068050</td></tr>
<tr><td>tr|A0A0G</td><td>Chr5</td><td>50</td><td>28</td><td>14</td><td>0</td><td>63</td><td>90</td><td>8282467</td></tr>
<tr><td>tr|A0A0G</td><td>Chr5</td><td>47.826</td><td>23</td><td>12</td><td>0</td><td>82</td><td>104</td><td>8334046</td></tr>
<tr><td>tr|A0A0G</td><td>Chr5</td><td>52.174</td><td>23</td><td>11</td><td>0</td><td>82</td><td>104</td><td>7739635</td></tr>
<tr><td>tr|A0A0G</td><td>Chr5</td><td>52.174</td><td>23</td><td>11</td><td>0</td><td>82</td><td>104</td><td>7092075</td></tr>
<tr><td>tr|A0A0G</td><td>Chr5</td><td>52.174</td><td>23</td><td>11</td><td>0</td><td>82</td><td>104</td><td>11985389</td></tr>
<tr><td>tr|A0A0G</td><td>Chr4</td><td>55.714</td><td>140</td><td>56</td><td>4</td><td>1</td><td>139</td><td>20739761</td></tr>
<tr><td>tr|A0A0G</td><td>Chr4</td><td>37.572</td><td>173</td><td>45</td><td>4</td><td>28</td><td>139</td><td>22463221</td></tr>
<tr><td>tr|A0A0G</td><td>Chr4</td><td>52.174</td><td>23</td><td>11</td><td>0</td><td>82</td><td>104</td><td>6034914</td></tr>
<tr><td>tr|A0A0G</td><td>Chr4</td><td>31.707</td><td>41</td><td>26</td><td>1</td><td>76</td><td>114</td><td>15614114</td></tr>
<tr><td>tr|A0A0G</td><td>Chr4</td><td>31.707</td><td>41</td><td>26</td><td>1</td><td>76</td><td>114</td><td>15776767</td></tr>
<tr><td>tr|A0A0G</td><td>Chr1</td><td>55.797</td><td>138</td><td>55</td><td>4</td><td>1</td><td>137</td><td>37513897</td></tr>
<tr><td>tr|A0A0G</td><td>Chr1</td><td>40.909</td><td>44</td><td>25</td><td>1</td><td>61</td><td>104</td><td>10092849</td></tr>
<tr><td>tr|A0A0G</td><td>Chr12</td><td>49.306</td><td>144</td><td>65</td><td>4</td><td>1</td><td>141</td><td>12808421</td></tr>
<tr><td>tr|A0A0G</td><td>Chr12</td><td>48.571</td><td>140</td><td>66</td><td>3</td><td>1</td><td>139</td><td>12792865</td></tr>
<tr><td>tr|A0A0G</td><td>Chr12</td><td>52.174</td><td>23</td><td>11</td><td>0</td><td>82</td><td>104</td><td>26725549</td></tr>
<tr><td>tr|A0A0G</td><td>Chr3</td><td>65.385</td><td>52</td><td>18</td><td>0</td><td>88</td><td>139</td><td>15647654</td></tr>
<tr><td>tr|A0A0G</td><td>Chr3</td><td>48.571</td><td>35</td><td>18</td><td>0</td><td>54</td><td>88</td><td>15647458</td></tr>
<tr><td>tr|A0A0G</td><td>Chr3</td><td>78.571</td><td>14</td><td>3</td><td>0</td><td>40</td><td>53</td><td>15647325</td></tr>
<tr><td>tr|A0A0G</td><td>Chr3</td><td>52.174</td><td>23</td><td>11</td><td>0</td><td>82</td><td>104</td><td>19623306</td></tr>
<tr><td>tr|A0A0G</td><td>Chr3</td><td>52.174</td><td>23</td><td>11</td><td>0</td><td>82</td><td>104</td><td>19302572</td></tr>
<tr><td>tr|A0A0G</td><td>Chr2</td><td>41.176</td><td>119</td><td>39</td><td>2</td><td>53</td><td>140</td><td>15225499</td></tr>
<tr><td>tr|A0A0G</td><td>Chr2</td><td>62.264</td><td>53</td><td>20</td><td>0</td><td>88</td><td>140</td><td>15204269</td></tr>
<tr><td>tr|A0A0G</td><td>Chr2</td><td>50</td><td>30</td><td>15</td><td>0</td><td>53</td><td>82</td><td>15204468</td></tr>
<tr><td>tr|A0A0G</td><td>Chr2</td><td>36.364</td><td>66</td><td>42</td><td>0</td><td>9</td><td>74</td><td>20134807</td></tr>
<tr><td>tr|A0A0G</td><td>Chr2</td><td>39.583</td><td>48</td><td>19</td><td>2</td><td>94</td><td>139</td><td>20135034</td></tr>
<tr><td>tr|A0A0G</td><td>Chr2</td><td>68.75</td><td>16</td><td>5</td><td>0</td><td>40</td><td>55</td><td>15225769</td></tr>
<tr><td>tr|A0A0G</td><td>Chr7</td><td>38.636</td><td>44</td><td>26</td><td>1</td><td>61</td><td>104</td><td>4633533</td></tr>
<tr><td>tr|A0A0G</td><td>Chr7</td><td>30.508</td><td>59</td><td>41</td><td>0</td><td>25</td><td>83</td><td>10560862</td></tr>
<tr><td>tr|A0A0G</td><td>Chr7</td><td>27.273</td><td>44</td><td>30</td><td>1</td><td>73</td><td>114</td><td>12292680</td></tr>
<tr><td>tr|A0A0G</td><td>Chr8</td><td>47.826</td><td>23</td><td>12</td><td>0</td><td>82</td><td>104</td><td>13449432</td></tr>
<tr><td>tr|A0A0G</td><td>Chr9</td><td>52.174</td><td>23</td><td>11</td><td>0</td><td>82</td><td>104</td><td>7580827</td></tr>
<tr><td>tr|A0A0G</td><td>Chr10</td><td>52.174</td><td>23</td><td>11</td><td>0</td><td>82</td><td>104</td><td>13418274</td></tr>
<tr><td>tr|A0A0G</td><td>Chr10</td><td>52.174</td><td>23</td><td>11</td><td>0</td><td>82</td><td>104</td><td>9325236</td></tr>
</table>

TBLASTN 2.10.1+
Query: tr|A0A0B6VLZ9|A0A0B6VLZ9_AVESA Centromere specific histone H3 (Fragment) OS=A
Database: Nippon
Fields: query acc.ver, subject acc.ver, % identity, alignment length, mismatches, gap opens, q. s
25 hits found

<table>
<tr><td>tr|A0A0B</td><td>Chr11</td><td>67.708</td><td>96</td><td>28</td><td>2</td><td>66</td><td>160</td><td>2626109</td></tr>
<tr><td>tr|A0A0B</td><td>Chr6</td><td>67.708</td><td>96</td><td>28</td><td>2</td><td>66</td><td>160</td><td>3040834</td></tr>
<tr><td>tr|A0A0B</td><td>Chr6</td><td>67.708</td><td>96</td><td>28</td><td>2</td><td>66</td><td>160</td><td>3055511</td></tr>
<tr><td>tr|A0A0B</td><td>Chr6</td><td>67.708</td><td>96</td><td>28</td><td>2</td><td>66</td><td>160</td><td>3046864</td></tr>
<tr><td>tr|A0A0B</td><td>Chr6</td><td>67.708</td><td>96</td><td>28</td><td>2</td><td>66</td><td>160</td><td>3056282</td></tr>
<tr><td>tr|A0A0B</td><td>Chr6</td><td>66.667</td><td>96</td><td>29</td><td>2</td><td>66</td><td>160</td><td>1646003</td></tr>
<tr><td>tr|A0A0B</td><td>Chr5</td><td>67.708</td><td>96</td><td>28</td><td>2</td><td>66</td><td>160</td><td>21499984</td></tr>
<tr><td>tr|A0A0B</td><td>Chr5</td><td>88.571</td><td>35</td><td>4</td><td>0</td><td>66</td><td>100</td><td>24069154</td></tr>
<tr><td>tr|A0A0B</td><td>Chr5</td><td>51.852</td><td>27</td><td>13</td><td>0</td><td>100</td><td>126</td><td>24068958</td></tr>
</table>

tr|A0A0B(	Chr5	75	28	7	0	122	149	24068455
tr|A0A0B(	Chr5	61.111	36	14	0	1	36	24070126
tr|A0A0B(	Chr1	67.708	96	28	2	66	160	37514008
tr|A0A0B(	Chr4	67.708	96	28	2	66	160	20739872
tr|A0A0B(	Chr4	41.667	156	30	3	66	160	22463254
tr|A0A0B(	Chr12	67.368	95	30	1	66	160	12808310
tr|A0A0B(	Chr12	64.211	95	33	1	66	160	12792754
tr|A0A0B(	Chr2	44.737	114	32	1	78	160	15225499
tr|A0A0B(	Chr2	64.583	48	17	0	113	160	15204269
tr|A0A0B(	Chr2	73.077	26	7	0	78	103	15204468
tr|A0A0B(	Chr2	82.353	17	3	0	66	82	20134903
tr|A0A0B(	Chr2	73.333	15	4	0	66	80	15225766
tr|A0A0B(	Chr3	68.75	48	15	0	113	160	15647654
tr|A0A0B(	Chr3	76	25	6	0	79	103	15647458
tr|A0A0B(	Chr3	43.182	44	17	2	66	102	15647328
tr|A0A0B(	Chr9	42.222	45	22	2	79	121	1018147

TBLASTN 2.10.1+
Query: tr|A0A0B6VNT5|A0A0B6VNT5_AVESA Centromere specific histone H3 (Fragment) OS=/
Database: Nippon
Fields: query acc.ver, subject acc.ver, % identity, alignment length, mismatches, gap opens, q. s
25 hits found

tr|A0A0B(	Chr11	67.01	97	29	2	62	157	2626112
tr|A0A0B(	Chr6	67.01	97	29	2	62	157	3040837
tr|A0A0B(	Chr6	67.01	97	29	2	62	157	3055514
tr|A0A0B(	Chr6	67.01	97	29	2	62	157	3046867
tr|A0A0B(	Chr6	67.01	97	29	2	62	157	3056279
tr|A0A0B(	Chr6	65.306	98	31	2	61	157	1646009
tr|A0A0B(	Chr5	67.01	97	29	2	62	157	21499987
tr|A0A0B(	Chr5	80.488	41	8	0	57	97	24069172
tr|A0A0B(	Chr5	51.852	27	13	0	97	123	24068958
tr|A0A0B(	Chr5	75	28	7	0	119	146	24068455
tr|A0A0B(	Chr5	72.727	22	6	0	1	22	24070126
tr|A0A0B(	Chr1	67.01	97	29	2	62	157	37514005
tr|A0A0B(	Chr12	66.667	96	31	1	62	157	12808313
tr|A0A0B(	Chr12	63.542	96	34	1	62	157	12792757
tr|A0A0B(	Chr4	66.327	98	30	2	61	157	20739866
tr|A0A0B(	Chr4	40.506	158	33	3	61	157	22463248
tr|A0A0B(	Chr2	44.737	114	32	1	75	157	15225499
tr|A0A0B(	Chr2	64.583	48	17	0	110	157	15204269
tr|A0A0B(	Chr2	73.077	26	7	0	75	100	15204468
tr|A0A0B(	Chr2	78.947	19	4	0	61	79	20134897
tr|A0A0B(	Chr2	64.706	17	6	0	61	77	15225772
tr|A0A0B(	Chr3	68.75	48	15	0	110	157	15647654
tr|A0A0B(	Chr3	76	25	6	0	76	100	15647458
tr|A0A0B(	Chr3	42.222	45	18	2	62	99	15647325
tr|A0A0B(	Chr9	42.222	45	22	2	76	118	1018147

TBLASTN 2.10.1+
Query: tr|A0A0G3YJC9|A0A0G3YJC9_AEGSP Centromeric histone H3 OS=Aegilops speltoides O

Database: Nippon
Fields: query acc.ver, subject acc.ver, % identity, alignment length, mismatches, gap opens, q. s
23 hits found

tr|A0A0G:	Chr11	64.356	101	33	3	52	151	2626112
tr|A0A0G:	Chr6	64.356	101	33	3	52	151	3040837
tr|A0A0G:	Chr6	64.356	101	33	3	52	151	3055514
tr|A0A0G:	Chr6	64.356	101	33	3	52	151	3056279
tr|A0A0G:	Chr6	64.356	101	33	3	52	151	1646006
tr|A0A0G:	Chr6	64.356	101	33	3	52	151	3046867
tr|A0A0G:	Chr5	64.356	101	33	3	52	151	21499987
tr|A0A0G:	Chr5	75.676	37	9	0	50	86	24069163
tr|A0A0G:	Chr5	74.194	31	8	0	106	136	24068464
tr|A0A0G:	Chr5	41.176	34	20	0	120	153	24068065
tr|A0A0G:	Chr4	64.356	101	33	3	52	151	20739869
tr|A0A0G:	Chr4	39.752	161	36	3	52	151	22463251
tr|A0A0G:	Chr1	64.646	99	32	3	52	149	37514005
tr|A0A0G:	Chr12	57.426	101	40	2	52	151	12808313
tr|A0A0G:	Chr12	57.426	101	40	2	52	151	12792757
tr|A0A0G:	Chr3	66.667	51	17	0	101	151	15647657
tr|A0A0G:	Chr3	62.5	32	11	1	66	96	15647458
tr|A0A0G:	Chr3	55.556	18	8	0	52	69	15647325
tr|A0A0G:	Chr2	45.833	120	32	4	65	152	15225499
tr|A0A0G:	Chr2	58.333	60	22	1	96	152	15204290
tr|A0A0G:	Chr2	72.727	22	6	0	65	86	15204468
tr|A0A0G:	Chr2	72.222	18	5	0	52	69	20134900
tr|A0A0G:	Chr2	36	50	22	2	104	151	20135028

TBLASTN 2.10.1+
Query: tr|A0A0B6VJW4|A0A0B6VJW4_AVESA Centromere specific histone H3 (Fragment) OS=
Database: Nippon
Fields: query acc.ver, subject acc.ver, % identity, alignment length, mismatches, gap opens, q. s
26 hits found

tr|A0A0B6	Chr11	70.833	96	25	2	66	160	2626109
tr|A0A0B6	Chr11	29.851	67	32	1	67	118	8742263
tr|A0A0B6	Chr6	70.833	96	25	2	66	160	3040834
tr|A0A0B6	Chr6	70.833	96	25	2	66	160	3055511
tr|A0A0B6	Chr6	70.833	96	25	2	66	160	3046864
tr|A0A0B6	Chr6	70.833	96	25	2	66	160	3056282
tr|A0A0B6	Chr6	69.792	96	26	2	66	160	1646003
tr|A0A0B6	Chr5	70.833	96	25	2	66	160	21499984
tr|A0A0B6	Chr5	85.714	35	5	0	66	100	24069154
tr|A0A0B6	Chr5	59.259	27	11	0	100	126	24068958
tr|A0A0B6	Chr5	75	28	7	0	122	149	24068455
tr|A0A0B6	Chr5	61.111	36	14	0	1	36	24070126
tr|A0A0B6	Chr1	70.833	96	25	2	66	160	37514008
tr|A0A0B6	Chr4	70.833	96	25	2	66	160	20739872
tr|A0A0B6	Chr4	42.949	156	28	3	66	160	22463254
tr|A0A0B6	Chr12	69.474	95	28	1	66	160	12808310
tr|A0A0B6	Chr12	66.316	95	31	1	66	160	12792754

<table>
<tr><td>tr|A0A0B6</td><td>Chr2</td><td>45.614</td><td>114</td><td>31</td><td>2</td><td>78</td><td>160</td><td>15225499</td></tr>
<tr><td>tr|A0A0B6</td><td>Chr2</td><td>66.667</td><td>48</td><td>16</td><td>0</td><td>113</td><td>160</td><td>15204269</td></tr>
<tr><td>tr|A0A0B6</td><td>Chr2</td><td>76.923</td><td>26</td><td>6</td><td>0</td><td>78</td><td>103</td><td>15204468</td></tr>
<tr><td>tr|A0A0B6</td><td>Chr2</td><td>82.353</td><td>17</td><td>3</td><td>0</td><td>66</td><td>82</td><td>20134903</td></tr>
<tr><td>tr|A0A0B6</td><td>Chr2</td><td>37.647</td><td>85</td><td>37</td><td>4</td><td>78</td><td>160</td><td>20134929</td></tr>
<tr><td>tr|A0A0B6</td><td>Chr2</td><td>73.333</td><td>15</td><td>4</td><td>0</td><td>66</td><td>80</td><td>15225766</td></tr>
<tr><td>tr|A0A0B6</td><td>Chr3</td><td>70.833</td><td>48</td><td>14</td><td>0</td><td>113</td><td>160</td><td>15647654</td></tr>
<tr><td>tr|A0A0B6</td><td>Chr3</td><td>75.862</td><td>29</td><td>6</td><td>1</td><td>79</td><td>106</td><td>15647458</td></tr>
<tr><td>tr|A0A0B6</td><td>Chr3</td><td>43.182</td><td>44</td><td>17</td><td>2</td><td>66</td><td>102</td><td>15647328</td></tr>
</table>

TBLASTN 2.10.1+
Query: tr|A0A0H3VKW3|A0A0H3VKW3_9POAL Centromeric histone H3 isoform B (Fragment) C
Database: Nippon
Fields: query acc.ver, subject acc.ver, % identity, alignment length, mismatches, gap opens, q. s
23 hits found

<table>
<tr><td>tr|A0A0H3</td><td>Chr6</td><td>58.929</td><td>112</td><td>39</td><td>2</td><td>55</td><td>166</td><td>3040867</td></tr>
<tr><td>tr|A0A0H3</td><td>Chr6</td><td>58.929</td><td>112</td><td>39</td><td>2</td><td>55</td><td>166</td><td>3056249</td></tr>
<tr><td>tr|A0A0H3</td><td>Chr6</td><td>58.929</td><td>112</td><td>39</td><td>2</td><td>55</td><td>166</td><td>3055544</td></tr>
<tr><td>tr|A0A0H3</td><td>Chr6</td><td>52.143</td><td>140</td><td>55</td><td>5</td><td>29</td><td>166</td><td>3046972</td></tr>
<tr><td>tr|A0A0H3</td><td>Chr6</td><td>59.821</td><td>112</td><td>38</td><td>2</td><td>55</td><td>166</td><td>1646036</td></tr>
<tr><td>tr|A0A0H3</td><td>Chr11</td><td>58.929</td><td>112</td><td>39</td><td>2</td><td>55</td><td>166</td><td>2626142</td></tr>
<tr><td>tr|A0A0H3</td><td>Chr5</td><td>58.929</td><td>112</td><td>39</td><td>2</td><td>55</td><td>166</td><td>21500017</td></tr>
<tr><td>tr|A0A0H3</td><td>Chr5</td><td>67.5</td><td>40</td><td>13</td><td>0</td><td>67</td><td>106</td><td>24069169</td></tr>
<tr><td>tr|A0A0H3</td><td>Chr5</td><td>73.333</td><td>15</td><td>4</td><td>0</td><td>106</td><td>120</td><td>24068958</td></tr>
<tr><td>tr|A0A0H3</td><td>Chr5</td><td>63.636</td><td>22</td><td>8</td><td>0</td><td>134</td><td>155</td><td>24068437</td></tr>
<tr><td>tr|A0A0H3</td><td>Chr1</td><td>58.929</td><td>112</td><td>39</td><td>2</td><td>55</td><td>166</td><td>37513975</td></tr>
<tr><td>tr|A0A0H3</td><td>Chr4</td><td>58.929</td><td>112</td><td>39</td><td>2</td><td>55</td><td>166</td><td>20739839</td></tr>
<tr><td>tr|A0A0H3</td><td>Chr4</td><td>39.655</td><td>174</td><td>36</td><td>5</td><td>55</td><td>166</td><td>22463221</td></tr>
<tr><td>tr|A0A0H3</td><td>Chr12</td><td>56.14</td><td>114</td><td>43</td><td>2</td><td>53</td><td>166</td><td>12808349</td></tr>
<tr><td>tr|A0A0H3</td><td>Chr12</td><td>53.043</td><td>115</td><td>47</td><td>2</td><td>53</td><td>167</td><td>12792793</td></tr>
<tr><td>tr|A0A0H3</td><td>Chr2</td><td>45.614</td><td>114</td><td>31</td><td>2</td><td>84</td><td>166</td><td>15225499</td></tr>
<tr><td>tr|A0A0H3</td><td>Chr2</td><td>71.875</td><td>32</td><td>9</td><td>0</td><td>135</td><td>166</td><td>15204221</td></tr>
<tr><td>tr|A0A0H3</td><td>Chr2</td><td>54.054</td><td>37</td><td>15</td><td>1</td><td>84</td><td>118</td><td>15204468</td></tr>
<tr><td>tr|A0A0H3</td><td>Chr2</td><td>42.857</td><td>35</td><td>20</td><td>0</td><td>71</td><td>105</td><td>20134900</td></tr>
<tr><td>tr|A0A0H3</td><td>Chr2</td><td>50</td><td>32</td><td>10</td><td>1</td><td>55</td><td>86</td><td>15225799</td></tr>
<tr><td>tr|A0A0H3</td><td>Chr3</td><td>78.125</td><td>32</td><td>7</td><td>0</td><td>135</td><td>166</td><td>15647702</td></tr>
<tr><td>tr|A0A0H3</td><td>Chr3</td><td>60</td><td>25</td><td>10</td><td>0</td><td>85</td><td>109</td><td>15647458</td></tr>
<tr><td>tr|A0A0H3</td><td>Chr3</td><td>38.596</td><td>57</td><td>23</td><td>3</td><td>55</td><td>106</td><td>15647295</td></tr>
</table>

TBLASTN 2.10.1+
Query: tr|A0A0H3VKW8|A0A0H3VKW8_9POAL Centromeric histone H3 isoform A (Fragment) C
Database: Nippon
Fields: query acc.ver, subject acc.ver, % identity, alignment length, mismatches, gap opens, q. s
24 hits found

<table>
<tr><td>tr|A0A0H3</td><td>Chr6</td><td>55.455</td><td>110</td><td>48</td><td>1</td><td>55</td><td>164</td><td>3056237</td></tr>
<tr><td>tr|A0A0H3</td><td>Chr6</td><td>57.407</td><td>108</td><td>45</td><td>1</td><td>57</td><td>164</td><td>1646042</td></tr>
<tr><td>tr|A0A0H3</td><td>Chr6</td><td>55.455</td><td>110</td><td>48</td><td>1</td><td>55</td><td>164</td><td>3040879</td></tr>
<tr><td>tr|A0A0H3</td><td>Chr6</td><td>55.455</td><td>110</td><td>48</td><td>1</td><td>55</td><td>164</td><td>3055556</td></tr>
<tr><td>tr|A0A0H3</td><td>Chr6</td><td>61.458</td><td>96</td><td>36</td><td>1</td><td>69</td><td>164</td><td>3046867</td></tr>
<tr><td>tr|A0A0H3</td><td>Chr1</td><td>55.963</td><td>109</td><td>47</td><td>1</td><td>56</td><td>164</td><td>37513966</td></tr>
</table>

tr|A0A0H3	Chr12	51.24	121	58	1	44	164	12808388
tr|A0A0H3	Chr12	50	116	57	1	50	165	12792814
tr|A0A0H3	Chr4	55.455	110	48	1	55	164	20739827
tr|A0A0H3	Chr4	35.882	170	48	3	56	164	22463212
tr|A0A0H3	Chr5	55.455	110	48	1	55	164	21500029
tr|A0A0H3	Chr5	72.5	40	11	0	65	104	24069169
tr|A0A0H3	Chr5	57.576	33	12	1	104	134	24068958
tr|A0A0H3	Chr5	53.333	30	14	0	126	155	24068455
tr|A0A0H3	Chr11	55.455	110	48	1	55	164	2626154
tr|A0A0H3	Chr2	42.982	114	34	2	82	164	15225499
tr|A0A0H3	Chr2	55.357	56	25	0	109	164	15204293
tr|A0A0H3	Chr2	51.351	37	16	1	82	116	15204468
tr|A0A0H3	Chr2	39.062	64	37	2	49	112	20134843
tr|A0A0H3	Chr2	42.857	42	14	2	125	164	20135040
tr|A0A0H3	Chr3	57.627	59	25	0	106	164	15647621
tr|A0A0H3	Chr3	60	25	10	0	83	107	15647458
tr|A0A0H3	Chr3	37.778	45	23	1	56	95	15647286
tr|A0A0H3	Chr3	37.736	53	32	1	43	94	25276911

TBLASTN 2.10.1+
Query: tr|A0A0H3VKW4|A0A0H3VKW4_9POAL Centromeric histone H3 isoform A (Fragment) C
Database: Nippon
Fields: query acc.ver, subject acc.ver, % identity, alignment length, mismatches, gap opens, q. s
24 hits found

tr|A0A0H3	Chr1	58.333	96	39	1	66	161	37514005
tr|A0A0H3	Chr1	42.857	42	23	1	75	115	4816782
tr|A0A0H3	Chr6	58.333	96	39	1	66	161	3056279
tr|A0A0H3	Chr6	60.417	96	37	1	66	161	1646006
tr|A0A0H3	Chr6	58.333	96	39	1	66	161	3040837
tr|A0A0H3	Chr6	58.333	96	39	1	66	161	3055514
tr|A0A0H3	Chr6	58.333	96	39	1	66	161	3046867
tr|A0A0H3	Chr4	58.333	96	39	1	66	161	20739869
tr|A0A0H3	Chr4	36.943	157	38	3	66	161	22463251
tr|A0A0H3	Chr11	58.333	96	39	1	66	161	2626112
tr|A0A0H3	Chr5	58.333	96	39	1	66	161	21499987
tr|A0A0H3	Chr5	72.5	40	11	0	62	101	24069169
tr|A0A0H3	Chr5	59.259	27	11	0	101	127	24068958
tr|A0A0H3	Chr5	42.105	38	22	0	115	152	24068479
tr|A0A0H3	Chr12	44.776	134	71	2	28	161	12808421
tr|A0A0H3	Chr12	52.577	97	45	1	66	162	12792757
tr|A0A0H3	Chr3	62.5	48	18	0	114	161	15647654
tr|A0A0H3	Chr3	64	25	9	0	80	104	15647458
tr|A0A0H3	Chr3	57.143	21	9	0	61	81	15647310
tr|A0A0H3	Chr2	41.228	114	36	2	79	161	15225499
tr|A0A0H3	Chr2	58.333	48	20	0	114	161	15204269
tr|A0A0H3	Chr2	51.351	37	16	1	79	113	15204468
tr|A0A0H3	Chr2	36.842	57	35	1	66	122	20134900
tr|A0A0H3	Chr2	50	34	14	1	9	42	7595248

TBLASTN 2.10.1+

Query: tr|A0A0H3VKW7|A0A0H3VKW7_9POAL Centromeric histone H3 isoform A (Fragment) O
Database: Nippon
Fields: query acc.ver, subject acc.ver, % identity, alignment length, mismatches, gap opens, q. s
22 hits found

tr|A0A0H3	Chr1	55.046	109	48	1	56	164	37513966
tr|A0A0H3	Chr6	56.481	108	46	1	57	164	1646042
tr|A0A0H3	Chr6	54.545	110	49	1	55	164	3056237
tr|A0A0H3	Chr6	54.545	110	49	1	55	164	3040879
tr|A0A0H3	Chr6	54.545	110	49	1	55	164	3055556
tr|A0A0H3	Chr6	60.417	96	37	1	69	164	3046867
tr|A0A0H3	Chr5	54.545	110	49	1	55	164	21500029
tr|A0A0H3	Chr5	76.316	38	9	0	67	104	24069163
tr|A0A0H3	Chr5	57.576	33	12	1	104	134	24068958
tr|A0A0H3	Chr5	56.667	30	13	0	126	155	24068455
tr|A0A0H3	Chr4	54.545	110	49	1	55	164	20739827
tr|A0A0H3	Chr4	35.882	170	48	2	56	164	22463212
tr|A0A0H3	Chr11	54.545	110	49	1	55	164	2626154
tr|A0A0H3	Chr12	50.413	121	59	1	44	164	12808388
tr|A0A0H3	Chr12	50	116	57	1	50	165	12792814
tr|A0A0H3	Chr2	43.86	114	33	2	82	164	15225499
tr|A0A0H3	Chr2	62.5	48	18	0	117	164	15204269
tr|A0A0H3	Chr2	54.054	37	15	1	82	116	15204468
tr|A0A0H3	Chr2	47.368	38	19	1	49	86	20134843
tr|A0A0H3	Chr3	66.667	48	16	0	117	164	15647654
tr|A0A0H3	Chr3	64	25	9	0	83	107	15647458
tr|A0A0H3	Chr3	34.568	81	43	4	56	129	15647286

TBLASTN 2.10.1+
Query: tr|A0A0H3VKV8|A0A0H3VKV8_9POAL Centromeric histone H3 isoform A (Fragment) OS
Database: Nippon
Fields: query acc.ver, subject acc.ver, % identity, alignment length, mismatches, gap opens, q. s
25 hits found

tr|A0A0H3	Chr6	47.368	152	72	2	13	164	3056132
tr|A0A0H3	Chr6	47.368	152	72	2	13	164	3040984
tr|A0A0H3	Chr6	58.333	108	44	1	57	164	1646042
tr|A0A0H3	Chr6	50.37	135	59	2	30	164	3055610
tr|A0A0H3	Chr6	54.31	116	52	1	49	164	3046927
tr|A0A0H3	Chr1	54.783	115	51	1	50	164	37513948
tr|A0A0H3	Chr5	50.37	135	59	2	30	164	21500083
tr|A0A0H3	Chr5	75	40	10	0	65	104	24069169
tr|A0A0H3	Chr5	57.576	33	12	1	104	134	24068958
tr|A0A0H3	Chr5	53.333	30	14	0	126	155	24068455
tr|A0A0H3	Chr4	50.37	135	59	2	30	164	20739773
tr|A0A0H3	Chr4	36.471	170	47	3	56	164	22463212
tr|A0A0H3	Chr11	50.37	135	59	2	30	164	2626208
tr|A0A0H3	Chr12	51.24	121	58	1	44	164	12808388
tr|A0A0H3	Chr12	50	116	57	1	50	165	12792814
tr|A0A0H3	Chr3	57.627	59	25	0	106	164	15647621
tr|A0A0H3	Chr3	64	25	9	0	83	107	15647458

tr|A0A0H3	Chr3	44.828	29	16	0	56	84	15647286
tr|A0A0H3	Chr3	37.736	53	32	1	43	94	25276911
tr|A0A0H3	Chr3	38.095	42	21	1	54	95	23469204
tr|A0A0H3	Chr2	43.86	114	33	2	82	164	15225499
tr|A0A0H3	Chr2	54.386	57	26	0	108	164	15204296
tr|A0A0H3	Chr2	54.054	37	15	1	82	116	15204468
tr|A0A0H3	Chr2	39.062	64	37	2	49	112	20134843
tr|A0A0H3	Chr2	42.857	42	14	2	125	164	20135040

TBLASTN 2.10.1+
Query: tr|A0A0H3VKW5|A0A0H3VKW5_9POAL Centromeric histone H3 isoform A (Fragment) C
Database: Nippon
Fields: query acc.ver, subject acc.ver, % identity, alignment length, mismatches, gap opens, q. s
25 hits found

tr|A0A0H3	Chr1	54.128	109	49	1	56	164	37513966
tr|A0A0H3	Chr6	55.556	108	47	1	57	164	1646042
tr|A0A0H3	Chr6	53.636	110	50	1	55	164	3056237
tr|A0A0H3	Chr6	53.636	110	50	1	55	164	3040879
tr|A0A0H3	Chr6	53.636	110	50	1	55	164	3055556
tr|A0A0H3	Chr6	59.375	96	38	1	69	164	3046867
tr|A0A0H3	Chr4	53.636	110	50	1	55	164	20739827
tr|A0A0H3	Chr4	35.294	170	49	2	56	164	22463212
tr|A0A0H3	Chr5	53.636	110	50	1	55	164	21500029
tr|A0A0H3	Chr5	72.5	40	11	0	65	104	24069169
tr|A0A0H3	Chr5	54.545	33	13	1	104	134	24068958
tr|A0A0H3	Chr5	53.333	30	14	0	126	155	24068455
tr|A0A0H3	Chr11	53.636	110	50	1	55	164	2626154
tr|A0A0H3	Chr12	49.587	121	60	1	44	164	12808388
tr|A0A0H3	Chr12	50	116	57	1	50	165	12792814
tr|A0A0H3	Chr2	42.982	114	34	2	82	164	15225499
tr|A0A0H3	Chr2	60.417	48	19	0	117	164	15204269
tr|A0A0H3	Chr2	54.054	37	15	1	82	116	15204468
tr|A0A0H3	Chr2	47.368	38	19	1	49	86	20134843
tr|A0A0H3	Chr2	41.463	41	14	2	126	164	20135043
tr|A0A0H3	Chr2	50	34	14	1	9	42	7595248
tr|A0A0H3	Chr3	64.583	48	17	0	117	164	15647654
tr|A0A0H3	Chr3	64	25	9	0	83	107	15647458
tr|A0A0H3	Chr3	37.736	53	32	1	43	94	25276911
tr|A0A0H3	Chr3	35.185	54	29	2	56	104	15647286

TBLASTN 2.10.1+
Query: tr|A0A0H3VKX0|A0A0H3VKX0_9POAL Centromeric histone H3 isoform A (Fragment) OS
Database: Nippon
Fields: query acc.ver, subject acc.ver, % identity, alignment length, mismatches, gap opens, q. s
23 hits found

tr|A0A0H3	Chr1	53.211	109	50	1	56	164	37513966
tr|A0A0H3	Chr6	54.63	108	48	1	57	164	1646042
tr|A0A0H3	Chr6	52.727	110	51	1	55	164	3056237
tr|A0A0H3	Chr6	52.727	110	51	1	55	164	3040879
tr|A0A0H3	Chr6	52.727	110	51	1	55	164	3055556

tr|A0A0H3	Chr6	58.333	96	39	1	69	164	3046867
tr|A0A0H3	Chr4	52.727	110	51	1	55	164	20739827
tr|A0A0H3	Chr4	34.706	170	50	2	56	164	22463212
tr|A0A0H3	Chr5	52.727	110	51	1	55	164	21500029
tr|A0A0H3	Chr5	77.5	40	9	0	65	104	24069169
tr|A0A0H3	Chr5	57.576	33	12	1	104	134	24068958
tr|A0A0H3	Chr5	53.333	30	14	0	126	155	24068455
tr|A0A0H3	Chr11	52.727	110	51	1	55	164	2626154
tr|A0A0H3	Chr12	47.934	121	62	1	44	164	12808388
tr|A0A0H3	Chr12	47.414	116	60	1	50	165	12792814
tr|A0A0H3	Chr2	42.105	114	35	2	82	164	15225499
tr|A0A0H3	Chr2	58.333	48	20	0	117	164	15204269
tr|A0A0H3	Chr2	54.054	37	15	1	82	116	15204468
tr|A0A0H3	Chr2	44.737	38	20	1	49	86	20134843
tr|A0A0H3	Chr2	50	34	14	1	9	42	7595248
tr|A0A0H3	Chr3	62.5	48	18	0	117	164	15647654
tr|A0A0H3	Chr3	64	25	9	0	83	107	15647458
tr|A0A0H3	Chr3	34.568	81	43	4	56	129	15647286

TBLASTN 2.10.1+
Query: tr|A0A0H3VKX1|A0A0H3VKX1_9POAL Centromeric histone H3 isoform A (Fragment) OS
Database: Nippon
Fields: query acc.ver, subject acc.ver, % identity, alignment length, mismatches, gap opens, q. s
26 hits found

tr|A0A0H3	Chr1	54.128	109	49	1	56	164	37513966
tr|A0A0H3	Chr6	46.053	152	74	3	13	164	3056132
tr|A0A0H3	Chr6	46.053	152	74	3	13	164	3040984
tr|A0A0H3	Chr6	55.556	108	47	1	57	164	1646042
tr|A0A0H3	Chr6	52.459	122	54	2	46	164	3055592
tr|A0A0H3	Chr6	54.545	110	49	1	55	164	3046909
tr|A0A0H3	Chr4	52.459	122	54	2	46	164	20739791
tr|A0A0H3	Chr4	35.294	170	49	2	56	164	22463212
tr|A0A0H3	Chr11	52.459	122	54	2	46	164	2626190
tr|A0A0H3	Chr5	52.459	122	54	2	46	164	21500065
tr|A0A0H3	Chr5	75	40	10	0	65	104	24069169
tr|A0A0H3	Chr5	57.576	33	12	1	104	134	24068958
tr|A0A0H3	Chr5	53.333	30	14	0	126	155	24068455
tr|A0A0H3	Chr12	49.587	121	60	1	44	164	12808388
tr|A0A0H3	Chr12	49.138	116	58	1	50	165	12792814
tr|A0A0H3	Chr2	42.982	114	34	2	82	164	15225499
tr|A0A0H3	Chr2	60.417	48	19	0	117	164	15204269
tr|A0A0H3	Chr2	54.054	37	15	1	82	116	15204468
tr|A0A0H3	Chr2	47.368	38	19	1	49	86	20134843
tr|A0A0H3	Chr2	42.857	42	14	2	125	164	20135040
tr|A0A0H3	Chr3	64.583	48	17	0	117	164	15647654
tr|A0A0H3	Chr3	64	25	9	0	83	107	15647458
tr|A0A0H3	Chr3	37.736	53	32	1	43	94	25276911
tr|A0A0H3	Chr3	34.568	81	43	4	56	129	15647286
tr|A0A0H3	Chr3	35.714	42	22	1	54	95	23469204

tr|A0A0H3	Chr10	43.59	39	18	1	49	83	18761422

TBLASTN 2.10.1+
Query: tr|A0A0H3VKV7|A0A0H3VKV7_9POAL Centromeric histone H3 isoform A (Fragment) OS
Database: Nippon
Fields: query acc.ver, subject acc.ver, % identity, alignment length, mismatches, gap opens, q. s
24 hits found

tr|A0A0H3	Chr1	55.046	109	48	1	56	164	37513966
tr|A0A0H3	Chr6	57.407	108	45	1	57	164	1646042
tr|A0A0H3	Chr6	54.545	110	49	1	55	164	3040879
tr|A0A0H3	Chr6	55.455	110	48	1	55	164	3056237
tr|A0A0H3	Chr6	54.545	110	49	1	55	164	3055556
tr|A0A0H3	Chr6	60.417	96	37	1	69	164	3046867
tr|A0A0H3	Chr4	54.545	110	49	1	55	164	20739827
tr|A0A0H3	Chr4	36.471	170	47	3	56	164	22463212
tr|A0A0H3	Chr5	54.545	110	49	1	55	164	21500029
tr|A0A0H3	Chr5	72.5	40	11	0	65	104	24069169
tr|A0A0H3	Chr5	54.545	33	13	1	104	134	24068958
tr|A0A0H3	Chr5	44.737	38	21	0	118	155	24068479
tr|A0A0H3	Chr5	35.556	45	27	1	106	150	11619317
tr|A0A0H3	Chr11	54.545	110	49	1	55	164	2626154
tr|A0A0H3	Chr12	49.587	121	60	1	44	164	12808388
tr|A0A0H3	Chr12	48.276	116	59	1	50	165	12792814
tr|A0A0H3	Chr3	66.667	48	16	0	117	164	15647654
tr|A0A0H3	Chr3	64	25	9	0	83	107	15647458
tr|A0A0H3	Chr3	44.828	29	16	0	56	84	15647286
tr|A0A0H3	Chr2	42.982	114	34	2	82	164	15225499
tr|A0A0H3	Chr2	62.5	48	18	0	117	164	15204269
tr|A0A0H3	Chr2	51.351	37	16	1	82	116	15204468
tr|A0A0H3	Chr2	33.333	81	49	3	49	126	20134843
tr|A0A0H3	Chr2	50	34	14	1	9	42	7595248

TBLASTN 2.10.1+
Query: tr|A0A0H3VKW2|A0A0H3VKW2_9POAL Centromeric histone H3 isoform B (Fragment) C
Database: Nippon
Fields: query acc.ver, subject acc.ver, % identity, alignment length, mismatches, gap opens, q. s
24 hits found

tr|A0A0H3	Chr1	62.5	96	35	1	64	159	37514005
tr|A0A0H3	Chr6	47.297	148	64	3	12	159	3040954
tr|A0A0H3	Chr6	62.5	96	35	1	64	159	3056279
tr|A0A0H3	Chr6	47.887	142	60	3	18	159	3055613
tr|A0A0H3	Chr6	62.5	96	35	1	64	159	3046867
tr|A0A0H3	Chr6	63.542	96	34	1	64	159	1646006
tr|A0A0H3	Chr11	47.887	142	60	3	18	159	2626211
tr|A0A0H3	Chr5	62.5	96	35	1	64	159	21499987
tr|A0A0H3	Chr5	70.732	41	12	0	59	99	24069172
tr|A0A0H3	Chr5	66.667	27	9	0	99	125	24068958
tr|A0A0H3	Chr5	57.143	28	12	0	121	148	24068455
tr|A0A0H3	Chr4	62.5	96	35	1	64	159	20739869
tr|A0A0H3	Chr4	38.854	157	35	3	64	159	22463251

tr|A0A0H3	Chr12	47.183	142	61	3	18	159	12808412
tr|A0A0H3	Chr12	44.755	143	65	3	18	160	12792856
tr|A0A0H3	Chr2	43.86	114	33	2	77	159	15225499
tr|A0A0H3	Chr2	66.667	48	16	0	112	159	15204269
tr|A0A0H3	Chr2	56.667	30	13	0	77	106	15204468
tr|A0A0H3	Chr2	33.333	81	38	4	81	159	20134941
tr|A0A0H3	Chr2	37.288	59	35	1	64	120	20134900
tr|A0A0H3	Chr2	21.739	46	36	0	79	124	26040846
tr|A0A0H3	Chr3	70.833	48	14	0	112	159	15647654
tr|A0A0H3	Chr3	42.857	35	20	0	78	112	15647458
tr|A0A0H3	Chr3	29.508	61	43	0	96	156	16794415

TBLASTN 2.10.1+
Query: tr|A0A0H3VKX2|A0A0H3VKX2_LUZMU Centromeric histone H3 isoform B (Fragment) O
Database: Nippon
Fields: query acc.ver, subject acc.ver, % identity, alignment length, mismatches, gap opens, q. s
24 hits found

tr|A0A0H3	Chr1	62.5	96	35	1	70	165	37514005
tr|A0A0H3	Chr6	62.5	96	35	1	70	165	3040837
tr|A0A0H3	Chr6	62.5	96	35	1	70	165	3056279
tr|A0A0H3	Chr6	62.5	96	35	1	70	165	3055514
tr|A0A0H3	Chr6	62.5	96	35	1	70	165	3046867
tr|A0A0H3	Chr6	63.542	96	34	1	70	165	1646006
tr|A0A0H3	Chr11	62.5	96	35	1	70	165	2626112
tr|A0A0H3	Chr5	62.5	96	35	1	70	165	21499987
tr|A0A0H3	Chr5	70.732	41	12	0	65	105	24069172
tr|A0A0H3	Chr5	66.667	27	9	0	105	131	24068958
tr|A0A0H3	Chr5	57.143	28	12	0	127	154	24068455
tr|A0A0H3	Chr4	62.5	96	35	1	70	165	20739869
tr|A0A0H3	Chr4	38.854	157	35	3	70	165	22463251
tr|A0A0H3	Chr12	53.448	116	45	2	50	165	12808349
tr|A0A0H3	Chr12	56.701	97	41	1	70	166	12792757
tr|A0A0H3	Chr2	43.86	114	33	2	83	165	15225499
tr|A0A0H3	Chr2	66.667	48	16	0	118	165	15204269
tr|A0A0H3	Chr2	56.667	30	13	0	83	112	15204468
tr|A0A0H3	Chr2	33.333	81	38	4	87	165	20134941
tr|A0A0H3	Chr2	37.288	59	35	1	70	126	20134900
tr|A0A0H3	Chr2	21.739	46	36	0	85	130	26040846
tr|A0A0H3	Chr3	70.833	48	14	0	118	165	15647654
tr|A0A0H3	Chr3	42.857	35	20	0	84	118	15647458
tr|A0A0H3	Chr3	29.508	61	43	0	102	162	16794415

TBLASTN 2.10.1+
Query: tr|A0A0H3VKW9|A0A0H3VKW9_9POAL Centromeric histone H3 isoform B (Fragment) (
Database: Nippon
Fields: query acc.ver, subject acc.ver, % identity, alignment length, mismatches, gap opens, q. s
24 hits found

tr|A0A0H3	Chr1	62.5	96	35	1	70	165	37514005
tr|A0A0H3	Chr6	62.5	96	35	1	70	165	3056279
tr|A0A0H3	Chr6	62.5	96	35	1	70	165	3040837

tr|A0A0H3	Chr6	62.5	96	35	1	70	165	3055514
tr|A0A0H3	Chr6	62.5	96	35	1	70	165	3046867
tr|A0A0H3	Chr6	58.182	110	37	2	56	165	1646024
tr|A0A0H3	Chr5	62.5	96	35	1	70	165	21499987
tr|A0A0H3	Chr5	70.732	41	12	0	65	105	24069172
tr|A0A0H3	Chr5	66.667	27	9	0	105	131	24068958
tr|A0A0H3	Chr5	57.143	28	12	0	127	154	24068455
tr|A0A0H3	Chr11	62.5	96	35	1	70	165	2626112
tr|A0A0H3	Chr4	62.5	96	35	1	70	165	20739869
tr|A0A0H3	Chr4	38.854	157	35	3	70	165	22463251
tr|A0A0H3	Chr12	59.375	96	38	1	70	165	12808313
tr|A0A0H3	Chr12	56.701	97	41	1	70	166	12792757
tr|A0A0H3	Chr2	43.86	114	33	2	83	165	15225499
tr|A0A0H3	Chr2	66.667	48	16	0	118	165	15204269
tr|A0A0H3	Chr2	56.667	30	13	0	83	112	15204468
tr|A0A0H3	Chr2	33.333	81	38	4	87	165	20134941
tr|A0A0H3	Chr2	37.288	59	35	1	70	126	20134900
tr|A0A0H3	Chr2	21.739	46	36	0	85	130	26040846
tr|A0A0H3	Chr3	70.833	48	14	0	118	165	15647654
tr|A0A0H3	Chr3	42.857	35	20	0	84	118	15647458
tr|A0A0H3	Chr3	29.508	61	43	0	102	162	16794415

TBLASTN 2.10.1+
Query: tr|A0A0H3VKW0|A0A0H3VKW0_9POAL Centromeric histone H3 isoform A (Fragment) (
Database: Nippon
Fields: query acc.ver, subject acc.ver, % identity, alignment length, mismatches, gap opens, q. s
25 hits found

tr|A0A0H3	Chr1	60.417	96	37	1	66	161	37514005
tr|A0A0H3	Chr1	42.857	42	23	1	75	115	4816782
tr|A0A0H3	Chr6	60.417	96	37	1	66	161	3056279
tr|A0A0H3	Chr6	62.5	96	35	1	66	161	1646006
tr|A0A0H3	Chr6	60.417	96	37	1	66	161	3040837
tr|A0A0H3	Chr6	60.417	96	37	1	66	161	3055514
tr|A0A0H3	Chr6	60.417	96	37	1	66	161	3046867
tr|A0A0H3	Chr4	60.417	96	37	1	66	161	20739869
tr|A0A0H3	Chr4	38.217	157	36	3	66	161	22463251
tr|A0A0H3	Chr11	60.417	96	37	1	66	161	2626112
tr|A0A0H3	Chr5	60.417	96	37	1	66	161	21499987
tr|A0A0H3	Chr5	72.5	40	11	0	62	101	24069169
tr|A0A0H3	Chr5	54.545	33	13	1	101	131	24068958
tr|A0A0H3	Chr5	44.737	38	21	0	115	152	24068479
tr|A0A0H3	Chr5	35.556	45	27	1	103	147	11619317
tr|A0A0H3	Chr5	32.895	76	47	1	76	147	2715650
tr|A0A0H3	Chr12	48.031	127	63	2	35	161	12808400
tr|A0A0H3	Chr12	54.639	97	43	1	66	162	12792757
tr|A0A0H3	Chr3	66.667	48	16	0	114	161	15647654
tr|A0A0H3	Chr3	64	25	9	0	80	104	15647458
tr|A0A0H3	Chr3	57.143	21	9	0	61	81	15647310
tr|A0A0H3	Chr2	42.982	114	34	2	79	161	15225499

tr|A0A0H3	Chr2	62.5	48	18	0	114	161	15204269
tr|A0A0H3	Chr2	51.351	37	16	1	79	113	15204468
tr|A0A0H3	Chr2	36.066	61	35	2	66	123	20134900

TBLASTN 2.10.1+
Query: tr|A0A0H3VKW6|A0A0H3VKW6_9POAL Centromeric histone H3 isoform B (Fragment) C
Database: Nippon
Fields: query acc.ver, subject acc.ver, % identity, alignment length, mismatches, gap opens, q. s
24 hits found

tr|A0A0H3	Chr1	62.5	96	35	1	75	170	37514005
tr|A0A0H3	Chr6	62.5	96	35	1	75	170	3040837
tr|A0A0H3	Chr6	62.5	96	35	1	75	170	3056279
tr|A0A0H3	Chr6	62.5	96	35	1	75	170	3055514
tr|A0A0H3	Chr6	62.5	96	35	1	75	170	3046867
tr|A0A0H3	Chr6	58.182	110	37	2	61	170	1646024
tr|A0A0H3	Chr11	62.5	96	35	1	75	170	2626112
tr|A0A0H3	Chr5	62.5	96	35	1	75	170	21499987
tr|A0A0H3	Chr5	70.732	41	12	0	70	110	24069172
tr|A0A0H3	Chr5	66.667	27	9	0	110	136	24068958
tr|A0A0H3	Chr5	57.143	28	12	0	132	159	24068455
tr|A0A0H3	Chr4	62.5	96	35	1	75	170	20739869
tr|A0A0H3	Chr4	39.241	158	33	4	75	170	22463251
tr|A0A0H3	Chr12	59.375	96	38	1	75	170	12808313
tr|A0A0H3	Chr12	57.732	97	40	1	75	171	12792757
tr|A0A0H3	Chr2	43.86	114	33	2	88	170	15225499
tr|A0A0H3	Chr2	66.667	48	16	0	123	170	15204269
tr|A0A0H3	Chr2	51.351	37	16	1	88	122	15204468
tr|A0A0H3	Chr2	33.333	81	38	4	92	170	20134941
tr|A0A0H3	Chr2	37.288	59	35	1	75	131	20134900
tr|A0A0H3	Chr2	21.739	46	36	0	90	135	26040846
tr|A0A0H3	Chr3	70.833	48	14	0	123	170	15647654
tr|A0A0H3	Chr3	42.857	35	20	0	89	123	15647458
tr|A0A0H3	Chr3	27.869	61	44	0	107	167	16794415

TBLASTN 2.10.1+
Query: tr|A0A0H3VKV9|A0A0H3VKV9_9POAL Centromeric histone H3 isoform B (Fragment) OS
Database: Nippon
Fields: query acc.ver, subject acc.ver, % identity, alignment length, mismatches, gap opens, q. s
24 hits found

tr|A0A0H3	Chr1	62.5	96	35	1	70	165	37514005
tr|A0A0H3	Chr6	62.5	96	35	1	70	165	3056279
tr|A0A0H3	Chr6	62.5	96	35	1	70	165	3040837
tr|A0A0H3	Chr6	62.5	96	35	1	70	165	3055514
tr|A0A0H3	Chr6	62.5	96	35	1	70	165	3046867
tr|A0A0H3	Chr6	63.542	96	34	1	70	165	1646006
tr|A0A0H3	Chr5	62.5	96	35	1	70	165	21499987
tr|A0A0H3	Chr5	70.732	41	12	0	65	105	24069172
tr|A0A0H3	Chr5	66.667	27	9	0	105	131	24068958
tr|A0A0H3	Chr5	57.143	28	12	0	127	154	24068455
tr|A0A0H3	Chr11	62.5	96	35	1	70	165	2626112

tr|A0A0H3	Chr4	62.5	96	35	1	70	165	20739869
tr|A0A0H3	Chr4	37.58	157	37	2	70	165	22463251
tr|A0A0H3	Chr12	59.375	96	38	1	70	165	12808313
tr|A0A0H3	Chr12	57.732	97	40	1	70	166	12792757
tr|A0A0H3	Chr2	43.86	114	33	2	83	165	15225499
tr|A0A0H3	Chr2	66.667	48	16	0	118	165	15204269
tr|A0A0H3	Chr2	51.351	37	16	1	83	117	15204468
tr|A0A0H3	Chr2	43.182	44	15	2	124	165	20135034
tr|A0A0H3	Chr2	45.714	35	19	0	70	104	20134900
tr|A0A0H3	Chr2	21.739	46	36	0	85	130	26040846
tr|A0A0H3	Chr3	70.833	48	14	0	118	165	15647654
tr|A0A0H3	Chr3	42.857	35	20	0	84	118	15647458
tr|A0A0H3	Chr3	29.508	61	43	0	102	162	16794415

TBLASTN 2.10.1+
Query: tr|A0A0G3YLA7|A0A0G3YLA7_TRIUA Centromeric histone H3 OS=Triticum urartu OX=4
Database: Nippon
Fields: query acc.ver, subject acc.ver, % identity, alignment length, mismatches, gap opens, q. s
26 hits found

tr|A0A0G3	Chr6	64.286	98	33	2	52	149	3040828
tr|A0A0G3	Chr6	64.286	98	33	2	52	149	3055505
tr|A0A0G3	Chr6	64.286	98	33	2	52	149	3056288
tr|A0A0G3	Chr6	64.286	98	33	2	52	149	3046858
tr|A0A0G3	Chr6	63.265	98	34	2	52	149	1645997
tr|A0A0G3	Chr11	64.286	98	33	2	52	149	2626103
tr|A0A0G3	Chr5	64.286	98	33	2	52	149	21499978
tr|A0A0G3	Chr5	74.286	35	9	0	50	84	24069154
tr|A0A0G3	Chr5	46.429	28	14	1	84	111	24068958
tr|A0A0G3	Chr5	74.194	31	8	0	104	134	24068464
tr|A0A0G3	Chr5	45.455	33	18	0	119	151	24068062
tr|A0A0G3	Chr4	64.286	98	33	2	52	149	20739878
tr|A0A0G3	Chr4	39.13	161	36	4	50	149	22463254
tr|A0A0G3	Chr1	63.265	98	34	2	50	147	37514008
tr|A0A0G3	Chr1	28.571	49	35	0	96	144	29575612
tr|A0A0G3	Chr12	58.163	98	39	1	52	149	12808304
tr|A0A0G3	Chr12	58.163	98	39	1	52	149	12792748
tr|A0A0G3	Chr3	65.385	52	18	0	99	150	15647657
tr|A0A0G3	Chr3	59.375	32	13	0	63	94	15647458
tr|A0A0G3	Chr3	58.824	17	7	0	50	66	15647328
tr|A0A0G3	Chr2	45.378	119	33	4	62	149	15225499
tr|A0A0G3	Chr2	57.627	59	22	1	94	149	15204290
tr|A0A0G3	Chr2	65.385	26	9	0	62	87	15204468
tr|A0A0G3	Chr2	80	15	3	0	52	66	20134909
tr|A0A0G3	Chr2	39.13	46	18	2	106	149	20135040
tr|A0A0G3	Chr10	30.556	36	25	0	77	112	18302971

TBLASTN 2.10.1+
Query: tr|G1C1P4|G1C1P4_HORMA Beta centromeric histone H3 (Fragment) OS=Hordeum ma
Database: Nippon
Fields: query acc.ver, subject acc.ver, % identity, alignment length, mismatches, gap opens, q. s

23 hits found

tr|G1C1P4	Chr6	65.934	91	29	2	1	91	3040807
tr|G1C1P4	Chr6	65.934	91	29	2	1	91	3056309
tr|G1C1P4	Chr6	65.934	91	29	2	1	91	3055484
tr|G1C1P4	Chr6	65.934	91	29	2	1	91	3046837
tr|G1C1P4	Chr6	65.934	91	29	2	1	91	1645976
tr|G1C1P4	Chr11	65.934	91	29	2	1	91	2626082
tr|G1C1P4	Chr5	65.934	91	29	2	1	91	21499957
tr|G1C1P4	Chr5	75	28	7	0	49	76	24068455
tr|G1C1P4	Chr5	84.615	26	4	0	1	26	24069127
tr|G1C1P4	Chr5	42.857	28	15	1	26	53	24068958
tr|G1C1P4	Chr5	51.724	29	7	1	65	93	24068029
tr|G1C1P4	Chr4	65.934	91	29	2	1	91	20739899
tr|G1C1P4	Chr4	48.305	118	29	3	5	91	22463383
tr|G1C1P4	Chr1	66.292	89	28	2	1	89	37514035
tr|G1C1P4	Chr1	26.531	49	36	0	38	86	29575612
tr|G1C1P4	Chr12	61.538	91	33	1	1	91	12792727
tr|G1C1P4	Chr12	60.44	91	34	1	1	91	12808283
tr|G1C1P4	Chr2	46.667	120	32	3	4	92	15225499
tr|G1C1P4	Chr2	50.704	71	29	1	22	92	15204305
tr|G1C1P4	Chr2	73.077	26	7	0	4	29	15204468
tr|G1C1P4	Chr2	38	50	21	2	44	91	20135028
tr|G1C1P4	Chr3	66.667	51	17	0	41	91	15647657
tr|G1C1P4	Chr3	65.625	32	11	0	5	36	15647458

TBLASTN 2.10.1+
Query: tr|A0A0A9F0R2|A0A0A9F0R2_ARUDO CenH3 OS=Arundo donax OX=35708 PE=3 SV=1
Database: Nippon
Fields: query acc.ver, subject acc.ver, % identity, alignment length, mismatches, gap opens, q. s
22 hits found

tr|A0A0A9	Chr6	50.538	93	42	2	32	123	1646006
tr|A0A0A9	Chr6	49.462	93	43	2	32	123	3040837
tr|A0A0A9	Chr6	49.462	93	43	2	32	123	3046867
tr|A0A0A9	Chr6	49.462	93	43	2	32	123	3055514
tr|A0A0A9	Chr6	49.462	93	43	2	32	123	3056279
tr|A0A0A9	Chr5	49.462	93	43	2	32	123	21499987
tr|A0A0A9	Chr5	70.213	47	13	1	21	67	24069187
tr|A0A0A9	Chr5	68.182	22	6	1	71	92	24068946
tr|A0A0A9	Chr5	45.946	37	14	1	52	82	21481350
tr|A0A0A9	Chr11	49.462	93	43	2	32	123	2626112
tr|A0A0A9	Chr11	37.5	32	20	0	71	102	13565402
tr|A0A0A9	Chr11	45.946	37	14	1	52	82	25104229
tr|A0A0A9	Chr4	49.462	93	43	2	32	123	20739869
tr|A0A0A9	Chr4	30.263	152	46	2	32	123	22463251
tr|A0A0A9	Chr1	49.462	93	43	2	32	123	37514005
tr|A0A0A9	Chr12	46.237	93	46	2	32	123	12808313
tr|A0A0A9	Chr12	45.161	93	47	2	32	123	12792757
tr|A0A0A9	Chr3	44.186	43	24	0	81	123	15647654
tr|A0A0A9	Chr3	45.946	37	17	2	46	81	15647458

tr|A0A0A9	Chr3	85.714	14	2	0	32	45	15647325
tr|A0A0A9	Chr3	36.508	63	38	1	20	82	2880168
tr|A0A0A9	Chr2	48.718	39	20	0	31	69	20134897

TBLASTN 2.10.1+
Query: tr|A0A0A9RZT3|A0A0A9RZT3_ARUDO CenH3 OS=Arundo donax OX=35708 PE=3 SV=1
Database: Nippon
Fields: query acc.ver, subject acc.ver, % identity, alignment length, mismatches, gap opens, q. s
34 hits found

tr|A0A0A9	Chr4	39.844	128	74	1	222	346	16554942
tr|A0A0A9	Chr4	51.111	90	43	1	29	118	20739854
tr|A0A0A9	Chr4	32.099	162	47	4	20	118	22463203
tr|A0A0A9	Chr4	42	50	23	1	358	401	16555650
tr|A0A0A9	Chr4	30.682	88	48	2	316	390	21808501
tr|A0A0A9	Chr4	30	80	52	2	316	392	11687220
tr|A0A0A9	Chr4	32.468	77	48	2	319	392	25699561
tr|A0A0A9	Chr1	51.136	88	42	1	31	118	37513996
tr|A0A0A9	Chr1	31.25	80	51	2	316	392	40113946
tr|A0A0A9	Chr1	31.25	80	51	2	316	392	24681489
tr|A0A0A9	Chr11	51.111	90	43	1	29	118	2626127
tr|A0A0A9	Chr11	31.25	80	51	2	316	392	1480527
tr|A0A0A9	Chr6	51.111	90	43	1	29	118	3046882
tr|A0A0A9	Chr6	52.273	88	41	1	31	118	1646015
tr|A0A0A9	Chr6	51.111	90	43	1	29	118	3040852
tr|A0A0A9	Chr6	51.111	90	43	1	29	118	3055529
tr|A0A0A9	Chr6	51.111	90	43	1	29	118	3056264
tr|A0A0A9	Chr5	51.111	90	43	1	29	118	21500002
tr|A0A0A9	Chr5	55.814	43	19	0	27	69	24069178
tr|A0A0A9	Chr5	62.963	27	10	0	69	95	24068958
tr|A0A0A9	Chr5	46.154	52	23	1	69	120	24068506
tr|A0A0A9	Chr12	42.453	106	59	2	13	118	12808373
tr|A0A0A9	Chr12	42.453	106	59	2	13	118	12792817
tr|A0A0A9	Chr3	57.895	38	16	0	81	118	15647651
tr|A0A0A9	Chr3	52	25	12	0	48	72	15647458
tr|A0A0A9	Chr3	57.895	19	8	0	31	49	15647316
tr|A0A0A9	Chr3	32.5	80	50	2	316	392	30807
tr|A0A0A9	Chr3	32.5	80	50	2	316	392	27307918
tr|A0A0A9	Chr2	52.632	38	18	0	81	118	15204272
tr|A0A0A9	Chr2	53.846	26	12	0	47	72	15204468
tr|A0A0A9	Chr2	33.01	103	38	1	47	118	15225499
tr|A0A0A9	Chr2	31.25	80	51	2	316	392	17476403
tr|A0A0A9	Chr7	28.916	83	52	2	15	93	18492869
tr|A0A0A9	Chr8	29.412	85	56	2	311	392	1103493

TBLASTN 2.10.1+
Query: tr|A0A0K0PMX0|A0A0K0PMX0_LOLPR CENH3 (Fragment) OS=Lolium perenne OX=4522
Database: Nippon
Fields: query acc.ver, subject acc.ver, % identity, alignment length, mismatches, gap opens, q. s
17 hits found

tr|A0A0K0	Chr6	94.444	18	1	0	61	78	3040840

<table>
<tr><td>tr|A0A0KC</td><td>Chr6</td><td>94.444</td><td>18</td><td>1</td><td>0</td><td>61</td><td>78</td><td>3055517</td></tr>
<tr><td>tr|A0A0KC</td><td>Chr6</td><td>94.444</td><td>18</td><td>1</td><td>0</td><td>61</td><td>78</td><td>3046870</td></tr>
<tr><td>tr|A0A0KC</td><td>Chr6</td><td>94.444</td><td>18</td><td>1</td><td>0</td><td>61</td><td>78</td><td>3056276</td></tr>
<tr><td>tr|A0A0KC</td><td>Chr6</td><td>88.889</td><td>18</td><td>2</td><td>0</td><td>61</td><td>78</td><td>1646009</td></tr>
<tr><td>tr|A0A0KC</td><td>Chr11</td><td>94.444</td><td>18</td><td>1</td><td>0</td><td>61</td><td>78</td><td>2626115</td></tr>
<tr><td>tr|A0A0KC</td><td>Chr12</td><td>94.444</td><td>18</td><td>1</td><td>0</td><td>61</td><td>78</td><td>12808316</td></tr>
<tr><td>tr|A0A0KC</td><td>Chr12</td><td>77.778</td><td>18</td><td>4</td><td>0</td><td>61</td><td>78</td><td>12792760</td></tr>
<tr><td>tr|A0A0KC</td><td>Chr5</td><td>94.444</td><td>18</td><td>1</td><td>0</td><td>61</td><td>78</td><td>21499990</td></tr>
<tr><td>tr|A0A0KC</td><td>Chr5</td><td>76.19</td><td>21</td><td>5</td><td>0</td><td>58</td><td>78</td><td>24069169</td></tr>
<tr><td>tr|A0A0KC</td><td>Chr5</td><td>45.161</td><td>31</td><td>17</td><td>0</td><td>1</td><td>31</td><td>24070111</td></tr>
<tr><td>tr|A0A0KC</td><td>Chr1</td><td>94.444</td><td>18</td><td>1</td><td>0</td><td>61</td><td>78</td><td>37514002</td></tr>
<tr><td>tr|A0A0KC</td><td>Chr4</td><td>94.444</td><td>18</td><td>1</td><td>0</td><td>61</td><td>78</td><td>20739866</td></tr>
<tr><td>tr|A0A0KC</td><td>Chr4</td><td>72.222</td><td>18</td><td>5</td><td>0</td><td>61</td><td>78</td><td>22463248</td></tr>
<tr><td>tr|A0A0KC</td><td>Chr2</td><td>83.333</td><td>18</td><td>3</td><td>0</td><td>61</td><td>78</td><td>20134897</td></tr>
<tr><td>tr|A0A0KC</td><td>Chr2</td><td>76.471</td><td>17</td><td>4</td><td>0</td><td>61</td><td>77</td><td>15225772</td></tr>
<tr><td>tr|A0A0KC</td><td>Chr3</td><td>76.471</td><td>17</td><td>4</td><td>0</td><td>61</td><td>77</td><td>15647322</td></tr>
</table>

TBLASTN 2.10.1+
Query: tr|A0A0K0PMV8|A0A0K0PMV8_9POAL CENH3 (Fragment) OS=Glyceria maxima OX=388
Database: Nippon
0 hits found
TBLASTN 2.10.1+
Query: tr|A0A0K0PMC2|A0A0K0PMC2_9POAL CENH3 (Fragment) OS=Glyceria lithuanica OX=11
Database: Nippon
0 hits found
TBLASTN 2.10.1+
Query: tr|A0A0K0PNL1|A0A0K0PNL1_9POAL CENH3 (Fragment) OS=Glyceria alnasteretum OX=
Database: Nippon
0 hits found
TBLASTN 2.10.1+
Query: tr|A0A0K0PMD0|A0A0K0PMD0_9POAL CENH3.M2 (Fragment) OS=Melica nutans OX=86
Database: Nippon
Fields: query acc.ver, subject acc.ver, % identity, alignment length, mismatches, gap opens, q. s
1 hits found

<table>
<tr><td>tr|A0A0KC</td><td>Chr5</td><td>77.778</td><td>18</td><td>2</td><td>1</td><td>1</td><td>16</td><td>24070111</td></tr>
</table>

TBLASTN 2.10.1+
Query: tr|A0A0K0PMC7|A0A0K0PMC7_9POAL CENH3 (Fragment) OS=Glyceria triflora OX=1104
Database: Nippon
Fields: query acc.ver, subject acc.ver, % identity, alignment length, mismatches, gap opens, q. s
12 hits found

<table>
<tr><td>tr|A0A0KC</td><td>Chr11</td><td>92.857</td><td>14</td><td>1</td><td>0</td><td>26</td><td>39</td><td>2626103</td></tr>
<tr><td>tr|A0A0KC</td><td>Chr6</td><td>92.857</td><td>14</td><td>1</td><td>0</td><td>26</td><td>39</td><td>3055505</td></tr>
<tr><td>tr|A0A0KC</td><td>Chr6</td><td>92.857</td><td>14</td><td>1</td><td>0</td><td>26</td><td>39</td><td>3046858</td></tr>
<tr><td>tr|A0A0KC</td><td>Chr6</td><td>85.714</td><td>14</td><td>2</td><td>0</td><td>26</td><td>39</td><td>1645997</td></tr>
<tr><td>tr|A0A0KC</td><td>Chr6</td><td>92.857</td><td>14</td><td>1</td><td>0</td><td>26</td><td>39</td><td>3056288</td></tr>
<tr><td>tr|A0A0KC</td><td>Chr6</td><td>92.857</td><td>14</td><td>1</td><td>0</td><td>26</td><td>39</td><td>3040828</td></tr>
<tr><td>tr|A0A0KC</td><td>Chr1</td><td>92.857</td><td>14</td><td>1</td><td>0</td><td>26</td><td>39</td><td>37514014</td></tr>
<tr><td>tr|A0A0KC</td><td>Chr5</td><td>92.857</td><td>14</td><td>1</td><td>0</td><td>26</td><td>39</td><td>24069148</td></tr>
<tr><td>tr|A0A0KC</td><td>Chr5</td><td>92.857</td><td>14</td><td>1</td><td>0</td><td>26</td><td>39</td><td>21499978</td></tr>
</table>

tr|A0A0K0	Chr2	85.714	14	2	0	26	39	20134909
tr|A0A0K0	Chr12	85.714	14	2	0	26	39	12808304
tr|A0A0K0	Chr4	92.857	14	1	0	26	39	20739878

TBLASTN 2.10.1+
Query: tr|A0A0A9IDC5|A0A0A9IDC5_ARUDO CenH3 OS=Arundo donax OX=35708 PE=4 SV=1
Database: Nippon
Fields: query acc.ver, subject acc.ver, % identity, alignment length, mismatches, gap opens, q. s
6 hits found

tr|A0A0A9	Chr3	45.161	31	16	1	8	37	16368281
tr|A0A0A9	Chr12	38.235	34	21	0	1	34	5339559
tr|A0A0A9	Chr5	46.875	32	17	0	3	34	15198538
tr|A0A0A9	Chr5	41.667	24	14	0	12	35	13369016
tr|A0A0A9	Chr8	46.429	28	13	1	19	44	18557631
tr|A0A0A9	Chr8	42.308	26	15	0	12	37	5362996

TBLASTN 2.10.1+
Query: tr|A0A0K0PME4|A0A0K0PME4_GLYFL CENH3 (Fragment) OS=Glyceria fluitans OX=2547
Database: Nippon
0 hits found
TBLASTN 2.10.1+
Query: tr|A0A0K0PMW4|A0A0K0PMW4_9POAL CENH3 (Fragment) OS=Melica nutans OX=8603
Database: Nippon
Fields: query acc.ver, subject acc.ver, % identity, alignment length, mismatches, gap opens, q. s
20 hits found

tr|A0A0K0	Chr5	66.667	27	8	1	23	49	24069184
tr|A0A0K0	Chr5	82.353	17	3	0	33	49	21499987
tr|A0A0K0	Chr1	82.353	17	3	0	33	49	37514005
tr|A0A0K0	Chr1	39.394	33	20	0	2	34	7848565
tr|A0A0K0	Chr1	40	25	14	1	24	48	6071688
tr|A0A0K0	Chr6	82.353	17	3	0	33	49	3040837
tr|A0A0K0	Chr6	82.353	17	3	0	33	49	3056279
tr|A0A0K0	Chr6	82.353	17	3	0	33	49	3046867
tr|A0A0K0	Chr6	82.353	17	3	0	33	49	3055514
tr|A0A0K0	Chr6	82.353	17	3	0	33	49	1646006
tr|A0A0K0	Chr11	82.353	17	3	0	33	49	2626112
tr|A0A0K0	Chr4	82.353	17	3	0	33	49	20739869
tr|A0A0K0	Chr4	64.706	17	6	0	33	49	22463251
tr|A0A0K0	Chr12	76.471	17	4	0	33	49	12808313
tr|A0A0K0	Chr2	76.471	17	4	0	33	49	20134900
tr|A0A0K0	Chr2	68.75	16	5	0	33	48	15225769
tr|A0A0K0	Chr10	58.333	24	7	1	23	46	23027325
tr|A0A0K0	Chr10	45.238	42	18	2	3	39	21059003
tr|A0A0K0	Chr3	68.75	16	5	0	33	48	15647325
tr|A0A0K0	Chr7	71.429	14	4	0	22	35	4467810

TBLASTN 2.10.1+
Query: tr|A0A0K0PME8|A0A0K0PME8_9POAL CENH3 (Fragment) OS=Pleuropogon sabinei OX=
Database: Nippon
0 hits found
TBLASTN 2.10.1+

Query: tr|A0A0A9LKJ7|A0A0A9LKJ7_ARUDO CenH3 OS=Arundo donax OX=35708 PE=4 SV=1
Database: Nippon
Fields: query acc.ver, subject acc.ver, % identity, alignment length, mismatches, gap opens, q. s
3 hits found

tr|A0A0A9	Chr5	75	16	4	0	17	32	24067962
tr|A0A0A9	Chr3	33.333	42	23	1	5	41	14301107
tr|A0A0A9	Chr9	34.884	43	24	1	6	44	18264741

TBLASTN 2.10.1+
Query: tr|A0A0A9AVD8|A0A0A9AVD8_ARUDO CenH3 OS=Arundo donax OX=35708 PE=4 SV=1
Database: Nippon
Fields: query acc.ver, subject acc.ver, % identity, alignment length, mismatches, gap opens, q. s
6 hits found

tr|A0A0A9	Chr2	40	75	28	4	10	78	21991206
tr|A0A0A9	Chr1	43.243	37	21	0	10	46	2973164
tr|A0A0A9	Chr11	33.929	56	36	1	12	67	26320707
tr|A0A0A9	Chr11	45.833	24	13	0	31	54	8612620
tr|A0A0A9	Chr6	43.243	37	21	0	10	46	29039196
tr|A0A0A9	Chr9	35.185	54	27	2	28	75	5182979

TBLASTN 2.10.1+
Query: tr|A0A0A9NFP0|A0A0A9NFP0_ARUDO CenH3 OS=Arundo donax OX=35708 PE=4 SV=1
Database: Nippon
0 hits found
TBLASTN 2.10.1+
Query: tr|A0A0A9N0H4|A0A0A9N0H4_ARUDO CenH3 OS=Arundo donax OX=35708 PE=4 SV=1
Database: Nippon
Fields: query acc.ver, subject acc.ver, % identity, alignment length, mismatches, gap opens, q. s
6 hits found

tr|A0A0A9	Chr12	37.5	40	25	0	30	69	12792467
tr|A0A0A9	Chr11	43.902	41	19	2	22	62	15559538
tr|A0A0A9	Chr4	41.379	29	17	0	29	57	20740763
tr|A0A0A9	Chr10	41.379	29	17	0	32	60	7780241
tr|A0A0A9	Chr10	51.852	27	13	0	24	50	11837721
tr|A0A0A9	Chr6	27.419	62	42	1	6	67	2815080

TBLASTN 2.10.1+
Query: tr|A0A0A9NCE1|A0A0A9NCE1_ARUDO CenH3 OS=Arundo donax OX=35708 PE=4 SV=1
Database: Nippon
Fields: query acc.ver, subject acc.ver, % identity, alignment length, mismatches, gap opens, q. s
17 hits found

tr|A0A0A9	Chr6	61.765	34	13	0	1	34	3056544
tr|A0A0A9	Chr6	57.692	26	11	0	1	26	3040572
tr|A0A0A9	Chr6	57.692	26	11	0	1	26	3046602
tr|A0A0A9	Chr6	53.846	26	12	0	1	26	3055249
tr|A0A0A9	Chr6	58.333	24	10	0	1	24	1645741
tr|A0A0A9	Chr5	54.054	37	17	0	5	41	24068370
tr|A0A0A9	Chr5	52	25	12	0	1	25	21499722
tr|A0A0A9	Chr3	56.757	37	16	0	1	37	15647778
tr|A0A0A9	Chr12	56.25	32	14	0	1	32	12792492
tr|A0A0A9	Chr12	48.649	37	19	0	1	37	12808048

tr|A0A0A9	Chr4	57.692	26	11	0	1	26	22463702
tr|A0A0A9	Chr4	56	25	11	0	1	25	20740134
tr|A0A0A9	Chr2	52	25	12	0	2	26	15225180
tr|A0A0A9	Chr2	54.167	24	11	0	2	25	15204148
tr|A0A0A9	Chr1	52	25	12	0	1	25	37514270
tr|A0A0A9	Chr1	38.462	39	23	1	3	40	32964564
tr|A0A0A9	Chr11	52	25	12	0	1	25	2625847

TBLASTN 2.10.1+
Query: tr|A0A0A9P9H2|A0A0A9P9H2_ARUDO CenH3 OS=Arundo donax OX=35708 PE=4 SV=1
Database: Nippon
0 hits found
TBLASTN 2.10.1+
Query: tr|A0A0A9LV64|A0A0A9LV64_ARUDO CenH3 OS=Arundo donax OX=35708 PE=4 SV=1
Database: Nippon
0 hits found
TBLASTN 2.10.1+
Query: tr|A0A0A9AYD3|A0A0A9AYD3_ARUDO CenH3 OS=Arundo donax OX=35708 PE=4 SV=1
Database: Nippon
Fields: query acc.ver, subject acc.ver, % identity, alignment length, mismatches, gap opens, q. s
7 hits found

tr|A0A0A9	Chr1	47.826	23	12	0	1	23	11679950
tr|A0A0A9	Chr2	40	30	18	0	3	32	21105121
tr|A0A0A9	Chr2	60	20	8	0	5	24	16516990
tr|A0A0A9	Chr12	55	20	9	0	1	20	20508492
tr|A0A0A9	Chr4	59.091	22	9	0	4	25	27774635
tr|A0A0A9	Chr3	41.379	29	14	1	5	30	18467504
tr|A0A0A9	Chr11	46.875	32	15	1	1	32	5525746

BLAST processed 148 queries

ome assembly

34.1] [location=join(1..55,167..207,315..366,492..526,609..645,730..842,1206..1283,1405..1480,15

tart, q. end, s. start, s. end, evalue, bit score

12808020	9.55E-21	91.7
12792464	2.62E-17	81.6
37514295	2.75E-20	90.5
3040544	4.86E-20	89.7
3056572	7.28E-20	89
3055221	9.90E-20	88.6
1645713	1.56E-19	88.2
3046574	2.00E-19	87.8
2625819	6.30E-20	89.4
21499694	7.71E-20	89
24069050	7.20E-07	51.6
20740162	1.75E-19	88.2
22463730	2.10E-11	64.7
15225149	3.58E-08	55.1
15204117	5.74E-07	51.6
15647806	5.32E-07	52
15647538	0.073	27.7
15647372	0.073	26.9

28.1] [location=join(161..212,310..361,947..978,1115..1233,1579..1653,2099..2174,2765..2820)] [g

tart, q. end, s. start, s. end, evalue, bit score

37514295	3.72E-28	111
25931517	7.3	30
3056569	4.39E-28	111
3040547	5.98E-28	111
3055224	8.80E-28	110
3046577	1.35E-27	110
1645716	6.79E-27	108
21499697	5.43E-28	111
24069050	4.67E-09	47.4
24068878	4.67E-09	32.3
24068366	0.33	33.9
2625822	6.34E-28	110
20740159	1.04E-27	110
22463727	1.29E-16	78.6
12808023	2.99E-24	100
12792467	7.26E-21	90.9
15647803	9.58E-16	70.5
15647562	9.58E-16	32
15225152	1.26E-15	75.9
15204120	5.89E-15	69.7

15204379	5.89E-15	30
2140312	4.8	30.4

23.1] [location=join(161..212,310..361,946..977,1114..1232,1590..1664,2109..2184,2771..2826)] [g

tart, q. end, s. start, s. end, evalue, bit score

2625822	6.74E-32	122
3040547	9.10E-32	122
3055224	9.46E-32	122
3046577	1.07E-31	121
3056569	1.24E-31	121
1645716	2.01E-31	120
21499697	9.64E-32	122
24069050	3.23E-10	52
24068878	3.23E-10	31.6
24068366	0.06	36.2
37514295	1.38E-31	121
20740159	2.24E-31	120
22463727	1.02E-21	93.2
12808023	8.23E-28	110
12792467	8.01E-24	99.4
15647803	8.63E-19	72
15647562	8.63E-19	31.2
15647372	8.63E-19	29.3
35714190	5	30.4
15204120	1.15E-15	72
15204379	1.15E-15	30
15225152	1.95E-15	75.1
20134953	0.19	28.9
20135147	0.19	23.9
26026390	5.1	30.4

17.1] [location=join(149..200,298..349,936..967,1104..1222,1568..1642,2087..2162,2750..2805)] [g

tart, q. end, s. start, s. end, evalue, bit score

37514295	9.11E-31	119
3040547	9.57E-31	119
3056569	9.66E-31	119
3055224	1.37E-30	118
3046577	2.62E-30	117
1645716	1.32E-29	115
2625822	1.02E-30	119
21499697	1.10E-30	119
24069050	3.56E-10	51.2
24068878	3.56E-10	32.3
24068366	0.11	35.4

20740159	2.59E-30	117
22463727	1.79E-18	84
12808023	2.43E-27	109
12792467	5.60E-24	99.8
15647803	5.69E-18	72
15647562	5.69E-18	32.3
15647372	5.69E-18	25.4
15225152	2.38E-16	77.8
15204120	1.71E-15	71.6
15204379	1.71E-15	30
20134953	0.14	28.9
20135147	0.14	24.3
26026390	6.1	30.4

16.1] [location=join(149..200,289..340,928..959,1096..1214,1570..1644,2093..2168,2755..2810)] [g

tart, q. end, s. start, s. end, evalue, bit score

2625822	4.43E-34	128
3040547	6.27E-34	128
3055224	6.65E-34	128
3046577	8.89E-34	127
3056569	1.35E-33	127
1645716	1.34E-32	124
21499697	9.79E-34	127
24069050	2.63E-11	55.8
24068878	2.63E-11	31.6
24068366	0.06	36.2
20740159	3.10E-33	126
22463727	2.87E-23	97.8
37514295	4.57E-33	125
12808023	3.86E-29	114
12792467	2.30E-25	103
15647803	8.18E-20	72
15647562	8.18E-20	32.7
15647372	8.18E-20	30.8
15225152	4.24E-16	77
15204120	4.80E-16	72
15204379	4.80E-16	31.6
20134953	0.032	32
20135147	0.032	23.5
2140312	5.2	30.4
15225722	5.7	30.4
26026390	5.2	30.4

15.1] [location=join(149..200,298..349,927..958,1095..1213,1569..1643,2088..2163,2657..2712)] [g

tart, q. end, s. start, s. end, evalue, bit score

2625822	3.30E-30	117
3055224	3.67E-30	117
3040547	4.73E-30	117
3046577	4.77E-30	117
3056569	5.36E-30	117
1645716	8.70E-30	116
21499697	5.11E-30	117
24069050	9.82E-10	52.4
24068878	9.82E-10	29.6
24068366	0.39	33.9
37514295	6.38E-30	116
20740159	1.20E-29	115
22463727	4.51E-20	88.6
12808023	5.59E-27	108
12792467	3.84E-23	97.4
15647803	1.79E-17	69.7
15647372	1.79E-17	29.3
15647535	1.79E-17	28.9
35714190	0.95	32.7
35730523	3.6	30.8
15225152	7.67E-15	73.6
15204120	1.49E-14	69.7
15204388	1.49E-14	28.9
20134953	0.22	28.9
20135147	0.22	23.5
9042014	4.7	30.4

12.1] [location=join(149..200,298..349,941..972,1109..1227,1584..1658,2102..2177,2764..2819)] [g

tart, q. end, s. start, s. end, evalue, bit score

2625822	5.05E-31	119
3040547	6.13E-31	119
3056569	6.75E-31	119
3055224	7.29E-31	119
3046577	9.66E-31	119
1645716	1.08E-30	119
21499697	7.02E-31	119
24069050	1.30E-09	50.1
24068878	1.30E-09	31.6
24068366	0.06	36.2
27687855	6.4	30
37514295	8.04E-31	119
20740159	1.32E-30	118
22463727	1.17E-20	90.1
12808023	8.64E-27	107
12792467	3.32E-24	100

15647803	2.97E-18	72
15647562	2.97E-18	30.8
15647372	2.97E-18	27.3
35714190	4.2	30.8
15225152	6.52E-17	79.3
15204120	1.23E-15	72
15204379	1.23E-15	30
20134953	0.39	27.3
20135147	0.39	23.9
7812287	3.8	30.8
23758929	1.9	31.6
26026390	5	30.4

11.1] [location=join(149..200,298..349,927..958,1097..1215,1571..1645,2090..2165,2749..2804)] [g

tart, q. end, s. start, s. end, evalue, bit score

2625822	1.28E-30	118
3040547	1.66E-30	118
3055224	1.69E-30	118
3046577	2.08E-30	118
3056569	2.52E-30	117
1645716	4.13E-30	117
21499697	2.04E-30	118
24069050	1.40E-10	52.4
24068878	1.40E-10	32.7
24068366	1	32.7
37514295	2.45E-30	117
20740159	4.73E-30	117
22463727	2.02E-20	89.7
12808023	2.61E-26	106
12792467	2.07E-22	95.1
15647803	4.81E-18	68.2
15647562	4.81E-18	32.3
15647372	4.81E-18	29.3
15225152	3.13E-15	74.7
15204120	1.44E-14	68.2
15204379	1.44E-14	30
20134953	0.58	28.9
20135147	0.58	21.6
7812245	5.1	30.4
2140312	9.6	29.6

895.1] [location=join(1..103,198..223,338..374,1443..1573,1667..1741,2185..2260,2642..2694)] [gb

tart, q. end, s. start, s. end, evalue, bit score

3040541	9.76E-30	116

3055218	2.33E-29	115
3056575	2.49E-29	115
3046571	5.73E-29	114
1645710	8.02E-28	111
2625816	1.41E-29	116
21499691	1.81E-29	115
24069050	5.82E-20	80.1
24068911	5.82E-20	37
24070031	1.79E-11	64.3
24068372	8.56E-07	50.8
24067964	0.013	38.5
20740165	5.96E-29	114
22463733	2.81E-16	78.2
37514295	6.01E-29	114
12808017	4.40E-24	100
12792461	3.98E-23	97.8
15225146	1.24E-13	70.5
15204114	3.27E-09	57.8
15204379	0.003	40
20134953	1	32.7
15647812	3.03E-09	57.8
15647562	7.32E-06	40.4
15647372	7.32E-06	28.5
11774869	7	30.4

894.1] [location=join(1..103,201..226,354..390,971..1101,1197..1271,1694..1769,2167..2219)] [gbk

tart, q. end, s. start, s. end, evalue, bit score

3040541	2.45E-30	118
3055218	4.21E-30	117
3056575	8.05E-30	117
3046571	1.12E-29	116
1645710	1.83E-28	113
2625816	2.83E-30	118
21499691	4.42E-30	117
24069050	1.75E-20	81.6
24068911	1.75E-20	37.4
24070031	9.14E-11	62.4
24068372	8.56E-07	50.8
24067964	0.016	38.1
20740165	1.76E-29	116
22463733	2.19E-17	81.3
37514295	2.62E-29	115
12808017	9.30E-25	102
12792461	5.83E-24	100
15225146	7.90E-14	71.2
15204114	3.12E-09	57.8

15204379	0.01	38.9
20134953	0.11	35.8
15225722	2.6	31.6
15647812	3.43E-09	57.8
15647562	2.23E-06	39.3
15647378	2.23E-06	31.6
11774869	7.4	30.4

893.1] [location=join(1..106,240..265,381..405,1067..1197,1294..1368,1444..1519,1603..1658)] [gb

tart, q. end, s. start, s. end, evalue, bit score

3040541	1.43E-31	122
3055218	2.64E-31	121
3056575	4.44E-31	120
3046571	6.67E-31	120
1645710	1.16E-29	116
2625816	1.62E-31	121
21499691	2.61E-31	121
24069050	6.48E-21	83.2
24068911	6.48E-21	37
24070031	7.33E-07	50.8
24068372	1.86E-06	49.7
24067964	0.059	36.6
20740165	1.18E-30	119
22463733	6.76E-19	85.5
37514295	1.34E-30	119
12808002	2.10E-26	107
12792446	1.81E-25	104
15225146	5.38E-15	74.3
15204114	6.69E-15	59.3
15204379	6.69E-15	40.4
20134953	0.11	35.8
15225722	2.5	31.6
15647827	5.87E-10	60.1
15647562	1.07E-06	40.4
15647372	1.07E-06	31.2
11774869	7.5	30

889.1] [location=join(1..106,237..262,378..402,1074..1204,1301..1375,1450..1525,1609..1664)] [gb

tart, q. end, s. start, s. end, evalue, bit score

3040541	1.26E-31	122
3055218	2.96E-31	120
3056575	4.49E-31	120
3046571	8.09E-31	119
1645710	1.34E-29	116

2625816	1.77E-31	121
21499691	2.69E-31	121
24069050	5.73E-21	83.6
24068911	5.73E-21	37
24070031	1.38E-08	55.8
24068372	1.86E-06	49.7
24067964	0.058	36.6
20740165	1.09E-30	119
22463733	6.83E-19	85.5
37514295	1.49E-30	119
12808002	1.80E-26	107
12792446	2.14E-25	104
15225146	5.13E-15	74.3
15204114	6.80E-15	59.7
15204379	6.80E-15	40.4
20134953	0.11	35.8
15225722	2.5	31.6
15647827	5.54E-10	60.1
15647562	1.02E-06	40.4
15647372	1.02E-06	31.6
11774869	7.2	30.4

=join(26..68,189..310,551..625,9869..9944,10062..10117)] [gbkey=CDS]

tart, q. end, s. start, s. end, evalue, bit score

1645716	9.99E-24	97.8
3040547	1.79E-23	97.1
3055224	2.05E-23	96.7
3056569	2.07E-23	96.7
3046577	3.20E-23	96.3
21499697	1.93E-23	97.1
24069050	5.93E-11	49.7
24068878	5.93E-11	35.4
24068366	0.18	33.9
2625822	2.11E-23	96.7
20740159	3.33E-23	96.3
22463727	1.90E-14	71.2
37514295	5.25E-23	95.5
12808023	1.33E-21	91.7
12792467	2.38E-18	82.4
15225152	1.93E-13	68.2
15204120	1.97E-09	57
15204379	0.02	36.6
15647803	2.42E-12	55.5
15647544	2.42E-12	34.7

=join(6..48,187..305,583..657,9709..9784,9872..9927)] [gbkey=CDS]

tart, q. end, s. start, s. end, evalue, bit score

21499718	2.21E-19	85.1
24069050	1.23E-09	47
24068878	1.23E-09	33.9
24068366	0.31	33.1
3040568	2.32E-19	85.1
3056548	2.46E-19	85.1
3055245	3.53E-19	84.7
1645737	5.79E-19	84
3046598	6.08E-19	84
37514274	3.08E-19	84.7
2625843	3.67E-19	84.7
20740138	4.00E-19	84.3
22463706	3.23E-10	58.9
358879	4.7	29.6
12808044	6.68E-18	80.9
12792488	1.50E-15	74.3
15647782	5.42E-10	47.8
15647562	5.42E-10	34.3
15225173	6.18E-10	58.2
15204141	4.35E-06	47.4
15204379	0.1	34.7
23860388	9.4	28.9

=join(6..45,184..305,585..659,9308..9383,9471..9526)] [gbkey=CDS]

tart, q. end, s. start, s. end, evalue, bit score

3056548	2.39E-22	93.6
3040568	2.43E-22	93.6
3055245	3.49E-22	93.2
1645737	3.52E-22	93.2
3046598	6.12E-22	92.4
21499718	2.53E-22	93.6
24069050	8.05E-12	54.3
24068878	8.05E-12	33.9
24068366	0.051	35.4
2625843	3.04E-22	93.6
37514274	3.66E-22	93.2
20740138	5.24E-22	92.8
22463706	5.61E-12	63.9
12808044	1.30E-20	88.6
12792488	1.84E-18	82.4
15647782	5.23E-12	48.9
15647544	5.23E-12	34.3
15647366	5.23E-12	24.6

12758389	9.9	28.9
15225173	2.06E-11	62.4
15204141	8.74E-07	49.3
15204379	0.021	36.6
20135112	4.2	30

=join(97..127,273..394,521..595,3268..3343,3633..3733)] [gbkey=CDS]

tart, q. end, s. start, s. end, evalue, bit score

2625822	8.71E-26	103
27980006	7.3	29.6
3056569	9.78E-26	103
3040547	1.04E-25	103
1645716	1.04E-25	103
3055224	1.18E-25	103
3046577	1.18E-25	103
12807990	1.21E-25	103
12792434	1.09E-21	92.4
4809283	4.7	30
21499697	1.40E-25	103
24069050	1.51E-11	53.9
24068860	1.51E-11	33.9
24068366	0.051	35.8
37514295	1.59E-25	103
20740159	1.65E-25	103
22463727	7.76E-16	75.5
15225152	4.41E-13	67.8
15204120	3.77E-12	56.2
15204379	3.77E-12	33.5
10942960	6.1	29.6
15647803	6.42E-11	61.6
15647634	0.002	35.4
15647366	0.002	23.9
9963181	6.1	29.6

=join(88..118,248..369,662..736,2209..2284,2501..2556)] [gbkey=CDS]

tart, q. end, s. start, s. end, evalue, bit score

3056569	5.33E-24	98.2
3040547	5.33E-24	98.2
3055224	7.19E-24	97.8
1645716	1.02E-23	97.4
3046577	1.04E-23	97.4
2625822	5.38E-24	98.2
12808023	7.63E-24	97.8
12792467	5.71E-20	86.7

21499697	7.78E-24	97.8
24069050	1.40E-10	52.4
24068860	1.40E-10	31.6
24068366	0.019	36.6
37514295	1.18E-23	97.4
20740159	1.34E-23	97.1
22463727	8.09E-14	69.3
15225152	1.19E-12	65.9
15204120	4.78E-11	55.5
15204388	4.78E-11	30
10942960	4.4	29.6
15647803	9.19E-11	60.5
15647634	0.023	33.1
15647366	0.023	21.9
9963181	9.8	28.9

=join(86..116,246..367,660..734,2208..2283,2500..2555)] [gbkey=CDS]

tart, q. end, s. start, s. end, evalue, bit score

3056569	1.07E-24	100
3040547	1.21E-24	100
3055224	1.44E-24	99.8
1645716	1.58E-24	99.8
3046577	1.66E-24	99.8
2625822	1.17E-24	100
12808023	1.46E-24	99.8
12792467	9.03E-21	89
21499697	1.63E-24	99.8
24069050	1.63E-11	55.8
24068860	1.63E-11	31.6
24068366	0.019	36.6
20740159	2.10E-24	99.4
22463727	9.38E-15	72
37514295	2.14E-24	99.4
15225152	8.15E-13	66.2
15204120	3.04E-11	55.5
15204382	3.04E-11	30.8
10942960	9.8	28.9
15647803	8.03E-11	60.5
15647640	0.007	33.5
15647366	0.007	23.5
9963181	9.8	28.9

=join(56..86,216..337,440..514,2117..2192,2284..2339)] [gbkey=CDS]

tart, q. end, s. start, s. end, evalue, bit score

3056569	1.23E-24	100
3040547	1.41E-24	99.8
1645716	1.60E-24	99.8
3055224	1.68E-24	99.8
3046577	1.80E-24	99.8
2625822	1.42E-24	99.8
12808023	1.68E-24	99.8
12792467	9.85E-21	89
21499697	1.69E-24	99.8
24069050	4.51E-11	54.7
24068860	4.51E-11	30.8
24068366	0.02	36.6
37514295	2.00E-24	99.4
20740159	2.29E-24	99.4
22463727	1.34E-14	71.2
15225152	3.60E-12	64.3
15204120	2.39E-11	55.5
15204385	2.39E-11	31.2
11947225	7.8	29.3
10942960	9.8	28.9
15647803	7.95E-11	60.8
15647634	0.006	33.9
15647366	0.006	23.5
9963181	9.8	28.9

=join(56..86,216..337,440..514,2117..2192,2284..2339)] [gbkey=CDS]

tart, q. end, s. start, s. end, evalue, bit score

3040547	8.42E-25	100
3056569	8.50E-25	100
1645716	9.65E-25	100
3055224	1.08E-24	100
3046577	1.45E-24	99.8
2625822	1.00E-24	100
21499697	1.25E-24	100
24069050	1.10E-10	53.5
24068860	1.10E-10	30.8
24068366	0.019	36.6
12808023	1.38E-24	100
12792467	8.27E-21	89.4
37514295	1.58E-24	99.8
20740159	1.85E-24	99.8
22463727	1.23E-14	71.6
15225152	2.96E-12	64.7
15204120	2.20E-11	55.5
15204382	2.20E-11	31.2
10942960	8.6	28.9

15647803	7.14E-11	60.8
15647640	0.007	33.9
15647366	0.007	23.1
9963181	9.8	28.9

=join(108..138,279..400,562..636,3452..3527,3766..3821)] [gbkey=CDS]

tart, q. end, s. start, s. end, evalue, bit score

2625822	1.26E-23	97.1
3040547	1.26E-23	97.1
3056569	1.43E-23	97.1
3055224	1.55E-23	97.1
3046577	2.67E-23	96.3
1645716	3.57E-23	95.9
21499697	1.84E-23	96.7
24069050	1.66E-08	46.2
24068860	1.66E-08	30.4
24068366	0.019	36.6
37514295	4.01E-23	95.9
20740159	6.03E-23	95.1
22463727	2.34E-15	73.6
12808023	7.54E-23	95.1
12792467	3.68E-19	84.3
15225152	4.56E-14	70.1
15204120	7.63E-12	57.8
15204382	7.63E-12	30.4
15647803	5.83E-11	61.2
15647640	0.025	36.2

=join(108..138,279..400,562..636,3452..3527,3766..3821)] [gbkey=CDS]

tart, q. end, s. start, s. end, evalue, bit score

2625822	1.26E-23	97.1
3040547	1.26E-23	97.1
3056569	1.43E-23	97.1
3055224	1.55E-23	97.1
3046577	2.67E-23	96.3
1645716	3.57E-23	95.9
21499697	1.84E-23	96.7
24069050	1.69E-08	45.8
24068860	1.69E-08	31.2
24068366	0.019	36.6
37514295	4.01E-23	95.9
20740159	6.03E-23	95.1
22463727	2.98E-15	73.2
12808023	7.54E-23	95.1

12792467	3.68E-19	84.3
15225152	4.56E-14	70.1
15204120	7.63E-12	57.8
15204382	7.63E-12	30.4
15647803	5.83E-11	61.2
15647640	0.025	36.2

=join(114..144,281..402,535..609,3406..3481,3717..3772)] [gbkey=CDS]

tart, q. end, s. start, s. end, evalue, bit score

2625822	2.15E-26	105
27980006	3.6	30
3040547	2.28E-26	105
3055224	2.56E-26	104
3056569	3.26E-26	104
1645716	9.76E-26	103
3046577	1.07E-25	103
21499697	3.52E-26	104
24069050	4.21E-12	55.5
24068860	4.21E-12	33.5
24068366	0.021	36.6
12808023	6.95E-26	103
12792467	4.68E-22	92.8
20740159	1.07E-25	103
22463727	4.95E-16	75.5
37514295	1.24E-25	102
15225152	2.52E-13	67.8
15204120	6.60E-12	55.1
15204382	6.60E-12	33.5
10942960	9.8	28.9
15647803	5.44E-11	61.2
15647640	0.002	35.4
15647366	0.002	23.5
9963181	9.8	28.9
23882767	9.5	28.9

=join(114..144,281..402,535..609,3406..3481,3717..3772)] [gbkey=CDS]

tart, q. end, s. start, s. end, evalue, bit score

2625822	1.91E-25	102
27980006	1.7	31.2
3040547	2.18E-25	102
3055224	2.29E-25	102
3056569	3.04E-25	101
3046577	1.13E-24	100
1645716	1.15E-24	100

21499697	3.22E-25	101
24069050	1.82E-11	55.5
24068860	1.82E-11	31.6
24068366	0.026	36.2
12808023	8.50E-25	100
12792467	6.24E-21	89.4
20740159	8.67E-25	100
22463727	9.38E-15	72
37514295	9.46E-25	100
15647803	1.39E-14	59.3
15647532	1.39E-14	33.9
15647366	1.39E-14	23.5
9963181	4.5	29.6
15225152	3.48E-14	70.1
15204120	5.18E-13	58.9
15204391	5.18E-13	33.5
10942960	4.5	29.6
23882752	1	31.6

=join(130..160,311..432,556..630,2941..3016,3291..3346)] [gbkey=CDS]

tart, q. end, s. start, s. end, evalue, bit score

3040547	2.45E-24	99.4
3056569	2.55E-24	99.4
3055224	2.81E-24	99
3046577	3.65E-24	98.6
1645716	7.55E-24	97.8
21499697	2.73E-24	99
24069050	5.26E-11	53.5
24068860	5.26E-11	32
24068366	0.019	36.6
2625822	2.86E-24	99
18696910	3	30.4
20740159	4.65E-24	98.6
22463727	3.30E-13	67.4
37514295	5.64E-24	98.2
12808023	3.57E-23	95.9
12792467	5.94E-20	86.7
15225152	8.96E-12	63.5
15204120	9.50E-11	55.1
15204370	9.50E-11	29.6
15647803	1.58E-10	59.7

=join(100..130,240..361,492..566,2828..2903,2994..3049)] [gbkey=CDS]

tart, q. end, s. start, s. end, evalue, bit score

3056569	2.33E-23	96.3
1645716	2.37E-23	96.3
3040547	2.54E-23	96.3
3055224	3.00E-23	96.3
3046577	3.78E-23	95.9
2625822	2.59E-23	96.3
12808023	3.21E-23	95.9
12792467	8.59E-20	86.3
21499697	3.30E-23	95.9
24069050	5.84E-11	53.9
24068863	5.84E-11	31.2
24068366	0.072	35
20740159	4.13E-23	95.9
22463727	9.27E-14	68.9
37514295	5.16E-23	95.5
15225152	9.59E-12	63.2
15204120	2.18E-10	54.3
15204382	2.18E-10	29.3
15647803	2.81E-10	58.9
15647634	0.021	32
15647366	0.021	23.5

=join(123..171,268..389,1598..1672,3898..3973,4767..4822)] [gbkey=CDS]

tart, q. end, s. start, s. end, evalue, bit score

2625822	5.31E-24	98.6
3040547	7.60E-24	98.2
3055224	7.68E-24	98.2
3056569	4.19E-23	95.9
3046577	2.65E-22	93.6
1645716	1.86E-21	91.3
21499697	1.84E-23	97.1
24069050	1.12E-08	54.7
24068366	0.62	32.3
20740159	4.62E-23	95.9
22463727	4.34E-09	55.8
37514295	1.89E-22	94
12808023	2.08E-19	85.5
12792467	2.24E-16	76.6
15225152	2.84E-09	56.6
15204120	1.59E-05	45.8
15204373	0.096	34.7
15647803	7.73E-06	46.6
15647562	0.026	32.7
15647366	0.026	22.3

=join(20..74,155..282,2036..2110,3866..3941,5009..5064)] [gbkey=CDS]

tart, q. end, s. start, s. end, evalue, bit score

3040547	2.64E-20	88.2
3055224	2.85E-20	88.2
3056569	5.15E-20	87.4
3046577	1.75E-19	85.9
1645716	9.36E-19	83.6
1833486	5.6	29.6
2625822	2.77E-20	88.2
21499697	3.27E-20	87.8
24069050	6.82E-07	49.7
24068366	0.88	32
10354879	7.2	29.6
20740159	6.63E-20	87
22463727	2.87E-08	53.9
37514295	8.70E-20	86.7
12808023	2.26E-16	77
12792467	5.00E-14	70.1
15225152	2.61E-08	53.9
15204120	2.63E-06	48.1
15204385	2.1	31.2
15647803	2.99E-06	48.1
15647562	0.25	33.9

=join(106..160,251..378,2114..2188,3968..4043,5071..5126)] [gbkey=CDS]

tart, q. end, s. start, s. end, evalue, bit score

3040547	6.80E-19	84.3
3056569	8.66E-19	84
3055224	1.41E-18	83.2
3046577	1.79E-18	82.8
1645716	5.26E-18	81.6
21499697	9.83E-19	83.6
24069050	4.75E-08	53.1
24068366	0.66	32.3
10354879	7.2	29.6
2625822	1.01E-18	83.6
37514295	1.01E-18	83.6
20740159	1.02E-18	83.6
22463727	7.40E-08	52.8
12808023	3.57E-16	76.3
12792467	7.23E-14	69.7
15225152	1.06E-07	52
15204120	2.49E-06	48.1
15204373	7.9	29.3
15647803	2.09E-05	45.4

15647562	9.3	29.3

=join(143..173,315..436,574..648,3469..3544,3777..3832)] [gbkey=CDS]

tart, q. end, s. start, s. end, evalue, bit score

1645716	7.85E-24	97.8
3040547	1.11E-23	97.4
3055224	1.20E-23	97.4
3056569	1.50E-23	97.1
3046577	2.35E-23	96.3
2625822	1.08E-23	97.4
27980006	9	28.9
21499697	1.94E-23	96.7
24069050	7.20E-11	49.3
24068848	7.20E-11	35.8
24068366	0.024	36.2
20740159	2.44E-23	96.3
22463727	3.22E-14	70.5
37514295	3.78E-23	95.9
30558935	2.5	30.4
12808023	6.52E-23	95.1
12792467	1.57E-19	85.5
15225152	8.95E-16	74.7
15204120	5.22E-13	60.8
15204391	5.22E-13	31.6
10942960	7.6	29.3
15647803	2.02E-11	62.4
15647640	0.66	32.3
9963181	7.6	29.3

=join(55..97,220..341,1621..1695,4522..4597,5523..5578)] [gbkey=CDS]

tart, q. end, s. start, s. end, evalue, bit score

12808023	7.00E-25	100
12792467	2.17E-20	88.2
1645716	2.03E-24	99.8
3040547	2.57E-24	99.4
3055224	3.15E-24	99
3056569	3.57E-24	99
3046577	4.92E-24	98.6
2625822	2.54E-24	99.4
21499697	3.64E-24	99
24069050	3.23E-09	46.6
24068878	3.23E-09	32.7
24068366	0.01	37.7
20740159	5.11E-24	98.6

22463727	2.00E-14	71.2
37514295	6.65E-24	98.2
15225152	8.89E-14	69.3
15204120	1.65E-13	68.6
3450725	5.3	29.6
15647803	5.35E-13	67
26238251	4.9	29.6
19897898	4.3	30

=join(38..68,190..311,450..524,2427..2502,2783..2838)] [gbkey=CDS]

tart, q. end, s. start, s. end, evalue, bit score

2625822	2.86E-18	82
3040547	3.27E-18	81.6
3055224	3.82E-18	81.6
3046577	5.07E-18	81.3
3056569	6.71E-18	80.9
1645716	1.42E-17	80.1
21499697	5.16E-18	81.3
24069050	1.70E-07	51.2
24068366	2.2	30.8
20740159	7.92E-18	80.5
22463727	6.72E-08	52.4
12808023	1.62E-17	79.7
12792467	7.08E-15	72.4
37514295	1.76E-17	79.7
15225152	1.50E-08	54.3
15204120	1.07E-04	43.1
15204382	0.23	33.5
15647803	3.05E-05	44.7
15647634	0.15	34.3

=join(38..68,190..311,450..524,2427..2502,2783..2838)] [gbkey=CDS]

tart, q. end, s. start, s. end, evalue, bit score

1645716	1.03E-22	94.7
3040547	2.03E-22	93.6
3055224	2.46E-22	93.6
3056569	2.91E-22	93.2
3046577	3.74E-22	92.8
2625822	1.82E-22	94
12808023	2.24E-22	93.6
12792467	8.01E-19	83.6
21499697	2.69E-22	93.6
24069050	1.87E-12	51.2
24068851	1.87E-12	39.3

24068366	0.66	32.3
20740159	3.63E-22	93.2
22463727	3.75E-13	67.4
34485691	5.6	29.6
37514295	5.73E-22	92.4
30558875	6.9	29.3
15225152	2.97E-13	67.8
15204120	2.19E-09	56.6
15204382	0.98	31.6
15647803	1.32E-09	57
15647634	0.1	34.7

=join(38..68,209..330,472..546,2261..2336,2565..2620)] [gbkey=CDS]

tart, q. end, s. start, s. end, evalue, bit score

1645716	2.81E-24	99
3040547	5.38E-24	98.2
3055224	5.48E-24	98.2
3056569	6.16E-24	98.2
3046577	9.44E-24	97.4
2625822	4.34E-24	98.6
27980006	8.5	28.9
21499697	6.04E-24	98.2
24069050	4.77E-11	50.8
24068848	4.77E-11	34.7
24068366	0.025	36.2
20740159	1.04E-23	97.4
22463727	1.66E-14	71.2
37514295	1.24E-23	97.1
12808023	2.62E-23	96.3
12792467	5.49E-20	86.7
15225152	1.00E-13	68.9
15204120	1.16E-10	60.1
15204382	0.3	33.1
10942960	8.8	28.9
15647803	1.98E-11	62.4
15647640	0.27	33.5
9963181	8.8	28.9

=join(38..68,210..331,471..545,2958..3033,3309..3364)] [gbkey=CDS]

tart, q. end, s. start, s. end, evalue, bit score

1645716	1.81E-22	94
3040547	1.99E-22	94
3055224	2.49E-22	93.6
3056569	2.59E-22	93.6

3046577	4.24E-22	92.8
2625822	1.90E-22	94
21499697	3.70E-22	93.2
24069050	5.07E-10	48.9
24068848	5.07E-10	33.1
24068366	0.011	37.4
20740159	4.81E-22	92.8
22463727	1.13E-12	65.9
34485685	7.1	29.3
37514295	6.63E-22	92.4
12808023	1.84E-21	90.9
12792467	6.71E-18	80.9
15225152	7.78E-14	69.3
15204120	9.97E-12	56.6
15204391	9.97E-12	31.2
10942960	5.3	29.6
15647803	1.80E-10	59.7
15647640	0.76	32
9963181	5.3	29.6

=join(38..68,210..331,452..526,3494..3569,3845..3900)] [gbkey=CDS]

tart, q. end, s. start, s. end, evalue, bit score

3040547	6.08E-17	78.2
3055224	7.38E-17	77.8
3046577	9.98E-17	77.4
3056569	1.22E-16	77.4
1645716	2.48E-16	76.3
2625822	6.38E-17	78.2
21499697	9.69E-17	77.4
24069050	1.34E-06	48.5
24068366	0.65	32.3
20740159	1.49E-16	77
22463727	9.55E-07	48.9
37514295	2.99E-16	76.3
12808023	1.13E-15	74.3
12792467	4.78E-13	67
15225152	1.03E-07	51.6
15204120	1.88E-04	42.4
15204367	1.2	31.6
15647803	4.41E-05	44.3
15647532	1.5	31.2

=join(42..72,215..336,549..623,1557..1632,1915..1970)] [gbkey=CDS]

tart, q. end, s. start, s. end, evalue, bit score

1645716	1.83E-24	99.8
3040547	4.26E-24	98.6
3055224	4.56E-24	98.6
3056569	5.93E-24	98.2
3046577	7.19E-24	97.8
16886082	2.6	30.4
2625822	3.51E-24	99
21499697	6.72E-24	97.8
24069050	1.27E-11	52.8
24068869	1.27E-11	35
24068366	0.062	35
20740159	8.99E-24	97.4
22463727	6.86E-14	69.3
37514295	1.53E-23	97.1
30558830	3.1	30.4
12808023	1.76E-23	96.7
12792467	3.99E-20	87
15225152	4.10E-14	70.1
15204120	1.80E-10	59.7
15204370	0.056	35.4
15647803	4.23E-11	61.6
15647562	0.38	32.7
1887211	5.3	29.6

=join(38..68,213..334,452..526,3607..3682,3961..4016)] [gbkey=CDS]

tart, q. end, s. start, s. end, evalue, bit score

1645716	2.97E-23	96.3
3040547	3.64E-23	95.9
3055224	4.01E-23	95.9
3056569	4.59E-23	95.5
3046577	7.76E-23	95.1
2625822	3.50E-23	95.9
27980006	8.7	28.9
21499697	6.58E-23	95.1
24069050	7.32E-11	49.3
24068851	7.32E-11	35.8
24068366	0.022	36.6
20740159	8.39E-23	94.7
22463727	1.22E-13	68.6
37514295	1.67E-22	94
30558875	3.7	30
12808023	2.28E-22	93.6
12792467	6.16E-19	84
15225152	2.40E-13	67.8
15204120	3.03E-10	58.9
15204376	1	31.6

15647803	1.01E-10	60.5
15647640	0.53	32.3

=join(42..72,215..336,548..622,3079..3154,3455..3510)] [gbkey=CDS]

tart, q. end, s. start, s. end, evalue, bit score

1645716	5.38E-24	98.2
3040547	1.07E-23	97.4
3055224	1.16E-23	97.4
3056569	1.37E-23	97.1
3046577	1.97E-23	96.7
2625822	9.44E-24	97.4
21499697	1.67E-23	96.7
24069050	3.99E-11	50.8
24068869	3.99E-11	35
24068366	0.017	37
20740159	2.24E-23	96.7
22463727	1.71E-13	68.2
37514295	3.47E-23	95.9
30558830	2.9	30.4
12808023	7.39E-23	95.1
12792467	1.65E-19	85.5
15225152	2.20E-13	67.8
15204120	1.66E-10	59.7
15204370	0.18	33.9
15647803	2.98E-11	62
15647562	1.5	31.2
1887211	2.3	30.8

=join(33..75,167..288,453..527,1327..1402,1523..1578)] [gbkey=CDS]

tart, q. end, s. start, s. end, evalue, bit score

1645716	8.59E-22	92
3040547	1.32E-21	91.7
3056569	1.46E-21	91.7
3055224	1.54E-21	91.3
3046577	2.75E-21	90.9
21499697	1.41E-21	91.7
24069050	1.02E-09	46.2
24068878	1.02E-09	34.7
24068366	0.24	33.5
2625822	1.45E-21	91.7
20740159	2.57E-21	90.9
22463727	1.00E-12	66.2
37514295	2.97E-21	90.5
12808023	1.55E-20	88.6

12792467	4.60E-17	78.6
15225152	1.04E-12	66.2
15204120	1.15E-09	57.4
15204379	0.17	33.9
15647803	1.27E-11	55.8
15647544	1.27E-11	32
21409556	6.5	29.3
19946801	8.8	28.9
14583619	10	28.9

=join(27..69,179..300,1448..1522,2923..2998,3117..3172)] [gbkey=CDS]

tart, q. end, s. start, s. end, evalue, bit score

1645716	8.78E-23	95.1
3040547	1.56E-22	94.4
3056569	1.65E-22	94.4
3055224	2.00E-22	94
3046577	3.07E-22	93.6
21499697	1.37E-22	94.4
24068921	3.93E-07	50.4
24068878	0.093	34.7
24068366	0.24	33.5
2625822	1.89E-22	94
20740159	3.13E-22	93.6
22463727	4.53E-13	67.4
37514295	3.73E-22	93.2
12808023	4.97E-21	90.1
12792467	6.11E-18	81.3
15225152	4.40E-13	67.4
15204120	2.41E-09	56.6
15204379	0.057	35.4
15647803	5.56E-12	55.1
15647562	5.56E-12	33.9

=join(26..68,188..309,517..591,3224..3299,3418..3473)] [gbkey=CDS]

tart, q. end, s. start, s. end, evalue, bit score

1645716	2.49E-24	99.4
3056569	3.06E-24	99
3040547	4.05E-24	99
3055224	5.75E-24	98.2
3046577	8.64E-24	97.8
21499697	3.86E-24	99
24069050	1.42E-10	50.1
24068878	1.42E-10	33.9
24068366	0.15	34.3

2625822	5.02E-24	98.6
20740159	5.37E-24	98.6
22463727	7.94E-15	72.4
37514295	6.65E-24	98.2
12808023	1.10E-22	94.7
12792467	4.76E-19	84.3
15647803	1.83E-13	55.8
15647562	1.83E-13	33.9
15647366	1.83E-13	23.5
21409556	9.8	28.9
15225152	4.45E-13	67.4
15204120	1.57E-09	57
15204379	0.049	35.4

=join(26..68,188..309,517..591,3224..3299,3418..3473)] [gbkey=CDS]

tart, q. end, s. start, s. end, evalue, bit score

1645716	1.70E-22	94.4
3056569	2.36E-22	93.6
3040547	2.39E-22	93.6
3055224	3.59E-22	93.2
3046577	4.11E-22	93.2
21499697	2.32E-22	94
24069014	1.32E-10	50.1
24068878	1.32E-10	33.9
24068366	5.9	29.6
20740159	3.26E-22	93.2
22463727	2.09E-13	68.2
2625822	3.29E-22	93.2
37514295	3.80E-22	93.2
12808023	4.34E-21	90.1
12792467	1.07E-17	80.5
15647803	5.62E-12	51.2
15647544	5.62E-12	33.1
15647366	5.62E-12	23.1
21409556	9.8	28.9
15225152	1.86E-11	62.8
15204120	4.67E-08	52.8
15204379	0.055	35.4

=join(26..68,189..310,549..623,2260..2335,2453..2508)] [gbkey=CDS]

tart, q. end, s. start, s. end, evalue, bit score

3040547	9.99E-24	97.8
3056569	1.30E-23	97.4
3055224	1.38E-23	97.4

1645716	1.38E-23	97.4
3046577	2.15E-23	96.7
21499697	1.10E-23	97.4
24068921	5.67E-07	49.7
24068878	0.057	35.4
24068366	0.26	33.5
2625822	1.19E-23	97.4
20740159	2.88E-23	96.3
22463727	2.86E-14	70.9
37514295	3.49E-23	96.3
12808023	7.95E-22	92.4
12792467	1.63E-18	82.8
15225152	2.85E-13	67.8
15204120	3.19E-09	56.2
15204379	0.02	36.6
15647803	2.68E-12	55.1
15647544	2.68E-12	34.7
9249244	3.7	30
19946801	9.1	28.9

=join(13..43,159..280,432..506,2692..2767,3048..3103)] [gbkey=CDS]

tart, q. end, s. start, s. end, evalue, bit score

1645716	2.55E-25	102
3056569	3.80E-25	101
3040547	3.99E-25	101
3055224	4.94E-25	101
3046577	8.75E-25	100
2625822	4.39E-25	101
21499697	4.84E-25	101
24069050	1.71E-13	57
24068851	1.71E-13	37
24068366	0.019	36.6
20740159	6.17E-25	100
22463727	9.63E-14	68.9
37514295	1.09E-24	100
30558935	7.9	29.3
12808023	1.54E-24	99.8
12792467	1.04E-20	89
9051156	6.5	29.3
15647803	1.41E-14	61.6
15647562	1.41E-14	32.3
15647366	1.41E-14	23.1
15225152	9.07E-13	66.2
15204120	1.88E-10	59.7
15204370	1.1	31.6

=join(103..133,278..399,517..591,2974..3049,3309..3364)] [gbkey=CDS]

tart, q. end, s. start, s. end, evalue, bit score

1645716	6.64E-23	95.1
3040547	1.01E-22	94.7
3055224	1.19E-22	94.4
3056569	1.34E-22	94.4
3046577	1.86E-22	94
2625822	8.98E-23	94.7
27980006	8.8	28.9
21499697	1.67E-22	94
24069050	1.69E-10	48.1
24068848	1.69E-10	35.8
24068366	0.085	34.7
12808023	1.70E-22	94
12792467	4.34E-19	84.3
20740159	2.09E-22	93.6
22463727	2.62E-13	67.8
37514295	3.30E-22	93.2
30558935	2.8	30.4
15225152	3.71E-13	67.4
15204120	1.55E-10	59.7
15204382	2.6	30.4
15647803	5.83E-11	61.2
15647634	1.8	30.8

=join(35..77,194..315,580..654,3340..3415,3533..3588)] [gbkey=CDS]

tart, q. end, s. start, s. end, evalue, bit score

12808023	1.84E-22	94
12792467	3.56E-19	84.7
21499697	3.59E-22	93.2
24069050	4.42E-10	50.8
24068878	4.42E-10	31.2
24068366	0.61	32.3
3056569	3.88E-22	93.2
3040547	4.15E-22	93.2
1645716	4.66E-22	92.8
3055224	5.29E-22	92.8
3046577	8.76E-22	92
2625822	4.85E-22	92.8
20740159	6.42E-22	92.4
22463727	4.45E-13	67.4
37514295	8.42E-22	92
15225152	2.57E-12	65.1
15204120	1.86E-09	57

15204379	2.2	30.8
15647803	5.23E-11	54.7
15647562	5.23E-11	28.9
15647366	5.23E-11	21.2

otein_id=AHW98238.1] [location=1..372] [gbkey=CDS]

tart, q. end, s. start, s. end, evalue, bit score

12808023	5.95E-26	103
12792467	2.33E-21	90.9
2625822	2.50E-25	102
3040547	2.63E-25	102
1645716	3.13E-25	102
3055224	3.28E-25	102
3056569	3.69E-25	101
3046577	5.13E-25	101
21499697	2.84E-25	102
24069050	6.62E-10	49.7
24068878	6.62E-10	32
24068366	0.012	37.4
20740159	5.93E-25	101
22463727	1.52E-14	71.6
1121350	8.4	29.3
37514295	7.94E-25	100
15225152	1.29E-14	71.6
15204120	4.15E-14	68.2
15204391	4.15E-14	27.7
28327459	9.1	28.9
15647803	1.87E-14	67
15647562	1.87E-14	27.3
15647366	1.87E-14	21.9
25284109	8	29.3

otein_id=AHW98236.1] [location=1..372] [gbkey=CDS]

tart, q. end, s. start, s. end, evalue, bit score

12808023	2.66E-26	105
12792467	9.74E-22	92
2625822	1.04E-25	103
4447083	8.5	29.3
1645716	1.08E-25	103
3040547	1.22E-25	103
3055224	1.44E-25	102
3056569	1.70E-25	102
3046577	2.38E-25	102
6305171	0.74	32.3

21499697	1.76E-25	102
24069050	5.96E-10	49.7
24068878	5.96E-10	32.3
24068366	0.006	38.5
20740159	2.58E-25	102
22463727	7.28E-15	72.4
1121350	7.6	29.3
37514295	3.69E-25	101
15225152	5.99E-15	72.8
15204120	2.25E-14	69.3
15204391	2.25E-14	27.7
15647803	1.10E-14	68.2
15647562	1.10E-14	27.3
15647366	1.10E-14	21.9

otein_id=AHW98229.1] [location=1..474] [gbkey=CDS]

tart, q. end, s. start, s. end, evalue, bit score

3040547	3.00E-22	95.1
3056569	3.82E-22	94.7
3055224	4.54E-22	94.4
3046577	8.19E-22	93.6
1645716	2.09E-21	92.4
21499697	3.40E-22	94.7
24069050	3.10E-08	50.4
24068911	3.10E-08	26.6
24068366	2.6	31.6
2625822	3.46E-22	94.7
37514295	7.88E-22	94
20740159	1.41E-21	93.2
22463727	4.85E-11	62.8
12808023	2.20E-19	86.7
12792467	5.13E-18	82.8
15225152	1.83E-09	58.2
15204120	1.21E-07	53.1
15204373	0.61	33.5
15647803	7.50E-07	50.8
15647562	0.008	37.4
15647378	0.008	20.4

otein_id=AHW98228.1] [location=1..474] [gbkey=CDS]

tart, q. end, s. start, s. end, evalue, bit score

3040547	1.31E-20	90.1
3056569	1.80E-20	89.7
3055224	2.19E-20	89.7

3046577	3.91E-20	89
1645716	5.64E-20	88.6
21499697	1.39E-20	90.1
24069050	6.47E-08	52
24068911	6.47E-08	23.9
24068366	8.9	29.6
2625822	1.55E-20	90.1
37514295	2.32E-20	89.7
20740159	3.13E-20	89.4
22463727	3.61E-10	60.5
12808023	2.60E-17	80.9
12792467	3.69E-16	77.4
15225152	3.99E-08	54.3
15204120	1.91E-06	49.7
15204373	1.4	32.3
15647803	5.00E-06	48.1
15647562	0.059	36.2

ariant protein] [protein_id=ADN92693.1] [location=153..704] [gbkey=CDS]

tart, q. end, s. start, s. end, evalue, bit score

1645713	4.73E-28	112
3040544	2.99E-27	110
3056572	3.32E-27	110
3055221	4.27E-27	110
3046574	4.84E-27	109
37514295	2.22E-27	110
21499694	3.29E-27	110
24069050	1.45E-10	51.6
24068878	1.45E-10	33.9
24068357	0.29	34.7
2625819	3.36E-27	110
20740162	3.96E-27	110
22463730	2.94E-18	84.7
12808020	6.42E-26	106
12792464	2.23E-23	99.4
15647806	3.44E-17	68.9
15647544	3.44E-17	29.6
15647378	3.44E-17	28.9
15225149	2.66E-16	79
15204117	1.09E-14	68.9
15204379	1.09E-14	30.8
24733536	9.8	30

ric protein B] [protein_id=QGY64363.1] [location=24..500] [gbkey=CDS]

tart, q. end, s. start, s. end, evalue, bit score

2625819	4.13E-27	108
3040544	4.77E-27	108
3055221	6.38E-27	108
3046574	6.83E-27	108
3056572	1.80E-26	107
1645713	1.01E-25	105
21499694	9.13E-27	107
24069050	1.06E-12	67.8
24068357	1.3	32.3
37514295	5.57E-26	105
20740162	6.50E-26	105
22463730	8.97E-17	79.3
12808020	5.20E-25	102
12792464	1.11E-22	96.3
15225149	5.53E-12	65.5
15204117	8.00E-07	50.4
15204379	0.02	37.7
20134950	1.3	32.3
15225722	4	30.8
15647806	8.47E-07	50.4
15647562	6.43E-06	38.1
15647378	6.43E-06	30.8

ric protein A] [protein_id=QGY64362.1] [location=24..500] [gbkey=CDS]

tart, q. end, s. start, s. end, evalue, bit score

2625819	5.06E-28	111
3040544	5.36E-28	111
3055221	7.45E-28	110
3046574	8.78E-28	110
3056572	1.70E-27	110
1645713	1.22E-26	107
21499694	1.14E-27	110
24069050	1.68E-13	70.1
24068357	1.2	32.3
20740162	5.85E-27	108
22463730	1.13E-17	82
37514295	5.96E-27	108
12808020	1.08E-25	104
12792464	9.00E-23	96.7
15225149	3.29E-12	66.2
15204117	9.96E-07	50.4
15204379	0.01	38.5
20134953	0.19	34.7
15225722	0.63	33.1
15647806	9.59E-07	50.4

15647562	1.45E-06	38.5
15647372	1.45E-06	32.7

ric protein 2] [protein_id=QGY64360.1] [location=106..555] [gbkey=CDS]

tart, q. end, s. start, s. end, evalue, bit score

12808020	3.53E-15	74.3
12792464	3.85E-11	62.8
2625819	4.80E-15	73.9
3055221	5.24E-15	73.9
3040544	6.00E-15	73.6
3046574	1.01E-14	73.2
1645713	1.43E-14	72.8
3056572	1.70E-14	72.4
21499694	7.42E-15	73.6
24069050	3.90E-09	57
20740162	3.87E-14	71.2
22463730	4.40E-06	48.1
37514295	7.82E-14	70.5
15647535	2.59E-04	33.5
15647378	2.59E-04	29.3
15647806	2.2	31.6
15225149	0.002	40
15204388	0.44	33.5
20135004	0.6	33.1
15204117	1.7	32

ric protein 1A] [protein_id=QGY64359.1] [location=123..569] [gbkey=CDS]

tart, q. end, s. start, s. end, evalue, bit score

2625819	2.22E-33	126
3055221	2.49E-33	126
3040544	3.21E-33	125
3056572	6.14E-33	125
3046574	8.72E-32	122
1645713	5.88E-31	119
21499694	5.91E-33	125
24069053	4.47E-12	58.9
24068878	4.47E-12	30.8
24068357	0.006	38.9
20740162	7.75E-33	124
22463730	8.89E-21	90.5
22951329	3.6	30.8
37514295	2.68E-32	123
12808020	3.30E-30	117
12792464	2.91E-25	103

15647806	1.01E-17	63.2
15647390	1.01E-17	34.3
15647535	1.01E-17	31.2
15225149	1.16E-14	72.8
15204117	1.98E-13	62
15204349	1.98E-13	32.7
20135076	0.18	34.7
15225722	0.63	33.1

ric protein 1B] [protein_id=QGY64358.1] [location=161..607] [gbkey=CDS]

tart, q. end, s. start, s. end, evalue, bit score

2625828	2.09E-33	126
3055230	2.45E-33	126
3040553	3.50E-33	125
3056563	6.96E-33	125
3046583	9.07E-32	121
1645722	5.44E-31	119
21499703	6.90E-33	125
24069053	4.15E-12	59.3
24068878	4.15E-12	30.8
24068357	0.015	37.7
20740153	1.01E-32	124
22463721	7.39E-21	90.5
22951329	2.3	31.6
37514289	2.89E-32	123
12808029	9.66E-30	115
12792479	1.16E-25	104
15647797	1.23E-17	62.8
15647381	1.23E-17	34.3
15647535	1.23E-17	31.2
15225158	2.43E-15	74.7
15204126	5.70E-14	63.5
15204349	5.70E-14	32.7
20135076	0.13	35
15225722	0.56	33.1

e H3] [protein_id=AUN88460.1] [location=1..501] [gbkey=CDS]

tart, q. end, s. start, s. end, evalue, bit score

2625816	1.18E-33	128
18024302	0.14	35.4
24024542	1.4	32.3
3040541	1.33E-33	127
3055218	2.14E-33	127
3056575	5.32E-33	126

3046571	7.11E-33	125
1645710	3.44E-32	124
27987538	1.7	32.3
16492671	2.8	31.6
14859475	4.1	31.2
23790530	5.3	30.8
21499691	2.76E-33	127
24069050	1.85E-17	69.3
24068878	1.85E-17	39.3
24068372	3.20E-06	49.3
24070067	0.091	35.8
24067964	0.57	33.5
11985457	2.2	32
7739703	2.2	32
8334114	4	31.2
11691936	4.5	30.8
7092007	5.3	30.8
20740165	2.27E-32	124
22463733	1.05E-22	96.7
6034982	1.3	32.7
6359028	9.1	30
37514295	2.98E-32	124
10092977	0.93	33.1
12808017	2.64E-29	115
12792461	1.76E-27	110
26725481	2	32
15225143	6.32E-18	82.8
15204111	2.32E-17	71.6
15204379	2.32E-17	36.6
20135004	1.37E-04	36.6
20135153	1.37E-04	27.7
15225722	2.7	31.6
15122935	5.6	30.8
15647809	5.79E-17	70.1
15647562	5.79E-17	37
15647456	1.3	32.3
19623374	1.5	32.3
19302504	2.3	32
19499674	9.1	30
6630257	2.5	31.6
9325304	3.4	31.2
11365685	3.8	31.2
13418342	6.7	30.4
7380767	6.9	30.4
7580759	3.9	31.2
4633661	5.7	30.4
9874017	6.8	30.4

ne 3] [protein_id=AMH40810.1] [location=1..504] [gbkey=CDS]

tart, q. end, s. start, s. end, evalue, bit score

3040541	9.56E-26	105
3056575	1.51E-25	104
3055218	1.71E-25	104
3046571	5.94E-25	103
1645710	3.22E-24	100
2625816	1.21E-25	105
21499691	2.54E-25	104
24069050	3.52E-12	45.4
24068878	3.52E-12	45.1
24068372	0.002	40.8
24067964	3.8	31.2
5979887	7.2	30.4
37514295	3.42E-25	103
20740165	8.99E-25	102
22463733	9.41E-17	79.7
12808017	2.32E-22	95.9
12792461	8.46E-22	94
15225146	4.15E-18	83.6
15204114	2.36E-15	69.3
15204379	2.36E-15	32.3
15647809	6.23E-15	68.9
15647538	6.23E-15	31.2

ne 3] [protein_id=AMH40809.1] [location=1..504] [gbkey=CDS]

tart, q. end, s. start, s. end, evalue, bit score

3040541	3.26E-29	115
3055218	6.41E-29	114
3056575	8.41E-29	114
3046571	3.10E-28	112
1645710	4.92E-27	108
11621097	9.3	30
2625816	4.57E-29	115
21499691	1.52E-28	113
24069050	7.68E-17	60.8
24068878	7.68E-17	45.8
24068372	0.002	40.8
24067964	4.6	30.8
5979887	7.5	30.4
37514295	2.58E-28	112
10032301	7	28.9
10032158	7	17.7
20740165	5.81E-28	111

22463733	1.07E-16	79.3
11135376	1.3	32.7
9964684	6.3	30.4
12808017	7.16E-26	105
12792461	7.00E-25	102
11492753	1.7	32.3
15225146	1.11E-15	76.6
15204114	1.52E-15	70.5
15204379	1.52E-15	32
20134950	0.076	28.9
20135159	0.076	25.8
15647809	5.56E-15	69.7
15647538	5.56E-15	30.8
22269829	2.5	31.6
22986457	4.2	31.2
27133484	5.2	30.8

3] [protein_id=AKI32619.1] [location=1..471] [gbkey=CDS]

tart, q. end, s. start, s. end, evalue, bit score

3046571	9.44E-29	113
3040541	2.07E-28	112
3055218	3.32E-28	112
3056575	4.27E-28	111
1645710	1.35E-26	107
7680274	7.4	30
2625816	2.11E-28	112
21499691	4.94E-28	111
24069050	1.27E-15	58.9
24068878	1.27E-15	43.5
24068372	0.002	40.8
24067964	4.3	30.8
20740165	2.26E-27	109
22463733	2.65E-18	83.6
11135376	3.1	31.2
21299547	7.1	30
37514295	2.37E-27	109
12808017	2.79E-26	106
12792461	3.26E-24	100
11492753	2.4	31.6
2433977	9.5	29.6
15225146	1.02E-16	79
15204114	2.67E-15	68.9
15204379	2.67E-15	32
20135159	0.033	29.3
20134950	0.033	26.6
15647809	9.18E-15	68.2

15647544	9.18E-15	30.8
22269829	1	32.7
8621132	1.3	32.3
28183754	9.6	29.6

3] [protein_id=AKI32617.1] [location=1..306] [gbkey=CDS]

tart, q. end, s. start, s. end, evalue, bit score

24069071	1.18E-04	42.4
21499916	0.4	32.3
12808188	0.006	37.4
12792632	0.079	34.3
3046796	0.012	36.6
3056404	0.04	35
3040766	0.045	35
1645881	0.19	33.1
3055443	0.75	31.6
7680274	3.1	29.6
20739994	0.048	35
2626041	0.053	34.7
37514076	0.067	34.3

3] [protein_id=AKI32616.1] [location=1..471] [gbkey=CDS]

tart, q. end, s. start, s. end, evalue, bit score

3046571	1.58E-26	107
3040541	3.32E-26	106
3056575	4.44E-26	105
3055218	4.75E-26	105
1645710	1.20E-24	101
7680274	7.2	30
2625816	2.93E-26	106
21499691	7.49E-26	105
24069050	3.23E-14	56.2
24068878	3.23E-14	41.2
24068372	0.002	40.8
37514295	1.50E-25	104
20740165	2.24E-25	103
22463733	1.33E-16	78.6
12808017	7.14E-25	102
12792461	6.34E-23	97.1
15225146	4.87E-15	74.3
15204114	7.30E-14	68.6
15204379	7.30E-14	27.7
20135153	0.2	29.3
20134950	0.2	23.5

15647809	2.57E-13	67.8
15647544	2.57E-13	26.6

3] [protein_id=AKI32612.1] [location=1..471] [gbkey=CDS]

tart, q. end, s. start, s. end, evalue, bit score

3040598	1.65E-16	78.6
3055275	2.31E-16	78.2
3056518	3.50E-16	77.4
3046628	6.19E-16	77
1645767	2.01E-15	75.5
2625873	1.68E-16	78.6
21499748	4.54E-16	77.4
24069050	2.85E-12	45.4
24068878	2.85E-12	45.4
24068366	0.84	32.7
5979887	8.9	29.6
37514241	4.63E-16	77
20740129	1.59E-15	75.5
22463697	1.10E-07	53.1
12808074	3.16E-14	72
12792518	2.79E-13	69.3
15225182	1.03E-08	56.2
15204150	1.82E-07	41.6
15204379	1.82E-07	32.3
15647749	2.30E-07	42.4
15647538	2.30E-07	31.6

3] [protein_id=AKI32608.1] [location=1..219] [gbkey=CDS]

tart, q. end, s. start, s. end, evalue, bit score

5306151	1.1	29.6
5979887	1.6	29.3
11875817	3.2	28.5
2141507	6.4	27.7
11990524	6.4	27.7
25463858	7.1	27.7
35302166	8.1	27.3
17187421	9.2	27.3

3] [protein_id=AKI32602.1] [location=1..477] [gbkey=CDS]

tart, q. end, s. start, s. end, evalue, bit score

3040541	4.09E-26	106
3056575	6.76E-26	105

3055218	8.45E-26	105
3046571	2.24E-25	103
1645710	9.76E-25	102
2625816	5.68E-26	105
21499691	1.08E-25	104
24069050	2.67E-12	45.8
24068878	2.67E-12	45.1
24068372	0.003	40
37514295	1.92E-25	104
20740165	3.47E-25	103
22463733	7.46E-17	79.7
12808017	1.86E-23	98.6
12792461	5.77E-23	97.1
15225146	1.56E-18	84.3
15204114	1.21E-15	70.1
15204379	1.21E-15	32.3
15647809	2.77E-15	69.7
15647538	2.77E-15	31.2

3] [protein_id=AKI32601.1] [location=1..471] [gbkey=CDS]

tart, q. end, s. start, s. end, evalue, bit score

3040541	7.27E-29	114
3055218	1.07E-28	113
3056575	1.24E-28	113
3046571	3.45E-28	112
1645710	3.70E-27	108
2625816	8.01E-29	113
21499691	2.07E-28	112
24069050	1.75E-15	56.6
24068878	1.75E-15	45.1
24068372	0.002	40.8
24067964	3	31.2
37514295	5.18E-28	111
20740165	8.33E-28	110
22463733	6.92E-17	79.7
12808017	5.04E-26	105
12792461	9.09E-25	102
15225146	6.57E-18	82.4
15204114	2.69E-15	69.7
15204379	2.69E-15	31.6
20134950	0.19	26.6
20135159	0.19	26.2
15647809	5.82E-15	69.3
15647562	5.82E-15	30.4

3] [protein_id=AKI32600.1] [location=1..504] [gbkey=CDS]

tart, q. end, s. start, s. end, evalue, bit score

3040541	5.34E-29	114
3056575	1.25E-28	113
3055218	1.61E-28	113
3046571	5.54E-28	111
1645710	8.46E-27	108
11621097	9.7	30
2625816	1.15E-28	114
21499691	2.37E-28	112
24069050	7.08E-17	60.8
24068851	7.08E-17	45.8
24068372	0.001	41.6
24067964	4.8	30.8
37514295	3.76E-28	112
20740165	9.07E-28	111
22463733	1.92E-16	78.6
11135376	1.2	32.7
9964684	6.1	30.4
12808017	2.86E-26	107
12792461	1.00E-24	102
11492753	1.7	32.3
15225146	5.93E-18	83.2
15204114	2.08E-15	70.1
15204379	2.08E-15	32
20134950	0.099	28.9
20135159	0.099	25.4
15647809	8.35E-15	68.9
15647538	8.35E-15	30.8
22269829	2.6	31.6
22986457	4.4	30.8
27133484	8.4	30

form b] [protein_id=BAP26971.1] [location=1..513] [gbkey=CDS]

tart, q. end, s. start, s. end, evalue, bit score

2625816	2.60E-27	110
3040541	2.86E-27	110
3055218	3.68E-27	109
3046571	4.60E-27	109
3056575	1.37E-26	107
1645710	2.44E-26	107
21499691	8.36E-27	108
24069050	5.20E-12	61.6
24068911	5.20E-12	28.9
24070046	0.001	41.6

24068372	0.009	38.9
20740165	6.06E-26	106
22463733	7.22E-16	77
37514295	1.52E-25	105
12808017	1.60E-22	96.3
12792461	1.30E-21	93.6
15225146	5.50E-10	60.1
15204111	2.62E-07	52.4
15204379	0.18	35
20134953	0.44	33.9
15647809	7.36E-07	51.2
15647562	3.95E-06	38.1
15647372	3.95E-06	31.6

form a] [protein_id=BAP26970.1] [location=1..492] [gbkey=CDS]

tart, q. end, s. start, s. end, evalue, bit score

2625816	2.03E-27	110
3040541	2.23E-27	110
3055218	2.87E-27	109
3046571	3.59E-27	109
3056575	1.07E-26	107
1645710	1.91E-26	107
21499691	6.54E-27	108
24069050	4.70E-12	61.6
24068911	4.70E-12	28.9
24070046	0.001	41.6
24068372	0.008	38.9
20740165	4.74E-26	106
22463733	5.80E-16	77
37514295	1.19E-25	105
12808017	1.26E-22	96.3
12792461	1.03E-21	93.6
15225146	4.53E-10	60.1
15204111	2.20E-07	52.4
15204379	0.16	35
20134953	0.39	33.9
15647809	6.20E-07	51.2
15647562	3.58E-06	38.1
15647372	3.58E-06	31.6

tein_id=AEH95352.1] [location=1..492] [gbkey=CDS]

tart, q. end, s. start, s. end, evalue, bit score

3040541	9.03E-33	125
3055218	1.46E-32	124

3056575	3.78E-32	123
3046571	5.05E-32	123
1645710	2.94E-31	120
27987538	1.4	32.3
16492671	1.7	32
14859475	2.7	31.6
23790530	3.5	31.2
2625816	9.56E-33	125
18024302	0.12	35.4
24024542	0.93	32.7
21499691	1.88E-32	124
24069050	8.10E-17	67
24068878	8.10E-17	39.3
24068372	6.07E-06	48.1
24070037	0.021	37.7
24067964	0.29	34.3
11985457	1.4	32.3
7739703	1.6	32.3
8334114	3	31.2
11691936	3.3	31.2
7092007	3.6	31.2
20740165	1.51E-31	122
22463733	8.14E-22	94
6034982	1	32.7
6359028	6.3	30.4
16909699	7.8	30
37514295	2.28E-31	121
10092977	0.69	33.1
12808017	7.22E-29	114
12792461	5.34E-27	108
26725481	1.5	32.3
15225143	4.40E-19	86.3
15204111	1.03E-16	71.6
15204379	1.03E-16	34.3
20135004	1.15E-04	37
20135153	1.15E-04	27.7
15225722	2.6	31.6
15122935	3.8	31.2
15647809	2.81E-16	70.1
15647562	2.81E-16	34.7
19623374	0.98	32.7
19302504	1.6	32.3
15647456	1.8	32
19499674	6.3	30.4
6630257	2	32
9325304	2.3	31.6
11365685	2.8	31.6
13418342	4.8	30.8

7380767	5	30.8
7580759	3	31.2
2881009	7.1	30.4
4633661	3.9	31.2
9874017	5.4	30.4
13449364	9	30

tein_id=AEH95350.1] [location=1..501] [gbkey=CDS]

tart, q. end, s. start, s. end, evalue, bit score

3040541	3.22E-33	126
3055218	4.92E-33	126
3056575	1.57E-32	124
3046571	1.98E-32	124
1645710	1.10E-31	122
27987538	1.7	32.3
16492671	2.6	31.6
14859475	3.7	31.2
23790530	4.8	30.8
2625816	3.28E-33	126
18024302	0.15	35.4
24024545	1.2	32.7
21499691	6.71E-33	125
24069050	3.59E-17	68.6
24068878	3.59E-17	39.3
24068372	5.05E-06	48.5
24070067	0.078	36.2
24067964	0.51	33.9
11985457	2	32
7739703	2.1	32
8334114	3.9	31.2
11691936	4.8	30.8
7092007	5.4	30.8
20740165	5.75E-32	123
22463733	2.72E-22	95.5
6034982	1.4	32.3
6359028	8.7	30
37514295	7.39E-32	122
10092977	0.85	33.1
12808017	5.62E-29	114
12792461	4.48E-27	109
26725481	2.1	32
15225143	1.41E-19	87.8
15204111	4.32E-17	71.6
15204379	4.32E-17	35.8
20135004	1.67E-04	36.2
20135153	1.67E-04	27.7

15225722	2.5	31.6
15122935	5	30.8
15647809	1.27E-16	70.1
15647562	1.27E-16	35.8
19623374	1.4	32.3
15647456	1.9	32
19302504	2.2	32
19499674	8.4	30
6630257	2.5	31.6
9325304	3	31.6
11365685	3.8	31.2
13418342	5.9	30.4
7380767	6.7	30.4
7580759	3.7	31.2
4633661	5.6	30.8
9874017	6.7	30.4

3] [protein_id=AEK21394.1] [location=1..411] [gbkey=CDS]

tart, q. end, s. start, s. end, evalue, bit score

2625828	9.83E-31	118
3055230	1.41E-30	117
3040553	1.64E-30	117
1645722	4.46E-30	116
3056563	9.78E-30	115
3046583	4.79E-29	113
21499703	3.64E-30	116
24069050	1.31E-12	66.6
24068372	1.08E-06	49.7
37514289	1.87E-29	114
20740153	2.43E-29	114
22463721	1.10E-19	86.7
31958891	0.19	34.3
12808029	5.23E-27	107
12792470	5.11E-23	96.3
3562616	0.83	32.3
3718644	6.4	29.6
15647797	6.71E-18	63.2
15647544	6.71E-18	37.4
15647372	6.71E-18	28.1
15225158	1.50E-17	80.5
15204126	2.03E-16	63.2
15204379	2.03E-16	41.2
20134959	0.27	33.9
27393628	4.8	30
4506970	4.7	30

3] [protein_id=AEK21393.1] [location=44..463] [gbkey=CDS]

tart, q. end, s. start, s. end, evalue, bit score

2625813	2.77E-27	108
3040538	3.93E-27	108
3055215	4.37E-27	108
3046568	9.31E-27	107
3056578	1.13E-26	106
1645722	2.70E-26	105
21499688	9.22E-27	107
24068987	2.45E-10	60.1
24068372	3.13E-05	45.4
20740168	2.58E-26	105
22463721	1.68E-16	77.8
37514289	2.60E-26	105
17014615	4.7	30.4
12808029	4.67E-25	102
12792470	3.54E-21	91.3
15225158	4.13E-16	76.6
15204126	2.73E-11	62.8
15204379	0.007	38.5
20134959	2	31.2
15647797	1.40E-10	60.8
15647544	2.93E-04	38.1
15647372	2.93E-04	24.3
16364901	7	29.6

riant protein] [protein_id=ACZ04984.1] [location=1..546] [gbkey=CDS]

tart, q. end, s. start, s. end, evalue, bit score

1645713	1.27E-27	111
3040544	7.62E-27	109
3056572	8.31E-27	108
3055221	9.60E-27	108
3046574	1.09E-26	108
37514295	6.34E-27	109
21499694	8.31E-27	108
24069050	1.47E-10	51.6
24068878	1.47E-10	33.9
24068357	0.33	34.7
2625819	8.47E-27	108
1069651	0.57	33.9
20740162	1.36E-26	108
22463730	8.12E-18	83.2
12808020	1.23E-25	105
12792464	4.52E-23	98.2

15647806	6.26E-17	68.2
15647544	6.26E-17	29.6
15647378	6.26E-17	28.5
15225149	3.37E-16	78.6
15204117	1.34E-14	68.2
15204379	1.34E-14	30.8
24733536	8.4	30.4

riant protein] [protein_id=ACZ04982.1] [location=1..555] [gbkey=CDS]

tart, q. end, s. start, s. end, evalue, bit score

1645713	7.86E-28	112
3055221	2.41E-27	110
3040544	2.73E-27	110
3056572	4.34E-27	110
3046574	6.56E-27	109
37514295	1.82E-27	111
2625819	1.91E-27	110
21499694	4.82E-27	109
24069050	4.23E-11	51.6
24068878	4.23E-11	35.8
24068357	0.3	34.7
20740162	6.20E-27	109
22463730	1.84E-18	85.1
12808020	1.38E-26	108
12792464	8.56E-24	100
15647806	3.49E-17	68.6
15647544	3.49E-17	30
15647372	3.49E-17	28.9
15225149	1.54E-16	79.7
15204117	9.29E-15	68.6
15204379	9.29E-15	31.2
24733536	6.9	30.8

riant protein] [protein_id=ACZ04980.1] [location=1..549] [gbkey=CDS]

tart, q. end, s. start, s. end, evalue, bit score

1645713	1.66E-20	90.9
3040544	3.20E-20	90.1
3055221	3.39E-20	90.1
3046574	3.46E-20	90.1
3056572	3.73E-20	90.1
2625819	2.72E-20	90.5
37514295	3.56E-20	90.1
21499694	3.92E-20	90.1
24069050	2.66E-07	52.8

24068357	5.2	30.8
20740162	6.21E-20	89.4
22463730	5.05E-12	66.6
12808020	3.65E-19	87
12792464	1.02E-17	82.8
15225149	6.15E-09	57.4
15204117	2.29E-07	52.8
15647806	1.91E-07	53.1
15647378	0.031	28.5
15647538	0.031	27.7

riant protein] [protein_id=ACZ04978.1] [location=1..489] [gbkey=CDS]

tart, q. end, s. start, s. end, evalue, bit score

1645713	2.17E-19	87
3056572	3.13E-19	86.7
3046574	3.65E-19	86.3
3040544	3.69E-19	86.3
3055221	3.76E-19	86.3
21499694	3.04E-19	86.7
24069050	2.25E-06	49.3
24068357	3.7	31.2
2625819	3.22E-19	86.7
37514295	4.14E-19	86.3
20740162	6.45E-19	85.5
22463730	5.19E-10	60.1
12808020	1.81E-18	84.3
12792464	6.87E-17	79.7
15225149	3.56E-09	57.8
15204117	4.93E-08	54.3
15647806	4.19E-08	54.3
15647538	0.58	27.7
15647378	0.58	23.1

ne H3] [protein_id=QBB85873.1] [location=join(1..52,159..205,292..343,424..461,556..595,665..78

tart, q. end, s. start, s. end, evalue, bit score

1645713	1.32E-27	111
3040544	8.68E-27	108
3055221	1.01E-26	108
3046574	1.08E-26	108
3056572	1.13E-26	108
37514295	6.76E-27	109
2625819	7.37E-27	109
1069651	0.75	33.5
21499694	1.00E-26	108

24069050	5.13E-11	51.6
24068878	5.13E-11	35.4
24068357	0.3	34.7
20740162	1.53E-26	108
22463730	3.54E-18	84.3
12808020	1.37E-25	105
12792464	4.40E-23	98.6
15647806	6.06E-17	68.2
15647544	6.06E-17	30
15647378	6.06E-17	28.5
15225149	3.19E-16	78.6
15204117	1.30E-14	68.2
15204379	1.30E-14	31.2
24733536	6.6	30.8

ne H3-3 variant] [protein_id=QBB85871.1] [location=1..537] [gbkey=CDS]

tart, q. end, s. start, s. end, evalue, bit score

1645713	4.14E-28	112
3040544	2.88E-27	110
3056572	3.43E-27	110
3055221	4.00E-27	109
3046574	5.67E-27	109
37514295	2.24E-27	110
2625819	3.06E-27	110
1069651	0.86	33.1
21499694	3.50E-27	110
24069050	5.24E-11	51.6
24068878	5.24E-11	35.4
24068357	0.34	34.7
20740162	4.33E-27	109
22463730	2.33E-18	84.7
12808020	6.21E-26	106
12792464	2.25E-23	99
15647806	2.82E-17	68.6
15647544	2.82E-17	30
15647372	2.82E-17	29.3
15225149	2.97E-16	78.6
15204117	1.29E-14	68.2
15204379	1.29E-14	30.8
24733536	6.5	30.8

iant 2] [protein_id=AOR06535.1] [location=1..525] [gbkey=CDS]

tart, q. end, s. start, s. end, evalue, bit score

3040541	7.58E-31	120

3055218	1.92E-30	119
3056575	2.39E-30	119
3046571	3.56E-30	118
1645710	1.56E-29	116
2625816	1.42E-30	119
21499691	2.05E-30	119
24069050	6.05E-13	65.9
24068914	6.05E-13	27.7
24068372	5.1	30.8
37514295	3.23E-30	118
20740165	1.03E-29	117
22463733	7.01E-20	89
12808017	9.21E-26	105
12792461	1.41E-23	99.4
15225143	1.03E-14	73.9
15204111	2.32E-09	58.5
15204379	0.002	41.6
20135004	0.003	33.1
20135153	0.003	26.6
15647809	3.06E-09	58.2
15647562	1.00E-06	43.1
15647372	1.00E-06	28.9

iant 1] [protein_id=AOR06534.1] [location=1..549] [gbkey=CDS]

tart, q. end, s. start, s. end, evalue, bit score

3040541	1.37E-30	120
3055218	2.61E-30	119
3056575	3.02E-30	119
3046571	4.45E-30	118
1645710	1.70E-29	116
2625816	1.81E-30	119
21499691	2.82E-30	119
24069050	7.25E-13	65.9
24068914	7.25E-13	27.7
24068372	5.6	30.8
37514295	4.20E-30	118
20740165	1.25E-29	117
22463733	4.66E-20	89.7
12808017	2.74E-25	104
12792461	2.25E-23	99.4
15225143	4.41E-15	75.5
15204111	2.72E-09	58.5
15204379	0.002	41.6
20135004	0.003	33.1
20135153	0.003	26.6
15647809	4.09E-09	58.2

15647562	1.08E-06	43.1
15647372	1.08E-06	28.9

ne H3-4 variant] [protein_id=AOH73624.1] [location=124..705] [gbkey=CDS]

tart, q. end, s. start, s. end, evalue, bit score

1645713	5.02E-28	113
3040544	2.82E-27	110
3056572	4.23E-27	110
3055221	4.27E-27	110
3046574	4.31E-27	110
37514295	2.64E-27	110
21499694	2.99E-27	110
24069050	1.21E-10	52
24068878	1.21E-10	33.9
24068357	0.37	34.7
2625819	3.13E-27	110
20740162	4.70E-27	110
22463730	1.90E-18	85.5
12808020	6.71E-26	107
12792464	2.60E-23	99.4
15647806	2.13E-17	69.3
15647544	2.13E-17	30
15647372	2.13E-17	29.3
15225149	2.14E-16	79.7
15204117	8.31E-15	68.9
15204379	8.31E-15	31.2
24733536	6.5	30.8

ne H3-2] [protein_id=AOH73622.1] [location=72..611] [gbkey=CDS]

tart, q. end, s. start, s. end, evalue, bit score

1645713	5.25E-28	112
3040544	2.76E-27	110
3056572	3.99E-27	109
3055221	4.35E-27	109
3046574	5.92E-27	109
37514295	2.21E-27	110
2625819	3.55E-27	110
21499694	4.43E-27	109
24069050	1.22E-10	51.6
24068878	1.22E-10	33.9
24068357	0.19	35.4
20740162	4.98E-27	109
22463730	1.82E-18	85.1
12808020	6.87E-26	106

12792464	1.92E-23	99.4
15647806	3.29E-17	68.9
15647544	3.29E-17	29.6
15647378	3.29E-17	28.9
15225149	1.82E-16	79.3
15204117	9.19E-15	68.9
15204379	9.19E-15	30.8

ıe H3-2] [protein_id=AOH73621.1] [location=107..655] [gbkey=CDS]

tart, q. end, s. start, s. end, evalue, bit score

1645713	5.39E-21	92.4
3040544	6.60E-21	92
3046574	6.73E-21	92
3055221	7.06E-21	92
3056572	7.27E-21	92
21499694	5.50E-21	92.4
24069050	6.79E-07	51.6
24068357	2.1	32.3
2625819	6.11E-21	92.4
37514295	1.02E-20	91.7
20740162	1.25E-20	91.3
22463730	3.32E-12	67
12808020	7.39E-20	89.4
12792464	2.00E-18	85.1
15225149	1.71E-08	56.2
15204117	1.06E-07	53.9
15647806	8.90E-08	54.3
15647378	0.023	28.9
15647538	0.023	27.7

ıe H3] [protein_id=AOH73620.1] [location=167..652] [gbkey=CDS]

tart, q. end, s. start, s. end, evalue, bit score

2625819	3.30E-19	86.3
1645713	3.30E-19	86.3
3056572	3.53E-19	86.3
3040544	3.71E-19	86.3
3055221	4.00E-19	86.3
3046574	5.05E-19	85.9
37514295	4.37E-19	86.3
21499694	4.72E-19	85.9
24069050	1.88E-06	49.7
24068357	5.6	30.4
20740162	7.01E-19	85.5
22463730	4.67E-10	60.1

12808020	2.21E-18	84
12792464	7.92E-17	79.7
15225149	1.02E-08	56.2
15204117	1.02E-07	53.1
15647806	6.57E-08	53.9
15647538	0.5	27.7
15647378	0.5	23.1

ne H3] [protein_id=AOH73617.1] [location=1..555] [gbkey=CDS]

tart, q. end, s. start, s. end, evalue, bit score

3040544	1.53E-19	88.2
1645713	2.06E-19	87.8
3056572	2.22E-19	87.8
3055221	2.52E-19	87.8
3046574	2.91E-19	87.4
21499694	1.65E-19	88.2
24069050	4.26E-07	52.4
24068357	7.7	30.4
2625819	1.94E-19	88.2
20740162	2.31E-19	87.8
22463730	3.42E-09	58.5
37514295	2.42E-19	87.8
12808020	1.49E-18	85.5
12792464	1.90E-17	82.4
15225149	1.13E-10	62.8
15204117	1.77E-06	50.4
15204379	0.2	35.4
15647806	1.63E-06	50.4
15647544	0.011	31.6
15647378	0.011	26.2

iant 2] [protein_id=ALF04640.1] [location=29..565] [gbkey=CDS]

tart, q. end, s. start, s. end, evalue, bit score

2625816	3.96E-31	121
3040541	3.96E-31	121
3055218	5.55E-31	120
3056575	1.25E-30	119
3046571	1.68E-30	119
1645710	1.99E-30	119
21499691	5.60E-31	120
24069050	6.21E-12	66.2
24068372	0.034	37.4
20740165	4.02E-30	118
22463733	2.30E-20	90.5

37514295	7.90E-30	117
19908895	2.2	32
12808005	2.52E-25	104
12792461	2.81E-23	99
15647809	5.37E-18	60.5
15647544	5.37E-18	38.5
15647372	5.37E-18	31.2
15225143	8.11E-17	80.1
15204111	2.28E-15	61.6
15204379	2.28E-15	40.4
20135004	8.65E-05	36.2
20135153	8.65E-05	29.3
28996498	0.76	33.5
15225722	1.4	32.7

iant 1] [protein_id=ALF04639.1] [location=29..565] [gbkey=CDS]

tart, q. end, s. start, s. end, evalue, bit score

3040541	4.07E-31	121
3055218	1.04E-30	120
3056575	1.40E-30	119
3046571	1.91E-30	119
1645710	2.58E-30	119
2625816	6.86E-31	120
21499691	7.85E-31	120
24069050	1.16E-12	68.2
24068372	0.051	37
20740165	3.79E-30	118
22463733	3.58E-20	89.7
37514295	6.77E-30	117
19908895	1.8	32.3
12808005	5.29E-25	103
12792461	3.44E-23	98.6
15647809	6.05E-18	60.1
15647544	6.05E-18	38.5
15647372	6.05E-18	31.6
15225143	1.38E-16	79.7
15204111	2.67E-15	61.2
15204379	2.67E-15	40.4
20135004	4.92E-05	35.8
20135153	4.92E-05	30.4
28996498	1.8	32.3
15225722	2.5	32

e H3 variant] [protein_id=AHH01567.1] [location=61..540] [gbkey=CDS]

tart, q. end, s. start, s. end, evalue, bit score

3040547	1.84E-25	104
3056569	2.19E-25	103
3055224	3.32E-25	103
3046577	3.99E-25	103
1645716	1.49E-24	101
2625822	2.01E-25	104
21499697	2.07E-25	103
24069050	3.41E-08	54.7
24068366	0.45	33.9
20740159	2.99E-25	103
22463727	1.09E-16	79
37514295	3.63E-25	103
12808023	2.47E-22	95.5
12792467	2.33E-19	86.7
15225152	1.04E-13	70.5
15204120	4.62E-09	57
15204367	0.037	37
15647803	2.09E-09	58.2
15647562	6.41E-04	37.7
15647372	6.41E-04	24.3

ie H3 variant] [protein_id=AGQ21573.1] [location=66..557] [gbkey=CDS]

start, q. end, s. start, s. end, evalue, bit score

3040547	2.91E-21	92.4
3056569	3.57E-21	92
3055224	5.20E-21	91.7
3046577	9.18E-21	90.9
1645716	1.74E-20	90.1
2625822	3.53E-21	92
21499697	4.28E-21	92
24069050	6.14E-08	50.1
24068911	6.14E-08	25.8
37514295	4.54E-21	92
20740159	6.74E-21	91.3
22463727	3.19E-11	63.5
12808023	5.01E-18	83.2
12792467	4.64E-17	80.5
15225152	3.26E-09	57.8
15204120	9.43E-07	50.4
15204373	0.029	37.4
15647803	9.16E-07	50.4
15647562	0.005	39.7
29457910	6.3	30.4

otein_id=AAR85315.1] [location=68..562] [gbkey=CDS]

tart, q. end, s. start, s. end, evalue, bit score

3040541	6.54E-31	120
3055218	1.30E-30	119
3056575	2.09E-30	118
3046571	3.11E-30	118
1645710	5.44E-29	114
2625816	7.94E-31	119
21499691	1.34E-30	119
24069050	3.80E-22	87
24068911	3.80E-22	37.4
24070031	2.26E-11	63.9
24068372	8.14E-07	50.8
24067964	0.014	38.5
20740165	5.71E-30	117
22463733	9.87E-18	82.4
37514295	7.86E-30	117
12808017	3.20E-25	103
12792461	2.21E-24	101
15225146	3.72E-14	72
15204114	2.54E-09	58.2
15204379	0.003	40
20134953	0.096	35.8
15225722	2.2	32
15647812	2.82E-09	57.8
15647562	9.93E-07	40.4
15647372	9.93E-07	31.6
11774869	7.4	30

tein_id=AAM74226.1] [location=66..539] [gbkey=CDS]

tart, q. end, s. start, s. end, evalue, bit score

3040541	1.04E-26	107
3055218	1.67E-26	107
3046571	3.51E-26	106
3056575	4.47E-26	105
1645710	1.40E-25	104
2625816	1.15E-26	107
21499691	2.98E-26	106
24069050	5.00E-10	59.7
24068372	2.28E-04	43.5
24070052	0.001	41.6
20740165	1.15E-25	104
22463733	4.31E-15	74.3
37514295	2.55E-25	103
12808017	1.27E-22	95.9

12792461	1.86E-21	92.8
15225146	2.58E-12	66.6
15204111	2.44E-09	57.8
15204391	0.077	35.8
20134953	3.5	31.2
15647809	1.95E-09	58.2
15647532	8.04E-04	35.8
15647372	8.04E-04	25.8

3 isoform B] [protein_id=ADM18965.1] [location=1..534] [gbkey=CDS]

start, q. end, s. start, s. end, evalue, bit score

3040553	1.69E-26	108
3056563	3.05E-26	107
3055230	5.83E-26	106
3046583	1.27E-25	105
1645722	1.03E-24	102
37514289	1.80E-26	107
2625828	3.92E-26	107
21499703	4.67E-26	106
24069050	4.32E-12	63.9
24068914	4.32E-12	26.6
24068372	1.7	32.3
20740153	1.23E-25	105
22463721	1.17E-15	77
12808029	1.68E-22	96.7
12792467	5.30E-21	92.4
15225158	1.62E-11	64.7
15204126	8.53E-08	53.9
15204358	0.002	41.2
20135004	2.1	32
15647797	6.66E-08	54.3
15647532	0.092	36.2
15647444	9.8	30

[protein_id=AYA72175.1] [location=11..475] [gbkey=CDS]

start, q. end, s. start, s. end, evalue, bit score

2625816	1.08E-36	136
3040541	1.19E-36	135
3055218	1.71E-36	135
3056575	1.97E-36	135
3046571	2.09E-35	132
1645710	4.68E-35	131
21499691	3.39E-36	134
24069050	9.78E-19	67.4

24068878	9.78E-19	45.1
24068372	0.003	40
20740165	5.14E-36	134
22463733	3.25E-25	103
37514295	8.19E-36	133
12808023	3.26E-32	123
12792461	2.66E-30	117
15225146	3.98E-23	97.4
15204114	2.49E-19	73.2
15204391	2.49E-19	41.6
20134953	1.47E-04	36.2
20135162	1.47E-04	27.7
15647809	1.42E-22	72.8
15647562	1.42E-22	41.2
15647372	1.42E-22	31.2

[protein_id=AYA72192.1] [location=15..479] [gbkey=CDS]

tart, q. end, s. start, s. end, evalue, bit score

3040541	2.59E-35	132
3056575	3.57E-35	131
3055218	5.20E-35	131
3046571	3.79E-34	129
1645710	3.90E-34	129
2625816	2.77E-35	132
21499691	7.67E-35	130
24069050	3.49E-16	60.8
24068878	3.49E-16	43.1
24068372	0.004	39.7
20740165	9.58E-35	130
22463733	3.06E-24	100
37514295	1.27E-34	130
12808017	8.91E-32	122
12792461	9.03E-29	113
15225146	5.91E-23	97.1
15204114	6.58E-19	73.6
15204391	6.58E-19	39.7
20134953	0.008	30.8
20135162	0.008	26.9
15225722	7.1	30
15647809	1.23E-21	73.6
15647532	1.23E-21	39.3
15647372	1.23E-21	29.6

[protein_id=AYA72176.1] [location=30..494] [gbkey=CDS]

tart, q. end, s. start, s. end, evalue, bit score

2625816	4.25E-39	143
3040541	6.32E-39	142
3055218	6.51E-39	142
3056575	1.93E-38	141
3046571	6.28E-38	139
1645710	6.94E-36	134
3154068	9.5	29.6
21499691	1.78E-38	141
24069050	1.52E-17	66.6
24068878	1.52E-17	42.4
24068372	0.003	40
20740165	9.07E-38	139
22463733	1.05E-26	107
37514295	2.11E-37	138
12808017	6.34E-33	125
12792461	6.00E-31	119
15225146	1.24E-21	93.2
15204114	1.78E-14	72.4
15204394	0.007	38.9
20135112	0.23	34.7
15225722	0.56	33.5
20135162	3	31.2
15647809	1.62E-14	72.8
15647529	5.34E-07	38.9
15647372	5.34E-07	33.5

H3] [protein_id=AUN88474.1] [location=1..456] [gbkey=CDS]

tart, q. end, s. start, s. end, evalue, bit score

2625816	8.86E-32	122
3040541	9.95E-32	121
3055218	1.52E-31	121
3046571	2.20E-31	120
3056575	2.89E-31	120
1645710	9.60E-31	119
21499691	2.10E-31	120
24069050	1.08E-10	59.7
24068878	1.08E-10	25.8
24068372	4.28E-07	51.2
24067964	0.68	33.1
20740165	7.83E-31	119
22463733	6.26E-21	90.9
19539781	1.1	32.3
37514295	1.36E-30	118
12808017	3.48E-29	114
12792461	1.81E-27	109

15225143	2.80E-20	89
15204111	3.69E-17	69.7
15204391	3.69E-17	37.4
20134953	0.01	30
20135153	0.01	27.3
15647809	4.31E-20	71.2
15647553	4.31E-20	40.8
15647372	4.31E-20	24.6

tone H3] [protein_id=AUN88469.1] [location=1..456] [gbkey=CDS]

tart, q. end, s. start, s. end, evalue, bit score

3040541	6.96E-32	122
3055218	1.05E-31	121
3056575	1.60E-31	121
3046571	2.00E-31	120
1645710	1.02E-30	119
2625816	7.16E-32	122
21499691	1.36E-31	121
24069050	4.71E-11	59.7
24068878	4.71E-11	26.9
24068372	4.16E-07	51.2
24067964	2.5	31.2
20740165	4.92E-31	119
22463733	8.22E-22	93.6
19539781	6.6	30
37514295	5.42E-31	119
12808017	5.55E-29	114
12792461	3.24E-27	108
15225143	2.40E-20	89.4
15204111	1.12E-16	68.2
15204391	1.12E-16	37.4
20134953	0.01	30
20135153	0.01	27.3
15647809	4.59E-20	71.2
15647553	4.59E-20	40.8
15647372	4.59E-20	24.6

istone H3] [protein_id=AUN88463.1] [location=1..462] [gbkey=CDS]

tart, q. end, s. start, s. end, evalue, bit score

2625816	7.96E-31	119
3040541	8.85E-31	119
3055218	1.28E-30	118
3056575	1.60E-30	118
3046571	2.10E-30	118

1645710	3.86E-30	117
21499691	1.22E-30	119
24069050	4.25E-10	56.2
24068878	4.25E-10	26.9
24068372	3.41E-08	54.3
24067964	0.91	32.7
20740165	4.55E-30	117
22463733	1.22E-20	90.1
37514295	8.29E-30	116
12808017	1.18E-27	110
12792461	5.28E-26	105
15225143	1.34E-19	87
15204111	2.48E-17	73.6
15204391	2.48E-17	34.3
20134953	0.008	30
20135153	0.008	27.7
15647809	2.56E-19	71.6
15647553	2.56E-19	37.4
15647372	2.56E-19	24.6

d=ALK04343.1] [location=103..633] [gbkey=CDS]

tart, q. end, s. start, s. end, evalue, bit score

2625819	1.88E-22	96.3
3040544	2.49E-22	95.9
3055221	2.51E-22	95.9
3046574	2.90E-22	95.9
3056572	4.79E-22	95.1
1645713	5.70E-22	95.1
21499694	4.52E-22	95.1
24069050	9.45E-09	57
24068357	2.3	32
37514295	6.28E-22	94.7
20740162	1.10E-21	94.4
22463730	4.84E-14	72
12808020	1.96E-21	93.6
12792464	1.76E-19	87.8
15225149	4.40E-10	60.8
15204117	1.06E-07	53.9
20134950	0.65	33.5
15204382	2.8	31.6
15225722	8.4	30.4
15647806	9.97E-08	53.9
15647444	2.6	32

d=ALK04341.1] [location=59..583] [gbkey=CDS]

tart, q. end, s. start, s. end, evalue, bit score

37514295	1.99E-27	110
1645713	5.60E-27	109
3056572	5.87E-27	109
3040544	8.16E-27	108
3055221	1.12E-26	108
3046574	1.68E-26	107
12808020	6.99E-27	108
12792464	4.59E-24	100
20740162	7.34E-27	108
22463730	1.53E-18	85.1
21499694	8.64E-27	108
24069050	4.77E-10	55.5
24068878	4.77E-10	28.1
24068357	0.027	37.7
2625819	8.73E-27	108
15647806	8.11E-17	71.2
15647532	8.11E-17	28.1
15647378	8.11E-17	26.6
15204117	4.10E-15	71.2
15204391	4.10E-15	29.6
15225149	7.02E-15	74.3

tone H3] [protein_id=BAO51832.1] [location=1..459] [gbkey=CDS]

tart, q. end, s. start, s. end, evalue, bit score

2625819	3.58E-30	117
3055221	4.92E-30	117
3040544	5.16E-30	117
3056572	2.08E-29	115
3046574	9.92E-27	107
1645713	1.94E-25	103
21499694	1.07E-29	115
24069050	3.57E-07	51.6
24068357	0.22	34.7
20740162	1.26E-28	113
22463730	3.80E-16	77.4
37514295	1.43E-28	112
12808020	1.72E-21	92.4
12792464	1.20E-19	87.4
15225149	1.90E-11	63.9
15204117	2.21E-07	52
15204379	0.003	39.7
15204716	0.71	33.1
15647806	2.39E-07	52
15647562	0.023	37.4

15647456	0.61	33.1
4666601	0.66	33.1

R12] [protein_id=AAL86777.1] [location=1..531] [gbkey=CDS]

tart, q. end, s. start, s. end, evalue, bit score

2625819	1.21E-22	97.1
3040544	1.64E-22	96.7
3055221	1.90E-22	96.3
3046574	2.30E-22	96.3
3056572	2.64E-22	95.9
1645713	2.54E-21	93.2
37514295	2.69E-22	95.9
21499694	2.93E-22	95.9
24069050	1.98E-08	55.8
24068357	1.2	32.7
20740162	5.38E-22	95.1
22463730	3.39E-13	69.7
12808020	2.26E-21	93.2
12792464	1.48E-19	88.2
15225149	6.21E-10	60.1
15204117	2.90E-08	55.5
20134950	0.35	34.3
15647806	2.16E-08	55.8
15647372	0.028	29.3
15647538	0.028	26.9

R12] [protein_id=AAL86775.1] [location=1..537] [gbkey=CDS]

tart, q. end, s. start, s. end, evalue, bit score

3040544	1.99E-21	93.6
3055221	2.66E-21	93.2
3056572	2.79E-21	93.2
3046574	5.69E-21	92
1645713	6.90E-21	92
2625819	2.19E-21	93.6
37514295	2.24E-21	93.2
21499694	2.90E-21	93.2
24069050	6.03E-07	51.6
24068357	1.2	32.7
20740162	5.80E-21	92
22463730	3.87E-13	69.7
12808005	2.29E-18	84.7
12792464	5.57E-17	80.9
15225149	1.97E-09	58.9
15204117	3.20E-08	55.5

20134950	0.74	33.5
15647806	2.59E-08	55.5

R12] [protein_id=AAL86776.1] [location=join(1..52,166..206,306..351,447..481,552..585,675..787,1

tart, q. end, s. start, s. end, evalue, bit score

2625819	1.20E-22	97.1
3040544	1.66E-22	96.7
3055221	1.74E-22	96.7
3046574	2.24E-22	96.3
3056572	2.35E-22	96.3
1645713	2.56E-21	93.2
37514295	2.54E-22	95.9
21499694	2.77E-22	95.9
24069050	1.82E-08	55.8
24068357	1.2	32.7
20740162	5.33E-22	95.1
22463730	5.16E-13	69.3
12808020	2.11E-21	93.2
12792464	1.42E-19	88.2
15225149	8.58E-10	59.7
15204117	2.85E-08	55.5
20134950	0.34	34.7
15647806	2.06E-08	55.8
15647372	0.027	29.3
15647538	0.027	26.9

37682 GN=alphaCENH3 PE=2 SV=1

tart, q. end, s. start, s. end, evalue, bit score

2625816	1.34E-36	135
18024302	0.62	32.7
13565292	5.8	30
24024545	7.1	29.6
3040541	2.12E-36	134
3055218	2.16E-36	134
3056575	2.51E-35	131
3046571	7.16E-32	121
1645710	5.22E-31	119
27987538	3.5	30.8
16492671	9.7	29.3
21499691	6.22E-36	133
24069050	1.47E-16	66.2
24068878	1.47E-16	38.5
24068372	3.01E-06	48.5
24070019	0.007	38.5

24067964	0.19	34.3
8282384	1.3	32
8334114	6.4	30
7739703	7.2	29.6
7092007	7.4	29.6
11985457	7.8	29.6
20740165	9.19E-35	130
22463733	2.82E-21	91.7
6034982	2	31.6
15614236	5.9	30
15776889	5.9	30
37514295	6.77E-34	127
10092977	1	32.3
12808005	8.15E-31	118
12792461	2.49E-29	114
26725481	6.4	30
15647809	2.69E-18	70.1
15647562	2.69E-18	32
15647366	2.69E-18	28.5
19623374	4	30.4
19302504	7.2	29.6
15225143	4.17E-17	79.7
15204111	1.69E-15	67
15204379	1.69E-15	34.3
20135004	1.96E-05	36.6
20135153	1.96E-05	30
15225722	9.7	29.3
4633661	3.3	30.8
10561038	3.8	30.4
12292811	5	30.4
13449364	6.6	30
7580759	9	29.6
13418342	9.6	29.3
9325304	10	29.3

vena sativa OX=4498 GN=AsCENH3-2 PE=2 SV=1

tart, q. end, s. start, s. end, evalue, bit score

2625828	1.52E-32	124
3040553	1.84E-32	124
3055230	2.03E-32	124
3046583	4.52E-32	123
3056563	5.93E-32	122
1645722	3.69E-31	120
21499703	4.70E-32	123
24069050	1.13E-15	67.4
24068878	1.13E-15	35

24068372	7.03E-06	47.8
24070019	3.11E-04	43.1
37514289	1.13E-31	122
20740153	3.19E-31	120
22463721	3.73E-22	94.7
12808029	3.42E-31	120
12792473	2.09E-27	109
15225158	8.46E-19	85.1
15204126	4.96E-17	67.4
15204391	4.96E-17	39.3
20134953	0.17	35
15225722	7	30
15647797	8.84E-17	67.4
15647532	8.84E-17	38.9
15647456	5.6	30.4
1018019	1.1	32.7

Avena sativa OX=4498 GN=AsCENH3-3 PE=2 SV=1

start, q. end, s. start, s. end, evalue, bit score

2625828	1.21E-32	124
3040553	1.41E-32	124
3055230	1.66E-32	124
3046583	4.17E-32	123
3056563	4.77E-32	123
1645722	2.20E-31	121
21499703	4.64E-32	123
24069050	7.09E-17	71.6
24068878	7.09E-17	34.7
24068372	1.07E-05	47.4
24070061	0.99	32.7
37514289	9.04E-32	122
12808029	1.98E-31	121
12792473	1.78E-27	110
20740153	2.81E-31	120
22463721	5.10E-22	94.4
15225158	2.55E-19	86.7
15204126	6.45E-17	67.4
15204391	6.45E-17	38.9
20134953	0.037	37
15225722	4.2	30.8
15647797	1.16E-16	67
15647532	1.16E-16	38.5
15647456	3	31.2
1018019	0.82	33.1

X=4573 GN=betaCENH3 PE=2 SV=1

tart, q. end, s. start, s. end, evalue, bit score

2625816	2.08E-28	112
3040541	2.08E-28	112
3055218	2.84E-28	112
3056575	3.22E-28	112
1645710	4.43E-28	111
3046571	4.60E-28	111
21499691	3.44E-28	111
24069053	3.44E-10	60.1
24068372	2.11E-08	55.1
24067964	0.8	32.7
20740165	1.02E-27	110
22463733	1.69E-18	84
37514295	1.59E-27	109
12808017	8.08E-26	105
12792461	2.46E-24	100
15647809	8.50E-18	68.9
15647553	8.50E-18	33.9
15647378	8.50E-18	26.2
15225143	6.45E-17	79.7
15204111	1.31E-15	70.9
15204403	1.31E-15	31.2
20134953	0.019	31.6
20135153	0.019	25

Avena sativa OX=4498 GN=AsCENH3-1 PE=2 SV=1

tart, q. end, s. start, s. end, evalue, bit score

2625828	2.06E-34	129
8742463	3.2	31.2
3040553	2.65E-34	129
3055230	3.10E-34	129
3046583	6.28E-34	128
3056563	7.19E-34	128
1645722	3.38E-33	126
21499703	5.43E-34	128
24069050	1.06E-15	63.9
24068878	1.06E-15	38.5
24068372	7.03E-06	47.8
24070019	3.27E-04	43.1
37514289	7.47E-34	128
20740153	1.37E-33	127
22463721	5.63E-24	100
12808029	1.30E-32	124
12792473	3.23E-29	115

15225158	1.71E-18	84.3
15204126	3.06E-18	69.7
15204391	3.06E-18	41.2
20134953	1.87E-05	35
20135141	1.87E-05	32
15225722	5.6	30.4
15647797	2.62E-18	69.7
15647544	2.62E-18	41.6
15647456	4.3	30.8

)S=Luzula pilosa OX=223700 PE=2 SV=1

tart, q. end, s. start, s. end, evalue, bit score

3040553	2.63E-27	109
3056563	5.69E-27	108
3055230	8.70E-27	108
3046583	9.14E-27	108
1645722	6.31E-26	105
2625828	4.43E-27	109
21499703	6.97E-27	108
24069050	1.13E-11	62.4
24068914	1.13E-11	26.6
24068372	0.7	33.5
37514289	7.46E-27	108
20740153	3.06E-26	107
22463721	6.97E-16	77
12808029	5.10E-23	97.8
12792470	4.02E-22	95.1
15225158	1.65E-12	67.4
15204126	8.05E-08	53.9
15204358	0.001	41.6
20135004	1.7	32.3
15225722	9.1	30
15647797	4.63E-08	54.7
15647532	0.073	36.2
15647444	7.3	30.4

)S=Luzula pilosa OX=223700 PE=3 SV=1

tart, q. end, s. start, s. end, evalue, bit score

3056563	2.21E-30	118
1645722	2.39E-30	118
3040553	3.28E-30	118
3055230	1.18E-29	116
3046583	3.44E-29	115
37514289	2.25E-30	118

12808029	3.98E-30	117
12792470	1.09E-26	108
20740153	4.34E-30	117
22463721	9.95E-20	88.2
21499703	5.48E-30	117
24069050	6.89E-15	63.5
24068860	6.89E-15	36.2
24068366	0.2	35
2625828	6.34E-30	117
15225158	2.42E-18	84
15204126	2.70E-16	68.6
15204358	2.70E-16	36.2
20135028	3.07E-04	35.8
20135141	3.07E-04	27.3
15647797	1.23E-15	69.7
15647532	1.23E-15	32.7
15647420	1.3	32.3
25276753	4.7	30.8

)S=Luzula sylvatica OX=59018 PE=2 SV=1

tart, q. end, s. start, s. end, evalue, bit score

37514289	2.45E-28	112
4816907	8.5	30
3056563	4.86E-28	111
1645722	5.96E-28	111
3040553	6.56E-28	111
3055230	1.25E-27	110
3046583	2.31E-27	109
20740153	5.36E-28	111
22463721	8.13E-19	85.5
2625828	1.01E-27	110
21499703	1.09E-27	110
24069050	2.49E-13	63.5
24068878	2.49E-13	31.2
24068366	0.042	37
12808029	2.44E-26	107
12792470	1.75E-23	98.6
15647797	4.27E-17	65.1
15647532	4.27E-17	33.5
15647372	4.27E-17	28.1
15225158	5.67E-17	80.1
15204126	7.53E-15	63.9
15204358	7.53E-15	35.8
20135067	0.19	35
7595156	9.6	29.6

)S=Luzula alpinopilosa OX=630085 PE=3 SV=1

;tart, q. end, s. start, s. end, evalue, bit score

37514289	5.08E-31	120
1645722	6.22E-31	120
3056563	6.40E-31	120
3040553	8.98E-31	119
3055230	2.21E-30	118
3046583	5.75E-30	117
21499703	1.27E-30	119
24069050	1.80E-15	63.5
24068860	1.80E-15	38.5
24068366	0.076	36.2
20740153	1.36E-30	119
22463721	4.37E-21	92
2625828	2.14E-30	118
12808029	9.51E-30	116
12792470	3.26E-26	106
15225158	8.31E-19	85.5
15204126	1.21E-16	69.3
15204358	1.21E-16	36.6
20134953	0.77	33.1
15647797	2.26E-16	70.5
15647532	2.26E-16	34.3
15647519	4.1	30.8

;=Luzula luzuloides OX=318444 PE=3 SV=1

;tart, q. end, s. start, s. end, evalue, bit score

3056563	1.93E-31	121
3040553	2.36E-31	121
1645722	4.14E-31	120
3055230	9.80E-31	119
3046583	5.53E-30	117
37514289	2.71E-31	121
21499703	7.12E-31	120
24069050	2.03E-15	65.5
24068860	2.03E-15	36.2
24068366	0.2	35
20740153	7.47E-31	120
22463721	1.91E-20	90.1
2625828	7.92E-31	119
12808029	9.61E-31	119
12792470	2.67E-27	109
15647797	2.52E-19	70.1
15647532	2.52E-19	34.3

15647372	2.52E-19	30
25276753	3	31.6
23469314	3.5	31.2
15225158	4.11E-19	86.3
15204126	5.88E-17	68.9
15204358	5.88E-17	38.1
20135028	1.01E-04	37.4
20135141	1.01E-04	27.3

)S=Luzula lutea OX=630107 PE=3 SV=1

tart, q. end, s. start, s. end, evalue, bit score

37514289	1.37E-30	119
1645722	2.89E-30	118
3056563	3.01E-30	118
3040553	3.10E-30	118
3055230	9.70E-30	116
3046583	2.81E-29	115
20740153	4.18E-30	117
22463721	7.64E-21	91.3
21499703	4.97E-30	117
24069050	4.48E-15	63.5
24068860	4.48E-15	37
24068366	0.14	35.4
2625828	8.97E-30	117
12808029	3.25E-29	115
12792470	7.71E-26	105
15225158	3.03E-18	84
15204126	4.71E-16	67.4
15204358	4.71E-16	36.6
20134953	0.003	33.5
20135141	0.003	25.8
7595156	9	30
15647797	1.03E-15	68.2
15647532	1.03E-15	34.3
25276753	3	31.6
15647444	5.6	30.4

=Luzula arcuata OX=223686 PE=3 SV=1

tart, q. end, s. start, s. end, evalue, bit score

37514289	8.97E-30	117
1645722	1.27E-29	116
3056563	1.30E-29	116
3040553	1.59E-29	116
3055230	4.30E-29	115

3046583	9.41E-29	114
20740153	2.29E-29	115
22463721	8.29E-20	88.2
21499703	2.63E-29	115
24069050	1.32E-16	67.4
24068860	1.32E-16	38.1
24068366	0.25	34.7
2625828	3.61E-29	115
12808029	3.50E-28	112
12792470	8.31E-25	102
15225158	1.43E-17	82
15204126	1.46E-15	65.9
15204358	1.46E-15	36.6
20134953	1.4	32.3
7595156	8.3	30
15647797	2.85E-15	66.6
15647532	2.85E-15	34.3
15647519	5.4	30.8

=Luzula wahlenbergii OX=223707 PE=3 SV=1

tart, q. end, s. start, s. end, evalue, bit score

37514289	2.95E-30	118
3056563	3.87E-30	117
3040553	4.22E-30	117
1645722	4.65E-30	117
3055230	9.98E-30	116
3046583	1.70E-29	116
20740153	7.69E-30	117
22463721	3.22E-20	89.4
2625828	8.07E-30	117
21499703	8.72E-30	117
24069050	4.77E-16	65.5
24068860	4.77E-16	38.1
24068366	0.27	34.7
12808029	6.09E-29	114
12792470	1.65E-25	104
15225158	5.72E-18	83.2
15204126	6.47E-16	67
15204358	6.47E-16	36.6
20134953	0.002	33.1
20135141	0.002	26.9
15647797	1.19E-15	68.2
15647532	1.19E-15	34.7
25276753	2.8	31.6
15647519	5.4	30.8
23469314	7.5	30

18761306	9.6	30

S=Luzula abyssinica OX=630083 PE=3 SV=1

tart, q. end, s. start, s. end, evalue, bit score

37514289	8.72E-30	117
1645722	1.04E-29	116
3040553	1.72E-29	115
3056563	1.89E-29	115
3055230	5.97E-29	114
3046583	2.94E-28	112
20740153	1.54E-29	116
22463721	7.53E-20	88.6
21499703	3.34E-29	115
24069050	7.05E-14	63.9
24068860	7.05E-14	32.7
24068366	0.018	38.1
11619445	8.7	30
2625828	4.38E-29	114
12808029	5.31E-28	111
12792470	6.40E-25	102
15647797	2.69E-18	67.4
15647532	2.69E-18	33.9
15647372	2.69E-18	30
15225158	1.04E-17	82.4
15204126	1.71E-15	66.2
15204358	1.71E-15	35.8
20135079	0.049	36.6
7595156	7	30.4

OS=Luzula abyssinica OX=630083 PE=2 SV=1

tart, q. end, s. start, s. end, evalue, bit score

37514289	5.75E-34	128
3040553	1.30E-33	127
3056563	1.31E-33	127
3055230	3.65E-33	126
3046583	8.32E-33	125
1645722	2.46E-32	124
2625828	2.30E-33	127
21499703	2.55E-33	126
24069050	1.59E-16	65.1
24068878	1.59E-16	40
24068372	0.01	38.5
20740153	8.00E-33	125
22463721	7.27E-22	94

12808029	7.13E-31	119
12792470	3.99E-28	112
15225158	5.46E-20	88.6
15204126	5.32E-19	72.8
15204379	5.32E-19	40.8
20135141	0.66	33.1
20135076	1.1	32.7
26040983	1.8	32
15647797	7.16E-18	72.8
15647562	7.16E-18	37
16794597	0.39	33.9

S=Luzula multiflora OX=178423 PE=2 SV=1

tart, q. end, s. start, s. end, evalue, bit score

37514289	8.80E-34	128
3040553	1.75E-33	127
3056563	1.78E-33	127
3055230	5.42E-33	126
3046583	1.22E-32	125
1645722	3.15E-32	124
2625828	3.54E-33	126
21499703	3.54E-33	126
24069050	1.45E-16	65.1
24068878	1.45E-16	40.4
24068372	0.011	38.5
20740153	9.88E-33	125
22463721	1.33E-21	93.6
12808029	2.75E-29	115
12792470	3.20E-27	109
15225158	7.63E-20	88.6
15204126	5.64E-19	72.8
15204379	5.64E-19	41.2
20135141	0.9	33.1
20135076	1.9	32
26040983	2.2	32
15647797	8.10E-18	72.8
15647562	8.10E-18	37
16794597	0.45	33.9

)S=Luzula luzuloides OX=318444 PE=2 SV=1

tart, q. end, s. start, s. end, evalue, bit score

37514289	7.84E-34	128
3056563	1.36E-33	127
3040553	1.60E-33	127

3055230	4.60E-33	126
3046583	1.21E-32	125
1645722	2.50E-32	124
21499703	2.84E-33	127
24069050	1.16E-16	65.5
24068878	1.16E-16	40.4
24068372	0.011	38.5
2625828	2.92E-33	127
20740153	8.46E-33	125
22463721	1.67E-21	93.2
12808029	2.31E-29	115
12792470	2.69E-27	109
15225158	8.40E-20	88.2
15204126	6.02E-19	72.4
15204379	6.02E-19	41.2
20135141	0.63	33.5
20135076	1.6	32.3
26040983	2.1	32
15647797	7.65E-18	72.8
15647562	7.65E-18	37
16794597	0.5	33.9

)S=Luzula canariensis OX=223688 PE=2 SV=1

;tart, q. end, s. start, s. end, evalue, bit score

37514289	4.21E-29	114
4816907	7.4	30
3056563	8.13E-29	114
1645722	8.87E-29	114
3040553	9.77E-29	114
3055230	1.81E-28	113
3046583	4.33E-28	112
20740153	8.37E-29	114
22463721	5.12E-19	85.9
2625828	1.54E-28	113
21499703	1.63E-28	113
24069050	1.02E-13	63.5
24068860	1.02E-13	32.3
24068366	0.023	37.7
11619445	7.4	30
2715423	7.9	30
12808029	1.04E-26	107
12792470	5.70E-24	100
15647797	1.25E-17	67
15647532	1.25E-17	33.5
15647372	1.25E-17	28.1
15225158	1.27E-17	82

15204126	2.12E-15	65.9
15204358	2.12E-15	35.4
20135079	0.21	34.7

)S=Luzula canariensis OX=223688 PE=2 SV=1

tart, q. end, s. start, s. end, evalue, bit score

37514289	9.41E-34	128
3040553	1.41E-33	128
3056563	1.66E-33	127
3055230	5.21E-33	126
3046583	1.29E-32	125
1645722	2.91E-32	124
2625828	3.47E-33	127
21499703	3.64E-33	127
24069050	1.34E-16	65.5
24068878	1.34E-16	40.4
24068372	0.012	38.5
20740153	1.05E-32	125
22463721	6.10E-21	91.7
12808029	4.94E-30	117
12792470	9.30E-28	111
15225158	8.57E-20	88.6
15204126	7.27E-19	72.8
15204358	7.27E-19	40.8
20135141	0.48	33.9
20135076	2.5	31.6
26040983	2.7	31.6
15647797	1.17E-17	72.8
15647562	1.17E-17	36.6
16794597	2.4	32

;=Luzula divulgata OX=223689 PE=2 SV=1

tart, q. end, s. start, s. end, evalue, bit score

37514289	6.21E-34	129
3056563	1.27E-33	127
3040553	1.44E-33	127
3055230	4.92E-33	126
3046583	1.07E-32	125
1645722	3.61E-32	124
21499703	2.55E-33	127
24069050	2.15E-16	65.5
24068878	2.15E-16	39.7
24068372	0.011	38.5
2625828	2.68E-33	127

20740153	7.61E-33	125
22463721	3.51E-21	92.4
12808029	1.10E-29	116
12792470	7.64E-28	111
15225158	3.23E-20	89.7
15204126	6.43E-19	72.8
15204358	6.43E-19	40.8
20135141	1.33E-04	32.7
20135004	1.33E-04	31.6
26040983	3.3	31.2
15647797	8.50E-18	72.8
15647562	8.50E-18	37
16794597	0.24	34.7

572 GN=betaCENH3 PE=2 SV=1

tart, q. end, s. start, s. end, evalue, bit score

3040541	2.81E-30	117
3055218	4.22E-30	117
3056575	7.85E-30	116
3046571	8.90E-30	116
1645710	1.52E-29	115
2625816	3.25E-30	117
21499691	6.34E-30	116
24069050	5.88E-10	55.5
24068878	5.88E-10	26.9
24068372	2.24E-08	55.1
24067964	0.056	36.2
20740165	1.50E-29	115
22463733	2.47E-20	89.4
37514295	3.38E-29	114
29575758	7.8	30
12808017	2.72E-27	108
12792461	9.39E-26	104
15647812	5.13E-19	71.2
15647553	5.13E-19	37.7
15647378	5.13E-19	24.3
15225146	1.13E-17	81.6
15204114	7.56E-17	71.6
15204391	7.56E-17	34.3
20134953	0.026	29.6
20135153	0.026	26.2
18303078	9.5	29.6

rinum subsp. marinum OX=112516 GN=CENH3 PE=3 SV=1

tart, q. end, s. start, s. end, evalue, bit score

3040541	2.20E-28	109
3056575	2.57E-28	109
3055218	4.21E-28	108
3046571	6.21E-28	108
1645710	6.71E-28	108
2625816	2.86E-28	108
21499691	3.54E-28	108
24068372	4.06E-07	48.9
24069050	6.70E-07	47
24068878	6.70E-07	24.3
24067964	0.78	31.2
20740165	9.53E-28	107
22463733	2.67E-21	89.4
37514295	2.10E-26	103
29575758	9.9	28.1
12792461	6.66E-26	102
12808017	1.39E-25	101
15225143	1.39E-21	90.1
15204111	1.52E-18	73.6
15204391	1.52E-18	37.7
20135153	7.1	28.5
15647809	5.69E-19	71.2
15647553	5.69E-19	41.2

:tart, q. end, s. start, s. end, evalue, bit score

1645737	8.42E-16	75.5
3040568	9.45E-16	75.5
3046598	1.34E-15	75.1
3055245	1.49E-15	74.7
3056548	3.60E-15	73.6
21499718	8.58E-16	75.5
24069050	9.15E-14	63.2
24068884	9.15E-14	32
21481460	2.9	30.8
2625843	1.09E-15	75.1
13565307	6.4	29.6
25104119	9.9	29.3
20740138	4.91E-15	73.2
22463706	3.76E-08	53.5
37514274	5.51E-15	73.2
12808044	2.62E-14	71.2
12792488	1.28E-12	66.2
15647782	3.37E-07	34.7
15647562	3.37E-07	28.9

15647366	3.37E-07	27.7
2879986	0.45	33.1
20135013	0.4	33.1

tart, q. end, s. start, s. end, evalue, bit score

16555325	4.25E-16	84
20740120	7.32E-13	73.9
22463688	8.91E-05	48.1
16555799	0.027	40.4
21808238	4.3	33.1
11686984	9.2	32
25699334	9.4	32
37514256	2.18E-13	75.5
40114182	3.7	33.5
24681253	5	33.1
2625861	3.30E-13	75.1
1480291	5	33.1
3046616	3.42E-13	75.1
1645755	4.54E-13	74.7
3040586	4.92E-13	74.7
3055263	5.00E-13	74.7
3056530	6.88E-13	73.9
21499736	7.86E-13	73.9
24069050	2.11E-09	48.5
24068878	2.11E-09	34.7
24068366	0.003	43.1
12808062	8.83E-10	64.3
12792506	3.01E-08	59.3
15647764	1.10E-05	41.2
15647532	1.10E-05	24.3
15647372	1.10E-05	23.5
30571	1.7	34.7
27307682	2.5	33.9
15204159	4.13E-04	39.7
15204391	4.13E-04	25.4
15225191	0.004	43.1
17476167	4	33.1
18492630	1.4	34.7
1103744	6.4	32.7

GN=CenH3 PE=4 SV=1

tart, q. end, s. start, s. end, evalue, bit score

3040787	2.70E-04	40.4

3055464	3.55E-04	40
3046817	3.69E-04	40
3056329	4.96E-04	39.7
1645956	0.001	38.5
2626062	2.92E-04	40
12808263	3.03E-04	40
12792707	0.032	34.3
21499937	3.62E-04	40
24069107	0.009	35.8
24070019	1.4	29.6
37514055	5.05E-04	39.7
20739919	6.15E-04	39.3
22463301	0.12	32.7
20134950	0.014	35.4
15225722	0.091	33.1
15647372	0.088	33.1

3680 GN=CenH3 PE=4 SV=1

104334 GN=CenH3 PE=4 SV=1

=1104331 GN=CenH3 PE=4 SV=1

60324 GN=CenH3.M2 PE=4 SV=1

tart, q. end, s. start, s. end, evalue, bit score

24070058	5	27.3

336 GN=CenH3 PE=4 SV=1

tart, q. end, s. start, s. end, evalue, bit score

2626062	0.24	30
3055464	0.26	29.6
3046817	0.29	29.6
1645956	1.2	28.1
3056329	2.3	27.3
3040787	4	26.6
37514055	0.49	28.9
24069107	0.66	28.5
21499937	0.71	28.5

20134950	1.7	27.7
12808263	2.5	26.9
20739919	2.9	26.9

tart, q. end, s. start, s. end, evalue, bit score

16368373	3.4	27.3
5339458	5.5	26.6
15198633	7.3	26.2
13368945	7.5	26.2
18557714	8.6	26.2
5362919	9.8	26.2

'78 GN=CenH3 PE=4 SV=1

324 GN=CenH3 PE=4 SV=1

tart, q. end, s. start, s. end, evalue, bit score

24069107	0.007	35
21499937	0.022	33.5
37514055	0.018	33.5
7848467	1.6	28.1
6071617	7.4	26.2
3040787	0.022	33.5
3056329	0.023	33.5
3046817	0.027	33.1
3055464	0.03	33.1
1645956	0.062	32
2626062	0.027	33.1
20739919	0.042	32.7
22463301	3.6	27.3
12808263	0.086	31.6
20134950	0.19	30.8
15225722	3.2	27.3
23027387	5.6	26.6
21058878	6.6	26.6
15647372	7.7	26.2
4467769	8.6	26.2

669780 GN=CenH3 PE=4 SV=1

tart, q. end, s. start, s. end, evalue, bit score

24068009	1.4	28.9
14301232	2.3	28.1
18264613	5.4	27.3

tart, q. end, s. start, s. end, evalue, bit score

21991015	0.17	32.3
2973054	1.9	29.3
26320871	3.2	28.9
8612549	7.7	27.7
29039306	4.4	28.5
5183134	7.2	27.7

tart, q. end, s. start, s. end, evalue, bit score

12792586	0.56	30.4
15559648	4.4	28.1
20740849	5.2	27.7
7780327	6.3	27.7
11837801	9.1	26.9
2815256	9.3	26.9

tart, q. end, s. start, s. end, evalue, bit score

3056443	2.20E-06	44.3
3040649	0.008	34.3
3046679	0.009	34.3
3055326	0.055	32
1645812	0.24	30.4
24068480	3.35E-05	41.2
21499796	0.73	28.9
15647668	0.001	37
12792587	0.002	36.2
12808158	0.011	33.9

22463625	0.005	35
20740060	0.12	31.2
15225254	0.13	30.8
15204219	0.34	29.6
37514196	0.56	29.3
32964448	8	25.8
2625921	0.83	28.9

tart, q. end, s. start, s. end, evalue, bit score

11680018	1.3	27.7
21105032	1.4	27.3
16517049	6.3	25.8
20508551	4.4	26.2
27774700	5	25.8
18467418	5.2	25.8
5525657	5.6	25.8

66..1621)] [gbkey=CDS]

;bkey=CDS]

[bkey=CDS]

[bkey=CDS]

[bkey=CDS]

[bkey=CDS]

[bkey=CDS]

;bkey=CDS]

›key=CDS]

key=CDS]

ɔkey=CDS]

ɔkey=CDS]

0,920..997,1099..1174,1255..1310)] [gbkey=CDS]

207..1284,1416..1491,1632..1687)] [gbkey=CDS]
