# Supplementary material for: Uncovering natural allelic and structural variants of OsCENH3 gene by targeted resequencing and in silico mining in genus Oryza
Source: Sci Rep. 2023 Jan 16;13:830. doi: 10.1038/s41598-023-28053-w (PMC9842635; doi:10.1038/s41598-023-28053-w)
Supplement: Supplementary file 4 — Supplementary Table S2. [file 41598_2023_28053_MOESM4_ESM.pdf]

Supplementary Table S2: Results of BLASTn of CENH3 CDS query sequences against rice ge
BLASTN 2.10.1+
Query: lcl|KP202363.1_cds_AJF96734.1_1
Database: Nippon
0 hits found
BLASTN 2.10.1+
Query: lcl|KP177475.1_cds_AJD07128.1_1
Database: Nippon
0 hits found
BLASTN 2.10.1+
Query: lcl|KP177470.1_cds_AJD07123.1_1
Database: Nippon
0 hits found
BLASTN 2.10.1+
Query: lcl|KP177464.1_cds_AJD07117.1_1
Database: Nippon
0 hits found
BLASTN 2.10.1+
Query: lcl|KP177463.1_cds_AJD07116.1_1
Database: Nippon
0 hits found
BLASTN 2.10.1+
Query: lcl|KP177462.1_cds_AJD07115.1_1
Database: Nippon
0 hits found
BLASTN 2.10.1+
Query: lcl|KP177459.1_cds_AJD07112.1_1
Database: Nippon
0 hits found
BLASTN 2.10.1+
Query: lcl|KP177458.1_cds_AJD07111.1_1
Database: Nippon
0 hits found
BLASTN 2.10.1+
Query: lcl|GQ849334.1_cds_ACX30895.1_1
Database: Nippon
Fields: query acc.ver, subject acc.ver, % identity, alignment length, mismatches, gap oper
5 hits found

lcl|GQ849334.1_cds_ACX30895.1_1	Chr5
lcl|GQ849334.1_cds_ACX30895.1_1	Chr5
lcl|GQ849334.1_cds_ACX30895.1_1	Chr5
lcl|GQ849334.1_cds_ACX30895.1_1	Chr5
lcl|GQ849334.1_cds_ACX30895.1_1	Chr5

BLASTN 2.10.1+
Query: lcl|GQ849333.1_cds_ACX30894.1_1
Database: Nippon
Fields: query acc.ver, subject acc.ver, % identity, alignment length, mismatches, gap oper
5 hits found

lcl|GQ849333.1_cds_ACX30894.1_1	Chr5

lcl|GQ849333.1_cds_ACX30894.1_1	Chr5
lcl|GQ849333.1_cds_ACX30894.1_1	Chr5
lcl|GQ849333.1_cds_ACX30894.1_1	Chr5
lcl|GQ849333.1_cds_ACX30894.1_1	Chr5
BLASTN 2.10.1+
Query: lcl|GQ849332.1_cds_ACX30893.1_1
Database: Nippon
Fields: query acc.ver, subject acc.ver, % identity, alignment length, mismatches, gap oper
3 hits found
lcl|GQ849332.1_cds_ACX30893.1_1	Chr5
lcl|GQ849332.1_cds_ACX30893.1_1	Chr5
lcl|GQ849332.1_cds_ACX30893.1_1	Chr5
BLASTN 2.10.1+
Query: lcl|GQ849328.1_cds_ACX30889.1_1
Database: Nippon
Fields: query acc.ver, subject acc.ver, % identity, alignment length, mismatches, gap oper
3 hits found
lcl|GQ849328.1_cds_ACX30889.1_1	Chr5
lcl|GQ849328.1_cds_ACX30889.1_1	Chr5
lcl|GQ849328.1_cds_ACX30889.1_1	Chr5
BLASTN 2.10.1+
Query: lcl|KJ651124.1_cds_AJO61187.1_1
Database: Nippon
0 hits found
BLASTN 2.10.1+
Query: lcl|KJ651120.1_cds_AJO61183.1_1
Database: Nippon
0 hits found
BLASTN 2.10.1+
Query: lcl|KJ651113.1_cds_AJO61176.1_1
Database: Nippon
0 hits found
BLASTN 2.10.1+
Query: lcl|KJ651193.1_cds_AJO61254.1_1
Database: Nippon
0 hits found
BLASTN 2.10.1+
Query: lcl|KJ651192.1_cds_AJO61253.1_1
Database: Nippon
0 hits found
BLASTN 2.10.1+
Query: lcl|KJ651190.1_cds_AJO61251.1_1
Database: Nippon
0 hits found
BLASTN 2.10.1+
Query: lcl|KJ651189.1_cds_AJO61250.1_1
Database: Nippon
0 hits found

BLASTN 2.10.1+
Query: lcl|KJ651187.1_cds_AJO61248.1_1
Database: Nippon
0 hits found
BLASTN 2.10.1+
Query: lcl|KJ651184.1_cds_AJO61245.1_1
Database: Nippon
0 hits found
BLASTN 2.10.1+
Query: lcl|KJ651181.1_cds_AJO61243.1_1
Database: Nippon
0 hits found
BLASTN 2.10.1+
Query: lcl|KJ651178.1_cds_AJO61240.1_1
Database: Nippon
0 hits found
BLASTN 2.10.1+
Query: lcl|KJ651175.1_cds_AJO61237.1_1
Database: Nippon
0 hits found
BLASTN 2.10.1+
Query: lcl|KJ651171.1_cds_AJO61233.1_1
Database: Nippon
0 hits found
BLASTN 2.10.1+
Query: lcl|KJ651169.1_cds_AJO61231.1_1
Database: Nippon
0 hits found
BLASTN 2.10.1+
Query: lcl|KJ651166.1_cds_AJO61228.1_1
Database: Nippon
0 hits found
BLASTN 2.10.1+
Query: lcl|KJ651163.1_cds_AJO61225.1_1
Database: Nippon
0 hits found
BLASTN 2.10.1+
Query: lcl|KJ651160.1_cds_AJO61222.1_1
Database: Nippon
0 hits found
BLASTN 2.10.1+
Query: lcl|KJ651159.1_cds_AJO61221.1_1
Database: Nippon
0 hits found
BLASTN 2.10.1+
Query: lcl|KJ651157.1_cds_AJO61219.1_1
Database: Nippon
0 hits found

BLASTN 2.10.1+
Query: lcl|KJ651154.1_cds_AJO61216.1_1
Database: Nippon
0 hits found
BLASTN 2.10.1+
Query: lcl|KJ651151.1_cds_AJO61213.1_1 [protein=CenH3] [protein_id=AJO61213.1] [loca
Database: Nippon
0 hits found
BLASTN 2.10.1+
Query: lcl|KJ651146.1_cds_AJO61208.1_1 [protein=CenH3] [protein_id=AJO61208.1] [loca
Database: Nippon
0 hits found
BLASTN 2.10.1+
Query: lcl|KJ651142.1_cds_AJO61205.1_1 [protein=CenH3] [protein_id=AJO61205.1] [loca
Database: Nippon
0 hits found
BLASTN 2.10.1+
Query: lcl|KJ651140.1_cds_AJO61203.1_1 [protein=CenH3] [protein_id=AJO61203.1] [loca
Database: Nippon
0 hits found
BLASTN 2.10.1+
Query: lcl|KJ651136.1_cds_AJO61199.1_1 [protein=CenH3] [protein_id=AJO61199.1] [loca
Database: Nippon
0 hits found
BLASTN 2.10.1+
Query: lcl|KJ651133.1_cds_AJO61196.1_1 [protein=CenH3] [protein_id=AJO61196.1] [loca
Database: Nippon
0 hits found
BLASTN 2.10.1+
Query: lcl|KJ651128.1_cds_AJO61191.1_1 [protein=CenH3] [protein_id=AJO61191.1] [loca
Database: Nippon
0 hits found
BLASTN 2.10.1+
Query: lcl|KJ651123.1_cds_AJO61186.1_1 [protein=CenH3] [protein_id=AJO61186.1] [loca
Database: Nippon
0 hits found
BLASTN 2.10.1+
Query: lcl|KJ651119.1_cds_AJO61182.1_1 [protein=CenH3] [protein_id=AJO61182.1] [loca
Database: Nippon
0 hits found
BLASTN 2.10.1+
Query: lcl|KJ651118.1_cds_AJO61181.1_1 [protein=CenH3] [protein_id=AJO61181.1] [loca
Database: Nippon
0 hits found
BLASTN 2.10.1+
Query: lcl|KJ651116.1_cds_AJO61179.1_1 [protein=CenH3] [protein_id=AJO61179.1] [loca
Database: Nippon
0 hits found

BLASTN 2.10.1+
Query: lcl|KJ651112.1_cds_AJO61175.1_1 [protein=CenH3] [protein_id=AJO61175.1] [loca
Database: Nippon
0 hits found
BLASTN 2.10.1+
Query: lcl|KJ651108.1_cds_AJO61171.1_1 [protein=CenH3] [protein_id=AJO61171.1] [loca
Database: Nippon
0 hits found
BLASTN 2.10.1+
Query: lcl|KJ651107.1_cds_AJO61170.1_1 [protein=CenH3] [protein_id=AJO61170.1] [loca
Database: Nippon
0 hits found
BLASTN 2.10.1+
Query: lcl|KJ507244.1_cds_AHW98238.1_1 [gene=CenH3] [protein=centromeric histone 3
Database: Nippon
0 hits found
BLASTN 2.10.1+
Query: lcl|KJ507243.1_cds_AHW98237.1_1 [gene=CenH3] [protein=centromeric histone 3
Database: Nippon
0 hits found
BLASTN 2.10.1+
Query: lcl|KJ507242.1_cds_AHW98236.1_1 [gene=CenH3] [protein=centromeric histone 3
Database: Nippon
0 hits found
BLASTN 2.10.1+
Query: lcl|KJ507240.1_cds_AHW98234.1_1 [gene=CenH3] [protein=centromeric histone 3
Database: Nippon
0 hits found
BLASTN 2.10.1+
Query: lcl|KJ507235.1_cds_AHW98229.1_1 [gene=CenH3] [protein=centromeric histone 3
Database: Nippon
0 hits found
BLASTN 2.10.1+
Query: lcl|KJ507234.1_cds_AHW98228.1_1 [gene=CenH3] [protein=centromeric histone 3
Database: Nippon
0 hits found
BLASTN 2.10.1+
Query: lcl|HM582916.1_cds_ADN92693.1_1 [gene=CENH3] [protein=centromere-specific
Database: Nippon
0 hits found
BLASTN 2.10.1+
Query: lcl|MN625524.1_cds_QGY64363.1_1 [gene=CENH3] [protein=histone H3-like centı
Database: Nippon
0 hits found
BLASTN 2.10.1+
Query: lcl|MN625523.1_cds_QGY64362.1_1 [gene=CENH3] [protein=histone H3-like centı
Database: Nippon
0 hits found

BLASTN 2.10.1+
Query: lcl|MN625521.1_cds_QGY64360.1_1 [gene=CENH3] [protein=histone H3-like centr
Database: Nippon
0 hits found
BLASTN 2.10.1+
Query: lcl|MN625520.1_cds_QGY64359.1_1 [gene=CENH3] [protein=histone H3-like centr
Database: Nippon
0 hits found
BLASTN 2.10.1+
Query: lcl|MN625519.1_cds_QGY64358.1_1 [gene=CENH3] [protein=histone H3-like centr
Database: Nippon
0 hits found
BLASTN 2.10.1+
Query: lcl|MG384774.1_cds_AUN88460.1_1 [gene=CENH3] [protein=alpha centromeric h
Database: Nippon
0 hits found
BLASTN 2.10.1+
Query: lcl|KT600804.1_cds_AMH40810.1_1 [gene=CENH3] [protein=centromere-specific h
Database: Nippon
0 hits found
BLASTN 2.10.1+
Query: lcl|KT600803.1_cds_AMH40809.1_1 [gene=CENH3] [protein=centromere-specific h
Database: Nippon
0 hits found
BLASTN 2.10.1+
Query: lcl|KP878239.1_cds_AKI32619.1_1 [gene=CENH3] [protein=centromere specific his
Database: Nippon
0 hits found
BLASTN 2.10.1+
Query: lcl|KP878237.1_cds_AKI32617.1_1 [gene=CENH3] [protein=centromere specific his
Database: Nippon
0 hits found
BLASTN 2.10.1+
Query: lcl|KP878236.1_cds_AKI32616.1_1 [gene=CENH3] [protein=centromere specific his
Database: Nippon
0 hits found
BLASTN 2.10.1+
Query: lcl|KP878228.1_cds_AKI32608.1_1 [gene=CENH3] [protein=centromere specific his
Database: Nippon
0 hits found
BLASTN 2.10.1+
Query: lcl|KP878222.1_cds_AKI32602.1_1 [gene=CENH3] [protein=centromere specific his
Database: Nippon
0 hits found
BLASTN 2.10.1+
Query: lcl|KP878221.1_cds_AKI32601.1_1 [gene=CENH3] [protein=centromere specific his
Database: Nippon
0 hits found

BLASTN 2.10.1+
Query: lcl|AB770164.1_cds_BAP26971.1_1 [gene=CENH3] [protein=centromeric histone H
Database: Nippon
0 hits found
BLASTN 2.10.1+
Query: lcl|AB770163.1_cds_BAP26970.1_1 [gene=CENH3] [protein=centromeric histone H
Database: Nippon
0 hits found
BLASTN 2.10.1+
Query: lcl|JF969287.1_cds_AEH95352.1_1 [gene=CENH3] [protein=centromeric histone 3]
Database: Nippon
0 hits found
BLASTN 2.10.1+
Query: lcl|JF969286.1_cds_AEH95351.1_1 [gene=CENH3] [protein=centromeric histone 3]
Database: Nippon
0 hits found
BLASTN 2.10.1+
Query: lcl|JF969285.1_cds_AEH95350.1_1 [gene=CENH3] [protein=centromeric histone 3]
Database: Nippon
0 hits found
BLASTN 2.10.1+
Query: lcl|JF419330.1_cds_AEK21394.1_1 [gene=CENH3] [protein=beta centromeric histo
Database: Nippon
0 hits found
BLASTN 2.10.1+
Query: lcl|JF419329.1_cds_AEK21393.1_1 [gene=CENH3] [protein=beta centromeric histo
Database: Nippon
0 hits found
BLASTN 2.10.1+
Query: lcl|GU166744.1_cds_ACZ04984.1_1 [gene=CENH3] [protein=centromere-specific H
Database: Nippon
0 hits found
BLASTN 2.10.1+
Query: lcl|GU166742.1_cds_ACZ04982.1_1 [gene=CENH3] [protein=centromere-specific H
Database: Nippon
0 hits found
BLASTN 2.10.1+
Query: lcl|GU166740.1_cds_ACZ04980.1_1 [gene=CENH3] [protein=centromere-specific H
Database: Nippon
0 hits found
BLASTN 2.10.1+
Query: lcl|GU166738.1_cds_ACZ04978.1_1 [gene=CENH3] [protein=centromere-specific H
Database: Nippon
0 hits found
BLASTN 2.10.1+
Query: lcl|MH329772.1_cds_QBB85873.1_1 [gene=CENH3] [protein=centromere specific
Database: Nippon
0 hits found

BLASTN 2.10.1+
Query: lcl|MH329770.1_cds_QBB85871.1_1 [gene=CENH3] [protein=centromere specific
Database: Nippon
0 hits found
BLASTN 2.10.1+
Query: lcl|KT932954.1_cds_AOR06535.1_1 [gene=CENH3] [protein=centromere histone H
Database: Nippon
0 hits found
BLASTN 2.10.1+
Query: lcl|KT932953.1_cds_AOR06534.1_1 [gene=CENH3] [protein=centromere histone H
Database: Nippon
0 hits found
BLASTN 2.10.1+
Query: lcl|KU837266.1_cds_AOH73624.1_1 [gene=CENH3] [protein=centromere-specific I
Database: Nippon
0 hits found
BLASTN 2.10.1+
Query: lcl|KU837264.1_cds_AOH73622.1_1 [gene=CENH3] [protein=centromere-specific I
Database: Nippon
0 hits found
BLASTN 2.10.1+
Query: lcl|KU837263.1_cds_AOH73621.1_1 [gene=CENH3] [protein=centromere-specific I
Database: Nippon
0 hits found
BLASTN 2.10.1+
Query: lcl|KU837262.1_cds_AOH73620.1_1 [gene=CENH3] [protein=centromere-specific I
Database: Nippon
0 hits found
BLASTN 2.10.1+
Query: lcl|KU674827.1_cds_AOH73617.1_1 [gene=CENH3] [protein=centromere-specific I
Database: Nippon
0 hits found
BLASTN 2.10.1+
Query: lcl|KR029619.1_cds_ALF04640.1_1 [gene=CENH3] [protein=centromeric histone H
Database: Nippon
0 hits found
BLASTN 2.10.1+
Query: lcl|KR029618.1_cds_ALF04639.1_1 [gene=CENH3] [protein=centromeric histone H
Database: Nippon
0 hits found
BLASTN 2.10.1+
Query: lcl|KF214777.1_cds_AHH01567.1_1 [gene=CenH3] [protein=centromere-specific hi
Database: Nippon
0 hits found
BLASTN 2.10.1+
Query: lcl|KC491791.1_cds_AGQ21573.1_1 [gene=CENH3] [protein=centromere specific h
Database: Nippon
0 hits found

BLASTN 2.10.1+
Query: lcl|AY438639.1_cds_AAR85315.1_1 [gene=CenH3] [protein=centromeric histone 3
Database: Nippon
Fields: query acc.ver, subject acc.ver, % identity, alignment length, mismatches, gap oper
6 hits found
lcl|AY438639.1_cds_AAR85315.1_1 Chr5
lcl|AY438639.1_cds_AAR85315.1_1 Chr5
lcl|AY438639.1_cds_AAR85315.1_1 Chr5
lcl|AY438639.1_cds_AAR85315.1_1 Chr5
lcl|AY438639.1_cds_AAR85315.1_1 Chr5
lcl|AY438639.1_cds_AAR85315.1_1 Chr5
BLASTN 2.10.1+
Query: lcl|AF519807.2_cds_AAM74226.1_1 [protein=centromeric histone H3-like protein]
Database: Nippon
0 hits found
BLASTN 2.10.1+
Query: lcl|HM988988.1_cds_ADM18965.1_1 [gene=CENH3-B] [protein=centromeric histo
Database: Nippon
0 hits found
BLASTN 2.10.1+
Query: lcl|MH094618.1_cds_AYA72175.1_1 [protein=centromeric-specific histone H3 vari
Database: Nippon
0 hits found
BLASTN 2.10.1+
Query: lcl|MH182682.1_cds_AYA72192.1_1 [protein=centromeric-specific histone H3 vari
Database: Nippon
0 hits found
BLASTN 2.10.1+
Query: lcl|MH153697.1_cds_AYA72176.1_1 [protein=centromeric-specific histone H3 vari
Database: Nippon
0 hits found
BLASTN 2.10.1+
Query: lcl|MG384788.1_cds_AUN88474.1_1 [gene=betaCENH3] [protein=centromeric his
Database: Nippon
Fields: query acc.ver, subject acc.ver, % identity, alignment length, mismatches, gap oper
1 hits found
lcl|MG384788.1_cds_AUN88474.1_1 Chr5
BLASTN 2.10.1+
Query: lcl|MG384787.1_cds_AUN88473.1_1 [gene=betaCENH3] [protein=beta centromer
Database: Nippon
Fields: query acc.ver, subject acc.ver, % identity, alignment length, mismatches, gap oper
1 hits found
lcl|MG384787.1_cds_AUN88473.1_1 Chr5
BLASTN 2.10.1+
Query: lcl|MG384783.1_cds_AUN88469.1_1 [gene=betaCENH3] [protein=beta centromer
Database: Nippon
Fields: query acc.ver, subject acc.ver, % identity, alignment length, mismatches, gap oper
1 hits found

lcl|MG384783.1_cds_AUN88469.1_1 Chr5
BLASTN 2.10.1+
Query: lcl|MG384777.1_cds_AUN88463.1_1 [gene=betaCENH3-2] [protein=beta centrom
Database: Nippon
Fields: query acc.ver, subject acc.ver, % identity, alignment length, mismatches, gap oper
1 hits found
lcl|MG384777.1_cds_AUN88463.1_1 Chr5
BLASTN 2.10.1+
Query: lcl|KR676382.1_cds_ALK04343.1_1 [protein=centromeric histone H3 protein] [prot
Database: Nippon
0 hits found
BLASTN 2.10.1+
Query: lcl|KR676380.1_cds_ALK04341.1_1 [protein=centromeric histone H3 protein] [prot
Database: Nippon
0 hits found
BLASTN 2.10.1+
Query: lcl|AB793504.1_cds_BAO51832.1_1 [gene=TbCENH3] [protein=centromere specifi
Database: Nippon
0 hits found
BLASTN 2.10.1+
Query: lcl|AB678377.1_cds_BAM74170.1_1 [gene=PsCENH3A] [protein=centromere speci
Database: Nippon
0 hits found
BLASTN 2.10.1+
Query: lcl|AF465802.1_cds_AAL86777.1_1 [gene=HTR12] [protein=centromeric histone H
Database: Nippon
0 hits found
BLASTN 2.10.1+
Query: lcl|AF465800.1_cds_AAL86775.1_1 [gene=HTR12] [protein=centromeric histone H
Database: Nippon
0 hits found
BLAST processed 107 queries

enome assembly

ıs, q. start, q. end, s. start, s. end, evalue, bit score

97.037	135	4	0	165	299
97.196	107	3	0	1	107
100	78	0	0	371	448
97.531	81	1	1	297	376
100	53	0	0	449	501

ıs, q. start, q. end, s. start, s. end, evalue, bit score

96.35	137	5	0	163	299

95.238	105	5	0	1	105
100	78	0	0	371	448
98.765	81	0	1	297	376
100	53	0	0	449	501

ıs, q. start, q. end, s. start, s. end, evalue, bit score

90.179	112	8	2	1	112
92.308	78	6	0	362	439
94	50	3	0	440	489

ıs, q. start, q. end, s. start, s. end, evalue, bit score

94.545	110	3	2	1	110
93.59	78	5	0	362	439
94	50	3	0	440	489

ation=join(38..62,190..311,450..524,2427..2502,2783..2838)] [gbkey=CDS]

ation=join(38..68,209..330,472..546,2261..2336,2565..2620)] [gbkey=CDS]

ation=join(38..68,210..331,471..545,2958..3033,3309..3364)] [gbkey=CDS]

ation=join(42..72,215..336,549..623,1557..1632,1915..1970)] [gbkey=CDS]

ation=join(38..68,213..334,452..526,3607..3682,3961..4016)] [gbkey=CDS]

ation=join(42..72,215..336,548..622,3079..3154,3455..3510)] [gbkey=CDS]

ation=join(33..75,167..288,453..527,1327..1402,1523..1578)] [gbkey=CDS]

ation=join(27..69,179..300,1448..1522,2923..2998,3117..3172)] [gbkey=CDS]

ation=join(26..68,188..309,517..591,3224..3299,3418..3473)] [gbkey=CDS]

ation=join(26..68,188..309,517..591,3224..3299,3418..3473)] [gbkey=CDS]

ation=join(26..68,189..310,549..623,2260..2335,2453..2508)] [gbkey=CDS]

ation=join(13..43,159..280,432..506,2692..2767,3048..3103)] [gbkey=CDS]

ation=join(103..133,278..399,517..591,2974..3049,3309..3364)] [gbkey=CDS]

ation=join(35..77,194..315,580..654,3340..3415,3533..3588)] [gbkey=CDS]

] [protein_id=AHW98238.1] [location=1..372] [gbkey=CDS]

] [protein_id=AHW98237.1] [location=1..372] [gbkey=CDS]

] [protein_id=AHW98236.1] [location=1..372] [gbkey=CDS]

] [protein_id=AHW98234.1] [location=1..372] [gbkey=CDS]

] [protein_id=AHW98229.1] [location=1..474] [gbkey=CDS]

] [protein_id=AHW98228.1] [location=1..474] [gbkey=CDS]

H3 variant protein] [protein_id=ADN92693.1] [location=153..704] [gbkey=CDS]

romeric protein B] [protein_id=QGY64363.1] [location=24..500] [gbkey=CDS]

romeric protein A] [protein_id=QGY64362.1] [location=24..500] [gbkey=CDS]

romeric protein 2] [protein_id=QGY64360.1] [location=106..555] [gbkey=CDS]

romeric protein 1A] [protein_id=QGY64359.1] [location=123..569] [gbkey=CDS]

romeric protein 1B] [protein_id=QGY64358.1] [location=161..607] [gbkey=CDS]

istone H3] [protein_id=AUN88460.1] [location=1..501] [gbkey=CDS]

histone 3] [protein_id=AMH40810.1] [location=1..504] [gbkey=CDS]

histone 3] [protein_id=AMH40809.1] [location=1..504] [gbkey=CDS]

stone 3] [protein_id=AKI32619.1] [location=1..471] [gbkey=CDS]

stone 3] [protein_id=AKI32617.1] [location=1..306] [gbkey=CDS]

stone 3] [protein_id=AKI32616.1] [location=1..471] [gbkey=CDS]

stone 3] [protein_id=AKI32608.1] [location=1..219] [gbkey=CDS]

stone 3] [protein_id=AKI32602.1] [location=1..477] [gbkey=CDS]

stone 3] [protein_id=AKI32601.1] [location=1..471] [gbkey=CDS]

-I3 isoform b] [protein_id=BAP26971.1] [location=1..513] [gbkey=CDS]

-I3 isoform a] [protein_id=BAP26970.1] [location=1..492] [gbkey=CDS]

| [protein_id=AEH95352.1] [location=1..492] [gbkey=CDS]

| [protein_id=AEH95351.1] [location=1..492] [gbkey=CDS]

| [protein_id=AEH95350.1] [location=1..501] [gbkey=CDS]

ıne H3] [protein_id=AEK21394.1] [location=1..411] [gbkey=CDS]

ıne H3] [protein_id=AEK21393.1] [location=44..463] [gbkey=CDS]

-I3 variant protein] [protein_id=ACZ04984.1] [location=1..546] [gbkey=CDS]

-I3 variant protein] [protein_id=ACZ04982.1] [location=1..555] [gbkey=CDS]

-I3 variant protein] [protein_id=ACZ04980.1] [location=1..549] [gbkey=CDS]

-I3 variant protein] [protein_id=ACZ04978.1] [location=1..489] [gbkey=CDS]

histone H3] [protein_id=QBB85873.1] [location=join(1..52,159..205,292..343,424..461,556..5

histone H3-3 variant] [protein_id=QBB85871.1] [location=1..537] [gbkey=CDS]

l3 variant 2] [protein_id=AOR06535.1] [location=1..525] [gbkey=CDS]

l3 variant 1] [protein_id=AOR06534.1] [location=1..549] [gbkey=CDS]

histone H3-4 variant] [protein_id=AOH73624.1] [location=124..705] [gbkey=CDS]

histone H3-2] [protein_id=AOH73622.1] [location=72..611] [gbkey=CDS]

histone H3-2] [protein_id=AOH73621.1] [location=107..655] [gbkey=CDS]

histone H3] [protein_id=AOH73620.1] [location=167..652] [gbkey=CDS]

histone H3] [protein_id=AOH73617.1] [location=1..555] [gbkey=CDS]

3 variant 2] [protein_id=ALF04640.1] [location=29..565] [gbkey=CDS]

3 variant 1] [protein_id=ALF04639.1] [location=29..565] [gbkey=CDS]

istone H3 variant] [protein_id=AHH01567.1] [location=61..540] [gbkey=CDS]

histone H3 variant] [protein_id=AGQ21573.1] [location=66..557] [gbkey=CDS]

] [protein_id=AAR85315.1] [location=68..562] [gbkey=CDS]

ıs, q. start, q. end, s. start, s. end, evalue, bit score

100	136	0	0	158	293
100	107	0	0	1	107
100	78	0	0	365	442
98.765	81	0	1	291	370
100	53	0	0	443	495
100	30	0	0	132	161

[protein_id=AAM74226.1] [location=66..539] [gbkey=CDS]

ne H3 isoform B] [protein_id=ADM18965.1] [location=1..534] [gbkey=CDS]

ant] [protein_id=AYA72175.1] [location=11..475] [gbkey=CDS]

ant] [protein_id=AYA72192.1] [location=15..479] [gbkey=CDS]

ant] [protein_id=AYA72176.1] [location=30..494] [gbkey=CDS]

tone H3] [protein_id=AUN88474.1] [location=1..456] [gbkey=CDS]

ıs, q. start, q. end, s. start, s. end, evalue, bit score

84.615	78	12	0	326	403

ic histone H3] [protein_id=AUN88473.1] [location=1..456] [gbkey=CDS]

ıs, q. start, q. end, s. start, s. end, evalue, bit score

84.615	78	12	0	326	403

ic histone H3] [protein_id=AUN88469.1] [location=1..456] [gbkey=CDS]

ıs, q. start, q. end, s. start, s. end, evalue, bit score

84.615	78	12	0	326	403

eric histone H3] [protein_id=AUN88463.1] [location=1..462] [gbkey=CDS]

ıs, q. start, q. end, s. start, s. end, evalue, bit score

84.615	78	12	0	332	409

ein_id=ALK04343.1] [location=103..633] [gbkey=CDS]

ein_id=ALK04341.1] [location=59..583] [gbkey=CDS]

c histone H3] [protein_id=BAO51832.1] [location=1..459] [gbkey=CDS]

ific histone H3 variant] [protein_id=BAM74170.1] [location=61..432] [gbkey=CDS]

3 HTR12] [protein_id=AAL86777.1] [location=1..531] [gbkey=CDS]

3 HTR12] [protein_id=AAL86775.1] [location=1..537] [gbkey=CDS]

24069185	24069051	3.43E-58	228
24070126	24070020	2.71E-44	182
24068448	24068371	3.55E-33	145
24068959	24068879	5.95E-31	137
24068013	24067961	2.81E-19	99

24069187	24069051	1.23E-57	226

24070126	24070022	7.58E-40	167
24068448	24068371	3.55E-33	145
24068959	24068879	1.28E-32	143
24068013	24067961	2.81E-19	99

24070126	24070018	1.26E-32	143
24068448	24068371	3.56E-23	111
24068013	24067964	1.30E-12	76.8

24070126	24070020	7.49E-40	167
24068448	24068371	7.65E-25	117
24068013	24067964	1.30E-12	76.8

95,665..780,920..997,1099..1174,1255..1310)] [gbkey=CDS]

24069186	24069051	2.01E-65	252
24070126	24070020	2.65E-49	198
24068448	24068371	3.51E-33	145
24068959	24068879	1.26E-32	143
24068013	24067961	2.77E-19	99
24069753	24069724	1.69E-06	56.5

24068448	24068371	3.31E-13	78.7

24068448	24068371	3.31E-13	78.7

24068448	24068371	3.31E-13	78.7
24068448	24068371	3.35E-13	78.7
