# Supplementary material for: Uncovering natural allelic and structural variants of OsCENH3 gene by targeted resequencing and in silico mining in genus Oryza
Source: Sci Rep. 2023 Jan 16;13:830. doi: 10.1038/s41598-023-28053-w (PMC9842635; doi:10.1038/s41598-023-28053-w)
Supplement: Supplementary file 5 — Supplementary Table S5. [file 41598_2023_28053_MOESM5_ESM.pdf]

Supplementary Table S5: OsCENH3 variants extracted from SNPseek and HDRA panels

SNPseek

rs#	reference	pos	effect	alleles	chrom	genic pos	strand
175192445	C	24067759	3'utr	C/T	5	2435	+
175192933	G	24068247	Intronic	G/T	5	1947	+
175193154	T	24068468	Cys > Ser	T/A	5	1726	+
175193398	C	24068712	intronic	C/T	5	1482	+
175194292	A	24069606	intronic	A/G	5	588	+
175194553	G	24069867	intronic	G/A	5	327	+
175194581	G	24069895	intronic	G/A	5	299	+
175194744	T	24070058	intronic	T/C	5	136	+

HDRA

rs	Reference	pos	effect	alleles	chrom	enic positic	strand
5655822	C	24069122	syn	T/C	5	1072	-

assembly#	center	protLSID	assayLSID	panelLSID	QCcode	B001_B00	B002_B00	B003_B00
NA	NA	NA	NA	NA	NA	CC	CC	CC
NA	NA	NA	NA	NA	NA	GG	GG	GG
NA	NA	NA	NA	NA	NA	TT	TT	TT
NA	NA	NA	NA	NA	NA	CC	CC	CC
NA	NA	NA	NA	NA	NA	AA	AA	AA
NA	NA	NA	NA	NA	NA	GG	GG	GG
NA	NA	NA	NA	NA	NA	GG	GG	GG
NA	NA	NA	NA	NA	NA	TT	TT	TT

assembly	center	protLSID	assayLSID	panelLSID	QCcode	252580DN	252580DN	252580DN
NA	NA	NA	NA	NA	NA	AA	GG	AA

B004_B00	B005_B00	B006_B00	B007_B00	B008_B00	B009_B00	B010_B01	B011_B01	B012_B01
CC	CC	TT	TT	CC	TT	TT	TT	TT
GG	GG	GG	GG	GG	GG	GG	GG	GG
TT	TT	AA	AA	TT	AA	AA	AA	AA
CC	CC	CC	CC	CC	CC	CC	CC	CC
AA	AA	GG	GG	AA	GG	GG	GG	GG
GG	GG	AA	AA	GG	AA	AA	AA	AA
GG	GG	GG	GG	GG	GG	GG	GG	GG
TT	TT	CC	CC	TT	CC	CC	CC	CC

252580DN.	252580DN.	252580DN.	252580DN.	252580DN.	252580DN.	252580DN.	252580DN.	252580DN.
GG	GG	AA	GG	GG	AA	AA	AA	AA

B013_B01	B014_B01	B015_B01	B016_B01	B017_B01	B018_B01	B019_B01	B020_B02	B021_B02
TT	CC	TT	CC	CC	CC	TT	CC	TT
GG	GG	GG	GG	GG	GG	GG	GG	GG
AA	TT	AA	TT	TT	TT	AA	TT	AA
CC	CC	CC	CC	CC	CC	CC	CC	CC
GG	NN	GG	AA	AA	AA	GG	AA	GG
AA	GG	AA	GG	GG	GG	AA	GG	AA
GG	GG	GG	GG	GG	GG	GG	GG	GG
CC	NN	CC	TT	TT	TT	CC	TT	CC

252580DN.	252580DN.	252580DN.	252580DN.	252580DN.	252580DN.	252580DN.	252580DN.	252580DN.
GG	AA	GG	GG	AA	AA	AA	--	--

B023_B02	B024_B02	B025_B02	B026_B02	B027_B02	B028_B02	B029_B02	B030_B03	B031_B03
CC	TT	CC	TT	TT	TT	TT	TT	TT
GG	GG	GG	GG	GG	GG	GG	GG	GG
TT	AA	TT	AA	AA	AA	AT	AA	AA
CC	CC	CC	CC	CC	CC	CC	CC	CC
AA	GG	AA	GG	GG	GG	GA	GG	GG
GG	AA	GG	AA	AA	AA	AA	NN	AA
GG	GG	GG	GG	GG	GG	GG	GG	GG
TT	CC	TT	CC	CC	CC	CT	NN	CC

252580DN	252580DN	252580DN	252580DN	252580DN	252580DN	252580DN	252580DN	252580DN
AA	AA	AA	AA	GG	AA	AA	AA	AA

B032_B03	B033_B03	B034_B03	B035_B03	B036_B03	B037_B03	B038_B03	B039_B03	B040_B04
TT	TT	CC	TT	CC	NN	CC	TC	TT
GG	GG	GG	GG	GG	GG	GG	GG	GG
AA	AA	TT	AA	TT	TT	TT	AT	AA
CC	CC	CC	CC	CC	CC	CC	CC	CC
GG	GG	AA	GG	AA	AA	AA	GG	GG
AA	AA	GG	AA	GG	GG	NN	AG	AA
GG	GG	GG	GG	GG	GG	GG	GG	GG
CC	CC	NN	CC	TT	TT	TT	CC	CC

252580DN	252580DN	252580DN	252580DN	252580DN	252580DN	252580DN	252580DN	252580DN
AA	AA	GG	AA	AA	AA	AA	AA	AA

B043_B04	B044_B04	B045_B04	B046_B04	B047_B04	B048_B04	B049_B04	B051_B05	B052_B05
CC	TT	CC	CC	NN	TT	TC	TT	TT
GG	GG	GG	GG	GG	GG	GG	GG	GG
NN	AA	TT	TT	NN	AA	AA	AA	AA
CC	CC	CC	CC	CC	CC	CC	CC	CC
AA	GG	AA	AA	NN	GG	GA	GG	GG
GG	NN	GG	GG	NN	NN	AA	AA	AA
GG	GG	GG	GG	GG	NN	AA	GG	GG
TT	CC	TT	TT	TT	CC	CC	CC	CC

252580DN.	252580DN.	252580DN.	252580DN.	252580DN.	252580DN.	252580DN.	252580DN.	252580DN
AA	GG	AA	AA	GG	AA	AA	AA	AA

B053_B05	B054_B05	B055_B05	B056_B05	B057_B05	B058_B05	B059_B05	B060_B06	B061_B06
CC	CC	CC	CC	CC	TT	TT	TT	TT
GG	GG	GG	GG	GG	GG	GG	GG	GG
TT	TT	TT	NN	TT	AA	AA	AA	AA
CC	CC	CC	CC	CC	CC	CC	CC	CC
AA	AA	AA	AA	AA	GG	GG	GG	GG
GG	GG	NN	GG	GG	AA	AA	AA	AA
GG	GG	GG	GG	GG	GG	GG	GG	GG
TT	TT	TT	TT	TT	CC	CC	CC	CC

252580DN	252580DN	252580DN	252580DN	252580DN	252580DN	252580DN	252580DN	252580DN
AA	AA	AA	AA	AA	AA	AA	AA	AA

B062_B06	B063_B06	B064_B06	B065_B06	B066_B06	B067_B06	B068_B06	B069_B06	B070_B07
TT	TT	TT	TT	CC	TT	CC	CC	CC
GG	GG	GG	GG	GG	GG	GG	GG	GG
AA	AA	AA	AA	TT	AA	TT	TT	TT
CC	CC	CC	CC	CC	CC	CC	CC	CC
GG	GG	GG	GG	AA	GA	AA	AA	AA
AA	AA	AA	AA	GG	AA	GG	GG	GG
GG	GG	GG	GG	GG	GG	GG	GG	GG
CC	CC	CC	CC	TT	CC	TT	TT	TT

252580DN	252580DN	252580DN	252580DN	252580DN	252580DN	252580DN	252580DN	252580DN
AA	GG	AA	GG	GG	AA	AA	AA	AA

B071_B07	B072_B07	B073_B07	B074_B07	B075_B07	B076_B07	B077_B07	B079_B07	B081_B08
CC	TC	TT	TT	TT	TT	CC	TT	TT
GG	GG	GG	GG	GG	GG	GG	GG	GG
TT	AT	AA	AA	AA	AA	TT	AA	AA
CC	CC	CC	CC	CC	CC	CC	CC	CC
AA	GG	GG	GG	GG	GG	AA	GG	GG
GG	AA	AA	AA	AA	AA	GG	AA	AA
GG	GG	GG	GG	GG	GG	GG	GG	GG
TT	CC	CC	CC	CC	CC	TT	CC	CC

252580DN	252580DN	252580DN	252580DN	252580DN	252580DN	252580DN	252580DN	252580DN
AA	AA	GG	GG	GG	AA	GG	GG	GG

B082_B08	B083_B08	B084_B08	B085_B08	B086_B08	B087_B08	B088_B08	B089_B08	B090_B09
TT	TT	CC	TT	CC	TT	TT	TT	TT
GG	GG	GG	GG	GG	GG	GG	GG	GG
AA	AA	TT	AA	TT	AA	AA	AA	AA
CC	CC	CC	CC	CC	CC	CC	CC	CC
GG	GG	AA	GG	AA	GG	GG	GA	GG
AA	AA	GG	AA	GG	AA	AA	AA	AA
GG	GG	GG	GG	GG	GG	GG	GG	GG
CC	CC	TT	CC	TT	CC	CC	CT	CC

252580DN.	252580DN.	252580DN.	252580DN.	252580DN.	252580DN.	252580DN.	252580DN.	252580DN.
AA	AA	AA	AA	GG	AA	AA	GG	GG

B091_B09	B092_B09	B093_B09	B094_B09	B095_B09	B096_B09	B097_B09	B100_B10	B101_B10
TT	TT	TT	TT	TT	TT	TT	CC	CC
GG	GG	GG	GG	GG	GG	GG	GG	GG
AA	AA	AA	AA	AA	AA	AA	TT	NN
CC	CC	CC	CC	CC	CC	CC	CC	CC
GG	NN	GG	GG	GG	GG	GG	AA	AA
AA	AA	AA	AA	AA	AA	AA	GG	GG
GG	GG	GG	GG	GG	GG	GG	GG	GG
CC	CT	CC	CC	CC	CC	CC	TT	CT

252580DN	252580DN	252580DN	252580DN	252580DN	252580DN	252580DN	252580DN	252580DN
AA	GG	GG	AA	GG	AA	AA	GG	GG

B102_B10	B103_B10	B104_B10	B105_B10	B106_B10	B107_B10	B108_B10	B109_B10	B110_B11
CC	CC	TT	TT	TT	TT	TT	CC	NN
GG	GG	GG	GG	GG	GG	GG	GG	GG
TT	TT	AA	AA	AA	AA	AA	NN	TT
CC	CC	CC	CC	CC	CC	CC	CC	CC
AA	AA	GG	GG	GG	GG	GG	AA	AA
GG	NN	AA	AA	AA	AA	AA	GG	GG
GG	GG	GG	GG	GG	GG	GG	GG	GG
TT	TT	CC	CC	CC	CC	CC	TT	TT

252580DN	252580DN	9710032R	9710032R	9710032R	9710032R	9710032R	9710032R	9710032R
AA	AA	GG	AA	AA	AA	GG	AA	AA

B111_B11	B112_B11	B113_B11	B114_B11	B115_B11	B116_B11	B117_B11	B118_B11	B119_B11
CC	TT	TT	TT	TT	TT	CC	TT	TT
GG	GG	GG	GG	GG	GG	GG	GG	GG
TT	AA	AA	AA	AA	AA	TT	AA	AA
CC	CC	CC	CC	CC	CC	CC	CC	CC
AA	GG	GG	GG	GG	GG	AA	GG	GG
NN	AA	AA	AA	AA	AA	GG	AA	AA
GG	GG	GG	GG	GG	GG	GG	GG	GG
TT	CC	CC	CC	CC	CC	TT	CC	CC

9710032R	9710032R	9710032R	9710032R	9710032R	9710032R	9710032R	9710032R	9710032R
AA	GG	GG	GG	AA	AA	GG	GG	AA

B120_B12	B121_B12	B122_B12	B123_B12	B124_B12	B125_B12	B126_B12	B127_B12	B128_B12
TT	TT	CC	TT	NN	TT	TT	TT	TT
GG	GG	GG	GG	GG	GG	GG	GG	GG
AA	AA	TT	AA	TT	AA	AA	AA	AA
CC	CC	CC	CC	CC	CC	CC	CC	CC
GG	GG	AA	GG	AA	GG	GG	GG	GG
NN	AA	GG	AG	GG	AA	AA	AA	AA
GG	GG	GG	GG	GG	GG	GG	GG	GG
CC	CC	TT	CC	NN	CC	CC	CC	CC

.9710032R	.9710032R	.9710032R	.9710032R	.9710032R	.9710032R	.9710032R	.9710032R	.9710137R
AA	AA	GG	AA	AA	AA	AA	AA	AA

B129_B12	B130_B13	B131_B13	B132_B13	B133_B13	B134_B13	B135_B13	B136_B13	B137_B13
TT	TT	TT	TT	TT	CC	TT	CC	TT
GG	GG	GG	GG	GG	GG	GG	GG	GG
AA	AT	AA	AA	AA	TT	AA	TT	AA
CC	CC	CC	CC	CC	CC	CC	CC	CC
GG	GA	GG	GG	GG	NN	GG	AA	GA
AA	AA	AA	AA	AA	GG	AA	GG	AA
GG	GG	GG	GG	GG	GG	GG	GG	GG
CC	CC	CC	CC	CC	TT	CC	TT	NN

[illegible]9710137R[illegible]	[illegible]9710137R[illegible]	[illegible]9710137R[illegible]	[illegible]9710137R[illegible]	[illegible]9710137R[illegible]	[illegible]9710137R[illegible]	[illegible]9710137R[illegible]	[illegible]9710137R[illegible]	[illegible]9710137R[illegible]
GG	GG	AA	AA	AA	GG	AA	GG	GG

B138_B13	B139_B13	B140_B14	B141_B14	B142_B14	B143_B14	B144_B14	B145_B14	B146_B14
NN	TC	TT	TT	CC	CC	CC	TT	TT
GG	GG	GG	GG	GG	GG	GG	GG	GG
NN	AT	AA	AA	TT	TT	TT	AA	AA
CC	CC	CC	CC	CC	CC	CC	CC	CC
AA	GA	GG	GG	AA	AA	AA	GA	GG
GG	NN	AA	AA	GG	GG	GG	AA	AA
GG	GG	GG	GG	GG	GG	GG	GG	GG
NN	CT	CC	CC	TT	TT	TT	NN	NN

9710137R	9710137R	9710137R	9710137R	9710137R	9710137R	9710137R	9710137R	9710137R
GG	GG	AA	GG	AG	GG	AA	AA	GG

B147_B14	B148_B14	B149_B14	B150_B15	B151_B15	B152_B15	B153_B15	B154_B15	B155_B15
TC	CC	TT	TT	TT	NN	TT	CC	TT
GG	GG	GG	GG	GG	GG	GG	GG	GG
AA	TT	AA	AA	AA	AT	AA	TT	AA
CC	CC	CC	CC	CC	CC	CC	CC	CC
GG	AA	GG	GG	GG	NN	GG	AA	GG
AA	GG	AA	AA	AA	NN	AA	GG	AA
GG	GG	GG	GG	GG	GG	NN	GG	GG
CC	TT	CC	CC	CC	TT	CC	NN	CC

9710137R	9710137R	9710137R	9710137R	9710137R	PURPLE	inShanZim	N32	Dular
AA	AA	AA	GG	AA	GG	GG	AA	AA

B156_B15	B157_B15	B158_B15	B159_B15	B160_B16	B161_B16	B162_B16	B163_B16	B164_B16
TT	TT	TC	TT	CC	CC	CC	TT	TT
GG	GG	GG	GG	GG	GG	GG	GG	GG
AA	AA	AT	AA	TT	TT	TT	AA	AA
CC	CC	CC	CC	CC	CC	CC	CC	CC
GG	GG	GA	GG	AA	AA	AA	GG	GG
AA	AA	AG	AA	GG	GG	GG	AA	AA
GG	GG	GG	GG	GG	GG	GG	GG	GG
CC	CC	NN	CC	TT	TT	TT	CC	CC

ashikalmiA	ataktaraAu	Bala	Angkrang	SravPrapay	B450IBP62	B450IBP16	PanJu	halaShaitt
GG	GG	AA	GG	GG	AA	AA	GG	GG

B165_B16	B166_B16	B167_B16	B168_B16	B169_B16	B170_B17	B171_B17	B173_B17	B176_B17
TT	CC	CC	CC	CC	CC	CC	TT	TT
GG	GG	GG	GG	GG	GG	GG	GG	GG
AA	TT	TT	TT	TT	TT	TT	AA	AA
CC	CC	CC	CC	CC	CC	CC	CC	CC
GG	NN	AA	AA	AA	AA	AA	GG	GG
AA	GG	GG	GG	GG	GG	GG	AA	AA
GG	GG	GG	GG	GG	GG	GG	GG	GG
CC	TT	TT	TT	TT	TT	TT	CC	CC

EarlyNo1	SelNo388	IMIZUMO	Charmarum	Dharial	ARC6578	TiaHeret	Pelu	Sufaida
GG	AA	AA	AA	AA	GG	AA	GG	GG

B179_B17	B180_B18	B181_B18	B182_B18	B183_B18	B184_B18	B185_B18	B187_B18	B188_B18
CC	TT	TT	CC	CC	TT	TT	TT	CC
GG	GG	GG	GG	GG	GG	GG	GG	GG
TT	AA	AA	TT	TT	AA	AA	AA	TT
CC	CC	CC	CC	CC	CC	CC	CC	CC
AA	GG	GG	AA	AA	GG	GG	GG	AA
GG	AA	NN	GG	GG	AA	AA	NN	GG
GG	GG	NN	GG	GG	GG	GG	NN	GG
TT	CC	CC	TT	TT	CC	CC	CC	TT

Mahlar	P22	Ziri	Sathra	ChuehTaPai	N22	Sigoendaba	Sabharaj	ASWINA330
GG	AA	AA	GG	GG	AA	AA	GG	AA

B189_B18	B190_B19	B191_B19	B192_B19	B193_B19	B194_B19	B195_B19	B196_B19	B197_B19
CC	CC	CC	TT	TT	TT	TT	CC	TT
GG	GG	GG	GG	GG	GG	GG	GG	GG
TT	TT	TT	AA	AA	AA	AA	TT	AA
CC	CC	CC	CC	CC	CC	CC	CC	CC
AA	AA	AA	GG	GG	GG	GG	AA	GG
GG	GG	GG	AA	NN	AA	AA	GG	AA
GG	GG	GG	GG	NN	GG	GG	GG	GG
TT	TT	TT	CC	CC	CC	CC	TT	CC

DNJ151	SUNGLIAO	andeupKan	Banajira	Brondol	DJ90	DJ102	amDawkM	DV132
GG	AA	GG	GG	AA	AA	AA	GG	AA

B198_B19	B199_B19	B200_B20	B201_B20	B202_B20	B203_B20	B204_B20	B205_B20	B207_B20
TT	CC	TT	TT	TT	TT	CC	CC	TT
GG	GG	GG	GG	GG	GG	GG	GG	GG
AA	TT	AA	AA	AA	AA	NN	TT	AA
CC	CC	CC	CC	CC	CC	CC	CC	CC
GG	AA	GG	GG	GG	GG	AA	AA	GG
AA	GG	AA	AA	NN	AA	GG	GG	AA
GG	GG	GG	GG	GG	GG	GG	GG	GG
CC	TT	CC	CC	CC	NN	TT	TT	CC

GubuhBala	Bengawan	Sug	Calmochi10	Pakhedhan	Ghorbhai	AkTokhum	MBRAPA12	Jefferson
AA	GG	AA	AA	AA	GG	AA	AA	AA

B208_B20	B210_B21	B212_B21	B213_B21	B214_B21	B215_B21	B216_B21	B217_B21	B218_B21
TT	TT	CC	TT	TT	CC	TT	TT	CC
GG	GG	GG	GG	GG	GG	GG	GG	GG
AA	AA	TT	AA	AA	TT	AA	AA	TT
CC	CC	CC	CC	CC	CC	CC	CC	CC
GG	GG	AA	GG	GG	AA	GG	GG	AA
AA	AA	GG	AA	AA	GG	AA	AA	GG
GG	GG	GG	GG	GG	GG	GG	GG	GG
CC	CC	TT	CC	CC	TT	CC	CC	TT

BhuwaDhar	Caucasica	Vulgaris	ARPASHAL	UZROSZ5	KyzylShala	MMANAN	ShatoLua	Kabre
AA	AA	AA	AA	AA	AA	GG	GG	AA

B219_B21	B221_B22	B222_B22	B223_B22	B224_B22	B225_B22	B226_B22	B227_B22	B228_B22
TT	TT	TT	CC	TT	CC	CC	TT	CC
GG	GG	GG	GG	GG	GG	GG	GG	GG
AA	AA	AA	TT	AA	TT	TT	AA	TT
CC	CC	CC	CC	CC	CC	CC	CC	CC
GG	GG	GG	AA	GG	AA	AA	GA	AA
AA	AA	AA	GG	AA	GG	GG	AA	GG
GG	GG	GG	GG	GG	GG	GG	GG	GG
CC	CC	CT	TT	CT	TT	TT	NN	TT

Atemo	Dulugu	Boma	TOg5603	TOg5882	TOg6231	TOg6238	RGC10357	4501113P4
AA	AA	AA	AA	GG	AA	AA	AA	AA

B229_B22	B230_B23	B231_B23	B232_B23	B233_B23	B234_B23	B235_B23	B236_B23	B238_B23
TT	CC	TT	TT	TT	TT	CC	CC	TT
GG	GG	GG	GG	GG	GG	GG	GG	GG
AA	TT	AA	AA	AA	AA	TT	TT	AA
CC	CC	CC	CC	CC	CC	CC	CC	CC
GG	AA	GG	GG	GG	GG	AA	AA	GG
AA	GG	NN	AA	AA	AA	GG	GG	AA
GG	GG	GG	GG	GG	GG	GG	GG	GG
CC	TT	CC	CC	CC	CC	TT	TT	CC

4502423P	450243P3	4502432P	B450IBP2	B450IBP3	Kharsu	ongGnarJir	layangKhar	ryPatnaOri
AA	AA	AA	AG	AA	AA	AA	GG	AA

B239_B23	B240_B24	B241_B24	B242_B24	B243_B24	B244_B24	B245_B24	B246_B24	B247_B24
TT	CC	CC	CC	TT	TT	CC	TT	TT
GG	GG	GG	GG	GG	GG	GG	GG	GG
AA	TT	TT	TT	AA	AA	TT	AA	AA
CC	CC	CC	CC	CC	CC	CC	CC	CC
GG	AA	AA	AA	GG	GG	AA	GG	GG
AA	GG	GG	GG	AA	AA	GG	AA	AA
GG	GG	GG	GG	GG	GG	GG	GG	GG
CC	TT	TT	TT	CC	CC	TT	CC	CC

SpinMere	canodoBra	Sigadis	lKhoshaCer	GHRAIBA	KhaoPhoi	Karayal	PURPLERep	C8447
AA	AA	AA	AA	AA	GG	AA	GG	AA

B248_B24	B249_B24	B250_B25	B252_B25	B253_B25	B254_B25	B255_B25	B258_B25	B259_B25
TT	TT	CC	TT	TT	TT	TT	CC	TT
GG	GG	GG	GG	GG	GG	GG	GG	GG
AA	AA	TT	AA	AA	AA	AA	TT	AA
CC	CC	CC	CC	CC	CC	CC	CC	CC
GG	GG	AA	GG	GG	GG	GG	AA	GG
AA	AA	GG	AA	AA	AA	AA	GG	AA
GG	GG	GG	GG	GG	GG	GG	GG	GG
CC	CC	TT	CC	CC	CC	CC	TT	CC

diPohonBa	Ratna	Jhona	HeoTrang	ipirasikkan	Lantjang	AliCombo	Torh	Sugdasi
AA	GG	GG	GG	AA	GG	GG	GG	AA

B260_B26	B261_B26	B263_B26	B264_B26	B265_B26	B266_B26	B267_B26	B268_B26	B269_B26
TT	TT	CC	TT	TT	CC	TT	TT	CC
GG	GG	GG	GG	GG	GG	GG	GG	GG
AA	AA	TT	AA	AA	TT	AA	AA	TT
CC	CC	CC	CC	CC	CC	CC	CC	CC
GG	GG	AA	GG	GG	AA	GG	GG	AA
AA	AA	GG	AA	AA	GG	AA	AA	GG
GG	GG	GG	GG	GG	GG	GG	GG	GG
CC	CC	TT	CC	CC	TT	CC	CC	TT

JiraShahi	KerrSail	AS46	Janeri	uningTing	KoiMurali	Angana	DJ53	DM55
GG	GG	AA	GG	AA	AA	AA	GG	GG

CX2_CX2	CX3_CX3	CX4_CX4	CX5_CX5	CX6_CX6	CX8_CX8	CX9_CX9	CX10_CX1(	CX11_CX1:
TT	TT	TC	TT	CC	TT	TT	TT	NN
GG	GG	GG	GG	GG	GG	GG	GG	GG
AT	AA	AT	AA	TT	AA	AA	AA	AT
CC	CC	CC	CC	CC	CC	CC	CC	CC
GG	GG	GA	GG	AA	GG	GG	GG	AA
AA	AA	AG	AA	GG	AA	AA	AA	NN
GG	GG	GG	NN	GG	GG	GG	GG	GG
CC	CC	CT	CC	TT	CC	CC	CC	NN

DV85	79	udzaiField	M202	IPPONBAR	AcharDhog	JhingaSail	BakTushi	Gambir
AA	AA	AA	AA	AA	GG	GG	AA	AA

CX12_CX1	CX13_CX1	CX14_CX1	CX15_CX1	CX16_CX1	CX17_CX1	CX18_CX1	CX19_CX1	CX20_CX2
TT	TT	CC	TT	CC	TT	TT	TT	TT
GG	GG	GG	GG	GG	GG	GG	GG	GG
AA	AT	TT	AA	TT	AA	NN	AA	AA
CC	CC	CC	CC	CC	CC	CC	CC	CC
GG	NN	AA	GG	AA	GG	NN	GG	GG
AA	AG	GG	AA	GG	AA	AA	AA	AA
GG	GG	GG	GG	GG	GG	GG	GG	GG
CC	CC	TT	CC	TT	CC	CC	CC	CC

Shoni	FIROOZ	Kalamkati	DomZard	Sirkat	eritingTing	ShaliiLuk	Berenj	2
AA	AA	GG	AA	AA	AA	GG	GG	AA

CX21_CX2	CX22_CX2	CX23_CX2	CX24_CX2	CX25_CX2	CX26_CX2	CX27_CX2	CX28_CX2	CX29_CX2
TT	TT	TT	TT	TT	TT	TT	TT	TT
GG	GG	GG	GG	GG	GG	GG	GG	GG
AA	AA	AA	AA	AA	AA	AA	AA	AA
CC	CC	CC	CC	CC	CC	CC	CC	CC
GG	GG	GG	GG	GG	GG	GG	GG	GG
AA	AG	AA	AA	AA	AA	AA	AA	AA
GG	GG	GG	GG	GG	GG	GG	GG	GG
CC	CC	CC	CC	CC	CC	NN	CC	CC

afutKhosh	evkatiKunc	UzRosz17	JZROSZ26	JzRosz274	JzRosz283	Vrosz213	JZROSZM	UzRoszM9
AA	GG	AA	AA	AA	AA	AA	AA	AA

CX30_CX3(	CX31_CX3:	CX32_CX3:	CX33_CX3:	CX34_CX3	CX35_CX3!	CX37_CX3	CX42_CX4:	CX43_CX4:
TT	TT	TT	TT	TT	TT	TT	TT	TT
GG	GG	GG	GG	GG	GG	GG	GG	GG
AA	AA	AA	AA	AA	AA	AA	AA	AA
CC	CC	CC	CC	CC	CC	CC	CC	CC
GG	GG	GG	GG	GG	GG	GG	GG	GG
AA	AA	AA	AA	AA	AA	AA	AA	AA
GG	GG	GG	GG	GG	GG	GG	GG	GG
CC	CC	CC	CC	CC	CC	CC	CC	CC

UzRosz215	Nahodka	VILKIDZIRE	Primorsk6	KUBAN3	UzRos275	AZROS637	RIMASALI	nbarbyWhi
AA	AA	AA	AA	AA	AA	AA	AA	AA

CX44_CX4	CX45_CX4	CX46_CX4	CX47_CX4	CX48_CX4	CX49_CX4	CX50_CX5	CX51_CX5	CX52_CX5
TT	TT	TT	CC	TT	CC	TT	TC	TT
GG	GG	GG	GG	GG	GG	GG	GG	GG
AA	AA	AA	TT	AA	NN	AA	AA	AA
CC	CC	CC	CC	CC	CC	CC	CC	CC
GG	GG	GG	AA	GA	AA	NN	GG	GG
AA	AA	AA	GG	AA	NN	AA	AA	AA
GG	GG	GG	GG	GG	NN	GG	GG	GG
CC	CC	CC	TT	CC	TT	CC	NN	CC

Shirkati	udiShortG	MaienGarn	Jubilejnyj	eraWadi14	Cat1747	P817	Y2272	risKoChAzp
GG	AA	GG	AG	GG	GG	GG	GG	GG

CX53_CX53	CX54_CX54	CX55_CX55	CX56_CX56	CX57_CX57	CX58_CX58	CX59_CX59	CX60_CX60	CX61_CX61
TT	TT	TT	CC	CC	CC	CC	TT	TT
GG	GG	GG	GG	GG	GG	GG	GG	GG
AA	AA	AA	TT	TT	TT	TT	AT	AA
CC	CC	CC	CC	CC	CC	CC	CC	CC
GG	GG	GG	AA	AA	AA	AA	GG	GG
AA	AA	AA	GG	GG	GG	GG	AA	AA
GG	GG	GG	GG	GG	GG	GG	GG	GG
CC	CC	CC	TT	TT	TT	TT	CC	CC

ecaBatVro	UZROS59	aKoraMuaz	AlefAmbes	P1042	Caloro	Koshihikari	Colorado	IR64Sub1A
GG	GG	AA	AA	GG	GG	AA	AA	GG

CX63_CX63	CX64_CX64	CX65_CX65	CX66_CX66	CX67_CX67	CX68_CX68	CX69_CX69	CX70_CX70	CX71_CX71
TT	TT	TT	TT	TT	TT	TC	TT	TT
GG	GG	TT	TT	TT	GG	GG	GG	GG
AA	AA	AA	AA	AA	AA	AT	AA	NN
CC	CC	TT	TT	TT	CC	CC	CC	CC
GG	GG	GG	GG	GG	GG	GA	GG	GG
AA	AA	GG	GG	GG	AA	AA	AA	AA
GG	GG	GG	GG	GG	GG	GG	GG	GG
CC	CC	CC	CC	CC	CC	CT	CC	CC

Quilla66304	IR42	Fujisaka5	Suncheongbyeo	Cocodrie	Diamante	Antonio	P1048	HZROS637
AA	GG	AA	AA	AA	AA	AA	AA	GG

CX72_CX7	CX73_CX7	CX74_CX7	CX75_CX7	CX76_CX7	CX77_CX7	CX78_CX7	CX79_CX7	CX80_CX8
TT	TT	CC	TT	TT	CC	CC	TT	TT
GG	GG	GG	GG	GG	GG	GG	GG	GG
AA	AA	TT	AA	AA	TT	TT	AA	AA
CC	CC	CC	CC	CC	CC	CC	CC	CC
GG	GG	AA	GG	GG	AA	AA	GG	GG
AA	AA	GG	AA	AA	GG	GG	AA	AA
GG	NN	GG	GG	GG	GG	GG	GG	GG
CC	CC	TT	CC	CC	TT	TT	CC	CC

Affinis	asakstanic	Vavilovi	Nigrescens	Melanotri	AkTohum	Dicolorata	AzRos1646	kSalyMest
AA	GG	AA	AA	AA	AA	AA	GG	AA

CX82_CX8[illegible]	CX83_CX8[illegible]	CX84_CX8[illegible]	CX85_CX8[illegible]	CX86_CX8[illegible]	CX87_CX8[illegible]	CX88_CX8[illegible]	CX89_CX8[illegible]	CX90_CX9[illegible]
TT	TT	TT	TT	NN	TT	TT	TT	TT
GG	GG	GG	GG	GG	GG	GG	GG	GG
AA	AA	AA	AA	AA	AA	AA	AA	AA
CC	CC	CC	CC	CC	CC	CC	CC	CC
GG	GG	GG	GG	GG	GG	GG	GG	GG
AA	AA	AA	AA	NN	NN	AA	AA	AA
GG	GG	GG	GG	GG	GG	GG	GG	GG
CC	CC	CC	CC	CC	CC	CC	CC	CC

DONSKOJ2	DvRos0219	DvRos2568	Hokkajdo	liComboRe	kiShalaMe	Kesa	JBANETS5(	Mallai
AA	AA	AA	AA	GG	AA	GG	AA	AA

CX91_CX9	CX92_CX92	CX93_CX9	CX94_CX9	CX96_CX9	CX97_CX9	CX98_CX9	CX99_CX9	CX100_CX
TT	TT	TT	TT	TT	TT	TT	TT	TT
GG	GG	GG	GG	GG	GG	GG	GG	GG
AA	AA	AA	AA	AA	AA	AA	AA	AA
CC	CC	CC	CC	CC	CC	CC	CC	CC
GG	GG	GG	GG	GG	GG	GG	GG	GG
AA	AA	AA	AA	AA	AA	AA	AA	AA
GG	GG	GG	GG	GG	GG	GG	GG	GG
CC	CC	CC	CC	CC	CC	CC	CC	CC

Severnyj	JZBEKSKIJ	UzRos421	olotyjeVsho	Pioner320	rasnodarsk	UKTAKHAI	WIR623	Chernyj
AA	AA	AA	GG	AA	AA	GG	AA	AA

CX101_CX	CX102_CX	CX103_CX	CX104_CX	CX106_CX	CX107_CX	CX108_CX	CX109_CX	CX110_CX
TT	TT	TT	TC	CC	TT	TT	CC	TT
GG	GG	GG	TT	GG	GG	GG	GG	TT
AA	AA	AA	AA	TT	AA	AA	TT	AA
CC	CC	CC	CT	CC	CC	CC	CC	TT
GG	GG	GG	GG	AA	GG	GG	AA	GG
AA	AA	AA	GG	GG	AA	AA	GG	GG
GG	GG	GG	GG	GG	GG	GG	GG	GG
CC	CC	CC	CC	TT	CC	CC	TT	CC

WIR1528	HiMuke	WIR2623	Celiaj	JZROS275	WIR3764	Kendzo	KROS358	NF1
AA	AA	AA	AA	AA	AA	AA	AA	AA

CX111_CX	CX112_CX	CX113_CX	CX114_CX	CX115_CX	CX116_CX	CX117_CX	CX118_CX	CX119_CX
CC	TT	NN	TT	TT	CC	TT	TT	TT
GG	TT	GG	GG	GG	GG	GG	GG	GG
TT	AA	TT	AA	AA	TT	AA	AA	AA
CC	TT	CC	CC	CC	CC	CC	CC	CC
AA	GG	AA	GG	GG	NN	GG	GG	GG
GG	GG	GG	AA	AA	GG	AA	AA	AA
GG	GG	GG	GG	GG	GG	GG	GG	GG
TT	CC	TT	CC	CC	TT	CC	CC	CC

NF9	SPALCIK	M667	NTENSIVNY	EMCYZNY	WIR3419	DALRIS13	WIR2462	Shala
AA	AA	AA	GG	AA	AA	AA	AA	GG

CX120_CX:	CX121_CX:	CX122_CX:	CX123_CX:	CX124_CX:	CX125_CX:	CX126_CX:	CX128_CX:	CX129_CX:
TT	TT	TT	TT	TT	TT	TT	TT	CC
GG	GG	GG	GG	GG	GG	GG	GG	GG
AA	AA	AA	AA	AA	AA	AA	AA	TT
CC	CC	CC	CC	CC	CC	CC	CC	CC
GG	GG	GG	GG	GG	GG	GG	GG	AA
AA	AA	AA	AA	AA	AA	AA	NN	GG
GG	GG	GG	GG	GG	GG	GG	GG	GG
CC	CC	CC	CC	CC	CC	CC	CC	TT

Styk	Gidej	Bankoram	Saka	Mala	Sanganyan	Sakatiana	TOg5548	TOg6248
AA	AA	AG	AA	GG	GG	AA	GG	AA

CX130_CX:	CX131_CX:	CX132_CX:	CX133_CX:	CX134_CX:	CX138_CX:	CX139_CX:	CX140_CX:	CX141_CX:
TT	TT	CC	TT	TT	CC	CC	CC	TT
GG	GG	GG	GG	GG	GG	GG	GG	GG
AA	AA	TT	AA	AA	TT	TT	TT	AA
CC	CC	CC	CC	CC	CC	CC	CC	CC
GG	GG	AA	GG	GG	AA	AA	AA	GG
AA	AA	GG	AA	AA	GG	GG	GG	AA
GG	GG	GG	GG	GG	GG	GG	GG	GG
CC	CC	TT	CC	CC	TT	TT	TT	CC

TOg6264	TOg7201	\B450IBP2(	Presidio	Quinimpol	Stormproo	CreoleBred	erangSerar	SadriType
AA	AA	AA	AA	AA	AA	AA	AA	AA

CX142_CX:	CX143_CX:	CX144_CX:	CX145_CX:	CX146_CX:	CX147_CX:	CX148_CX:	CX149_CX:	CX150_CX:
CC	CC	TT	TT	TT	TT	TT	TT	TT
GG	GG	GG	GG	GG	GG	GG	TT	GG
TT	TT	AA	AA	AA	AA	AA	AA	AA
CC	CC	CC	CC	CC	CC	CC	TT	CC
AA	AA	GG	GG	GG	GG	GG	GG	GG
GG	GG	AA	AA	AA	AA	AA	GG	AA
GG	GG	GG	GG	GG	GG	GG	GG	GG
TT	NN	CC	CC	CC	CC	CC	CC	CC

arangSerar	SerangSel	olloChivacc	Juppa	Peta	Kamodi	PATNAI23	WC4443	Hassawi
AA	AG	AA	GG	GG	AA	AA	--	GG

CX151_CX:	CX152_CX:	CX153_CX:	CX154_CX:	CX155_CX:	CX156_CX:	CX157_CX:	CX158_CX:	CX160_CX:
CC	TC	TT	TT	TT	TT	TT	TT	TT
GG	GG	GG	GG	GG	GG	GG	GG	GG
TT	AA	AA	AA	AA	AA	AA	AA	AA
CC	CC	CC	CC	CC	CC	CC	CC	CC
AA	GG	GG	GG	GG	GG	GG	GG	GG
GG	AA	NN	AA	AA	AA	AA	AA	AA
GG	GG	NN	GG	GG	GG	GG	GG	GG
TT	CC	CC	CC	CC	CC	CC	CC	CC

Samanis	AshKataAus	ARC10638	ARC11524	ARC11611	Basmati	Achhame	endjahBant	WW82290
AA	GG	GG	AA	GG	GG	AA	GG	GG

CX161_CX	CX162_CX	CX165_CX	CX182_CX	CX205_CX	CX206_CX	CX207_CX	CX210_CX	CX211_CX
TT	TT	CC	TT	CC	TT	TT	TC	CC
GG	GG	GG	GG	GG	GG	GG	GG	GG
AA	AA	TT	AA	TT	AA	AA	AT	TT
CC	CC	CC	CC	CC	CC	CC	CC	CC
GG	GG	AA	GG	AA	GG	GG	NN	AA
AA	AA	GG	AA	GG	AA	AA	NN	GG
NN	GG	GG	GG	GG	GG	GG	GG	NN
CC	CC	TT	CC	TT	CC	CC	NN	TT

/ARYLAVA	aryVato27	galyMorinc	Jayanthi	Mushkan	Akabona	Aus8	Ngoba	IR64A
AA	GG	GG	GG	AA	GG	GG	GG	GG

CX212_CX	CX213_CX	CX214_CX	CX218_CX	CX219_CX	CX220_CX	CX221_CX	CX225_CX	CX226_CX
CC	CC	CC	TT	TT	CC	TT	TT	TT
GG	GG	GG	GG	GG	GG	GG	GG	GG
TT	TT	TT	AA	AA	TT	AA	AA	AA
CC	CC	CC	CC	CC	CC	CC	CC	CC
AA	AA	AA	GG	GG	AA	GG	GG	GG
GG	GG	GG	AA	AA	GG	AA	AA	AA
GG	GG	GG	GG	GG	GG	GG	GG	GG
TT	TT	TT	CC	CC	TT	CC	CC	CC

Karngi	Dudhel	Toga	sudiLongGr	Barah	Dehraduni	Qumanani	Shinali	P807
AA	AA	GG	AA	AA	AA	AA	AA	AA

CX227_CX2	CX228_CX2	CX230_CX2	CX231_CX2	CX232_CX2	CX233_CX2	CX234_CX2	CX235_CX2	CX236_CX2
TT	TT	TT	TT	TT	TT	TT	TT	TT
GG	GT	GG	GG	GG	GG	GG	GG	TT
AA	AA	AA	AA	AA	AA	AA	AA	AA
CC	TT	CC	CC	CC	CC	CC	CC	TT
GG	GG	GG	GG	GG	GG	GG	GG	GG
AA	GG	AA	AA	AA	AA	AA	AA	GG
GG	GG	GG	GG	GG	GG	GG	GG	GG
CC	CC	CC	CC	CC	CC	CC	CC	CC

JzBegohef2	ustSoclriMa	ownKros35	P1041	P1049	zerbaidjanic	WIR3412	TOg6281	TOg6288
GG	GG	GG	GG	AA	AA	GG	AA	AA

CX237_CX:	CX238_CX:	CX240_CX:	CX241_CX:	CX242_CX:	CX243_CX:	CX247_CX:	CX248_CX:	CX249_CX:
TT	TT	TT	CC	CC	TC	TT	CC	NN
GG	GG	GG	GG	GG	GG	GG	GG	GG
AA	AA	AA	TT	TT	NN	AA	TT	TT
CC	CC	CC	CC	CC	CC	CC	CC	CC
GG	GG	GG	AA	AA	NN	GG	AA	AA
AA	AA	AA	GG	GG	NN	NN	GG	GG
GG	GG	GG	GG	GG	GG	GG	GG	GG
CC	CC	CC	TT	TT	TT	CC	TT	TT

otyjeVshody	TOg6302	TOg6303	TOg6307	TOg6314	TOg6328	TOg6328B	TOg6335	TOg6342
GG	AA	AA	AA	AA	AA	AA	AA	AA

CX250_CX:	CX251_CX:	CX262_CX:	CX263_CX:	CX265_CX:	CX266_CX:	CX267_CX:	CX268_CX:	CX269_CX:
TT	CC	CC	TT	TT	CC	TT	TT	CC
GG	GG	GG	GG	GG	GG	GG	GG	GG
AA	TT	TT	AA	AA	TT	AA	AA	NN
CC	CC	CC	CC	CC	CC	CC	CC	CC
GG	AA	AA	GG	GG	AA	GG	GG	AA
AA	GG	GG	AA	AA	NN	AA	AA	GG
GG	GG	GG	GG	GG	GG	GG	GG	GG
CC	TT	TT	CC	CT	TT	CC	CC	TT

TOg6367	TOg6392	TOg6405	TOg6422	TOg6464	TOg6465	TOg6468	TOg6474	TOg6511
AA	AA	AA	AA	AA	AA	AA	AA	AA

CX270_CX	CX273_CX	CX274_CX	CX275_CX	CX276_CX	CX277_CX	CX278_CX	CX280_CX	CX281_CX
TT	TT	TT	TT	TT	CC	TT	TC	TT
GG	GG	GG	GG	GG	GG	GT	GG	GG
AA	AA	AA	AA	AA	TT	AA	AT	AA
CC	CC	CC	CC	CC	CC	NN	CC	CC
GG	GG	GG	GG	GG	AA	GG	GA	GG
AG	AA	AA	AA	AA	GG	AG	NN	AA
GG	GG	GG	GG	GG	GG	GG	GG	GG
CC	CC	CC	CC	CC	TT	CC	NN	CC

TOg6512	TOg6916	TOg6943	TOg6946	TOg6951	TOg7194	TOg7199	CG14	B450IBP23
AA	AA	AA	AA	AA	GG	GG	AA	AA

CX282_CX2	CX284_CX2	CX285_CX2	CX286_CX2	CX287_CX2	CX288_CX2	CX290_CX2	CX291_CX2	CX296_CX2
CC	NN	CC	CC	CC	TT	TT	TT	TT
GG	GG	GG	GG	GG	GG	GG	GG	GG
NN	NN	TT	TT	TT	AA	AA	AA	AA
CC	CC	CC	CC	CC	CC	CC	CC	CC
NN	AA	AA	AA	AA	GG	GG	GG	GG
GG	GG	GG	GG	GG	NN	AA	AA	AA
GG	GG	GG	GG	GG	GG	GG	GG	GG
NN	NN	TT	TT	TT	CC	CC	CC	CC

Pokkali	Simpor	PutihMonto	BuluPote	Daudzai	Pakkali	Kirak	I363	gaPaukhiDh
GG	AA	AA	AA	GG	AA	GG	GG	GG

CX303_CX3	CX304_CX3	CX305_CX3	CX306_CX3	CX307_CX3	CX313_CX3	CX314_CX3	CX315_CX3	CX316_CX3
TT	TT	TT	CC	CC	TT	TT	CC	CC
GG	GG	GG	GG	GG	GG	GG	GG	GG
AA	AA	AA	TT	TT	AA	AA	TT	TT
CC	CC	CC	CC	CC	CC	CC	CC	CC
GG	GG	GG	AA	AA	GG	GG	AA	NN
AA	AA	AA	GG	GG	AG	AA	GG	GG
GG	GG	GG	GG	GG	GG	GG	GG	GG
CC	CC	CC	TT	TT	CC	CC	TT	TT

Latisail	PadiKasalle	HAHORA14	KaloMarsi	TOg6244	TOg6249	TOg6250	TOg6251	TOg6253
GG	AA	AA	AA	AA	AA	AA	AA	AA

CX317_CX3	CX318_CX3	CX319_CX3	CX328_CX3	CX329_CX3	CX330_CX3	CX340_CX3	CX341_CX3	CX342_CX3
CC	TT	TT	TT	CC	CC	TT	TT	TT
GG	GG	GG	GG	GG	GG	GG	GG	GG
TT	AT	AA	AA	TT	TT	AA	AA	AA
CC	CC	CC	CC	CC	CC	CC	CC	CC
AA	GG	GG	GG	AA	AA	GG	GG	GG
GG	AA	AA	AA	GG	GG	AA	AA	AA
GG	GG	GG	GG	GG	GG	GG	GG	GG
TT	CC	CC	CC	TT	TT	CC	CC	CC

TOg6259	TOg6266	TOg6271	TOg7174	RU1303138	RU0803147	RU1303153	RU0803153	RU1003123
AA	AA	AA	AA	GG	AA	GG	AA	AA

CX343_CX3	CX344_CX3	CX345_CX3	CX346_CX3	CX347_CX3	CX348_CX3	CX349_CX3	CX350_CX3	CX351_CX3
TT	CC	CC	TT	TC	TT	TT	CC	CC
GG	GG	GG	GG	GG	GG	GG	GG	GG
AA	TT	TT	AA	AA	AA	AA	TT	TT
CC	CC	CC	CC	CC	CC	CC	CC	CC
GG	AA	AA	GG	GG	GG	GG	AA	AA
AA	GG	GG	AA	AA	AA	AA	GG	GG
GG	GG	GG	GG	GG	GG	GG	GG	GG
CC	TT	TT	CC	CC	CC	CC	TT	TT

RU1503175	RU1503147	RU1603138	RU1403141	RU1603144	RU1603178	RU1603113	RU1003098	RU1303181
AA	AA	AA	AA	AA	AA	AA	AA	AA

CX352_CX3	CX353_CX3	CX354_CX3	CX355_CX3	CX356_CX3	CX357_CX3	CX358_CX3	CX359_CX3	CX360_CX3
CC	CC	CC	CC	CC	TT	TT	CC	TT
GG	GG	GG	GG	GG	GG	GG	GG	GG
TT	TT	TT	TT	TT	AA	AA	TT	AA
CC	CC	CC	CC	CC	CC	CC	CC	CC
AA	AA	AA	AA	AA	GG	GG	AA	GG
GG	GG	GG	GG	GG	AA	AA	GG	AA
GG	GG	GG	GG	GG	GG	GG	GG	GG
TT	TT	TT	TT	TT	CC	CC	TT	CC

RU1403089	RU1403138	RU1403153	RU1503169	RU1603086	RU1603089	RU1603116	RU1603123	CL111
AA	AA	AA	AA	AA	AA	AA	AA	AA

CX361_CX	CX362_CX	CX363_CX	CX364_CX	CX365_CX	CX366_CX	CX367_CX	CX368_CX	CX369_CX
TT	TT	TT	TT	TC	TT	NN	TT	TT
GG	GG	GG	GG	GG	GG	NN	TT	NN
AA	AA	AA	AA	NN	AA	NN	AA	AA
CC	CC	CC	CC	CC	CC	NN	TT	CC
GG	GG	GG	GG	NN	GG	GA	GG	GG
AA	AA	AA	AA	GG	AG	GG	GG	AA
GG	GG	GG	GG	GG	GG	GG	GG	GG
CC	CC	CC	CC	CT	CC	NN	CC	CC

CL153	PRESIDIO	IERMENTA	JUPITER	WELLS	LAKAST	DIAMOND	MM14	REX
AA	AA	AA	AA	AA	AA	AA	AA	AA

CX370_CX:	CX371_CX:	CX372_CX:	CX373_CX:	CX374_CX:	CX375_CX:	CX376_CX:	CX377_CX:	CX378_CX:
TT	CC	CC	CC	CC	TT	TT	TT	TT
GG	GG	GG	GG	GG	GG	GG	GG	GG
AA	NN	TT	TT	TT	AA	AA	AA	AA
CC	CC	CC	CC	CC	CC	CC	CC	CC
GG	AA	AA	AA	AA	GG	GG	GG	GG
NN	GG	GG	GG	GG	AA	NN	AA	NN
GG	GG	GG	GG	GG	GG	NN	GG	GG
CC	TT	TT	TT	TT	CC	CC	CC	CC

CHENIERE	COCODRIE	CL272	ROYJ	TITAN	JAZZMAN2	CL172	M206	CL163
AA	AA	AA	AA	AA	AA	AA	AA	AA

CX379_CX	CX380_CX	CX381_CX	CX382_CX	CX383_CX	CX384_CX	CX385_CX	CX386_CX	CX387_CX
TT	CC	TT	TT	CC	CC	TT	TT	TT
GG	GG	GG	GG	GG	GG	GG	GG	GG
AA	TT	AA	AA	TT	TT	AA	AA	AA
CC	CC	CC	CC	CC	CC	CC	CC	CC
GG	AA	GG	GG	AA	AA	GG	GG	GG
AA	GG	AA	AA	GG	GG	AA	AA	AA
GG	GG	GG	NN	GG	GG	GG	GG	GG
CC	TT	CC	CC	TT	TT	CC	CC	CC

DELLA2	ANTONIO	THAD	RONDO	CL151	IR64Sub1	Og6342re	IR64IRRI	erangSub1A
AA	AA	AA	GG	AA	GG	AA	GG	GG

CX388_CX3	CX389_CX3	CX390_CX3	CX391_CX3	CX392_CX3	CX393_CX3	CX394_CX3	CX395_CX3	CX396_CX3
TT	CC	TT	CC	TT	TT	TT	TC	CC
TT	GG	GG	GG	GG	GG	GG	GG	GG
AA	NN	AA	TT	AA	AA	AA	TT	TT
TT	CC	CC	CC	CC	CC	CC	CC	CC
GG	AA	GA	AA	GG	GG	GG	GA	AA
GG	GG	AA	GG	AA	AA	AA	NN	GG
GG	GG	GG	GG	GG	GG	GG	GG	GG
CC	TT	CC	TT	CC	CC	CC	CT	TT

iherangSub	Ciherang	IR64AG1	Darij8	64Beaumo	1670036R	1670036R	1670036R	1670036R
GG	GG	GG	AA	GG	GG	GG	GG	GG

CX397_CX3	CX399_CX3	CX400_CX4	CX401_CX4	CX402_CX4	CX403_CX4	CX408_CX4	CX409_CX4	CX410_CX4
CC	TT	CC	TC	CC	TT	TT	TT	TT
GG	GG	GG	GG	GG	GG	GG	GG	GG
TT	AA	AT	AA	AA	AA	AA	AA	AA
CC	CC	CC	CC	CC	CC	CC	CC	CC
AA	GG	GA	GG	GG	GG	GG	GG	GG
GG	AA	AG	AG	GG	AA	AA	AA	AA
GG	GG	GG	GG	GG	GG	GG	GG	GG
TT	CC	CT	CC	CC	CC	CC	CC	CC

31670036R	31670036R	31670036R	31670036R	31670036R	31670036R	31670036R	31670036R	31670036R
GG	GG	GG	GG	GG	GG	GG	GG	GG

CX411_CX4	CX413_CX4	CX414_CX4	CX416_CX4	CX417_CX4	CX418_CX4	CX419_CX4	CX420_CX4	CX421_CX4
TT	TT	TT	TT	TT	TT	TT	TT	TT
GG	GG	GG	GG	GG	GG	GG	GG	GG
AA	AA	AA	AA	AA	AA	AA	AA	AA
CC	CC	CC	CC	CC	CC	CC	CC	CC
GG	GG	GG	GG	GG	GG	GG	GG	GG
AA	AA	AA	AA	AA	AA	AA	AA	AA
GG	GG	GG	GG	GG	GG	GG	GG	GG
CC	CC	CC	CC	CC	CC	CC	CC	CC

31670036R	31670036R	31670036R	31670036R	31670036R	31670036R	31670036R	31670036R	31670036R
GG	GG	GG	GG	GG	GG	GG	GG	GG

CX422_CX4	CX423_CX4	CX424_CX4	CX425_CX4	CX426_CX4	CX427_CX4	CX428_CX4	CX429_CX4	CX431_CX4
TT	TT	TT	TT	TT	CC	CC	CC	TT
GG	GG	GG	GG	GG	GG	GG	GG	GG
AA	AA	AA	AA	AA	TT	TT	TT	AA
CC	CC	CC	CC	CC	CC	CC	CC	CC
GG	GG	GG	GG	GG	AA	AA	AA	GG
AA	AA	AA	AA	AA	GG	GG	GG	AA
GG	GG	GG	GG	GG	GG	GG	GG	GG
CC	CC	CC	CC	CC	TT	TT	TT	CC

31670036R12C01
GG

CX534_CX5	CX542_CX5	CX548_CX5	CX561_CX5	CX578_CX5	CX579_CX5	IRIS_313-7	IRIS_313-7	IRIS_313-7
CC	TT	TT	TT	CC	CC	TT	TT	TT
GG	GT	GG	GG	GG	GG	GG	GG	GG
TT	AA	AA	AA	TT	TT	AA	AA	AA
CC	CT	CC	CC	CC	CC	CC	CC	CC
AA	GG	GG	GG	AA	AA	GG	GG	GG
GG	AG	AA	AA	GG	GG	AA	AA	AA
GG	GG	GG	GG	GG	GG	GG	GG	GG
TT	CC	CC	CC	TT	TT	NN	CC	CC

IRIS_313-7	IRIS_313-7	IRIS_313-7	IRIS_313-7	IRIS_313-7	IRIS_313-7	IRIS_313-7	IRIS_313-7	IRIS_313-7
TT	TT	TT	TT	TT	TT	TT	TT	TT
GG	GG	GG	GG	GG	GG	GG	GG	GG
AA	AA	AA	AA	AA	AA	AA	AA	AA
CC	CC	CC	CC	CC	CC	CC	CC	CC
GG	GG	GG	GG	GG	NN	GG	GG	GG
AA	AA	AA	AA	AA	AA	AA	AA	AA
GG	GG	GG	GG	GG	GG	GG	GG	GG
CC	CC	CC	CC	CC	CC	CC	CC	CC

IRIS_313-7	IRIS_313-7	IRIS_313-7	IRIS_313-7	IRIS_313-7	IRIS_313-7	IRIS_313-7	IRIS_313-7	IRIS_313-7
TT	TT	TT	TT	TT	TT	TT	TT	TT
GG	GG	GG	GG	GG	GG	GG	GG	GG
AA	AA	AA	AA	AA	AA	AA	AA	AA
CC	CC	CC	CC	CC	CC	CC	CC	CC
GG	GG	GG	GG	GG	GG	GG	GG	GG
AA	AA	AA	AA	AA	AA	AA	NN	NN
GG	GG	GG	GG	GG	GG	GG	GG	GG
CC	CC	CC	CC	CC	CC	CC	CC	CC

IRIS_313-7	IRIS_313-7	IRIS_313-7	IRIS_313-7	IRIS_313-7	IRIS_313-7	IRIS_313-7	IRIS_313-7	IRIS_313-7
TT	TT	TT	TT	TT	TT	TT	TT	TT
GG	GG	GG	GG	GG	GG	GG	GG	GG
AA	AA	AA	AA	AA	AA	AA	AA	AA
CC	CC	CC	CC	CC	CC	CC	CC	CC
GG	GG	GG	GG	GG	GG	GG	GG	GG
AA	AA	AA	AA	AA	NN	AA	AA	AA
GG	GG	GG	GG	GG	NN	GG	GG	GG
CC	CC	CC	CC	CC	CC	CC	CC	CC

IRIS_313-7	IRIS_313-7	IRIS_313-7	IRIS_313-7	IRIS_313-7	IRIS_313-7	IRIS_313-7	IRIS_313-7	IRIS_313-7
TT	TT	TT	TT	TT	TT	TT	TT	TT
GG	GG	GG	GG	GG	GG	GG	GG	GG
AA	AA	AA	AA	AA	AA	AA	AA	AA
CC	CC	CC	CC	CC	CC	CC	CC	CC
GG	GG	GG	GG	GG	GG	GG	GG	GG
AA	NN	AA	AA	AA	AA	AA	AA	AA
GG	GG	GG	GG	GG	GG	GG	GG	GG
CC	CC	CC	CC	CC	CC	CC	CC	CC

IRIS_313-7	IRIS_313-7	IRIS_313-7	IRIS_313-7	IRIS_313-7	IRIS_313-7	IRIS_313-7	IRIS_313-7	IRIS_313-7
TT	TT	TT	TT	TT	TT	TT	TT	TT
GG	GG	GG	GG	GG	GG	GG	GG	GG
AA	AA	AA	AA	AA	AA	AA	AA	AA
CC	CC	CC	CC	CC	CC	CC	CC	CC
GG	GG	GG	GG	GG	GG	GG	GG	GG
AA	AA	AA	AA	AA	AA	AA	AA	AA
GG	GG	GG	GG	GG	GG	GG	GG	GG
CC	CC	CC	CC	CC	CC	CC	CC	CC

IRIS_313-7	IRIS_313-7	IRIS_313-7	IRIS_313-7	IRIS_313-7	IRIS_313-7	IRIS_313-7	IRIS_313-7	IRIS_313-7
TT	TT	CC	CC	CC	CC	CC	CC	CC
GG	GG	GG	GG	GG	GG	GG	GG	GG
AA	AA	TT	TT	TT	TT	TT	TT	TT
CC	CC	CC	CC	CC	CC	CC	CC	CC
GG	GG	AA	AA	AA	AA	AA	AA	AA
AA	AA	GG	GG	GG	GG	GG	GG	GG
GG	GG	GG	GG	GG	GG	GG	GG	GG
CC	CC	TT	TT	TT	TT	TT	TT	TT

IRIS_313-7	IRIS_313-7	IRIS_313-7	IRIS_313-7	IRIS_313-7	IRIS_313-7	IRIS_313-7	IRIS_313-7	IRIS_313-7
CC	CC	CC	TT	CC	CC	CC	TT	CC
GG	GG	GG	GG	GG	GG	GG	GG	GG
TT	TT	TT	AA	TT	TT	TT	AA	TT
CC	CC	CC	CC	CC	CC	CC	CC	CC
AA	AA	AA	GG	AA	AA	AA	GG	AA
GG	GG	GG	AA	GG	GG	GG	AA	GG
GG	GG	GG	GG	GG	GG	GG	GG	GG
TT	TT	TT	CC	TT	TT	TT	CC	TT

IRIS_313-7	IRIS_313-7	IRIS_313-7	IRIS_313-7	IRIS_313-7	IRIS_313-7	IRIS_313-7	IRIS_313-7	IRIS_313-8
CC	CC	CC	CC	CC	CC	CC	CC	CC
GG	GG	GG	GG	GG	GG	GG	GG	GG
TT	TT	TT	TT	TT	TT	TT	TT	TT
CC	CC	CC	CC	CC	CC	CC	CC	CC
AA	AA	AA	AA	AA	AA	AA	AA	AA
GG	GG	GG	GG	GG	GG	GG	GG	GG
GG	GG	GG	GG	GG	GG	GG	GG	GG
TT	TT	TT	TT	TT	TT	TT	TT	TT

IRIS_313-ᶓ	IRIS_313-ᶓ	IRIS_313-ᶓ	IRIS_313-ᶓ	IRIS_313-ᶓ	IRIS_313-ᶓ	IRIS_313-ᶓ	IRIS_313-ᶓ	IRIS_313-ᶓ
CC	CC	CC	CC	CC	CC	CC	CC	CC
GG	GG	GG	GG	GG	GG	GG	GG	GG
TT	TT	TT	TT	TT	TT	TT	TT	TT
CC	CC	CC	CC	CC	CC	CC	CC	CC
AA	AA	AA	AA	AA	AA	AA	AA	AA
GG	GG	GG	GG	GG	GG	GG	GG	GG
GG	GG	GG	GG	GG	GG	GG	GG	GG
TT	TT	TT	TT	TT	TT	TT	TT	TT

IRIS_313-8	IRIS_313-8	IRIS_313-8	IRIS_313-8	IRIS_313-8	IRIS_313-8	IRIS_313-8	IRIS_313-8	IRIS_313-8
CC	CC	CC	CC	CC	CC	CC	CC	CC
GG	GG	GG	GG	GG	GG	GG	GG	GG
TT	TT	TT	TT	TT	TT	TT	TT	TT
CC	CC	CC	CC	CC	CC	CC	CC	CC
AA	AA	AA	AA	AA	AA	AA	AA	AA
GG	GG	GG	GG	GG	GG	GG	GG	GG
GG	GG	GG	GG	GG	GG	GG	GG	GG
TT	TT	TT	TT	TT	TT	TT	TT	TT

IRIS_313-8	IRIS_313-8	IRIS_313-8	IRIS_313-8	IRIS_313-8	IRIS_313-8	IRIS_313-8	IRIS_313-8	IRIS_313-8
CC	CC	CC	CC	CC	CC	CC	CC	CC
GG	GG	GG	GG	GG	GG	GG	GG	GG
TT	TT	TT	TT	TT	TT	TT	TT	TT
CC	CC	CC	CC	CC	CC	CC	CC	CC
AA	AA	AA	AA	AA	AA	AA	AA	AA
GG	GG	GG	GG	GG	GG	GG	GG	GG
GG	GG	GG	GG	GG	GG	GG	GG	GG
TT	TT	TT	TT	TT	TT	TT	TT	TT

IRIS_313-8	IRIS_313-8	IRIS_313-8	IRIS_313-8	IRIS_313-8	IRIS_313-8	IRIS_313-8	IRIS_313-8	IRIS_313-8
CC	CC	CC	TC	TT	TT	CC	TT	CC
GG	GG	GG	GG	GG	GG	GG	GG	GG
TT	TT	TT	AT	AA	AA	TT	AA	TT
CC	CC	CC	CC	CC	CC	CC	CC	CC
AA	AA	AA	NN	GG	GG	AA	GG	AA
GG	GG	GG	NN	AA	AA	GG	AA	GG
GG	GG	GG	GG	GG	GG	GG	GG	GG
TT	TT	NN	CC	CC	CC	TT	CC	TT

IRIS_313-8	IRIS_313-8	IRIS_313-8	IRIS_313-8	IRIS_313-8	IRIS_313-8	IRIS_313-8	IRIS_313-8	IRIS_313-8
CC	CC	CC	CC	CC	CC	CC	CC	CC
GG	GG	GG	GG	GG	GG	GG	GG	GG
TT	TT	TT	TT	TT	TT	TT	TT	TT
CC	CC	CC	CC	CC	CC	CC	CC	CC
AA	AA	AA	AA	AA	AA	AA	AA	AA
GG	GG	GG	GG	GG	GG	GG	GG	GG
GG	GG	GG	GG	GG	GG	GG	GG	GG
TT	TT	TT	TT	TT	TT	TT	TT	TT

IRIS_313-8	IRIS_313-8	IRIS_313-8	IRIS_313-8	IRIS_313-8	IRIS_313-8	IRIS_313-8	IRIS_313-8	IRIS_313-8
CC	CC	CC	CC	CC	CC	CC	CC	CC
GG	GG	GG	GG	GG	GG	GG	GG	GG
TT	TT	TT	TT	TT	TT	TT	TT	TT
CC	CC	CC	CC	CC	CC	CC	CC	CC
AA	AA	AA	AA	AA	AA	AA	AA	AA
GG	GG	GG	GG	GG	GG	GG	GG	GG
GG	GG	GG	GG	GG	GG	GG	GG	GG
TT	TT	TT	TT	TT	TT	TT	TT	TT

IRIS_313-8	IRIS_313-8	IRIS_313-8	IRIS_313-8	IRIS_313-8	IRIS_313-8	IRIS_313-8	IRIS_313-8	IRIS_313-8
CC	CC	CC	CC	CC	CC	CC	CC	CC
GG	GG	GG	GG	GG	GG	GG	GG	GG
TT	TT	TT	TT	TT	TT	TT	TT	TT
CC	CC	CC	CC	CC	CC	CC	CC	CC
AA	AA	AA	AA	AA	AA	AA	AA	AA
GG	GG	GG	GG	GG	GG	GG	GG	GG
GG	GG	GG	GG	GG	NN	GG	GG	GG
TT	TT	TT	TT	TT	TT	TT	TT	TT

IRIS_313-8	IRIS_313-8	IRIS_313-8	IRIS_313-8	IRIS_313-8	IRIS_313-8	IRIS_313-8	IRIS_313-8	IRIS_313-8
CC	CC	CC	CC	CC	CC	CC	CC	CC
GG	GG	GG	GG	GG	GG	GG	GG	GG
TT	TT	TT	TT	TT	TT	TT	TT	TT
CC	CC	CC	CC	CC	CC	CC	CC	CC
AA	AA	AA	AA	AA	AA	AA	AA	AA
GG	GG	GG	GG	GG	GG	GG	GG	GG
GG	GG	GG	GG	GG	GG	GG	GG	GG
TT	TT	TT	TT	TT	TT	TT	TT	TT

IRIS_313-[illegible]	IRIS_313-[illegible]	IRIS_313-[illegible]	IRIS_313-[illegible]	IRIS_313-[illegible]	IRIS_313-[illegible]	IRIS_313-[illegible]	IRIS_313-[illegible]	IRIS_313-[illegible]
CC	CC	CC	CC	CC	CC	CC	CC	CC
GG	GG	GG	GG	GG	GG	GG	GG	GG
TT	TT	TT	TT	TT	TT	TT	TT	TT
CC	CC	CC	CC	CC	CC	CC	CC	CC
AA	AA	AA	AA	AA	AA	AA	AA	AA
GG	GG	GG	GG	GG	GG	GG	GG	GG
GG	GG	GG	GG	GG	GG	GG	GG	GG
TT	TT	TT	TT	TT	TT	TT	TT	TT

IRIS_313-8	IRIS_313-8	IRIS_313-8	IRIS_313-8	IRIS_313-8	IRIS_313-8	IRIS_313-8	IRIS_313-8	IRIS_313-8
CC	CC	CC	CC	CC	TT	CC	CC	CC
GG	GG	GG	GG	GG	GG	GG	GG	GG
TT	TT	TT	TT	TT	AA	TT	TT	TT
CC	CC	CC	CC	CC	CC	CC	CC	CC
AA	AA	AA	AA	AA	GG	AA	AA	AA
GG	GG	GG	GG	GG	AA	GG	GG	GG
GG	GG	GG	GG	GG	GG	GG	GG	GG
TT	TT	TT	TT	TT	CC	TT	TT	TT

IRIS_313-8	IRIS_313-8	IRIS_313-8	IRIS_313-8	IRIS_313-8	IRIS_313-8	IRIS_313-8	IRIS_313-8	IRIS_313-8
CC	CC	CC	CC	CC	CC	CC	CC	CC
GG	GG	GG	GG	GG	GG	GG	GG	GG
TT	TT	TT	TT	TT	TT	TT	TT	TT
CC	CC	CC	CC	CC	CC	CC	CC	CC
AA	AA	AA	AA	AA	AA	AA	AA	AA
GG	GG	GG	GG	GG	GG	GG	GG	GG
GG	GG	GG	GG	GG	GG	GG	GG	GG
TT	TT	TT	TT	TT	TT	TT	TT	TT

IRIS_313-8	IRIS_313-8	IRIS_313-8	IRIS_313-8	IRIS_313-8	IRIS_313-8	IRIS_313-8	IRIS_313-8	IRIS_313-8
CC	TT	CC	CC	CC	CC	CC	CC	CC
GG	GG	GG	GG	GG	GG	GG	GG	GG
TT	AA	TT	TT	TT	TT	TT	TT	TT
CC	CC	CC	CC	CC	CC	CC	CC	CC
AA	GG	AA	AA	AA	AA	AA	AA	AA
GG	AA	GG	GG	GG	GG	GG	GG	GG
GG	GG	GG	GG	GG	GG	GG	GG	GG
TT	CC	TT	TT	TT	TT	TT	TT	TT

IRIS_313-8	IRIS_313-8	IRIS_313-8	IRIS_313-8	IRIS_313-8	IRIS_313-8	IRIS_313-8	IRIS_313-8	IRIS_313-8
CC	CC	CC	CC	TT	CC	CC	TT	CC
GG	GG	GG	GG	GG	GG	GG	GG	GG
TT	TT	TT	TT	AA	TT	TT	AA	TT
CC	CC	CC	CC	CC	CC	CC	CC	CC
AA	AA	AA	AA	GG	AA	AA	GG	AA
GG	GG	GG	GG	AA	GG	GG	NN	GG
GG	GG	GG	GG	GG	GG	GG	GG	GG
TT	TT	TT	TT	CC	TT	TT	CC	TT

IRIS_313-8	IRIS_313-8	IRIS_313-8	IRIS_313-8	IRIS_313-8	IRIS_313-8	IRIS_313-8	IRIS_313-8	IRIS_313-8
CC	CC	CC	TT	TT	TT	CC	TT	CC
GG	GG	GG	GG	TT	GG	GG	GG	GG
TT	TT	TT	AA	AA	AA	TT	AA	TT
CC	CC	CC	CC	TT	CC	CC	CC	CC
AA	AA	AA	GG	GG	GG	AA	GG	AA
GG	GG	GG	AA	GG	AA	GG	AA	GG
GG	NN	GG	GG	GG	GG	GG	GG	GG
TT	TT	TT	CC	CC	CC	TT	CC	TT

IRIS_313-8	IRIS_313-8	IRIS_313-8	IRIS_313-8	IRIS_313-8	IRIS_313-8	IRIS_313-8	IRIS_313-8	IRIS_313-8
CC	CC	TT	CC	TT	TT	TT	TT	TC
GG	GG	TT	GG	GG	GG	GG	GG	GT
TT	TT	AA	TT	AA	AA	AA	AA	AT
CC	CC	TT	CC	CC	CC	CC	CC	CT
AA	AA	GG	AA	GG	GG	GG	GG	GA
GG	GG	GG	GG	AA	AA	AA	AA	GG
GG	GG	GG	GG	GG	GG	GG	GG	GG
TT	TT	CC	TT	CC	CC	CC	CC	CT

IRIS_313-8	IRIS_313-8	IRIS_313-8	IRIS_313-8	IRIS_313-8	IRIS_313-8	IRIS_313-8	IRIS_313-8	IRIS_313-8
TT	TT	TT	TT	CC	TT	TT	TT	CC
GG	TT	GG	GG	GG	GG	GG	TT	GG
AA	AA	AA	AA	TT	AA	AA	AA	TT
CC	TT	CC	CC	CC	CC	CC	TT	CC
GG	GG	GG	GG	AA	GG	GG	GG	AA
AA	GG	AA	AA	GG	AA	AA	GG	GG
GG	GG	GG	GG	GG	GG	GG	GG	GG
NN	CC	CC	CC	TT	CC	CC	CC	TT

IRIS_313-8	IRIS_313-8	IRIS_313-8	IRIS_313-8	IRIS_313-8	IRIS_313-8	IRIS_313-8	IRIS_313-8	IRIS_313-8
TT	CC	TT	CC	TT	TT	TT	CC	TT
GG	GG	GG	GG	GG	GG	GG	GG	GG
AA	TT	AA	TT	AA	AA	AA	TT	AA
CC	CC	CC	CC	CC	CC	CC	CC	CC
GG	AA	GG	AA	GG	GG	GG	AA	GG
AA	GG	AA	GG	AA	AA	AA	GG	AA
GG	GG	GG	GG	GG	GG	GG	GG	GG
CC	TT	CC	TT	CC	CC	CC	TT	CC

IRIS_313-8	IRIS_313-8	IRIS_313-8	IRIS_313-8	IRIS_313-8	IRIS_313-8	IRIS_313-8	IRIS_313-8	IRIS_313-8
TT	CC	TT	TT	CC	TT	CC	TT	TT
GG	GG	GG	GG	GG	GG	GG	GG	GG
AA	TT	AA	AA	TT	AA	TT	AA	AA
CC	CC	CC	CC	CC	CC	CC	CC	CC
GG	AA	GG	GG	AA	GG	AA	GG	GG
AA	GG	AA	NN	GG	AA	GG	AA	AA
GG	GG	GG	NN	GG	GG	GG	GG	GG
CC	TT	CC	CC	TT	CC	TT	CC	CC

IRIS_313-8	IRIS_313-8	IRIS_313-8	IRIS_313-8	IRIS_313-8	IRIS_313-8	IRIS_313-8	IRIS_313-8	IRIS_313-8
TT	TT	CC	CC	TT	TT	TT	TT	TT
GG	TT	GG	GG	GG	GG	GG	GG	GG
AA	AA	TT	TT	AA	AA	AA	AA	AA
CC	TT	CC	CC	CC	CC	CC	CC	CC
GG	GG	AA	AA	GG	GG	GG	GG	GG
AA	GG	GG	GG	AA	AA	AA	NN	AA
GG	GG	GG	GG	GG	GG	GG	NN	AA
CC	CC	TT	TT	NN	CC	CC	CC	CC

IRIS_313-8	IRIS_313-8	IRIS_313-8	IRIS_313-8	IRIS_313-8	IRIS_313-8	IRIS_313-8	IRIS_313-8	IRIS_313-8
TT	TT	CC	TT	CC	TT	CC	TT	CC
GG	GG	GG	GG	GG	GG	GG	GG	GG
AA	AA	TT	AA	TT	AA	TT	AA	TT
CC	CC	CC	CC	CC	CC	CC	CC	CC
GG	GG	AA	GG	AA	GG	AA	GG	AA
AA	AA	GG	AA	GG	AA	GG	AA	GG
GG	GG	GG	GG	GG	GG	GG	GG	GG
NN	CC	TT	CC	TT	CC	TT	CC	TT

IRIS_313-8	IRIS_313-8	IRIS_313-8	IRIS_313-8	IRIS_313-8	IRIS_313-8	IRIS_313-8	IRIS_313-8	IRIS_313-8
CC	TT	TT	TT	NN	TT	TT	TT	TT
GG	GG	GG	GG	GG	GG	GG	GG	GG
TT	AA	AA	AA	AA	AA	AA	AA	AA
CC	CC	CC	CC	CC	CC	CC	CC	CC
AA	GG	GG	GG	GG	GG	GG	GG	GG
GG	AA	AA	AA	AA	NN	AA	AA	AA
GG	GG	GG	GG	GG	GG	GG	GG	GG
TT	CC	CC	CC	CC	CC	CC	CC	CC

IRIS_313-8	IRIS_313-8	IRIS_313-8	IRIS_313-8	IRIS_313-8	IRIS_313-8	IRIS_313-8	IRIS_313-8	IRIS_313-8
CC	TT	CC	TT	TT	CC	CC	TT	TT
GG	GG	GG	GG	GG	GG	GG	GG	TT
TT	AA	TT	AA	AA	TT	TT	AA	AA
CC	CC	CC	CC	CC	CC	CC	CC	TT
AA	GG	AA	GG	GG	AA	AA	GG	GG
GG	AA	GG	AA	AA	GG	GG	AA	NN
GG	GG	GG	GG	GG	NN	GG	GG	NN
TT	CC	TT	CC	CC	NN	TT	CC	CC

IRIS_313-8	IRIS_313-8	IRIS_313-8	IRIS_313-8	IRIS_313-8	IRIS_313-8	IRIS_313-8	IRIS_313-8	IRIS_313-8
CC	CC	TT	TT	TT	TT	TT	CC	TT
GG	GG	GG	GG	TT	GG	GG	GG	GG
TT	TT	AA	AA	AA	AA	AA	TT	AA
CC	CC	CC	CC	TT	CC	CC	CC	CC
AA	AA	GG	GG	GG	GG	GG	AA	GG
GG	GG	AA	AA	GG	AA	AA	GG	AA
GG	GG	GG	GG	GG	GG	GG	GG	GG
TT	TT	CC	CC	CC	CC	CC	TT	CC

IRIS_313-8	IRIS_313-8	IRIS_313-8	IRIS_313-8	IRIS_313-8	IRIS_313-8	IRIS_313-8	IRIS_313-8	IRIS_313-8
TT	TT	CC	CC	CC	TT	TT	TT	CC
TT	GG	GG	GG	GG	GG	GG	GG	GG
AA	AA	TT	TT	TT	AA	AA	AA	TT
TT	CC	CC	CC	CC	CC	CC	CC	CC
GG	GG	AA	AA	AA	GG	GG	GG	AA
GG	AA	GG	GG	GG	AA	AA	AA	GG
GG	GG	GG	GG	GG	GG	GG	GG	GG
CC	CC	TT	TT	TT	CC	CC	CC	TT

IRIS_313-[illegible]	IRIS_313-[illegible]	IRIS_313-[illegible]	IRIS_313-[illegible]	IRIS_313-[illegible]	IRIS_313-[illegible]	IRIS_313-[illegible]	IRIS_313-[illegible]	IRIS_313-[illegible]
TT	CC	TT	TT	TT	TT	TT	TT	TT
GG	GG	GG	GG	GG	GG	GG	GG	TT
AA	TT	AA	AA	AA	AA	AA	AA	AA
CC	CC	CC	CC	CC	CC	CC	CC	TT
GG	AA	GG	GG	GG	GG	GG	GG	GG
AA	GG	AA	AA	AA	AA	AA	AA	GG
GG	GG	GG	GG	GG	GG	GG	GG	GG
CC	TT	CC	CC	CC	CC	CC	CC	CC

IRIS_313-[illegible]	IRIS_313-[illegible]	IRIS_313-[illegible]	IRIS_313-[illegible]	IRIS_313-[illegible]	IRIS_313-[illegible]	IRIS_313-[illegible]	IRIS_313-[illegible]	IRIS_313-[illegible]
CC	CC	TT	TT	CC	TT	TT	TT	TT
GG	GG	GG	GG	GG	GG	GG	GG	GG
TT	TT	AA	AA	TT	AA	AA	AA	AA
CC	CC	CC	CC	CC	CC	CC	CC	CC
AA	AA	GG	GG	AA	GG	GG	GG	GG
GG	GG	AA	AA	GG	AA	AA	AA	AA
GG	GG	GG	GG	GG	GG	GG	GG	GG
TT	TT	CC	CC	TT	CT	CC	CC	CC

IRIS_313-8	IRIS_313-8	IRIS_313-8	IRIS_313-8	IRIS_313-8	IRIS_313-8	IRIS_313-8	IRIS_313-8	IRIS_313-8
TT	TT	CC	TT	CC	TT	TT	CC	CC
GG	TT	GG	GG	GG	GG	GG	GG	GG
AA	AA	TT	AA	TT	AA	AA	TT	TT
CC	TT	CC	CC	CC	CC	CC	CC	CC
GG	GG	AA	GG	AA	GG	GG	AA	AA
AA	GG	GG	NN	GG	AA	AA	GG	GG
GG	GG	GG	GG	GG	GG	GG	GG	GG
CC	CC	TT	CC	TT	CC	CC	TT	TT

IRIS_313-8	IRIS_313-8	IRIS_313-8	IRIS_313-8	IRIS_313-8	IRIS_313-8	IRIS_313-8	IRIS_313-8	IRIS_313-8
CC	TT	TT	TT	TT	CC	CC	CC	TT
GG	GG	GG	GG	GG	GG	GG	GG	GG
TT	AA	AA	AA	AA	TT	TT	TT	AA
CC	CC	CC	CC	CC	CC	CC	CC	CC
AA	GG	GG	GG	GG	AA	AA	AA	GG
GG	AA	NN	AA	AA	GG	GG	GG	AA
GG	GG	GG	GG	GG	GG	GG	GG	GG
TT	CC	CC	CC	CC	TT	TT	TT	CC

IRIS_313-8	IRIS_313-8	IRIS_313-8	IRIS_313-8	IRIS_313-8	IRIS_313-8	IRIS_313-8	IRIS_313-8	IRIS_313-8
TT	TT	TT	TT	CC	TT	TT	TT	TT
GG	GG	GG	GG	GG	GG	GG	TT	GG
AA	AA	AA	AA	TT	AA	AA	AA	AA
CC	CC	CC	CC	CC	CC	CC	TT	CC
GG	GG	GG	GG	AA	GG	GG	GG	GG
AA	AA	AA	AA	GG	AA	AA	GG	AA
GG	GG	GG	GG	GG	GG	GG	GG	GG
CC	CC	CC	NN	TT	CC	CC	CC	CC

IRIS_313-8	IRIS_313-8	IRIS_313-8	IRIS_313-8	IRIS_313-8	IRIS_313-8	IRIS_313-8	IRIS_313-8	IRIS_313-8
TT	TT	TT	TT	TT	TT	CC	TT	TT
GG	GG	GG	TT	GG	GG	GG	GG	GG
NN	AA	AA	AA	AA	AA	TT	AA	AA
CC	CC	CC	TT	CC	CC	CC	CC	CC
GG	GG	GG	GG	GG	GG	AA	GG	GG
AA	AA	AA	GG	NN	AA	GG	AA	AA
GG	GG	GG	GG	GG	GG	GG	GG	GG
CC	CC	CC	CC	CC	CC	TT	CC	CC

IRIS_313-8	IRIS_313-8	IRIS_313-8	IRIS_313-8	IRIS_313-8	IRIS_313-8	IRIS_313-8	IRIS_313-8	IRIS_313-8
TT	TT	CC	TT	TT	TT	CC	TT	CC
GG	GG	GG	TT	GG	GG	GG	GG	GG
AA	AA	TT	AA	AA	AA	TT	AA	TT
CC	CC	CC	TT	CC	CC	CC	CC	CC
GG	GG	AA	GG	GG	GG	AA	GG	AA
NN	AA	GG	GG	AA	AA	GG	AA	GG
GG	GG	GG	GG	GG	GG	GG	GG	GG
CC	NN	TT	CC	CC	CC	TT	CC	TT

IRIS_313-8	IRIS_313-8	IRIS_313-8	IRIS_313-8	IRIS_313-8	IRIS_313-8	IRIS_313-8	IRIS_313-8	IRIS_313-8
TT	CC	CC	TT	CC	TT	TT	TT	TT
GG	GG	GG	TT	GG	GG	NN	GG	TT
AA	TT	TT	AA	TT	AA	AA	AA	AA
CC	CC	CC	TT	CC	CC	CT	CC	TT
GG	AA	AA	GG	AA	GG	GG	GG	GG
AA	GG	GG	GG	GG	AA	AA	AA	GG
GG	GG	GG	GG	GG	GG	GG	GG	GG
CC	TT	TT	CC	TT	CC	NN	CC	CC

IRIS_313-8	IRIS_313-8	IRIS_313-8	IRIS_313-8	IRIS_313-8	IRIS_313-8	IRIS_313-8	IRIS_313-8	IRIS_313-8
TT	TT	TT	CC	TT	TT	CC	CC	CC
GG	GG	GG	GG	GG	GG	GG	GG	GG
AA	AA	AA	TT	AA	AA	TT	TT	TT
CC	CC	CC	CC	CC	CC	CC	CC	CC
GG	NN	GG	AA	GG	GG	AA	AA	AA
AA	AA	AA	GG	NN	AA	GG	GG	GG
GG	GG	GG	GG	NN	GG	GG	GG	GG
CC	CC	CC	TT	CC	CC	TT	TT	TT

IRIS_313-8	IRIS_313-8	IRIS_313-8	IRIS_313-8	IRIS_313-8	IRIS_313-8	IRIS_313-8	IRIS_313-8	IRIS_313-8
TT	TT	TT	TT	TT	TT	TT	CC	CC
TT	GG	GG	GG	GG	GG	GG	GG	GG
AA	AA	AA	AA	AA	AA	AA	TT	TT
TT	CC	CC	CC	CC	CC	CC	CC	CC
GG	GG	GG	GG	GG	GG	GG	AA	AA
GG	AA	AA	AA	AA	NN	NN	GG	GG
GG	GG	GG	GG	GG	NN	NN	GG	GG
CC	CC	CC	CC	CC	CC	CC	TT	TT

IRIS_313-8	IRIS_313-8	IRIS_313-8	IRIS_313-8	IRIS_313-8	IRIS_313-8	IRIS_313-8	IRIS_313-8	IRIS_313-8
TT	TT	CC	TT	TC	CC	CC	TT	CC
GG	TT	GG	GG	GG	GG	GG	GG	GG
AA	AA	TT	AA	AT	TT	TT	AA	TT
CC	TT	CC	CC	CC	CC	CC	CC	CC
GG	GG	AA	GG	GA	AA	AA	GG	AA
NN	GG	GG	AA	AG	GG	GG	NN	GG
NN	GG	GG	GG	GG	GG	GG	GG	GG
CC	CC	TT	CC	CT	TT	TT	CC	TT

IRIS_313-8	IRIS_313-8	IRIS_313-8	IRIS_313-8	IRIS_313-8	IRIS_313-8	IRIS_313-8	IRIS_313-8	IRIS_313-8
CC	TT	CC	CC	TT	TT	TT	TT	TC
GG	GG	GG	GG	GG	GG	GG	GG	GG
TT	AA	TT	TT	AA	AA	AA	AA	AT
CC	CC	CC	CC	CC	CC	NN	CC	CC
AA	GG	AA	AA	GG	GG	GG	GG	GA
GG	NN	GG	GG	AA	AA	AA	AA	AG
GG	GG	GG	GG	GG	GG	GG	GG	GG
TT	CC	TT	TT	CC	CC	CC	CC	CT

IRIS_313-8	IRIS_313-8	IRIS_313-8	IRIS_313-8	IRIS_313-8	IRIS_313-8	IRIS_313-8	IRIS_313-8	IRIS_313-8
TT	TT	NN	TT	CC	TT	TT	CC	TT
GG	GG	NN	GG	GG	GG	GG	GG	GG
AA	AA	NN	AA	TT	AA	AA	TT	AA
CC	CC	NN	CC	CC	CC	CC	CC	CC
GG	GG	NN	GG	AA	GG	GG	AA	GG
AA	AA	NN	AA	GG	AA	AA	GG	AA
GG	GG	NN	GG	GG	GG	GG	GG	GG
CC	CC	NN	CC	TT	CC	CC	TT	CC

IRIS_313-8	IRIS_313-8	IRIS_313-8	IRIS_313-8	IRIS_313-8	IRIS_313-8	IRIS_313-8	IRIS_313-8	IRIS_313-8
TT	TC	TT	TT	TT	CC	TT	TT	CC
GG	GG	GG	GG	GG	GG	GG	GG	GG
AA	AT	AA	AA	AA	TT	AA	AA	TT
CC	CC	CC	CC	CC	CC	CC	CC	CC
GG	GA	GG	GG	GG	AA	GG	GG	AA
AA	AG	AA	AA	AA	GG	AA	AA	GG
GG	GG	GG	GG	GG	GG	GG	GG	GG
CC	CT	CC	CC	CC	TT	CC	CC	TT

IRIS_313-8	IRIS_313-8	IRIS_313-8	IRIS_313-8	IRIS_313-8	IRIS_313-8	IRIS_313-8	IRIS_313-8	IRIS_313-8
TT	TT	TT	CC	TT	TT	TT	TT	TT
TT	GG	GG	GG	GG	GG	GG	GG	GG
AA	AA	AA	TT	AA	AA	AA	AA	AA
CT	CC	CC	CC	CC	CC	CC	CC	CC
GG	GG	GG	AA	GG	NN	GG	GG	GG
GG	AA	AA	GG	AA	AA	AA	AA	AA
GG	GG	GG	GG	GG	GG	GG	GG	GG
CC	CC	CC	TT	CC	CC	CC	CC	CC

IRIS_313-8	IRIS_313-8	IRIS_313-8	IRIS_313-8	IRIS_313-8	IRIS_313-8	IRIS_313-9	IRIS_313-9	IRIS_313-9
TT	TT	TT	TT	CC	CC	CC	TT	TT
GG	GG	GG	GG	GG	GG	GG	GG	GG
AA	AA	AA	AA	TT	TT	TT	AA	AA
CC	CC	CC	CC	CC	CC	CC	CC	CC
GG	GG	GG	GG	AA	AA	AA	GG	GG
AA	AA	AA	AA	GG	GG	GG	AA	AA
GG	GG	GG	GG	GG	GG	GG	GG	GG
CC	CC	CC	CC	TT	TT	TT	CC	CC

IRIS_313-[illegible]	IRIS_313-[illegible]	IRIS_313-[illegible]	IRIS_313-[illegible]	IRIS_313-[illegible]	IRIS_313-[illegible]	IRIS_313-[illegible]	IRIS_313-[illegible]	IRIS_313-[illegible]
TT	TT	TT	TT	TT	TT	TT	NN	TT
GG	GG	GG	GG	GG	GG	GG	GG	GG
AA	AA	AA	AA	AA	AA	AA	TT	AA
CC	CC	CC	CC	CC	CC	CC	CC	CC
GG	GG	GG	GG	GG	GG	GG	AA	GG
AA	AA	AA	AA	AA	AA	AA	GG	AA
GG	GG	GG	GG	GG	GG	GG	GG	GG
CC	CC	CC	CC	CC	CC	CC	TT	CC

IRIS_313-9	IRIS_313-9	IRIS_313-9	IRIS_313-9	IRIS_313-9	IRIS_313-9	IRIS_313-9	IRIS_313-9	IRIS_313-9
CC	CC	TT	TT	TT	TT	TT	TT	TT
GG	GG	GG	GG	GG	GG	GG	GG	GG
TT	TT	AA	AA	AA	AA	AA	AA	AA
CC	CC	CC	CC	CC	CC	CC	CC	CC
AA	AA	GG	GG	GG	GG	GG	GG	GG
GG	GG	AA	AA	AA	AA	AA	AA	AA
GG	GG	GG	GG	GG	GG	GG	GG	GG
TT	TT	CC	CC	CC	CC	CC	CC	CC

IRIS_313-[illegible]	IRIS_313-[illegible]	IRIS_313-[illegible]	IRIS_313-[illegible]	IRIS_313-[illegible]	IRIS_313-[illegible]	IRIS_313-[illegible]	IRIS_313-[illegible]	IRIS_313-[illegible]
CC	TT	TT	TT	TT	TC	TT	TT	TT
GG	TT	GG	GG	GG	GG	GG	GT	NN
TT	AA	AA	AA	AA	AT	AA	AA	AA
CC	TT	CC	CC	CC	CC	CC	CT	CC
AA	GG	GG	GG	GG	GA	GG	GG	GG
GG	GG	AA	AA	AA	AG	AA	AA	AA
GG	GG	GG	GG	GG	GG	GG	GG	NN
TT	CC	CC	CC	CC	CT	CC	CC	NN

IRIS_313-9	IRIS_313-9	IRIS_313-9	IRIS_313-9	IRIS_313-9	IRIS_313-9	IRIS_313-9	IRIS_313-9	IRIS_313-9
TT	TC	TT	TT	TT	TT	TC	TT	CC
GG	GG	GG	GG	GG	GG	GG	GG	GG
AA	AA	AA	AA	AA	AA	AT	AA	NN
CC	CC	CC	CC	CC	CC	CC	CC	CC
GG	GG	GG	GG	GG	GG	GA	GG	AA
AA	AA	AA	AA	AA	AA	AA	AA	GG
GG	GG	GG	GG	GG	GG	GG	GG	GG
CC	CC	CC	CC	CC	CC	CT	CC	TT

IRIS_313-9	IRIS_313-9	IRIS_313-9	IRIS_313-9	IRIS_313-9	IRIS_313-9	IRIS_313-9	IRIS_313-9	IRIS_313-9
CC	TT	TT	TT	CC	TT	TT	TT	CC
GG	GG	GG	GG	GG	TT	GG	GG	GG
TT	AA	AA	AA	TT	AA	AA	AA	TT
CC	CC	CC	CC	CC	TT	CC	CC	CC
AA	GG	GG	GG	AA	GG	GG	GG	AA
GG	AA	AA	AA	NN	GG	AA	AA	GG
NN	GG	GG	GG	GG	GG	GG	GG	GG
TT	CC	CC	CC	TT	CC	CC	CC	TT

IRIS_313-9	IRIS_313-9	IRIS_313-9	IRIS_313-9	IRIS_313-9	IRIS_313-9	IRIS_313-9	IRIS_313-9	IRIS_313-9
CC	TT	CC	TT	TT	TT	TT	TT	CC
GG	GG	GG	GG	GG	GG	GG	GG	GG
TT	AA	TT	AA	AA	AA	AA	AA	TT
CC	CC	CC	CC	CC	CC	CC	CC	CC
AA	GG	AA	GG	GG	GG	GG	GG	AA
GG	AA	GG	AA	NN	NN	AA	AA	GG
GG	GG	GG	GG	NN	GG	GG	GG	GG
TT	CC	TT	CC	CC	CC	CC	CC	TT

IRIS_313-9	IRIS_313-9	IRIS_313-9	IRIS_313-9	IRIS_313-9	IRIS_313-9	IRIS_313-9	IRIS_313-9	IRIS_313-9
TT	TT	CC	TT	TT	TT	TT	TT	TT
GG	GG	GG	GG	GG	GG	GG	GG	GG
AA	AA	TT	AA	AA	AA	AA	AA	AA
CC	CC	CC	CC	CC	CC	CC	CC	CC
GG	GG	AA	GG	GG	GG	GG	GG	GG
AA	AA	GG	AA	AA	AA	AA	AA	AA
GG	GG	GG	GG	GG	GG	GG	GG	GG
CC	CC	TT	CC	CC	CC	CC	CC	CC

IRIS_313-9	IRIS_313-9	IRIS_313-9	IRIS_313-9	IRIS_313-9	IRIS_313-9	IRIS_313-9	IRIS_313-9	IRIS_313-9
CC	CC	CC	TC	TT	CC	TT	TT	TT
GG	GG	GG	GG	GG	GG	GG	GG	GG
TT	TT	TT	AT	AA	TT	AA	AA	AA
CC	CC	CC	CC	CC	CC	CC	CC	CC
AA	AA	AA	GA	GG	AA	GG	GG	GG
GG	GG	GG	AG	AA	GG	AA	AA	AA
GG	GG	GG	GG	GG	GG	GG	GG	GG
TT	TT	TT	CT	CC	TT	CC	CC	CC

IRIS_313-9	IRIS_313-9	IRIS_313-9	IRIS_313-9	IRIS_313-9	IRIS_313-9	IRIS_313-9	IRIS_313-9	IRIS_313-9
TT	TT	TT	CC	TT	TT	TT	TT	TT
GG	GG	GG	GG	GG	GG	GG	GG	GG
AA	AA	AA	TT	AA	AA	AA	AA	AA
CC	CC	CC	CC	CC	CC	CC	CC	CC
GG	GG	GG	AA	GG	GG	GG	GG	GG
AA	AA	AA	GG	AA	AA	AA	AA	NN
GG	GG	GG	GG	GG	GG	GG	GG	GG
CC	NN	CC	TT	CC	CC	CC	CC	CC

IRIS_313-9	IRIS_313-9	IRIS_313-9	IRIS_313-9	IRIS_313-9	IRIS_313-9	IRIS_313-9	IRIS_313-9	IRIS_313-9
TT	TT	TT	TT	CC	CC	TT	TT	TT
GG	GG	GG	GG	GG	GG	GG	GG	GG
AA	AA	AA	AA	TT	TT	AA	AA	AA
CC	CC	CC	CC	CC	CC	CC	CC	CC
GG	GG	GG	GG	AA	AA	GG	GG	GG
AA	AA	AA	AA	GG	GG	AA	AA	AA
GG	GG	GG	GG	GG	GG	GG	GG	GG
CC	CC	CC	CC	TT	TT	CC	CC	CC

IRIS_313-9	IRIS_313-9	IRIS_313-9	IRIS_313-9	IRIS_313-9	IRIS_313-9	IRIS_313-9	IRIS_313-9	IRIS_313-9
TT	TT	TT	TT	TT	TT	TT	CC	TT
GG	GG	GG	GG	GG	GG	GG	GG	GG
AA	AA	AA	AA	AA	AA	AA	TT	AA
CC	CC	CC	CC	CC	CC	CC	CC	CC
GG	GG	GG	GG	GG	GG	GG	AA	GG
AA	AA	AA	AA	AA	AA	AA	GG	NN
GG	GG	GG	GG	GG	GG	GG	GG	NN
CC	CC	CC	CC	CC	CC	CC	TT	CC

IRIS_313-9	IRIS_313-9	IRIS_313-9	IRIS_313-9	IRIS_313-9	IRIS_313-9	IRIS_313-9	IRIS_313-9	IRIS_313-9
TT	TT	TT	TT	CC	CC	TT	TT	CC
GG	GG	GG	GG	GG	GG	TT	GG	GG
AA	AA	AA	AA	NN	TT	AA	AA	TT
CC	CC	CC	CC	CC	CC	TT	CC	CC
GG	GG	GG	GG	AA	AA	GG	GG	AA
AA	AA	AA	AA	GG	GG	GG	AA	GG
GG	GG	GG	GG	GG	GG	GG	GG	GG
CC	CC	CC	CC	TT	TT	CC	CC	TT

IRIS_313-9	IRIS_313-9	IRIS_313-9	IRIS_313-9	IRIS_313-9	IRIS_313-9	IRIS_313-9	IRIS_313-9	IRIS_313-9
CC	TT	TT	CC	TT	TC	TT	TT	TT
GG	GG	GG	GG	GG	GG	GG	GG	GG
TT	AA	AA	TT	AA	AT	AA	AA	AA
CC	CC	CC	CC	CC	CC	CC	CC	CC
AA	GG	GG	AA	GG	AA	GG	GG	GG
GG	AA	AA	GG	AA	NN	AA	AA	NN
GG	GG	GG	GG	GG	GG	GG	GG	GG
TT	CC	CC	TT	CC	NN	CC	CC	CC

IRIS_313-9	IRIS_313-9	IRIS_313-9	IRIS_313-9	IRIS_313-9	IRIS_313-9	IRIS_313-9	IRIS_313-9	IRIS_313-9
TT	TC	CC	TC	TT	CC	TT	TT	CC
GG	GG	GG	GG	GG	GG	GG	GG	GG
AA	AT	TT	AA	AA	TT	AA	AA	TT
CC	CC	CC	CC	CC	CC	CC	CC	CC
GG	AA	AA	GG	GG	AA	GG	GG	AA
AA	AG	GG	AA	AA	GG	AA	AA	GG
GG	GG	GG	GG	GG	GG	GG	AA	GG
CC	NN	TT	CC	CC	TT	CC	CC	TT

IRIS_313-9	IRIS_313-9	IRIS_313-9	IRIS_313-9	IRIS_313-9	IRIS_313-9	IRIS_313-9	IRIS_313-9	IRIS_313-9
TT	TT	TT	TT	CC	CC	TT	TT	CC
GG	GG	GG	GG	GG	GG	GG	GG	GG
AA	AA	AA	AA	TT	TT	AA	AA	TT
CC	CC	CC	CC	CC	CC	CC	CC	CC
GG	GG	GG	GG	AA	AA	GG	GG	AA
AA	AA	AA	AA	GG	GG	AA	AA	GG
GG	GG	GG	GG	GG	GG	GG	GG	GG
CC	CC	CC	CC	TT	TT	CC	CC	TT

IRIS_313-9	IRIS_313-9	IRIS_313-9	IRIS_313-9	IRIS_313-9	IRIS_313-9	IRIS_313-9	IRIS_313-9	IRIS_313-9
TT	CC	TT	CC	TT	CC	TT	TT	TT
GG	GG	GG	GG	GG	GG	GG	GG	GG
AA	TT	AA	TT	AA	TT	AA	AA	AA
CC	CC	CC	CC	CC	CC	CC	CC	CC
GG	AA	GG	AA	GG	AA	GG	GG	GG
NN	GG	AA	GG	AA	GG	AA	AA	AA
GG	GG	GG	GG	GG	GG	GG	GG	GG
CC	TT	CC	TT	CC	TT	CC	CC	CC

IRIS_313-9	IRIS_313-9	IRIS_313-9	IRIS_313-9	IRIS_313-9	IRIS_313-9	IRIS_313-9	IRIS_313-9	IRIS_313-9
CC	TT	TT	TT	TT	TT	CC	TT	CC
GG	GG	GG	GG	GG	GT	GG	GG	GG
TT	AA	AA	AA	AA	AA	TT	AA	TT
CC	CC	CC	CC	CC	CT	CC	CC	CC
AA	GG	GG	GG	GG	GG	AA	GG	AA
GG	AA	AA	AA	AA	GG	GG	AA	GG
GG	GG	GG	GG	GG	GG	GG	GG	GG
TT	CC	CC	CC	CC	CC	TT	CC	TT

IRIS_313-9	IRIS_313-9	IRIS_313-9	IRIS_313-9	IRIS_313-9	IRIS_313-9	IRIS_313-9	IRIS_313-9	IRIS_313-9
CC	TT	CC	TT	CC	TT	TT	TT	CC
GG	GG	GG	GG	GG	GG	GG	GG	GG
TT	AA	TT	AA	TT	AA	AA	AA	TT
CC	CC	CC	CC	CC	CC	CC	CC	CC
AA	GG	AA	GG	AA	GG	GG	GG	AA
GG	AA	GG	AA	GG	AA	AA	AA	GG
GG	GG	GG	GG	GG	GG	GG	GG	GG
TT	CC	TT	CC	TT	CC	CC	CC	TT

IRIS_313-[illegible]	IRIS_313-[illegible]	IRIS_313-[illegible]	IRIS_313-[illegible]	IRIS_313-[illegible]	IRIS_313-[illegible]	IRIS_313-[illegible]	IRIS_313-[illegible]	IRIS_313-[illegible]
TT	TT	TT	CC	TT	TT	TT	TT	TT
GG	GG	GG	GG	GG	GG	GG	GG	GG
AA	AA	AA	TT	AA	AA	AA	AA	AA
CC	CC	CC	CC	CC	CC	CC	CC	CC
GG	GG	GG	AA	GG	GG	GG	GG	GG
AA	AA	AA	GG	AA	AA	AA	AA	AA
GG	GG	GG	GG	GG	GG	GG	GG	GG
CC	CC	CC	TT	CC	CC	CC	CC	CC

IRIS_313-9	IRIS_313-9	IRIS_313-9	IRIS_313-9	IRIS_313-9	IRIS_313-9	IRIS_313-9	IRIS_313-9	IRIS_313-9
TT	TT	TT	TT	CC	TT	TT	TT	TT
GG	GG	GG	GG	GG	GG	GG	GG	GG
AA	AA	AA	AA	TT	AA	AA	AA	AA
CC	CC	CC	CC	CC	CC	CC	CC	CC
GG	GG	GG	GG	AA	GG	GG	GG	GG
AA	AA	AA	AA	GG	AA	AA	AA	AA
GG	GG	GG	GG	GG	GG	GG	GG	GG
CC	CC	CC	CC	TT	CC	CC	CC	CC

IRIS_313-9	IRIS_313-9	IRIS_313-9	IRIS_313-9	IRIS_313-9	IRIS_313-9	IRIS_313-9	IRIS_313-9	IRIS_313-9
TT	TT	TT	CC	TT	TT	TT	TT	CC
GG	TT	GG	GG	GG	GG	GG	GG	GG
AA	AA	AA	TT	AA	AA	AA	AA	TT
CC	TT	CC	CC	CC	CC	CC	CC	CC
GG	GG	GG	AA	GG	GG	GG	GG	AA
AA	GG	AA	GG	AA	AA	AA	AA	GG
GG	GG	GG	GG	GG	GG	GG	AA	GG
CC	CC	CC	TT	CC	CC	CC	CC	TT

IRIS_313-9	IRIS_313-9	IRIS_313-9	IRIS_313-9	IRIS_313-9	IRIS_313-9	IRIS_313-9	IRIS_313-9	IRIS_313-9
TT	TT	TT	TT	CC	TT	TT	CC	TT
GG	TT	GG	GG	GG	GG	GG	GG	GG
AA	AA	AA	AA	TT	AA	AA	TT	AA
CC	TT	CC	CC	CC	CC	CC	CC	CC
GG	GG	GG	GG	AA	GG	GG	AA	GG
AA	GG	AG	AA	GG	AA	AA	GG	AA
GG	GG	GG	GG	GG	GA	GG	GG	GG
CC	CC	CC	CC	TT	CC	CC	TT	CC

IRIS_313-9	IRIS_313-9	IRIS_313-9	IRIS_313-9	IRIS_313-9	IRIS_313-9	IRIS_313-9	IRIS_313-9	IRIS_313-9
TT	CC	TT	TT	TT	TT	CC	TT	CC
GG	GG	GG	GG	GG	GG	GG	GG	GG
AA	TT	AA	AA	AA	AA	TT	AA	TT
CC	CC	CC	CC	CC	CC	CC	CC	CC
GG	AA	GG	GG	GG	GG	AA	GG	AA
AA	GG	AA	AA	AA	AA	GG	AA	GG
GG	GG	GG	GG	GG	GG	GG	GG	GG
NN	TT	CC	CC	CC	CC	TT	CC	TT

IRIS_313-9	IRIS_313-9	IRIS_313-9	IRIS_313-9	IRIS_313-9	IRIS_313-9	IRIS_313-9	IRIS_313-9	IRIS_313-9
CC	CC	TT	TT	TT	TT	TT	CC	CC
GG	GG	GG	GG	GG	GG	GG	GG	GG
TT	TT	AA	AA	AA	AA	AA	TT	TT
CC	CC	CC	CC	CC	CC	CC	CC	CC
AA	AA	GG	GG	GG	GG	GG	AA	AA
GG	GG	AA	AA	AA	AA	AA	GG	GG
GG	GG	GG	GG	GG	GG	GG	GG	GG
TT	TT	CC	CC	CC	CC	CC	TT	TT

IRIS_313-[illegible]	IRIS_313-[illegible]	IRIS_313-[illegible]	IRIS_313-[illegible]	IRIS_313-[illegible]	IRIS_313-[illegible]	IRIS_313-[illegible]	IRIS_313-[illegible]	IRIS_313-[illegible]
TT	TT	TT	TT	CC	CC	TT	CC	TT
GG	GG	GG	GG	GG	GG	GG	GG	GG
AA	AA	AA	AA	TT	TT	AA	TT	AA
CC	CC	CC	CC	CC	CC	CC	CC	CC
GG	GG	GG	GG	AA	AA	GG	AA	GG
AA	AA	AA	AA	GG	GG	AA	GG	AA
GG	GG	GG	GG	GG	GG	GG	GG	GG
CC	CC	CC	CC	TT	TT	CC	TT	CC

IRIS_313-9	IRIS_313-9	IRIS_313-9	IRIS_313-9	IRIS_313-9	IRIS_313-9	IRIS_313-9	IRIS_313-9	IRIS_313-9
CC	CC	CC	CC	TT	CC	TT	CC	CC
GG	GG	GG	GG	TT	GG	GG	GG	GG
TT	TT	TT	TT	AA	TT	AA	TT	TT
CC	CC	CC	CC	CT	CC	CC	CC	CC
AA	AA	AA	AA	GG	AA	GG	AA	AA
GG	GG	GG	GG	GG	GG	AA	GG	GG
GG	GG	GG	GG	GG	GG	GG	GG	GG
TT	TT	TT	TT	CC	TT	CC	TT	TT

IRIS_313-9	IRIS_313-9	IRIS_313-9	IRIS_313-9	IRIS_313-9	IRIS_313-9	IRIS_313-9	IRIS_313-9	IRIS_313-9
TT	TT	CC	TT	CC	CC	CC	CC	TT
GG	GG	GG	GG	GG	GG	GG	GG	GG
AA	AA	TT	AA	TT	TT	TT	NN	AA
CC	CC	CC	CC	CC	CC	CC	CC	CC
GG	GG	AA	GG	AA	AA	AA	AA	GG
AA	AA	GG	AA	GG	GG	GG	GG	AA
GG	GG	GG	GG	GG	GG	GG	GG	GG
CC	CC	TT	CC	TT	TT	TT	TT	NN

IRIS_313-[illegible]	IRIS_313-[illegible]	IRIS_313-[illegible]	IRIS_313-[illegible]	IRIS_313-[illegible]	IRIS_313-[illegible]	IRIS_313-[illegible]	IRIS_313-[illegible]	IRIS_313-[illegible]
TT	TT	TT	TT	CC	CC	CC	CC	TT
GG	GG	GG	GG	GG	GG	GG	GG	TT
AA	AA	AA	AA	TT	TT	TT	TT	AA
CC	CC	CC	CC	CC	CC	CC	CC	TT
GG	GG	GG	GG	AA	AA	AA	AA	GG
AA	AA	AA	AA	GG	GG	GG	GG	GG
GG	GG	GG	GG	GG	GG	GG	GG	GG
CC	CC	CC	CC	TT	TT	TT	TT	CC

IRIS_313-9	IRIS_313-9	IRIS_313-9	IRIS_313-9	IRIS_313-9	IRIS_313-9	IRIS_313-9	IRIS_313-9	IRIS_313-9
TT	TT	CC	TT	CC	CC	CC	CC	CC
GG	GG	GG	GG	GG	GG	GG	GG	GG
AA	AA	TT	AA	TT	TT	TT	TT	TT
CC	CC	CC	CC	CC	CC	CC	CC	CC
GG	GG	AA	GG	AA	AA	AA	AA	AA
AA	AA	GG	AA	GG	GG	GG	GG	GG
GG	GG	GG	GG	GG	GG	GG	GG	GG
NN	CC	TT	CC	TT	TT	TT	TT	TT

IRIS_313-9	IRIS_313-9	IRIS_313-9	IRIS_313-9	IRIS_313-9	IRIS_313-9	IRIS_313-9	IRIS_313-9	IRIS_313-9
CC	TT	TT	TT	TT	TT	TT	CC	CC
GG	GG	GG	GG	GG	GG	GG	GG	NN
TT	AA	AA	AA	AA	AA	AA	TT	TT
CC	CC	CC	CC	CC	CC	CC	CC	CC
AA	GG	GG	GG	GG	GG	GG	AA	AA
GG	AA	AA	AA	AA	NN	AA	GG	GG
GG	GG	GG	GG	GG	GG	GG	GG	GG
TT	CC	CC	CC	CC	CC	CC	TT	TT

IRIS_313-9	IRIS_313-9	IRIS_313-9	IRIS_313-9	IRIS_313-9	IRIS_313-9	IRIS_313-9	IRIS_313-9	IRIS_313-9
TT	TT	CC	TT	TT	TT	CC	TT	CC
GG	GG	GG	GG	GG	GG	GG	GG	GG
AA	AA	TT	AA	AA	AA	TT	AA	TT
CC	CC	CC	CC	CC	CC	CC	CC	CC
GG	GG	AA	GG	GG	GG	AA	GG	AA
AA	AA	GG	NN	AA	AA	GG	AA	GG
GG	GG	GG	GG	GG	GG	GG	GG	GG
CC	CC	TT	CC	NN	CC	TT	CC	TT

IRIS_313-9	IRIS_313-9	IRIS_313-9	IRIS_313-9	IRIS_313-9	IRIS_313-9	IRIS_313-9	IRIS_313-9	IRIS_313-9
TT	CC	TT	TT	TT	TT	CC	TT	TT
GG	GG	GG	GG	GG	GG	GG	GG	TT
AA	TT	AA	AA	AA	AA	TT	AA	AA
CC	CC	CC	CC	CC	CC	CC	CC	TT
GG	AA	GG	GG	GG	GG	AA	GG	GG
AA	GG	AA	AA	AA	AA	GG	AA	GG
AA	GG	GG	GG	GG	GG	GG	GG	GG
CC	TT	CC	CC	CC	CC	TT	CC	CC

IRIS_313-9	IRIS_313-9	IRIS_313-9	IRIS_313-9	IRIS_313-9	IRIS_313-1	IRIS_313-1	IRIS_313-1	IRIS_313-1
CC	TT	TT	CC	CC	TT	TT	TT	TT
GG	GG	GG	GG	GG	GG	GG	GG	GG
TT	AA	AA	TT	TT	AA	AA	AA	AA
CC	CC	CC	CC	CC	CC	CC	CC	CC
AA	GG	GG	AA	AA	GG	GG	GG	GG
GG	AA	AA	GG	GG	AA	AA	AA	AA
GG	GG	GG	GG	GG	GG	GG	GG	GG
TT	CC	CC	TT	TT	CC	CC	CC	CC

IRIS_313-1	IRIS_313-1	IRIS_313-1	IRIS_313-1	IRIS_313-1	IRIS_313-1	IRIS_313-1	IRIS_313-1	IRIS_313-1
TT	CC	TT	TT	CC	TT	TT	TT	TT
GG	GG	GG	TT	GG	GG	GG	TT	GG
AA	TT	AA	AA	TT	AA	AA	AA	AA
CC	CC	CC	TT	CC	CC	CC	TT	CC
GG	AA	GG	GG	AA	GG	GG	GG	GG
AA	GG	AA	GG	GG	AA	AA	GG	AA
GG	GG	GG	GG	GG	GG	GG	GG	GG
CC	TT	CC	CC	TT	CC	CC	CC	CC

IRIS_313-1	IRIS_313-1	IRIS_313-1	IRIS_313-1	IRIS_313-1	IRIS_313-1	IRIS_313-1	IRIS_313-1	IRIS_313-1
TT	TT	CC	TT	TT	TT	TT	TT	CC
GG	GG	GG	GG	GG	GG	GG	GG	GG
AA	AA	TT	AA	AA	AA	AA	AA	TT
CC	CC	CC	CC	CC	CC	CC	CC	CC
GG	GG	AA	GG	GG	GG	GG	GG	AA
AA	AA	GG	AA	AA	AA	AA	AA	GG
GG	GG	GG	GG	GG	GG	GG	GG	GG
CC	CC	TT	CC	CC	CC	CC	CC	TT

IRIS_313-1	IRIS_313-1	IRIS_313-1	IRIS_313-1	IRIS_313-1	IRIS_313-1	IRIS_313-1	IRIS_313-1	IRIS_313-1
TT	CC	CC	CC	CC	CC	CC	CC	CC
GG	GG	GG	GG	GG	GG	GG	GG	GG
AA	TT	TT	TT	TT	TT	TT	TT	TT
CC	CC	CC	CC	CC	CC	CC	CC	CC
GG	AA	AA	AA	AA	AA	AA	AA	AA
AA	GG	GG	GG	GG	GG	GG	GG	GG
GG	GG	GG	GG	GG	GG	GG	GG	GG
CC	TT	TT	TT	TT	TT	TT	TT	TT

IRIS_313-1	IRIS_313-1	IRIS_313-1	IRIS_313-1	IRIS_313-1	IRIS_313-1	IRIS_313-1	IRIS_313-1	IRIS_313-1
CC	CC	CC	CC	TT	CC	CC	CC	CC
GG	GG	GG	GG	GG	GG	GG	GG	GG
TT	TT	TT	TT	AA	TT	TT	TT	TT
CC	CC	CC	CC	CC	CC	CC	CC	CC
AA	AA	AA	AA	GG	AA	AA	AA	AA
GG	GG	GG	GG	AA	GG	GG	GG	GG
GG	GG	GG	GG	GG	GG	GG	GG	GG
TT	TT	TT	TT	CC	TT	TT	TT	TT

IRIS_313-1	IRIS_313-1	IRIS_313-1	IRIS_313-1	IRIS_313-1	IRIS_313-1	IRIS_313-1	IRIS_313-1	IRIS_313-1
CC	CC	CC	CC	CC	CC	CC	CC	CC
GG	GG	GG	GG	GG	GG	GG	GG	GG
TT	TT	TT	TT	TT	TT	TT	TT	TT
CC	CC	CC	CC	CC	CC	CC	CC	CC
AA	AA	AA	AA	AA	AA	AA	AA	AA
GG	GG	GG	GG	GG	GG	GG	GG	GG
GG	GG	GG	GG	GG	GG	GG	GG	GG
TT	TT	TT	TT	TT	TT	TT	TT	TT

IRIS_313-1	IRIS_313-1	IRIS_313-1	IRIS_313-1	IRIS_313-1	IRIS_313-1	IRIS_313-1	IRIS_313-1	IRIS_313-1
TT	TT	TT	CC	TT	TT	CC	CC	TT
GG	GG	GG	GG	GG	GG	GG	GG	GG
AA	AA	AA	TT	AA	AA	TT	TT	AA
CC	CC	CC	CC	CC	CC	CC	CC	CC
GG	GG	GG	AA	GG	GG	AA	AA	GG
AA	AA	AA	GG	AA	AA	GG	GG	AA
GG	GG	GG	GG	GG	GG	GG	GG	GG
CC	CC	CC	TT	CC	CC	TT	TT	CC

IRIS_313-1	IRIS_313-1	IRIS_313-1	IRIS_313-1	IRIS_313-1	IRIS_313-1	IRIS_313-1	IRIS_313-1	IRIS_313-1
TT	TT	TT	TT	TT	CC	TT	TT	TT
GG	GG	GG	GG	GG	GG	GG	GG	GG
AA	AA	AA	AA	AA	TT	AA	AA	AA
CC	CC	CC	CC	CC	CC	CC	CC	CC
GG	GG	GG	GG	GG	AA	GG	GG	GG
AA	AA	AA	AA	AA	GG	AA	AA	AA
GG	GG	GG	GG	GG	GG	GG	GG	GG
CC	CC	CC	CC	CC	TT	CC	CC	CC

IRIS_313-1	IRIS_313-1	IRIS_313-1	IRIS_313-1	IRIS_313-1	IRIS_313-1	IRIS_313-1	IRIS_313-1	IRIS_313-1
CC	TT	TT	TT	TT	CC	TT	TT	TT
GG	GG	GG	GG	GG	GG	GG	GG	GG
TT	AA	AA	AA	AA	TT	AA	AA	AA
CC	CC	CC	CC	CC	CC	CC	CC	CC
AA	GG	GG	GG	GG	AA	GG	GG	GG
GG	AA	AA	AA	AA	GG	AA	AA	AA
GG	GG	GG	GG	GG	GG	GG	GG	GG
TT	CC	CC	CC	CC	TT	CC	CC	CC

IRIS_313-1	IRIS_313-1	IRIS_313-1	IRIS_313-1	IRIS_313-1	IRIS_313-1	IRIS_313-1	IRIS_313-1	IRIS_313-1
TT	TT	TT	CC	TT	TT	TT	TT	TT
GG	GG	GG	GG	GG	GG	GG	GG	GG
AA	AA	AA	TT	AA	AA	AA	AA	AA
CC	CC	CC	CC	CC	CC	CC	CC	CC
GG	GG	GG	AA	GG	GG	GG	GG	GG
AA	AA	AA	NN	AA	AA	AA	AA	AA
GG	GG	GG	GG	GG	GG	GG	GG	GG
CC	CC	CC	TT	CC	CC	CC	CC	CC

IRIS_313-1	IRIS_313-1	IRIS_313-1	IRIS_313-1	IRIS_313-1	IRIS_313-1	IRIS_313-1	IRIS_313-1	IRIS_313-1
CC	TT	TT	TT	TT	TT	CC	TT	TT
GG	GG	GG	GG	GG	GG	GG	GG	GG
TT	AA	AA	AA	AA	AA	TT	AA	AA
CC	CC	CC	CC	CC	CC	CC	CC	CC
AA	GG	GG	GG	GG	GG	AA	GG	GG
GG	AA	AA	AA	AA	AA	GG	AA	AA
GG	GG	GG	GG	GG	GG	GG	GG	GG
TT	CC	CC	CC	CC	CC	TT	CC	CC

IRIS_313-1	IRIS_313-1	IRIS_313-1	IRIS_313-1	IRIS_313-1	IRIS_313-1	IRIS_313-1	IRIS_313-1	IRIS_313-1
CC	TT	TT	TT	TT	CC	TT	TT	TT
GG	GG	GG	GG	GG	GG	GG	GG	GG
TT	AA	AA	AA	AA	TT	AA	AA	AA
CC	CC	CC	CC	CC	CC	CC	CC	CC
AA	GG	GG	GG	GG	AA	GG	GG	GG
GG	AA	AA	AA	AA	GG	AA	AA	AA
GG	GG	GG	GG	GG	GG	GG	GG	GG
TT	CC	CC	CC	CC	TT	CC	CC	CC

IRIS_313-1	IRIS_313-1	IRIS_313-1	IRIS_313-1	IRIS_313-1	IRIS_313-1	IRIS_313-1	IRIS_313-1	IRIS_313-1
TT	TT	TT	TT	TT	TT	CC	TT	TT
GG	GG	GG	GG	GG	GG	GG	GG	GG
AA	AA	AA	AA	AA	AA	TT	AA	AA
CC	CC	CC	CC	CC	CC	CC	CC	CC
GG	GG	GG	GG	GG	GG	AA	GG	GG
AA	AA	AA	AA	AA	AA	GG	AA	AA
GG	GG	NN	GG	GG	GG	GG	GG	GG
CC	CC	CC	CC	CC	CC	TT	CC	CC

IRIS_313-1	IRIS_313-1	IRIS_313-1	IRIS_313-1	IRIS_313-1	IRIS_313-1	IRIS_313-1	IRIS_313-1	IRIS_313-1
TT	TT	TT	TT	TT	CC	TT	TT	TT
GG	GG	GG	GG	GG	GG	GG	GG	GG
AA	AA	AA	AA	AA	TT	AA	AA	AA
CC	CC	CC	CC	CC	CC	CC	CC	CC
GG	GG	GG	GG	GA	AA	GG	GG	GG
AA	AA	AA	AA	AA	GG	AA	AA	AA
GG	GG	GG	GG	GG	GG	GG	GG	NN
CC	CC	CC	CC	CC	TT	CC	CC	CC

IRIS_313-1	IRIS_313-1	IRIS_313-1	IRIS_313-1	IRIS_313-1	IRIS_313-1	IRIS_313-1	IRIS_313-1	IRIS_313-1
CC	TT	TT	TT	TT	TT	TT	TT	TT
GG	GG	GG	GG	GG	GG	GG	GG	GG
TT	AA	AA	NN	AA	AA	AA	AA	AA
CC	CC	CC	CC	CT	CC	CC	CC	CC
AA	GG	GG	GG	GG	GG	GG	GG	GG
GG	AA	AA	AA	NN	AA	AA	AA	AA
GG	GG	GG	GG	NN	GG	GG	GG	GG
TT	CC	CC	CC	CC	CC	CC	CC	CC

IRIS_313-1	IRIS_313-1	IRIS_313-1	IRIS_313-1	IRIS_313-1	IRIS_313-1	IRIS_313-1	IRIS_313-1	IRIS_313-1
TT	TT	TT	TT	TT	TT	CC	TT	TT
GG	GG	GG	GG	TT	GG	GG	GG	GG
AA	AA	NN	AA	AA	AA	TT	AA	AA
CC	CC	CC	CC	TT	CC	CC	CC	CC
GG	GG	GG	GG	GG	GG	AA	GG	NN
AA	NN	AA	AA	GG	AA	GG	NN	AA
GG	GG	GG	GG	GG	GG	GG	GG	GG
CC	CC	CC	CC	CC	CC	TT	CC	CT

IRIS_313-1	IRIS_313-1	IRIS_313-1	IRIS_313-1	IRIS_313-1	IRIS_313-1	IRIS_313-1	IRIS_313-1	IRIS_313-1
CC	TT	TT	TT	TT	TT	TT	TT	TT
GG	GG	GG	GG	GG	GG	GG	GG	GG
TT	AA	AA	AA	AA	AA	AA	AA	AA
CC	CC	CC	CC	CC	CC	CC	CC	CC
AA	GG	GG	GG	GG	GG	GG	GG	NN
GG	AA	AA	AA	AA	AA	AA	NN	NN
GG	GG	GG	GG	GG	GG	GG	GG	GG
TT	CC	CC	CC	CC	CC	CC	CC	CC

IRIS_313-1	IRIS_313-1	IRIS_313-1	IRIS_313-1	IRIS_313-1	IRIS_313-1	IRIS_313-1	IRIS_313-1	IRIS_313-1
TT	TT	TT	TT	TT	TT	TT	TT	TT
GG	GG	GG	GG	GG	GG	GG	GG	GG
AA	AA	AA	AA	AA	AA	AA	AA	AA
CC	CC	CC	CC	CC	CC	CC	CC	CC
GG	GG	GG	GG	GG	GG	GG	GG	GG
AA	AA	AA	NN	AA	AA	NN	AA	AA
GG	GG	GG	GG	GG	NN	GG	GG	GG
CC	CC	CC	CC	CC	CC	CC	CC	CC

IRIS_313-1	IRIS_313-1	IRIS_313-1	IRIS_313-1	IRIS_313-1	IRIS_313-1	IRIS_313-1	IRIS_313-1	IRIS_313-1
TT	TT	CC	CC	TT	CC	CC	TT	TC
GG	GG	GG	GG	GG	GG	GG	GG	GG
AA	AA	TT	TT	AA	TT	TT	AA	NN
CC	CC	CC	CC	CC	CC	CC	CC	CC
GG	GG	AA	AA	GG	AA	AA	GG	GA
AA	AA	GG	GG	AA	GG	GG	AA	AG
GG	GG	GG	GG	GG	GG	GG	GG	GG
CC	CC	TT	TT	CC	TT	TT	CC	NN

IRIS_313-1	IRIS_313-1	IRIS_313-1	IRIS_313-1	IRIS_313-1	IRIS_313-1	IRIS_313-1	IRIS_313-1	IRIS_313-1
TT	TT	TT	TT	CC	TT	TC	CC	TT
GG	GG	GG	GG	GG	GG	GG	GG	GG
AA	AA	AA	AA	TT	AA	AT	TT	AA
CC	CC	CC	CC	CC	CC	CC	CC	CC
GG	GG	GG	GG	AA	GG	GA	AA	GG
AA	AA	AA	AA	GG	AA	AG	GG	AA
GG	GG	GG	GG	GG	GG	GG	GG	GG
CC	CC	CC	CC	TT	CC	NN	TT	CC

IRIS_313-1	IRIS_313-1	IRIS_313-1	IRIS_313-1	IRIS_313-1	IRIS_313-1	IRIS_313-1	IRIS_313-1	IRIS_313-1
TT	TT	TC	CC	CC	TT	TT	TT	TT
GG	GG	GG	GG	GG	GG	GG	GG	GG
AA	AA	AT	TT	TT	AA	AA	AA	AA
CC	CC	CC	CC	CC	CC	CC	CC	CC
GG	GG	GA	AA	AA	GG	GG	GG	GG
AA	AA	AA	GG	GG	NN	AA	AA	NN
GG	GG	GG	GG	GG	NN	GG	GG	GG
CC	CC	CT	TT	TT	CC	CC	CC	CC

IRIS_313-1	IRIS_313-1	IRIS_313-1	IRIS_313-1	IRIS_313-1	IRIS_313-1	IRIS_313-1	IRIS_313-1	IRIS_313-1
TT	CC	TT	TT	TT	TT	TT	TT	TT
GG	GG	GG	GG	GG	GG	GG	GG	GG
AA	TT	AA	AA	AA	AA	AA	AA	AA
CC	CC	CC	CC	CC	CC	CC	CC	CC
GG	AA	GG	GG	GG	GG	GG	GG	GG
AA	GG	AA	AA	AA	AA	AA	AA	AA
GG	GG	GG	GG	GG	GG	GG	GG	GG
CC	TT	CC	CC	CC	CC	CC	CC	CC

IRIS_313-1	IRIS_313-1	IRIS_313-1	IRIS_313-1	IRIS_313-1	IRIS_313-1	IRIS_313-1	IRIS_313-1	IRIS_313-1
TT	TT	TT	TT	CC	CC	TT	TT	TT
GG	GG	GG	GG	GG	GG	GG	GG	GG
AA	AA	AA	AA	TT	TT	AA	AA	AA
CC	CC	CC	CC	CC	CC	CC	CC	CC
GG	GG	GG	GG	AA	AA	GG	GG	GG
AA	AA	AA	AA	GG	GG	AA	AA	AA
GG	GG	GG	GG	GG	GG	GG	GG	GG
CC	CC	CC	CC	TT	TT	CC	CC	CC

IRIS_313-1	IRIS_313-1	IRIS_313-1	IRIS_313-1	IRIS_313-1	IRIS_313-1	IRIS_313-1	IRIS_313-1	IRIS_313-1
TT	TT	TT	TT	TT	CC	TT	CC	TT
GG	GG	GT	TT	GG	GG	TT	GG	GG
AA	AA	AA	AA	AA	TT	AA	TT	AA
CC	CC	CT	TT	CC	CC	TT	CC	CC
GG	GG	GG	GG	GG	AA	GG	AA	GG
AA	AA	NN	GG	AA	GG	GG	GG	AA
GG	GG	GG	GG	GG	GG	GG	GG	GG
CC	CC	CC	CC	CC	TT	CC	TT	CC

IRIS_313-1	IRIS_313-1	IRIS_313-1	IRIS_313-1	IRIS_313-1	IRIS_313-1	IRIS_313-1	IRIS_313-1	IRIS_313-1
TT	TT	TT	TT	TT	TT	CC	TT	TT
TT	GG	TT	GG	GG	GG	GG	GG	GG
AA	AA	AA	AA	AA	AA	TT	AA	AA
TT	CC	TT	CC	CC	CC	CC	CC	CC
GG	GG	GG	GG	GG	GG	AA	GG	GG
GG	AA	GG	AA	AA	AA	GG	AA	AA
GG	GG	GG	GG	GG	GG	GG	GG	GG
CC	CC	NN	CC	CC	CC	TT	CC	CC

IRIS_313-1	IRIS_313-1	IRIS_313-1	IRIS_313-1	IRIS_313-1	IRIS_313-1	IRIS_313-1	IRIS_313-1	IRIS_313-1
TT	TT	CC	CC	TT	TT	CC	CC	CC
GG	GG	GG	GG	GG	GG	GG	GG	GG
AA	AA	TT	TT	AA	AA	TT	TT	TT
CC	CC	CC	CC	CC	CC	CC	CC	CC
GG	GG	AA	AA	GG	GG	AA	AA	AA
AA	AA	GG	GG	AA	AA	GG	GG	GG
GG	GG	GG	GG	GG	GG	GG	GG	GG
CC	CC	TT	TT	CC	CC	TT	TT	TT

IRIS_313-1	IRIS_313-1	IRIS_313-1	IRIS_313-1	IRIS_313-1	IRIS_313-1	IRIS_313-1	IRIS_313-1	IRIS_313-1
TT	TT	CC	CC	CC	CC	TT	TT	TT
GG	GG	GG	GG	GG	GG	GG	GG	GG
AA	AA	TT	TT	TT	TT	AA	AA	AA
CC	CC	CC	CC	CC	CC	CC	CC	CC
GG	GG	AA	AA	AA	AA	GG	GG	GG
AA	AA	GG	GG	GG	GG	AA	AA	AA
GG	GG	GG	NN	GG	GG	GG	GG	GG
CC	CC	TT	TT	TT	TT	CC	CC	CC

IRIS_313-1	IRIS_313-1	IRIS_313-1	IRIS_313-1	IRIS_313-1	IRIS_313-1	IRIS_313-1	IRIS_313-1	IRIS_313-1
TT	TT	CC	CC	CC	CC	CC	CC	TT
GG	GG	GG	GG	GG	GG	GG	GG	GG
AA	AA	TT	TT	TT	TT	TT	TT	AA
CC	CC	CC	CC	CC	CC	CC	CC	CC
GG	GG	AA	AA	AA	AA	AA	AA	GG
AA	AA	GG	GG	GG	GG	GG	GG	AA
GG	GG	GG	GG	GG	NN	GG	GG	GG
CC	CC	TT	TT	TT	TT	TT	TT	CC

IRIS_313-1	IRIS_313-1	IRIS_313-1	IRIS_313-1	IRIS_313-1	IRIS_313-1	IRIS_313-1	IRIS_313-1	IRIS_313-1
TT	TT	TT	TT	TT	TT	TT	TT	TT
GG	GG	TT	GG	GG	TT	GG	TT	TT
AA	AA	AA	AA	AA	AA	AA	AA	AA
CC	CC	TT	CT	CC	CT	CC	TT	TT
GG	GG	GG	GG	GG	GG	GG	GG	GG
AA	AA	GG	NN	AA	GG	AA	GG	GG
AA	AA	GG	GG	AA	GG	GG	GG	GG
CC	CC	CC	CC	CC	NN	CC	CC	CC

IRIS_313-1	IRIS_313-1	IRIS_313-1	IRIS_313-1	IRIS_313-1	IRIS_313-1	IRIS_313-1	IRIS_313-1	IRIS_313-1
TT	TT	TT	TT	TT	TT	TT	CC	CC
TT	TT	TT	GG	GG	GG	GG	GG	GG
AA	AA	AA	AA	AA	AA	AA	TT	TT
TT	TT	TT	CC	CC	CC	CC	CC	CC
GG	GG	GG	GG	GG	GG	GG	AA	AA
GG	GG	GG	AA	AA	AA	AA	GG	GG
GG	GG	GG	GG	GG	GG	GG	GG	GG
CC	CC	CC	CC	CC	CC	CC	TT	TT

IRIS_313-1	IRIS_313-1	IRIS_313-1	IRIS_313-1	IRIS_313-1	IRIS_313-1	IRIS_313-1	IRIS_313-1	IRIS_313-1
CC	CC	TT	TT	CC	TT	CC	CC	CC
GG	GG	GG	GG	GG	GG	GG	GG	GG
TT	TT	AA	AA	TT	AA	TT	TT	TT
CC	CC	CC	CC	CC	CC	CC	CC	CC
AA	AA	GG	GG	AA	GG	AA	AA	AA
GG	GG	AA	AA	GG	AA	GG	GG	GG
GG	GG	AA	GG	GG	GG	GG	GG	GG
TT	TT	CC	CC	TT	CC	TT	TT	TT

IRIS_313-1	IRIS_313-1	IRIS_313-1	IRIS_313-1	IRIS_313-1	IRIS_313-1	IRIS_313-1	IRIS_313-1	IRIS_313-1
TT	CC	TT	TT	TT	CC	TT	TT	CC
GG	GG	GG	GG	GG	GG	GG	GG	GG
AA	TT	AA	AA	AA	TT	AA	AA	TT
CC	CC	CC	CC	CC	CC	CC	CC	CC
GG	AA	GG	GG	GG	AA	GG	GG	AA
AA	GG	AA	AA	AA	GG	AA	AA	GG
GG	GG	GG	GG	GG	GG	GG	GG	GG
CC	TT	CC	CC	CC	TT	CC	CC	TT

IRIS_313-1	IRIS_313-1	IRIS_313-1	IRIS_313-1	IRIS_313-1	IRIS_313-1	IRIS_313-1	IRIS_313-1	IRIS_313-1
CC	TT	CC	TT	TT	TT	TT	TT	TT
GG	GG	GG	GG	GG	GG	GG	GG	GG
TT	AA	TT	AA	AA	AA	AA	AA	AA
CC	CC	CC	CC	CC	CC	CC	CC	CC
AA	GG	AA	GG	GG	GG	GG	GG	GG
GG	AA	GG	AA	AA	AA	AA	AA	AA
GG	GG	GG	GG	GG	GG	GG	AA	GG
TT	CC	TT	CC	CC	CC	CC	CC	CC

IRIS_313-1	IRIS_313-1	IRIS_313-1	IRIS_313-1	IRIS_313-1	IRIS_313-1	IRIS_313-1	IRIS_313-1	IRIS_313-1
CC	TT	TT	TT	TT	TT	CC	TT	CC
GG	GG	GG	GG	GG	GG	GG	GG	GG
TT	AA	AA	AA	AA	AA	TT	AA	TT
CC	CC	CC	CC	CC	CC	CC	CC	CC
AA	GG	GG	GG	GG	GG	AA	GG	AA
GG	AA	AA	AA	AA	AA	GG	AA	GG
NN	GG	AA	GG	GG	GG	GG	GG	GG
TT	CC	CC	CC	CC	CC	TT	CC	TT

IRIS_313-1	IRIS_313-1	IRIS_313-1	IRIS_313-1	IRIS_313-1	IRIS_313-1	IRIS_313-1	IRIS_313-1	IRIS_313-1
TT	TT	TT	TT	TT	TT	TT	CC	TT
GG	GG	GG	GG	GG	GG	GG	GG	GG
AA	AA	AA	AA	AA	AA	AA	TT	AA
CC	CC	CC	CC	CC	CC	CC	CC	CC
GG	GG	GG	GG	GG	GG	GG	AA	GG
AA	AG	AA	AA	AA	AA	AA	GG	AA
GG	GG	GG	GG	GG	GG	GG	GG	GG
CC	CC	CC	CC	CC	CC	CC	TT	CC

IRIS_313-1	IRIS_313-1	IRIS_313-1	IRIS_313-1	IRIS_313-1	IRIS_313-1	IRIS_313-1	IRIS_313-1	IRIS_313-1
TT	CC	TT	TT	TT	TT	TT	TT	TT
GG	GG	GG	GG	GG	GG	GG	GG	GG
AA	TT	AA	AA	AA	AA	AA	AA	AA
CC	CC	CC	CC	CC	CC	CC	CC	CC
GG	AA	GG	GG	GG	GG	GG	GG	GG
NN	GG	AA	AA	AA	AA	AA	NN	AA
NN	GG	GG	GG	GG	GG	NN	GG	GG
CC	TT	CC	CC	CC	CC	CC	CC	CC

IRIS_313-1	IRIS_313-1	IRIS_313-1	IRIS_313-1	IRIS_313-1	IRIS_313-1	IRIS_313-1	IRIS_313-1	IRIS_313-1
TT	CC	CC	TC	TT	TT	CC	CC	CC
GG	GG	GG	GG	GG	GG	GG	GG	GG
AA	TT	TT	AT	AA	AA	TT	TT	TT
CC	CC	CC	CC	CC	CC	CC	CC	CC
GG	AA	AA	GA	GG	GG	AA	AA	AA
AA	GG	GG	AG	AA	AA	GG	GG	GG
GG	GG	GG	GG	GG	GG	GG	GG	GG
CC	TT	TT	CT	CC	CC	TT	TT	TT

IRIS_313-1	IRIS_313-1	IRIS_313-1	IRIS_313-1	IRIS_313-1	IRIS_313-1	IRIS_313-1	IRIS_313-1	IRIS_313-1
CC	TT	TT	TT	TT	TT	TT	TT	CC
GG	GG	GG	GG	TT	GG	GG	GG	GG
TT	AA	AA	AA	AA	AA	AA	AA	TT
CC	CC	CC	CC	TT	CC	CC	CC	CC
AA	GG	GG	GG	GG	GG	GG	GG	AA
GG	AA	AA	AA	GG	AA	AA	AG	GG
GG	GG	GG	GG	GG	GG	GG	GG	GG
TT	CC	CC	CC	CC	CT	CC	CC	TT

IRIS_313-1	IRIS_313-1	IRIS_313-1	IRIS_313-1	IRIS_313-1	IRIS_313-1	IRIS_313-1	IRIS_313-1	IRIS_313-1
TT	TT	TT	TT	TT	TT	CC	CC	TT
GG	GG	GG	GG	GG	GG	GG	GG	GG
AA	AA	AA	AA	AA	AA	AA	TT	AA
CC	CC	CC	CC	CC	CC	CC	CC	CC
GG	GG	GG	GG	GG	GG	GG	AA	GG
AA	AA	AA	AA	AA	AA	GG	GG	NN
GG	GG	GG	GG	GG	GG	GG	GG	NN
CC	CC	CC	CC	CC	CC	CC	TT	CC

IRIS_313-1	IRIS_313-1	IRIS_313-1	IRIS_313-1	IRIS_313-1	IRIS_313-1	IRIS_313-1	IRIS_313-1	IRIS_313-1
CC	TT	TT	TT	TT	TT	TT	TT	CC
GG	GG	TT	GG	TT	GG	GG	GG	GG
TT	AA	AA	AT	AA	AA	AA	AA	TT
CC	CC	TT	CC	TT	CC	CC	CC	CC
AA	GG	GG	GG	GG	GG	GG	GG	AA
GG	AA	GG	AA	GG	AA	AA	AA	GG
GG	GG	GG	GG	GG	GG	GG	GG	GG
TT	CC	CC	CC	CC	CC	CC	CC	TT

IRIS_313-1	IRIS_313-1	IRIS_313-1	IRIS_313-1	IRIS_313-1	IRIS_313-1	IRIS_313-1	IRIS_313-1	IRIS_313-1
CC	TT	CC	CC	CC	CC	CC	TT	TT
GG	GG	GG	GG	GG	GG	GG	GG	GG
TT	AA	TT	TT	TT	TT	TT	AA	AA
CC	CC	CC	CC	CC	CC	CC	CC	CC
AA	GG	AA	AA	AA	AA	AA	GG	GG
GG	AA	GG	GG	GG	GG	GG	AA	AA
GG	GG	GG	GG	GG	NN	GG	GG	GG
TT	CC	TT	TT	TT	NN	TT	CC	CC

IRIS_313-1	IRIS_313-1	IRIS_313-1	IRIS_313-1	IRIS_313-1	IRIS_313-1	IRIS_313-1	IRIS_313-1	IRIS_313-1
TT	TT	CC	TT	TT	TT	TT	TT	CC
GG	GG	GG	GG	GG	GG	GG	GG	GG
AA	AA	TT	AA	AA	AA	AA	AA	TT
CC	CC	CC	CC	CC	CC	CC	CC	CC
GG	GG	AA	GG	GG	GG	GG	GG	AA
AA	AA	GG	AA	AA	AA	AA	AA	GG
GG	GG	GG	GG	GG	GG	GG	GG	GG
CC	CC	TT	CC	CC	CC	CC	CC	TT

IRIS_313-1	IRIS_313-1	IRIS_313-1	IRIS_313-1	IRIS_313-1	IRIS_313-1	IRIS_313-1	IRIS_313-1	IRIS_313-1
CC	TT	CC	TT	CC	CC	CC	TT	TT
GG	GG	GG	GG	GG	GG	GG	GG	GG
TT	AA	TT	AA	TT	TT	TT	AA	AA
CC	CC	CC	CC	CC	CC	CC	CC	CC
AA	GA	AA	GG	AA	AA	AA	GG	GG
GG	AA	GG	AA	GG	GG	GG	AA	AA
GG	GG	GG	GG	GG	GG	GG	GG	GG
TT	CC	TT	CC	TT	TT	TT	CC	CC

IRIS_313-1	IRIS_313-1	IRIS_313-1	IRIS_313-1	IRIS_313-1	IRIS_313-1	IRIS_313-1	IRIS_313-1	IRIS_313-1
CC	NN	TT	CC	TT	TT	CC	TT	TT
GG	GG	GG	GG	GG	GG	GG	GG	GG
TT	AT	AA	TT	AA	AA	TT	AA	AA
CC	CC	CC	CC	CC	CC	CC	CC	CC
AA	GA	GG	AA	GG	GG	AA	GG	GG
GG	AG	AA	GG	AA	AA	GG	AG	AA
GG	GG	GG	GG	GG	GG	GG	GG	GG
TT	TT	CC	TT	CC	CC	TT	CC	CC

IRIS_313-1	IRIS_313-1	IRIS_313-1	IRIS_313-1	IRIS_313-1	IRIS_313-1	IRIS_313-1	IRIS_313-1	IRIS_313-1
TT	CC	CC	TT	CC	CC	CC	TT	TC
GG	GG	GG	GG	GG	GG	GG	GG	GG
AA	TT	TT	AA	TT	TT	TT	AA	AT
CC	CC	CC	CC	CC	CC	CC	CC	CC
GG	AA	AA	GG	AA	AA	AA	GG	GA
AA	GG	GG	AA	GG	GG	GG	AA	AA
GG	GG	GG	GG	GG	GG	GG	GG	GG
CC	TT	TT	CC	TT	TT	TT	CC	NN

IRIS_313-1	IRIS_313-1	IRIS_313-1	IRIS_313-1	IRIS_313-1	IRIS_313-1	IRIS_313-1	IRIS_313-1	IRIS_313-1
NN	CC	CC	TT	TT	TT	CC	CC	TT
GG	GG	GG	GG	GG	GG	GG	GG	GG
NN	TT	TT	AA	AA	AA	TT	TT	AA
CC	CC	CC	CC	CC	CC	CC	CC	CC
AA	AA	AA	GG	GG	GG	AA	AA	GG
GG	GG	GG	AA	AA	AA	GG	GG	AA
GG	GG	GG	GG	GG	GG	GG	GG	GG
TT	TT	TT	CC	CC	CC	TT	TT	CC

IRIS_313-1	IRIS_313-1	IRIS_313-1	IRIS_313-1	IRIS_313-1	IRIS_313-1	IRIS_313-1	IRIS_313-1	IRIS_313-1
TT	CC	CC	CC	CC	CC	CC	TT	CC
GG	GG	GG	GG	GG	GG	GG	GG	GG
AA	TT	TT	TT	TT	TT	TT	AA	TT
CC	CC	CC	CC	CC	CC	CC	CC	CC
GG	AA	AA	AA	AA	AA	AA	GG	AA
AA	GG	GG	GG	GG	GG	GG	AA	GG
GG	GG	GG	GG	GG	GG	GG	GG	GG
CC	TT	TT	TT	TT	TT	TT	CC	TT

IRIS_313-1	IRIS_313-1	IRIS_313-1	IRIS_313-1	IRIS_313-1	IRIS_313-1	IRIS_313-1	IRIS_313-1	IRIS_313-1
TT	TT	CC	CC	TT	TT	TT	TT	TT
GG	GG	GG	GG	GG	GG	GG	GG	GG
AA	AA	TT	TT	AA	AA	AA	AA	AA
CC	CC	CC	CC	CC	CC	CC	CC	CC
GG	GG	AA	AA	GG	GG	GG	GG	GG
AA	AA	GG	GG	AA	AA	AA	AA	AA
GG	NN	GG	GG	GG	GG	GG	GG	GG
CC	CC	TT	TT	CC	CC	CC	CC	CC

IRIS_313-1	IRIS_313-1	IRIS_313-1	IRIS_313-1	IRIS_313-1	IRIS_313-1	IRIS_313-1	IRIS_313-1	IRIS_313-1
CC	CC	CC	TT	TT	TT	TT	TT	TT
GG	GG	GG	GG	GG	GG	GG	GG	GG
TT	TT	TT	AA	AA	AA	AA	AA	AA
CC	CC	CC	CC	CC	CC	CC	CC	CC
AA	AA	AA	GG	GG	GG	GG	GG	GG
GG	GG	GG	AA	NN	AA	AA	AA	AA
GG	GG	GG	GG	GG	GG	GG	GG	GG
TT	TT	TT	CC	CC	CC	CC	CC	CC

IRIS_313-1	IRIS_313-1	IRIS_313-1	IRIS_313-1	IRIS_313-1	IRIS_313-1	IRIS_313-1	IRIS_313-1	IRIS_313-1
TT	TT	CC	CC	CC	CC	CC	CC	TT
GG	GG	GG	GG	GG	GG	GG	GG	GG
AA	AA	TT	TT	TT	TT	TT	TT	AA
CC	CC	CC	CC	CC	CC	CC	CC	CC
GG	GG	AA	AA	AA	AA	AA	AA	GG
AA	AA	GG	GG	GG	GG	GG	GG	NN
GG	GG	GG	GG	GG	GG	GG	GG	GG
CC	CC	TT	TT	TT	TT	TT	TT	CC

IRIS_313-1	IRIS_313-1	IRIS_313-1	IRIS_313-1	IRIS_313-1	IRIS_313-1	IRIS_313-1	IRIS_313-1	IRIS_313-1
CC	TT	TT	CC	CC	CC	CC	CC	TT
GG	GG	GG	GG	GG	GG	GG	GG	GG
TT	AA	AA	TT	TT	TT	TT	TT	AA
CC	CC	CC	CC	CC	CC	CC	CC	CC
AA	GG	GG	AA	AA	AA	AA	AA	GG
GG	AA	AA	GG	GG	GG	GG	GG	AA
GG	GG	NN	GG	GG	GG	GG	GG	GG
TT	CC	CC	TT	TT	TT	TT	TT	CC

IRIS_313-1	IRIS_313-1	IRIS_313-1	IRIS_313-1	IRIS_313-1	IRIS_313-1	IRIS_313-1	IRIS_313-1	IRIS_313-1
TT	TT	TT	CC	TT	TC	CC	TT	CC
GG	TT	GG	GG	TT	GG	GG	TT	GG
AA	AA	AA	TT	AA	AT	TT	AA	TT
CC	TT	CC	CC	TT	CC	CC	TT	CC
GG	GG	GG	AA	GG	GA	AA	GG	AA
AA	GG	AA	GG	GG	AG	GG	GG	GG
GG	GG	GG	GG	GG	GG	GG	GG	GG
CC	CC	CC	TT	CC	CT	TT	CC	TT

IRIS_313-1	IRIS_313-1	IRIS_313-1	IRIS_313-1	IRIS_313-1	IRIS_313-1	IRIS_313-1	IRIS_313-1	IRIS_313-1
TT	TT	TT	TT	TT	TT	TT	CC	TT
GG	GG	GG	GG	GG	GG	GG	GG	GG
AA	AA	AA	AA	AA	AA	AA	TT	AA
CC	CC	CC	CC	CC	CC	CC	CC	CC
GG	GG	GG	GG	GG	GG	GG	AA	GG
AA	AA	AA	AA	AA	AA	AA	GG	AA
GG	GG	GG	GG	GG	GG	GG	GG	GG
NN	CC	CC	CC	CC	CC	CC	TT	CC

IRIS_313-1	IRIS_313-1	IRIS_313-1	IRIS_313-1	IRIS_313-1	IRIS_313-1	IRIS_313-1	IRIS_313-1	IRIS_313-1
CC	CC	CC	CC	CC	TT	CC	TT	TC
GG	GG	GG	NN	GG	GG	GG	GG	GG
TT	TT	TT	TT	TT	AA	TT	AA	AT
CC	CC	CC	CC	CC	CC	CC	CC	CC
AA	AA	AA	AA	AA	GG	AA	GG	GA
GG	GG	GG	GG	GG	AA	GG	AA	NN
GG	GG	GG	GG	GG	GG	GG	GG	GG
TT	TT	TT	TT	TT	CC	TT	CC	CT

IRIS_313-1	IRIS_313-1	IRIS_313-1	IRIS_313-1	IRIS_313-1	IRIS_313-1	IRIS_313-1	IRIS_313-1	IRIS_313-1
TT	CC	TT	TT	TT	TT	TT	TT	TT
GG	GG	TT	GG	GG	GG	GG	GG	GG
AA	TT	AA	AA	AA	AA	AA	AA	AA
CC	CC	TT	CC	CC	CC	CC	CC	CC
GG	AA	GG	GG	GG	GG	GG	GG	GG
AA	GG	GG	AA	AA	AA	AA	AA	AA
GG	GG	GG	GG	GG	GG	GG	GG	GG
CC	TT	CC	CC	CC	CC	CC	CC	CC

IRIS_313-1	IRIS_313-1	IRIS_313-1	IRIS_313-1	IRIS_313-1	IRIS_313-1	IRIS_313-1	IRIS_313-1	IRIS_313-1
TT	TT	CC	TT	CC	TT	CC	CC	CC
GG	GG	GG	GG	GG	GG	GG	GG	GG
AA	AA	TT	AA	TT	AA	TT	TT	TT
CC	CC	CC	CC	CC	CC	CC	CC	CC
GG	GG	AA	GG	AA	GG	AA	AA	AA
AA	AA	GG	AA	GG	AA	GG	GG	GG
GG	GG	GG	GG	GG	GG	GG	GG	GG
CC	CC	TT	CC	TT	CC	TT	TT	TT

IRIS_313-1	IRIS_313-1	IRIS_313-1	IRIS_313-1	IRIS_313-1	IRIS_313-1	IRIS_313-1	IRIS_313-1	IRIS_313-1
TT	TT	CC	TT	CC	TT	TT	TT	TT
GG	GG	GG	GG	GG	GG	GG	GG	GG
AA	AA	TT	AA	TT	AA	AA	AA	AA
CC	CC	CC	CC	CC	CC	CC	CC	CC
GG	GG	AA	GG	AA	GG	GG	GG	GG
AA	AA	GG	AA	NN	AA	AA	AA	AA
GG	GG	GG	GG	GG	GG	GG	GG	GG
CC	CC	TT	CC	TT	CC	CC	CC	CC

IRIS_313-1	IRIS_313-1	IRIS_313-1	IRIS_313-1	IRIS_313-1	IRIS_313-1	IRIS_313-1	IRIS_313-1	IRIS_313-1
TT	TT	TT	TT	TT	TT	TT	TT	TT
GG	GG	GG	GG	GG	GG	GG	GG	GG
AA	AA	AA	AA	AA	AA	AA	AA	AA
CC	CC	CC	CC	CC	CC	CC	NN	CC
GG	GG	GG	GG	GG	GG	GG	GG	GG
AA	AA	AA	AA	AA	AA	AA	NN	AA
GG	GG	GG	GG	GG	GG	GG	GG	GG
CC	CC	CC	CC	CC	CC	CC	CC	CC

IRIS_313-1	IRIS_313-1	IRIS_313-1	IRIS_313-1	IRIS_313-1	IRIS_313-1	IRIS_313-1	IRIS_313-1	IRIS_313-1
TT	TT	TT	TT	TT	TT	CC	TT	TC
GG	GG	GG	GG	GG	GG	GG	GG	GG
AA	AA	AA	AA	AA	AA	TT	AA	AT
CC	CC	CC	CC	CC	CC	CC	CC	CC
GG	GG	GG	GG	GG	GG	AA	GG	GA
AA	AA	NN	AA	AA	AA	GG	AA	AG
GG	GG	GG	GG	GG	GG	GG	GG	GG
CC	CC	CC	CC	CC	CC	TT	CC	CT

IRIS_313-1	IRIS_313-1	IRIS_313-1	IRIS_313-1	IRIS_313-1	IRIS_313-1	IRIS_313-1	IRIS_313-1	IRIS_313-1
CC	CC	TT	CC	CC	TT	TT	CC	TT
GG	GG	GG	GG	GG	GG	GG	GG	TT
TT	TT	AA	TT	TT	AA	AA	TT	AA
CC	CC	CC	CC	CC	CC	CC	CC	TT
AA	AA	GG	AA	AA	GG	GG	AA	GG
GG	GG	AA	GG	NN	AA	AA	GG	GG
GG	GG	GG	GG	GG	GG	GG	GG	GG
TT	TT	CC	TT	TT	CC	CC	TT	CC

IRIS_313-1	IRIS_313-1	IRIS_313-1	IRIS_313-1	IRIS_313-1	IRIS_313-1	IRIS_313-1	IRIS_313-1	IRIS_313-1
TT	TT	TT	CC	TT	CC	TT	TT	CC
GG	GG	TT	GG	GG	GG	GG	GG	GG
AA	AA	AA	TT	AA	TT	AA	AA	TT
CC	CC	TT	CC	CC	CC	CC	CC	CC
GG	GG	GG	AA	GG	AA	GG	GG	AA
AA	AA	GG	GG	AA	GG	AA	AA	GG
GG	GG	GG	GG	GG	GG	GG	GG	GG
CC	CC	CC	TT	CC	TT	NN	CC	TT

IRIS_313-1	IRIS_313-1	IRIS_313-1	IRIS_313-1	IRIS_313-1	IRIS_313-1	IRIS_313-1	IRIS_313-1	IRIS_313-1
TT	TT	TT	TT	TT	TT	TT	CC	CC
GG	GG	GG	GG	GG	GG	GG	GG	GG
AA	AA	AA	AA	AA	AA	AA	TT	TT
CC	CC	CC	CC	CC	CC	CC	CC	CC
GG	GG	GG	GG	GG	GG	GG	AA	AA
AA	AA	AA	AA	AA	AA	AA	GG	GG
GG	GG	GG	GG	GG	GG	GG	GG	GG
CC	CC	CC	CC	CC	CC	CC	TT	TT

IRIS_313-1	IRIS_313-1	IRIS_313-1	IRIS_313-1	IRIS_313-1	IRIS_313-1	IRIS_313-1	IRIS_313-1	IRIS_313-1
TT	TC	CC	CC	TT	CC	CC	TT	TT
GG	GG	GG	GG	GG	GG	GG	GG	GG
AA	AT	TT	TT	AA	TT	TT	AA	AA
CC	CC	CC	CC	CC	CC	CC	CC	CC
GG	GA	AA	AA	GG	AA	AA	GA	GG
AA	AG	GG	GG	AA	GG	GG	AA	AA
GG	GG	GG	GG	GG	GG	GG	GG	GG
CC	CT	TT	TT	CC	TT	TT	CC	NN

IRIS_313-1	IRIS_313-1	IRIS_313-1	IRIS_313-1	IRIS_313-1	IRIS_313-1	IRIS_313-1	IRIS_313-1	IRIS_313-1
CC	CC	CC	CC	CC	TT	TT	TT	TT
GG	GG	GG	GG	GG	GG	GG	GG	GT
TT	TT	TT	TT	TT	AA	AA	AA	AA
CC	CC	CC	CC	CC	CC	CC	CC	TT
AA	AA	AA	AA	AA	GG	GG	GG	GG
GG	GG	GG	GG	GG	AA	AA	AA	GG
GG	GG	GG	GG	GG	GG	GG	GG	GG
TT	TT	TT	TT	TT	CC	CC	CC	CC

IRIS_313-1	IRIS_313-1	IRIS_313-1	IRIS_313-1	IRIS_313-1	IRIS_313-1	IRIS_313-1	IRIS_313-1	IRIS_313-1
TT	TT	CC	TT	TT	TT	TT	NN	TT
GG	GG	GG	GG	GG	GG	GG	GG	GG
AA	AA	TT	AA	AA	AA	AA	AA	AA
CC	CC	CC	CC	CC	CC	CC	CC	CC
GG	GG	AA	GG	GG	GG	GG	GG	GG
AA	AA	GG	AA	AA	NN	NN	AA	AA
AA	GG	GG	GG	GG	GG	NN	GG	GG
CC	CC	TT	CC	CC	CC	CC	CC	CC

IRIS_313-1	IRIS_313-1	IRIS_313-1	IRIS_313-1	IRIS_313-1	IRIS_313-1	IRIS_313-1	IRIS_313-1	IRIS_313-1
TC	TT	TT	TT	TT	TT	TT	TT	TT
GG	GG	TT	GG	GG	TT	GT	TT	GG
AA	AA	AA	AA	AA	AA	AA	AA	AA
CC	CC	TT	CC	CC	TT	CT	TT	CC
GG	GG	GG	GG	GG	GG	GG	GG	GG
AA	AA	GG	AA	AA	GG	NN	GG	AA
GG	GG	GG	GG	GG	GG	GG	GG	GG
CC	CC	CC	CC	CC	CC	CC	CC	CC

IRIS_313-1	IRIS_313-1	IRIS_313-1	IRIS_313-1	IRIS_313-1	IRIS_313-1	IRIS_313-1	IRIS_313-1	IRIS_313-1
TT	TT	TT	TT	TT	TT	TT	TT	CC
GG	GG	GG	GG	TT	GG	GG	GG	GG
AA	AA	AA	AA	AA	AA	AA	AA	TT
CC	CC	CC	CC	TT	CC	CC	CC	CC
GG	GG	GG	GG	GG	GG	GG	GG	AA
AA	AA	AA	AA	GG	AA	AA	AA	GG
GG	GG	GG	GG	GG	GG	GG	GG	GG
CC	CC	CC	CC	CC	CC	CC	CC	TT

IRIS_313-1	IRIS_313-1	IRIS_313-1	IRIS_313-1	IRIS_313-1	IRIS_313-1	IRIS_313-1	IRIS_313-1	IRIS_313-1
CC	CC	CC	TT	TT	TT	TT	CC	TT
GG	GG	GG	GG	GG	GG	GG	GG	GG
TT	TT	TT	AA	AA	AA	AA	TT	AA
CC	CC	CC	CC	CC	CC	CC	CC	CC
AA	AA	AA	GG	GG	GG	GG	AA	GG
GG	GG	GG	AA	AA	AA	NN	GG	AA
GG	GG	GG	GG	GG	GG	GG	GG	GG
TT	TT	TT	CC	CC	CC	CC	TT	CC

IRIS_313-1	IRIS_313-1	IRIS_313-1	IRIS_313-1	IRIS_313-1	IRIS_313-1	IRIS_313-1	IRIS_313-1	IRIS_313-1
CC	CC	CC	CC	TT	CC	CC	CC	TT
GG	GG	GG	GG	GG	GG	GG	GG	GG
TT	TT	TT	TT	AA	TT	TT	TT	AA
CC	CC	CC	CC	CC	CC	CC	CC	CC
AA	AA	AA	AA	GG	AA	AA	AA	GG
GG	GG	GG	GG	AA	GG	GG	GG	AA
GG	GG	GG	GG	GG	GG	GG	GG	GG
TT	TT	TT	TT	CC	TT	TT	TT	CC

IRIS_313-1	IRIS_313-1	IRIS_313-1	IRIS_313-1	IRIS_313-1	IRIS_313-1	IRIS_313-1	IRIS_313-1	IRIS_313-1
TT	TT	TT	TT	TT	TT	TT	TT	TT
GG	GG	TT	TT	TT	TT	TT	TT	TT
AA	AA	AA	AA	AA	AA	AA	AA	AA
CC	CC	TT	CT	TT	TT	TT	TT	TT
GG	GG	GG	GG	GG	GG	GG	GG	GG
AA	NN	GG	GG	GG	GG	GG	GG	GG
GG	NN	GG	GG	GG	GG	GG	GG	GG
CC	NN	CC	CC	CC	CC	CC	CC	CC

IRIS_313-1	IRIS_313-1	IRIS_313-1	IRIS_313-1	IRIS_313-1	IRIS_313-1	IRIS_313-1	IRIS_313-1	IRIS_313-1
TT	TT	TT	TT	TT	TT	CC	TT	TT
GG	TT	TT	TT	GG	TT	GG	GG	GG
AA	AA	AA	AA	AA	AA	TT	AA	AA
CC	TT	TT	TT	CC	TT	CC	CC	CC
GG	GG	GG	GG	GG	GG	AA	GG	GG
AA	GG	GG	GG	AA	NN	GG	AA	AA
GG	GG	GG	GG	GG	NN	GG	GG	GG
CC	CC	CC	CC	CC	CC	TT	CC	CC

IRIS_313-1	IRIS_313-1	IRIS_313-1	IRIS_313-1	IRIS_313-1	IRIS_313-1	IRIS_313-1	IRIS_313-1	IRIS_313-1
TT	CC	TT	TT	TC	TT	TT	TT	TT
TT	GG	GG	TT	GG	GG	GG	GG	GG
AA	TT	AA	AA	AT	AA	AA	AA	AA
TT	CC	CC	TT	CC	CC	CC	CC	CC
GG	AA	GG	GG	GA	GG	GG	GG	GG
GG	GG	NN	GG	AG	AA	AA	AA	AA
GG	GG	NN	GG	GG	GG	GG	GG	GG
CC	TT	CC	CC	CT	CC	CC	CC	CC

IRIS_313-1	IRIS_313-1	IRIS_313-1	IRIS_313-1	IRIS_313-1	IRIS_313-1	IRIS_313-1	IRIS_313-1	IRIS_313-1
TT	TT	TT	TT	TT	TT	CC	CC	CC
GG	GG	GG	GG	GG	GG	GG	GG	GG
AA	AA	AA	AA	AA	AA	TT	TT	TT
CC	CC	CC	CC	CC	CC	CC	CC	CC
GG	GG	GG	GG	GG	GG	AA	AA	AA
AA	AA	AA	AA	AA	AA	GG	GG	GG
GG	GG	GG	GG	GG	GG	GG	GG	GG
CC	CC	CC	CC	CC	CC	TT	TT	TT

IRIS_313-1	IRIS_313-1	IRIS_313-1	IRIS_313-1	IRIS_313-1	IRIS_313-1	IRIS_313-1	IRIS_313-1	IRIS_313-1
TT	TT	TT	TT	TT	TT	TT	TT	TT
GG	TT	TT	GG	TT	GG	TT	TT	GT
AA	AA	AA	AA	AA	AA	AA	AA	AA
CC	TT	TT	CC	TT	CC	TT	TT	CT
GG	GG	GG	GG	GG	GG	GG	GG	GG
AA	GG	GG	AA	GG	AA	GG	GG	AG
NN	GG	GG	AA	NN	AA	GG	GG	GA
CC	CC	CC	CC	CC	CC	CC	CC	CC

IRIS_313-1	IRIS_313-1	IRIS_313-1	IRIS_313-1	IRIS_313-1	IRIS_313-1	IRIS_313-1	IRIS_313-1	IRIS_313-1
TT	TT	TT	TT	TT	TT	TT	TT	TT
TT	TT	GG	GG	TT	GG	TT	TT	TT
AA	AA	AA	AA	AA	AA	AA	AA	AA
TT	TT	CC	CC	TT	CC	TT	TT	TT
GG	GG	GG	GG	GG	GG	GG	GG	GG
GG	GG	AA	AA	GG	AA	GG	GG	GG
GG	GG	AA	AA	GG	GG	GG	GG	GG
CC	CC	CC	CC	CC	CC	CC	CC	CC

IRIS_313-1	IRIS_313-1	IRIS_313-1	IRIS_313-1	IRIS_313-1	IRIS_313-1	IRIS_313-1	IRIS_313-1	IRIS_313-1
TT	TT	TT	TT	TT	TT	TT	TT	CC
TT	TT	TT	TT	TT	TT	GG	GG	GG
AA	AA	AA	AA	AA	AA	AA	AA	TT
TT	TT	TT	TT	TT	TT	CC	CC	CC
GG	GG	GG	GG	GG	GG	GG	GG	AA
GG	GG	GG	GG	GG	GG	NN	AA	GG
GG	GG	GG	GG	GG	GG	GG	GG	GG
CC	CC	CC	CC	CC	CC	CC	CC	TT

IRIS_313-1	IRIS_313-1	IRIS_313-1	IRIS_313-1	IRIS_313-1	IRIS_313-1	IRIS_313-1	IRIS_313-1	IRIS_313-1
TT	CC	TT	CC	TT	TT	TT	TT	TT
GG	GG	GG	GG	GG	GG	GG	GG	GG
AA	TT	AA	TT	AA	AA	AA	AA	AA
CC	CC	CC	CC	CC	CC	CC	CC	CC
GG	AA	GG	AA	GG	GG	GG	GG	GG
AA	GG	AA	GG	AA	AA	AA	AA	AA
GG	GG	GG	GG	GG	GG	GG	GG	GG
CC	TT	CC	TT	CC	CC	CC	CC	CC

IRIS_313-1	IRIS_313-1	IRIS_313-1	IRIS_313-1	IRIS_313-1	IRIS_313-1	IRIS_313-1	IRIS_313-1	IRIS_313-1
TT	TT	TT	TT	TT	TT	TT	TT	TT
GG	GG	GG	GG	GG	GG	GG	GG	GG
AA	AA	AA	AA	AA	AA	AA	AA	AA
CC	CC	CC	CC	CC	CC	CC	CC	CC
GG	GG	GG	GG	GG	GG	GG	GG	GG
AA	AA	AA	AA	AA	AA	AA	AA	AA
GG	GG	GG	GG	GG	GG	GG	GG	GG
CC	CC	CC	CC	CC	CC	CC	CC	CC

IRIS_313-1	IRIS_313-1	IRIS_313-1	IRIS_313-1	IRIS_313-1	IRIS_313-1	IRIS_313-1	IRIS_313-1	IRIS_313-1
TC	CC	CC	TC	TT	TT	TT	TT	CC
GG	GG	GG	GG	GG	GG	GG	GG	GG
NN	TT	TT	AT	AA	AA	AA	AA	TT
CC	CC	CC	CC	CC	CC	CC	CC	CC
NN	AA	AA	GA	GG	GG	GG	GG	AA
AG	GG	GG	AG	AA	AA	AA	AA	GG
GG	GG	GG	GG	GG	GG	GG	GG	GG
CT	TT	TT	CT	CC	CC	CC	CC	TT

IRIS_313-1	IRIS_313-1	IRIS_313-1	IRIS_313-1	IRIS_313-1	IRIS_313-1	IRIS_313-1	IRIS_313-1	IRIS_313-1
TT	CC	CC	CC	CC	CC	CC	TT	CC
GG	GG	GG	GG	GG	GG	GG	GG	GG
AA	TT	TT	TT	TT	TT	TT	AA	TT
CC	CC	CC	CC	CC	CC	CC	CC	CC
GG	AA	AA	AA	AA	AA	NN	GG	AA
AA	GG	GG	GG	GG	GG	GG	AA	GG
GG	GG	GG	GG	GG	GG	GG	GG	GG
CC	TT	TT	TT	TT	TT	TT	CC	TT

IRIS_313-1	IRIS_313-1	IRIS_313-1	IRIS_313-1	IRIS_313-1	IRIS_313-1	IRIS_313-1	IRIS_313-1	IRIS_313-1
TT	TT	TT	TT	TT	TT	TT	CC	TT
GG	GG	TT	GT	GG	GG	TT	GG	GG
AA	AA	AA	AA	AA	AA	AA	TT	AA
CC	CC	TT	CT	CC	CC	TT	CC	CC
GG	GG	GG	GG	GG	GG	GG	AA	GG
AA	AA	GG	AG	AA	AA	GG	GG	AA
GG	AA	GG	GA	GG	GG	NN	GG	GG
CC	CC	CC	CC	CC	CC	CC	TT	CC

IRIS_313-1	IRIS_313-1	IRIS_313-1	IRIS_313-1	IRIS_313-1	IRIS_313-1	IRIS_313-1	IRIS_313-1	IRIS_313-1
TT	TT	CC	TT	TT	TT	TT	TT	CC
GG	GG	GG	GG	GG	GG	GG	GG	GG
AA	AA	TT	AA	AA	AA	AA	AA	TT
CC	CC	CC	CC	CC	CC	CC	CC	CC
GG	GG	AA	GG	GG	GG	GG	GG	AA
NN	AA	GG	AA	AA	AA	AA	AA	GG
NN	GG	GG	GG	AA	NN	GG	GG	GG
CC	CC	TT	CC	CC	CC	NN	CC	TT

IRIS_313-1	IRIS_313-1	IRIS_313-1	IRIS_313-1	IRIS_313-1	IRIS_313-1	IRIS_313-1	IRIS_313-1	IRIS_313-1
TT	TT	TT	TT	TT	TT	TT	TT	TT
GG	GG	GG	GG	GG	GG	GG	GG	GG
AA	AA	AA	AA	AA	AA	AA	AA	AA
CC	CC	CC	CC	CC	CC	CC	CC	CC
GG	GG	GG	GG	GG	GG	GG	GG	GG
AA	AA	NN	AA	AA	NN	AA	AA	AA
GG	GG	NN	GG	GG	GG	GG	GG	NN
CC	CC	CC	CC	CC	CC	CC	NN	CC

IRIS_313-1	IRIS_313-1	IRIS_313-1	IRIS_313-1	IRIS_313-1	IRIS_313-1	IRIS_313-1	IRIS_313-1	IRIS_313-1
TT	TT	TT	TT	TT	TT	TT	TT	TT
GG	GG	GG	GG	GG	GG	GG	GG	GG
AA	AA	AA	AA	AA	AA	AA	AA	AA
CC	CC	CC	CC	CC	CC	CC	CC	CC
GG	GG	GG	GG	GG	GG	GG	GG	GG
AA	NN	NN	AA	AA	AA	AA	AA	AA
GG	GG	GG	GG	GG	GG	GG	GG	GG
CC	NN	CC	CC	NN	CC	CC	NN	CC

IRIS_313-1	IRIS_313-1	IRIS_313-1	IRIS_313-1	IRIS_313-1	IRIS_313-1	IRIS_313-1	IRIS_313-1	IRIS_313-1
TT	TT	TT	TT	TT	TT	TT	CC	TT
GG	GG	GG	GG	GG	GG	GG	GG	GG
AA	AA	AA	AA	AA	AA	AA	NN	AA
CC	CC	CC	CC	CC	CC	CC	CC	CC
GG	GG	GG	GG	GG	GG	GG	NN	GG
AA	AA	AA	AA	AA	AA	AA	GG	NN
GG	GG	GG	GG	GG	GG	GG	GG	AA
NN	CC	CC	CC	CC	CC	CC	TT	CC

IRIS_313-1	IRIS_313-1	IRIS_313-1	IRIS_313-1	IRIS_313-1	IRIS_313-1	IRIS_313-1	IRIS_313-1	IRIS_313-1
CC	CC	TT	TT	CC	TT	CC	TT	TT
GG	GG	GG	GG	GG	GG	GG	GG	TT
TT	TT	AA	AA	TT	AA	TT	AA	AA
CC	CC	CC	CC	CC	CC	CC	CC	TT
AA	AA	GG	GG	AA	GG	AA	GG	GG
GG	GG	AA	NN	GG	AA	GG	AA	GG
GG	GG	GG	GG	NN	GG	GG	GG	GG
TT	TT	CC	CC	NN	CC	TT	CC	CC

IRIS_313-1	IRIS_313-1	IRIS_313-1	IRIS_313-1	IRIS_313-1	IRIS_313-1	IRIS_313-1	IRIS_313-1	IRIS_313-1
TT	TT	TT	TT	TT	TT	TT	TT	TT
TT	GG	TT	GG	GG	TT	GG	GG	GG
AA	AA	AA	AA	AA	AA	AA	AA	AA
TT	CC	TT	CC	CC	TT	CC	CC	CC
GG	GG	GG	GG	GG	GG	GG	GG	GG
GG	AA	GG	NN	NN	GG	AA	AA	AA
GG	GG	GG	NN	GG	GG	AA	AA	AA
CC	CC	CC	CC	NN	NN	CC	CC	CC

IRIS_313-1	IRIS_313-1	IRIS_313-1	IRIS_313-1	IRIS_313-1	IRIS_313-1	IRIS_313-1	IRIS_313-1	IRIS_313-1
TT	TT	TT	TT	TT	TT	TT	TT	TT
TT	GG	GG	GG	GG	GG	GG	GG	GG
AA	AA	AA	AA	AT	AA	AA	AA	AA
TT	CC	CC	CC	CC	CC	CC	CC	CC
GG	GG	GG	GG	GG	GG	GG	GG	GG
GG	AA	NN	AA	AA	AA	AA	AA	AA
GG	GG	GG	GG	GG	GG	GG	GG	GG
NN	CC	NN	CC	NN	CC	CC	CC	CC

IRIS_313-1	IRIS_313-1	IRIS_313-1	IRIS_313-1	IRIS_313-1	IRIS_313-1	IRIS_313-1	IRIS_313-1	IRIS_313-1
TT	CC	TT	TT	TT	TT	TT	CC	TT
GG	GG	GG	TT	GG	GG	GG	GG	GG
AA	TT	AA	AA	AA	AA	AA	TT	AA
CC	CC	CC	TT	CC	CC	CC	CC	CC
GG	AA	GG	GG	GG	GG	GG	AA	GG
AA	GG	AA	GG	NN	NN	AA	GG	AA
GG	GG	GG	GG	GG	NN	GG	GG	GG
CC	TT	CC	CC	CC	CC	CC	TT	CC

IRIS_313-1	IRIS_313-1	IRIS_313-1	IRIS_313-1	IRIS_313-1	IRIS_313-1	IRIS_313-1	IRIS_313-1	IRIS_313-1
TT	CC	TT	TT	CC	CC	TT	TT	TT
GG	GG	GG	GG	GG	GG	GG	GG	GG
AA	TT	AA	AA	TT	TT	AA	AA	AA
CC	CC	CC	CC	CC	CC	CC	CC	CC
GG	AA	GG	GG	AA	AA	GG	GG	GG
AA	GG	AA	AA	GG	GG	AA	AA	AA
GG	GG	GG	GG	GG	GG	GG	GG	GG
CC	TT	CC	CC	TT	TT	CC	CC	CC

IRIS_313-1	IRIS_313-1	IRIS_313-1	IRIS_313-1	IRIS_313-1	IRIS_313-1	IRIS_313-1	IRIS_313-1	IRIS_313-1
TT	TT	TT	TT	TT	TT	TT	TT	TT
GG	GG	GG	TT	GG	GG	GG	TT	TT
AA	AA	AA	AA	AA	AA	AA	AA	AA
CC	CC	CC	TT	CC	CC	CC	TT	TT
GG	GG	GG	GG	GG	GG	GG	GG	GG
AA	NN	AA	GG	AA	AA	AA	GG	GG
GG	GG	GG	GG	AA	GG	GG	GG	GG
CC	CC	CC	CC	CC	CC	CC	CC	CC

IRIS_313-1	IRIS_313-1	IRIS_313-1	IRIS_313-1	IRIS_313-1	IRIS_313-1	IRIS_313-1	IRIS_313-1	IRIS_313-1
TT	CC	CC	TT	TT	TT	TT	CC	TT
TT	GG	GG	GG	GG	GG	GG	GG	GG
AA	TT	TT	AA	AA	AA	AA	AT	AA
TT	CC	CC	CC	CC	CC	CC	CC	CC
GG	AA	AA	GG	GG	GG	GG	GA	GG
GG	GG	GG	AA	AA	AA	AA	GG	AA
GG	GG	GG	GG	GG	GG	GG	GG	GG
CC	TT	TT	CC	CC	CC	CC	CT	CC

IRIS_313-1	IRIS_313-1	IRIS_313-1	IRIS_313-1	IRIS_313-1	IRIS_313-1	IRIS_313-1	IRIS_313-1	IRIS_313-1
TT	TT	TT	TT	TT	TT	TT	TT	TT
GG	GG	GG	GG	GG	GG	GG	TT	GG
AA	AA	AA	AA	AA	AA	AA	AA	AA
CC	CC	CC	CC	CC	CC	CC	TT	CC
GG	GG	GG	GG	GG	GG	GG	GG	GG
AA	AA	AA	AA	AA	AA	AA	GG	AA
GG	GG	GG	GG	GG	GG	GG	GG	NN
CC	CC	CC	CC	CC	CC	CC	CC	CC

IRIS_313-1	IRIS_313-1	IRIS_313-1	IRIS_313-1	IRIS_313-1	IRIS_313-1	IRIS_313-1	IRIS_313-1	IRIS_313-1
TT	TT	TT	TT	CC	TT	TT	TT	TT
GG	GG	GG	GG	GG	GG	GG	GG	GG
AA	AA	AA	AA	TT	AA	AA	AA	AA
CC	CC	CC	CC	CC	CC	CC	CC	CC
GG	GG	GG	GG	AA	GG	GG	GG	GG
AA	AA	NN	AA	GG	AA	AA	AA	AA
GG	GG	GG	GG	GG	GG	GG	GG	GA
CC	CC	CC	CC	TT	CC	CC	CC	CC

IRIS_313-1	IRIS_313-1	IRIS_313-1	IRIS_313-1	IRIS_313-1	IRIS_313-1	IRIS_313-1	IRIS_313-1	IRIS_313-1
TT	TT	TT	CC	TT	CC	TT	TT	TT
GG	GG	GG	GG	GG	GG	GG	GG	GG
AA	AA	AA	TT	AA	TT	AA	AA	AA
CC	CC	CC	CC	CC	CC	CC	CC	CC
GG	GG	GG	AA	GG	AA	GG	GG	GG
AA	AA	AA	GG	AA	GG	AA	AA	AA
GG	GG	GG	GG	GG	GG	GG	GG	GG
CC	CC	CC	TT	CC	TT	CC	CC	CC

IRIS_313-1	IRIS_313-1	IRIS_313-1	IRIS_313-1	IRIS_313-1	IRIS_313-1	IRIS_313-1	IRIS_313-1	IRIS_313-1
TT	TT	TT	TT	TT	TT	CC	CC	TT
GG	GG	GG	GG	GG	GG	GG	GG	GG
AA	AA	AA	AA	AA	AA	TT	TT	AA
CC	CC	CC	CC	CC	CC	CC	CC	CC
GG	GG	GG	GG	GG	GG	AA	AA	GG
AA	AA	AA	AA	AA	AA	GG	GG	AA
GG	GG	GG	GG	GG	GG	NN	GG	GG
CC	CC	CC	NN	CC	CC	TT	TT	CC

IRIS_313-1	IRIS_313-1	IRIS_313-1	IRIS_313-1	IRIS_313-1	IRIS_313-1	IRIS_313-1	IRIS_313-1	IRIS_313-1
TT	TT	TT	TT	TT	TT	TT	CC	TC
GG	GG	GG	GG	TT	GG	GG	GG	GG
AA	AA	AA	AA	AA	AA	AA	TT	AT
NN	CC	CC	CC	TT	CC	CC	CC	CC
GA	GG	GG	GG	GG	GG	GG	AA	GA
AA	AA	AA	AA	GG	AA	AA	GG	AG
GG	GG	GG	GG	GG	GG	GG	GG	GG
CC	CC	CC	CC	CC	CC	CC	TT	CT

IRIS_313-1	IRIS_313-1	IRIS_313-1	IRIS_313-1	IRIS_313-1	IRIS_313-1	IRIS_313-1	IRIS_313-1	IRIS_313-1
CC	TT	TT	TT	TT	TT	TT	TT	TT
GG	GG	GG	GG	GG	GG	GG	TT	GG
NN	AA	AA	AA	AA	AA	AT	AA	AA
CC	CC	CC	CC	CC	CC	CC	TT	CC
NN	GG	GG	GG	GG	GG	GA	GG	GG
NN	AA	NN	AA	AA	AA	AG	GG	AG
GG	AA	NN	GG	AA	GG	GG	GG	GG
TT	CC	CC	CC	CC	CC	CC	CC	CC

IRIS_313-1	IRIS_313-1	IRIS_313-1	IRIS_313-1	IRIS_313-1	IRIS_313-1	IRIS_313-1	IRIS_313-1	IRIS_313-1
TT	TT	TT	TT	TT	TT	TT	TT	TT
GG	GG	GG	GG	GG	GG	GG	GG	GG
AA	AA	AA	AA	AA	AA	AA	AA	AA
CC	CC	CC	CC	CC	CC	CC	CC	CC
GG	GG	GG	GG	GG	GG	GG	GG	GG
AA	AA	AA	AA	AA	AA	AA	AA	AA
GG	GG	GG	GG	GG	GG	GG	GG	GG
CC	CC	CC	CC	CC	CC	CC	CC	CC

IRIS_313-1	IRIS_313-1	IRIS_313-1	IRIS_313-1	IRIS_313-1	IRIS_313-1	IRIS_313-1	IRIS_313-1	IRIS_313-1
TT	CC	TC	TT	TT	CC	TT	CC	TT
GG	GG	GG	GG	GG	GG	GG	GG	GG
AA	TT	AT	AA	AA	TT	AA	TT	AA
CC	CC	CC	CC	CC	CC	CC	CC	CC
GG	AA	GA	GG	GG	AA	GG	AA	GG
AA	GG	AG	AA	AA	GG	AA	GG	AA
GG	GG	GG	GG	GG	GG	GG	GG	GG
CC	TT	CT	CC	CC	TT	CC	TT	CC

IRIS_313-1	IRIS_313-1	IRIS_313-1	IRIS_313-1	IRIS_313-1	IRIS_313-1	IRIS_313-1	IRIS_313-1	IRIS_313-1
CC	TT	TT	TT	TT	TT	TT	TT	TT
GG	TT	GG	TT	GG	GG	GG	GG	GG
TT	AA	AA	AA	AA	AA	AA	AA	AA
CC	TT	CC	TT	CC	CC	CC	CC	CC
AA	GG	GG	GG	GG	GG	GG	GG	GG
GG	GG	AA	GG	AA	AA	AA	AA	AA
GG	GG	GG	GG	GG	GG	GG	GG	GG
TT	CC	CC	CC	CC	CC	CC	CC	CC

IRIS_313-1	IRIS_313-1	IRIS_313-1	IRIS_313-1	IRIS_313-1	IRIS_313-1	IRIS_313-1	IRIS_313-1	IRIS_313-1
TT	TT	TT	TT	TT	CC	TT	CC	CC
GG	GG	GG	GG	GG	GG	GG	GG	GG
AA	AA	AA	AA	AA	TT	AA	TT	TT
CC	CC	CC	CC	CC	CC	CC	CC	CC
GG	GG	GG	GG	GG	AA	GG	AA	AA
AA	AA	AA	AA	NN	GG	AA	GG	GG
GG	GG	GG	GG	GG	GG	GG	GG	GG
CC	CC	CC	CC	CC	TT	CC	TT	TT

IRIS_313-1	IRIS_313-1	IRIS_313-1	IRIS_313-1	IRIS_313-1	IRIS_313-1	IRIS_313-1	IRIS_313-1	IRIS_313-1
TT	TT	TT	TT	TT	TT	TT	TT	TT
GG	GG	GG	GG	GG	GG	GG	TT	TT
AA	AA	AA	AA	AA	AA	AA	AA	AA
CC	CC	CC	CC	CC	CC	CC	TT	TT
GG	GG	GG	GG	GG	GG	GG	GG	GG
AA	AA	AA	AA	AA	AA	AA	GG	GG
GG	GG	GG	GG	GG	GG	GG	GG	GG
CC	CC	CC	CC	CC	CC	CC	CC	CC

IRIS_313-1	IRIS_313-1	IRIS_313-1	IRIS_313-1	IRIS_313-1	IRIS_313-1	IRIS_313-1	IRIS_313-1	IRIS_313-1
TT	TT	TT	TT	CC	CC	TT	TT	TT
TT	GG	GG	GG	GG	GG	GG	GG	GG
AA	AA	AA	AA	TT	TT	AA	AA	AA
TT	CC	CC	CC	CC	CC	CC	CC	CC
GG	GG	GG	GG	AA	AA	GG	GG	GG
GG	AA	AA	AA	GG	GG	AA	AA	AA
GG	GG	GG	GG	GG	GG	GG	GG	GG
CC	CC	CC	CC	TT	TT	CC	CC	CC

IRIS_313-1	IRIS_313-1	IRIS_313-1	IRIS_313-1	IRIS_313-1	IRIS_313-1	IRIS_313-1	IRIS_313-1	IRIS_313-1
TT	CC	CC	TT	TT	CC	TT	TT	TT
GG	GG	GG	GG	GG	GG	GG	GG	GG
AA	TT	TT	AA	AA	TT	AA	AA	AA
CC	CC	CC	CC	CC	CC	CC	CC	CC
GG	AA	AA	GG	GG	AA	GG	GG	GG
AA	GG	GG	AA	AA	GG	AA	AA	AA
GG	GG	GG	GG	GG	GG	GG	GG	GG
CC	TT	TT	CC	CC	TT	CC	CC	CC

IRIS_313-1	IRIS_313-1	IRIS_313-1	IRIS_313-1	IRIS_313-1	IRIS_313-1	IRIS_313-1	IRIS_313-1	IRIS_313-1
TT	TT	TT	TT	TT	TT	CC	TT	NN
GG	GG	GG	GG	TT	GG	GG	GG	GG
AA	AA	AA	AA	AA	AA	TT	AA	AT
CC	CC	CC	CC	TT	CC	CC	CC	CC
GG	GG	GG	GG	GG	GG	AA	GG	NN
AA	AA	AA	AA	GG	AA	GG	AA	NN
GG	GG	GG	NN	GG	NN	GG	GG	GG
CC	CC	CC	CC	CC	CC	TT	CC	NN

IRIS_313-1	IRIS_313-1	IRIS_313-1	IRIS_313-1	IRIS_313-1	IRIS_313-1	IRIS_313-1	IRIS_313-1	IRIS_313-1
TT	TT	TT	CC	TT	TT	TT	CC	TT
TT	GG	GG	GG	GG	GG	GG	GG	GG
AA	AA	AA	TT	AA	AA	AA	TT	AA
TT	CC	CC	CC	CC	CC	CC	NN	CC
GG	GG	GG	AA	GG	GG	GG	AA	GG
GG	AA	AA	GG	AA	AA	AA	GG	AA
GG	GG	GG	GG	GG	GG	AA	GG	AA
CC	CC	CC	TT	CC	CC	CC	TT	CC

IRIS_313-1	IRIS_313-1	IRIS_313-1	IRIS_313-1	IRIS_313-1	IRIS_313-1	IRIS_313-1	IRIS_313-1	IRIS_313-1
CC	TT	TT	TT	TT	TT	TT	TT	TT
GG	GG	GG	GG	GG	GG	GG	GG	TT
TT	AA	AA	AA	AA	AA	AA	AA	AA
CC	CC	CC	CC	CC	CC	CC	CC	TT
AA	GG	GG	GG	GG	GG	GG	GG	GG
GG	AA	AA	AA	AA	NN	AA	AA	GG
GG	GG	GG	GG	GG	GG	GG	GG	GG
TT	CC	CC	CC	CC	NN	CC	CC	CC

IRIS_313-1	IRIS_313-1	IRIS_313-1	IRIS_313-1	IRIS_313-1	IRIS_313-1	IRIS_313-1	IRIS_313-1	IRIS_313-1
TT	TT	TT	CC	CC	TT	CC	TC	TT
GG	TT	GG	GG	GG	GG	GG	GG	GG
AA	AA	AA	TT	TT	AA	TT	AT	AA
CC	TT	CC	CC	CC	CC	CC	CC	CC
GG	GG	GG	AA	AA	GG	AA	AA	GG
AA	GG	AA	GG	GG	AA	GG	GG	AA
GG	GG	GG	GG	GG	GG	GG	GG	GG
CC	CC	CC	TT	TT	CC	TT	TT	CC

IRIS_313-1	IRIS_313-1	IRIS_313-1	IRIS_313-1	IRIS_313-1	IRIS_313-1	IRIS_313-1	IRIS_313-1	IRIS_313-1
TT	TT	TT	TT	TT	TT	TT	TT	TT
GG	GG	GG	GG	GG	GG	GG	GG	GG
AA	AA	AA	AA	AA	AA	AA	AA	AA
CC	CC	CC	CC	CC	CC	CC	CC	CC
GG	GG	GG	GG	GG	GG	GG	GG	GG
AA	AA	AA	AA	AA	AA	AA	AA	AA
GG	GG	GG	GG	GG	GG	GG	GG	GG
CC	CC	CC	CC	CC	CC	CC	CC	CC

IRIS_313-1	IRIS_313-1	IRIS_313-1	IRIS_313-1	IRIS_313-1	IRIS_313-1	IRIS_313-1	IRIS_313-1	IRIS_313-1
TT	TT	TT	TT	TT	CC	CC	TT	TT
GG	GG	GG	GG	GG	GG	GG	GG	GG
AA	AA	AA	AA	AA	TT	TT	AA	AA
CC	CC	CC	CC	CC	CC	CC	CC	CC
GG	GG	GG	GG	GG	AA	AA	GG	GG
AA	AA	AA	AA	AA	GG	GG	NN	AA
GG	GG	GG	GG	GG	GG	GG	GG	GG
CC	CC	CC	CC	CC	TT	TT	CC	CC

IRIS_313-1	IRIS_313-1	IRIS_313-1	IRIS_313-1	IRIS_313-1	IRIS_313-1	IRIS_313-1	IRIS_313-1	IRIS_313-1
TT	CC	TT	TT	TT	TT	TT	TT	TT
GG	GG	GG	GG	GG	GG	GG	GG	GG
AA	TT	AA	AA	AA	AA	AA	AA	AA
CC	CC	CC	CC	CC	CC	CC	CC	CC
GA	AA	GG	GG	GG	GG	GA	GG	GG
AA	GG	AA	AA	AA	AA	NN	AA	AA
GG	GG	GG	GG	GG	GG	GG	GG	GG
CC	TT	CC	NN	CC	CC	CC	CC	CC

IRIS_313-1	IRIS_313-1	IRIS_313-1	IRIS_313-1	IRIS_313-1	IRIS_313-1	IRIS_313-1	IRIS_313-1	IRIS_313-1
TT	TT	TT	TT	TT	TC	TT	TT	TT
TT	GG	GG	GG	GG	GG	GG	GG	GG
AA	AA	AA	AA	AA	AA	AA	AA	AA
TT	CC	CC	CC	CC	CC	CC	CC	CC
GA	GG	GG	GG	GG	GG	GG	GG	GG
GG	AA	NN	AA	AA	AG	AA	AA	AA
GG	GG	NN	GA	GG	GG	GG	GG	GG
CC	CC	CC	CC	CC	CC	CC	CC	CC

IRIS_313-1	IRIS_313-1	IRIS_313-1	IRIS_313-1	IRIS_313-1	IRIS_313-1	IRIS_313-1	IRIS_313-1	IRIS_313-1
TT	TT	TT	TT	TT	CC	TC	CC	NN
GG	GG	GG	GG	GG	GG	GG	GG	GG
AA	AA	AA	AA	AA	TT	AT	TT	TT
CC	CC	CC	CC	CC	CC	CC	CC	CC
GG	GG	GG	GG	GG	AA	GA	AA	AA
AA	AA	AA	AA	AA	GG	NN	GG	GG
GG	GG	GG	GG	GG	GG	GG	GG	GG
CC	CC	CC	CC	NN	TT	CT	TT	TT

IRIS_313-1	IRIS_313-1	IRIS_313-1	IRIS_313-1	IRIS_313-1	IRIS_313-1	IRIS_313-1	IRIS_313-1	IRIS_313-1
CC	CC	TT	TT	TT	CC	CC	CC	TT
GG	GG	GG	GG	GG	GG	GG	GG	GG
TT	TT	AA	AA	AA	TT	TT	TT	AA
CC	CC	CC	CC	CC	CC	CC	CC	CC
AA	AA	GG	GG	GG	AA	AA	AA	GG
GG	GG	AA	AA	AA	GG	GG	GG	AA
GG	GG	GG	NN	GG	GG	GG	GG	GG
TT	TT	CC	CC	CC	TT	TT	TT	CC

IRIS_313-1	IRIS_313-1	IRIS_313-1	IRIS_313-1	IRIS_313-1	IRIS_313-1	IRIS_313-1	IRIS_313-1	IRIS_313-1
TT	CC	CC	TT	TT	TT	TT	TT	TT
GG	GG	GG	GG	GG	GG	GG	GG	GG
AA	TT	TT	AA	AA	AA	AA	AA	AA
CC	CC	CC	CC	CC	CC	CC	CC	CC
GG	AA	AA	GG	GG	GG	GG	GG	GG
AA	GG	NN	AA	AA	AA	AA	AA	AA
GG	GG	GG	GG	GG	GG	GG	GG	GG
CC	TT	TT	CC	CC	CC	CC	CC	CC

IRIS_313-1	IRIS_313-1	IRIS_313-1	IRIS_313-1	IRIS_313-1	IRIS_313-1	IRIS_313-1	IRIS_313-1	IRIS_313-1
TT	CC	TT	TT	TT	TT	TT	TT	TT
GG	NN	TT	GG	TT	TT	GG	TT	TT
AA	TT	AA	AA	AA	AA	AA	AA	AA
CC	CC	TT	CC	TT	TT	CC	TT	TT
GG	AA	GG	GG	GG	GG	GG	GG	GG
AA	GG	GG	AA	GG	GG	AA	GG	GG
GG	GG	GG	GG	GG	GG	GG	GG	GG
CC	TT	CC	CC	CC	CC	CC	CC	CC

IRIS_313-1	IRIS_313-1	IRIS_313-1	IRIS_313-1	IRIS_313-1	IRIS_313-1	IRIS_313-1	IRIS_313-1	IRIS_313-1
TT	TT	TT	CC	CC	TT	TT	TT	CC
GG	GG	GG	GG	GG	GG	GG	GG	GG
AA	AA	AA	TT	TT	AA	AA	AA	TT
CC	CC	CC	CC	CC	CC	CC	CC	CC
GG	GG	GG	AA	AA	GG	GG	GG	AA
AA	AA	AA	GG	GG	AA	AA	AA	GG
GG	GG	GG	GG	GG	GG	GG	GG	GG
CC	CC	CC	TT	TT	CC	CC	CC	TT

IRIS_313-1	IRIS_313-1	IRIS_313-1	IRIS_313-1	IRIS_313-1	IRIS_313-1	IRIS_313-1	IRIS_313-1	IRIS_313-1
TT	TT	TT	CC	TT	TT	TT	TT	TT
GG	TT	TT	GG	GG	TT	TT	TT	TT
AA	AA	AA	TT	AA	AA	AA	AA	AA
CC	TT	TT	CC	CC	TT	TT	TT	TT
GG	GG	GG	AA	GG	GG	GG	GG	GG
AA	GG	GG	GG	AA	GG	GG	GG	GG
GG	GG	GG	GG	GG	GG	GG	GG	GG
CC	CC	CC	TT	CC	CC	CC	CC	CC

IRIS_313-1	IRIS_313-1	IRIS_313-1	IRIS_313-1	IRIS_313-1	IRIS_313-1	IRIS_313-1	IRIS_313-1	IRIS_313-1
TT	TT	TT	TT	TT	TT	TT	TT	CC
GG	GT	TT	GG	GG	GG	TT	GG	GG
AA	AA	AA	AA	AA	AA	AA	AA	TT
CC	CT	TT	CC	CC	CC	TT	CC	CC
GG	NN	GG	GG	GG	GG	GG	GG	AA
AA	AA	GG	AA	AA	AA	GG	AA	GG
GG	GG	GG	GG	GG	GG	GG	GG	GG
CC	CC	CC	CC	CC	CC	CC	CC	TT

IRIS_313-1	IRIS_313-1	IRIS_313-1	IRIS_313-1	IRIS_313-1	IRIS_313-1	IRIS_313-1	IRIS_313-1	IRIS_313-1
TT	CC	TC	CC	TT	TT	TT	TT	TT
GG	GG	GG	GG	GG	GG	GG	GG	GG
AA	TT	AA	TT	AA	AA	AA	AA	AA
CC	CC	CC	CC	CC	CC	CC	CC	CC
GG	AA	GG	AA	GG	GG	GG	GG	GG
AA	GG	AA	GG	AA	AA	AA	AA	AA
GG	GG	GG	GG	GG	GG	GG	GG	GG
CC	TT	CC	TT	CC	CC	CC	CC	CC

IRIS_313-1	IRIS_313-1	IRIS_313-1	IRIS_313-1	IRIS_313-1	IRIS_313-1	IRIS_313-1	IRIS_313-1	IRIS_313-1
TT	TT	TT	CC	TT	TT	TT	TT	TT
GG	GG	GG	GG	GG	GG	GG	GG	GG
AA	AA	AA	TT	AA	AA	AA	AA	AA
CC	CC	CC	CC	CC	CC	CC	CC	CC
GG	GG	GG	AA	GG	GG	GG	GG	GG
AA	NN	AA	GG	AA	AA	AA	AA	AA
GG	GG	GG	GG	GG	GG	GG	GG	GG
CC	CC	CC	TT	CC	CC	CC	CC	CC

IRIS_313-1	IRIS_313-1	IRIS_313-1	IRIS_313-1	IRIS_313-1	IRIS_313-1	IRIS_313-1	IRIS_313-1	IRIS_313-1
TT	CC	TT	CC	TT	CC	CC	TT	TT
GG	GG	GG	GG	GG	GG	GG	GG	GG
AA	TT	AA	TT	AA	TT	TT	AA	AA
CC	CC	CC	CC	CC	CC	CC	CC	CC
GG	AA	GG	AA	GG	AA	AA	GG	GG
AA	GG	AA	GG	AA	GG	GG	AA	AA
NN	GG	GG	GG	GG	GG	GG	GG	GG
CC	TT	CC	TT	CC	TT	TT	CC	CC

IRIS_313-1	IRIS_313-1	IRIS_313-1	IRIS_313-1	IRIS_313-1	IRIS_313-1	IRIS_313-1	IRIS_313-1	IRIS_313-1
CC	TT	TT	CC	CC	TT	CC	CC	TT
GG	GG	GG	GG	GG	GG	GG	GG	GG
TT	AA	AA	TT	TT	AA	TT	TT	AA
CC	CC	CC	CC	CC	CC	CC	CC	CC
AA	GG	GG	AA	AA	GG	AA	AA	GG
GG	NN	AA	GG	GG	AA	GG	GG	AA
GG	GG	GG	GG	GG	GG	GG	GG	GG
TT	CC	CC	TT	TT	CC	TT	TT	CC

IRIS_313-1	IRIS_313-1	IRIS_313-1	IRIS_313-1	IRIS_313-1	IRIS_313-1	IRIS_313-1	IRIS_313-1	IRIS_313-1
CC	TT	TT	TT	TT	CC	TT	TT	TT
GG	GG	GG	GG	GG	GG	GG	GG	GG
TT	AA	AA	AA	AA	TT	AA	AA	AA
CC	CC	CC	CC	CC	CC	CC	CC	CC
AA	GG	GG	GG	GG	AA	GG	GG	GG
GG	AA	AA	AA	AA	GG	AA	NN	AA
GG	GG	GG	GG	GG	GG	GG	NN	GG
TT	CC	CC	CC	CC	TT	CC	CC	CC

IRIS_313-1	IRIS_313-1	IRIS_313-1	IRIS_313-1	IRIS_313-1	IRIS_313-1	IRIS_313-1	IRIS_313-1	IRIS_313-1
TT	TT	TT	TT	TT	TT	TT	CC	TT
GG	GG	GG	TT	GG	GG	GG	GG	GG
AA	AA	AA	AA	AA	AA	AA	TT	AA
CC	CC	CC	TT	CC	CC	CC	CC	CC
GG	GG	GG	GG	GG	GG	GG	AA	GG
AA	AA	AA	GG	AA	AA	AA	GG	AA
GG	GG	GG	GG	GG	GG	AA	GG	GG
CC	CC	CC	CC	CC	CC	CC	TT	CC

IRIS_313-1	IRIS_313-1	IRIS_313-1	IRIS_313-1	IRIS_313-1	IRIS_313-1	IRIS_313-1	IRIS_313-1	IRIS_313-1
TT	CC	TT	TT	CC	TT	TT	CC	CC
GG	GG	GG	GG	GG	GG	GG	GG	GG
AA	TT	AA	AA	TT	AA	AA	TT	TT
CC	CC	CC	CC	CC	CC	CC	CC	CC
GG	AA	GG	GG	AA	GG	GG	AA	AA
AA	GG	AA	AA	GG	AA	AA	GG	GG
GG	GG	GG	GG	GG	GG	GG	GG	GG
CC	TT	CC	CC	TT	CC	CC	TT	TT

IRIS_313-1	IRIS_313-1	IRIS_313-1	IRIS_313-1	IRIS_313-1	IRIS_313-1	IRIS_313-1	IRIS_313-1	IRIS_313-1
TT	TT	CC	TT	TT	CC	CC	TT	CC
GG	GG	GG	GG	GG	GG	GG	GG	GG
AA	AA	TT	AA	AA	TT	TT	AA	TT
CC	CC	CC	CC	CC	CC	CC	CC	CC
GG	GG	AA	GG	GG	AA	AA	GG	AA
AA	AA	GG	AA	AA	GG	GG	AA	GG
GG	GG	GG	GG	GG	GG	GG	GG	GG
CC	CC	TT	CC	CC	TT	TT	CC	TT

IRIS_313-1	IRIS_313-1	IRIS_313-1	IRIS_313-1	IRIS_313-1	IRIS_313-1	IRIS_313-1	IRIS_313-1	IRIS_313-1
TT	CC	TT	TT	TT	TT	TT	TT	TT
GG	GG	GG	TT	TT	GG	GT	GG	GG
AA	TT	AA	AA	AA	AA	AA	AA	AA
CC	CC	CC	TT	TT	CC	CT	CC	CC
GG	AA	GG	GG	GG	GG	GG	GG	GG
AA	GG	AA	GG	GG	AA	GG	AA	AA
GG	GG	GG	GG	GG	GG	GG	GG	GG
CC	TT	CC	CC	CC	CC	CC	CC	CC

IRIS_313-1	IRIS_313-1	IRIS_313-1	IRIS_313-1	IRIS_313-1	IRIS_313-1	IRIS_313-1	IRIS_313-1	IRIS_313-1
TT	TT	TT	TT	TT	TT	TT	TT	CC
TT	TT	GG	GG	GG	GG	GG	GG	GG
AA	AA	AA	NN	AA	AA	AA	AA	TT
TT	TT	CC	CC	CC	CC	CC	CC	CC
GG	GG	GG	GG	GG	GG	GG	GG	AA
GG	GG	AA	AA	AA	NN	AA	AA	GG
GG	GG	GG	GG	GG	GG	GG	GG	GG
CC	CC	CC	CC	CC	CC	CC	NN	TT

IRIS_313-1	IRIS_313-1	IRIS_313-1	IRIS_313-1	IRIS_313-1	IRIS_313-1	IRIS_313-1	IRIS_313-1	IRIS_313-1
TT	TT	TT	TT	TT	TT	TT	TT	CC
GG	TT	GG	GG	TT	GG	GG	GG	GG
AA	AA	AA	AA	AA	AA	AA	AA	TT
CC	TT	CC	CC	TT	CC	CC	CC	CC
GG	GG	GG	GG	GG	GG	GG	GG	AA
AA	GG	AA	AG	GG	AA	AA	AA	GG
GG	GG	GG	GG	GG	GG	GG	GG	GG
CC	CC	CC	CC	CC	CC	CC	CC	TT

IRIS_313-1	IRIS_313-1	IRIS_313-1	IRIS_313-1	IRIS_313-1	IRIS_313-1	IRIS_313-1	IRIS_313-1	IRIS_313-1
TT	CC	CC	CC	TC	TT	CC	CC	TT
GG	GG	GG	GG	NN	GG	GG	GG	GG
AA	TT	TT	TT	AT	AA	TT	TT	AA
CC	CC	CC	CC	CC	CC	CC	CC	CC
GG	AA	AA	AA	GA	GG	AA	AA	GG
AA	GG	GG	GG	AG	AA	GG	GG	AA
GG	GG	GG	GG	GG	GG	GG	GG	GG
CC	TT	TT	TT	NN	CC	TT	TT	CC

IRIS_313-1	IRIS_313-1	IRIS_313-1	IRIS_313-1	IRIS_313-1	IRIS_313-1	IRIS_313-1	IRIS_313-1	IRIS_313-1
TT	TT	TT	TT	TT	TT	TT	TT	TT
TT	GG	GG	GG	GG	GG	GG	TT	GG
AA	AA	AA	AA	AA	AA	AA	AA	AA
TT	CC	CC	CC	CC	CC	CC	TT	CC
GG	GG	GG	GG	GG	GG	GG	GG	GG
GG	AA	AA	AA	AA	AA	AA	GG	AA
GG	GG	GG	GG	GG	GG	GG	GG	GG
CC	CC	CC	CC	CC	CC	CC	CC	CC

IRIS_313-1	IRIS_313-1	IRIS_313-1	IRIS_313-1	IRIS_313-1	IRIS_313-1	IRIS_313-1	IRIS_313-1	IRIS_313-1
TT	TT	TT	TT	CC	CC	CC	CC	CC
GG	GG	GG	GG	GG	GG	GG	GG	GG
AA	AA	AA	AA	TT	TT	TT	TT	TT
CC	CC	CC	CC	CC	CC	CC	CC	CC
GG	GG	GG	GG	AA	AA	AA	AA	AA
AA	AA	AA	AA	GG	GG	GG	GG	GG
GG	GG	GG	GG	GG	GG	GG	GG	GG
CC	CC	CC	CC	TT	TT	TT	TT	TT

IRIS_313-1	IRIS_313-1	IRIS_313-1	IRIS_313-1	IRIS_313-1	IRIS_313-1	IRIS_313-1	IRIS_313-1	IRIS_313-1
TT	TT	CC	CC	CC	CC	TT	TT	TT
GG	GG	GG	GG	GG	GG	GG	GG	GG
AA	AA	TT	TT	TT	TT	AA	AA	AA
CC	CC	CC	CC	CC	CC	CC	CC	CC
GG	GG	AA	AA	AA	AA	GG	GG	GG
AA	AA	GG	GG	GG	GG	AA	AA	AA
GG	GG	GG	GG	GG	GG	GG	GG	GG
CC	CC	TT	TT	TT	TT	CC	CC	CC

IRIS_313-1	IRIS_313-1	IRIS_313-1	IRIS_313-1	IRIS_313-1	IRIS_313-1	IRIS_313-1	IRIS_313-1	IRIS_313-1
TT	TT	TT	TT	TT	CC	CC	TT	TT
GG	GG	GG	GG	GG	GG	GG	GG	GG
AA	AA	AA	AA	AA	TT	TT	AA	AA
CC	CC	CC	CC	CC	CC	CC	CC	CC
GG	GG	GG	GG	GG	AA	AA	GG	GG
AA	AA	AA	AA	AA	GG	GG	AA	AA
GG	GG	GG	GG	GG	GG	GG	NN	GG
CC	CC	CC	CC	CC	TT	TT	CC	CC

IRIS_313-1	IRIS_313-1	IRIS_313-1	IRIS_313-1	IRIS_313-1	IRIS_313-1	IRIS_313-1	IRIS_313-1	IRIS_313-1
TT	TT	TT	TT	TT	TT	TT	TT	TT
GG	GG	GG	GG	GG	GG	GG	GG	GG
AA	AA	AA	AA	AA	AA	AA	AA	AA
CC	CC	CC	CC	CC	CC	CC	CC	CC
GG	GG	GG	GG	GG	GG	GG	GG	GG
AG	AA	AA	AA	AA	AA	AA	AA	AA
GG	GG	GG	GG	GG	GG	GG	GG	GG
CC	CC	CC	CC	CC	CC	CC	CC	CC

IRIS_313-1	IRIS_313-1	IRIS_313-1	IRIS_313-1	IRIS_313-1	IRIS_313-1	IRIS_313-1	IRIS_313-1	IRIS_313-1
TT	TT	TT	CC	TT	CC	TT	TT	TT
GG	GG	GG	GG	GG	GG	GG	GG	GG
AA	AA	AA	TT	AA	TT	AA	AA	AA
CC	CC	CC	CC	CC	CC	CC	CC	CC
GG	GG	GG	AA	GG	AA	GG	GG	GG
AA	AA	AA	GG	AA	GG	AA	AA	AA
GG	GG	GG	GG	GG	GG	GG	GG	GG
CC	CC	CC	TT	CC	TT	CC	CC	CC

IRIS_313-1	IRIS_313-1	IRIS_313-1	IRIS_313-1	IRIS_313-1	IRIS_313-1	IRIS_313-1	IRIS_313-1	IRIS_313-1
TT	CC	TT	CC	TT	TT	TT	TT	TT
GG	GG	GG	GG	GG	GG	GG	GG	GG
AA	TT	AA	TT	AA	AA	AA	AA	AA
CC	CC	CC	CC	CC	CC	CC	CC	CC
GG	AA	GG	AA	GG	GG	GG	GG	GG
AA	GG	AA	GG	AA	AA	AA	AA	AA
GG	GG	GG	GG	GG	GG	GG	GG	GG
CC	TT	CC	TT	CC	CC	CC	CC	CC

IRIS_313-1	IRIS_313-1	IRIS_313-1	IRIS_313-1	IRIS_313-1	IRIS_313-1	IRIS_313-1	IRIS_313-1	IRIS_313-1
TT	TT	TT	TT	TT	TT	TT	TT	TT
GG	GG	GG	TT	GG	GG	GG	GG	GG
AA	AA	AA	AA	AA	AA	AA	AA	AA
CC	CC	CC	TT	CC	CC	CC	CC	CC
GG	GG	GG	GG	GG	NN	GG	GG	GG
AA	AA	AA	GG	AA	AA	AA	AA	AA
GG	GG	GG	GG	GG	GG	GG	GG	GG
CC	CC	CC	CC	CC	CC	CC	CC	CC

IRIS_313-1	IRIS_313-1	IRIS_313-1	IRIS_313-1	IRIS_313-1	IRIS_313-1	IRIS_313-1	IRIS_313-1	IRIS_313-1
TT	TT	TT	TT	TT	CC	TT	TT	TT
GG	GG	GG	GG	GG	GG	GG	GG	GG
AA	AA	AA	AA	AA	TT	AA	AA	AA
CC	CC	CC	CC	CC	CC	CC	CC	CC
GG	GG	GG	GG	GG	AA	GG	GG	GG
AA	AA	AA	AA	AA	GG	AA	AG	AA
GG	GG	GG	GG	GG	GG	GG	GG	GG
CC	CC	CC	CC	CC	TT	CC	CC	CC

IRIS_313-1	IRIS_313-1	IRIS_313-1	IRIS_313-1	IRIS_313-1	IRIS_313-1	IRIS_313-1	IRIS_313-1	IRIS_313-1
TT	TT	TT	TT	TT	TT	TT	CC	TT
GG	GG	GG	GG	GG	GG	GG	GG	GG
AA	AA	AA	AA	AA	AA	AA	TT	AA
CC	CC	CC	CC	CC	CC	CC	CC	CC
GG	GG	GG	GG	GG	GG	GG	AA	GG
AA	AA	AA	AA	AA	AA	AA	GG	AA
GG	GG	GG	GG	GG	GG	GG	GG	GG
CC	CC	CC	CC	CC	CC	CC	TT	CC

IRIS_313-1	IRIS_313-1	IRIS_313-1	IRIS_313-1	IRIS_313-1	IRIS_313-1	IRIS_313-1	IRIS_313-1	IRIS_313-1
TT	CC	TT	TT	TT	CC	TT	TT	TT
GG	GG	GG	GG	TT	GG	GG	GG	GG
AA	NN	AA	AA	AA	TT	AA	AA	AA
CC	CC	CC	CC	TT	CC	CC	CC	CC
GG	AA	GG	GG	GG	AA	GG	GG	GG
AA	GG	AA	AA	GG	GG	AA	AA	AA
GG	GG	GG	GG	GG	GG	GG	GG	GG
CC	TT	CC	CC	CC	TT	CC	CC	CC

IRIS_313-1	IRIS_313-1	IRIS_313-1	IRIS_313-1	IRIS_313-1	IRIS_313-1	IRIS_313-1	IRIS_313-1	IRIS_313-1
TT	TT	TT	TT	TT	TT	TT	CC	CC
GG	GG	GG	GG	GG	GG	GG	GG	GG
AA	AA	AA	AA	AA	AA	AA	TT	TT
CC	CC	CC	CC	CC	CC	CC	CC	CC
GG	GG	GG	GG	GG	GG	GG	AA	AA
AA	AA	AA	AA	AA	AA	AA	GG	GG
GG	GG	GG	GG	GG	GG	GG	GG	GG
CC	CC	CC	CC	CC	CC	CC	TT	TT

IRIS_313-1	IRIS_313-1	IRIS_313-1	IRIS_313-1	IRIS_313-1	IRIS_313-1	IRIS_313-1	IRIS_313-1	IRIS_313-1
TT	TT	CC	TT	TT	TT	TT	TT	CC
GG	GG	GG	GG	GG	GG	GG	GG	GG
AA	AA	TT	AA	AA	AA	AA	AA	TT
CC	CC	CC	CC	CC	CC	CC	CC	CC
GG	GG	AA	GG	GG	GG	GG	GG	AA
AA	AA	GG	AA	AA	AA	AA	AA	GG
GG	GG	GG	GG	GG	GG	GG	GG	GG
CC	CC	TT	CC	CC	CC	CC	CC	TT

IRIS_313-1	IRIS_313-1	IRIS_313-1	IRIS_313-1	IRIS_313-1	IRIS_313-1	IRIS_313-1	IRIS_313-1	IRIS_313-1
TT	TT	TT	TT	TT	TT	TT	TT	TT
TT	GG	GG	GG	GG	GG	GG	GG	GG
AA	AA	AA	AA	AA	AA	AA	AA	AA
TT	CC	CC	CC	CC	CC	CC	CC	CC
GG	GG	GG	GG	GG	GG	GG	GG	GG
GG	AA	AA	AA	AA	AA	NN	AA	AA
GG	GG	GG	GG	GG	GG	GG	GG	GG
CC	CC	CC	CC	CC	CC	CC	CC	CC

IRIS_313-1	IRIS_313-1	IRIS_313-1	IRIS_313-1	IRIS_313-1	IRIS_313-1	IRIS_313-1	IRIS_313-1	IRIS_313-1
TT	TT	TT	CC	TT	CC	TT	CC	TT
GG	GG	GG	GG	GG	GG	GG	GG	GG
AA	AA	AA	TT	AA	TT	AA	TT	AA
CC	CC	CC	CC	CC	CC	CC	CC	CC
GG	GG	GG	AA	GG	AA	GG	AA	GG
AA	AA	AA	GG	AA	GG	AA	GG	AA
GG	GG	GG	GG	GG	GG	GG	GG	GG
CC	CC	CC	TT	CC	TT	CC	TT	CC

IRIS_313-1	IRIS_313-1	IRIS_313-1	IRIS_313-1	IRIS_313-1	IRIS_313-1	IRIS_313-1	IRIS_313-1	IRIS_313-1
TT	TT	TT	TT	TT	TT	CC	TT	TT
GG	GG	GG	GG	GG	GG	GG	GG	GG
AA	AA	AA	AA	AA	AA	TT	AA	AA
CC	CC	CC	CC	CC	CC	CC	CC	CC
GG	GG	GG	GG	GG	GG	AA	GG	GG
AA	AA	AA	AA	AA	AA	GG	AA	AA
GG	GG	GG	GG	GG	GG	GG	GG	GG
CC	CC	CC	CC	CC	CC	TT	CC	CC

IRIS_313-1	IRIS_313-1	IRIS_313-1	IRIS_313-1	IRIS_313-1	IRIS_313-1	IRIS_313-1	IRIS_313-1	IRIS_313-1
CC	TT	TT	TT	TT	TT	TT	TT	TT
GG	GG	GG	GG	GG	GG	GG	GG	GG
TT	AA	AA	AA	AA	AA	AA	AA	AA
CC	CC	CC	CC	CC	CC	CC	CC	CC
AA	GG	GG	GG	GG	GG	GG	GG	GG
GG	AA	AA	AA	AA	AA	AA	AA	AA
GG	GG	GG	GG	GG	GG	GG	GG	GG
TT	CC	CC	CC	CC	CC	CC	CC	CC

IRIS_313-1	IRIS_313-1	IRIS_313-1	IRIS_313-1	IRIS_313-1	IRIS_313-1	IRIS_313-1	IRIS_313-1	IRIS_313-1
TT	TT	TT	TT	TT	TT	TT	TT	TT
GG	GG	GG	GG	GG	GG	GG	GG	GG
AA	AA	AA	AA	AA	AA	AA	AA	AA
CC	CC	CC	CC	CC	CC	CC	CC	CC
GG	GG	GG	GG	GG	GG	GG	GG	GG
AA	AA	AA	AA	AA	AA	AA	AA	AA
GG	GG	GG	GG	GG	GG	GG	GG	GG
CC	CC	CC	CC	CC	CC	CC	CC	CC

IRIS_313-1	IRIS_313-1	IRIS_313-1	IRIS_313-1	IRIS_313-1	IRIS_313-1	IRIS_313-1	IRIS_313-1	IRIS_313-1
TT	TT	TT	TT	CC	CC	TT	TT	CC
GG	GG	GG	GG	GG	GG	GG	GG	GG
AA	AA	AA	AA	TT	TT	AA	AA	TT
CC	CC	CC	CC	CC	CC	CC	CC	CC
GG	GG	GG	GG	AA	AA	GG	GG	AA
AA	AA	AA	AA	GG	GG	AA	AA	GG
GG	GG	GG	GG	GG	GG	GG	GG	GG
CC	CC	CC	CC	TT	TT	CC	CC	TT

IRIS_313-1	IRIS_313-1	IRIS_313-1	IRIS_313-1	IRIS_313-1	IRIS_313-1	IRIS_313-1	IRIS_313-1	IRIS_313-1
TT	CC	CC	TT	TT	TT	TT	TT	TT
GG	GG	GG	GG	GG	GG	GG	GG	GG
AA	TT	TT	AA	AA	AA	AA	AA	AA
CC	CC	CC	CC	CC	CC	CC	CC	CC
GG	AA	AA	GG	GG	GG	GG	GG	GG
AA	GG	GG	AA	AA	AA	AA	AA	AA
GG	GG	GG	GG	GG	GG	GG	GG	GG
CC	TT	TT	CC	CC	CC	CC	CC	CC

IRIS_313-1	IRIS_313-1	IRIS_313-1	IRIS_313-1	IRIS_313-1	IRIS_313-1	IRIS_313-1	IRIS_313-1	IRIS_313-1
TT	TT	TT	TT	TT	TT	CC	CC	TT
GG	GG	GG	GG	GG	GG	GG	GG	GG
AA	AA	AA	AA	AA	AA	TT	TT	AT
CC	CC	CC	CC	CC	CC	NN	CC	CC
GG	GG	GG	GG	GG	GG	AA	NN	GG
AA	AA	AA	AA	AA	AA	GG	NN	AA
GG	GG	GG	GG	GG	GG	GG	GG	GG
CC	CC	CC	CC	CC	CC	TT	TT	CC

IRIS_313-1	IRIS_313-1	IRIS_313-1	IRIS_313-1	IRIS_313-1	IRIS_313-1	IRIS_313-1	IRIS_313-1	IRIS_313-1
TT	TT	CC	TT	TT	TT	TT	TT	TT
GG	GG	GG	GG	GG	GG	GG	GG	GG
AA	AA	TT	AA	AA	AA	AA	AA	AA
CC	CC	CC	CC	CC	CC	CC	CC	CC
GG	GG	AA	GG	GG	GG	GG	GG	GG
AA	AA	GG	AA	AA	AA	AA	AA	AA
GG	GG	GG	GG	GG	GG	GG	GG	GG
CC	CC	TT	CC	CC	CC	CC	CC	CC

IRIS_313-1	IRIS_313-1	IRIS_313-1	IRIS_313-1	IRIS_313-1	IRIS_313-1	IRIS_313-1	IRIS_313-1	IRIS_313-1
TT	TT	TT	TT	TT	TT	TT	TT	TT
GG	GG	GG	GG	GG	GG	GG	GG	GG
AA	AA	AA	AA	AA	AA	AA	AA	AA
CC	CC	CC	CC	CC	CC	CC	CC	CC
GG	GG	GG	GG	GG	GG	GG	GG	GG
AA	AA	AA	AA	AA	AA	AA	AA	AA
GG	GG	GG	GG	GG	GG	GG	GG	GG
CC	CC	CC	CC	CC	NN	CC	CC	CC

IRIS_313-1	IRIS_313-1	IRIS_313-1	IRIS_313-1	IRIS_313-1	IRIS_313-1	IRIS_313-1	IRIS_313-1	IRIS_313-1
TT	TT	TT	TT	TT	CC	TT	TT	TT
GG	GG	GG	GG	GG	GG	GG	GG	GG
AA	AA	AA	AA	AA	TT	AA	AA	AA
CC	CC	CC	CC	CC	CC	CC	CC	CC
GG	GG	GG	GG	GG	AA	GG	GG	GG
AA	AA	AA	AA	NN	GG	AA	AA	AA
GG	GG	GG	GG	GG	GG	GG	GG	GG
CC	CC	CC	CC	CC	TT	CC	CC	CC

IRIS_313-1	IRIS_313-1	IRIS_313-1	IRIS_313-1	IRIS_313-1	IRIS_313-1	IRIS_313-1	IRIS_313-1	IRIS_313-1
TT	TT	TT	TT	TT	TT	TT	TT	CC
GG	GG	GG	GG	GG	GG	GG	TT	GG
AT	AA	AT	AA	AA	AA	AA	AA	TT
CC	CC	CC	CC	CC	CC	CC	TT	CC
GG	GG	GG	GG	GG	GG	GG	GG	AA
AA	AA	AA	AA	AA	AA	AA	GG	GG
GG	GG	GG	GG	GG	GG	GG	GG	GG
CC	CC	CC	CC	CC	CC	CC	CC	TT

IRIS_313-1	IRIS_313-1	IRIS_313-1	IRIS_313-1	IRIS_313-1	IRIS_313-1	IRIS_313-1	IRIS_313-1	IRIS_313-1
CC	TT	TT	TT	TT	CC	TT	TT	CC
GG	GG	GG	GG	GG	GG	GG	GG	GG
TT	AA	AA	AA	AA	TT	AA	AA	TT
CC	CC	CC	CC	CC	CC	CC	CC	CC
AA	GG	GG	GG	GG	AA	GG	GG	AA
GG	AA	AA	AA	AA	GG	AA	AA	GG
GG	GG	GG	GG	GG	GG	GG	GG	GG
TT	CC	CC	CC	CC	TT	CC	CC	TT

IRIS_313-1	IRIS_313-1	IRIS_313-1	IRIS_313-1	IRIS_313-1	IRIS_313-1	IRIS_313-1	IRIS_313-1	IRIS_313-1
TT	TT	CC	TT	TT	CC	CC	CC	TT
GG	GG	GG	GG	GG	GG	GG	GG	GG
AA	AA	TT	AA	AA	TT	TT	TT	AA
CC	CC	CC	CC	CC	CC	CC	CC	CC
GG	GG	AA	GG	GG	AA	AA	AA	GG
AA	AA	GG	AA	AA	GG	GG	GG	AA
GG	GG	GG	GG	GG	GG	GG	GG	GG
CC	CC	TT	CC	CC	TT	TT	TT	CC

IRIS_313-1	IRIS_313-1	IRIS_313-1	IRIS_313-1	IRIS_313-1	IRIS_313-1	IRIS_313-1	IRIS_313-1	IRIS_313-1
TT	TT	TT	CC	TT	CC	TT	TT	TT
GG	GG	GG	GG	TT	GG	GG	TT	GG
AA	AA	AA	TT	AA	TT	AA	AA	AA
CC	CC	CC	CC	TT	CC	CC	TT	CC
GG	GG	GG	AA	GG	AA	GG	GG	GG
AA	AA	AA	GG	GG	GG	AA	GG	AA
GG	GG	GG	GG	GG	GG	GG	GG	GG
CC	CC	CC	TT	CC	TT	CC	CC	CC

IRIS_313-1	IRIS_313-1	IRIS_313-1	IRIS_313-1	IRIS_313-1	IRIS_313-1	IRIS_313-1	IRIS_313-1	IRIS_313-1
TT	TT	TT	CC	CC	CC	TT	CC	TT
GG	GG	GG	GG	GG	GG	GG	GG	GG
AA	AA	AA	TT	TT	TT	AA	TT	AA
CC	CC	CC	CC	CC	CC	CC	CC	CC
GG	GG	GG	AA	AA	AA	GG	AA	GG
AA	AA	AA	GG	GG	GG	AA	GG	AA
GG	GG	GG	GG	GG	GG	GG	GG	GG
CC	CC	CC	TT	TT	TT	CC	TT	CC

IRIS_313-1	IRIS_313-1	IRIS_313-1	IRIS_313-1	IRIS_313-1	IRIS_313-1	IRIS_313-1	IRIS_313-1	IRIS_313-1
CC	CC	TT	CC	TT	TT	TT	TT	TT
GG	GG	GG	GG	GG	GG	GG	GG	GG
TT	TT	AA	TT	AA	AA	AA	AA	AA
CC	CC	CC	CC	CC	CC	CC	CC	CC
AA	AA	GG	AA	GG	GG	GG	GG	GG
GG	GG	AA	GG	AA	AA	AA	AA	AA
GG	GG	GG	GG	GG	GG	GG	GG	GG
TT	TT	CC	TT	CC	CC	CC	CC	CC

IRIS_313-1	IRIS_313-1	IRIS_313-1	IRIS_313-1	IRIS_313-1	IRIS_313-1	IRIS_313-1	IRIS_313-1	IRIS_313-1
TT	TT	TT	TT	TT	TT	TT	CC	TT
GG	GG	GG	GG	GG	GG	GG	GG	GG
AA	AA	AA	AA	AA	AA	AA	TT	AA
CC	CC	CC	CC	CC	CC	CC	CC	CC
GG	GG	GG	GG	GG	GG	GG	AA	GG
AA	AA	AA	AA	AA	AA	AA	GG	AA
GG	GG	GG	GG	GG	GG	GG	GG	GG
CC	CC	CC	CC	CC	CC	CC	TT	CC

IRIS_313-1	IRIS_313-1	IRIS_313-1	IRIS_313-1	IRIS_313-1	IRIS_313-1	IRIS_313-1	IRIS_313-1	IRIS_313-1
TT	TT	TT	TT	TT	TT	TT	TT	TC
GG	GG	GG	GG	GG	GG	GG	GG	GG
AA	AA	AA	AA	AA	AA	AA	AA	NN
CC	CC	CC	CC	CC	CC	CC	CC	CC
GG	GG	GG	GG	GG	GG	GG	GG	NN
AA	AA	AA	AA	AA	AA	AA	AA	NN
GG	GG	GG	GG	GG	GG	GG	GG	GG
CC	CC	CC	CC	CC	CC	CC	CC	NN

IRIS_313-1	IRIS_313-1	IRIS_313-1	IRIS_313-1	IRIS_313-1	IRIS_313-1	IRIS_313-1	IRIS_313-1	IRIS_313-1
TT	TT	TT	TT	TT	TT	TT	TT	TT
GG	GG	GG	GG	GG	GG	TT	GG	GG
AA	AA	AA	AA	AA	AA	AA	AA	AA
CC	CC	CC	CC	CC	CC	TT	CC	CC
GG	GG	GG	GG	GG	GG	GG	GG	GG
AA	AA	AA	AA	AA	AA	GG	AA	AA
GG	GG	GG	GG	GG	GG	GG	GG	GG
CC	CC	CC	CC	CC	CC	CC	CC	CC

IRIS_313-1	IRIS_313-1	IRIS_313-1	IRIS_313-1	IRIS_313-1	IRIS_313-1	IRIS_313-1	IRIS_313-1	IRIS_313-1
TT	TT	TT	TT	CC	CC	CC	TT	TT
GG	GG	GG	TT	GG	GG	GG	GG	GG
AA	AA	AA	AA	TT	TT	TT	AA	AA
CC	CC	CC	TT	CC	CC	CC	CC	CC
GG	GG	GG	GG	AA	AA	AA	GG	GG
AA	AA	AA	GG	GG	GG	GG	AA	AA
GG	GG	GG	GG	GG	GG	GG	GG	GG
CC	CC	CC	CC	TT	TT	TT	CC	NN

IRIS_313-1	IRIS_313-1	IRIS_313-1	IRIS_313-1	IRIS_313-1	IRIS_313-1	IRIS_313-1	IRIS_313-1	IRIS_313-1
TT	TT	TT	TT	TT	CC	TT	TT	CC
GG	GG	GG	GG	GG	GG	TT	GG	GG
AA	AA	AA	AA	AA	TT	AA	AA	TT
CC	CC	CC	CC	CC	CC	TT	CC	CC
GG	GG	GG	GG	GG	NN	GG	GG	AA
AA	AA	AA	AA	AA	GG	GG	AA	GG
GG	GG	GG	GG	GG	GG	GG	GG	GG
CC	CC	CC	CC	CC	TT	CC	CC	TT

IRIS_313-1	IRIS_313-1	IRIS_313-1	IRIS_313-1	IRIS_313-1	IRIS_313-1	IRIS_313-1	IRIS_313-1	IRIS_313-1
TT	TT	TT	TT	TT	TT	TT	TT	TT
GG	GG	GG	GG	GG	GG	GG	GG	GG
AA	AA	AA	AA	AA	AA	AA	AA	AA
CC	CC	CC	CC	CC	CC	CC	CC	CC
GG	GG	GG	GG	GG	GG	GG	GG	GG
AA	AA	AA	AA	AA	AA	AA	AA	NN
GG	GG	GG	GG	GG	GG	GG	GG	NN
CC	CC	CC	CC	CC	CC	CC	CC	CC

IRIS_313-1	IRIS_313-1	IRIS_313-1	IRIS_313-1	IRIS_313-1	IRIS_313-1	IRIS_313-1	IRIS_313-1	IRIS_313-1
CC	CC	TT	TT	TT	TT	TT	TT	CC
GG	GG	GG	GG	GG	GG	GG	TT	GG
TT	TT	AA	AA	AA	AA	AA	AA	TT
CC	CC	CC	CC	CC	CC	CC	TT	CC
AA	AA	GG	GG	GG	GG	GG	GG	AA
GG	GG	AA	AA	AA	AA	AA	GG	GG
GG	GG	GG	GG	GG	GG	GG	GG	GG
TT	TT	CC	CC	CC	CC	CC	CC	TT

IRIS_313-1	IRIS_313-1	IRIS_313-1	IRIS_313-1	IRIS_313-1	IRIS_313-1	IRIS_313-1	IRIS_313-1	IRIS_313-1
TT	TT	CC	TT	TT	TT	TT	TT	TT
GG	GG	GG	GG	GG	GG	GG	GG	GG
AA	AA	TT	AA	AA	AA	AA	AA	AA
CC	CC	CC	CC	CC	CC	CC	CC	CC
GG	GG	AA	GG	GG	GG	GG	GG	GG
AA	AA	GG	AA	AA	AA	AA	AA	AA
GG	GG	GG	GG	GG	NN	GG	GG	GG
CC	CC	TT	CC	CC	CC	CC	CC	CC

IRIS_313-1	IRIS_313-1	IRIS_313-1	IRIS_313-1	IRIS_313-1	IRIS_313-1	IRIS_313-1	IRIS_313-1	IRIS_313-1
TT	TT	TT	TT	CC	CC	TT	TT	TT
GG	GG	GG	GG	GG	GG	GG	GG	GG
AA	AA	AA	AA	TT	TT	AA	AA	AA
CC	CC	CC	CC	CC	CC	CC	CC	CC
GG	GG	GG	GG	AA	AA	GG	GG	GG
AA	AA	AA	AA	GG	GG	AA	AA	AA
GG	GG	GG	GG	GG	GG	GG	GG	GG
CC	CC	CC	CC	TT	TT	CC	CC	CC

IRIS_313-1	IRIS_313-1	IRIS_313-1	IRIS_313-1	IRIS_313-1	IRIS_313-1	IRIS_313-1	IRIS_313-1	IRIS_313-1
CC	TT	CC	TT	TT	TT	TT	TT	TT
GG	GG	GG	GG	GG	GG	GG	GG	GG
TT	AA	TT	AA	AA	AA	AA	AA	AA
CC	CC	CC	CC	CC	CC	CC	CC	CC
AA	GG	AA	GG	GG	GG	GG	GG	GG
GG	AA	GG	AA	AA	NN	AA	AA	AA
GG	GG	GG	GG	GG	GG	GG	GG	GG
TT	CC	TT	CC	CC	CC	CC	CC	CC

IRIS_313-1	IRIS_313-1	IRIS_313-1	IRIS_313-1	IRIS_313-1	IRIS_313-1	IRIS_313-1	IRIS_313-1	IRIS_313-1
TT	TT	TT	TT	CC	TT	TT	TT	TT
GG	GG	GG	GG	GG	GG	GG	GG	GG
AA	AA	AA	AA	TT	AA	AA	AA	AA
CC	CC	CC	CC	CC	CC	CC	CC	CC
GG	GG	GG	GG	AA	GG	GG	GG	GG
AA	AA	AA	AA	GG	AA	AA	AA	AA
GG	GG	GG	GG	GG	GG	GG	GG	GG
CC	CC	CC	CC	TT	CC	CC	CC	CC

IRIS_313-1	IRIS_313-1	IRIS_313-1	IRIS_313-1	IRIS_313-1	IRIS_313-1	IRIS_313-1	IRIS_313-1	IRIS_313-1
TT	CC	TT	TT	CC	TT	TT	TT	CC
GG	GG	GG	GG	GG	GG	GG	GG	GG
AA	TT	AA	AA	TT	AA	AA	AA	TT
CC	CC	CC	CC	CC	CC	CC	CC	CC
GG	AA	GG	GG	AA	GG	GG	GG	AA
AA	GG	AA	AA	GG	AA	AA	AA	GG
GG	GG	GG	GG	GG	GG	GG	GG	GG
CC	TT	CC	CC	TT	CC	CC	CC	TT

IRIS_313-1	IRIS_313-1	IRIS_313-1	IRIS_313-1	IRIS_313-1	IRIS_313-1	IRIS_313-1	IRIS_313-1	IRIS_313-1
CC	CC	CC	TT	TT	TT	CC	CC	CC
GG	GG	GG	TT	GG	GG	GG	GG	GG
TT	TT	TT	AA	AA	AA	TT	TT	TT
CC	CC	CC	TT	CC	CC	CC	CC	CC
AA	AA	AA	GG	GG	GG	AA	AA	AA
GG	GG	GG	GG	AA	AA	GG	GG	GG
GG	GG	GG	GG	GG	GG	GG	GG	GG
TT	TT	TT	CC	CC	CC	TT	TT	TT

IRIS_313-1	IRIS_313-1	IRIS_313-1	IRIS_313-1	IRIS_313-1	IRIS_313-1	IRIS_313-1	IRIS_313-1	IRIS_313-1
CC	TT	CC	CC	CC	CC	TT	TT	TT
GG	GG	GG	GG	GG	GG	GG	GG	GG
TT	AA	TT	TT	TT	TT	AA	AA	AA
CC	CC	CC	CC	CC	CC	CC	CC	CC
AA	GG	AA	AA	AA	AA	GG	GG	GG
GG	AA	GG	GG	GG	GG	AA	AA	AA
GG	GG	GG	GG	GG	GG	GG	GG	GG
TT	CC	TT	TT	TT	TT	CC	CC	CC

IRIS_313-1	IRIS_313-1	IRIS_313-1	IRIS_313-1	IRIS_313-1	IRIS_313-1	IRIS_313-1	IRIS_313-1	IRIS_313-1
TT	TT	TT	TT	CC	TT	TT	TT	TT
GG	GG	GG	GG	GG	GG	GG	GG	GG
AA	AA	AA	AA	TT	AA	AA	AA	AA
CC	CC	CC	CC	CC	CC	CC	CC	CC
GG	GG	GG	GG	AA	GG	GG	GG	GG
AA	AA	AA	AA	GG	AA	AA	AA	AA
GG	GG	GG	GG	GG	GG	GG	GG	GG
CC	CC	CC	CC	TT	NN	CC	CC	CC

IRIS_313-1	IRIS_313-1	IRIS_313-1	IRIS_313-1	IRIS_313-1	IRIS_313-1	IRIS_313-1	IRIS_313-1	IRIS_313-1
CC	TT	TT	TT	TT	TT	CC	TT	TT
GG	GG	GG	GG	GG	GG	GG	GG	GG
TT	AA	AA	AA	AA	AA	TT	AA	AA
CC	CC	CC	CC	CC	CC	CC	CC	CC
AA	GG	GG	GG	GG	GG	AA	GG	GG
GG	AA	AA	AA	AA	AA	GG	AA	AA
GG	GG	GG	GG	GG	GG	GG	GG	GG
TT	CC	CC	CC	CC	CC	TT	CC	CC

IRIS_313-1	IRIS_313-1	IRIS_313-1	IRIS_313-1	IRIS_313-1	IRIS_313-1	IRIS_313-1	IRIS_313-1	IRIS_313-1
TT	CC	TT	TT	TT	TT	TT	TT	CC
GG	GG	GG	GG	GG	TT	GG	GG	GG
AA	TT	AA	AA	AA	AA	AA	AA	TT
CC	CC	CC	CC	CC	TT	CC	CC	CC
GG	AA	GG	GG	GG	GG	GG	GG	AA
AA	GG	AA	AA	AA	GG	AA	AA	GG
GG	GG	GG	GG	GG	GG	GG	GG	GG
CC	TT	CC	CC	CC	CC	CC	CC	TT

IRIS_313-1	IRIS_313-1	IRIS_313-1	IRIS_313-1	IRIS_313-1	IRIS_313-1	IRIS_313-1	IRIS_313-1	IRIS_313-1
TT	TT	TT	TT	TT	TT	CC	TT	TT
GG	GG	GG	GG	GG	GG	GG	GG	GG
AA	AA	AA	AA	AA	AA	TT	AA	AA
CC	CC	CC	CC	CC	CC	CC	CC	CC
GG	GG	GG	GG	GG	GG	AA	GG	GG
AA	NN	AA	AA	AA	AA	GG	AA	AA
GG	GG	GG	GG	GG	GG	GG	GG	GG
CC	CC	CC	CC	CC	CC	TT	CC	CC

IRIS_313-1	IRIS_313-1	IRIS_313-1	IRIS_313-1	IRIS_313-1	IRIS_313-1	IRIS_313-1	IRIS_313-1	IRIS_313-1
TT	TT	TT	TT	TT	TT	TT	TT	CC
GG	GG	GG	GG	GG	GG	GG	GG	GG
AA	AA	AA	AA	AA	AA	AA	AA	TT
CC	CC	CC	CC	CC	CC	CC	CC	CC
GG	GG	GG	GG	GG	GG	GG	GG	AA
AA	AA	AA	AA	AA	AA	AA	AA	GG
GG	GG	GG	GG	GG	GG	GG	GG	GG
CC	CC	CT	CC	CC	CC	CC	CC	TT

IRIS_313-1	IRIS_313-1	IRIS_313-1	IRIS_313-1	IRIS_313-1	IRIS_313-1	IRIS_313-1	IRIS_313-1	IRIS_313-1
TT	TT	CC	TT	TT	TT	TT	TT	CC
GG	GG	GG	GG	GG	GG	GG	GG	GG
AA	AA	TT	AA	AA	AA	AA	AA	TT
CC	CC	CC	CC	CC	CC	CC	CC	CC
GG	GG	AA	GG	GG	GG	GG	GG	AA
AA	AA	GG	AA	AA	AA	AA	AA	GG
AA	GG	GG	GG	GG	GG	GG	GG	GG
CC	CC	TT	CC	CC	CC	CC	CC	TT

IRIS_313-1	IRIS_313-1	IRIS_313-1	IRIS_313-1	IRIS_313-1	IRIS_313-1	IRIS_313-1	IRIS_313-1	IRIS_313-1
CC	CC	TT	TT	TT	TT	TT	TT	TT
GG	GG	GG	GG	GG	GG	GG	GG	GG
TT	TT	AA	AA	AA	AA	AA	AA	AA
CC	CC	CC	CC	CC	CC	CC	CC	CC
AA	AA	GG	NN	GA	GG	GG	GG	GG
GG	GG	AA	AA	AA	AA	AA	AA	AA
GG	GG	GG	GG	GG	GG	GG	GG	GG
TT	TT	CC	CC	CC	CC	CC	CC	CC

IRIS_313-1	IRIS_313-1	IRIS_313-1	IRIS_313-1	IRIS_313-1	IRIS_313-1	IRIS_313-1	IRIS_313-1	IRIS_313-1
CC	CC	CC	TT	TT	TT	TT	CC	CC
GG	GG	GG	GG	GG	GG	GG	GG	GG
TT	TT	TT	AA	AA	AA	AA	TT	TT
CC	CC	CC	CC	CC	CC	CC	CC	CC
AA	AA	AA	GG	GG	GG	GG	AA	AA
GG	GG	GG	AA	AA	AA	AA	GG	GG
GG	GG	GG	GG	GG	GG	GG	GG	GG
TT	TT	TT	CC	CC	CC	CC	TT	TT

IRIS_313-1	IRIS_313-1	IRIS_313-1	IRIS_313-1	IRIS_313-1	IRIS_313-1	IRIS_313-1	IRIS_313-1	IRIS_313-1
CC	CC	TT	TT	TT	CC	TT	CC	CC
GG	GG	GG	GG	GG	GG	GG	GG	GG
TT	TT	AA	AA	AA	TT	AA	TT	TT
CC	CC	CC	CC	CC	CC	CC	CC	CC
AA	AA	GG	GG	GG	AA	GG	AA	AA
GG	GG	AA	AA	AA	GG	AA	GG	GG
GG	GG	GG	GG	GG	GG	GG	GG	GG
TT	TT	CC	CC	CC	TT	CC	TT	TT

IRIS_313-1	IRIS_313-1	IRIS_313-1	IRIS_313-1	IRIS_313-1	IRIS_313-1	IRIS_313-1	IRIS_313-1	IRIS_313-1
TT	TT	TT	CC	CC	TT	TT	TT	CC
GG	GG	GG	GG	GG	GG	GG	GG	GG
AA	AA	AA	TT	TT	AA	AA	AA	TT
CC	CC	CC	CC	CC	CC	CC	CC	CC
GG	GG	GG	AA	AA	GG	GG	GG	AA
AA	AA	AA	GG	GG	AA	AA	AA	GG
GG	GG	GG	GG	GG	GG	GG	GG	GG
CC	CC	NN	TT	TT	CC	CC	CC	TT

IRIS_313-1	IRIS_313-1	IRIS_313-1	IRIS_313-1	IRIS_313-1	IRIS_313-1	IRIS_313-1	IRIS_313-1	IRIS_313-1
TT	CC	TT	TT	TT	CC	TT	TT	TT
GG	GG	GG	GG	GG	GG	GG	GG	GG
AA	TT	AA	AA	AA	TT	AA	AA	AA
CC	CC	CC	CC	CC	CC	CC	CC	CC
GG	AA	GG	GG	GG	AA	GG	GG	GG
AA	GG	AA	AA	AA	GG	AA	AA	AA
GG	GG	GG	GG	GG	GG	GG	GG	GG
CC	TT	CC	CC	CC	TT	CC	CC	CC

IRIS_313-1	IRIS_313-1	IRIS_313-1	IRIS_313-1	IRIS_313-1	IRIS_313-1	IRIS_313-1	IRIS_313-1	IRIS_313-1
CC	TT	TT	TT	TT	TT	CC	TT	TT
GG	GG	GG	GG	GG	GG	GG	GG	GG
TT	AA	AA	AA	AA	AA	TT	AA	AA
CC	CC	CC	CC	CC	CC	CC	CC	CC
AA	GG	GG	GG	GG	GG	AA	GG	GG
GG	AA	AA	NN	NN	AA	GG	AA	AA
GG	GG	GG	NN	NN	GG	GG	GG	GG
TT	CC	CC	CC	CC	CC	TT	CC	CC

IRIS_313-1	IRIS_313-1	IRIS_313-1	IRIS_313-1	IRIS_313-1	IRIS_313-1	IRIS_313-1	IRIS_313-1	IRIS_313-1
TT	TT	TT	CC	TT	TT	CC	CC	CC
GG	GG	GG	GG	GG	GG	GG	GG	GG
AA	AA	AA	TT	AA	AA	TT	TT	TT
CC	CC	CC	CC	CC	CC	CC	CC	CC
GG	GG	GG	AA	GG	GG	AA	AA	AA
AA	AA	AA	GG	AA	AA	GG	GG	GG
GG	GG	GG	GG	GG	GG	GG	GG	GG
CC	CC	CC	TT	CC	CC	TT	TT	TT

IRIS_313-1	IRIS_313-1	IRIS_313-1	IRIS_313-1	IRIS_313-1	IRIS_313-1	IRIS_313-1	IRIS_313-1	IRIS_313-1
TT	CC	CC	CC	CC	TT	TT	CC	CC
GG	GG	GG	GG	GG	GG	GG	GG	GG
AA	TT	TT	TT	TT	AA	AA	TT	TT
CC	CC	CC	CC	CC	CC	CC	CC	CC
GG	AA	AA	AA	AA	GG	GG	AA	AA
AA	GG	GG	GG	GG	AA	AA	GG	GG
GG	GG	GG	GG	GG	GG	GG	GG	GG
CC	TT	TT	TT	TT	CC	CC	TT	TT

IRIS_313-1	IRIS_313-1	IRIS_313-1	IRIS_313-1	IRIS_313-1	IRIS_313-1	IRIS_313-1	IRIS_313-1	IRIS_313-1
TT	CC	CC	CC	CC	CC	CC	CC	CC
GG	GG	GG	GG	GG	GG	GG	GG	GG
AA	TT	TT	TT	TT	TT	TT	TT	TT
NN	CC	CC	CC	CC	CC	CC	CC	CC
GG	AA	AA	AA	AA	AA	AA	AA	AA
AA	GG	GG	GG	GG	GG	GG	GG	GG
GG	GG	GG	GG	GG	GG	GG	GG	GG
CC	TT	TT	TT	TT	TT	TT	TT	TT

IRIS_313-1	IRIS_313-1	IRIS_313-1	IRIS_313-1	IRIS_313-1	IRIS_313-1	IRIS_313-1	IRIS_313-1	IRIS_313-1
CC	CC	CC	CC	CC	CC	CC	TT	TT
GG	GG	GG	GG	GG	GG	GG	GG	GG
TT	TT	TT	TT	TT	TT	TT	AA	AA
CC	CC	CC	CC	CC	CC	CC	CC	CC
AA	AA	AA	AA	AA	AA	AA	GG	GG
GG	GG	GG	GG	GG	GG	GG	AA	AA
GG	GG	GG	GG	GG	GG	GG	GG	GG
TT	TT	TT	TT	TT	TT	TT	CC	CC

IRIS_313-1	IRIS_313-1	IRIS_313-1	IRIS_313-1	IRIS_313-1	IRIS_313-1	IRIS_313-1	IRIS_313-1	IRIS_313-1
TT	TT	TT	TT	TT	TT	TT	TT	CC
GG	GG	GG	GG	GG	GG	GG	TT	GG
AA	AA	AA	AA	AA	AA	AA	AA	TT
CC	CC	CC	CC	CC	CC	CC	TT	CC
GG	GG	GG	GG	GG	GG	GG	GG	AA
AA	AA	AA	AA	AA	AA	AA	GG	GG
GG	GG	GG	GG	GG	GG	GG	GG	GG
CC	CC	CC	CC	CC	CC	CC	CC	TT

IRIS_313-1	IRIS_313-1	IRIS_313-1	IRIS_313-1	IRIS_313-1	IRIS_313-15910_IRIS_313-15910
CC	TT	CC	TT	TT	CC
GG	GG	GG	TT	TT	GG
TT	AA	TT	AA	AA	TT
CC	CC	CC	TT	TT	CC
AA	GG	AA	GG	GG	AA
GG	AA	GG	GG	GG	GG
GG	GG	GG	GG	GG	GG
NN	CC	TT	CC	CC	TT
